# Never the Two Shall Mix: Robust Indel Markers to Ensure the Fidelity of Two Pivotal and Closely-Related Accessions of *Brachypodium distachyon*

**DOI:** 10.3390/plants8060153

**Published:** 2019-06-06

**Authors:** Rhoda A. T. Brew-Appiah, Luigi M. Peracchi, Karen A. Sanguinet

**Affiliations:** Department of Crop and Soil Sciences, Washington State University, Pullman, WA 99164-6420, USA; brewappr@wsu.edu (R.A.T.B.-A.); luigi.peracchi@wsu.edu (L.M.P.)

**Keywords:** *Brachypodium distachyon*, Bd21, Bd21-3, indels

## Abstract

*Brachypodium distachyon* is an established model for monocotyledonous plants. Numerous markers intended for gene discovery and population genetics have been designed. However to date, very few indel markers with larger and easily scored length polymorphism differences, that distinguish between the two morphologically similar and highly utilized *B. distachyon* accessions, Bd21, the reference genome accession, and Bd21-3, the transformation-optimal accession, are publically available. In this study, 22 indel markers were designed and utilized to produce length polymorphism differences of 150 bp or more, for easy discrimination between Bd21 and Bd21-3. When tested on four other *B. distachyon* accessions, one case of multiallelism was observed. It was also shown that the markers could be used to determine homozygosity and heterozygosity at specific loci in a Bd21 x Bd3-1 F2 population. The work done in this study allows researchers to maintain the fidelity of Bd21 and Bd21-3 stocks for both transgenic and nontransgenic studies. It also provides markers that can be utilized in conjunction with others already available for further research on population genetics, gene discovery and gene characterization, all of which are necessary for the relevance of *B. distachyon* as a model species.

## 1. Introduction

Monocots are considered to be a highly valuable clade of plants. These organisms, such as orchids, lilies, bananas and oil palm, from the families Orchidaceae, Liliaceae, Musaceae and Arecaceae, respectively, have aesthetic, nutritional and medicinal uses, and therefore command a niche market with substantial economic value [1,2,3,4,5]. Another monocotyledonous family with a wide geographic range, used for food, feed, forage and fuel is the Poaceae (grass family) [6,7,8,9]. Within the grass family, the Triticeae tribe is well known for its significant contribution to global food security [10,11]. In order for Triticeae production to keep pace with or exceed demand, the continued exploration of critical developmental pathways and mechanisms for stress tolerance and yield is imperative [12,13,14,15,16,17,18].

This research can be accelerated with the use of model systems. As defined by Leonelli and Ankeny [19], a model organism is used to represent a larger group of species in order to investigate several pathways (genetic, developmental, physiological, evolutionary and ecological), and reach conclusions that can be extrapolated to the species in the group. To this end, the model organism must be small in size, be very fertile, have a rapid life cycle, be easy and cheap to maintain, and be amenable to genetic modification. In addition, infrastructure such as stock centers, easily accessible databases, and rules and regulations governing the utilization of standardized materials, must be in place to foster a multidisciplinary culture of collaboration that maintains the value of the chosen model organism [19]. In flowering plants, the dicotyledonous model organism *Arabidopsis thaliana* is the most studied, generating an unparalleled plethora of data and publications that have been extremely useful for research in numerous other plant species [20,21]. Despite these advances, there are unavoidable limitations in *A. thaliana* aptly summarized by Brutnell et al. [22]; (1) Species-specific developmental phenomena such as C4 photosynthesis, root nodulation or seed starch accumulation are nonexistent in *A. thaliana*, and therefore alternatives are necessary for studying these pathways. (2) Although certain pathways, such as those involved in flowering, are common across the land plants, and have been extensively studied in *A. thaliana*, the translation of this research to monocotyledonous plants can be problematic. For example, factors that promote flowering in *A. thaliana* inhibit the same process in the monocot *Oryza sativa* [22,23]. The dire need to bridge this gap, as well as the recognition of extensive chromosomal synteny in the grasses has spurred exploration into monocot model systems [22,24,25].

Over the years, the grass species rice, maize and barley have all been presented as alternative monocotyledonous model systems for some of the following reasons; a manageable genome size, a sequenced genome, relative ease of transformation, availability of functional genomic tools, and conserved developmental pathways or close evolutionary relationships that allow for translatable research to be conducted [12,22,26,27,28,29,30,31,32,33,34,35,36]. However, factors such as distinct differences in physiology, laborious growth requirements, large statures, slow life cycles, and a lack of feasibility, especially in geographic regions without the requisite cost-prohibitive infrastructure, have precluded these same species from having the long term impact of *A. thaliana* [22,34,35,36]. Recently, a C3 temperate grass *Brachypodium distachyon*, has emerged as a viable model system for the Triticeae species critical for global food security [22,35,36,37]. This temperate grass embodies all the advantages and none of the disadvantages of previously proposed monocot model systems [35,37,38,39]. This small, self-fertile diploid grass (2n=2x=10) has a small, fully sequenced genome (272 Mb), which can be genetically modified using standardized protocols, and has a substantial body of germplasm, genomic resources and publically accessible databases [22,35,36,37,38,39]. *B. distachyon* is undomesticated and highly syntenous with wheat, barley, rice and maize, leading to the acceleration of genomic studies, as well as the identification of genes involved in domestication [35,36]. To date, accessions of this model have been used in numerous transgenic and nontransgenic studies, including disease resistance and pathosystem identification, flowering, generation of transposon lines, cell wall development, drought tolerance and root development [37,40]. Although, both Bd21, the reference genome accession, and Bd21-3, are distinct inbred lines from the same germplasm source (PI 254867) and are transformable, Bd21-3 has higher transformation efficiencies, and has been used to generate knock-out lines for the *Brachypodium* scientific community [37,41,42,43]. In addition, many of these investigations have utilized multiple other *B. distachyon* accessions, as well as markers developed for the discrimination and identification of genetic variation within the *Brachypodium* genus [44,45,46,47,48,49,50,51].

The multitude of marker types necessitates the use of diverse visualization methods which can depend on codominance as well as loci copy number [52]. In this regard, indel markers are one of the simplest markers to score, as they can be codominant over a single locus, and are cost effective due to the simple method of visualization (PCR followed by ethidium bromide staining on an agarose gel) [52]. Although indel markers have been used in *B. distachyon* for studies ranging from population genetics to gene discovery, it must be noted that in many of these cases, the size of the indels can be small, and might require resolution with high percentage agarose gels [44,53,54]. The impressive body of work done on whole genome analysis of *B. distachyon* accessions has revealed areas of the genome with large indels among different accessions, as well as smaller indels (less than 50 bp) between Bd21 and Bd21-3 [46,51]. Morphologically, Bd21 and Bd21-3 are very similar, and yet developmental disparities, such as heading dates and photoresponses, are evident [43,55,56,57,58]. Given that environmental fluctuations can lead to unpredictability in flowering and heading dates, a more reliable method is needed for routine discrimination between these two closely related accessions, in order to optimize transformation efficiency [58,59,60]. While leveraging the small indels found by previous researchers can be useful in differentiating between Bd21 and Bd21-3, the process can be expedited with larger amplicon size variations which are easier to score. This has been shown in Arabidopsis, where Indel Group in Genomes (IGG) markers have been utilized for selectivity between Arabidopsis ecotypes Columbia (Col-0) and Landsberg *erecta* (L*er*-0) [52]. IGG markers differ from indels because the former, predominantly located upstream and downstream of the translation start and stop sites, can contain several indels (called Indel Groups) with cumulative lengths of 1500 bp or more. This increases the resolution of genotyping due to larger amplicon size differences easily scored with lower percentage agarose gels [52].

In the current study, we leveraged the availability of the genomes of Bd21 and Bd21-3, the knowledge that indels were available to discriminate between these closely related accessions, as well as the demonstration of larger indels in Arabidopsis and *B. distachyon* [37,51,52], to develop 22 large indel markers upstream and downstream of designated genes, for an efficient discrimination between Bd21 and Bd21-3. There was also one incidence of multiallelism among the *B. distachyon* accessions tested. The markers developed will ensure that researchers can easily and efficiently maintain stocks of Bd21 and Bd21-3 for transgenic studies, as well as aid in genotyping the progeny of biparental crosses.

## 2. Results

### 2.1. Identification of Indels or Indel Groups and Marker Development From Brachypodium distachyon Putative Orthologs of Arabidopsis thaliana Genes

Putative *B. distachyon* orthologs of *A. thaliana* genes were used to generate 22 indel markers from all five chromosomes for easy discrimination between Bd21 and Bd21-3 (Table 1 and Table S1). The only exception was BdindelWSU_8 obtained from one of the Bd21 candidate genes (*Bradi3g00757*) listed by Cui et al. [46], and its Bd21-3 ortholog from the Phytozome database (Version 12.1.6, default settings: https://phytozome.jgi.doe.gov/pz/portal.html). Alignments of upstream and downstream regions of the 22 curated genes yielded single or cumulative gaps of 150 nucleotides or more (Figure S1). Primers were then successfully designed, flanking these regions, and then in silico PCR was used to predict the amplicon sizes of the two accessions (Table 1, Figure S1). Out of the 22 markers, 13 were designed from upstream regions, and 9 were designed from downstream regions of the designated genes (Table S1). In addition, for half of these markers, the predicted amplicon size was greater in Bd21 than in Bd21-3 (Table 1 and Table S1).

### 2.2. Marker Assessment on Six Brachypodium distachyon Accessions and F2 Population

**Figure 1:**
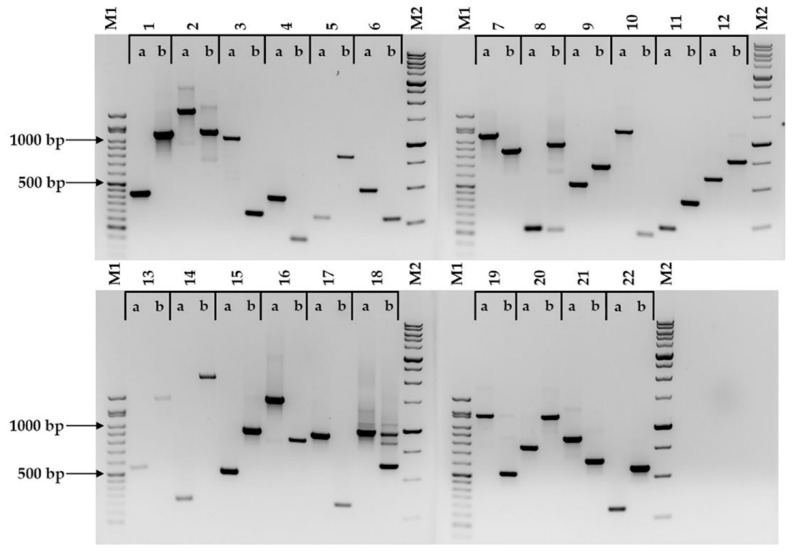
22 markers (BdindelWSU_1 to BdindelWSU_22) that discriminate between the two closely related *Brachypodium distachyon* accessions Bd21 (a) and Bd21-3 (b). The amplicons were visualized on a 1.5% agarose gel stained with ethidium bromide. M1 represents the GoldBio^®^ 50 bp DNA ladder, and M2 represents the GoldBio^®^ 1 kb PLUS^™^ DNA ladder.

**Figure 2:**
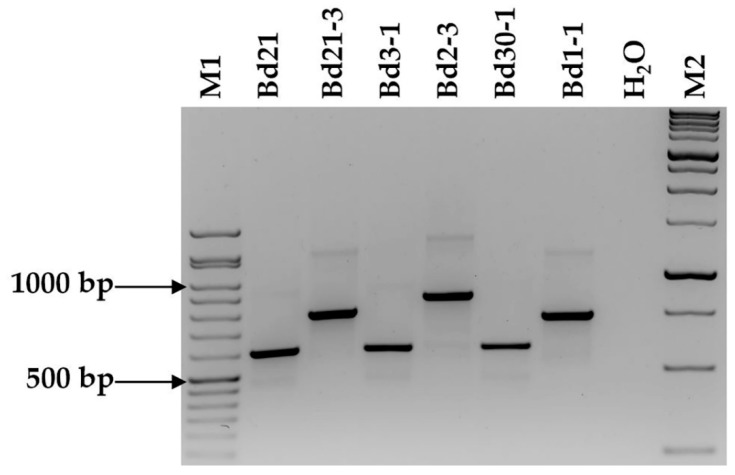
Multiallelism observed with the marker BdindelWSU_12 for six *Brachypodium distachyon* accessions. The amplicons were visualized on a 2% agarose gel stained with ethidium bromide. M1 represents the GoldBio^®^ 50 bp DNA ladder and M2 represents the GoldBio^®^ 1 kb PLUS^™^ DNA ladder.

**Figure 3:**
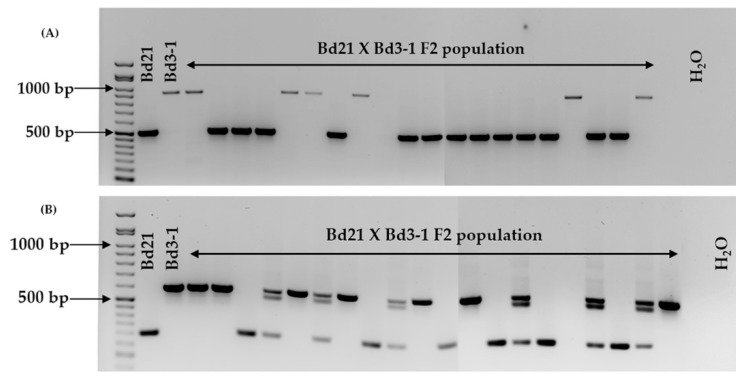
BdindelWSU_15 (**A**) and BdindelWSU_22 (**B**) tested on a Bd21 x Bd3-1 F2 population. The amplicons were visualized on a 1.5% agarose gel stained with ethidium bromide. The GoldBio^®^ 50 bp DNA ladder was used for amplicon size determination.

All 22 markers tested produced amplicons that allowed for the discrimination between Bd21 and Bd21-3 (Figure 1, Table 2 and Table S2). When tested against four other *B. distachyon* accessions, in most cases, the amplicon sizes followed those displayed by Bd21 or Bd21-3 (Table 2, Figure S2). There was one exception, (BdindelWSU_12), where multiallelism was observed within the six accessions tested (Figure 2 and Figure S2, Table 2 and Table S2). Two markers (BdindelWSU_15 and BdindelWSU_22) were tested on a Bd21 x Bd3-1 F2 population to show homozygosity and heterozygosity, highlighting the efficiency of the markers designed (Figure 3).

## 3. Discussion

Since the inception of *B. distachyon* as a model system, many tools including markers have been developed to aid in research and discovery. Markers are available for the discrimination of different species in the genus *Brachypodium*, and within multiple populations of the same species [44,48,49,50,54]. In many cases, the length polymorphisms require the use of high percentage agarose gels. Following the work that has been done with indel markers in Arabidopsis as well as the evidence provided for larger indels in the *Brachypodium* genus [51,52], the current study aimed to design indel markers with sufficient length polymorphisms easily visualized on low percentage agarose gels, for the two highly utilized accessions needed for functional gene characterization, Bd21 and Bd21-3.

The parameters for these indel markers were as follows: (1) The markers had to be upstream or downstream of a designated gene. This was done to avoid designing primers from gene families which could lead to multiple amplification products. Although there is literature showing that an intron can be used to discriminate within the *Brachypodium* genus [50], the primers were designed upstream and downstream of the start and stop codons to increase the likelihood of robust and unequivocal results. This approach also increased our understanding of promoter region differences between Bd21 and Bd21-3 that could be exploited in future gene expression variation studies. (2) The markers needed to amplify in both accessions in order to avoid diagnostic ambiguity created by reaction failure. (3) The predicted amplicon sizes were between 150 bp and 2000 bp, a range easily covered by a cost-effective *Taq* polymerase routinely used in PCR. This would potentially increase the utility of the markers by multiple research groups. (4) The amplicon size difference generated by each marker had to be 150 bp or more for easy discrimination on a 1.5% agarose gel.

All 22 markers tested showed discrimination between Bd21 and Bd21-3 (Figure 1). With the exception of one case (BdindelWSU_3), the predicted amplicon sizes were obtained (Table 1 and Table 2). With regard to the deviation from the expected amplicon size, this was a nonissue, as distinct and sizeable length polymorphism differences were still observed between Bd21 and Bd21-3, thus fulfilling the primary objective of the study. In some cases, other amplicons besides the ones predicted were noted (Figure 1, Figure S2, Table S2). This observation did not affect the primary aim of the study, as very distinct patterns could be observed between Bd21 and Bd21-3. It must be noted that these markers are yet to be tested on different populations of the same accession from a wide geographic range, or on other species in the *Brachypodium* genus. The expectation is that such analyses would yield multiple alleles, as has been shown by other studies [48,50,54]. The in silico sequence analyses showed that some of the regions selected for marker development were distant from areas that could reasonably be considered to be the promoter region for the designated gene (within 2 Kb upstream of the translation start site), and may in fact have been part of the structure of genes in a different orientation (Figure S1). Out of the 22 pairs of sequences aligned, 13 were upstream of the start codons and 12 of these showed indels within 2 Kb of the designated gene, and may help to explain expression differences between these two accessions (Table S1, Figure S1). Conducting the marker development for discrimination between Bd21 and Bd21-3 on a larger scale with higher throughput resources, could show more promoter regions with indels that can explain transcriptomic differences between these two accessions, and contribute clues regarding observed phenotypic differences (e.g., transformation efficiency) [37,43]. This approach has merit as genome-wide scans have shown that single nucleotide polymorphisms can give clues about the environmental adaptation of genes [61,62]. Our approach could also help with comparative transcriptomics between Bd21 and Bd21-3 by ensuring that the starting plant materials used for the analyses are accurate, and that the data reported in the scientific literature are precise.

When the markers were screened on four other *B. distachyon* accessions, one case of multiallelism (BdindelWSU_12) was observed (Figure 2, Table 2). Locus homozygosity and heterozygosity was also noted when selected markers (BdindelWSU_15 and BdindelWSU_22) were used on a Bd21 x Bd3-1 F2 population (Figure 3). The observed multiallelism in different accessions indicates that our approach in conjunction with other markers developed can be utilized on a larger scale to verify accessions slated for biparental crosses. Broadening the scope of this study would add to the body of marker types available for population genetics and gene discovery. The markers developed ensure that researchers are able to optimize their *B. distachyon* transformation pipelines by actively selecting for the more efficient accession, Bd21-3. Researchers will therefore protect the accuracy of the transgenic data generated. For example, phenotypes (e.g., early or late flowering) obtained from overexpression and knockdown of a gene can be accurately reported as being a result of the transgenic event rather than cross-contamination of the accessions used for the independent transformation events. Researchers can also ensure the generation of transgenic or gene-edited lines in a genetic background consistent with those of publicly available stocks of T-DNA lines. This prevents false positives and negatives as well as the loss of valuable time and resources.

There is also the potential for cross-contamination during material transfer from one researcher to another. Rather than a reliance on unpredictable and environment-dependent morphological differences, our markers give a quick way to ensure that the right accession is being given or received. There is a very valid argument of simply obtaining Bd21 and Bd21-3 from seed banks. However some seed banks have a policy of denying repeated requests for the same material. It is the responsibility of the researcher to propagate and maintain the seed stocks, and the markers developed in the current study aid with fidelity during cultivation and stock maintenance. In addition, seed viability can decrease over time, and the markers developed in the current study help researchers maintain the desired accession fidelity long after the original seed material has been completely exhausted via repeated propagation.

## 4. Materials and Methods

### 4.1. Identification of Indels or Indel Groups and Primer Design

The protein sequences of curated Arabidopsis genes were used in a Basic Local Alignment Search Tool for proteins (BLASTP) in the Phytozome database (Version 12.1.6, default settings: https://phytozome.jgi.doe.gov/pz/portal.html) to obtain putative orthologs in the *B. distachyon* accessions Bd21 (Bd21, JGI v3.0 assembly, JGI v3.1 annotation) and Bd21-3 (Bd21-3 v1.1 DOE-JGI, http://phytozome.jgi.doe.gov/). Up to 10,000 bp upstream (defined as the region before the start codon) and 10,000 bp downstream (defined as region after the stop codon) of the Bd21 and Bd21-3 genes were used in alignments in the program Serial Cloner (Version 2.6.1: http://serialbasics.free.fr/Serial_Cloner.html). Using the Primer3Plus software [63], primers were designed from conserved regions flanking one or more large indel sites. The amplicon sizes were predicted with Serial Cloner (Version 2.6.1: http://serialbasics.free.fr/Serial_Cloner.html) using the PCR tool.

### 4.2. Marker Validation on Brachypodium Distachyon Accessions

The *B. distachyon* accessions Bd21 (W6 36678), Bd21-3 (W6 39233), Bd3-1 (W6 46203), Bd2-3 (W6 46202), Bd30-1 (W6 46206) and Bd1-1 (W6 46201) were obtained from the United States Department of Agriculture (USDA) National Plant Germplasm System (NPGS) (https://www.ars-grin.gov/npgs/) and propagated for seed increase ([Bd21, Bd21-3, Bd3-1]: 22 °C 16 h, 18 °C 8 h, light intensity 200 µmol/sm^2^, 16 h light/8 h dark photoperiod; [Bd2-3, Bd30-1]: 4 °C 24 h, light intensity 200 µmol/sm^2^, 12 h light/12 h dark photoperiod, for 3 weeks, followed by 22 °C, 16 h, 18 °C 8 h, light intensity 200 µmol/sm^2^, 16 h light/8 h dark photoperiod; [Bd1-1]: 4 °C 24 h, light intensity 200 µmol/sm^2^, 12 h light/12 h dark photoperiod, for 10 weeks, followed by 22 °C, 16 h, 18 °C 8 h, light intensity 200 µmol/sm^2^, 16 h light/8 h dark photoperiod). Genomic DNA (gDNA) was extracted from the leaf tissue as follows: The leaves were homogenized under liquid nitrogen and then added to 800 µL of the extraction buffer (200 mM Tris [pH 8.5], 200 mM NaCl, 50 mM EDTA, 1% SDS), mixed and centrifuged at 18,000 × g after incubation at 65 °C for 30 min. 0.3 × volume of cold 5 M Potassium acetate was added to the supernatant, mixed and incubated on ice for 10 min. This was centrifuged (18,000 × g, room temperature) and a phenol chloroform isoamyl (25:24:1) extraction was performed, followed by a chloroform isoamyl (24:1) extraction. The gDNA was reprecipitated with 0.7 × volume of 100% Isopropanol, and resuspended in nuclease-free water to create 100 ng/µL stocks. For the Bd21 x Bd3-1 F2 population, the gDNA was extracted as previously described [64]. PCR was performed in a 20 µL volume as follows using an Eppendorf Mastercycler^®^ nexus GX2: 5 ng/µL gDNA for accessions and 2 µL for Bd21 x Bd3-1 F2 population, 0.5 µM of each primer, 0.25 mM dNTPs, 1.66 M Trehalose dihydrate, 2 µL 10x Econotaq buffer, 0.025 U/µL Econotaq^®^. The program used was; 1 cycle at 94 °C for 2 min, 35 cycles of 30 s at 94 °C, 30 s at 65 °C and 2 min at 72 °C. This was followed by a final extension step at 72 °C for 5 min. The PCR amplicons were resolved on a 1.5% gel or 2% gel (in the case of multiallelism) and visualized using the Bio-Rad ChemiDoc^™^ Touch Imaging System under the ethidium bromide, faint band setting. The GoldBio^®^ 50 bp DNA ladder and 1 Kb PLUS^™^ DNA ladder were used to estimate the amplicon sizes. For the four accessions (Bd3-1, Bd2-3, Bd30-1, Bd1-1) where no predictions were made before the markers were tested, the size determination was dependent upon comparison to the results of Bd21 or Bd21-3, as well as on the DNA ladders used.

## 5. Conclusions

There are many markers that have been developed for both population genetics and gene discovery that can distinguish between species in the *Brachypodium* genus. Generally the indel markers that have been developed tend to have small amplicon size differences, and very few of them have been reported to specifically distinguish between the transformation-optimal accession Bd21-3 and the reference genome accession Bd21. The current study sought to increase the knowledge in this area, and to this end, 22 indel markers were developed to distinguish between Bd21 and Bd21-3 with an amplicon size difference minimum of 150 bp. In addition, an incidence of multiallelism was observed, indicating that the approach utilized can be translated to other brachypodium accessions in the same species. The markers developed provide a quick, cost-efficient and easy way to score samples and accurately maintain the fidelity of Bd21 and Bd21-3, which are extremely critical to population genetics, evolutionary studies and functional gene characterization, all of which ensure that *B. distachyon* maintains its relevance as a model monocot.

## Figures and Tables

**Table 1:** Length polymorphism markers predicted for *Brachypodium distachyon* accessions Bd21 and Bd21-3. The sequences were obtained from the Phytozome database (Version 12.1.6), primers were designed with the Primer3Plus software and amplicon sizes were predicted with the PCR tool in the Serial Cloner software (Version 2.6.1).

Marker Name	Chromosome Location	Expected Size		Amplicon Size Difference	Forward Primer (5′ to 3′)	Reverse Primer (5′ to 3′)
		Bd21	Bd21-3			
BdindelWSU_1	1	419	1065	646	AAGCGCATCCTTCACGCTTTC	GTGCAGAGCTGCAGAGTAGAGATTAG
BdindelWSU_2	1	1522	1096	426	GGAACCTGTGTTCGGACTCG	TGCAGGAACGGAGAAATGGA
BdindelWSU_3	1	992	287	705	CCGTATCAGAGCACGCCAGAAT	CTTGGGAGGAACTAATACACCAGATCG
BdindelWSU_4	1	402	159	243	CGCCCTTCTTTTGAAACCCAAA	TGAGCACTTGACAGTTTTTGGTGA
BdindelWSU_5	2	288	826	538	TCTCCTTGAAATTGCATGTTGGA	CCTGACAGTGGGCCATAGCA
BdindelWSU_6	2	490	285	205	CCCCCATGGTTGAATGGTTC	CACATCACGTCGCACCAGAA
BdindelWSU_7	2	1103	866	237	CTGCAGAGCTTCAGCCTTGG	TTGCCAGCGGAACGATGTTA
BdindelWSU_8	3	213	940	727	AAGGCCTCCGCCAAGCTATC	GACCTCGGGACGCGACAG
BdindelWSU_9	3	509	683	174	CAAGCAAAAAGCCTTGGAGCA	CACACCATACAAAAACGTCCAGGAT
BdindelWSU_10	3	1160	206	954	TTCAGCGCTTTCGGTACTTTG	GAGATGCGATGGGGAAGCAT
BdindelWSU_11	3	246	410	164	TGAGAGCACCGGACAGGACA	CTGGCTCGGATTGGATTGATTTTC
BdindelWSU_12	3	600	782	182	GGTTAGCGAGGCGTCAATGG	CCCATGGACCCATGCATATTG
BdindelWSU_13	3	547	1388	841	CAGATCTGGGGGCTCGTTGA	CCAATCAAATTGCGGAGCTG
BdindelWSU_14	3	317	1908	1591	GGGACCCCATGTTACCCTGA	CGATGCCAATCCAAGAGACG
BdindelWSU_15	4	500	906	406	AAACAGCAAGGTTGGCCTTATCC	GGGGTCAGCAATTGAATGTGT
BdindelWSU_16	5	1413	827	586	ATGCCGGCTTAGCGTTAGTTG	CTGACCCTTGTACACACTCGTAGCA
BdindelWSU_17	5	900	323	577	CGGCCCTCCAGACATTGTTC	TGCATCCCCTATCTTGGACGA
BdindelWSU_18	5	987	619	368	CGTATCAATCACGATCGGTCCA	ATAGGCAGCAGCGTGGTGGT
BdindelWSU_19	5	1170	522	648	CGATACAAATTCATTTGACGGGTGT	CGCGGGAAACGTCTTTACAA
BdindelWSU_20	5	775	1165	390	CAGAACTACGGCAGTTAATTTGGATTG	CGGGATTATTTGCGCGAGA
BdindelWSU_21	5	862	632	230	CAACCCATCCGCAGACACAC	TGGATGAAGCCCATTGCAGA
BdindelWSU_22	5	300	569	269	GGTCAGTGCGATGTGGTGTTTC	GTGGGAATCCATCTGCTTTGTTCTT

**Table 2:** Nucleotide sizes of major and discriminating amplicons obtained for six *Brachypodium distachyon* accessions using 22 designed indel markers. Values shown are the main bands used for accession discrimination under the conditions tested. * Indicates amplicon size larger than predicted.

Marker	Bd21	Bd21-3	Bd3-1	Bd2-3	Bd30-1	Bd1-1
BdindelWSU_1	419	1065	419	419	419	1065
BdindelWSU_2	1522	1096	1096	1522	1096	1522
BdindelWSU_3	1000 *	300 *	1000	300	300	1000
BdindelWSU_4	402	159	159	159	159	159
BdindelWSU_5	288	826	826	826	826	288
BdindelWSU_6	490	285	285	285	285	490
BdindelWSU_7	1103	866	1103	1103	1103	866
BdindelWSU_8	213	940	213	940	940	213
BdindelWSU_9	509	683	509	509	683	683
BdindelWSU_10	1160	206	1160	1160	1160	1160
BdindelWSU_11	246	410	246	246	246	410
BdindelWSU_12	600	782	600	900	600	750
BdindelWSU_13	547	1388	547	1388	547	1388
BdindelWSU_14	317	1908	1908	1908	317	1908
BdindelWSU_15	500	906	906	500	500	906
BdindelWSU_16	1413	827	1413	827	827	827
BdindelWSU_17	900	323	900	323	323	323
BdindelWSU_18	987	619	619	619	619	987
BdindelWSU_19	1170	522	522	522	522	1170
BdindelWSU_20	775	1165	775	775	1165	775
BdindelWSU_21	862	632	862	862	632	862
BdindelWSU_22	300	569	569	569	300	569

## Supplementary Materials

The following are available online at https://www.mdpi.com/2223-7747/8/6/153/s1: Figure S1: Bd21 and Bd21-3 sequences used for alignments and detection of indels and flanking homologous regions.; Figure S2: Observed amplicon sizes of indel markers on six *Brachypodium distachyon* accessions.; Table S1: Complete information about *Brachypodium distachyon* genes used in the study.; Table S2: Observed amplicon sizes of the markers tested on six *Brachypodium distachyon* accessions.

## References

[1] Hinsley A., de Boer H.J., Fay M.F., Gale S.W., Gardiner L.M., Gunasekara R.S., Kumar P., Masters S., Metusala D., Roberts D.L. (2018). A review of the trade in orchids and its implications for conservation. Bot. J. Linn. Soc..

[2] Munafo J.P., Gianfagna T.J. (2015). Chemistry and biological activity of steroidal glycosides from the *Lilium* genus. Nat. Prod. Rep..

[3] Suh J.K., Wu X.W., Lee A.K., Roh M.S. (2013). Growth and flowering physiology, and developing new technologies to increase the flower numbers in the *Genus Lilium*. Hortic. Environ. Biotechnol..

[4] Paul J.Y., Harding R., Tushemereirwe W., Dale J. (2018). Banana21: From gene discovery to deregulated golden bananas. Front. Plant Sci..

[5] Masani M.Y.A., Izawati A.M.D., Rasid O.A., Parveez G.K.A. (2018). Biotechnology of oil palm: Current status of oil palm genetic transformation. Biocatal. Agric. Biotechnol..

[6] Linder H.P., Lehmann C.E.R., Archibald S., Osborne C.P., Richardson D.M. (2018). Global grass (Poaceae) success underpinned by traits facilitating colonization, persistence and habitat transformation. Biol. Rev. Camb. Philos. Soc..

[7] Allwright M.R., Taylors G. (2016). Molecular breeding for improved second generation bioenergy crops. Trends Plant Sci..

[8] Yang J.D., Udvardi M. (2018). Senescence and nitrogen use efficiency in perennial grasses for forage and biofuel production. J. Exp. Bot..

[9] Foley J.A., Ramankutty N., Brauman K.A., Cassidy E.S., Gerber J.S., Johnston M., Mueller N.D., O’Connell C., Ray D.K., West P.C. (2011). Solutions for a cultivated planet. Nature.

[10] Bolser D.M., Kerhornou A., Walts B., Kersey P. (2015). Triticeae resources in ensembl plants. Plant Cell Physiol..

[11] Mochida K., Shinozaki K. (2013). Unlocking triticeae genomics to sustainably feed the future. Plant Cell Physiol..

[12] Gurel F., Ozturk Z.N., Ucarli C., Rosellini D. (2016). Barley genes as tools to confer abiotic stress tolerance in crops. Front. Plant Sci..

[13] Cantalapiedra C.P., Garcia-Pereira M.J., Gracia M.P., Igartua E., Casas A.M., Contreras-Moreira B. (2017). Large differences in gene expression responses to drought and heat stress between elite barley cultivar Scarlett and a Spanish landrace. Front. Plant Sci..

[14] Brew-Appiah R.A.T., York Z.B., Krishnan V., Roalson E.H., Sanguinet K.A. (2018). Genome-wide identification and analysis of the *ALTERNATIVE OXIDASE* gene family in diploid and hexaploid wheat. PLoS ONE.

[15] Brew-Appiah R.A.T., Sanguinet K.A. (2018). Considerations of AOX functionality revealed by critical motifs and unique domains. Int. J. Mol. Sci..

[16] Alptekin B., Langridge P., Budak H. (2017). Abiotic stress miRNomes in the Triticeae. Funct. Integr. Genom..

[17] Galvez S., Merida-Garcia R., Camino C., Borrill P., Abrouk M., Ramirez-Gonzalez R.H., Biyiklioglu S., Amil-Ruiz F., Dorado G., Budak H. (2019). Hotspots in the genomic architecture of field drought responses in wheat as breeding targets. Funct. Integr. Genom..

[18] Tack J., Barkley A., Nalley L.L. (2015). Effect of warming temperatures on US wheat yields. Proc. Natl. Acad. Sci. USA.

[19] Leonelli S., Ankeny R.A. (2013). What makes a model organism?. Endeavour.

[20] Koornneef M., Meinke D. (2010). The development of Arabidopsis as a model plant. Plant J..

[21] Provart N.J., Alonso J., Assmann S.M., Bergmann D., Brady S.M., Brkljacic J., Browse J., Chapple C., Colot V., Cutler S. (2016). 50 years of Arabidopsis research: Highlights and future directions. New Phytol..

[22] Brutnell T.P., Bennetzen J.L., Vogel J.P. (2015). *Brachypodium distachyon* and *Setaria viridis*: Model genetic systems for the grasses. Annu. Rev. Plant Biol..

[23] Hayama R., Coupland G. (2004). The molecular basis of diversity in the photoperiodic flowering responses of arabidopsis and rice. Plant Physiol..

[24] Bennetzen J.L., Freeling M. (1993). Grasses as a single genetic system: Genome composition, collinearity and compatibility. Trends Genet..

[25] Freeling M. (2001). Grasses as a single genetic system. Reassessment 2001. Plant Physiol..

[26] Shimamoto K., Kyozuka J. (2002). Rice as a model for comparative genomics of plants. Annu. Rev. Plant Biol..

[27] Goff S.A., Ricke D., Lan T.H., Presting G., Wang R.L., Dunn M., Glazebrook J., Sessions A., Oeller P., Varma H. (2002). A draft sequence of the rice genome (*Oryza sativa* L. ssp *japonica*). Science.

[28] Yu J., Hu S.N., Wang J., Wong G.K.S., Li S.G., Liu B., Deng Y.J., Dai L., Zhou Y., Zhang X.Q. (2002). A draft sequence of the rice genome (*Oryza sativa* L. ssp *indica*). Science.

[29] Han B., Zhang Q.F. (2008). Rice genome research: Current status and future perspectives. Plant Genome.

[30] Schnable P.S., Ware D., Fulton R.S., Stein J.C., Wei F.S., Pasternak S., Liang C.Z., Zhang J.W., Fulton L., Graves T.A. (2009). The B73 maize genome: Complexity, diversity, and dynamics. Science.

[31] Beier S., Himmelbach A., Colmsee C., Zhang X.Q., Barrero R.A., Zhang Q.S., Li L., Bayer M., Bolser D., Taudien S. (2017). Construction of a map-based reference genome sequence for barley, *Hordeum vulgare* L.. Sci. Data.

[32] Colmsee C., Beier S., Himmelbach A., Schmutzer T., Stein N., Scholz U., Mascher M. (2015). BARLEX—The barley draft genome explorer. Mol. Plant.

[33] Szurman-Zubrzycka M.E., Zbieszczyk J., Marzec M., Jelonek J., Chmielewska B., Kurowska M.M., Krok M., Daszkowska-Golec A., Guzy-Wrobelska J., Gruszka D. (2018). HorTILLUS—A rich and renewable source of induced mutations for forward/reverse genetics and pre-breeding programs in barley (*Hordeum vulgare* L.). Front. Plant Sci..

[34] Chang C.R., Bowman J.L., Meyerowitz E.M. (2016). Field guide to plant model systems. Cell.

[35] Girin T., David L.C., Chardin C., Sibout R., Krapp A., Ferrario-Mery S., Daniel-Vedele F. (2014). *Brachypodium*: A promising hub between model species and cereals. J. Exp. Bot..

[36] Kellogg E.A. (2015). *Brachypodium distachyon* as a genetic model system. Annu. Rev. Genet..

[37] Scholthof K.B.G., Irigoyen S., Catalan P., Mandadi K.K. (2018). *Brachypodium*: A monocot grass model genus for plant biology. Plant Cell.

[38] Draper J., Mur L.A.J., Jenkins G., Ghosh-Biswas G.C., Bablak P., Hasterok R., Routledge A.P.M. (2001). *Brachypodium distachyon*. A new model system for functional genomics in grasses. Plant Physiol..

[39] Mur L.A.J., Allainguillaume J., Catalan P., Hasterok R., Jenkins G., Lesniewska K., Thomas I., Vogel J. (2011). Exploiting the brachypodium tool box in cereal and grass research. New Phytol..

[40] Gill U.S., Lee S., Jia Y.L., Mysore K.S. (2019). Exploring natural variation for rice sheath blight resistance in *Brachypodium distachyon*. Plant Signal. Behav..

[41] Bragg J.N., Wu J.J., Gordon S.P., Guttman M.E., Thilmony R., Lazo G.R., Gu Y.Q., Vogel J.P. (2012). Generation and characterization of the western regional research center brachypodium T-DNA insertional mutant collection. PLoS ONE.

[42] Hsia M.M., O’Malley R., Cartwright A., Nieu R., Gordon S.P., Kelly S., Williams T.G., Wood D.F., Zhao Y.J., Bragg J. (2017). Sequencing and functional validation of the JGI *Brachypodium distachyon* T-DNA collection. Plant J..

[43] Vogel J., Hill T. (2008). High-efficiency *Agrobacterium*-mediated transformation of *Brachypodium distachyon* inbred line Bd21-3. Plant Cell Rep..

[44] Vogel J.P., Tuna M., Budak H., Huo N.X., Gu Y.Q., Steinwand M.A. (2009). Development of SSR markers and analysis of diversity in Turkish populations of *Brachypodium distachyon*. BMC Plant Biol..

[45] Huo N.X., Garvin D.F., You F.M., McMahon S., Luo M.C., Gu Y.Q., Lazo G.R., Vogel J.P. (2011). Comparison of a high-density genetic linkage map to genome features in the model grass *Brachypodium distachyon*. Theor. Appl. Genet..

[46] Cui Y., Lee M.Y., Huo N.X., Bragg J., Yan L.J., Yuan C., Li C., Holditch S.J., Xie J.Z., Luo M.C. (2012). Fine mapping of the *Bsr1* barley stripe mosaic virus resistance gene in the model grass *Brachypodium distachyon*. PLoS ONE.

[47] Filiz E., Ozdemir B.S., Budak F., Vogel J.P., Tuna M., Budak H. (2009). Molecular, morphological, and cytological analysis of diverse *Brachypodium distachyon* inbred lines. Genome.

[48] Giraldo P., Rodriguez-Quijano M., Vazquez J.F., Carrillo J.M., Benavente E. (2012). Validation of microsatellite markers for cytotype discrimination in the model grass *Brachypodium distachyon*. Genome.

[49] Lopez-Alvarez D., Lopez-Herranz M.L., Betekhtin A., Catalan P. (2012). A DNA barcoding method to discriminate between the model plant *Brachypodium distachyon* and its close relatives *B. stacei* and *B. hybridum* (Poaceae). PLoS ONE.

[50] Contreras R., Figueiras A.M., Gallego F.J., Benavente E., Manzaneda A.J., Benito C. (2017). Neutral molecular markers support common origin of aluminium tolerance in three congeneric grass species growing in acidic soils. AoB Plants.

[51] Gordon S.P., Priest H., Marais D.L.D., Schackwitz W., Figueroa M., Martin J., Bragg J.N., Tyler L., Lee C.R., Bryant D. (2014). Genome diversity in *Brachypodium distachyon*: Deep sequencing of highly diverse inbred lines. Plant J..

[52] Toal T.W., Burkart-Waco D., Howell T., Ron M., Kuppu S., Britt A., Chetelat R., Brady S.M. (2016). Indel group in genomes (IGG) molecular genetic markers. Plant Physiol..

[53] Woods D.P., Ream T.S., Minevich G., Hobert O., Amasino R.M. (2014). PHYTOCHROME C is an essential light receptor for photoperiodic flowering in the temperate grass, *Brachypodium distachyon*. Genetics.

[54] Neji M., Guena F., Gordon S.P., Taamalli W., Vogel J.P., Ibrahim Y., Smaoui A.E., Abdelly C., Gandour M. (2016). Insertion/deletion markers for assessing the genetic variation and the spatial genetic structure of Tunisian *Brachypodium hybridum* populations. Recent Res. Sci. Technol..

[55] Cass C.L., Lavell A.A., Santoro N., Foster C.E., Karlen S.D., Smith R.A., Ralph J., Garvin D.F., Sedbrook J.C. (2016). Cell wall composition and biomass recalcitrance differences within a genotypically diverse set of *Brachypodium distachyon* inbred lines. Front. Plant Sci..

[56] Tyler L., Fangel J.U., Fagerstrom A.D., Steinwand M.A., Raab T.K., Willats W.G.T., Vogel J.P. (2014). Selection and phenotypic characterization of a core collection of *Brachypodium distachyon* inbred lines. BMC Plant Biol..

[57] Tran Q.-G., Han Y.-J., Hwang O.-J., Hoang Q.T.N., Kim J.-I. (2018). Exploring responses to light in the monocot model plant, *Brachypodium distachyon*. Korean J. Plant Res..

[58] Schwartz C.J., Doyle M.R., Manzaneda A.J., Rey P.J., Mitchell-Olds T., Amasino R.M. (2010). Natural variation of flowering time and vernalization responsiveness in *Brachypodium distachyon*. Bioenergy Res..

[59] Garvin D.F., Gu Y.Q., Hasterok R., Hazen S.P., Jenkins G., Mockler T.C., Mur L.A.J., Vogel J.P. (2008). Development of genetic and genomic research resources for *Brachypodium distachyon*, a new model system for grass crop research. Crop Sci..

[60] Ream T.S., Woods D.P., Schwartz C.J., Sanabria C.P., Mahoy J.A., Walters E.M., Kaeppler H.F., Amasino R.M. (2014). Interaction of photoperiod and vernalization determines flowering time of *Brachypodium distachyon*. Plant Physiol..

[61] Dell’Acqua M., Zuccolo A., Tuna M., Gianfranceschi L., Pe M.E. (2014). Targeting environmental adaptation in the monocot model *Brachypodium distachyon*: A multi-faceted approach. BMC Genom..

[62] Bourgeois Y., Stritt C., Walser J.C., Gordon S.P., Vogel J.P., Roulin A.C. (2018). Genome-wide scans of selection highlight the impact of biotic and abiotic constraints in natural populations of the model grass *Brachypodium distachyon*. Plant J..

[63] Untergasser A., Nijveen H., Rao X., Bisseling T., Geurts R., Leunissen J.A.M. (2007). Primer3Plus, an enhanced web interface to Primer3. Nucleic Acids Res..

[64] Edwards K., Johnstone C., Thompson C. (1991). A simple and rapid method for the preparation of plant genomic DNA for PCR analysis. Nucleic Acids Res..

