# Supplementary material for: Never the Two Shall Mix: Robust Indel Markers to Ensure the Fidelity of Two Pivotal and Closely-Related Accessions of Brachypodium distachyon
Source: Plants (Basel). 2019 Jun 6;8(6):153. doi: 10.3390/plants8060153 (PMC6630600; doi:10.3390/plants8060153)
Supplement: Supplementary file 1 [file plants-08-00153-s001.zip › Figure S1.pdf]

Figure S1. Bd21 and Bd21-3 sequences used for alignments and detection of indels and flanking homologous regions. Seq_1 or Sequence_1 refers to Bd21 and Seq_2 or Sequence_2 refers to Bd21-3. The alignments were done in with the Serial Cloner software (version 2.6.1). Green highlights are regions for forward primer design, red highlights are regions for reverse primer design. Black line indicates the first five nucleotides before the start codon (ATG), red line indicates the first five nucleotides after the stop codon.

BdindelWSU_1, DOWNSTREAM

>Bradi1g03290

ATGTCGAGGAGCTTCTGGATATGCTGCTTAAACGGCGACAGAACTCTGATGAGCCTTCGTTAACACTAGGACAAGAAAGGAAACATA
ATTGCATAAGAATTCTGAGTACAATTTGTATGGTTTGGTTTCCCCTAAACATCGTTTTCGTCGAGAATGTATTGCACAGCTGTGAGATT
AGAAGGCAAACAAATTAAGAACTTCAGAAGTGTTTCCCTCTGACAATGTGTGTGTTGCTTGTGATTCACAGCCTTGTGCATGCTCTCTT
GCTGCTCCAGTTTTCTTGAGCTGGGTGTTGAACGAAGATCACTCAGGATAGTTGCATTTGGTGTGTGAAGAGTTCAGTGTCCGCTCAA
GGCCAGTTGCCTGATGCGATCGTTTTAATAATAGTACATCTAAATGTCGCCTGTGAGGCAGTGACTTCAGTTTCTGTAAGCTGAGCGC
TTATTAAAAGGGCCTTCTGTGAACGGACTAGCCTTTGCTTCTTTCCGGGACGCTTTGACGGCGCCTACCTCTGCCGTTGCTGCTGGGG
CATTGATGCCGGCTTTGGGGGAGCCTGCCTCCTCCGGGTTTTCGCGTCCGCGTCTCAGGTCCGCCATCACTGCTGCTGCGCCAGGGTG
ACAGCTCGCCTCAGATCATTGATGACGATTGTTGGCAGTTGGCTCCGGGATCTGCTACTTAAGCAGCAGGCCTGGGGGAAGCTGAGC
TGAGGCAACCCGTCAAGAGCTCCCTTCTCCGCCGCCGCGTCTCGCTCCGGCTGCTCCTCCTCAGGCTCCTGCGCCCTGTTTCTTCTTCG
GTTATCTCCCCCGCTCATGGCCGCGCCGGCTGGTGACGCTGGGCTTCGCAGGCCGTCCATGGTGGCCACGGCCACTCGGACGGCGGC
CGCCATGGCGGATCAGGAGGAGCGAAACCTCGGGCGGCAAGCTCTAGTCGCGGCGTTGGCGGGCGGCTCCGGGCCCTGCGACGTC
CGGCCAGCGGAGATCAAGAGGGCTTTCGCTCAGCGCTTCAACATCCGTGAAGACGAGTTGCAAGTCTCCCCCCTCGCCAACAACGGC
GAGTTCCTGGTGTTCTTCCACGACCACAGATTCCGCAACGCCGCGTTGGCTCTCGCTCTGCAGCCGGGCGCCGCCGCCCCCATTGTCC
TGAGAAGCGCATCCTTCACGCTTTCTCCCTGGACAAGGTCGGCGTTCCGCAGGCCGGCCAATGCCATGGCTGCCGGGAAGAAGATGC
AGCGCAAGGCGCGGGTGTGCCTGGAGGGAGTCCCCAACCACGCGTGGAACATCGAGTCCGTCAAGGGGCTGTTCGACGGCGGGGC
GCCATGACCGTCGAAGAGCAGGACTTGAACGCCAACTCGGAAGAGGAGGAAGGGTGCATGACCGTGTGGGTTTGGGTTGACAGGG
TACTTGAACCAACAGTTGCTATTTCTCAGAGTTAAAATTGTCTGTCGAGTGATCACCGACTTATACAACCTCGTTCATTACAGTCTGAA
TCACCGATTATCATGTACTAGTACAATCAATTTGTTCAGAGCTATAGGACACTAATCTCTACTCTGCAGCTCTGCACCATAGAGTTGTG
CCTGACGCTAGTGCAAACAGCCTCTCGGCCATGACTTCAGAAGCCACACGTCCACGAAAAGACACCACCTGAAGCAGCATTCACTTCT
GTAAAACATGGCTGCTGAAAAATAAATAATATAGTAAAAGCTACCTTATTGTACACTATCTGACTATCTACACAGATGATTGCAACAG
AAAAAAAATGAAGCTGGTGGTTCTTAGATCCCTATATTTTTTATATGGCATTGCTATTTAGACAGCATTATTTTCTGCAACATCATCTAC
TATCTAGTACATTGTAAAATTAATAAGTATTATTAGCAAAACAACATCGACGATCTACTACGGAACAATGATAAGAATTCAAGAGAAT
CAGCTATGTAGATCCATATATATTCAAGAGGATCAGCAATTTAGCACCGCCACAGCCACAGGTATGGGGAAATGGACAATGGTTTAC
AGTACCTTCTCCAGAATCATGGAAAAAGATGCCAGGTCCTAACTCCCAAATATGGCTATGGAATAAGACTAATGCTAGTTCGACTCGC
TGAATCTCTCAAACACCAACGTGCATAGTCGTGCTTGTCGCGGAACACAACACTCAGTAACCACCTGGCTCCAGAAGTCATCTACCAA
ACTACAAACCTAAGATCTTTTTTCCTGCATTCCATCAGTAAGCACGTCACACGGTAGGAGAGCCATTCGGGACAAGAAGTCCTAACCT
GGTCTCAAGGTCGATGGGGTGGCTGATGTCAACGTTGACAAAGTCCCCTTCCTCGGAAGTATGGAACAACTGCTGCACATGCTCTCC
TACTTTCTGCTCCTCCCTGCCCTGTCCTCACCAAGGTTCGATGACGGCGGGCCA

>BdiBd21-3.1G0043300

ATGTCAAGGACCTTCTGGATATGCTGCTTAAACGGCGACAGAACTCTGATGAGCCTTCGTTAACACTAGGACAAGAAAGGAAACATA
ACTGCATAAGAATTCTGAGTACAATTTGTATGGTTTGGTTTCCCCTGAACATCGTTTTCGTCGAGAATGTATTGCACAGCTGTGAGATT
AGAAGGCAAACAAATTAAGAACTTCAGAAGTGTTTCCCTCTGACAATGTGTGTGTTGCTTGTGATTCACAGCCTTGTGCATGCTCTCTT
GCTGCTCCAGTTTTCTTGAGCTGGGTGTTGAATGAATATCACTCAGGATAGTTGCATTTGGTGTGTGAAGAGTTCAGTGTCCGCTCAA
GGCCAGTTGCCTGATGCGATCGTTTTAATAATAGTACATCTAAATGTCGCCTGTGAGGCCGTGACTTCAGTTTCTGTAATACCTCCGCG
TCTCAGGTCCGCCATCACTGCTCCTGCGCCAGGGTGACAGCTCGCCTCAGATCATTGATGACGGTGGTTGGCAGTTGGCTCCGGGAT
CTGCTACTTAAGCAGCAGGCCTGGGGGAAGCTGAGCTGAGGCAACCCGTCAAGAGCTCCCTTCTCCGCCGCCGCGTCTCGCTCCGGC
TGCTCCTCCTCAGGCTCCTGCGCCCTGTTTCTTCTTCGGTTATCTCCCCCGCTCATGGCCGCGCCGGCTGGTGACGCTGGGCTTCGCAG
GCCGTCCATGGTGGCCACGGCCACTCGGACGGCGGCGGCCATGGCGGATCAGGAGGAGCGAAACCTCGGGCGGCAAGCTCTAGTC
GCGGCGTTTGGCGGGCGGCTCCGGGCCCTGCGACGTCCGGCCAGCGGAGATCAAGAGGGCTTTCGCTCAGCGCTTCAACATCCGTG

AAGACGAGCTGCAAGTCTCCCCCCTCGCCAACAACGGCGAGTTCCTGGTGTTCTTCCACGACCACAGATTCCGCAACGCCGCGTTGGC
TCTCGCTCTGCAGCCGGGCGCCGCCGCCCCCATTGTCCTGAGAAGCGCATCCTTCACGCTTTCTCCCTGGACAAGGTCGGCGTTCCGC
AGGCCGGCCAATGCCATGGCTGCCGGGAAGAAGATGCAGCGCAAGGCGCGGGTGTACCTGGAGGGGGTCCCCAACCACGCGTGGA
ACATCGAGTCCGTCAAGGGGCTGTTCGACGGCGGCGGCGCCATGACCGTCGAAGATCAGGACTTGAACGCCAACTCGGAAGAGGA
GGAAGGGTGCATGACCGTGTGGGTTTGGGTTGACAGGGTCGAAGATTTGCCGAAGCGGGGGACTCTGCGGCTCCAGCGTCCTTCTT
CTTCTTCTTCTACGGCTACGGCTACGGCGCGGCAGATGCATTGTCTTCTTCCAGAGCTTGGCTTCTTTGAGGAGCATGACGTGTTGTTG
CACTTGGACAGCGTGATTGATTTCACTCCGCGTCCTGTCTAGATCTCGAGTTCGGGCAACTGGCCATACTGTTAACTCCCCTGTTAAGT
GTTAACTTGAGCACATGCGAGTAGTCTATCTCTTCGCTTATCCTTTAATTCATGAAGCTGTAATCGAATCTGTATAGGTTTCGTTTAACC
CATCATCGTAGATAAGGTGATGGAGTTGTTTGATCCGGTGTGTCTGCGTCGTGTTAGACGAGGTTTTCCCCTTCTCGAGTTGGGCGCG
GAGTCGTCTTTGTACTAGGTGCAACAATCGCAAATGTTGTTTCGAAAACATATACGGAGTATGATATACTTATCCCAACATATTTTGTA
CCGCATAGCCTGTATCTCAGTAGAAACTAGTTACAATTCATGCTATTTGGTCACTACTATGAAATGAAATCATTTCAAACATTTTTCAG
GCAGAGAGCCGGCAATACTAGAAACCCTGCTCCCTTCTCACAGAGCAACAGAGAAAAACAGAGTACTTGAACCAACAGTTGCTATTT
CTCAGAGTTAAAATTGTCTGTCGAGTGATCACCGACTTATACAACCTCGTTCATTACAGTCTGAATCACCGATTATCATGTACTAGTAC
AATCAATTTGTTCAGAGCTATAGGACACTAATCTCTACTCTGCAGCTCTGCACCATAGAGTTGTGCCTGACGCTAGTGCAAACAGCCT
CTCGGCCATGACTTCAGAAGCCACACGTCCACGAAAAGACACCACCTGAAGCAGCATTCACTTCTGTAAAACATGGCTGCTGAAAAA
TAAATAATGTAGTAAAAGCTACCTTATTGTACACTATCTGACTATCTACACAGATGATTGCAACAGAAAAAAAATGAAGCTGGTGTTT
CTTAGATCCCTGTATTTTTTTATATGGCATTGCTATTTAGACAGCATTATTTTCTGCAACATCATCTACTATCTAGTATATTGTAAAATTA
ATAAGTATTATTAGCAAAACAACATCGACGATCTACTACGGAACAAT

```
Alignment of Sequence_1:  [Untitled Sequence #1] with  Sequence_2: [Sequence Window #2]

Similarity : 1752/2421 (72.37 %)

Seq_1  1      ATGTCGAGGAGCTTCTGGATATGCTGCTTAAACGGCGACAGAACTCTGATGAGCCTTCGT  60
              ||||| |||| |||||||||||||||||||||||||||||||||||||||||||||||||
Seq_2  1      ATGTCAAGGACCTTCTGGATATGCTGCTTAAACGGCGACAGAACTCTGATGAGCCTTCGT  60


Seq_1  61     TAACACTAGGACAAGAAAGGAAACATAATTGCATAAGAATTCTGAGTACAATTTGTATGG  120
              |||||||||||||||||||||||||||| |||||||||||||||||||||||||||||||
Seq_2  61     TAACACTAGGACAAGAAAGGAAACATAACTGCATAAGAATTCTGAGTACAATTTGTATGG  120


Seq_1  121    TTTGGTTTCCCCTAAACATCGTTTTCGTCGAGAATGTATTGCACAGCTGTGAGATTAGAA  180
              ||||||||||||| ||||||||||||||||||||||||||||||||||||||||||||||
Seq_2  121    TTTGGTTTCCCCTGAACATCGTTTTCGTCGAGAATGTATTGCACAGCTGTGAGATTAGAA  180


Seq_1  181    GGCAAACAAATTAAGAACTTCAGAAGTGTTTCCCTCTGACAATGTGTGTGTTGCTTGTGA  240
              ||||||||||||||||||||||||||||||||||||||||||||||||||||||||||||
Seq_2  181    GGCAAACAAATTAAGAACTTCAGAAGTGTTTCCCTCTGACAATGTGTGTGTTGCTTGTGA  240


Seq_1  241    TTCACAGCCTTGTGCATGCTCTCTTGCTGCTCCAGTTTTCTTGAGCTGGGTGTTGAACGA  300
              ||||||||||||||||||||||||||||||||||||||||||||||||||||||||| ||
Seq_2  241    TTCACAGCCTTGTGCATGCTCTCTTGCTGCTCCAGTTTTCTTGAGCTGGGTGTTGAATGA  300


Seq_1  301    AGATCACTCAGGATAGTTGCATTTGGTGTGTGAAGAGTTCAGTGTCCGCTCAAGGCCAGT  360
              | ||||||||||||||||||||||||||||||||||||||||||||||||||||||||||
Seq_2  301    ATATCACTCAGGATAGTTGCATTTGGTGTGTGAAGAGTTCAGTGTCCGCTCAAGGCCAGT  360


Seq_1  361    TGCCTGATGCGATCGTTTTAATAATAGTACATCTAAATGTCGCCTGTGAGGCAGTGACTT  420
              |||||||||||||||||||||||||||||||||||||||||||||||||||| |||||||
Seq_2  361    TGCCTGATGCGATCGTTTTAATAATAGTACATCTAAATGTCGCCTGTGAGGCCGTGACTT  420


Seq_1  421    CAGTTTCTGTAAGCTGAGCGCTTATTAAAAGGGCCTTCTGTGAACGGACTAGCCTTTGCT  480
              ||||||||||||
Seq_2  421    CAGTTTCTGTAA------------------------------------------------  432
```

```
Seq_1  481   TCTTTCCGGGACGCTTTGACGGCGCCTACCTCTGCCGTTGCTGCTGGGGCATTGATGCCG  540
                                       ||||
Seq_2  433   --------------------------TACC------------------------------  436

Seq_1  541   GCTTTGGGGGAGCCTGCCTCCTCCGGGTTTTCGCGTCCGCGTCTCAGGTCCGCCATCACT  600
                                                ||||||||||||||||||||||||| 
Seq_2  437   -----------------------------------TCCGCGTCTCAGGTCCGCCATCACT  461

Seq_1  601   GCTGCTGCGCCAGGGTGACAGCTCGCCTCAGATCATTGATGACGATTGTTGGCAGTTGGC  660
             ||| |||||||||||||||||||||||||||||||||||||||| | |||||||||||||
Seq_2  462   GCTCCTGCGCCAGGGTGACAGCTCGCCTCAGATCATTGATGACGGTGGTTGGCAGTTGGC  521

Seq_1  661   TCCGGGATCTGCTACTTAAGCAGCAGGCCTGGGGGAAGCTGAGCTGAGGCAACCCGTCAA  720
             ||||||||||||||||||||||||||||||||||||||||||||||||||||||||||||
Seq_2  522   TCCGGGATCTGCTACTTAAGCAGCAGGCCTGGGGGAAGCTGAGCTGAGGCAACCCGTCAA  581

Seq_1  721   GAGCTCCCTTCTCCGCCGCCGCGTCTCGCTCCGGCTGCTCCTCCTCAGGCTCCTGCGCCC  780
             ||||||||||||||||||||||||||||||||||||||||||||||||||||||||||||
Seq_2  582   GAGCTCCCTTCTCCGCCGCCGCGTCTCGCTCCGGCTGCTCCTCCTCAGGCTCCTGCGCCC  641

Seq_1  781   TGTTTCTTCTTCGGTTATCTCCCCCGCTCATGGCCGCGCCGGCTGGTGACGCTGGGCTTC  840
             ||||||||||||||||||||||||||||||||||||||||||||||||||||||||||||
Seq_2  642   TGTTTCTTCTTCGGTTATCTCCCCCGCTCATGGCCGCGCCGGCTGGTGACGCTGGGCTTC  701

Seq_1  841   GCAGGCCGTCCATGGTGGCCACGGCCACTCGGACGGCGGCCGCCATGGCGGATCAGGAGG  900
             |||||||||||||||||||||||||||||||||||||||| |||||||||||||||||||
Seq_2  702   GCAGGCCGTCCATGGTGGCCACGGCCACTCGGACGGCGGCGGCCATGGCGGATCAGGAGG  761

Seq_1  901   AGCGAAACCTCGGGCGGCAAGCTCTAGTCGCGGCGTT-GGCGGGCGGCTCCGGGCCCTGC  959
             ||||||||||||||||||||||||||||||||||||| ||||||||||||||||||||||
Seq_2  762   AGCGAAACCTCGGGCGGCAAGCTCTAGTCGCGGCGTTTGGCGGGCGGCTCCGGGCCCTGC  821

Seq_1  960   GACGTCCGGCCAGCGGAGATCAAGAGGGCTTTCGCTCAGCGCTTCAACATCCGTGAAGAC  1019
             ||||||||||||||||||||||||||||||||||||||||||||||||||||||||||||
Seq_2  822   GACGTCCGGCCAGCGGAGATCAAGAGGGCTTTCGCTCAGCGCTTCAACATCCGTGAAGAC  881

Seq_1  1020  GAGTTGCAAGTCTCCCCCCTCGCCAACAACGGCGAGTTCCTGGTGTTCTTCCACGACCAC  1079
             ||| ||||||||||||||||||||||||||||||||||||||||||||||||||||||||
Seq_2  882   GAGCTGCAAGTCTCCCCCCTCGCCAACAACGGCGAGTTCCTGGTGTTCTTCCACGACCAC  941

Seq_1  1080  AGATTCCGCAACGCCGCGTTGGCTCTCGCTCTGCAGCCGGGCGCCGCCGCCCCCATTGTC  1139
             ||||||||||||||||||||||||||||||||||||||||||||||||||||||||||||
Seq_2  942   AGATTCCGCAACGCCGCGTTGGCTCTCGCTCTGCAGCCGGGCGCCGCCGCCCCCATTGTC  1001

Seq_1  1140  CTGAGAAGCGCATCCTTCACGCTTTCTCCCTGGACAAGGTCGGCGTTCCGCAGGCCGGCC  1199
             ||||||||||||||||||||||||||||||||||||||||||||||||||||||||||||
Seq_2  1002  CTGAGAAGCGCATCCTTCACGCTTTCTCCCTGGACAAGGTCGGCGTTCCGCAGGCCGGCC  1061

Seq_1  1200  AATGCCATGGCTGCCGGGAAGAAGATGCAGCGCAAGGCGCGGGTGTGCCTGGAGGGAGTC  1259
             |||||||||||||||||||||||||||||||||||||||||||||| ||||||||| |||
Seq_2  1062  AATGCCATGGCTGCCGGGAAGAAGATGCAGCGCAAGGCGCGGGTGTACCTGGAGGGGGTC  1121

Seq_1  1260  CCCAACCACGCGTGGAACATCGAGTCCGTCAAGGGGCTGTTCGACGGCGG-GGCGCCATG  1318
             |||||||||||||||||||||||||||||||||||||||||||||||||| |||||||||
Seq_2  1122  CCCAACCACGCGTGGAACATCGAGTCCGTCAAGGGGCTGTTCGACGGCGGCGGCGCCATG  1181
```

```
Seq_1  1319  ACCGTCGAAGAGCAGGACTTGAACGCCAACTCGGAAGAGGAGGAAGGGTGCATGACCGTG  1378
             ||||||||||| ||||||||||||||||||||||||||||||||||||||||||||||||
Seq_2  1182  ACCGTCGAAGATCAGGACTTGAACGCCAACTCGGAAGAGGAGGAAGGGTGCATGACCGTG  1241

Seq_1  1379  TGGGTTTGGGTTGACAGGGT----------------------------------------  1398
             ||||||||||||||||||||
Seq_2  1242  TGGGTTTGGGTTGACAGGGTCGAAGATTTGCCGAAGCGGGGGACTCTGCGGCTCCAGCGT  1301

Seq_1  1399  ------------------------------------------------------------  1398

Seq_2  1302  CCTTCTTCTTCTTCTTCTACGGCTACGGCTACGGCGCGGCAGATGCATTGTCTTCTTCCA  1361

Seq_1  1399  ------------------------------------------------------------  1398

Seq_2  1362  GAGCTTGGCTTCTTTGAGGAGCATGACGTGTTGTTGCACTTGGACAGCGTGATTGATTTC  1421

Seq_1  1399  ------------------------------------------------------------  1398

Seq_2  1422  ACTCCGCGTCCTGTCTAGATCTCGAGTTCGGGCAACTGGCCATACTGTTAACTCCCCTGT  1481

Seq_1  1399  ------------------------------------------------------------  1398

Seq_2  1482  TAAGTGTTAACTTGAGCACATGCGAGTAGTCTATCTCTTCGCTTATCCTTTAATTCATGA  1541

Seq_1  1399  ------------------------------------------------------------  1398

Seq_2  1542  AGCTGTAATCGAATCTGTATAGGTTTCGTTTAACCCATCATCGTAGATAAGGTGATGGAG  1601

Seq_1  1399  ------------------------------------------------------------  1398

Seq_2  1602  TTGTTTGATCCGGTGTGTCTGCGTCGTGTTAGACGAGGTTTTCCCCTTCTCGAGTTGGGC  1661

Seq_1  1399  ------------------------------------------------------------  1398

Seq_2  1662  GCGGAGTCGTCTTTGTACTAGGTGCAACAATCGCAAATGTTGTTTCGAAAACATATACGG  1721

Seq_1  1399  ------------------------------------------------------------  1398

Seq_2  1722  AGTATGATATACTTATCCCAACATATTTTGTACCGCATAGCCTGTATCTCAGTAGAAACT  1781

Seq_1  1399  ------------------------------------------------------------  1398

Seq_2  1782  AGTTACAATTCATGCTATTTGGTCACTACTATGAAATGAAATCATTTCAAACATTTTTCA  1841

Seq_1  1399  ------------------------------------------------------------  1398

Seq_2  1842  GGCAGAGAGCCGGCAATACTAGAAACCCTGCTCCCTTCTCACAGAGCAACAGAGAAAAAC  1901

Seq_1  1399  -----ACTTGAACCAACAGTTGCTATTTCTCAGAGTTAAAATTGTCTGTCGAGTGATCAC  1453
                  |||||||||||||||||||||||||||||||||||||||||||||||||||||||
Seq_2  1902  AGAGTACTTGAACCAACAGTTGCTATTTCTCAGAGTTAAAATTGTCTGTCGAGTGATCAC  1961

Seq_1  1454  CGACTTATACAACCTCGTTCATTACAGTCTGAATCACCGATTATCATGTACTAGTACAAT  1513
             ||||||||||||||||||||||||||||||||||||||||||||||||||||||||||||
Seq_2  1962  CGACTTATACAACCTCGTTCATTACAGTCTGAATCACCGATTATCATGTACTAGTACAAT  2021
```

```
Seq_1  1514  CAATTTGTTCAGAGCTATAGGACACTAATCTCTACTCTGCAGCTCTGCACCATAGAGTTG  1573
             ||||||||||||||||||||||||||||||||||||||||||||||||||||||||||||
Seq_2  2022  CAATTTGTTCAGAGCTATAGGACACTAATCTCTACTCTGCAGCTCTGCACCATAGAGTTG  2081

Seq_1  1574  TGCCTGACGCTAGTGCAAACAGCCTCTCGGCCATGACTTCAGAAGCCACACGTCCACGAA  1633
             ||||||||||||||||||||||||||||||||||||||||||||||||||||||||||||
Seq_2  2082  TGCCTGACGCTAGTGCAAACAGCCTCTCGGCCATGACTTCAGAAGCCACACGTCCACGAA  2141

Seq_1  1634  AAGACACCACCTGAAGCAGCATTCACTTCTGTAAAACATGGCTGCTGAAAAATAAATAAT  1693
             ||||||||||||||||||||||||||||||||||||||||||||||||||||||||||||
Seq_2  2142  AAGACACCACCTGAAGCAGCATTCACTTCTGTAAAACATGGCTGCTGAAAAATAAATAAT  2201

Seq_1  1694  ATAGTAAAAGCTACCTTATTGTACACTATCTGACTATCTACACAGATGATTGCAACAGAA  1753
              |||||||||||||||||||||||||||||||||||||||||||||||||||||||||||
Seq_2  2202  GTAGTAAAAGCTACCTTATTGTACACTATCTGACTATCTACACAGATGATTGCAACAGAA  2261

Seq_1  1754  AAAAAATGAAGCTGGTGGTTCTTAGATCCCTATA-TTTTTTATATGGCATTGCTATTTAG  1812
             ||||||||||||||||| ||||||||||||| || |||||||||||||||||||||||||
Seq_2  2262  AAAAAATGAAGCTGGTGTTTCTTAGATCCCTGTATTTTTTTATATGGCATTGCTATTTAG  2321

Seq_1  1813  ACAGCATTATTTTCTGCAACATCATCTACTATCTAGTACATTGTAAAATTAATAAGTATT  1872
             |||||||||||||||||||||||||||||||||||||| |||||||||||||||||||||
Seq_2  2322  ACAGCATTATTTTCTGCAACATCATCTACTATCTAGTATATTGTAAAATTAATAAGTATT  2381

Seq_1  1873  ATTAGCAAAACAACATCGACGATCTACTACGGAACAATGATAAGAATTCAAGAGAATCAG  1932
             ||||||||||||||||||||||||||||||||||||||
Seq_2  2382  ATTAGCAAAACAACATCGACGATCTACTACGGAACAAT----------------------  2419

Seq_1  1933  CTATGTAGATCCATATATATTCAAGAGGATCAGCAATTTAGCACCGCCACAGCCACAGGT  1992

Seq_2  2420  ------------------------------------------------------------  2419

Seq_1  1993  ATGGGGAAATGGACAATGGTTTACAGTACCTTCTCCAGAATCATGGAAAAAGATGCCAGG  2052

Seq_2  2420  ------------------------------------------------------------  2419

Seq_1  2053  TCCTAACTCCCAAATATGGCTATGGAATAAGACTAATGCTAGTTCGACTCGCTGAATCTC  2112

Seq_2  2420  ------------------------------------------------------------  2419

Seq_1  2113  TCAAACACCAACGTGCATAGTCGTGCTTGTCGCGGAACACAACACTCAGTAACCACCTGG  2172

Seq_2  2420  ------------------------------------------------------------  2419

Seq_1  2173  CTCCAGAAGTCATCTACCAAACTACAAACCTAAGATCTTTTTTCCTGCATTCCATCAGTA  2232

Seq_2  2420  ------------------------------------------------------------  2419

Seq_1  2233  AGCACGTCACACGGTAGGAGAGCCATTCGGGACAAGAAGTCCTAACCTGGTCTCAAGGTC  2292

Seq_2  2420  ------------------------------------------------------------  2419

Seq_1  2293  GATGGGGTGGCTGATGTCAACGTTGACAAAGTCCCCTTCCTCGGAAGTATGGAACAACTG  2352

Seq_2  2420  ------------------------------------------------------------  2419
```

```
Seq_1  2353  CTGCACATGCTCTCCTACTTTCTGCTCCTCCCTGCCCTGTCCTCACCAAGGTTCGATGAC  2412

Seq_2  2420  ------------------------------------------------------------  2419


Seq_1  2413  GGCGGGCCA  2421

Seq_2  2420  ---------  2419
```

BdindelWSU_2, UPSTREAM

>Bradi1g42790

AAGACAACCTTTAAAACAAAATTCGGGTTATATGAGTGGTTAGTCATGCCTTTTGGGTTAACTAATGCACCTAGTACTTTCATGCGTTT
GATGAACCATGTTTTAAGGCACTTCATAGGAAATTTGTGGTTGTGTACTTTGATGATATATTAATTTACAGCCGCAATGAATCTGAACA
TTGTGATCATATTCGACAAGTTTTGCAAGTGTTGCGTCATGCTAGGCTTTATGGTAATCTTGATAAGTGCACATTTTGCAAAGATAAG
GTCATATTTCTGGGATATGTTGTTTTTAAACATGGAGTTGAGGTAGATGATTCTAAAATTGAAGCTATTAAAAATTGGCCTACTCCAAT
GAATGTTAGTCAAGTACGAAGTTTTCACGGTCTTGCTGGTTTCTATAGGCGTTTTGTGAAAGATTTCAGTACTATTGTTGCACATTTGA
ATGAGTTAACTAAAAAAGGTGTTGAGTTTGTTTGGGGCCCATCCAAAGATAATGCTTTTGATGAACTAAAACGTCGTTTGACTACTGC
ACCTTTGCTTGTACTTCCTGATTTCACTAAACAATTTGAGATTGCGTGTGATGCTAGTGGAATTGGTATTGGAGGTGTGTTGATGCAA
GAGGGTAGACCGATTGCTTATTTTTCTGAAAAATTGAATGGTGCACAATTGAACTATCCTGTTTATGATAAAGAATTGTATGCTCTTGT
TCGTGTTCTTAAGGTTTGGCAACATTATTTGTGGCCAAAGTAATTTATCATACATTCTGATCATGAGCCATTGAAATATTTGAAAGCTC
AATCCAATTTACATAAACATCATGCTAAGTGGGTTGAATTTATTGAGTCTTTCACGTACATTATTAAGCATAAGAAGGGTAAAGATAA
TGTGGTGGCTGATGCTCTATCTAGGAAAAATATGTTATTGACACTTTTGGATGTTAAAGTTCCTGGTTTAGAGACTTTGCGTGATTTAT
ATGCTGCTGATCGTGACTTTGCCGCACCATATTCTCTTTGTACAGCAGGGAAAGCATGGGAAAAATATCACATACATGATGGCCTGTT
GTTTAGAGCTAACAAACTATGTGTTCCAGAATCGTCCGTGCGTTTGCTATTGTTGCAGGAATCCCATGCGGGCGGTTTGATGGGTCAC
TTTGGACGTGAGAAGACATTGCTTATGCTCGCTGATCATTTTTATTGGCCTAAGATGAGGCGTGATGTGGACAGGTTTGTGAAACGG
TGTATCACTTGCAATAAGTCAAAGTCCAAGCTGAAGCCTCATGGTTTGTATACTCCTCTTCTTGCTCCTACTACTCCTTGGGAAGATAT
AAGCATGGATTTTGTGTTAGGATTGCCTAGGACTAGAAGATGACATGATTCAATCTTTGTGGTAGTAGATCGTTTTTCCAAGATGGCA
CATTTTATTGCTTGCCACAAGAGCGACGATGCGTCGCATATTGCTAATTTGTTTTTCAGGGACACTGTACGCTTACATGGAGTATCAAA
GACGATTGTTTCTGATCGTGATGTGAAGTTTATGAGCTATTTCTAGAAGACGCTTTGGGGCAAGCTCGGGACAAAGTTATTGTTCAGT
ACAACTTGTCATCCCCAAATAGATGGTAAAACGGAGGTGCATCGCACACTATCAATATTGCTGTGATCCATGATCAAGAAGAACCTAC
ATGAGTGGGAAGATTGTTTGCCGCATGTGGAGTTTGCATATAACAGGGCGGTACACTCAACAACGCAGCTCTGTCCCTTCGAGGTGG
TCTATGGTTTTAAACCCATTACACCCCTTAATTTGCTGCCTCTACCCATGCAAGAGCGCGCCGACATGGAGGCATCCAAGAGGCAGAT
TTTGTGAGGAAGATCCATGAGAAGACAAAAGAAGCAATTGAGAAGAAAGGCAAGTACACCGCTGACCGTGTGAACAAGAAGCGCA
AGGAGGTGTTGTTTTAGCCCGGCGACATGGTTTGGGTACACTATCGCAAGGACAGATTTCCAGAGCGTCGTAAGTCCAAGTTGCAAC
CACGTGGTGCTGGACCATATAAAGTGCTTGCCAGGATTAATAACAATGCATACAAGATTGATCTTCCAACCGATGAGTTTGGCGTTAG
TAATACATTAAATGTTGCTGATTTGACGCCGTATGCTGGAGAAGACCTTGTTGTGTCGGGGTCGACGCCTTTTCAAGGGGGGGAGGA
TGATGAGGACATCCCTAGTTCTTTATCATCAACTCCAAATGATGATGTTGCTGCACAAGACAAGCCCAATGAAGTTAGACTTGGGCCA
ATAACAAGAGCACGTGCGAAGCTACTTGAACAACAGGTGAACTCACTCTTAATTGAATCTGATGTTTTAATTGATGAGAGCTTTATAC
TACCTAAGTCTATGCATTTATGTATGATCAGATTTGAAGATGTTTCAAGCCTTGCACGTGGAGGATGAGAGTTGTAGCAAGATCCATT
CCCGTTGATTTCCAATATCAAGAAGAGCGCGAGGGAGGAGAGGGAGGCCGGCGCACCACATGAAGAGGAGGAAATCAACCAGCAA
TAGAAGTTATCGTGGCTGATGCTAGGACAAGTCCTATGACACGTCAAACATGTCTAAGGCATCAGCGCACGTCGATGAGCATGTGAC
ACACATAGGGTTGCCTACGACATGGTTTATTTGCCTAAGTGTGAGTTGGGCCTGCGTGCTCGTAGATTGGGCAACCTTGACTAGCAAT
GTGACTCAGTATGATGACTATGTTTCACAAGTTATAGGGCAAGGTGTTGCCAACTGATAGCATTGGACTCGACAATGAGATTGCCGG
GTCAGACAGACCCGCACTGAGACACAACGAGATGATTGTCATTTGTTAGTCTTAAGTATGATGTTTATGCCAGTTCTAGAACTGAGGT
CATCGCATGAGCTTGGGATGTGAATCGGCTTACTTAGGGGTTACCAAACGCTACTCCATAACTGGGTAGTTATAAAGGAAGCTTTTG
GGTTTGCTGAGAAGCATGCTGCAAGTCATGGTTGATCAAGATGGATTTGCCCCTCCCTTTGGGAGAGATATCTCTGGGCCCTCTCGAG
TGATCAGATTCGGAAAGCATGGCCATGCGACTTGGGTTAAGCATTAACCCGTTCGGGAATCTGAATCACGAGATTGAGAAGAGAGT
CGAGTTATACACAAGGATGACAAGTACTCGCCTTGAGCTCGACACACATATCGTGAGGCAGAAGGAATGTTGCATGTGGACACATTG
TATAGTTCGTCAATATACTTTGTGGTTATTCAGGAGTCAGCACGTGCTGCTCGGCGCCGCTGCTGACTATCGACTTGGGTCGGAACCT

GTGTTCGGACTCGTAGTTGTGTCCGTTTGACCACGAACCTGTAGGGTTGCACACTTAAGGGGATGGGGCCGAATTGGATCGGATCCA
ACTCGTATCCCGCTTACACTCCTGATACGGGTGCGGGTCGCCCGATCTTTCGATGAGATTGATAACTCTCGATTTGGGTAGGAGGTGA
CGTTGACGATCCGACTACGGCCTAGGAGGCAGCGTCTTAGCAATCGATACGCCAACTCCAGAAGGTTATTAATCACGCGGGGGCAC
GATCAACCTGACCACGAAGGTCTTTGTTCCTGCAAGCAATCGAAGAACAAGCAAGAACAAGATAAATAGCGGATGCAATCTCAAATT
GCGAGTATACTATATCAAACCAATGTCTCAACAAGACGATAAGTTGGAGTCTTGACGACGGTAAAACAGGTGGTCTAACCGACACAC
GCGATTACACGAAAGTAGCAAAGATGGCTAAACTTTATCTAATCAAAACCCAAGGCTCCTTAGGGGGGGGCTCATGGGGTATATAAA
GGAGGAGGGAGAGATATTTTCGTCCACCTTGAGTAAGGAGGCCGAAATCCAACTCTATCTCTAAGTTTCCTAACAAACTACAACTCTT
TAGGAAACAGAAAAACAGATCCGTCAGCTTGTTTTGTCCGTCACGGTCAGCACGAGTTGAATCTATTGATGGGACCAGATCCGTTGG
AAAGGTTTTGTAATTACCTTTCCAACAAGTACTTGTTTGCTTGATTTGGACTCCGGATACGGCCTAGGTGATTAAAACAAATTCGGGTC
TCCTGGCAGCTCGGATCCGAATCGGATTCAACTTTAATTCGTGATATCTTTCTCTAGCAAGCTCCGAATCATGTGCCGTTTGATTTGTT
GGAAAGTAGACTTGATAGGCTTCACTTTGAATCTCCCTTTGCAGGCTATCACACTTCATCTTCACTTTGGATTTGTAAAGTTCATTAAG
ATGTCTGCATAATAAGATTACACAAGATAGTAGCTCCGCCTCATTGCAAGTAACATATTGAAAACATTTTGTAATTGAATTCACCTTAT
TATAATTGTTATTAGATACACCAACAATTAGGGTTCTAATGGTCGTATCCATGGTGATCATGTCCATATCATCCTCCCTTTCTTGAAAGA
AAAGCCGTCCTCGGCGTCGTTTGTGCTGAAAAGAGACTGACAAAATAGACAAAGTGTGTGCATGTTATATGTATATGTCATCAATTTT
ACATTTTTGTATTGTTCTATAAATTTAGATCACGCACCTCATCAATTCAATCATGTTTTCTTTTTTTTAAAGTTTTTCTCCTTTTATACACA
TTTTGTCGCATGCTTCTTAGGTTTAGTTGTTTTCTAAATGTAAAATCAGAACATTTTTATATCTTTTTCCATTAATTGTAGTTAATTTAAA
ACATTTATTTATACATTGTTGGTGCATCATCTGTTTTTGACGGTTGTTGGTGCATCATCTAGTCGTTGTACAATTCTACGATCCATTTCTC
CGTTCCTGCACCTGAACGGAGCAACAATGTCTTTGCACGAATGGTTCATCGTGTGATCGGACGGTCGAGAATCGAGGGTGAAACAAC
GGAAGGACTGGAAAACAACCGCGCAATAACACCAGCCACTTCCTTGTAAAATCACCCCACAATCCGAAGCTTCTCCCCCCTTATTTCCT
TCGCGACGAAGCGGCACGCAGCTGCAACGACTTGCAAGACAGAAAACCTACCAAATCAAACCAAACCGCAACCAAATCTCCCAAATC
CAAATCCCCACCGTCGCGCCCTACCTCCGACTATATCCGCTGCTGCCCTCCTGTAGCTAGGCAGACATCTCCCGCTGCTGGGAGCGAC
ACGGAGCCGGCCAAGGGGCTACTGCTCCCCTACCTGCAATGCGACTTTGCAGGACTTCAATACCGAATTTTCGTAAGGGACCAAATG
TTTTCTGTCACGACTCAGGAGCATCTTGGTGTACATGTATGTTTGTTCGACTCAGTTCATCTTTCCTAATTGTTAACACACATATCTATTT
TTGTCCAGGATGCCAGTGCAACGTTATGCAACAAACAATATTCTACCTCCCCCTCGGCTCTTTCAGCAAAAGAAGTGCACAATAGGTA
CGTGGATGTTCTTACAAAGATGAAGAATACAACACAACAACCAATGTGGACCACTTAAAAAACGTCTCACAACTATCTCATGTCCATC
CTTTCAACAATGTCAGTTCCATCTTCAAGTACATCATGAAAGGTACATCACATGTACAATTTTTTGAGTGGAGAAGGGACTAAGACCG
ACAACAATAAACAAATCTTGCCATATATTTCCTGATTTAATTTATTAAAATCCCAGCAAAACATATAATAGTAGTCCAAATATTGCTCTT
CAATACAACATAGGTTCCCGCTGCAACGCGCGGGGTCTCGTCTAGTTTATACGTTATTGGCGGATCGTCTGTTTTTGACAGCTGTTGG
TGCATCATCTAGTTGCCGTACAATTCTAGCATCCAGTTCTCCGTTCCTGCATCCGAACGGAGCAACGGTGTCTTTGCACGAACAGTTCA
TCGTGTGATCGGACGGTCGAGACTCGAGGGTGAAACAGCGGAAGGACTGGGAAACAACCGCGCAATAACACCAGGCACTTCCTTGT
AAAATCACCCCACGATCCGAAGCTTCTTCCCCTCATCTTCCCGACGAAGCAGCACGCAGCTGCAACGACTTGCAAGACAGAAAACCTA
CCAAATCAAACCAAACCACAACCGAATCTCCCGAATCCAAACCCCCACCGTTGCGCCCTACCTCCGGCGATATCTGCCGCCGCCCTCCT
GTAGCTAGGCAGACATCTCCCGCTGCTG

>BdiBd21-3.1G0555100

TTTCGACGATGTGTTCCTGGATGAGGTACCCGCTGGTTTGCCACCCTTGCGTGGTATAGAACATCAAATCGATTTGATACCCGGTGCG
TCGCCGCCCAATCGAGCCCCCTACCGCACCAACCCTGAGGAAACGAAAGAAATTCAGAAACAAGTACAAGGGCTTCTCGACAAAGGT
TATATTTGCGTAAGTTTAAGTCCATGTGATGTTCCTGTTATTTTAGTTCCTAAGAAAGATGGTACATGGCGCATGTGCGTAGATTGTAG
AGCTATAAATAACATCACTATTCGATATCGTCATCCCATTCCACGTTTGGAAGATATGCTAGATGAATTGAGTGGTGCTGCTGTTTTCT
CTAAAATTGATTTGCGTAGTGGTTATCATCAAATTAGAATGAAAGAAGGGGATGAATGGAAGACAAACTTTAAAACAAAATTCGGGT
TATATGAGTGGTTAGTCATGCCTTTTGGGTTAACTAATGCACCTAGTACTTTCATGCGTTTGATGAACCATGTTTTAAGGCACTTCATA
GGAAAATTTGTGGTTGTGTACTTTGATGATATATTAATTTACAGCCGCAATGAATCTGAACATTGTGATCATATTCGACAAGTTTTGCA
AGTGTTGCGTCATGCTAGGCTTTATGGTAATCTTGATAAGTGCACATTTTGCAAAGATAAGGTCATATTTCTGGGATATGTTGTTTCTA
AACATGGAGTTGAGGTAGATGATTCTAAAATTGAAGCTATTAAAAATTGGCCTACTCCAATGAATGTTAGTCAAGTACGAAGTTTTCA
CGGTCTTGCTGGTTTCTATAGGCGTTTTGTGAAAGATTTCAGTACTATTGCTGCACATTTGAATGAGTTAACTAAAAAAGGTGTTGAG
TTTGTTTGGGGCCCAGCCCAAAATAATGCTTTTGATGAACTAAAACGTCGTTTGACTACTGCACCTTTGCTTGTACTTCCTGATTTCACT
AAACAATTTGAGATTGCGTGTGATGCTAGTGGAATTGTTATTGGAGGTGTGTTGATGCAAGAGGGTAGACCGATTGCTTATTTTTTTG
AAAAATTGAATGGTGCACAATTGAACTATCATGATTATGATAAAGAATTGTATGCTCTTGTTCGTGTTCTTGAGGTTTGGCAACATTAT
TCGTGGCCAAAGGAATTTATCATACATTCTGATCATGAGGCATTGAAATATTTGAAAGCTCAATCCAATTTACATAGACGTCATGCTAA

GTGGGTTGAATTTATTGAGTCTTTCACGTACATTATTAAGCATAAGAAGGGTAAAGATAATGTGGTGGCTGATGCTCTATCTAGGAAA
AATATGTTATTGACACTTTTGGATGTTAAAGTTCCTGGTTTAGAGAGTTTGCGTGATTTATATGCTGCTGATCGTGACTTTGCGCACCA
TATTCTCTTTGTACAGCAGGGAAAGCATGGGAAAAATATCACATACATGATGGCCTGTTGTTTAGAGCTAACAAACTATGTGTTCCAG
AATCGTCCGTGCGTTTGCTATTGTTGCAGGAATCCCATGCGGGCGGTTTGATGGGTCACTTTGGACGTGAGAAGATGTTGCTTATGCT
CGCTGATCATTTTTATTGGTCTAAGATGAGGCGTGATGTGGACAGGTTTGTGAAACGGTGTATCACTTGCAATAAGTCGAAGTCCAA
GCTGAAACCTCATGGTTTGTATACTCCTCTTCTTGCTCCTACTACTCCTTGGGAAGATATAAGCATGGATTTTGTGTTAGGATTGCCTA
GGACTAGAAGATGACATGATTCAATATTTGTGGTAGTAGATTGTTTTTCCAAGATGGCACATTTTATTGCTTGCCACAAGAGCGACGA
TGCGTCGCATATTGCTAATTTGTTTTTCAGGGACACTGTACGCTTACATGGAGTATCAAAGACGATTGTTTCTGATCGTGATGTGAAGT
TTATGAGCTATTTCTAGAAGACACTTTGGGGCAAGCTCGGGACAAAGTTATTGTTCAGTACAACTTGTCATCCCCAAACAGATGGTCA
AACGGAGGTGGTGAATCGCACACTATCAATGTTGCTGTGATCCATGATCAAGAAGAACCTACGTGAGTGGGAAGATTGTTTGCCGCA
TGTGGAGTTTGCATATAACAGGGCGGTACACTCAACAACGCAGCTCTGTCCCTTTGAGGTGGTCTATGGTTTTAAACCCATTACACCC
CTTGATTTGCTGCCTCTACCCATGTAAGAGCGCGCCGACATGGAGGCATCCAAGAGGCAGATTTTGTGAGGAAGATCCATGAGAAGA
CAAAAGAAGCAATTGAGAAGAAAGGCAAGTACACCGCTGACCGTGTGAACAAGAAGCGCAAGGAGGTGTTGTTTTAGCCCGGCGA
CATGGTTTGGGTGCACTATCGCAAGGACAGATTTCCAGAGCATCGTAAGTCCAAGTTGCAACCACGTGGTGCTGGACCATATAAAGT
GCTTGCCAAGATTAATAACAATGCATACAAGATTGATCTTCCAACCGATGAGTTTGGCGTTAGTAATACATTCAATGTTGCTGATTTGA
CGCCGTATGCTGGAGAAGACCTTGTTGTGTCGGGGTCGACGCCTTTTCAAGGCGGGGAGGATGATGAGGACATCCCTAGTTCTTTAT
CATCAACTCCAATTGATAATGTTGCTACACAAGACAAGCCCAATGAAGTTAGACTTGGGCCAATAACAAGAGCACGTGCGAAGCTAC
TTGAACAACAGGTGAACTCACTCTTAATTGAATCTGATGTTTTAATTGATGAGAGCTTTATACTACCTAAGTCTCTGCATTTATGTATG
ATCAGATTTGAAGATGTTTCAAGCCTTGCACGTGGAGGATGAGAGTTGCAGCAAGATCCATTCCCGTTGATTTCCAATATCAAGAAG
AGCGCGAGGAGAGGGAGGCCGGCGCACCACATGAAGAGGAGGAAATCAACCAGCAATAGAAGTTGTCGTGGCTGATGCTAGGAC
AAGTCCTATGACACGTCAAACATGTCTAAGGCATCAGCGCACGTCGATGAGCATGTGACACACATAGGGCTGCCTACGACATGGTTT
ATTTGCCTAAGTGTGAATTGGGTCTGCGTGCTCGTAGTTTGGGCAACCTTGACTAGCAATGTGACTCAGTATGATGACTATGTTTCAC
AAGTTATAGGGCAAGGTGTTGCCAACTGATAGCATTGGACTCGACAATGAGATTGCCGAGTCAGACAGACCCGCACTGAGACACAA
GGAGATGATTGTCATTTGTTAGTCTTAAGTACGATGTTTATGCCAGTCCTAGAACTGAGGTCATCGCATGAGCTTGGGATGTGAATCG
GCTTACTTAGAGGTTACCAAACGCTACTCCGTAACTGGGTAGTTATAAAGGTAGCTTTTGGGTTTGCTGAGAAGCATGCTGCAAGTCA
TGGTTGATCAAGATGGATTTGCCCCTCCCTTTGGGAGAGATATCTCTGGGCCCTCTCGAGTGATCAGATTCGGAAAGCATGGCCATGC
GACTTGGGTTAAGCATTAACCCGTTCGGGAATCTGAATCACGAGATCGAGAAGAGAGTCGAGTTATACACAAGGATGACAAGTACTC
GCCTTGAGCTCGACACACATATCGTGAGGCAGAAGGAATGTTGCATGTGGACACATTGTATGGTTCGTCAATATACTTTGTGGTTATT
CGGGAGTCAGCACGTGCTGCTCGGCGCCGCTGCTGACTATCGACTTGGGTCGGAACCTGTGTTCGGACTCGTAGTTGTGTCTGTTTG
ACCGCGAACCTGTAGGGTCGCACACTTAAGGGGATGGGGCCGAATTGGATCGGATCCAACTCGTATCCCGCTTAGACTCCTAACGGG
CCTCAAGTGTTGAAACCCACCAGGGAGGTCTATATAAGTGGAGGAGGCATACCTGGTTAGGGTTACCCCAATTCGTGGCACGAGTCA
GACTCGGCCGTCACATCTCCACGCCCAAACCTTGCGATCGGATCTAGCAGTCCGCCGCACGGAGTTCCTCTCTATATGTGTGGATACC
TCAGAGGCGCTGCATCTGCGGCGCTTGGATGAACTGTTCATGGGATTGGCGAGGACGAACAGGCCGATCGACTACTCGGCATTGAC
ACGCATCACTGACTCTACTTCTGTTATGGGTCTGCGCGTCTAGTGGTAATCTCGTGATCCATTATCTACAGCATTGATCCGGGTGGAAT
CAGGTAAAAAAATTTGTTTTGTGCTCGCGTAGCCTATCGTGTTCCCCAACAATAACCCCTAAAGTATATTGTGAGCACCATGATTTGAA
TCTTGGTGGGTGGAGTCATGCACCCACAACTCCACCATCACACCAAGAGGTATTATTCTATCAAATCAAACAATGCCATCTCATTAATT
CAGTCATGTTTTCATTTTTTGAGACCCTCGGTACATGATTTTTTCTCAACATGCAAGTATGTCACCTTATTAGGCCCACCACATGCAAAC
TATGAATGCGAGTATGCCACCTCATTAGGTTATGTATGTATTTTTTTTGAAGCCATGCATGTATTCACTTACATTTTTGTATTGTTCTAT
AAATTTAGATCATGCACCTCATCAATTCAATCATGTTTTCTTTTTTTTTTAAGTTTTTCTCTTTTTATACACATTTTGTCGCATGCTTCTTA
GGTTTAGTTGTTTTCTAAATGTAAAATCAGAACATTTTTATATCTTTTTCCATTAATTGTAGTTAATTTAAAACATTTATTTATACATTGT
TGGTGCATCATCTGTTTTTGACGATTGTTGGTGCATCATCTAGTCGTTGTACAATTCTACGATCCATTTCTCCGTTCCTGCACCTGAACG
GAGCAACAGTGTCTTTGCACGAATGGTTCATCGTGTGATCGGACGGTCGAGAATCGAGGGTGAAACAACAGAAGGACCGGAAAACA
ACCGCGCAATAACACCAGCCACTTCCTTGTAAAATCACCCCACAATCTGAAGCTTCTCCCCCCTTATTTCCTTCGCGACGAAGCGGCAC
GCAGCTGCAACGACTTGCAAGACAGAAAACCTACCAAATCAAACCAAACCGCAACCAAATCTCCCAAATCCAAATCCCCACCGTCGCG
CCCTACCTCCGACTATATCCGCTGCCGCCCTCCTATAGCTAGGCAGACATCTCCCGCTGCTGGGAGCGACACGGAGCCGGCCAAGGG
GCTACTGCTCCCCTACCTGCAATGCGACTTTGCAGGACTTCAATACCGAATTTTCGTAAGGGACCAATGTTTTCTGTCATGACTCAGGA
GCATCTTGGTGTACATGTATGTTTGTTCGACTCAGTTCATCTTTCCTAATTGTTAACACACATATCTATTTTTGTCCAGGATGCCAGTGC
AACGTTATGCAACAAACAATATTCTACCTCCCCTCGGCTCTTTCAGCAAAAGAAGTGCACAATAGGTACGTGGATGTTCTTACAAAGA
TGAAGAATACAACACAACAACCAATGTGGACCACTTAAAAAACGTCTCACATTTGAACTATCTCATGTCCATCCTCTCAACAATATCAG

TTCCATCTTCAAGTACATCATGAAAGGTACATCACATGTACAATTTTTTGAGTGGAGAAGGGACTAAGACCGACAACAATAAACAAAT
CTTGCCATATATTTCCTGATTTAATTTATTAAAATCCCAGCAAAACATATAATAGTAGTCCAAATATTGCTCTTCAATACAACATAGGTT
CCCGCTGCAACGCGCGGAGTCTCGTTAGTTTATACGTTATTGGCGGATCGTCTGTTTTTGACAGCTGTTGGTGCATCATCTAGTTGCC
GTACAATTCTAGCATCCAGTTCTCCGTTCCTGCATCCGAACGGAGTAACGGTGTCTTTGCACGAACAGTTCATCGTGTGATCGGACGG
TCGAGACTCGAGGGTGAAACAGCGGAAGGACTGGGAAACAACCGCGCAATAACACCAGGCACTTCCTTGTAAAATCACCCCACGAT
CCGAAGCTTCTTCCCCTCATATTCCCGACGAAGCAGCACGCAGCTGCAACGACTTGCAAGACAGAAAACCTACCAAATCAAACCAAAC
CACAACCGAATCTCCCGAATCCAAACCCCCACCGTTGCGCCCTACCTCCGGCGATATCTGCCGCCGCCCTCCTGTAGCTAGGCAGACA
TCTCCCGCTGCTG

```
Alignment of Sequence_1:  [Untitled Sequence #1] with  Sequence_2: [Sequence Window #2]

Similarity : 4180/6193 (67.50 %)

Seq_1  1     ------------------------------------------------------------  0

Seq_2  1     TTTCGACGATGTGTTCCTGGATGAGGTACCCGCTGGTTTGCCACCCTTGCGTGGTATAGA  60

Seq_1  1     ------------------------------------------------------------  0

Seq_2  61    ACATCAAATCGATTTGATACCCGGTGCGTCGCCGCCCAATCGAGCCCCCTACCGCACCAA  120

Seq_1  1     ------------------------------------------------------------  0

Seq_2  121   CCCTGAGGAAACGAAAGAAATTCAGAAACAAGTACAAGGGCTTCTCGACAAAGGTTATAT  180

Seq_1  1     ------------------------------------------------------------  0

Seq_2  181   TTGCGTAAGTTTAAGTCCATGTGATGTTCCTGTTATTTTAGTTCCTAAGAAAGATGGTAC  240

Seq_1  1     ------------------------------------------------------------  0

Seq_2  241   ATGGCGCATGTGCGTAGATTGTAGAGCTATAAATAACATCACTATTCGATATCGTCATCC  300

Seq_1  1     ------------------------------------------------------------  0

Seq_2  301   CATTCCACGTTTGGAAGATATGCTAGATGAATTGAGTGGTGCTGCTGTTTTCTCTAAAAT  360

Seq_1  1     ----------------------------------------------------AAGACAAC  8
                                                                 |||||||
Seq_2  361   TGATTTGCGTAGTGGTTATCATCAAATTAGAATGAAAGAAGGGGATGAATGGAAGACAAA  420

Seq_1  9     CTTTAAAACAAAATTCGGGTTATATGAGTGGTTAGTCATGCCTTTTGGGTTAACTAATGC  68
             ||||||||||||||||||||||||||||||||||||||||||||||||||||||||||||
Seq_2  421   CTTTAAAACAAAATTCGGGTTATATGAGTGGTTAGTCATGCCTTTTGGGTTAACTAATGC  480

Seq_1  69    ACCTAGTACTTTCATGCGTTTGATGAACCATGTTTTAAGGCACTTCATAGGAAA-TTTGT  127
             |||||||||||||||||||||||||||||||||||||||||||||||||||||| |||||
Seq_2  481   ACCTAGTACTTTCATGCGTTTGATGAACCATGTTTTAAGGCACTTCATAGGAAAATTTGT  540

Seq_1  128   GGTTGTGTACTTTGATGATATATTAATTTACAGCCGCAATGAATCTGAACATTGTGATCA  187
             ||||||||||||||||||||||||||||||||||||||||||||||||||||||||||||
Seq_2  541   GGTTGTGTACTTTGATGATATATTAATTTACAGCCGCAATGAATCTGAACATTGTGATCA  600

Seq_1  188   TATTCGACAAGTTTTGCAAGTGTTGCGTCATGCTAGGCTTTATGGTAATCTTGATAAGTG  247
             ||||||||||||||||||||||||||||||||||||||||||||||||||||||||||||
Seq_2  601   TATTCGACAAGTTTTGCAAGTGTTGCGTCATGCTAGGCTTTATGGTAATCTTGATAAGTG  660
```

```
Seq_1  248   CACATTTTGCAAAGATAAGGTCATATTTCTGGGATATGTTGTTTTTAAACATGGAGTTGA  307
             |||||||||||||||||||||||||||||||||||||||||||| |||||||||||||||
Seq_2  661   CACATTTTGCAAAGATAAGGTCATATTTCTGGGATATGTTGTTTCTAAACATGGAGTTGA  720

Seq_1  308   GGTAGATGATTCTAAAATTGAAGCTATTAAAAATTGGCCTACTCCAATGAATGTTAGTCA  367
             ||||||||||||||||||||||||||||||||||||||||||||||||||||||||||||
Seq_2  721   GGTAGATGATTCTAAAATTGAAGCTATTAAAAATTGGCCTACTCCAATGAATGTTAGTCA  780

Seq_1  368   AGTACGAAGTTTTCACGGTCTTGCTGGTTTCTATAGGCGTTTTGTGAAAGATTTCAGTAC  427
             ||||||||||||||||||||||||||||||||||||||||||||||||||||||||||||
Seq_2  781   AGTACGAAGTTTTCACGGTCTTGCTGGTTTCTATAGGCGTTTTGTGAAAGATTTCAGTAC  840

Seq_1  428   TATTGTTGCACATTTGAATGAGTTAACTAAAAAAGGTGTTGAGTTTGTTTGGGGCCCAT-  486
             ||||| ||||||||||||||||||||||||||||||||||||||||||||||||||||  
Seq_2  841   TATTGCTGCACATTTGAATGAGTTAACTAAAAAAGGTGTTGAGTTTGTTTGGGGCCCAGC  900

Seq_1  487   CCAAAGATAATGCTTTTGATGAACTAAAACGTCGTTTGACTACTGCACCTTTGCTTGTAC  546
             ||||| ||||||||||||||||||||||||||||||||||||||||||||||||||||||
Seq_2  901   CCAAA-ATAATGCTTTTGATGAACTAAAACGTCGTTTGACTACTGCACCTTTGCTTGTAC  959

Seq_1  547   TTCCTGATTTCACTAAACAATTTGAGATTGCGTGTGATGCTAGTGGAATTGGTATTGGAG  606
             ||||||||||||||||||||||||||||||||||||||||||||||||||| ||||||||
Seq_2  960   TTCCTGATTTCACTAAACAATTTGAGATTGCGTGTGATGCTAGTGGAATTGTTATTGGAG  1019

Seq_1  607   GTGTGTTGATGCAAGAGGGTAGACCGATTGCTTATTTTTCTGAAAAATTGAATGGTGCAC  666
             ||||||||||||||||||||||||||||||||||||||| ||||||||||||||||||||
Seq_2  1020  GTGTGTTGATGCAAGAGGGTAGACCGATTGCTTATTTTTTTGAAAAATTGAATGGTGCAC  1079

Seq_1  667   AATTGAACTATCCTGTTTATGATAAAGAATTGTATGCTCTTGTTCGTGTTCTTAAGGTTT  726
             |||||||||||| || ||||||||||||||||||||||||||||||||||||| ||||||
Seq_2  1080  AATTGAACTATCATGATTATGATAAAGAATTGTATGCTCTTGTTCGTGTTCTTGAGGTTT  1139

Seq_1  727   GGCAACATTATTTGTGGCCAAAGTAATTTATCATACATTCTGATCATGAGCCATTGAAAT  786
             |||||||||||| |||||||||| |||||||||||||||||||||||||| |||||||||
Seq_2  1140  GGCAACATTATTCGTGGCCAAAGGAATTTATCATACATTCTGATCATGAGGCATTGAAAT  1199

Seq_1  787   ATTTGAAAGCTCAATCCAATTTACATAAACATCATGCTAAGTGGGTTGAATTTATTGAGT  846
             ||||||||||||||||||||||||||| || |||||||||||||||||||||||||||||
Seq_2  1200  ATTTGAAAGCTCAATCCAATTTACATAGACGTCATGCTAAGTGGGTTGAATTTATTGAGT  1259

Seq_1  847   CTTTCACGTACATTATTAAGCATAAGAAGGGTAAAGATAATGTGGTGGCTGATGCTCTAT  906
             ||||||||||||||||||||||||||||||||||||||||||||||||||||||||||||
Seq_2  1260  CTTTCACGTACATTATTAAGCATAAGAAGGGTAAAGATAATGTGGTGGCTGATGCTCTAT  1319

Seq_1  907   CTAGGAAAAATATGTTATTGACACTTTTGGATGTTAAAGTTCCTGGTTTAGAGACTTTGC  966
             |||||||||||||||||||||||||||||||||||||||||||||||||||||| |||||
Seq_2  1320  CTAGGAAAAATATGTTATTGACACTTTTGGATGTTAAAGTTCCTGGTTTAGAGAGTTTGC  1379

Seq_1  967   GTGATTTATATGCTGCTGATCGTGACTTTGCCGCACCATATTCTCTTTGTACAGCAGGGA  1026
             ||||||||||||||||||||||||||||||| ||||||||||||||||||||||||||||
Seq_2  1380  GTGATTTATATGCTGCTGATCGTGACTTTGC-GCACCATATTCTCTTTGTACAGCAGGGA  1438

Seq_1  1027  AAGCATGGGAAAAATATCACATACATGATGGCCTGTTGTTTAGAGCTAACAAACTATGTG  1086
             ||||||||||||||||||||||||||||||||||||||||||||||||||||||||||||
Seq_2  1439  AAGCATGGGAAAAATATCACATACATGATGGCCTGTTGTTTAGAGCTAACAAACTATGTG  1498
```

```
Seq_1  1087  TTCCAGAATCGTCCGTGCGTTTGCTATTGTTGCAGGAATCCCATGCGGGCGGTTTGATGG  1146
             ||||||||||||||||||||||||||||||||||||||||||||||||||||||||||||
Seq_2  1499  TTCCAGAATCGTCCGTGCGTTTGCTATTGTTGCAGGAATCCCATGCGGGCGGTTTGATGG  1558


Seq_1  1147  GTCACTTTGGACGTGAGAAGACATTGCTTATGCTCGCTGATCATTTTTATTGGCCTAAGA  1206
             |||||||||||||||||||||  |||||||||||||||||||||||||||||| ||||||
Seq_2  1559  GTCACTTTGGACGTGAGAAGATGTTGCTTATGCTCGCTGATCATTTTTATTGGTCTAAGA  1618


Seq_1  1207  TGAGGCGTGATGTGGACAGGTTTGTGAAACGGTGTATCACTTGCAATAAGTCAAAGTCCA  1266
             |||||||||||||||||||||||||||||||||||||||||||||||||||| |||||||
Seq_2  1619  TGAGGCGTGATGTGGACAGGTTTGTGAAACGGTGTATCACTTGCAATAAGTCGAAGTCCA  1678


Seq_1  1267  AGCTGAAGCCTCATGGTTTGTATACTCCTCTTCTTGCTCCTACTACTCCTTGGGAAGATA  1326
             ||||||| ||||||||||||||||||||||||||||||||||||||||||||||||||||
Seq_2  1679  AGCTGAAACCTCATGGTTTGTATACTCCTCTTCTTGCTCCTACTACTCCTTGGGAAGATA  1738


Seq_1  1327  TAAGCATGGATTTTGTGTTAGGATTGCCTAGGACTAGAAGATGACATGATTCAATCTTTG  1386
             ||||||||||||||||||||||||||||||||||||||||||||||||||||||| ||||
Seq_2  1739  TAAGCATGGATTTTGTGTTAGGATTGCCTAGGACTAGAAGATGACATGATTCAATATTTG  1798


Seq_1  1387  TGGTAGTAGATCGTTTTTCCAAGATGGCACATTTTATTGCTTGCCACAAGAGCGACGATG  1446
             ||||||||||| ||||||||||||||||||||||||||||||||||||||||||||||||
Seq_2  1799  TGGTAGTAGATTGTTTTTCCAAGATGGCACATTTTATTGCTTGCCACAAGAGCGACGATG  1858


Seq_1  1447  CGTCGCATATTGCTAATTTGTTTTTCAGGGACACTGTACGCTTACATGGAGTATCAAAGA  1506
             ||||||||||||||||||||||||||||||||||||||||||||||||||||||||||||
Seq_2  1859  CGTCGCATATTGCTAATTTGTTTTTCAGGGACACTGTACGCTTACATGGAGTATCAAAGA  1918


Seq_1  1507  CGATTGTTTCTGATCGTGATGTGAAGTTTATGAGCTATTTCTAGAAGACGCTTTGGGGCA  1566
             ||||||||||||||||||||||||||||||||||||||||||||||||| ||||||||||
Seq_2  1919  CGATTGTTTCTGATCGTGATGTGAAGTTTATGAGCTATTTCTAGAAGACACTTTGGGGCA  1978


Seq_1  1567  AGCTCGGGACAAAGTTATTGTTCAGTACAACTTGTCATCCCCAAATAGATGGTAAAACGG  1626
             ||||||||||||||||||||||||||||||||||||||||||||| ||||||| ||||||
Seq_2  1979  AGCTCGGGACAAAGTTATTGTTCAGTACAACTTGTCATCCCCAAACAGATGGTCAAACGG  2038


Seq_1  1627  AGGTGC---ATCGCACACTATCAATATTGCTGTGATCCATGATCAAGAAGAACCTACATG  1683
             |||||    |||||||||||||||| ||||||||||||||||||||||||||||||| ||
Seq_2  2039  AGGTGGTGAATCGCACACTATCAATGTTGCTGTGATCCATGATCAAGAAGAACCTACGTG  2098


Seq_1  1684  AGTGGGAAGATTGTTTGCCGCATGTGGAGTTTGCATATAACAGGGCGGTACACTCAACAA  1743
             ||||||||||||||||||||||||||||||||||||||||||||||||||||||||||||
Seq_2  2099  AGTGGGAAGATTGTTTGCCGCATGTGGAGTTTGCATATAACAGGGCGGTACACTCAACAA  2158


Seq_1  1744  CGCAGCTCTGTCCCTTCGAGGTGGTCTATGGTTTTAAACCCATTACACCCCTTAATTTGC  1803
             |||||||||||||||| |||||||||||||||||||||||||||||||||||| ||||||
Seq_2  2159  CGCAGCTCTGTCCCTTTGAGGTGGTCTATGGTTTTAAACCCATTACACCCCTTGATTTGC  2218


Seq_1  1804  TGCCTCTACCCATGCAAGAGCGCGCCGACATGGAGGCATCCAAGAGGCAGATTTTGTGAG  1863
             |||||||||||||| |||||||||||||||||||||||||||||||||||||||||||||
Seq_2  2219  TGCCTCTACCCATGTAAGAGCGCGCCGACATGGAGGCATCCAAGAGGCAGATTTTGTGAG  2278


Seq_1  1864  GAAGATCCATGAGAAGACAAAAGAAGCAATTGAGAAGAAAGGCAAGTACACCGCTGACCG  1923
             ||||||||||||||||||||||||||||||||||||||||||||||||||||||||||||
Seq_2  2279  GAAGATCCATGAGAAGACAAAAGAAGCAATTGAGAAGAAAGGCAAGTACACCGCTGACCG  2338
```

```
Seq_1  1924  TGTGAACAAGAAGCGCAAGGAGGTGTTGTTTTAGCCCGGCGACATGGTTTGGGTACACTA  1983
             |||||||||||||||||||||||||||||||||||||||||||||||||||||| |||||
Seq_2  2339  TGTGAACAAGAAGCGCAAGGAGGTGTTGTTTTAGCCCGGCGACATGGTTTGGGTGCACTA  2398

Seq_1  1984  TCGCAAGGACAGATTTCCAGAGCGTCGTAAGTCCAAGTTGCAACCACGTGGTGCTGGACC  2043
             ||||||||||||||||||||||| ||||||||||||||||||||||||||||||||||||
Seq_2  2399  TCGCAAGGACAGATTTCCAGAGCATCGTAAGTCCAAGTTGCAACCACGTGGTGCTGGACC  2458

Seq_1  2044  ATATAAAGTGCTTGCCAGGATTAATAACAATGCATACAAGATTGATCTTCCAACCGATGA  2103
             ||||||||||||||||| ||||||||||||||||||||||||||||||||||||||||||
Seq_2  2459  ATATAAAGTGCTTGCCAAGATTAATAACAATGCATACAAGATTGATCTTCCAACCGATGA  2518

Seq_1  2104  GTTTGGCGTTAGTAATACATTAAATGTTGCTGATTTGACGCCGTATGCTGGAGAAGACCT  2163
             ||||||||||||||||||||| ||||||||||||||||||||||||||||||||||||||
Seq_2  2519  GTTTGGCGTTAGTAATACATTCAATGTTGCTGATTTGACGCCGTATGCTGGAGAAGACCT  2578

Seq_1  2164  TGTTGTGTCGGGGTCGACGCCTTTTCAAGGGGGGGAGGATGATGAGGACATCCCTAGTTC  2223
             |||||||||||||||||||||||||||||| |||||||||||||||||||||||||||||
Seq_2  2579  TGTTGTGTCGGGGTCGACGCCTTTTCAAGGCGGGGAGGATGATGAGGACATCCCTAGTTC  2638

Seq_1  2224  TTTATCATCAACTCCAAATGATGATGTTGCTGCACAAGACAAGCCCAATGAAGTTAGACT  2283
             ||||||||||||||||| |||| |||||||| ||||||||||||||||||||||||||||
Seq_2  2639  TTTATCATCAACTCCAATTGATAATGTTGCTACACAAGACAAGCCCAATGAAGTTAGACT  2698

Seq_1  2284  TGGGCCAATAACAAGAGCACGTGCGAAGCTACTTGAACAACAGGTGAACTCACTCTTAAT  2343
             ||||||||||||||||||||||||||||||||||||||||||||||||||||||||||||
Seq_2  2699  TGGGCCAATAACAAGAGCACGTGCGAAGCTACTTGAACAACAGGTGAACTCACTCTTAAT  2758

Seq_1  2344  TGAATCTGATGTTTTAATTGATGAGAGCTTTATACTACCTAAGTCTATGCATTTATGTAT  2403
             |||||||||||||||||||||||||||||||||||||||||||||| |||||||||||||
Seq_2  2759  TGAATCTGATGTTTTAATTGATGAGAGCTTTATACTACCTAAGTCTCTGCATTTATGTAT  2818

Seq_1  2404  GATCAGATTTGAAGATGTTTCAAGCCTTGCACGTGGAGGATGAGAGTTGTAGCAAGATCC  2463
             ||||||||||||||||||||||||||||||||||||||||||||||||| ||||||||||
Seq_2  2819  GATCAGATTTGAAGATGTTTCAAGCCTTGCACGTGGAGGATGAGAGTTGCAGCAAGATCC  2878

Seq_1  2464  ATTCCCGTTGATTTCCAATATCAAGAAGAGCGCGAGGGAGGAGAGGGAGGCCGGCGCACC  2523
             |||||||||||||||||||||||||||||||||||||    |||||||||||||||||||
Seq_2  2879  ATTCCCGTTGATTTCCAATATCAAGAAGAGCGCGAGG----AGAGGGAGGCCGGCGCACC  2934

Seq_1  2524  ACATGAAGAGGAGGAAATCAACCAGCAATAGAAGTTATCGTGGCTGATGCTAGGACAAGT  2583
             |||||||||||||||||||||||||||||||||||| |||||||||||||||||||||||
Seq_2  2935  ACATGAAGAGGAGGAAATCAACCAGCAATAGAAGTTGTCGTGGCTGATGCTAGGACAAGT  2994

Seq_1  2584  CCTATGACACGTCAAACATGTCTAAGGCATCAGCGCACGTCGATGAGCATGTGACACACA  2643
             ||||||||||||||||||||||||||||||||||||||||||||||||||||||||||||
Seq_2  2995  CCTATGACACGTCAAACATGTCTAAGGCATCAGCGCACGTCGATGAGCATGTGACACACA  3054

Seq_1  2644  TAGGGTTGCCTACGACATGGTTTATTTGCCTAAGTGTGAGTTGGGCCTGCGTGCTCGTAG  2703
             ||||| ||||||||||||||||||||||||||||||||| ||||| ||||||||||||||
Seq_2  3055  TAGGGCTGCCTACGACATGGTTTATTTGCCTAAGTGTGAATTGGGTCTGCGTGCTCGTAG  3114

Seq_1  2704  ATTGGGCAACCTTGACTAGCAATGTGACTCAGTATGATGACTATGTTTCACAAGTTATAG  2763
              |||||||||||||||||||||||||||||||||||||||||||||||||||||||||||
Seq_2  3115  TTTGGGCAACCTTGACTAGCAATGTGACTCAGTATGATGACTATGTTTCACAAGTTATAG  3174
```

```
Seq_1  2764  GGCAAGGTGTTGCCAACTGATAGCATTGGACTCGACAATGAGATTGCCGGGTCAGACAGA  2823
             ||||||||||||||||||||||||||||||||||||||||||||||||| ||||||||||
Seq_2  3175  GGCAAGGTGTTGCCAACTGATAGCATTGGACTCGACAATGAGATTGCCGAGTCAGACAGA  3234

Seq_1  2824  CCCGCACTGAGACACAACGAGATGATTGTCATTTGTTAGTCTTAAGTATGATGTTTATGC  2883
             ||||||||||||||||| |||||||||||||||||||||||||||||||| |||||||||||
Seq_2  3235  CCCGCACTGAGACACAAGGAGATGATTGTCATTTGTTAGTCTTAAGTACGATGTTTATGC  3294

Seq_1  2884  CAGTTCTAGAACTGAGGTCATCGCATGAGCTTGGGATGTGAATCGGCTTACTTAGGGGTT  2943
             |||| |||||||||||||||||||||||||||||||||||||||||||||||||| ||||
Seq_2  3295  CAGTCCTAGAACTGAGGTCATCGCATGAGCTTGGGATGTGAATCGGCTTACTTAGAGGTT  3354

Seq_1  2944  ACCAAACGCTACTCCATAACTGGGTAGTTATAAAGGAAGCTTTTGGGTTTGCTGAGAAGC  3003
             ||||||||||||||| |||||||||||||||||||| |||||||||||||||||||||||
Seq_2  3355  ACCAAACGCTACTCCGTAACTGGGTAGTTATAAAGGTAGCTTTTGGGTTTGCTGAGAAGC  3414

Seq_1  3004  ATGCTGCAAGTCATGGTTGATCAAGATGGATTTGCCCCTCCCTTTGGGAGAGATATCTCT  3063
             ||||||||||||||||||||||||||||||||||||||||||||||||||||||||||||
Seq_2  3415  ATGCTGCAAGTCATGGTTGATCAAGATGGATTTGCCCCTCCCTTTGGGAGAGATATCTCT  3474

Seq_1  3064  GGGCCCTCTCGAGTGATCAGATTCGGAAAGCATGGCCATGCGACTTGGGTTAAGCATTAA  3123
             ||||||||||||||||||||||||||||||||||||||||||||||||||||||||||||
Seq_2  3475  GGGCCCTCTCGAGTGATCAGATTCGGAAAGCATGGCCATGCGACTTGGGTTAAGCATTAA  3534

Seq_1  3124  CCCGTTCGGGAATCTGAATCACGAGATTGAGAAGAGAGTCGAGTTATACACAAGGATGAC  3183
             ||||||||||||||||||||||||||| ||||||||||||||||||||||||||||||||
Seq_2  3535  CCCGTTCGGGAATCTGAATCACGAGATCGAGAAGAGAGTCGAGTTATACACAAGGATGAC  3594

Seq_1  3184  AAGTACTCGCCTTGAGCTCGACACACATATCGTGAGGCAGAAGGAATGTTGCATGTGGAC  3243
             ||||||||||||||||||||||||||||||||||||||||||||||||||||||||||||
Seq_2  3595  AAGTACTCGCCTTGAGCTCGACACACATATCGTGAGGCAGAAGGAATGTTGCATGTGGAC  3654

Seq_1  3244  ACATTGTATAGTTCGTCAATATACTTTGTGGTTATTCAGGAGTCAGCACGTGCTGCTCGG  3303
             ||||||||| ||||||||||||||||||||||||||| ||||||||||||||||||||||
Seq_2  3655  ACATTGTATGGTTCGTCAATATACTTTGTGGTTATTCGGGAGTCAGCACGTGCTGCTCGG  3714

Seq_1  3304  CGCCGCTGCTGACTATCGACTTGGGTCGGAACCTGTGTTCGGACTCGTAGTTGTGTCCGT  3363
             ||||||||||||||||||||||||||||||||||||||||||||||||||||||||| ||
Seq_2  3715  CGCCGCTGCTGACTATCGACTTGGGTCGGAACCTGTGTTCGGACTCGTAGTTGTGTCTGT  3774

Seq_1  3364  TTGACCACGAACCTGTAGGGTTGCACACTTAAGGGGATGGGGCCGAATTGGATCGGATCC  3423
             |||||| |||||||||||||| ||||||||||||||||||||||||||||||||||||||
Seq_2  3775  TTGACCGCGAACCTGTAGGGTCGCACACTTAAGGGGATGGGGCCGAATTGGATCGGATCC  3834

Seq_1  3424  AACTCGTATCCCGCTTACACTCCTGATACGGGTGCGGGTCGCCCGATCTTTCGATGAGAT  3483
             |||||||||||||||||
Seq_2  3835  AACTCGTATCCCGCTTA-------------------------------------------  3851

Seq_1  3484  TGATAACTCTCGATTTGGGTAGGAGGTGACGTTGACGATCCGACTACGGCCTAGGAGGCA  3543

Seq_2  3852  ------------------------------------------------------------  3851

Seq_1  3544  GCGTCTTAGCAATCGATACGCCAACTCCAGAAGGTTATTAATCACGCGGGGGCACGATCA  3603

Seq_2  3852  ------------------------------------------------------------  3851
```

```
Seq_1  3604  ACCTGACCACGAAGGTCTTTGTTCCTGCAAGCAATCGAAGAACAAGCAAGAACAAGATAA  3663

Seq_2  3852  ------------------------------------------------------------  3851


Seq_1  3664  ATAGCGGATGCAATCTCAAATTGCGAGTATACTATATCAAACCAATGTCTCAACAAGACG  3723

Seq_2  3852  ------------------------------------------------------------  3851


Seq_1  3724  ATAAGTTGGAGTCTTGACGACGGTAAAACAGGTGGTCTAACCGACACACGCGATTACACG  3783

Seq_2  3852  ------------------------------------------------------------  3851


Seq_1  3784  AAAGTAGCAAAGATGGCTAAACTTTATC-TAATCAAAACCC--AAGGCTCCTTAGGGGGG  3840
                               |  | | |      ||||       ||  | ||   | ||
Seq_2  3852  -----------------GACTCCTAACGGGCCTCAAGTGTTGAAACCCACCAGGGAGGTC  3894


Seq_1  3841  GGC-TCA-TGGGGTATATAAAGGAGGAGGGAGAGATATTTTCGTCCACCTTGAGTAAGGA  3898
                 | | ||| | |   | |   ||   | |  |             |  |||| ||
Seq_2  3895  TATATAAGTGGAGGAGGCATACCTGGTTAGGGTTACCCCAATTCGTGGCACGAGTCAGAC  3954


Seq_1  3899  GGCCGAAATCCAACTCTATCTCTAAGTTTCCTAACAAACTACAACTCTTTAGGAAACAGA  3958
                       || ||| |   | ||   |    |     |  | |  |       || ||
Seq_2  3955  TCGGCCGTCACATCTCCACGCCCAAACCTTGCGATCGGATCTAGCAGTCCGCCGCACGGA  4014


Seq_1  3959  AAAACAGATCCGTCAGCTTGTTTTGTCCGTCACGGTCAGCACGAGTTGAATCTATTGATG  4018
                 |            | | |   || |   || |      | |  |  |  ||  |
Seq_2  4015  GTTCCTCTCTATATGTGTGGATACCTCAGAGGCGCTGCATCTGCGGCGCTTGGATGAACT  4074


Seq_1  4019  GGACCAGATCCGTTGGAAAGGTTTTGTAATTACCTTTCCAACAAGTACTTGTTTGCTTGA  4078
             |  |  |        ||              | |       |              |
Seq_2  4075  GTTCATGGGATTGGCGAGGACGAACAGGCCGATCGACTACTCGGCATTGACACGCATCAC  4134


Seq_1  4079  TTTGGACTCCGGATACGGCCTAGGTGATTAAAACAAATTCGGGTCTCCTGGCAGCTCGGA  4138
             |       |                       |    |       |  |      ||
Seq_2  4135  TGACTCTACTTCTGTTATGGGTCTGCGCGTCTAGTGGTAATCTCGTGATCCATTATCTAC  4194


Seq_1  4139  TCCGAATCGGATTCAACTTTAATTCGTGATATCTTTCTCTAGCAAGCTCCGAATCATGTG  4198
               |                | |      | |  ||  | |               ||
Seq_2  4195  AGCATTGATCCGGGTGGAATCAGGTAAAAAAATTTGTTTTGTGCTCGCGTAGCCTATCGT  4254


Seq_1  4199  CCGTTTGATTTGTTGGAAAGTAGACTTGATAGGCTTCACTTTGAATCTCCCTTTGCAGGC  4258
                    |    |        |    |  |  |     | |    |    ||| |  ||
Seq_2  4255  GTTCCCCAACAATAACCCCTAAAGTATATTGTGAGCACCATGATTTGAATCTTGGTGGGT  4314


Seq_1  4259  TATCACACTTCATCTTCACTTTGGATTTGTAAAGTTCATTAAGATGTCTGCATAATAAGA  4318
                  ||      |   |||      |   |        ||  ||     || |  ||
Seq_2  4315  GGAGTCATGCACCCACAACTCCACCATCACACCAAGAGGTATTATTCTATCAAATCAAAC  4374


Seq_1  4319  TTACACAAGATAGTAGCTCCGCCTCATTGCAAGTAACATATTGAAAACATTTTGTAATTG  4378
                  ||    | ||  || | |   ||   | |     |         |     | ||
Seq_2  4375  AATGCCATCTCATTAATTCAGTCATGTTTTCATTTTTTGAGACCCTCGGTACATGATTTT  4434


Seq_1  4379  AATTCACCTTATTATAATTGTTATTAGATACACCAACAATTAGGGTTCTAATGGTCGTAT  4438
                ||| | |   |  ||      |   ||  || || |   |    |||    |   |
Seq_2  4435  TTCTCAACATGCAAGTATGTCACCTTATTAGGCCCACCACATGCAAACTATGAATGCGAG  4494
```

```
Seq_1  4439  CCATGGTGATCATGTCCATATCATCCTCCCTTTCTTGAAAGAAAAGCCGTCCTCGGCGTC  4498
                      ||||     |||     |   ||| |||||   |     ||  ||
Seq_2  4495  TATGCCACCTCATTAGGTTATGTATGTATTTTTTTTGAAGCCATGCATGTATTCAC----  4550

Seq_1  4499  GTTTGTGCTGAAAAGAGACTGACAAAATAGACAAAGTGTGTGCATGTTATATGTATATGT  4558

Seq_2  4551  ------------------------------------------------------------  4550

Seq_1  4559  CATCAATTTTACATTTTTGTATTGTTCTATAAATTTAGATCACGCACCTCATCAATTCAA  4618
                     |||||||||||||||||||||||||||||||||| |||||||||||||||||
Seq_2  4551  --------TTACATTTTTGTATTGTTCTATAAATTTAGATCATGCACCTCATCAATTCAA  4602

Seq_1  4619  TCATGTTTTCTTTTTTTT-AAAGTTTTTCTCCTTTTATACACATTTTGTCGCATGCTTCT  4677
             ||||||||||||||||||  ||||||||||| ||||||||||||||||||||||||||||
Seq_2  4603  TCATGTTTTCTTTTTTTTTTAAGTTTTTCTCTTTTTATACACATTTTGTCGCATGCTTCT  4662

Seq_1  4678  TAGGTTTAGTTGTTTTCTAAATGTAAAATCAGAACATTTTTATATCTTTTTCCATTAATT  4737
             ||||||||||||||||||||||||||||||||||||||||||||||||||||||||||||
Seq_2  4663  TAGGTTTAGTTGTTTTCTAAATGTAAAATCAGAACATTTTTATATCTTTTTCCATTAATT  4722

Seq_1  4738  GTAGTTAATTTAAAACATTTATTTATACATTGTTGGTGCATCATCTGTTTTTGACGGTTG  4797
             |||||||||||||||||||||||||||||||||||||||||||||||||||||||| |||
Seq_2  4723  GTAGTTAATTTAAAACATTTATTTATACATTGTTGGTGCATCATCTGTTTTTGACGATTG  4782

Seq_1  4798  TTGGTGCATCATCTAGTCGTTGTACAATTCTACGATCCATTTCTCCGTTCCTGCACCTGA  4857
             ||||||||||||||||||||||||||||||||||||||||||||||||||||||||||||
Seq_2  4783  TTGGTGCATCATCTAGTCGTTGTACAATTCTACGATCCATTTCTCCGTTCCTGCACCTGA  4842

Seq_1  4858  ACGGAGCAACAATGTCTTTGCACGAATGGTTCATCGTGTGATCGGACGGTCGAGAATCGA  4917
             ||||||||||| ||||||||||||||||||||||||||||||||||||||||||||||||
Seq_2  4843  ACGGAGCAACAGTGTCTTTGCACGAATGGTTCATCGTGTGATCGGACGGTCGAGAATCGA  4902

Seq_1  4918  GGGTGAAACAACGGAAGGACTGGAAAACAACCGCGCAATAACACCAGCCACTTCCTTGTA  4977
             |||||||||||| ||||||| |||||||||||||||||||||||||||||||||||||||
Seq_2  4903  GGGTGAAACAACAGAAGGACCGGAAAACAACCGCGCAATAACACCAGCCACTTCCTTGTA  4962

Seq_1  4978  AAATCACCCCACAATCCGAAGCTTCTCCCCCCTTATTTCCTTCGCGACGAAGCGGCACGC  5037
             |||||||||||||||| |||||||||||||||||||||||||||||||||||||||||||
Seq_2  4963  AAATCACCCCACAATCTGAAGCTTCTCCCCCCTTATTTCCTTCGCGACGAAGCGGCACGC  5022

Seq_1  5038  AGCTGCAACGACTTGCAAGACAGAAAACCTACCAAATCAAACCAAACCGCAACCAAATCT  5097
             ||||||||||||||||||||||||||||||||||||||||||||||||||||||||||||
Seq_2  5023  AGCTGCAACGACTTGCAAGACAGAAAACCTACCAAATCAAACCAAACCGCAACCAAATCT  5082

Seq_1  5098  CCCAAATCCAAATCCCCACCGTCGCGCCCTACCTCCGACTATATCCGCTGC-----T---  5149
             |||||||||||||||||||||||||||||||||||||||||||||||||||     |
Seq_2  5083  CCCAAATCCAAATCCCCACCGTCGCGCCCTACCTCCGACTATATCCGCTGCCGCCCTCCT  5142

Seq_1  5150  ------------------------------------------------------------  5149

Seq_2  5143  ATAGCTAGGCAGACATCTCCCGCTGCTGGGAGCGACACGGAGCCGGCCAAGGGGCTACTG  5202

Seq_1  5150  ------------------------------------------------------------  5149

Seq_2  5203  CTCCCCTACCTGCAATGCGACTTTGCAGGACTTCAATACCGAATTTTCGTAAGGGACCAA  5262
```

```
Seq_1  5150  ------------------------------------------------------------  5149

Seq_2  5263  TGTTTTCTGTCATGACTCAGGAGCATCTTGGTGTACATGTATGTTTGTTCGACTCAGTTC  5322

Seq_1  5150  ------------------------------------------------------------  5149

Seq_2  5323  ATCTTTCCTAATTGTTAACACACATATCTATTTTTGTCCAGGATGCCAGTGCAACGTTAT  5382

Seq_1  5150  ------------------------------------------------------------  5149

Seq_2  5383  GCAACAAACAATATTCTACCTCCCCTCGGCTCTTTCAGCAAAAGAAGTGCACAATAGGTA  5442

Seq_1  5150  ------------------------------------------------------------  5149

Seq_2  5443  CGTGGATGTTCTTACAAAGATGAAGAATACAACACAACAACCAATGTGGACCACTTAAAA  5502

Seq_1  5150  ------------------------------------------------------------  5149

Seq_2  5503  AACGTCTCACATTTGAACTATCTCATGTCCATCCTCTCAACAATATCAGTTCCATCTTCA  5562

Seq_1  5150  ------------------------------------------------------------  5149

Seq_2  5563  AGTACATCATGAAAGGTACATCACATGTACAATTTTTTGAGTGGAGAAGGGACTAAGACC  5622

Seq_1  5150  ------------------------------------------------------------  5149

Seq_2  5623  GACAACAATAAACAAATCTTGCCATATATTTCCTGATTTAATTTATTAAAATCCCAGCAA  5682

Seq_1  5150  ------------------------------------------------------------  5149

Seq_2  5683  AACATATAATAGTAGTCCAAATATTGCTCTTCAATACAACATAGGTTCCCGCTGCAACGC  5742

Seq_1  5150  ------------------------------------------------------------  5149

Seq_2  5743  GCGGAGTCTCGTTAGTTTATACGTTATTGGCGGATCGTCTGTTTTTGACAGCTGTTGGTG  5802

Seq_1  5150  ------------------------------------------------------------  5149

Seq_2  5803  CATCATCTAGTTGCCGTACAATTCTAGCATCCAGTTCTCCGTTCCTGCATCCGAACGGAG  5862

Seq_1  5150  ------------------------------------------------------------  5149

Seq_2  5863  TAACGGTGTCTTTGCACGAACAGTTCATCGTGTGATCGGACGGTCGAGACTCGAGGGTGA  5922

Seq_1  5150  ------------------------------------------------------------  5149

Seq_2  5923  AACAGCGGAAGGACTGGGAAACAACCGCGCAATAACACCAGGCACTTCCTTGTAAAATCA  5982

Seq_1  5150  ------------------------------------------------------------  5149

Seq_2  5983  CCCCACGATCCGAAGCTTCTTCCCCTCATATTCCCGACGAAGCAGCACGCAGCTGCAACG  6042

Seq_1  5150  ------------------------------------------------------------  5149

Seq_2  6043  ACTTGCAAGACAGAAAACCTACCAAATCAAACCAAACCACAACCGAATCTCCCGAATCCA  6102
```

```
Seq_1  5150  ------------------------------------------GCCCTCCTGTAGCTAGGC  5167
                                                       ||||||||||||||||||
Seq_2  6103  AACCCCCACCGTTGCGCCCTACCTCCGGCGATATCTGCCGCCGCCCTCCTGTAGCTAGGC  6162

Seq_1  5168  AGACATCTCCCGCTGCTGGGAGCGACACGGAGCCGGCCAAGGGGCTACTGCTCCCCTACC  5227
             ||||||||||||||||||
Seq_2  6163  AGACATCTCCCGCTGCTG------------------------------------------  6180

Seq_1  5228  TGCAATGCGACTTTGCAGGACTTCAATACCGAATTTTCGTAAGGGACCAAATGTTTTCTG  5287

Seq_2  6181  ------------------------------------------------------------  6180

Seq_1  5288  TCACGACTCAGGAGCATCTTGGTGTACATGTATGTTTGTTCGACTCAGTTCATCTTTCCT  5347

Seq_2  6181  ------------------------------------------------------------  6180

Seq_1  5348  AATTGTTAACACACATATCTATTTTTGTCCAGGATGCCAGTGCAACGTTATGCAACAAAC  5407

Seq_2  6181  ------------------------------------------------------------  6180

Seq_1  5408  AATATTCTACCTCCCCCTCGGCTCTTTCAGCAAAAGAAGTGCACAATAGGTACGTGGATG  5467

Seq_2  6181  ------------------------------------------------------------  6180

Seq_1  5468  TTCTTACAAAGATGAAGAATACAACACAACAACCAATGTGGACCACTTAAAAAACGTCTC  5527

Seq_2  6181  ------------------------------------------------------------  6180

Seq_1  5528  ACAACTATCTCATGTCCATCCTTTCAACAATGTCAGTTCCATCTTCAAGTACATCATGAA  5587

Seq_2  6181  ------------------------------------------------------------  6180

Seq_1  5588  AGGTACATCACATGTACAATTTTTTGAGTGGAGAAGGGACTAAGACCGACAACAATAAAC  5647

Seq_2  6181  ------------------------------------------------------------  6180

Seq_1  5648  AAATCTTGCCATATATTTCCTGATTTAATTTATTAAAATCCCAGCAAAACATATAATAGT  5707

Seq_2  6181  ------------------------------------------------------------  6180

Seq_1  5708  AGTCCAAATATTGCTCTTCAATACAACATAGGTTCCCGCTGCAACGCGCGGGGTCTCGTC  5767

Seq_2  6181  ------------------------------------------------------------  6180

Seq_1  5768  TAGTTTATACGTTATTGGCGGATCGTCTGTTTTTGACAGCTGTTGGTGCATCATCTAGTT  5827

Seq_2  6181  ------------------------------------------------------------  6180

Seq_1  5828  GCCGTACAATTCTAGCATCCAGTTCTCCGTTCCTGCATCCGAACGGAGCAACGGTGTCTT  5887

Seq_2  6181  ------------------------------------------------------------  6180

Seq_1  5888  TGCACGAACAGTTCATCGTGTGATCGGACGGTCGAGACTCGAGGGTGAAACAGCGGAAGG  5947

Seq_2  6181  ------------------------------------------------------------  6180
```

```
Seq_1  5948  ACTGGGAAACAACCGCGCAATAACACCAGGCACTTCCTTGTAAAATCACCCCACGATCCG  6007

Seq_2  6181  ------------------------------------------------------------  6180


Seq_1  6008  AAGCTTCTTCCCCTCATCTTCCCGACGAAGCAGCACGCAGCTGCAACGACTTGCAAGACA  6067

Seq_2  6181  ------------------------------------------------------------  6180


Seq_1  6068  GAAAACCTACCAAATCAAACCAAACCACAACCGAATCTCCCGAATCCAAACCCCCACCGT  6127

Seq_2  6181  ------------------------------------------------------------  6180


Seq_1  6128  TGCGCCCTACCTCCGGCGATATCTGCCGCCGCCCTCCTGTAGCTAGGCAGACATCTCCCG  6187

Seq_2  6181  ------------------------------------------------------------  6180


Seq_1  6188  CTGCTG  6193

Seq_2  6181  ------  6180
```

BdindelWSU_3, UPSTREAM

>Bradi1g45820

TTTTAATATGCATTGAGGCTTTGATTCCGTTTTCAGCTTCCCGCTTCAGTCTCGTGTTGAAGATAGTTGTAATCTTGGTTCCGACATTAG
AATCGGGGACCTAAGATGTTTTTGGGGTCTGTGGTACTTGTCACGATTTTGTTTAGATACGCATATATCTATATAGTGAAACGCATCTA
CATACATGTGTATCTAAACAAAGCTACAACAATTAATATTGATGGGAGAAATATTTTACTTTTATATTTTTTTAGTCCAAAAACGTCTAA
GCATGGATATTATACTAGTCAAGGGATCCAAGTAGTACAATGATGCGAGATGGACATCTACCTTACCTTTGTATGCATATACTCCATC
CGAAACTTTGTATTAAATTGGCAACACTTATTATGGATCGGAGAAAGTATATTCTATCGGATGTCCCTGAAGGGACACATTATCCATT
GTAGGCCCTAAATCTTTTGTAAAGGTGTCAAGTTAGTTCTAATACTTTTAAAATGCACTTTTGGTTCCTAATACTTTCATAAGTGGTTCA
TCTCAGGTCCTAAGTATGTGTCTCTAGATGTAACAATGTCACTTGGCTATTTGGACCCACAAGTCGGTTGACAAGCTGGATTTTCTTTT
GCCAAAAGAAAACCCTGCCTCCTTCTCGTTTCTCACCCGTGGTCCTTTTCTTTCTATGCTTTGCGGCTCTGGCCCTCTCTTGAATGTCCC
GCCCAGCTATTTCGCCAGAATATCCTTCACCGTTGGTCTCCTCGGCAGCACCGGATATTTTGTGAAGAGAGCAGAGAAGGGGAAGGG
CCACGAGTAGGAAATGAGAAGAAGTCAGTTTTTTTCTTCAAAGAATTCCCATTGAAGGGTTATATGATAGGTGGATTCAATCCATGTG
GCTGGCCATGTCGACAGATCAGACGCACACAAACAATATTGGGACATGAGACGTACAAATTATTAAACTATTATGACTCAATCGCGC
ATTTTGAAAGTATTAGAACTAATTTGACACATTTAAAAAAATTAGAACATACATTGTATTTTTATTTTTTTATTATGGATCGGAGGAAG
TATGTTCTACCATAGAGACACATTATCCATTTGCCACTTACAGGTATACTGATTCAAGCAGTGTAGTATACGAAACCGTCCATATCACT
TTTTGTGGGTTTTATGATTTTGTGGGTTTTATGATCTATATCAGCATGAAAATTATTATTCCGTATCAGAGCACGCCAGAATTTTGTTGA
CTATCGTTTGGTGCTGAATTTTTTACCGAGGATAGATCTTGAGTTCTTGACAAACCAAACATACCCGAAGTCGTTCCTGGGCACTCTTC
CTGGGAGAAAAATCCTCTATGACCTGGGTCGTTCTCCCTCCATGGCTAGACCACACTGAACGATCGACACCTGGCAAACCGAATCTTG
AAGCAATCCTGATTCTCTTCCTCGTTGAATCCTCCTTCCCCTTTCGTCCAAGCACGCAGGTAAGCAGGTTCCCATTCCTTTCTCCAAGCG
CCGGCCCTAAGCCTTGGATCCCCAGCTACCTCCTCGACGCTGCCGCTTCGTCTCCGCCGATCTTCCCACCGGCGACAACCATGCTCCTA
GGCGCCATGGCTGCTCATCCCTGCCGGCACAACTCCCCTGCTCGCTGCGTCGCAGGACCTTCGTCGCTCCGTCTCCGCTACTCCCACCC
GGTCCAGACCACGCTCCCCGGCGTCCAGCAAACGTATCGCAGGGGAGTTGTGCCGGCAGGGATGAACAGACATGGCGCCGAGGAG
CATGGTTGTCGCCAGCGGGAAGATCGGCGGAGACGAAGCGGCAGTGTCGAGGAGGTAGCTGGGGATCCAAGGCTTAGGGCCGGC
GCTTGGAGAAAGGAACGGGAACCTGCTTACCTGCGTGCTTGGACGAAAGGGGAAGGAGGATTCAGCGAGGAAGAGAGTCAGGATT
GCTTCGAGATTCGGTTTGCCAGGTGTCGATCGTTCAGTGTGGTCCAGCCATGGAGGGAGAACGACCCAGGTCGTAGAGGATTTTTCT
CCCTCTTCCTGGGCCACCCTCCACATCGAGCAATTCTGCTCTACACTGCATCCTCCTCCGCCGCGCTCCGACACCTCAGCCTCCTCCCCC
TCGCCGGCGCCGCCATCCACCATCCGCGCAACTCGCGCCACCGCCGTCTAACACCCACTCGATCTGGTGTATTAGTTCCTCCCAAGAC
AGCGCACCCCGGGCGGCG

>BdiBd21-3.1G0600100

TTTCGAAGTACTAAAAGGTAGTTGCACCAGTCTAGATCACGGAAGCTTTCCAGGTCAAGAGAGTTTCTCCTTGTAAATGTTACTCCGT
AGTAATTTTGCTGCAGTTGTCATCCATTTAAGCCGGCTGGCTATATTCTGATATTTTTTCTCGTAGGGTACGATCATTGTGCTGAAAGG
CATTGCTTTGACAACAAAAATCTGACAATAGCATGGGGACATCTCACGCGTTATTTGGTATTTTCTCCTGACATAGCCACGTTCCAGTT
CGGTTCAGATCTGTAGTATTTTTCAAGTAACAAAAAAAACTCATCGAATTTATAAGAAATTCAGAAGTTCTGGCAAATCATTCTTTTCG
GTGTAATGTGTTAGTGTTACCTTTGTCTTCTTTGGAAGGGAAAAGTCTTTAGCTTTATGGGGATGAAAGACGCACAAAACATAGTACA
GTAATTATATTTTCCGTTTATTTTCTTTTATACTTGTATTTTACTGCTACTATTTCGGTTTCAAATAGGCCAAAATAGCCTACCAAAACAT
CTTATATTTAGAAACAGATTGTAGTATATACTCTCTTTGTCCCATATTAAGTGCTAAAATATTACATGTATCTAAACGTATTTTAGTATA
TAAATATGTTTATACTTAGAAAAATTTGAGTCACTTAATATGAGACAAAGCGAGTATTTGGTCACTCATCCTACTCTTAAAAGTTAAAA
CTATTTTTAATATGCATTGAGGCTTCGATTCCGTTTTGAGCTTCCCGCTTCAGTCTCGCGTTGAAGATAGTTGTAATCTTGGTTCCGACA
TTAGAATGGGGGACCTAAGATGTTTTTGGGGTCCGTGGTACTTGTCACGATTTTGTTTAGATACGCATATACCTATATAGTGAAACGC
ATCTACGTACATGTGTATCTAAACAAAGCTACGACAATTAATATTGATCGGAGAAATATTTTACTTTTGTATTTTTTTAGTCCGAAAAA
CGTCTAAGCATGGATATTATACTAGTCAAGGGATCCAAGTAGTACAATGATGCGAGATGGACACCTACCTTACCTTTGTATGCATATA
CTCCATCCGAAACTTTGTATTAAATCGGCAACACTTATTATGGATCGGAGAAAGTATATTCTATCGGATGTCCTTGAAGGGACACATT
ATCCATTGTAGGTCCTAAATCTTTTGTAAAGGTACCAAGTTAGTCTTAATACTTTTAAAATGCACTTTTGGTTCCTAATACTTTCATAAG
TGGTTCATCCCAGGTCCTAAGTGTGTGTCTCTAGATGTAACAATGTCACTTGGCTATTTGGACCCACATGTCGGTTGACAAGCTGGAT
TTTCTTTTGCCAAAAGAAAACCCTGCCTCCTTCTAGTTCCTCACCCGTGGTCCTTTTCTTTCTATGCTTTGCGGCTCTGGCCCTCTCTTGA
ATGTCCCGCCCAGCTATTTCGCCAGAATATCCTTCACCGTCGGTCTCCTCGGCAGCACCGGATATTTTGTGAAGAGAGCAGAGAGGG
GGAAGGGCCACGAGTAGGAAATGAGAAGAAGTCAGTTTTTCTTCTTCAAAGAATTCCCATCTAAGGGTTATATGATAGGGTTATATG
ATAGGTGGATTCAATCCATGTGGCTGGCCACATCGACAGATCAGACGCACACAAACAATATTGGGACATGAGACGTATAAATTATTA
AACTGTTATGACTCAATCACGCATTTTGAAAATATTAGAACTAATTTGACACATTTAAAAAAATTAGAACATACATTGTATTTTTATTTT
TTTATTATGGATCGGAGGAAGTATGTTCTACTCATAGAGACACATTATTCATTTGCCACTTACAGGTATACTGATTCAAACAGTGTAGT
ATACGAAACCGTCCATATCACTTTTTGTGGGTTTTATGATCTATATCAGCATGAAAATTATTATTCCGTATCAGAGCACGCCAGAATTT
TGTTGACTATCGTTTGGTGCTGAATTTTTTACCGAGGATAGATCTTGAGTTCTTGACAAACCAAACATACCCGAAGTCGTTCCTGGGCA
CTCTTCCTGGGCCACCCTCCACATCGAGCAATTCTGCTCTACACTGCATCCTCCTCCGCCGCGCTCCGACACCTCAGCCTCCTCCCCCTC
GCCGGCGCCGCCATCCACCATCCGCGCAACTCGCGCCACCGCCGTCTAACACTCACTCGATCTGGTGTATTAGTTCCTCCCAAGACAG
CGCACCCCGGGCGGCG

```
Alignment of Sequence_1:  [Sequence Window #3] with  Sequence_2: [Sequence Window #4]

Similarity : 1468/2237 (65.62 %)

Seq_1  1     ------------------------------------------------------------  0

Seq_2  1     TTTCGAAGTACTAAAAGGTAGTTGCACCAGTCTAGATCACGGAAGCTTTCCAGGTCAAGA  60


Seq_1  1     ------------------------------------------------------------  0

Seq_2  61    GAGTTTCTCCTTGTAAATGTTACTCCGTAGTAATTTTGCTGCAGTTGTCATCCATTTAAG  120


Seq_1  1     ------------------------------------------------------------  0

Seq_2  121   CCGGCTGGCTATATTCTGATATTTTTTCTCGTAGGGTACGATCATTGTGCTGAAAGGCAT  180


Seq_1  1     ------------------------------------------------------------  0

Seq_2  181   TGCTTTGACAACAAAAATCTGACAATAGCATGGGGACATCTCACGCGTTATTTGGTATTT  240


Seq_1  1     ------------------------------------------------------------  0

Seq_2  241   TCTCCTGACATAGCCACGTTCCAGTTCGGTTCAGATCTGTAGTATTTTTCAAGTAACAAA  300


Seq_1  1     ------------------------------------------------------------  0
```

```
Seq_2  301   AAAAACTCATCGAATTTATAAGAAATTCAGAAGTTCTGGCAAATCATTCTTTTCGGTGTA  360

Seq_1  1     ------------------------------------------------------------  0

Seq_2  361   ATGTGTTAGTGTTACCTTTGTCTTCTTTGGAAGGGAAAAGTCTTTAGCTTTATGGGGATG  420

Seq_1  1     ------------------------------------------------------------  0

Seq_2  421   AAAGACGCACAAAACATAGTACAGTAATTATATTTTCCGTTTATTTTCTTTTATACTTGT  480

Seq_1  1     ------------------------------------------------------------  0

Seq_2  481   ATTTTACTGCTACTATTTCGGTTTCAAATAGGCCAAAATAGCCTACCAAAACATCTTATA  540

Seq_1  1     ------------------------------------------------------------  0

Seq_2  541   TTTAGAAACAGATTGTAGTATATACTCTCTTTGTCCCATATTAAGTGCTAAAATATTACA  600

Seq_1  1     ------------------------------------------------------------  0

Seq_2  601   TGTATCTAAACGTATTTTAGTATATAAATATGTTTATACTTAGAAAAATTTGAGTCACTT  660

Seq_1  1     ---------------------------------------------------------TTT  3
                                                                      |||
Seq_2  661   AATATGAGACAAAGCGAGTATTTGGTCACTCATCCTACTCTTAAAAGTTAAAACTATTTT  720

Seq_1  4     TAATATGCATTGAGGCTTTGATTCCGTTTTCAGCTTCCCGCTTCAGTCTCGTGTTGAAGA  63
             |||||||||||||||||| ||||||||||| ||||||||||||||||||| ||||||||
Seq_2  721   TAATATGCATTGAGGCTTCGATTCCGTTTTGAGCTTCCCGCTTCAGTCTCGCGTTGAAGA  780

Seq_1  64    TAGTTGTAATCTTGGTTCCGACATTAGAATCGGGGACCTAAGATGTTTTTGGGGTCTGTG  123
             ||||||||||||||||||||||||||||||| ||||||||||||||||||||||||| |||
Seq_2  781   TAGTTGTAATCTTGGTTCCGACATTAGAATGGGGGACCTAAGATGTTTTTGGGGTCCGTG  840

Seq_1  124   GTACTTGTCACGATTTTGTTTAGATACGCATATATCTATATAGTGAAACGCATCTACATA  183
             |||||||||||||||||||||||||||||||||||| ||||||||||||||||||||| ||
Seq_2  841   GTACTTGTCACGATTTTGTTTAGATACGCATATACCTATATAGTGAAACGCATCTACGTA  900

Seq_1  184   CATGTGTATCTAAACAAAGCTACAACAATTAATATTGATGGGAGAAATATTTTACTTTTA  243
             ||||||||||||||||||||||| ||||||||||||||| |||||||||||||||||||
Seq_2  901   CATGTGTATCTAAACAAAGCTACGACAATTAATATTGATCGGAGAAATATTTTACTTTTG  960

Seq_1  244   TATTTTTTTAGTCC-AAAAACGTCTAAGCATGGATATTATACTAGTCAAGGGATCCAAGT  302
             |||||||||||||| |||||||||||||||||||||||||||||||||||||||||||||
Seq_2  961   TATTTTTTTAGTCCGAAAAACGTCTAAGCATGGATATTATACTAGTCAAGGGATCCAAGT  1020

Seq_1  303   AGTACAATGATGCGAGATGGACATCTACCTTACCTTTGTATGCATATACTCCATCCGAAA  362
             ||||||||||||||||||||||| ||||||||||||||||||||||||||||||||||||
Seq_2  1021  AGTACAATGATGCGAGATGGACACCTACCTTACCTTTGTATGCATATACTCCATCCGAAA  1080

Seq_1  363   CTTTGTATTAAATTGGCAACACTTATTATGGATCGGAGAAAGTATATTCTATCGGATGTC  422
             ||||||||||||| ||||||||||||||||||||||||||||||||||||||||||||||
Seq_2  1081  CTTTGTATTAAATCGGCAACACTTATTATGGATCGGAGAAAGTATATTCTATCGGATGTC  1140

Seq_1  423   CCTGAAGGGACACATTATCCATTGTAGGCCCTAAATCTTTTGTAAAGGTGT-CAAGTTAG  481
```

```
             | |||||||||||||||||||||||||| ||||||||||||||||||||   ||||||||
Seq_2  1141  CTTGAAGGGACACATTATCCATTGTAGGTCCTAAATCTTTTGTAAAGGT-ACCAAGTTAG  1199

Seq_1  482   TTCTAATACTTTTAAAATGCACTTTTGGTTCCTAATACTTTCATAAGTGGTTCATCTCAG  541
             |  ||||||||||||||||||||||||||||||||||||||||||||||||||||| |||
Seq_2  1200  TCTTAATACTTTTAAAATGCACTTTTGGTTCCTAATACTTTCATAAGTGGTTCATCCCAG  1259

Seq_1  542   GTCCTAAGTATGTGTCTCTAGATGTAACAATGTCACTTGGCTATTTGGACCCACAAGTCG  601
             ||||||||| ||||||||||||||||||||||||||||||||||||||||||||| ||||
Seq_2  1260  GTCCTAAGTGTGTGTCTCTAGATGTAACAATGTCACTTGGCTATTTGGACCCACATGTCG  1319

Seq_1  602   GTTGACAAGCTGGATTTTCTTTTGCCAAAAGAAAACCCTGCCTCCTTCTCGTTTCTCACC  661
             ||||||||||||||||||||||||||||||||||||||||||||||||| ||| ||||||
Seq_2  1320  GTTGACAAGCTGGATTTTCTTTTGCCAAAAGAAAACCCTGCCTCCTTCTAGTTCCTCACC  1379

Seq_1  662   CGTGGTCCTTTTCTTTCTATGCTTTGCGGCTCTGGCCCTCTCTTGAATGTCCCGCCCAGC  721
             ||||||||||||||||||||||||||||||||||||||||||||||||||||||||||||
Seq_2  1380  CGTGGTCCTTTTCTTTCTATGCTTTGCGGCTCTGGCCCTCTCTTGAATGTCCCGCCCAGC  1439

Seq_1  722   TATTTCGCCAGAATATCCTTCACCGTTGGTCTCCTCGGCAGCACCGGATATTTTGTGAAG  781
             |||||||||||||||||||||||||| |||||||||||||||||||||||||||||||||
Seq_2  1440  TATTTCGCCAGAATATCCTTCACCGTCGGTCTCCTCGGCAGCACCGGATATTTTGTGAAG  1499

Seq_1  782   AGAGCAGAGAAGGGGAAGGGCCACGAGTAGGAAATGAGAAGAAGTCAGTTTTT-TTCTTC  840
             |||||||||| |||||||||||||||||||||||||||||||||||||||||| ||||||
Seq_2  1500  AGAGCAGAGAGGGGGAAGGGCCACGAGTAGGAAATGAGAAGAAGTCAGTTTTTCTTCTTC  1559

Seq_1  841   AAAGAATTCCCATTGAAGGGTTATATGATAGG-------------TGGATTCAATCCATG  887
             |||||||||||||  |||||||||||||||||             |||||||||||||||
Seq_2  1560  AAAGAATTCCCATCTAAGGGTTATATGATAGGGTTATATGATAGGTGGATTCAATCCATG  1619

Seq_1  888   TGGCTGGCCATGTCGACAGATCAGACGCACACAAACAATATTGGGACATGAGACGTACAA  947
             ||||||||||  ||||||||||||||||||||||||||||||||||||||||||||| ||
Seq_2  1620  TGGCTGGCCACATCGACAGATCAGACGCACACAAACAATATTGGGACATGAGACGTATAA  1679

Seq_1  948   ATTATTAAACTATTATGACTCAATCGCGCATTTTGAAAGTATTAGAACTAATTTGACACA  1007
             ||||||||||| ||||||||||||| |||||||||||| |||||||||||||||||||||
Seq_2  1680  ATTATTAAACTGTTATGACTCAATCACGCATTTTGAAAATATTAGAACTAATTTGACACA  1739

Seq_1  1008  TTTAAAAAAATTAGAACATACATTGTATTTTTATTTTTTTATTATGGATCGGAGGAAGTA  1067
             ||||||||||||||||||||||||||||||||||||||||||||||||||||||||||||
Seq_2  1740  TTTAAAAAAATTAGAACATACATTGTATTTTTATTTTTTTATTATGGATCGGAGGAAGTA  1799

Seq_1  1068  TGTTCTAC-CATAGAGACACATTATCCATTTGCCACTTACAGGTATACTGATTCAAGCAG  1126
             |||||||| |||||||||||||||| |||||||||||||||||||||||||||||| |||
Seq_2  1800  TGTTCTACTCATAGAGACACATTATTCATTTGCCACTTACAGGTATACTGATTCAAACAG  1859

Seq_1  1127  TGTAGTATACGAAACCGTCCATATCACTTTTTGTGGGTTTTATGATTTTGTGGGTTTTAT  1186
             ||||||||||||||||||||||||||||||||||||||||||||||
Seq_2  1860  TGTAGTATACGAAACCGTCCATATCACTTTTTGTGGGTTTTATGAT--------------  1905

Seq_1  1187  GATCTATATCAGCATGAAAATTATTATTCCGTATCAGAGCACGCCAGAATTTTGTTGACT  1246
                |||||||||||||||||||||||||||||||||||||||||||||||||||||||||
Seq_2  1906  ---CTATATCAGCATGAAAATTATTATTCCGTATCAGAGCACGCCAGAATTTTGTTGACT  1962

Seq_1  1247  ATCGTTTGGTGCTGAATTTTTTACCGAGGATAGATCTTGAGTTCTTGACAAACCAAACAT  1306
```

```
                ||||||||||||||||||||||||||||||||||||||||||||||||||||||||||||
Seq_2  1963  ATCGTTTGGTGCTGAATTTTTACCGAGGATAGATCTTGAGTTCTTGACAAACCAAACAT  2022

Seq_1  1307  ACCCGAAGTCGTTCCTGGGCACTCTTCCTGGGAGAAAAATCCTCTATGACCTGGGTCGTT  1366
             ||||||||||||||||||||||||||||||||
Seq_2  2023  ACCCGAAGTCGTTCCTGGGCACTCTTCCTGGG----------------------------  2054

Seq_1  1367  CTCCCTCCATGGCTAGACCACACTGAACGATCGACACCTGGCAAACCGAATCTTGAAGCA  1426

Seq_2  2055  ------------------------------------------------------------  2054

Seq_1  1427  ATCCTGATTCTCTTCCTCGTTGAATCCTCCTTCCCCTTTCGTCCAAGCACGCAGGTAAGC  1486

Seq_2  2055  ------------------------------------------------------------  2054

Seq_1  1487  AGGTTCCCATTCCTTTCTCCAAGCGCCGGCCCTAAGCCTTGGATCCCCAGCTACCTCCTC  1546

Seq_2  2055  ------------------------------------------------------------  2054

Seq_1  1547  GACGCTGCCGCTTCGTCTCCGCCGATCTTCCCACCGGCGACAACCATGCTCCTAGGCGCC  1606

Seq_2  2055  ------------------------------------------------------------  2054

Seq_1  1607  ATGGCTGCTCATCCCTGCCGGCACAACTCCCCTGCTCGCTGCGTCGCAGGACCTTCGTCG  1666

Seq_2  2055  ------------------------------------------------------------  2054

Seq_1  1667  CTCCGTCTCCGCTACTCCCACCCGGTCCAGACCACGCTCCCCGGCGTCCAGCAAACGTAT  1726

Seq_2  2055  ------------------------------------------------------------  2054

Seq_1  1727  CGCAGGGGAGTTGTGCCGGCAGGGATGAACAGACATGGCGCCGAGGAGCATGGTTGTCGC  1786

Seq_2  2055  ------------------------------------------------------------  2054

Seq_1  1787  CAGCGGGAAGATCGGCGGAGACGAAGCGGCAGTGTCGAGGAGGTAGCTGGGGATCCAAGG  1846

Seq_2  2055  ------------------------------------------------------------  2054

Seq_1  1847  CTTAGGGCCGGCGCTTGGAGAAAGGAACGGGAACCTGCTTACCTGCGTGCTTGGACGAAA  1906

Seq_2  2055  ------------------------------------------------------------  2054

Seq_1  1907  GGGGAAGGAGGATTCAGCGAGGAAGAGAGTCAGGATTGCTTCGAGATTCGGTTTGCCAGG  1966

Seq_2  2055  ------------------------------------------------------------  2054

Seq_1  1967  TGTCGATCGTTCAGTGTGGTCCAGCCATGGAGGGAGAACGACCCAGGTCGTAGAGGATTT  2026

Seq_2  2055  ------------------------------------------------------------  2054

Seq_1  2027  TTCTCCCTCTTCCTGGGCCACCCTCCACATCGAGCAATTCTGCTCTACACTGCATCCTCC  2086
                              |||||||||||||||||||||||||||||||||||||||||||
Seq_2  2055  -----------------CCACCCTCCACATCGAGCAATTCTGCTCTACACTGCATCCTCC  2097

Seq_1  2087  TCCGCCGCGCTCCGACACCTCAGCCTCCTCCCCCTCGCCGGCGCCGCCATCCACCATCCG  2146
```

```
               ||||||||||||||||||||||||||||||||||||||||||||||||||||||||||||
Seq_2  2098  TCCGCCGCGCTCCGACACCTCAGCCTCCTCCCCCTCGCCGGCGCCGCCATCCACCATCCG  2157

Seq_1  2147  CGCAACTCGCGCCACCGCCGTCTAACACCCACTCGATCTGGTGTATTAGTTCCTCCCAAG  2206
             |||||||||||||||||||||||||||| |||||||||||||||||||||||||||||||
Seq_2  2158  CGCAACTCGCGCCACCGCCGTCTAACACTCACTCGATCTGGTGTATTAGTTCCTCCCAAG  2217

Seq_1  2207  ACAGCGCACCCCGGGCGGCG  2226
             ||||||||||||||||||||
Seq_2  2218  ACAGCGCACCCCGGGCGGCG  2237
```

BdindelWSU_4, DOWNSTREAM

>Bradi1g63490

AGATAAACGGTAATCCAAACTATGTTTTTCATGTAAGTGATGAATGTAAGTTTTGATTTTCACTATGTTTATGTAGTTCTTGAGCTTGT
GGGAGTACAGTTAGCTATTAGTTGCAGAGATTGCTCTACCTTTTGATAATACCAAATAAGCAGAGGTGTTTTTAAAAGTTGTGAACTT
GTGATTATGCTATGGTTGTTGACATATTTTCTTTAAGGTTGCATGTTCCTGATTCTTGTATGTTGTAATTTGAGTAAATCACCTTTGCAC
CACCAACATTTTGAACCAATAATGCACACACGCTTTCCATTTCCAGTCCGGCAGCAACAACAATGATGAAAAGAAAGTGTCACTGCCA
ATCATAACAGAAGAATTTGAAGGCCCAACCTTAAAGTGTTATATAGTCACAGGATTCATCTATCCCATTAATATGGCACCACTACTATC
TATAGTCATATATCCAACTGAAAAAATTGTTAACATTTTGCCAATTGATAATTAGCCTTGGAATATTGGTAGCTCATACTTTGTACTATG
ACAACATTGATAATACGCCCTTCTTTTGAAACCCAAACATACCATATGATATAATCCCCGACACTTATTATGGATCGGAGGGAGTACCA
TATACTCCCTCCATTTCACAAAGGATGGCGTATTTCGTTTCGTTAAGACAAGCCTTTGACCAAGAATTACTCTATTAATATGTAAGTTAT
ATGATACGAAATCATGATCATTAGCAAGAACTTTTAAAGATGAATCCATTGATATAAATTTCATGTATGAAAAAATACATATCAATAG
AGTTTTCATTGGTCAAAGCCTTGTCTTAACAAAACCAAATACGCCAACCTTTGTGAAATGGAGGGAGTAATATTTGTTGATTATTGCTC
ATTCATATAACCTCAACAACCAGTTGATTCTGTAAAATCACCAAAAACTGTCAAGTGCTCAATATATGCTCTTGTAAAAAAACATACAA
TCAGAGGAAGTTAACACATATGGCAATCTACAAGGAAAATCCAACACATTATACAGTTTTAATAAACAAAAGGATGAAACAGATGTC
CCATGTTCTGATATACACAGAGCCTTTTCTTTTTCTTTGACATCTGATATACACAGAGCTTAACAGACTAAGGTACATACATCAGAGGC
ACAATACCTACATGGTAACAACACCATAGTTAGAAGAACATACTAAAAGTACAAGCCAGTACCTGCATTCTTGAGTTTCTGACCGAAA
ACCCAGTTCCTTAACGACCGAAACTAATATGAGTACACAACCCTATTACCCTACAAATCTTAAGGCCTGAAGCAGTCTAGGGATCCTG
TGATTGCTCGTCTCCCCTTCTTCCAGTGCTTCAGAATCTGCAGAGCCAAAGGAAAATATTGCTTGATAGCTTGCCATAAACTTAACTGA
AAGCAAAAGGGGTAATGATTATATGTATGAAGTATATTTATAACTCGGTTCTAGTGCAGACCATGTGGATGTGAGTTGCTTTCAAGA
AGTGGTAGGTCCTCAGCACAAAAAAAAAATCATTTGGTTGGTCTAACAACCTTCATGTGCTTAACACTGTATAGTACTAATATATGGA
GTATATGGAGGCAGAGTCCCATGTGAACCCTCTATTTCATGCATGTGTTTCACTGGAATAATATGTGCTGATTTTTCAGAAGAAACAC
CAAATGTTTTTATCATTTTTTTAGTAGAGAATAAACCTAATGAGCAGTGATGTTTGTTTTTTCAGAAAGAGGGAATGCTCCAGACTCTG
CACACAAACATAATGAGCAGTGATGTAGTGTATTCTATATATGCTTCATTGTTTACCGATTTTTTTTTTCAAGTGATGAAATACACAAC
ACAATACGTCAACGCGCAAAGGCAGTTGCGATGTTAGGTAGGAGCACGAAAGCCGCACGAAAGAGAACGAATAGTTTTTTTTAGGG
GAAGCTGGTCAAGGACTCAAGGTCTTCATTTTAATTTTCTGGAAAAAGACTCGAGGTCTTGATACAGTTCGAAAAATAGAGCACGCA
ATTAATTGTTATACAAAAACAAATGAAGAAATTAATTAGTACATGGCCGCGGTTCTTCTCACGCAAAATCTTCTTCGAGGCTGTGCAA
GCAGATCCAAACAAGGATAGAAAGCTTAAACTGCCAAGCAAAGAATCACGCTACAATGCCTAGATTAATGATACATACATGAGACAC
AGGAGAGGGGCGAAAGCCAGGCCAGGCGCCGTCGTCAAGCTCCCCACACGGTGATCCCCTGCATCATGACCTTGAACTCGCCGAAG
TCGACCTGGCCGTCGCGGTCGCGGTCGACGTTGCAGATCATCTCCTGCACCGCCGCCAGGTTCCGCGCCTCCGCCAGGCCGAGCTTCT
TGAGCACGGCCTGCAGCTCGGCCGCCGAGATGAACCCGTCGCCGTTCTCGTCGAACACCCGGAACGCCTCCTTCATGTCCCCTTCTTC
GTCCTCCTTCCGGAGCTCCTCCTCCTCGGCGATGGGGCCGAAGAGCGCGTCGCCGAGGGCGCGGTGGAGGCTCTCGAAGTCCTCGA
ACCCGAGCCCCGCGGCGCCGGCGGGGATGTAGGCGCCCACGGTGGCCTCCAGGCTAGGGCGGTCGGCGCCCAGGCCGAGCGTGTC
CAGGGCCGACGCCATCTCGTCCAGGGTGATCTCGCCGTCGCCGTTGCGGTCGAAGAGGTCGAACACGCGGCGCAGGCGCAG

>BdiBd21-3.1G0856600

AGATAAACGGTAATCCAAACTATGTTTTTCATGTAAGTGATGAATGTAAGTTTTGATTTTCACTATGTTTATGTAGTTCTTGAGCTTGT
GGGAGTACAGTTAGCTATTAGTTGCAGAGATTGCTCTACCTTTTGATAATACCAAATAAGCAGAGGTGTTTTTAAAAGTTGTGAACTT
GTGATTATGCTATGGTTGTTGACATATTTTCTTTAAGGTTGCATGTTCCTGATTCTTGTATGTTGTAATTTGAGTAAATCACCTTTGCAC

CACCAACATTTTGAACCAATAATGCACACACGCTTTCCATTTCCAGTCCGGCAGCAACAACAATGATGAAAAGAAAGTGTCACTGCCA
ATCATAACAGAAGAATTTGAAGGCCCAACCTTAAAGTGTTATATAGTCACAGGATTCATCTATCCCATTAATATGGCACCACTACTATC
TATAGTCATATATCCAACTGAAAAAATTGTTAACATTTTGCCAATTGATAATTAGCCTTGGAATATTGGTAGCTCATACTTTGTACTATG
ACAACATTGATAATACGCCCTTCTTTTGAAACCCAAACATACCATATGATATAATCCCCGACACTTATTATGGATCGGAGGGAGTACCA
TATAATATTTGTTGATTATTGCTAATTCATATAACCTCAACAACCAGTTGATTCTGTAAAATCACCAAAAACTGTCAAGTGCTCAATATA
TGCTCTTGTAAAAAAACATACAATCAGAGGAAGTTAACACATATGGCAATCTACAAGGAAAATCCAACACATTATACAGTTTTAATAA
ACAAAAGGATGAAACAGACGTCCCATGTTCTGATATACACAGAGCTTTTTCTTTTTCTTTGACATCTGATATACACAGAGCTTAACAGA
CTAAGGTACATACATCAGAGGCACAATACCTACATGGTAACAACACCATAGTTAGAAGAACATACTAAAAGTACAAGCCAGTACCTG
CATTCTTGAGTTTCTGACCGAAAACCCAGTTCCTTAACGACCGAAACTAATATGAGTACACAACCCTATTACCCTACAAATCTTAAGGC
CTGAAGCAGTCTAGGGATCCTGTGATTGCTCGTCTCCCCTTCTTCCAGTGCTTCAGAATCTGCAGAGCCAAAGGAAAATATTGCTTGA
TAGCTTGCCATAAACTTAACTGAAAGCAAAAGGGGTAATGATTATATGTATGAAGTATATTTATAACTCGGTTCTAGTGCAGACCATG
TGGATGTGAGTTGCTTTCAAGAAGTGGTAGGTCCTCAGCACAAAAAAAAAAATCATTTGGTTGGTCTAACAACCTTCATGTGCTTAACA
CTGTATAGTACTAATATATGGAGTATATGGAGGCAGAGTCCCATGTGAACCCTCTATTTCATGCATGTGTTTCACTGGAATAATATGT
GCTGATTTTTCAGAAGAAACACCAAATGTTTTTATCATTTTTTTAGTAGAGAATAAACCTAATGAGCAGTGATGTTTGTTTTTTCAGAA
AGAGGGAATGCTCCAGACTCTGCACACAAACATAATGAGCAGTGATGTAGTGTATTCTATATATGCTTCATTGTTTACCGATTTTTTTT
TCAAGTGATGAAATACACAACACAATACGTCAACGCGCAAAGGCAGTTGCGATGTTAGGTAGGAGCACGAAAGCCGCACGAAAGAG
AACGAATAGTTTTTTTTAGGGGAAGCTGGTCAAGGACTCAAGGTCTTCATTTTAATTTTCTGGAAAAAGACTCGAGGTCTTGATACAG
TTCGAAAAATAGAGCACGCAATTAATTGTTATACAAAAACAAATGAAGAAATTAATTAGTACATGGCCGCGGTTCTTCTCACGCAAAA
TCTTCTTCGAGGCTGTGCAAGCAGATCCAAACAAGGATAGAAAGCTTAAACTGCCAAGCAAATAATCACGCTACAATGCCTAGATTA
ATGATACATACATGAGACACAGGAGAGGGGCGAAAGCCAGGCCAGGCGCCGTCGTCAAGCTCCCCACACGGTAATCCCCTGCATCA
TGACCTTGAACTCGCCGAAGTCGACCTGGCCGTCGCGGTCGCGGTCGACGTTGCAGATCATCTCCTGCACCGCCGCCAGGTTCCGCG
CCTCCGCCAGGCCGAGCTTCTTGAGCACGGCCTGCAGCTCGGCCGCCGAGATGAACCCGTCGCCGTTCTCGTCGAACACCCGGAACG
CCTCCTTCATGTCCCCTTCTTCGTCCTCCTTCCGGAGCTCCTCCTCCTCGGCGATGGGGCCGAAGAGCGCGTCGCCGAGGGCGCGGTG
GAGGCTCTCGAAGTCCTCGAACCCGAGCCCCGCGGCGCCGGCGGGGATGTAGGCGCCCACGGTGGCCTCCAGGCTAGGGCGGTCG
GCGCCCAGGCCGAGCGTGTCCAGGGCCGACGCCATCTCGTCCAGGGTGATCTCGCCGTCGCCGTTGCGGTCGAAGAGGTCGAACAC
GCGGCGCAGGCGCAGCGCGTTCAGGCTGCCGTTGCGGAGGCGGAACGACGGCGAGGGCTTCTTGGACAAGGACGGCTTTGGGGG
AGCGGCGGCGTCCATGGCTTGAGTTCGCGGCGTTTATGTCTCTCCTTTTCTGTCTGGGTTACTAGGCTTTTGAGAACGCGGCGAATAT
TTTTGGAGTGCTTGAAACGGTGGCGAGGCAAGGTTTTATAGATGAGGCCAGTTCTGTAGAAAATGGTTTGTAAAGGTGGGGACGAA
CGTGATGGCGAGTCTCCGGGACTCGGAACGGATCAAACTGTATCTAAAAGAGAAAATGACAGTATTAGCAAAACTCAATCCATTCAA
AGACTGCTAGACCTATTAGTACGAGCATGTCAACACTAATCCAACATAAGACATGACGATATCAAATTAGTTGAAATCTTTGGAAACC
CACAATTTTAACTAGTAGTACTAGTTATATACCCTAACATTGTCAACTCTTCTATTTCTTTTTTCTCATTGGCATTGTCGTGGCTCATTTC
GATTGATTTTAAAAATTGCTTTCACTTTTCCACAATCACCGTAAACTTGTGTCAACATCTTCAAAATCTCATTAGCCACATGCTAGCGTT
ATGTGTCATCTAATCTTCCAAAGAGCATGACATGTAGACGGCACCATCGGCCTCCTTTCTTCTTCCTCGTCTTCTCTGCTCCCGCCTCCT
CTTCCTCACACATGAAATTCCCCATGGAACGAATACACCCCCCCCCCCCCCACCACACACACACACACCATCCACCACCACCACCACAC
ACACGCACCCAATCTCCTTGTCCGACGAGTGCTCGGGCGAGATGGCGGCAGGGTTCCCTCGTCCGATGCGGCCTCCTCCTATTGGAG
ATGTGCCTCAGTCGATGTAGAGGATGAACGCGCATGTCTCCTTTTCAAAGAAGAAACACTATTGGCGATGCGTTCAGTAAAACAATTT
CAAATGTTACCAATCTATTGGGTGAGGGGAGGTGCAATGATAAGAGATTGGAGGAAGATTTAACTAATGTGCAGTGAATTTCTTGG
GAATGTGAAGAGAAATACCAAGTAGATGTAATTTGTCCCCCTAGTTTCCACTTGTAATTTGTGTTTATGTGCCAAAAAAA

Alignment of Sequence_1: [Untitled Sequence #1] with Sequence_2: [Sequence Window #2]

Similarity : 2475/3599 (68.77 %)

```
Seq_1  1      AGATAAACGGTAATCCAAACTATGTTTTTTCATGTAAGTGATGAATGTAAGTTTTGATTT  60
              ||||||||||||||||||||||||||||||||||||||||||||||||||||||||||||
Seq_2  1      AGATAAACGGTAATCCAAACTATGTTTTTTCATGTAAGTGATGAATGTAAGTTTTGATTT  60


Seq_1  61     TCACTATGTTTATGTAGTTCTTGAGCTTGTGGGAGTACAGTTAGCTATTAGTTGCAGAGA  120
              ||||||||||||||||||||||||||||||||||||||||||||||||||||||||||||
Seq_2  61     TCACTATGTTTATGTAGTTCTTGAGCTTGTGGGAGTACAGTTAGCTATTAGTTGCAGAGA  120


Seq_1  121    TTGCTCTACCTTTTGATAATACCAAATAAGCAGAGGTGTTTTTAAAAGTTGTGAACTTGT  180
```

```
               ||||||||||||||||||||||||||||||||||||||||||||||||||||||||||||
Seq_2  121     TTGCTCTACCTTTTGATAATACCAAATAAGCAGAGGTGTTTTTAAAAGTTGTGAACTTGT  180

Seq_1  181     GATTATGCTATGGTTGTTGACATATTTTCTTTAAGGTTGCATGTTCCTGATTCTTGTATG  240
               ||||||||||||||||||||||||||||||||||||||||||||||||||||||||||||
Seq_2  181     GATTATGCTATGGTTGTTGACATATTTTCTTTAAGGTTGCATGTTCCTGATTCTTGTATG  240

Seq_1  241     TTGTAATTTGAGTAAATCACCTTTGCACCACCAACATTTTGAACCAATAATGCACACACG  300
               ||||||||||||||||||||||||||||||||||||||||||||||||||||||||||||
Seq_2  241     TTGTAATTTGAGTAAATCACCTTTGCACCACCAACATTTTGAACCAATAATGCACACACG  300

Seq_1  301     CTTTCCATTTCCAGTCCGGCAGCAACAACAATGATGAAAAGAAAGTGTCACTGCCAATCA  360
               ||||||||||||||||||||||||||||||||||||||||||||||||||||||||||||
Seq_2  301     CTTTCCATTTCCAGTCCGGCAGCAACAACAATGATGAAAAGAAAGTGTCACTGCCAATCA  360

Seq_1  361     TAACAGAAGAATTTGAAGGCCCAACCTTAAAGTGTTATATAGTCACAGGATTCATCTATC  420
               ||||||||||||||||||||||||||||||||||||||||||||||||||||||||||||
Seq_2  361     TAACAGAAGAATTTGAAGGCCCAACCTTAAAGTGTTATATAGTCACAGGATTCATCTATC  420

Seq_1  421     CCATTAATATGGCACCACTACTATCTATAGTCATATATCCAACTGAAAAAATTGTTAACA  480
               ||||||||||||||||||||||||||||||||||||||||||||||||||||||||||||
Seq_2  421     CCATTAATATGGCACCACTACTATCTATAGTCATATATCCAACTGAAAAAATTGTTAACA  480

Seq_1  481     TTTTGCCAATTGATAATTAGCCTTGGAATATTGGTAGCTCATACTTTGTACTATGACAAC  540
               ||||||||||||||||||||||||||||||||||||||||||||||||||||||||||||
Seq_2  481     TTTTGCCAATTGATAATTAGCCTTGGAATATTGGTAGCTCATACTTTGTACTATGACAAC  540

Seq_1  541     ATTGATAATACGCCCTTCTTTTGAAACCCAAACATACCATATGATATAATCCCCGACACT  600
               ||||||||||||||||||||||||||||||||||||||||||||||||||||||||||||
Seq_2  541     ATTGATAATACGCCCTTCTTTTGAAACCCAAACATACCATATGATATAATCCCCGACACT  600

Seq_1  601     TATTATGGATCGGAGGGAGTACCATATACTCCCTCCATTTCACAAAGGATGGCGTATTTC  660
               ||||||||||||||||||||||||||||
Seq_2  601     TATTATGGATCGGAGGGAGTACCATATA--------------------------------  628

Seq_1  661     GTTTCGTTAAGACAAGCCTTTGACCAAGAATTACTCTATTAATATGTAAGTTATATGATA  720

Seq_2  629     ------------------------------------------------------------  628

Seq_1  721     CGAAATCATGATCATTAGCAAGAACTTTTAAAGATGAATCCATTGATATAAATTTCATGT  780

Seq_2  629     ------------------------------------------------------------  628

Seq_1  781     ATGAAAAAATACATATCAATAGAGTTTTCATTGGTCAAAGCCTTGTCTTAACAAAACCAA  840

Seq_2  629     ------------------------------------------------------------  628

Seq_1  841     ATACGCCAACCTTTGTGAAATGGAGGGAGTAATATTTGTTGATTATTGCTCATTCATATA  900
                                               ||||||||||||||||||| |||||||||
Seq_2  629     -------------------------------ATATTTGTTGATTATTGCTAATTCATATA  657

Seq_1  901     ACCTCAACAACCAGTTGATTCTGTAAAATCACCAAAAACTGTCAAGTGCTCAATATATGC  960
               ||||||||||||||||||||||||||||||||||||||||||||||||||||||||||||
Seq_2  658     ACCTCAACAACCAGTTGATTCTGTAAAATCACCAAAAACTGTCAAGTGCTCAATATATGC  717

Seq_1  961     TCTTGTAAAAAAACATACAATCAGAGGAAGTTAACACATATGGCAATCTACAAGGAAAAT  1020
```

```
               ||||||||||||||||||||||||||||||||||||||||||||||||||||||||||||
Seq_2  718     TCTTGTAAAAAAACATACAATCAGAGGAAGTTAACACATATGGCAATCTACAAGGAAAAT  777

Seq_1  1021    CCAACACATTATACAGTTTTAATAAACAAAAGGATGAAACAGATGTCCCATGTTCTGATA  1080
               ||||||||||||||||||||||||||||||||||||||||||| ||||||||||||||||
Seq_2  778     CCAACACATTATACAGTTTTAATAAACAAAAGGATGAAACAGACGTCCCATGTTCTGATA  837

Seq_1  1081    TACACAGAGCCTTTTCTTTTTCTTTGACATCTGATATACACAGAGCTTAACAGACTAAGG  1140
               |||||||||| |||||||||||||||||||||||||||||||||||||||||||||||||
Seq_2  838     TACACAGAGCTTTTTCTTTTTCTTTGACATCTGATATACACAGAGCTTAACAGACTAAGG  897

Seq_1  1141    TACATACATCAGAGGCACAATACCTACATGGTAACAACACCATAGTTAGAAGAACATACT  1200
               ||||||||||||||||||||||||||||||||||||||||||||||||||||||||||||
Seq_2  898     TACATACATCAGAGGCACAATACCTACATGGTAACAACACCATAGTTAGAAGAACATACT  957

Seq_1  1201    AAAAGTACAAGCCAGTACCTGCATTCTTGAGTTTCTGACCGAAAACCCAGTTCCTTAACG  1260
               ||||||||||||||||||||||||||||||||||||||||||||||||||||||||||||
Seq_2  958     AAAAGTACAAGCCAGTACCTGCATTCTTGAGTTTCTGACCGAAAACCCAGTTCCTTAACG  1017

Seq_1  1261    ACCGAAACTAATATGAGTACACAACCCTATTACCCTACAAATCTTAAGGCCTGAAGCAGT  1320
               ||||||||||||||||||||||||||||||||||||||||||||||||||||||||||||
Seq_2  1018    ACCGAAACTAATATGAGTACACAACCCTATTACCCTACAAATCTTAAGGCCTGAAGCAGT  1077

Seq_1  1321    CTAGGGATCCTGTGATTGCTCGTCTCCCCTTCTTCCAGTGCTTCAGAATCTGCAGAGCCA  1380
               ||||||||||||||||||||||||||||||||||||||||||||||||||||||||||||
Seq_2  1078    CTAGGGATCCTGTGATTGCTCGTCTCCCCTTCTTCCAGTGCTTCAGAATCTGCAGAGCCA  1137

Seq_1  1381    AAGGAAAATATTGCTTGATAGCTTGCCATAAACTTAACTGAAAGCAAAAGGGGTAATGAT  1440
               ||||||||||||||||||||||||||||||||||||||||||||||||||||||||||||
Seq_2  1138    AAGGAAAATATTGCTTGATAGCTTGCCATAAACTTAACTGAAAGCAAAAGGGGTAATGAT  1197

Seq_1  1441    TATATGTATGAAGTATATTTATAACTCGGTTCTAGTGCAGACCATGTGGATGTGAGTTGC  1500
               ||||||||||||||||||||||||||||||||||||||||||||||||||||||||||||
Seq_2  1198    TATATGTATGAAGTATATTTATAACTCGGTTCTAGTGCAGACCATGTGGATGTGAGTTGC  1257

Seq_1  1501    TTTCAAGAAGTGGTAGGTCCTCAGCACAAAAAAAAAATCATTTGGTTGGTCTAACAACCT  1560
               ||||||||||||||||||||||||||||||||||||||||||||||||||||||||||||
Seq_2  1258    TTTCAAGAAGTGGTAGGTCCTCAGCACAAAAAAAAAATCATTTGGTTGGTCTAACAACCT  1317

Seq_1  1561    TCATGTGCTTAACACTGTATAGTACTAATATATGGAGTATATGGAGGCAGAGTCCCATGT  1620
               ||||||||||||||||||||||||||||||||||||||||||||||||||||||||||||
Seq_2  1318    TCATGTGCTTAACACTGTATAGTACTAATATATGGAGTATATGGAGGCAGAGTCCCATGT  1377

Seq_1  1621    GAACCCTCTATTTCATGCATGTGTTTCACTGGAATAATATGTGCTGATTTTTCAGAAGAA  1680
               ||||||||||||||||||||||||||||||||||||||||||||||||||||||||||||
Seq_2  1378    GAACCCTCTATTTCATGCATGTGTTTCACTGGAATAATATGTGCTGATTTTTCAGAAGAA  1437

Seq_1  1681    ACACCAAATGTTTTTATCATTTTTTTAGTAGAGAATAAACCTAATGAGCAGTGATGTTTG  1740
               ||||||||||||||||||||||||||||||||||||||||||||||||||||||||||||
Seq_2  1438    ACACCAAATGTTTTTATCATTTTTTTAGTAGAGAATAAACCTAATGAGCAGTGATGTTTG  1497

Seq_1  1741    TTTTTTCAGAAAGAGGGAATGCTCCAGACTCTGCACACAAACATAATGAGCAGTGATGTA  1800
               ||||||||||||||||||||||||||||||||||||||||||||||||||||||||||||
Seq_2  1498    TTTTTTCAGAAAGAGGGAATGCTCCAGACTCTGCACACAAACATAATGAGCAGTGATGTA  1557

Seq_1  1801    GTGTATTCTATATATGCTTCATTGTTTACCGATTTTTTTTTTCAAGTGATGAAATACACA  1860
```

```
              |||||||||||||||||||||||||||||||||||||||| |||||||||||||||||||
Seq_2  1558   GTGTATTCTATATATGCTTCATTGTTTACCGATTTTTTTT-CAAGTGATGAAATACACA  1616

Seq_1  1861   ACACAATACGTCAACGCGCAAAGGCAGTTGCGATGTTAGGTAGGAGCACGAAAGCCGCAC  1920
              ||||||||||||||||||||||||||||||||||||||||||||||||||||||||||||
Seq_2  1617   ACACAATACGTCAACGCGCAAAGGCAGTTGCGATGTTAGGTAGGAGCACGAAAGCCGCAC  1676

Seq_1  1921   GAAAGAGAACGAATAGTTTTTTTTAGGGGAAGCTGGTCAAGGACTCAAGGTCTTCATTTT  1980
              ||||||||||||||||||||||||||||||||||||||||||||||||||||||||||||
Seq_2  1677   GAAAGAGAACGAATAGTTTTTTTTAGGGGAAGCTGGTCAAGGACTCAAGGTCTTCATTTT  1736

Seq_1  1981   AATTTTCTGGAAAAAGACTCGAGGTCTTGATACAGTTCGAAAAATAGAGCACGCAATTAA  2040
              ||||||||||||||||||||||||||||||||||||||||||||||||||||||||||||
Seq_2  1737   AATTTTCTGGAAAAAGACTCGAGGTCTTGATACAGTTCGAAAAATAGAGCACGCAATTAA  1796

Seq_1  2041   TTGTTATACAAAAACAAATGAAGAAATTAATTAGTACATGGCCGCGGTTCTTCTCACGCA  2100
              ||||||||||||||||||||||||||||||||||||||||||||||||||||||||||||
Seq_2  1797   TTGTTATACAAAAACAAATGAAGAAATTAATTAGTACATGGCCGCGGTTCTTCTCACGCA  1856

Seq_1  2101   AAATCTTCTTCGAGGCTGTGCAAGCAGATCCAAACAAGGATAGAAAGCTTAAACTGCCAA  2160
              ||||||||||||||||||||||||||||||||||||||||||||||||||||||||||||
Seq_2  1857   AAATCTTCTTCGAGGCTGTGCAAGCAGATCCAAACAAGGATAGAAAGCTTAAACTGCCAA  1916

Seq_1  2161   GCAAAGAATCACGCTACAATGCCTAGATTAATGATACATACATGAGACACAGGAGAGGGG  2220
              ||||| ||||||||||||||||||||||||||||||||||||||||||||||||||||||
Seq_2  1917   GCAAATAATCACGCTACAATGCCTAGATTAATGATACATACATGAGACACAGGAGAGGGG  1976

Seq_1  2221   CGAAAGCCAGGCCAGGCGCCGTCGTCAAGCTCCCCACACGGTGATCCCCTGCATCATGAC  2280
              |||||||||||||||||||||||||||||||||||||||||| |||||||||||||||||
Seq_2  1977   CGAAAGCCAGGCCAGGCGCCGTCGTCAAGCTCCCCACACGGTAATCCCCTGCATCATGAC  2036

Seq_1  2281   CTTGAACTCGCCGAAGTCGACCTGGCCGTCGCGGTCGCGGTCGACGTTGCAGATCATCTC  2340
              ||||||||||||||||||||||||||||||||||||||||||||||||||||||||||||
Seq_2  2037   CTTGAACTCGCCGAAGTCGACCTGGCCGTCGCGGTCGCGGTCGACGTTGCAGATCATCTC  2096

Seq_1  2341   CTGCACCGCCGCCAGGTTCCGCGCCTCCGCCAGGCCGAGCTTCTTGAGCACGGCCTGCAG  2400
              ||||||||||||||||||||||||||||||||||||||||||||||||||||||||||||
Seq_2  2097   CTGCACCGCCGCCAGGTTCCGCGCCTCCGCCAGGCCGAGCTTCTTGAGCACGGCCTGCAG  2156

Seq_1  2401   CTCGGCCGCCGAGATGAACCCGTCGCCGTTCTCGTCGAACACCCGGAACGCCTCCTTCAT  2460
              ||||||||||||||||||||||||||||||||||||||||||||||||||||||||||||
Seq_2  2157   CTCGGCCGCCGAGATGAACCCGTCGCCGTTCTCGTCGAACACCCGGAACGCCTCCTTCAT  2216

Seq_1  2461   GTCCCCTTCTTCGTCCTCCTTCCGGAGCTCCTCCTCCTCGGCGATGGGGCCGAAGAGCGC  2520
              ||||||||||||||||||||||||||||||||||||||||||||||||||||||||||||
Seq_2  2217   GTCCCCTTCTTCGTCCTCCTTCCGGAGCTCCTCCTCCTCGGCGATGGGGCCGAAGAGCGC  2276

Seq_1  2521   GTCGCCGAGGGCGCGGTGGAGGCTCTCGAAGTCCTCGAACCCGAGCCCCGCGGCGCCGGC  2580
              ||||||||||||||||||||||||||||||||||||||||||||||||||||||||||||
Seq_2  2277   GTCGCCGAGGGCGCGGTGGAGGCTCTCGAAGTCCTCGAACCCGAGCCCCGCGGCGCCGGC  2336

Seq_1  2581   GGGGATGTAGGCGCCCACGGTGGCCTCCAGGCTAGGGCGGTCGGCGCCCAGGCCGAGCGT  2640
              ||||||||||||||||||||||||||||||||||||||||||||||||||||||||||||
Seq_2  2337   GGGGATGTAGGCGCCCACGGTGGCCTCCAGGCTAGGGCGGTCGGCGCCCAGGCCGAGCGT  2396

Seq_1  2641   GTCCAGGGCCGACGCCATCTCGTCCAGGGTGATCTCGCCGTCGCCGTTGCGGTCGAAGAG  2700
```

```
          ||||||||||||||||||||||||||||||||||||||||||||||||||||||||||||
Seq_2  2397  GTCCAGGGCCGACGCCATCTCGTCCAGGGTGATCTCGCCGTCGCCGTTGCGGTCGAAGAG  2456

Seq_1  2701  GTCGAACACGCGGCGCAGGCGCAG------------------------------------  2724
          ||||||||||||||||||||||||
Seq_2  2457  GTCGAACACGCGGCGCAGGCGCAGCGCGTTCAGGCTGCCGTTGCGGAGGCGGAACGACGG  2516

Seq_1  2725  ------------------------------------------------------------  2724

Seq_2  2517  CGAGGGCTTCTTGGACAAGGACGGCTTTGGGGGAGCGGCGGCGTCCATGGCTTGAGTTCG  2576

Seq_1  2725  ------------------------------------------------------------  2724

Seq_2  2577  CGGCGTTTATGTCTCTCCTTTTCTGTCTGGGTTACTAGGCTTTTGAGAACGCGGCGAATA  2636

Seq_1  2725  ------------------------------------------------------------  2724

Seq_2  2637  TTTTTGGAGTGCTTGAAACGGTGGCGAGGCAAGGTTTTATAGATGAGGCCAGTTCTGTAG  2696

Seq_1  2725  ------------------------------------------------------------  2724

Seq_2  2697  AAAATGGTTTGTAAAGGTGGGGACGAACGTGATGGCGAGTCTCCGGGACTCGGAACGGAT  2756

Seq_1  2725  ------------------------------------------------------------  2724

Seq_2  2757  CAAACTGTATCTAAAAGAGAAAATGACAGTATTAGCAAAACTCAATCCATTCAAAGACTG  2816

Seq_1  2725  ------------------------------------------------------------  2724

Seq_2  2817  CTAGACCTATTAGTACGAGCATGTCAACACTAATCCAACATAAGACATGACGATATCAAA  2876

Seq_1  2725  ------------------------------------------------------------  2724

Seq_2  2877  TTAGTTGAAATCTTTGGAAACCCACAATTTTAACTAGTAGTACTAGTTATATACCCTAAC  2936

Seq_1  2725  ------------------------------------------------------------  2724

Seq_2  2937  ATTGTCAACTCTTCTATTTCTTTTTTCTCATTGGCATTGTCGTGGCTCATTTCGATTGAT  2996

Seq_1  2725  ------------------------------------------------------------  2724

Seq_2  2997  TTTAAAAATTGCTTTCACTTTTCCACAATCACCGTAAACTTGTGTCAACATCTTCAAAAT  3056

Seq_1  2725  ------------------------------------------------------------  2724

Seq_2  3057  CTCATTAGCCACATGCTAGCGTTATGTGTCATCTAATCTTCCAAAGAGCATGACATGTAG  3116

Seq_1  2725  ------------------------------------------------------------  2724

Seq_2  3117  ACGGCACCATCGGCCTCCTTTCTTCTTCCTCGTCTTCTCTGCTCCCGCCTCCTCTTCCTC  3176

Seq_1  2725  ------------------------------------------------------------  2724

Seq_2  3177  ACACATGAAATTCCCCATGGAACGAATACACCCCCCCCCCCCCCACCACACACACACACA  3236

Seq_1  2725  ------------------------------------------------------------  2724
```

Seq_2 3237 CCATCCACCACCACCACCACACACACGCACCCAATCTCCTTGTCCGACGAGTGCTCGGGC 3296

Seq_1 2725 -- 2724

Seq_2 3297 GAGATGGCGGCAGGGTTCCCTCGTCCGATGCGGCCTCCTCCTATTGGAGATGTGCCTCAG 3356

Seq_1 2725 -- 2724

Seq_2 3357 TCGATGTAGAGGATGAACGCGCATGTCTCCTTTTCAAAGAAGAAACACTATTGGCGATGC 3416

Seq_1 2725 -- 2724

Seq_2 3417 GTTCAGTAAAACAATTTCAAATGTTACCAATCTATTGGGTGAGGGGAGGTGCAATGATAA 3476

Seq_1 2725 -- 2724

Seq_2 3477 GAGATTGGAGGAAGATTTAACTAATGTGCAGTGAATTTCTTGGGAATGTGAAGAGAAATA 3536

Seq_1 2725 -- 2724

Seq_2 3537 CCAAGTAGATGTAATTTGTCCCCCTAGTTTCCACTTGTAATTTGTGTTTATGTGCCAAAA 3596

Seq_1 2725 --- 2724

Seq_2 3597 AAA 3599

BdindelWSU_5, upstream

>Bradi2g10240

GGGGGGATGGGGATTGGTGGGATTCGCGTCTAGTGACCGTTGGGGGCGGCGCTAGCTGTTTCTGATGCCCGTTGCGTGCAGCAATT
ACTCGCGACAGCTTTCCGGTGAGGCTGGCTCCAACGGCTAGTTTGGGCCGGCGGTGCCGGTGTCACACGCAGACTTGACTGACTGCC
CTGTCTGCTCTGCAGTTGGTCACGTCACGTAGTACTGCTACGCCGTACGTGCGTCTCGGAACATGCGGTGCCAGCAATCATACTGAAA
ATTGGTGCAGATTTGACCTCTTGAGTAAACATCATAGCTAGGACGGTGTACCATGATGTTTATCTTCAGTCGTTGTGGGGCATTACAG
TAAGCGAGGTTTGTCGCCAATATCCGATGCAACATCGCCTATGTGGACGTTTAAAAGAGAAAAACACAAATGCACATATAAGCGTCG
CCCACTTCAATACTCCATCACAGTACACTGTACACCAATGCAGAACACAACTAAAAACGCACCCGTGATTCACCTATGCTCCAAGTTAA
ACGTCGATGCTAACTTGTTTGGAATAGATCCTGAACAAACTAAAAGGCTGGGCTTTCAGCTCCACTCTCAAGCATCCCAGGAGATGGC
TCTAGGTGATTAGTTGACAAGTGACCTCCATTCCTCTTCGTCATATTCTATTAATCATGGTCTGTAAGAACATGCTTCGGCTTTGGATCA
ATCAATTGCTCATTTTCTCCTTGATGCAAAGCCTCCATCAACCACAAGAATGACGACTTTGCCATCACGATTCGTCTTTCTCTATGGTGT
TGAGGATGTTCTTAGTAATATATAAATTTTATGAAGTTCCCATACCAATTGCAAATCATTTCGAGAAAAAATAATTAACCTCACAAAAT
AGTGTGCATAGCAAAGGAGATTATAGGGGAGACAAATATAAGGAGATAGTGAGTTCTTGCCTGTCGAAGGTCAAGATGTGGCACTT
TGATCGAGAATAAGAGAGCTGAAACTTCTATTGGAAACAAGCTAGCAATGGTAGTCATATAAATAACTTGTCAATAAAGATGGATGA
CAAAAGGTTAGATATTATTGTTTAATGTTTAGTCATATGTTTTATTAGCCAAAAGAAGCAACGAACAGAGTTAGACACAACGCAGATC
ATACACTATTCACCGGTGGAGATAAAGGGACGAACGATGAATCACTCACAAAATATAACTGGAGGTGAACCATCAAAGCTATCGGTT
TGGCTTCCCCGTAGGAAACCGAACACCATGAAGGCCGTGGGTCCAAACTCAAACCATAACAATCTATTTTCATGGTATAGATTTGGCA
TTCCATAAAGTCAACGGCAAAGTCTTATCAATGACTCAACAATGTACTTTATCTCGGTCTAAATTGCATATTTATAATTTATTTTTACAA
AAAAACTGTAGTTCTTTAAGTTGTTATATGAATAAAAATCATGCTACACCATCGGTGTTTTCCTTTTGCTACAAAACTATTGTGATATTT
AAATTTATTGTCAGGATACCGCTATATTGCATCATGGAAAAATGAGAAATTGTCCAATCTTAAGACAAACACATAAATATTTCCTTTTA
CAAACTCTTGTCAGGTATAGTCACATTTTGTCCCTTAAATTTTAGGAAATCACCTGTTATAGAGTGAAACACGTCCTTTTGCATACGAT
CGGTTTGTTACGACAAATTACGATTAGTTTTTTCTTACTGTAATTGTTGATAAAAACCCAGCCAACTTATGCTGTCCACTAAATGCTACA
TTCTTTGTCGTAACAAAATCGTACTCCCATCGATGCATTAAAATTCAAGACGCTTGTTAGTGATTGGAGGGATATGTAAATGTTCGCA

GGTATAGCAGTCCTCTTTTCCACGACTTTCAGATATTTTGGAAAATCAGATGGCCAAAAAATGTGTTGGTGGGTGACCCCGTTCCGTA
CTGAAAAGAAACAAACACGGCTCGAACTGCCCCGAATCGACGGCACCGCTTTACTAACCCCGAGTCTACGACCCCATTGGTCTGCCAC
GAACCATCGTCTCTGTCCCGAACGAGCAATCGCCCCTAGGGTTTCTCCAACCACACGCGAGTACGCGACGCGAATCAGGTCGGTGGC
GTCCAGATCCTTCTCCCTCCTTTCCACCCCAAAATCTCCAATCCGGATCTCTGTACTCACCCTCTGATCTGCTCGGCCGCGTCGCAGGTA
CTAGACACCAGCACCAGCGCTGATGGCGGCGGCCCCGGCGGACGCGAAAGCTGAGGCGGCCAAGATGGACCTCCTCGAGGACGAC
GACGAGTTCGAGGAGTTCGAGATCGACCAAGGTATGTCCACGCGCTCTCCGCTGCCCCCCTCTCGTTACTACCATGTTGCTATATTTA
GGCTAGGCTAACGAGATCGATTCGCCGTAGCTTGTTTGCGCCGGGAGGCCGATTTGGGCGACTAGGTTTGGGTTGATTCGGAACCT
GTACCATGTCACACTTCCTTTGAGATTCGTTATGGAAGAGATTTGCGTGAAATTAAGGCAAAGTACAGACACTGGTATCGATTTTACA
GGGCATTAGGTAGGTCGTGACGTCAGGTAGTGCTCTCAGTGACCTGACTTAGTTATGGTCTCCACTGCTGTTCCTGGGTAACTCCCCT
CGCGCTTGTTGTAATATGTAAATAGCTAGCTGGTTTTGATAGATACATGTATATGTCCCGTTTAGTTTAGCATGAAACCGGTGCTTTAA
TAGTGGTGTGTATTTATGAGCGTCCACTTCTTGGCATGATTGCTTCTTTTAGGGAAAAATATTAGGCACATGAAACTATGTTTATCTGC
AGTTGGGCTTGTAGTTGTTTGAGACAATGAGACCCCAAGTGTTGGGATGTAGCTTAAGGAAAACACAAAATATATCTCCTTGAAATT
GCATGTTGGAACTAATTTTCTCCATTGGGAATAACAATCTTCACTTCCTGTTTCTTCACTGTTAACATGAAGAGAACTGCACCATTAGA
ATGACACAAACCCTGAAGCTTGAACATATATTGGAAAATAAACTCATGATTAGCCATCATGGTGCAGCCACACACCTAGCAGCAGAG
CAAATCGAGTGAGTTGCTATAGCTGCTGGCTTGCTCTGACTAGTAGTTGGCTAGGATCTTAAAGTTGCCTGACTTGGTGTGCTATGGC
CCACTGTCAGGACATCGACAAGTTACTCTCTTCCAGTCTAGAGGAGGTGAATTCCAAAAGCAGAGCACCGCATCAAGTCAAGGATAG
AAGAGTGTGTCAGAAGCAGAAAACCTTAGCCCAGGAGATTTATGTTAGCTATGAATATTTTTGCCACTTAAAGTATTCTTACATGTGT
TATGCCCACTACGAACACCCGTAAACATGCACCATCTATAACTTCAAAATCTCATCTATGCCTGCCTTCCCCTGACTGGTTAGACTAGT
CACCTAGCCATTCCGATGGCGACTTGTTGACTTGGAAAAATTGCATTGATCCTTTGTCTGGAGGTGAAATATCCAGTGGTGATCCAGT
GGTGACTTGTCGACTTTGTGATTATGCTGCTATATATAGTACACTTGTCTGACAGAAAAGAACTAAAAAATATCATAGTGTAAGTGTG
TAACCATCATGACATATATGATTTTGGTTATACACTTCATGTTCCATCACTGCTAAATACACAGGATATGATAAACTTCTGAATGTTGCT
AGAACCCTAATTTGATTGTTTGCATAGCTTATTGAGATGACAACTTAATAGCCCAAACCATTTTACTCCCTCCGATCCATATTAATTGTC
AAAATTACATGTATCTAGACGTTTTTTAGGCATAGATACATCCATATTTGGTCAAATTTGAGACAATTAATATGGATCGGAGGGAGTA
TTAGAAGCTGGTTGCTACTTTTCAGGGAGATGTACCTTTTTGTTCAATTTGTTATCTATTGTATATCTTTTTGGTTTGTGATATATGGAC
TGCTGTGAGTGTGTGCTGCTTTGTTGTTGCACATGCTTCGTTGCATAGGAATCCTTTTTACCTCTGCTGCAGAAGAGTATGTTGACTTC
TTTTTCATACTGTTCTGAAGTAATTTAGTAAAGTCTATTTATGTGTGTTGAATCTTGTATTCCTAGAGGTGAAACTGTGTATCTGCTGAA
CCAAATTTGCTGTTATACATATGAAAGCTTGCACTCACACCTAGCTTCGTCTTTCTTGTGATTCACAGAATGGGATGACAAAGAAGAC
GGCAATGAAGTGGTCCAGCAATGGGAGGACGATTGGGACGATGACGATGTGAACGATGATTTCTCGCTGCAGCTGAGGAAAGAGC
TGGAGGAAGGCAGTGCTCAGAAGAGCTGAACTACTTCCCTAGCAATGCCAAACTGTTGCTATCTTCGTTTAGGCACTGTGTGCGTCTG
CAAGCAGTCTAATTTGTGGAACAGTGGCAGTTTGTGCATGGCGTAGTACTATTCGGAAGTTTGCATGGATATACAGTTTGTGTATGGC
GTACCCGTAAGATCGGCCAGCTATTGAACTTGGATATGCTCCTTTCAATCCAATGTGGAACATTTGGTGACGCTGGTAGAATTCTCTTT
ATCGGCTGTGCCTTGTGATATGACAACGAGAATTGGTCCAATTGGATGATATTCACAAGCTGAAATTTACCCCAGTTTCAAAACACAA
CTTGTCCCAAACAAGCTCACTAAATTGTGCAGTTAGAATTAGAAATGATCTTGCATAAAACGCCATCTTTGTATGATCTGGTGTACAAA
TAAATACTCCCCCCGCTAGCCTTACACCCCTAAGCTCTTTGTTGCCTGTCATCACATCTTTCTGAAGCCAGACAAATCCCAATGTAACTT
ACTCAAGCTCATAGTTTTCAAATATTTTGTAAACTATTTTTTGGGGAAAATAGTTTTCAAAATATTCATCTAAGTGAGATTAATTTACTC
CCTCGGACCCATATTAGTTGTCTCAAATTTGCCCAAATTCGGGTGTATATATGTTTAAAAGCATCTAGATACACGTAATATTCGTAATA
TTTCGATAATTAATATGGGTCGGAGGGAGTAGTACATTTTAGAATTTGCTGTAAAGTTTGAAAAATCTTTTGAAATTTGGTCAAATTTA
GGTTGAAACTTTAGAAGCTCGTAGTTTTTGAAATGTTGACCCAAACGAGCTAAAACTTTCAGAAGTCACATAATTAGATATTTATTATT
TTCTGCATTCTTTTCAGATTTTTTATAAAATGTATAGCAAATTACTTTTGTTGGAAACCGTCAGACCTAGAATAGATGGAGATACGAAG
GGTAAGTTATGTTGTCATTTGTCAAAGGCAAATAACGTAATTCGGCTCCCTCTTCCTCCCGCCGCGTCACCAATTTCTATGTTTACTCAC
AGATCTCTCCTTCTCTATCCTCTTCGAAGCCTATTCCCCCCTTGCTCTTATTTAGCTGCCAAACATCATGAAGCCACCAAAGGCCGTCTC
TACCTCGGGTGTGGCTGTGCATAATCAAAGATCATCGTTACCGGTACCGATTGTTATCCAATAAACATTCTTTCCTTACATCTTTGATCT
CTCCTCCTTCCAAATTGATATTGGGCGAGCATTCACTATGCTGTGCAATCGATTATTCGTGCAATTATCAAGTTATATCTTTCTAAACTC
AAGGGGCGACGGTATTACCTGCTCTCAAGTGAGTCCCAATGGGTGTCGCTGTCACTAGTTGTCCTCAGGTCAGCCTCCCTTCCATTGC
ACATTTCCTCTTTCCTCCGAAATTAACAATATGTGAGAATTGCTGTACAAAATAATAAACAGGTGAGCATTCCCTCTCTGTCCGGCCAT
TGGATTTATCAAAAAAAGATAATCCTAGTTCAAATTTCGTTTCCATAAATTCAACCTTCTTTACCTTGAAATTTAAGCCCAGCGGTCCCT
TCCGGGACATCCGATAAAATTGAGATCTCGATAAAATTTTAACAGGGGCCTGACAGAGGTAGGCCGGAGGCCGTTGAGTTGGTGAC
TCCCGGGTTTCCCCAAGGAAATACCGAGATATTTGTTTGCCGTACCATACACTCCTCGCCGCACGCTGCGCCTGCGACGGCATAGTTC
GCCGCCGGAAACGCCCGTCTCCCAACGCGCGACGGCGCGAGGCTACCTCCCGTTATCGAGGAAATCCATTCGCCGCAGCGGGCGCC

>BdiBd21-3.2G0134900

TAGTACTGCTACGCCGTACGCGCGTCTCGGAACATGCGGTGCCAGCAATCATACTGAAAATTGGTGCAGATTTGACGGTGCTCCTTCA
GAATTGACCTCTTGAGTAAACATCATAGCTAGGACGGTGTACCATGATGTTTATCTTCAGTCGTTGTGGAGCATTACAGTAAGCGAGG
TTTGTCGCCAATATCCGATGCAACATCGCCTATGTGGACGTTTAAAAGAGAAAAACACAAATGCACATATAAGCGTCGCCCACTTCAA
TACTCCATCACAGTACACTGTACACCAATGCAGAACACAAATGAAAACGCACCCGTGATTCACCTATGCTCCAAGTTAAACGTCGATG
CTAACTTGTTTGGAATAGATCCTGAACAAACTAAAAGGCTGGGATTTCAGCTCCACTCTCAAGCATCCCATTGGAGATGGCTCTAGGT
GATTAGTTGACAAGTGACCTCCATTCCTCTTCGTCATATTCTACTAATCATGGTCTGTAAGAACATGCTTCGGCTTTGGATCAATCAATT
GCTCATTTTCTCCTTGATGCAAAGCCTCCATCAACCACAAGAATGACGACTTTGCCATCACGATTCGTCTTTCTCTATGCTGTTGAGGAT
GTTCTTAGTAATATATACATTTTATGAAGTTCCCATACCAATTGCAAATCATTTCAAGAAAAAAAAATTAACCTCACAAAATAGTGTGC
ATAGCAAAGGAGATTATAGGGGAGACAAATATAAGGAGATAGTGAGTTCTTGCCTGTCGAAGGTCAAGATGTGGCACTTTGATCGA
GAATAAGAGAGCTGAAACTTCTATTGGAAACAAGCTAGCAATGGTAGTCATATAAATAAATTGTCAATAAAGATGGATGACAAAAG
GTTAGATATTATTGTTTAATGTTTAGTCATATGTTTTATTAGCCAAAAGAAGCAACGAACAGAGTTAGACACAACGCAAATCATACACT
ATTCACCGGTGGAGATAAAGGGACGAACGATGAATCACTCACAAAATATAACTAGAGGTGAACCATCAAAGCTATCGGTTTGGCTTC
CTCGTAGGAAACCGAACACCATGAAGGTCGTGGGTCCAAACTCAAACCATAACAATCTATTTTCATGGTATAGATTTAGCGTTCCATA
AAGTCAACGGCAAAGTCTTATCAATGACTCAACAATGTACTTTATCTCGGTCTGAATTGCATATTTATAATTTATTTTTACAAAAGAACT
GTAGTTCTTTAAGTTGTTATATGAATAAAAATCATGCTACACCATCAGTGTTTTCCTTTTGCTACAAAACTATTGTGATATTTAAATTTA
TTGTCAGGATACCGCTATATTGCATCATGGAAAAATGAGAAATTGTCCAATCTTAAGACAAACACATAAATATTTCCTTTTACAAACTC
TTGTAGGGTATAGTCACATTTTGTCCCTTAAATTTTAGGAAATCACCTGTTATAGAGTGAAACACGTCCTTTTTCATACGATCGGTTTGT
TACGACAAATTACGATTAGTTTTTTCTTACTGTAATTGTTGATAAAAACCCAGCCAACTTATGCTGTCCACTAAATGCTACATTCTTTGT
CGTAACAAAATCGTACTCCCATCGATGCATTAAAATTCAAGACGCTTGTTAGTGATTGGAGGGATATGTAAATGTTCGCAGGTATAGC
AGTCCTCTTTTCCACAACTTTCAGATATTTTGGAAAATCAGATGGCCAAAAAATGTGTTGGTGGGTGACCCCGTTCCGTACTGAAAAG
AAACAAACACGGCTCGAACTGCCCCGAATCGACGGCACCGCTTTACTAACCCCGAGTCTACGACCCCATTGGTCTGCCACTAACCATC
GTCTCTGTCCCGAACGAGCAATCGCCCCTAGGGTTTCTCCAACCACACGCGAGTACGCGACGCGAATCAGGTCGGTGGCGTCCAGAT
CCTTCTCCCTCCTTTCCTCCCCAAAATCTCCAATCCGGATCTCTGTACTCACCCTCTGATCTGCTCGGCCGCGTCGCAGGTACTAGACAC
CAGCACCAGCGCTGATGGCGGCGGCCCCGGCGGACGCGAAAGCTGAGGCGGCCAAGATGGACCTCCTCGAGGACGACGACGAGTT
CGAGGAGTTCGAGATCGACCAAGGTATGTCCACGCGCTCTCCGCTGCCCCCCTCTCGTTACTACCATGTTGCTATATTTAGGCTAGGC
TAACGAGATCGATTCGCCGTAGCTTGTTTGCGCCGGGAGGCCGATTTGGGCGACTAGGTTTGGGTTGATTCGGAACCTGTACCATGT
CACACTTCCTTTGAGATTCGTTATGGAAGAGATTTGCGTGAAATTAAGGCAAAGTACAGACACTGGTATCGATTTTACAGGGCATTAG
GTAGGTCGTGACGTCAGGTAGTGCTCTCAGTGACCTGACTTAGTTATGGTCTCCACTGCTGTTCCTGGGTAACTCCCCTCGCGCTTGTT
GTGATATGTAAATAGCTAGGTGGTTTTGATAGATACATGTATATGTCCCGTTTAGTTTAGCATGAAACCGGTGCTTTAATAGTGGTGT
GTATTTATGAGCGTCCACTTCTTGGCATGATTGCTTCTTTTAGGGAAAAATATTAGGCACATGAAACTATGTTTATCTGCAGTTGGGCT
TGTAGTTGTTTGAGACAATGAGACCCCAAGTGTTGGGATGTAGCTTAAGGAAAACACAAAATATATCTCCTTGAAATTGCATGTTGG
AACTAATTTTCTCCATTGGGAATAACAATCTTCACTTCCTGTTTCTTCACTGTTAACATGAAGAGAACTGCACCATTAGAATGACACAA
ACCCTGAAGCTTGAACATATATTGGAAAATAAACCCATTGTTGTTGAGCAATTGATCAAAGACTGTTTGCTTCTCCTTTCAATTTCATTA
AAATGCTATAGTGTAGCAGTAATGAGTTTGTAGTAGTAATGAGTTTTTGATCTAATTAGTTTGTTGCATCTGTACACATGCTATTCATG
TTGGTGGGCCGGCAATAGTGAAAATACATAATACAATGAAATATATCAGTGGGAATTTGGGCTCAGGTGCCCCTTCTTAAAATTTAAT
GTTATATAAGACATGACGGTTCATGTAAATGGCAAAAAGACAGTAGGAGATCTCCAAACAATGCAATAATTTTTTTCTTTGATTGTTT
GATTGATTGAACCAAAAAGAAGTTGTGTGATGTCTTTTAGTTTGCAGGATGCACAATTGTTTGTTTCTTACTTCATACTTTACATCAAT
GGTCAGACTTGTTTTGCAATCAGAGAGGGTAAGATACAGGGTGAAAATTGCTCGTCATTTCAACAAGTTTTAGCCATGCTTCCTTAAT
GAAAGTTTTCCAGTTTCACTTATCGTTTGCATGTTGTCACTCATGATTAGCCATCATGGTGCAGCCACACACCTAGCAGCAGAGCAAAT
CGAGTGAGTTGCTATAGCCGCTGGCTTGCTCTGACTAGTAGTTGGCTAGGATCTTAAAGTTGCCTGACTTGGTGTGCTATGGCCCACT
GTCAGGACATCGACAAGTTACTCTCTTCCAGTCTAGAGGAGGTGAATTCCAAAAGCAGAGCACCGCATCAAGTCAAGGATATAAGAG
TGTGTCAGAAGCAGAAAACCTTAGCCCAGGAGATTTATGTTAGCTATGAATATTTTTGCCACTTAAAGTATTCTTACATGTGTTATGCC
CACTACGAACACCCGTAAACATGCACCATCTATAACTTCAAAATCTCATCTATGCCTGCCTTCCCCTGACTGGTTAGACTAGTCACCTA
GCCATTCCGATGGCGACTTGTTGACTTGGAAAAATTGCATTGATCCTTTGTCTGGAGGTGAAATATCCAGTGGTGATCCAGTGGTGAC
TTGTCGACTTTGTGATTATGCTGCTATATATAGTACACTTGTCTGACAGAAAAGAACTAAAAAATATCATAGTGTAAGTGTGTAACCAT
CATGACATATATGATTTTGGTTATACACTTCATGTTCCATCACTGCTAAATACACAGGATATGATAAACTTCTGAATGTTGCTAGAACC
CTAATTTGATTGTTTGCATAGCTTATTGAGATGACAACTTAATAGCCCAAACCATTTTACTCCCTCCGATCCATATTAATTGTCAAAATT
ACATGTATCTAGACGTTTTTTAGGCATAGATACATCCATATTTGGTCAAATTTGAGACAATTAATATGGATCGGAGGGAGTATTAGAA

GCTGGTTGCTACTTTTCGGGGAGATGTACCTTTTTGTTCAATTTGTTATCTATTGTATATCTTTTTGGTTTGTGATATATGGACTGCTGT
GAGTGTGTGCTGCTTTATTGTTGCACATGCTTCGCTGCATAGGAATCCTTTTTACCTCTGCTGCAGAAGAGTATGTTAACTTCTTTTTCA
TACTGTTCTGAAGTAATTTAGTAAAGTCTATTTATGTGTGTTGAATCTTGTATTCCTAGAGGTGAAACTGTGTATCTGCTGAACCAAAT
TTGCTGTTATACATATGAAAGCTTGCACTCACACCTAGCTTCCGTCTTTCTTGTGATTCACAGAATGGGATGACAAAGAAGACGGCAA
TGAAGTGGTCCAGCAATGGGAGGACGATTGGGACGATGACGATGTGAACGATGATTTCTCGCTGCAGCTGAGGAAAGAGCTGGAG
GAAGGCAGTGCTCAGAAGAGCTGAACTACTTCCCTAGCAATGCCAAACTGTTGCTATCTTCGTTTAGGCACTGTGTGCGTCTGCAAGC
AGTCTAATTTGTGGAACAGTGGCAGTTTGTGCATGGCGTAGTACTATTCGGAAGTTTGCATGGATATACAGTTTGTGTATGGCGTACC
CGTAAGATCGGCCAGCTATTGAACTTGGATATGCTCCTTTCAATCCAATGTGGAACATTTGGTGACGCTGGTAGAATTCTCTTTATCG
GCTGTGCCTTGTGATATGACAACGAGAATTGGTCCAATTGGATGATATTCACAAGCTGAAATTTACCCCAATTTCAAAACACAACTTG
TCCCAAACAAGCTCACTAAATTGTGCAGTTAGAATTAGAAATGATCTTGCATAAAACGCCATCTTTGTATGATCTGGTGTACAAATAA
ATACTCCCCCCGCTAGCCTTATACCCCCAAGCTCTTTGTTGCCTGTCATCACATCTTTCTGAAGCCAGACAAATCCCAATGTAACTTACT
CAAGCTCATAGTTTTCAAATATTTTGTAAACTATTTTTTGGGGAAAATAGTTTTCAAAATATTCATCTAAGTGAGATTAATTTACTCCCT
CGGACCCATATTACTTGTCTCAAATTTGCCTAAATTCGGGTGTATATATGTTTAAAAGCATCTAGATACACGTAATATTTCGATAAGTA
ATATGGGTCGGAGGGAGTAGTACATTTTAGAATTTGCTGTAAAGTTTGAAAAATCTTTTGAAATTTGGTCAAATTTAGGTTGAAACTT
TAGAAGCTCGTAGTTTTCGAATGTTGACCCAAACGAGCTAAAACTTTCAGAAGTCACATAATTAGATATTTATTATTTTCTGCATTCTTT
TCAGATTTTTTATAAAATGTATAGCAAATTACTTTTGTTGGAAACCGTCAGACCTAGAATAGATGGAGATACGAAGGGTAAGTTATGT
TGTCATTTGTCAAAGGCAAATAACGTAATTCGGCTCCCTCTTCCTCCCGCCGCGTCACCAATTTCTATGTTTACTCGCAGATCTCTCCTT
CTCTATCCTCTTCGAAGCCTATTCTCCCCTTGCTCTTATTTCGCTGCCAAACATCATGAAGCCACCAAAGGCCGTCTCTACCTCGGGTGT
GGCTGTGCATAATCAAAGATCATCGTTACCGGTACCGATTGTCATCCAATAAACATTCTTTCCTTACATCTTTGATCTCTCCTCCTTCCA
AATTGATGTTGGGCGAGCATTCACTATGCTGTGCAATCGATTATTCGTGCAATTATCAAGTTATATCTTTCTAAACTCAACGGGCGACG
GTATTATCTGCTCTCAAGTGAGTCCCAATGGGTGTCGCTGTCACTAGTTGTCCTCAGGTCAGCCTCCCTTCCATTGCACATTTCCTCTTT
CCTCCGAAATTAACAATATGTGAGAATTGCTGTACAAAATAATGAACAGGTGAGCATTCCCTCTCTGTCCGGCCATTGGATTTATCAA
AAAAAGATAATCCTAGTTCAAATTTCGTTTCCATAAATTCAACCTTCTTTACCTTGAAATTTAAGCCCAGCGGTCCCGACATCCGATAA
AATTGAGATCTCGATAAAATTTTAACAGGGGCCTGACAGAGGTAGGCCGGAGGCCGTTGAGTTGGTGACTCCCGGGTTTCCCCAAG
GAAATACCGAGATATTTGTTTGCCGTACCATACACTCCTCGCCGCACGCTGCGCCTGCGACGGCATAGTTCGCCGCCGGAAACGCCC
GTCTCCCAACGCGCGACGGCGCGAGGCTACCTCCCGTTATCGAGGAAATCCATTCGCCGCAGCGGGCGCC

```
Alignment of Sequence_1:  [Untitled Sequence #1] with  Sequence_2: [Sequence Window #2]

Similarity : 5919/6529 (90.66 %)

Seq_1  1     GGGGGGATGGGGATTGGTGGGATTCGCGTCTAGTGACCGTTGGGGGCGGCGCTAGCTGTT  60

Seq_2  1     ------------------------------------------------------------  0


Seq_1  61    TCTGATGCCCGTTGCGTGCAGCAATTACTCGCGACAGCTTTCCGGTGAGGCTGGCTCCAA  120

Seq_2  1     ------------------------------------------------------------  0


Seq_1  121   CGGCTAGTTTGGGCCGGCGGTGCCGGTGTCACACGCAGACTTGACTGACTGCCCTGTCTG  180

Seq_2  1     ------------------------------------------------------------  0


Seq_1  181   CTCTGCAGTTGGTCACGTCACGTAGTACTGCTACGCCGTACGTGCGTCTCGGAACATGCG  240
                                   |||||||||||||||||||| |||||||||||||||||
Seq_2  1     ----------------------TAGTACTGCTACGCCGTACGCGCGTCTCGGAACATGCG  38


Seq_1  241   GTGCCAGCAATCATACTGAAAATTGGTGCAGATTTGAC--------------------CT  280
             ||||||||||||||||||||||||||||||||||||||                    ||
Seq_2  39    GTGCCAGCAATCATACTGAAAATTGGTGCAGATTTGACGGTGCTCCTTCAGAATTGACCT  98


Seq_1  281   CTTGAGTAAACATCATAGCTAGGACGGTGTACCATGATGTTTATCTTCAGTCGTTGTGGG  340
             ||||||||||||||||||||||||||||||||||||||||||||||||||||||||||| 
Seq_2  99    CTTGAGTAAACATCATAGCTAGGACGGTGTACCATGATGTTTATCTTCAGTCGTTGTGGA  158
```

```
Seq_1  341   GCATTACAGTAAGCGAGGTTTGTCGCCAATATCCGATGCAACATCGCCTATGTGGACGTT  400
             ||||||||||||||||||||||||||||||||||||||||||||||||||||||||||||
Seq_2  159   GCATTACAGTAAGCGAGGTTTGTCGCCAATATCCGATGCAACATCGCCTATGTGGACGTT  218

Seq_1  401   TAAAAGAGAAAAACACAAATGCACATATAAGCGTCGCCCACTTCAATACTCCATCACAGT  460
             ||||||||||||||||||||||||||||||||||||||||||||||||||||||||||||
Seq_2  219   TAAAAGAGAAAAACACAAATGCACATATAAGCGTCGCCCACTTCAATACTCCATCACAGT  278

Seq_1  461   ACACTGTACACCAATGCAGAACACAACTAAAAACGCACCCGTGATTCACCTATGCTCCAA  520
             |||||||||||||||||||||||||| | |||||||||||||||||||||||||||||||
Seq_2  279   ACACTGTACACCAATGCAGAACACAAATGAAAACGCACCCGTGATTCACCTATGCTCCAA  338

Seq_1  521   GTTAAACGTCGATGCTAACTTGTTTGGAATAGATCCTGAACAAACTAAAAGGCTGGGCTT  580
             ||||||||||||||||||||||||||||||||||||||||||||||||||||||||| ||
Seq_2  339   GTTAAACGTCGATGCTAACTTGTTTGGAATAGATCCTGAACAAACTAAAAGGCTGGGATT  398

Seq_1  581   TCAGCTCCACTCTCAAGCATCCCA--GGAGATGGCTCTAGGTGATTAGTTGACAAGTGAC  638
             ||||||||||||||||||||||||  ||||||||||||||||||||||||||||||||||
Seq_2  399   TCAGCTCCACTCTCAAGCATCCCATTGGAGATGGCTCTAGGTGATTAGTTGACAAGTGAC  458

Seq_1  639   CTCCATTCCTCTTCGTCATATTCTATTAATCATGGTCTGTAAGAACATGCTTCGGCTTTG  698
             ||||||||||||||||||||||||| ||||||||||||||||||||||||||||||||||
Seq_2  459   CTCCATTCCTCTTCGTCATATTCTACTAATCATGGTCTGTAAGAACATGCTTCGGCTTTG  518

Seq_1  699   GATCAATCAATTGCTCATTTTCTCCTTGATGCAAAGCCTCCATCAACCACAAGAATGACG  758
             ||||||||||||||||||||||||||||||||||||||||||||||||||||||||||||
Seq_2  519   GATCAATCAATTGCTCATTTTCTCCTTGATGCAAAGCCTCCATCAACCACAAGAATGACG  578

Seq_1  759   ACTTTGCCATCACGATTCGTCTTTCTCTATGGTGTTGAGGATGTTCTTAGTAATATATAA  818
             ||||||||||||||||||||||||||||||| ||||||||||||||||||||||||||| 
Seq_2  579   ACTTTGCCATCACGATTCGTCTTTCTCTATGCTGTTGAGGATGTTCTTAGTAATATATAC  638

Seq_1  819   ATTTTATGAAGTTCCCATACCAATTGCAAATCATTTCGAGAAAAAATAATTAACCTCACA  878
             ||||||||||||||||||||||||||||||||||||| |||||||| |||||||||||||
Seq_2  639   ATTTTATGAAGTTCCCATACCAATTGCAAATCATTTCAAGAAAAAAAAAATTAACCTCACA  698

Seq_1  879   AAATAGTGTGCATAGCAAAGGAGATTATAGGGGAGACAAATATAAGGAGATAGTGAGTTC  938
             ||||||||||||||||||||||||||||||||||||||||||||||||||||||||||||
Seq_2  699   AAATAGTGTGCATAGCAAAGGAGATTATAGGGGAGACAAATATAAGGAGATAGTGAGTTC  758

Seq_1  939   TTGCCTGTCGAAGGTCAAGATGTGGCACTTTGATCGAGAATAAGAGAGCTGAAACTTCTA  998
             ||||||||||||||||||||||||||||||||||||||||||||||||||||||||||||
Seq_2  759   TTGCCTGTCGAAGGTCAAGATGTGGCACTTTGATCGAGAATAAGAGAGCTGAAACTTCTA  818

Seq_1  999   TTGGAAACAAGCTAGCAATGGTAGTCATATAAATAACTTGTCAATAAAGATGGATGACAA  1058
             |||||||||||||||||||||||||||||||||||| |||||||||||||||||||||||
Seq_2  819   TTGGAAACAAGCTAGCAATGGTAGTCATATAAATAAATTGTCAATAAAGATGGATGACAA  878

Seq_1  1059  AAGGTTAGATATTATTGTTTAATGTTTAGTCATATGTTTTATTAGCCAAAAGAAGCAACG  1118
             ||||||||||||||||||||||||||||||||||||||||||||||||||||||||||||
Seq_2  879   AAGGTTAGATATTATTGTTTAATGTTTAGTCATATGTTTTATTAGCCAAAAGAAGCAACG  938

Seq_1  1119  AACAGAGTTAGACACAACGCAGATCATACACTATTCACCGGTGGAGATAAAGGGACGAAC  1178
             ||||||||||||||||||||| ||||||||||||||||||||||||||||||||||||||
Seq_2  939   AACAGAGTTAGACACAACGCAAATCATACACTATTCACCGGTGGAGATAAAGGGACGAAC  998
```

```
Seq_1  1179  GATGAATCACTCACAAAATATAACTGGAGGTGAACCATCAAAGCTATCGGTTTGGCTTCC  1238
             ||||||||||||||||||||||||| ||||||||||||||||||||||||||||||||||
Seq_2  999   GATGAATCACTCACAAAATATAACTAGAGGTGAACCATCAAAGCTATCGGTTTGGCTTCC  1058


Seq_1  1239  CCGTAGGAAACCGAACACCATGAAGGCCGTGGGTCCAAACTCAAACCATAACAATCTATT  1298
              ||||||||||||||||||||||||| |||||||||||||||||||||||||||||||||
Seq_2  1059  TCGTAGGAAACCGAACACCATGAAGGTCGTGGGTCCAAACTCAAACCATAACAATCTATT  1118


Seq_1  1299  TTCATGGTATAGATTTGGCATTCCATAAAGTCAACGGCAAAGTCTTATCAATGACTCAAC  1358
             |||||||||||||||| || ||||||||||||||||||||||||||||||||||||||||
Seq_2  1119  TTCATGGTATAGATTTAGCGTTCCATAAAGTCAACGGCAAAGTCTTATCAATGACTCAAC  1178


Seq_1  1359  AATGTACTTTATCTCGGTCTAAATTGCATATTTATAATTTATTTTTACAAAAAAACTGTA  1418
             |||||||||||||||||||| ||||||||||||||||||||||||||||||| |||||||
Seq_2  1179  AATGTACTTTATCTCGGTCTGAATTGCATATTTATAATTTATTTTTACAAAAGAACTGTA  1238


Seq_1  1419  GTTCTTTAAGTTGTTATATGAATAAAAATCATGCTACACCATCGGTGTTTTCCTTTTGCT  1478
             ||||||||||||||||||||||||||||||||||||||||||| ||||||||||||||||
Seq_2  1239  GTTCTTTAAGTTGTTATATGAATAAAAATCATGCTACACCATCAGTGTTTTCCTTTTGCT  1298


Seq_1  1479  ACAAAACTATTGTGATATTTAAATTTATTGTCAGGATACCGCTATATTGCATCATGGAAA  1538
             ||||||||||||||||||||||||||||||||||||||||||||||||||||||||||||
Seq_2  1299  ACAAAACTATTGTGATATTTAAATTTATTGTCAGGATACCGCTATATTGCATCATGGAAA  1358


Seq_1  1539  AATGAGAAATTGTCCAATCTTAAGACAAACACATAAATATTTCCTTTTACAAACTCTTGT  1598
             ||||||||||||||||||||||||||||||||||||||||||||||||||||||||||||
Seq_2  1359  AATGAGAAATTGTCCAATCTTAAGACAAACACATAAATATTTCCTTTTACAAACTCTTGT  1418


Seq_1  1599  CAGGTATAGTCACATTTTGTCCCTTAAATTTTAGGAAATCACCTGTTATAGAGTGAAACA  1658
               ||||||||||||||||||||||||||||||||||||||||||||||||||||||||||
Seq_2  1419  AGGGTATAGTCACATTTTGTCCCTTAAATTTTAGGAAATCACCTGTTATAGAGTGAAACA  1478


Seq_1  1659  CGTCCTTTTGCATACGATCGGTTTGTTACGACAAATTACGATTAGTTTTTTCTTACTGTA  1718
             ||||||||| ||||||||||||||||||||||||||||||||||||||||||||||||||
Seq_2  1479  CGTCCTTTTTCATACGATCGGTTTGTTACGACAAATTACGATTAGTTTTTTCTTACTGTA  1538


Seq_1  1719  ATTGTTGATAAAAACCCAGCCAACTTATGCTGTCCACTAAATGCTACATTCTTTGTCGTA  1778
             ||||||||||||||||||||||||||||||||||||||||||||||||||||||||||||
Seq_2  1539  ATTGTTGATAAAAACCCAGCCAACTTATGCTGTCCACTAAATGCTACATTCTTTGTCGTA  1598


Seq_1  1779  ACAAAATCGTACTCCCATCGATGCATTAAAATTCAAGACGCTTGTTAGTGATTGGAGGGA  1838
             ||||||||||||||||||||||||||||||||||||||||||||||||||||||||||||
Seq_2  1599  ACAAAATCGTACTCCCATCGATGCATTAAAATTCAAGACGCTTGTTAGTGATTGGAGGGA  1658


Seq_1  1839  TATGTAAATGTTCGCAGGTATAGCAGTCCTCTTTTCCACGACTTTCAGATATTTTGGAAA  1898
             ||||||||||||||||||||||||||||||||||||||| ||||||||||||||||||||
Seq_2  1659  TATGTAAATGTTCGCAGGTATAGCAGTCCTCTTTTCCACAACTTTCAGATATTTTGGAAA  1718


Seq_1  1899  ATCAGATGGCCAAAAAATGTGTTGGTGGGTGACCCCGTTCCGTACTGAAAAGAAACAAAC  1958
             ||||||||||||||||||||||||||||||||||||||||||||||||||||||||||||
Seq_2  1719  ATCAGATGGCCAAAAAATGTGTTGGTGGGTGACCCCGTTCCGTACTGAAAAGAAACAAAC  1778


Seq_1  1959  ACGGCTCGAACTGCCCCGAATCGACGGCACCGCTTTACTAACCCCGAGTCTACGACCCCA  2018
             ||||||||||||||||||||||||||||||||||||||||||||||||||||||||||||
Seq_2  1779  ACGGCTCGAACTGCCCCGAATCGACGGCACCGCTTTACTAACCCCGAGTCTACGACCCCA  1838
```

```
Seq_1  2019  TTGGTCTGCCACGAACCATCGTCTCTGTCCCGAACGAGCAATCGCCCCTAGGGTTTCTCC  2078
             |||||||||||| |||||||||||||||||||||||||||||||||||||||||||||||
Seq_2  1839  TTGGTCTGCCACTAACCATCGTCTCTGTCCCGAACGAGCAATCGCCCCTAGGGTTTCTCC  1898

Seq_1  2079  AACCACACGCGAGTACGCGACGCGAATCAGGTCGGTGGCGTCCAGATCCTTCTCCCTCCT  2138
             ||||||||||||||||||||||||||||||||||||||||||||||||||||||||||||
Seq_2  1899  AACCACACGCGAGTACGCGACGCGAATCAGGTCGGTGGCGTCCAGATCCTTCTCCCTCCT  1958

Seq_1  2139  TTCCACCCCAAAATCTCCAATCCGGATCTCTGTACTCACCCTCTGATCTGCTCGGCCGCG  2198
             |||| |||||||||||||||||||||||||||||||||||||||||||||||||||||||
Seq_2  1959  TTCCTCCCCAAAATCTCCAATCCGGATCTCTGTACTCACCCTCTGATCTGCTCGGCCGCG  2018

Seq_1  2199  TCGCAGGTACTAGACACCAGCACCAGCGCTGATGGCGGCGGCCCCGGCGGACGCGAAAGC  2258
             ||||||||||||||||||||||||||||||||||||||||||||||||||||||||||||
Seq_2  2019  TCGCAGGTACTAGACACCAGCACCAGCGCTGATGGCGGCGGCCCCGGCGGACGCGAAAGC  2078

Seq_1  2259  TGAGGCGGCCAAGATGGACCTCCTCGAGGACGACGACGAGTTCGAGGAGTTCGAGATCGA  2318
             ||||||||||||||||||||||||||||||||||||||||||||||||||||||||||||
Seq_2  2079  TGAGGCGGCCAAGATGGACCTCCTCGAGGACGACGACGAGTTCGAGGAGTTCGAGATCGA  2138

Seq_1  2319  CCAAGGTATGTCCACGCGCTCTCCGCTGCCCCCCTCTCGTTACTACCATGTTGCTATATT  2378
             ||||||||||||||||||||||||||||||||||||||||||||||||||||||||||||
Seq_2  2139  CCAAGGTATGTCCACGCGCTCTCCGCTGCCCCCCTCTCGTTACTACCATGTTGCTATATT  2198

Seq_1  2379  TAGGCTAGGCTAACGAGATCGATTCGCCGTAGCTTGTTTGCGCCGGGAGGCCGATTTGGG  2438
             ||||||||||||||||||||||||||||||||||||||||||||||||||||||||||||
Seq_2  2199  TAGGCTAGGCTAACGAGATCGATTCGCCGTAGCTTGTTTGCGCCGGGAGGCCGATTTGGG  2258

Seq_1  2439  CGACTAGGTTTGGGTTGATTCGGAACCTGTACCATGTCACACTTCCTTTGAGATTCGTTA  2498
             ||||||||||||||||||||||||||||||||||||||||||||||||||||||||||||
Seq_2  2259  CGACTAGGTTTGGGTTGATTCGGAACCTGTACCATGTCACACTTCCTTTGAGATTCGTTA  2318

Seq_1  2499  TGGAAGAGATTTGCGTGAAATTAAGGCAAAGTACAGACACTGGTATCGATTTTACAGGGC  2558
             ||||||||||||||||||||||||||||||||||||||||||||||||||||||||||||
Seq_2  2319  TGGAAGAGATTTGCGTGAAATTAAGGCAAAGTACAGACACTGGTATCGATTTTACAGGGC  2378

Seq_1  2559  ATTAGGTAGGTCGTGACGTCAGGTAGTGCTCTCAGTGACCTGACTTAGTTATGGTCTCCA  2618
             ||||||||||||||||||||||||||||||||||||||||||||||||||||||||||||
Seq_2  2379  ATTAGGTAGGTCGTGACGTCAGGTAGTGCTCTCAGTGACCTGACTTAGTTATGGTCTCCA  2438

Seq_1  2619  CTGCTGTTCCTGGGTAACTCCCCTCGCGCTTGTTGTAATATGTAAATAGCTAGCTGGTTT  2678
             |||||||||||||||||||||||||||||||||||| |||||||||||||||| ||||||
Seq_2  2439  CTGCTGTTCCTGGGTAACTCCCCTCGCGCTTGTTGTGATATGTAAATAGCTAGGTGGTTT  2498

Seq_1  2679  TGATAGATACATGTATATGTCCCGTTTAGTTTAGCATGAAACCGGTGCTTTAATAGTGGT  2738
             ||||||||||||||||||||||||||||||||||||||||||||||||||||||||||||
Seq_2  2499  TGATAGATACATGTATATGTCCCGTTTAGTTTAGCATGAAACCGGTGCTTTAATAGTGGT  2558

Seq_1  2739  GTGTATTTATGAGCGTCCACTTCTTGGCATGATTGCTTCTTTTAGGGAAAAATATTAGGC  2798
             ||||||||||||||||||||||||||||||||||||||||||||||||||||||||||||
Seq_2  2559  GTGTATTTATGAGCGTCCACTTCTTGGCATGATTGCTTCTTTTAGGGAAAAATATTAGGC  2618

Seq_1  2799  ACATGAAACTATGTTTATCTGCAGTTGGGCTTGTAGTTGTTTGAGACAATGAGACCCCAA  2858
             ||||||||||||||||||||||||||||||||||||||||||||||||||||||||||||
Seq_2  2619  ACATGAAACTATGTTTATCTGCAGTTGGGCTTGTAGTTGTTTGAGACAATGAGACCCCAA  2678
```

```
Seq_1  2859  GTGTTGGGATGTAGCTTAAGGAAAACACAAAATATATCTCCTTGAAATTGCATGTTGGAA  2918
             ||||||||||||||||||||||||||||||||||||||||||||||||||||||||||||
Seq_2  2679  GTGTTGGGATGTAGCTTAAGGAAAACACAAAATATATCTCCTTGAAATTGCATGTTGGAA  2738

Seq_1  2919  CTAATTTTCTCCATTGGGAATAACAATCTTCACTTCCTGTTTCTTCACTGTTAACATGAA  2978
             ||||||||||||||||||||||||||||||||||||||||||||||||||||||||||||
Seq_2  2739  CTAATTTTCTCCATTGGGAATAACAATCTTCACTTCCTGTTTCTTCACTGTTAACATGAA  2798

Seq_1  2979  GAGAACTGCACCATTAGAATGACACAAACCCTGAAGCTTGAACATATATTGGAAAATAAA  3038
             ||||||||||||||||||||||||||||||||||||||||||||||||||||||||||||
Seq_2  2799  GAGAACTGCACCATTAGAATGACACAAACCCTGAAGCTTGAACATATATTGGAAAATAAA  2858

Seq_1  3039  C-----------------------------------------------------------  3039
             |
Seq_2  2859  CCCATTGTTGTTGAGCAATTGATCAAAGACTGTTTGCTTCTCCTTTCAATTTCATTAAAA  2918

Seq_1  3040  ------------------------------------------------------------  3039

Seq_2  2919  TGCTATAGTGTAGCAGTAATGAGTTTGTAGTAGTAATGAGTTTTTGATCTAATTAGTTTG  2978

Seq_1  3040  ------------------------------------------------------------  3039

Seq_2  2979  TTGCATCTGTACACATGCTATTCATGTTGGTGGGCCGGCAATAGTGAAAATACATAATAC  3038

Seq_1  3040  ------------------------------------------------------------  3039

Seq_2  3039  AATGAAATATATCAGTGGGAATTTGGGCTCAGGTGCCCCTTCTTAAAATTTAATGTTATA  3098

Seq_1  3040  ------------------------------------------------------------  3039

Seq_2  3099  TAAGACATGACGGTTCATGTAAATGGCAAAAAGACAGTAGGAGATCTCCAAACAATGCAA  3158

Seq_1  3040  ------------------------------------------------------------  3039

Seq_2  3159  TAATTTTTTTCTTTGATTGTTTGATTGATTGAACCAAAAAGAAGTTGTGTGATGTCTTTT  3218

Seq_1  3040  ------------------------------------------------------------  3039

Seq_2  3219  AGTTTGCAGGATGCACAATTGTTTGTTTCTTACTTCATACTTTACATCAATGGTCAGACT  3278

Seq_1  3040  ------------------------------------------------------------  3039

Seq_2  3279  TGTTTTGCAATCAGAGAGGGTAAGATACAGGGTGAAAATTGCTCGTCATTTCAACAAGTT  3338

Seq_1  3040  -----------------------------------------------------------T  3040
                                                                        |
Seq_2  3339  TTAGCCATGCTTCCTTAATGAAAGTTTTCCAGTTTCACTTATCGTTTGCATGTTGTCACT  3398

Seq_1  3041  CATGATTAGCCATCATGGTGCAGCCACACACCTAGCAGCAGAGCAAATCGAGTGAGTTGC  3100
             ||||||||||||||||||||||||||||||||||||||||||||||||||||||||||||
Seq_2  3399  CATGATTAGCCATCATGGTGCAGCCACACACCTAGCAGCAGAGCAAATCGAGTGAGTTGC  3458

Seq_1  3101  TATAGCTGCTGGCTTGCTCTGACTAGTAGTTGGCTAGGATCTTAAAGTTGCCTGACTTGG  3160
             |||||| |||||||||||||||||||||||||||||||||||||||||||||||||||||
Seq_2  3459  TATAGCCGCTGGCTTGCTCTGACTAGTAGTTGGCTAGGATCTTAAAGTTGCCTGACTTGG  3518
```

```
Seq_1  3161  TGTGCTATGGCCCACTGTCAGGACATCGACAAGTTACTCTCTTCCAGTCTAGAGGAGGTG  3220
             ||||||||||||||||||||||||||||||||||||||||||||||||||||||||||||
Seq_2  3519  TGTGCTATGGCCCACTGTCAGGACATCGACAAGTTACTCTCTTCCAGTCTAGAGGAGGTG  3578

Seq_1  3221  AATTCCAAAAGCAGAGCACCGCATCAAGTCAAGGATAGAAGAGTGTGTCAGAAGCAGAAA  3280
             ||||||||||||||||||||||||||||||||||||| ||||||||||||||||||||||
Seq_2  3579  AATTCCAAAAGCAGAGCACCGCATCAAGTCAAGGATATAAGAGTGTGTCAGAAGCAGAAA  3638

Seq_1  3281  ACCTTAGCCCAGGAGATTTATGTTAGCTATGAATATTTTTGCCACTTAAAGTATTCTTAC  3340
             ||||||||||||||||||||||||||||||||||||||||||||||||||||||||||||
Seq_2  3639  ACCTTAGCCCAGGAGATTTATGTTAGCTATGAATATTTTTGCCACTTAAAGTATTCTTAC  3698

Seq_1  3341  ATGTGTTATGCCCACTACGAACACCCGTAAACATGCACCATCTATAACTTCAAAATCTCA  3400
             ||||||||||||||||||||||||||||||||||||||||||||||||||||||||||||
Seq_2  3699  ATGTGTTATGCCCACTACGAACACCCGTAAACATGCACCATCTATAACTTCAAAATCTCA  3758

Seq_1  3401  TCTATGCCTGCCTTCCCCTGACTGGTTAGACTAGTCACCTAGCCATTCCGATGGCGACTT  3460
             ||||||||||||||||||||||||||||||||||||||||||||||||||||||||||||
Seq_2  3759  TCTATGCCTGCCTTCCCCTGACTGGTTAGACTAGTCACCTAGCCATTCCGATGGCGACTT  3818

Seq_1  3461  GTTGACTTGGAAAAATTGCATTGATCCTTTGTCTGGAGGTGAAATATCCAGTGGTGATCC  3520
             ||||||||||||||||||||||||||||||||||||||||||||||||||||||||||||
Seq_2  3819  GTTGACTTGGAAAAATTGCATTGATCCTTTGTCTGGAGGTGAAATATCCAGTGGTGATCC  3878

Seq_1  3521  AGTGGTGACTTGTCGACTTTGTGATTATGCTGCTATATATAGTACACTTGTCTGACAGAA  3580
             ||||||||||||||||||||||||||||||||||||||||||||||||||||||||||||
Seq_2  3879  AGTGGTGACTTGTCGACTTTGTGATTATGCTGCTATATATAGTACACTTGTCTGACAGAA  3938

Seq_1  3581  AAGAACTAAAAAATATCATAGTGTAAGTGTGTAACCATCATGACATATATGATTTTGGTT  3640
             ||||||||||||||||||||||||||||||||||||||||||||||||||||||||||||
Seq_2  3939  AAGAACTAAAAAATATCATAGTGTAAGTGTGTAACCATCATGACATATATGATTTTGGTT  3998

Seq_1  3641  ATACACTTCATGTTCCATCACTGCTAAATACACAGGATATGATAAACTTCTGAATGTTGC  3700
             ||||||||||||||||||||||||||||||||||||||||||||||||||||||||||||
Seq_2  3999  ATACACTTCATGTTCCATCACTGCTAAATACACAGGATATGATAAACTTCTGAATGTTGC  4058

Seq_1  3701  TAGAACCCTAATTTGATTGTTTGCATAGCTTATTGAGATGACAACTTAATAGCCCAAACC  3760
             ||||||||||||||||||||||||||||||||||||||||||||||||||||||||||||
Seq_2  4059  TAGAACCCTAATTTGATTGTTTGCATAGCTTATTGAGATGACAACTTAATAGCCCAAACC  4118

Seq_1  3761  ATTTTACTCCCTCCGATCCATATTAATTGTCAAAATTACATGTATCTAGACGTTTTTTAG  3820
             ||||||||||||||||||||||||||||||||||||||||||||||||||||||||||||
Seq_2  4119  ATTTTACTCCCTCCGATCCATATTAATTGTCAAAATTACATGTATCTAGACGTTTTTTAG  4178

Seq_1  3821  GCATAGATACATCCATATTTGGTCAAATTTGAGACAATTAATATGGATCGGAGGGAGTAT  3880
             ||||||||||||||||||||||||||||||||||||||||||||||||||||||||||||
Seq_2  4179  GCATAGATACATCCATATTTGGTCAAATTTGAGACAATTAATATGGATCGGAGGGAGTAT  4238

Seq_1  3881  TAGAAGCTGGTTGCTACTTTTCAGGGAGATGTACCTTTTTGTTCAATTTGTTATCTATTG  3940
             |||||||||||||||||||||| |||||||||||||||||||||||||||||||||||||
Seq_2  4239  TAGAAGCTGGTTGCTACTTTTCGGGGAGATGTACCTTTTTGTTCAATTTGTTATCTATTG  4298

Seq_1  3941  TATATCTTTTTGGTTTGTGATATATGGACTGCTGTGAGTGTGTGCTGCTTTGTTGTTGCA  4000
             ||||||||||||||||||||||||||||||||||||||||||||||||||| ||||||||
Seq_2  4299  TATATCTTTTTGGTTTGTGATATATGGACTGCTGTGAGTGTGTGCTGCTTTATTGTTGCA  4358
```

```
Seq_1  4001  CATGCTTCGTTGCATAGGAATCCTTTTTACCTCTGCTGCAGAAGAGTATGTTGACTTCTT  4060
             ||||||||| |||||||||||||||||||||||||||||||||||||||||| |||||||
Seq_2  4359  CATGCTTCGCTGCATAGGAATCCTTTTTACCTCTGCTGCAGAAGAGTATGTTAACTTCTT  4418

Seq_1  4061  TTTCATACTGTTCTGAAGTAATTTAGTAAAGTCTATTTATGTGTGTTGAATCTTGTATTC  4120
             ||||||||||||||||||||||||||||||||||||||||||||||||||||||||||||
Seq_2  4419  TTTCATACTGTTCTGAAGTAATTTAGTAAAGTCTATTTATGTGTGTTGAATCTTGTATTC  4478

Seq_1  4121  CTAGAGGTGAAACTGTGTATCTGCTGAACCAAATTTGCTGTTATACATATGAAAGCTTGC  4180
             ||||||||||||||||||||||||||||||||||||||||||||||||||||||||||||
Seq_2  4479  CTAGAGGTGAAACTGTGTATCTGCTGAACCAAATTTGCTGTTATACATATGAAAGCTTGC  4538

Seq_1  4181  ACTCACACCTAGCTTC-GTCTTTCTTGTGATTCACAGAATGGGATGACAAAGAAGACGGC  4239
             |||||||||||||||| |||||||||||||||||||||||||||||||||||||||||||
Seq_2  4539  ACTCACACCTAGCTTCCGTCTTTCTTGTGATTCACAGAATGGGATGACAAAGAAGACGGC  4598

Seq_1  4240  AATGAAGTGGTCCAGCAATGGGAGGACGATTGGGACGATGACGATGTGAACGATGATTTC  4299
             ||||||||||||||||||||||||||||||||||||||||||||||||||||||||||||
Seq_2  4599  AATGAAGTGGTCCAGCAATGGGAGGACGATTGGGACGATGACGATGTGAACGATGATTTC  4658

Seq_1  4300  TCGCTGCAGCTGAGGAAAGAGCTGGAGGAAGGCAGTGCTCAGAAGAGCTGAACTACTTCC  4359
             ||||||||||||||||||||||||||||||||||||||||||||||||||||||||||||
Seq_2  4659  TCGCTGCAGCTGAGGAAAGAGCTGGAGGAAGGCAGTGCTCAGAAGAGCTGAACTACTTCC  4718

Seq_1  4360  CTAGCAATGCCAAACTGTTGCTATCTTCGTTTAGGCACTGTGTGCGTCTGCAAGCAGTCT  4419
             ||||||||||||||||||||||||||||||||||||||||||||||||||||||||||||
Seq_2  4719  CTAGCAATGCCAAACTGTTGCTATCTTCGTTTAGGCACTGTGTGCGTCTGCAAGCAGTCT  4778

Seq_1  4420  AATTTGTGGAACAGTGGCAGTTTGTGCATGGCGTAGTACTATTCGGAAGTTTGCATGGAT  4479
             ||||||||||||||||||||||||||||||||||||||||||||||||||||||||||||
Seq_2  4779  AATTTGTGGAACAGTGGCAGTTTGTGCATGGCGTAGTACTATTCGGAAGTTTGCATGGAT  4838

Seq_1  4480  ATACAGTTTGTGTATGGCGTACCCGTAAGATCGGCCAGCTATTGAACTTGGATATGCTCC  4539
             ||||||||||||||||||||||||||||||||||||||||||||||||||||||||||||
Seq_2  4839  ATACAGTTTGTGTATGGCGTACCCGTAAGATCGGCCAGCTATTGAACTTGGATATGCTCC  4898

Seq_1  4540  TTTCAATCCAATGTGGAACATTTGGTGACGCTGGTAGAATTCTCTTTATCGGCTGTGCCT  4599
             ||||||||||||||||||||||||||||||||||||||||||||||||||||||||||||
Seq_2  4899  TTTCAATCCAATGTGGAACATTTGGTGACGCTGGTAGAATTCTCTTTATCGGCTGTGCCT  4958

Seq_1  4600  TGTGATATGACAACGAGAATTGGTCCAATTGGATGATATTCACAAGCTGAAATTTACCCC  4659
             ||||||||||||||||||||||||||||||||||||||||||||||||||||||||||||
Seq_2  4959  TGTGATATGACAACGAGAATTGGTCCAATTGGATGATATTCACAAGCTGAAATTTACCCC  5018

Seq_1  4660  AGTTTCAAAACACAACTTGTCCCAAACAAGCTCACTAAATTGTGCAGTTAGAATTAGAAA  4719
             | ||||||||||||||||||||||||||||||||||||||||||||||||||||||||||
Seq_2  5019  AATTTCAAAACACAACTTGTCCCAAACAAGCTCACTAAATTGTGCAGTTAGAATTAGAAA  5078

Seq_1  4720  TGATCTTGCATAAAACGCCATCTTTGTATGATCTGGTGTACAAATAAATACTCCCCCCGC  4779
             ||||||||||||||||||||||||||||||||||||||||||||||||||||||||||||
Seq_2  5079  TGATCTTGCATAAAACGCCATCTTTGTATGATCTGGTGTACAAATAAATACTCCCCCCGC  5138

Seq_1  4780  TAGCCTTACACCCCTAAGCTCTTTGTTGCCTGTCATCACATCTTTCTGAAGCCAGACAAA  4839
             |||||||| ||||| |||||||||||||||||||||||||||||||||||||||||||||
Seq_2  5139  TAGCCTTATACCCCCAAGCTCTTTGTTGCCTGTCATCACATCTTTCTGAAGCCAGACAAA  5198
```

```
Seq_1  4840  TCCCAATGTAACTTACTCAAGCTCATAGTTTTCAAATATTTTGTAAACTATTTTTTGGGG  4899
             ||||||||||||||||||||||||||||||||||||||||||||||||||||||||||||
Seq_2  5199  TCCCAATGTAACTTACTCAAGCTCATAGTTTTCAAATATTTTGTAAACTATTTTTTGGGG  5258


Seq_1  4900  AAAATAGTTTTCAAAATATTCATCTAAGTGAGATTAATTTACTCCCTCGGACCCATATTA  4959
             ||||||||||||||||||||||||||||||||||||||||||||||||||||||||||||
Seq_2  5259  AAAATAGTTTTCAAAATATTCATCTAAGTGAGATTAATTTACTCCCTCGGACCCATATTA  5318


Seq_1  4960  GTTGTCTCAAATTTGCCCAAATTCGGGTGTATATATGTTTAAAAGCATCTAGATACACGT  5019
              |||||||||||||||| ||||||||||||||||||||||||||||||||||||||||||
Seq_2  5319  CTTGTCTCAAATTTGCCTAAATTCGGGTGTATATATGTTTAAAAGCATCTAGATACACGT  5378


Seq_1  5020  AATATTCGTAATATTTCGATAATTAATATGGGTCGGAGGGAGTAGTACATTTTAGAATTT  5079
             ||||||         ||||||| |||||||||||||||||||||||||||||||||||||
Seq_2  5379  AATATT---------TCGATAAGTAATATGGGTCGGAGGGAGTAGTACATTTTAGAATTT  5429


Seq_1  5080  GCTGTAAAGTTTGAAAAATCTTTTGAAATTTGGTCAAATTTAGGTTGAAACTTTAGAAGC  5139
             ||||||||||||||||||||||||||||||||||||||||||||||||||||||||||||
Seq_2  5430  GCTGTAAAGTTTGAAAAATCTTTTGAAATTTGGTCAAATTTAGGTTGAAACTTTAGAAGC  5489


Seq_1  5140  TCGTAGTTTTTGAAATGTTGACCCAAACGAGCTAAAACTTTCAGAAGTCACATAATTAGA  5199
             |||||||||| | |||||||||||||||||||||||||||||||||||||||||||||||
Seq_2  5490  TCGTAGTTTTCG-AATGTTGACCCAAACGAGCTAAAACTTTCAGAAGTCACATAATTAGA  5548


Seq_1  5200  TATTTATTATTTTCTGCATTCTTTTCAGATTTTTTATAAAATGTATAGCAAATTACTTTT  5259
             ||||||||||||||||||||||||||||||||||||||||||||||||||||||||||||
Seq_2  5549  TATTTATTATTTTCTGCATTCTTTTCAGATTTTTTATAAAATGTATAGCAAATTACTTTT  5608


Seq_1  5260  GTTGGAAACCGTCAGACCTAGAATAGATGGAGATACGAAGGGTAAGTTATGTTGTCATTT  5319
             ||||||||||||||||||||||||||||||||||||||||||||||||||||||||||||
Seq_2  5609  GTTGGAAACCGTCAGACCTAGAATAGATGGAGATACGAAGGGTAAGTTATGTTGTCATTT  5668


Seq_1  5320  GTCAAAGGCAAATAACGTAATTCGGCTCCCTCTTCCTCCCGCCGCGTCACCAATTTCTAT  5379
             ||||||||||||||||||||||||||||||||||||||||||||||||||||||||||||
Seq_2  5669  GTCAAAGGCAAATAACGTAATTCGGCTCCCTCTTCCTCCCGCCGCGTCACCAATTTCTAT  5728


Seq_1  5380  GTTTACTCACAGATCTCTCCTTCTCTATCCTCTTCGAAGCCTATTCCCCCCTTGCTCTTA  5439
             |||||||| ||||||||||||||||||||||||||||||||||||| |||||||||||||
Seq_2  5729  GTTTACTCGCAGATCTCTCCTTCTCTATCCTCTTCGAAGCCTATTCTCCCCTTGCTCTTA  5788


Seq_1  5440  TTTAGCTGCCAAACATCATGAAGCCACCAAAGGCCGTCTCTACCTCGGGTGTGGCTGTGC  5499
             ||| ||||||||||||||||||||||||||||||||||||||||||||||||||||||||
Seq_2  5789  TTTCGCTGCCAAACATCATGAAGCCACCAAAGGCCGTCTCTACCTCGGGTGTGGCTGTGC  5848


Seq_1  5500  ATAATCAAAGATCATCGTTACCGGTACCGATTGTTATCCAATAAACATTCTTTCCTTACA  5559
             |||||||||||||||||||||||||||||||||| |||||||||||||||||||||||||
Seq_2  5849  ATAATCAAAGATCATCGTTACCGGTACCGATTGTCATCCAATAAACATTCTTTCCTTACA  5908


Seq_1  5560  TCTTTGATCTCTCCTCCTTCCAAATTGATATTGGGCGAGCATTCACTATGCTGTGCAATC  5619
             ||||||||||||||||||||||||||||| ||||||||||||||||||||||||||||||
Seq_2  5909  TCTTTGATCTCTCCTCCTTCCAAATTGATGTTGGGCGAGCATTCACTATGCTGTGCAATC  5968


Seq_1  5620  GATTATTCGTGCAATTATCAAGTTATATCTTTCTAAACTCAAGGGGCGACGGTATTACCT  5679
             |||||||||||||||||||||||||||||||||||||||||| |||||||||||||| ||
Seq_2  5969  GATTATTCGTGCAATTATCAAGTTATATCTTTCTAAACTCAACGGGCGACGGTATTATCT  6028
```

```
Seq_1  5680  GCTCTCAAGTGAGTCCCAATGGGTGTCGCTGTCACTAGTTGTCCTCAGGTCAGCCTCCCT  5739
             ||||||||||||||||||||||||||||||||||||||||||||||||||||||||||||
Seq_2  6029  GCTCTCAAGTGAGTCCCAATGGGTGTCGCTGTCACTAGTTGTCCTCAGGTCAGCCTCCCT  6088

Seq_1  5740  TCCATTGCACATTTCCTCTTTCCTCCGAAATTAACAATATGTGAGAATTGCTGTACAAAA  5799
             ||||||||||||||||||||||||||||||||||||||||||||||||||||||||||||
Seq_2  6089  TCCATTGCACATTTCCTCTTTCCTCCGAAATTAACAATATGTGAGAATTGCTGTACAAAA  6148

Seq_1  5800  TAATAAACAGGTGAGCATTCCCTCTCTGTCCGGCCATTGGATTTATCAAAAAAAGATAAT  5859
             |||| |||||||||||||||||||||||||||||||||||||||||||||||||||||||
Seq_2  6149  TAATGAACAGGTGAGCATTCCCTCTCTGTCCGGCCATTGGATTTATCAAAAAAAGATAAT  6208

Seq_1  5860  CCTAGTTCAAATTTCGTTTCCATAAATTCAACCTTCTTTACCTTGAAATTTAAGCCCAGC  5919
             ||||||||||||||||||||||||||||||||||||||||||||||||||||||||||||
Seq_2  6209  CCTAGTTCAAATTTCGTTTCCATAAATTCAACCTTCTTTACCTTGAAATTTAAGCCCAGC  6268

Seq_1  5920  GGTCCCTTCCGGGACATCCGATAAAATTGAGATCTCGATAAAATTTTAACAGGGGCCTGA  5979
             ||||||      ||||||||||||||||||||||||||||||||||||||||||||||||
Seq_2  6269  GGTCCC------GACATCCGATAAAATTGAGATCTCGATAAAATTTTAACAGGGGCCTGA  6322

Seq_1  5980  CAGAGGTAGGCCGGAGGCCGTTGAGTTGGTGACTCCCGGGTTTCCCCAAGGAAATACCGA  6039
             ||||||||||||||||||||||||||||||||||||||||||||||||||||||||||||
Seq_2  6323  CAGAGGTAGGCCGGAGGCCGTTGAGTTGGTGACTCCCGGGTTTCCCCAAGGAAATACCGA  6382

Seq_1  6040  GATATTTGTTTGCCGTACCATACACTCCTCGCCGCACGCTGCGCCTGCGACGGCATAGTT  6099
             ||||||||||||||||||||||||||||||||||||||||||||||||||||||||||||
Seq_2  6383  GATATTTGTTTGCCGTACCATACACTCCTCGCCGCACGCTGCGCCTGCGACGGCATAGTT  6442

Seq_1  6100  CGCCGCCGGAAACGCCCGTCTCCCAACGCGCGACGGCGCGAGGCTACCTCCCGTTATCGA  6159
             ||||||||||||||||||||||||||||||||||||||||||||||||||||||||||||
Seq_2  6443  CGCCGCCGGAAACGCCCGTCTCCCAACGCGCGACGGCGCGAGGCTACCTCCCGTTATCGA  6502

Seq_1  6160  GGAAATCCATTCGCCGCAGCGGGCGCC  6186
             |||||||||||||||||||||||||||
Seq_2  6503  GGAAATCCATTCGCCGCAGCGGGCGCC  6529
```

BdindelWSU_6, downstream

>Bradi2g12420

CCAGAGGACCAGATGTTACATATGATGTGTACAAATTTATGGATAATATGATGTATTTTGCTTTTGATTTAGTTTCCCTTGCCTAGTTG
AGTAGTTTCTTACCAACCTTATACATCTTCCTGGCCTGCGAAGTCCTTGATATTGTTTGTTCAGCCGTCACCAAAAGAGGTTGGTAATG
AGTTCTGGCAGACACTTGAGGACCATGTAAATCACAGTACTCGGTATTGACTAGCTATTGATGGTCTTTTGAGGATTTTAACATTCCTA
GTGATAGCATTTTGTTGCCGTTTCTTTTAAGGAAATAACACTATGTTACTACTGAACCAGAGAACATTTTACTGGGAGTATATTGGAGT
GCAGTCCTAACCAACCTGTTTTGAAATCTTGTCATGTGTGCGTGGCCACCTTCAGGTTGTGCAGATATCGCTCCCTACATGTCGCGTCA
CATAGCAAAGGTTATGATTCTTTTGATGACAAGGATCCCAGAGTTTTGCACCAAAACCACCCTTATCCGTGGTAGACTTGGCTATTTTT
TGGCAGGGGTCGCTAGTTTGCGAGCACAGCGCGTCAGCGCCGATGGATCTGAATTCGTTTATGGACGGCGTTCCAATCGGTCGTCCT
GGATGCCACCGCGGTGAACGACATGAGTTTGGACTTATTGCTGCTGCTGTTGGTCAATCGATTGTGCTCATGCTGAGATCTTGCTCCA
GCGATCTACGGCTCTTATACCAGCATGGGACTCAAAAATCTCCTTACCTTGACGGCTTGGCAGTTTTTCTTTTTAGGAAAACGGCTTGG
CAGCTTTGGCCGGAAACTAACAAGCGTGTATGTCACACTCCGATATGCCGTGCAGCAGACATGTTCCAAAATCCAGATTATCAAGGG
TGAACTGTAGATATTTGAACCTTTCCAAAAGTACGTCCCCGTCTTTTTTTTCCCCCCTTTTCTTGAGATGTGATAATTACAGCGATTCTC
ACGTCCAGACGCCCGATTCCTAGTAAAGAGTTTTCGCACGCTGAGAGGAAAATCTTTTGGCTTTTCGGATTTTATTTCTGGTACAGTG
ACCAGCCAGCCCCCATGGTTGAATGGTTCTCAGCCAAGCGGGGCCTCCGGTCTCCGGCGTTATCTTCATTCGCCGCGTCGGACGTTAT

CCGTGTGAGCAACGGAGAGGACGATCCAGCAAACTCCACGCAACGTTTTTTCTCCCCTTGCACAAGTCCTTCTCTCGTAAATCCCGTA
CTGTATACTGCAAGGGCATCTTCAATGGCAAGCCGTTACTAATTTTTATCTGTTTTTTTTATTTTTTTTTAATGGACTCACATGTCATTTT
TTTTTTCATTCTTGGTATCCATGCACAAGCTGTTACTTCTGCAAGTAATTAGTACTAATTTTTTCTCTTCATCTCTCCTTCACGTAGGACT
AGTACCTGCTTAGTACCTTCTATTGGAAATGCCTAATAGATACTTCTTCGATGTTTTCCAAACGGAACGTGGGTCTCGTCGTTTCCGAA
CCACAGATTTCGTAAAGCGAAATCGTTTTGTATTCTGGTGCGACGTGATGTGGCACCAATAAGGTCCAATCCTAGAGCCAGGTTTCTC
TTTCATGATGTGTCCTCGTTGAGCTGTTTTTTTTTACTCCAAATTAATTGTCTTCTCGTCTCTCATCCACATGGTTTTTCCCATTGTTTTAC
TAGAACGGCATGCAAAGCAACAAAATCCATTCCGGAGAATCCTTCGTACATGGGAGTTTTTACTTGGTTCAGGTTATAGGCCAGGAG
AAAAAGTTCACGTTGGCCTCGTGGCGTCTGGGTCAGCTCAGACAGAGGAGGAGTAGCCATCAGTGCCAGCCAGGTCATCCCACGAG
TCTTATCCCCTTAATTCATTCGCTTTTCTCCTGTTTTGGCTTTTGGTTGGCGTGGTGCTTGCAAGATAGACCAATGCGCGTAAATTCAGA
TTTTTATTTCTTCTACTCCGTAAATAAAATGAAAGAGAAGAGACGTTTCATTTTTGTCCATTCGTGGTGCGTTCTCTGGCAAACAAGAT
CACTTTGGAATTGGGCATGTACAAGTGCATGACATCAGCTTTCCTATTTGCGTGTCTACCTATAAGATGGTATTTTAGATGTTTTCCAC
TTCTGCAGGCAAAACTGAAATAGATTTATTGTTTTCAAGAACAAGAATATCTTCTCACAGAATTTACGCAGGGACACGGGATTTAGAG
AAGTGTGTGCCTTCTCCCTGCTGCCATTGACAATTATTCGAGGATTAATTTGCAATATAAAGATTTCATTGAAAACAATTTCAGTTATT
GCATAAACGTGAACAAACATATTTTGCTCCAAGGGCACCTTGAAACTCTTAACCGTGTGATTAGATCCAGACTTGCAACCATACTGAG
ATCATTCAGCAGCAACCAGGCAGCTTCCTCATTTGACCATTCGCACGGTGTGGAATGGTGAATGTAGGAATATGTGCTACAGTAGTG
TTGGTACAAATTAATTAAGGCCAAAGGGAGACAACGGGATGCAGAGACGCGTGGTGAACACCGACCATATCCTTTCCTGATGCGCCT
CGCGGGCACCGACTAACGGGGCCCATGGGACAGCTTCCATTCCTCTCTTTTTTTACCACAATTGGTACAGTTTTTTTTTTAATACTCCCG
AGCAGACCAATGACTCCCTTTCGGATTAAACTTTGAAACGCTGGCTGGTTTGAACGATCTCCACGAACGCTGGACCCTGAGCCGTTCG
ATCAACATCCAACGCTAGGCTTCACGCTGGTTAAGAATTAACAGGGGTCAGGCCATTGGCGTGCGAGAGGTGGACGGTGCGCCCAT
GTGACCGTTCGTAAATGCTTCATACACAGAGAGATTATACAGACTGTTCACGGACATATGGGCCGAGCAAGATTCATATACACCTTTT
GGTCCACATGTCAGTGCGGGTGTTTTCCGGGCATGTGTCGGATATGTGGTGTGTGGACGACCCTACATGTTTTCGATTATGAGTTTAT
GACGACCCACCGGCACTGCACGGTGTTGCCGGTGTCCAGACTCCAGCGCTAGTTGTCTTGCCCGGGGTGTAGTCAAAGTCGGAAATC
TTACCCCCGATGAGTTTTAGAGAAAATTGCAAAGACATTGAATACAAAAGGAAATGGAAGAGAATTGAGATGAAAAAAGAGTTATG
GAGCTCATTACTTAGAGGAGAGGGAGCACTCTTTTTTTTACGGCAGGGGAGGAACACTATTTTTTTTAAGGCAGGAGAGGGAACACT
CGAATTCTCTTGGAAAGTCAAGAGTCTAACTTATTCTTTTTGGCCACTAATTAATTAAAACAATTTATGATGGTCAGATTAATGTCACC
ACTAAATTTGTCATCAGAATTGTTTTGCAATATTTTTTTACATTCACTTATATATGTATAAGAGAACTAATGATCATAGTTCCATACGTG
ACTCGATCTACGGATAATCAACAGCACATATGTTTTGAAACCGAAGGTGTACATGCCAATTCTTTTAGTAGTCTCACCGATCCATATTA
TTCGTCGCTGATTTGGTTACATGCTCCCTCCCATGCATATTATCTGTCGTTGATTTACTGGATAGTCACAAGTAATATGGATCGGATGT
TCTGCTACATCGATATATTAAGAGATATATCCATATTAGAGCTCTAAACTAACTCCAATACCGGTTAGAACACAACCCTCAATTCTTAA
TACTGTGTAAATTATCCCCTGACCTAGCCAGAAGGTGGTTTTCACATTCCCGGCAGATTTTGACCGAGTCAACACACATTTTTCAGTCC
AAAAAAGATAAAACGAAAAATCTTGTACACAACCTACTATTAAATTTGGGCTTCAGTGAGCAAACGATAAGTACAACAAATGACATAT
TTTGTTTGAGACTCAAATTTTGAATTCAAAATTGAATTTCCAAATTTTTAATTATTTATAGGAAATCATAAACGTTGTTGAGAGCCTACT
GTTAAAGTTTCAGCGTCTCGCATGTATGATGTGCCAGTCAACTTGGTCAAAACCTGCCACGTACGTGTAAACAATCTTTTGGCTGGAT
CAAGAATAATTTAAGCGGTATGAATAGTTGAAGGATGTGTTTTAATTAGTATCGGAGTTCGGTGTGTGTGAATTTAAACTATATGGTT
AGTTCAACGTTGTAATATGGACTTATCACATATATTAGTCTAGTTCGCATAATACATGATGCTGATTTTTCTAGTTTATCTCAACATACC
ATTATTATTTGTACTACGTGTGCTTGAATTTATTTGTATGCGTACAGAACATGTTTTGGTTAGAATGAGTTTCATTTTTAACCTGGCGTT
TGTGCGATTTGCTAGATCTGAAAATCTTAGCTGCACACATGTAAATGTTGACTACAAATAGTCGTTAAAATTTTTTGAATATGTAAAAA
ATTGCACAAGTTAGCTGAATTTAAAACTTATGAGGTAACAATATGAAGTGCAAACACCTTGGAAACCTAAAATTAAACCCACCTCTCT
ATGAACAGTCTACCAGCAAGGAAGAACAAAAATAGAAAGCACAACGCCGACAACGGTAAATTCAGAAGCCCCAAAAAAATCCAAAG
AAGGCCAGATTCGAAAATGCCCCGTTTAGGCCGAAGGGGCAGAGAAAAAAGAGATAAAAAAAGCATTGAAAACATGCACTTTAATA
TCGGGTGGGTCCCACCAACTACCCCCCGCACTCTATAAAGGTCGCCTCCCCTCAGATCTTCCCGTCCATTACGCCACCTCTCCACCTAA
ACCAAACCGTACCGAACCTTGCCATTTTGCAACTCACACCTCGCCGACCACGTATTTACTATAACCCGCCTTACCGTTGAGGCCTTTAC
TCTCCTTTTTTGCGCCTAGCCCCCTACAAAGTTTCCACATCTTGCGTCTGTGACCTTCACCGGAGTTGCCATATGAATGGTCAAACTACG
ATGGGGCTCGCCGCAGCCGCAGCCGCCGCCGTGAGGCCGTGCCGCCGCCGCCTACTCTCGTCCGTCTCAGCCGGGGGCGCGAAGGC
ATCCGTGACCCCGCTCTTCCCGAGACGCCCCCACCCGCAGCACCACCACCACGGCCGCCGCCTCCCCTTCCTCGCCTCGGCGGCGTCG
CAGACCTCGCACTCCGACCCGAGCTCCACCCCAACAACCACCCCCACCCCTGTAAACTCCGATCCCCGCTCCGCCGTCGCCGGGAACC
TCCCTTTCTTTGACCGCGTGCTCTTCCCGGGCTCCTTCCCTCCTGTCGAGACCCCGCCTGTGGAGGAGCCGGCGCCGCCGACCGATGA
GGGTCTGGCTTCGCCGGTGAGGGAGGAGTCGGAGACGGAGAGGGAGGCCTGGAGGCTACTGAGGAGGGCGGTGGTGAGCTACTG
CGGCGAACCGGTGGGCACGGTGGCGGCGGAGGACCCCGAGTGCACGGAGGTGGCCAACTACGACCAGGTCTTCATCAGGGACTTC

GTCCCCTCCGCGCTCGCCTTCCTCATGCGCGGAGAGACTGAAATCGTCCGCAATTTCCTCCTCCACACCTTGCAGCTGCAGGTGAGCG
CCTTGCCTATTGGGGATCCGGATTGCTTAGTTTGAGTTGAAAATTAGCGCGTTGTGACTACTAGTGTTGATTGCGTGCACGTCACTTG
CTAGTTGGGGGGAAGTTAACACCGATTTTGCGTTGTTTGAATGTGTAGTACATCAGAATTTTCGGCATTAGTAAACGTAACATACCAT
TCCTTAATGAATTTGCTCTCAGCATGTGAATTTTTTTCTTCTAATTAATTCCTGTTCTAATTGGCTTGTATTGTATTCTATGGGTGAAATA
GAGATAAGATTTCACTTTTGGAGTCTAATATGTCTCTTTCATACATGTACTAAGCAGTACTTGTTTGGTAGCGATTATACATCCTCATA
GCTTAGCCTAAATTAGCCTAAACGGCAATTATGAGGTAACAATAGTTATCTGTATGCAATACTACCACACTGAGTAAACTTCACTTTTT
TATACGATTCGAAAAGCCTAGAACAGACGAGAAAAATCTACAACTTTTGTCCAAATTTCCTTAAGATGTGATCACATTTACCATAAAAC
GCCACTAAACTGGCTTGCTAGTAATTGTTTGTGAACTTTAGTTTCTTATCTGGCACTTGAATCAAATTTTCTTCATTTACTGATCTTTCTC
ATTTATTTGATGATCAGAGCTGGGAGAAAACTGTTGACTGTTACAGCCCTGGGCAAGGCTTGATGCCAGCCAGCTTTAAGATTAGGA
CTGTTCCTCTTGACGAAAACAACGAAGCATTTGAGGAGGTTTTGGACCCTGACTTTGGTGAATCCGCTATTGGCCGTGTAGCTCCAGT
TGATTC

>BdiBd21-3.2G0165900

CCAGAGGACCAGATGTTACATATGATGTGTACAAATTTATGGATAATATGATGTATTTTGCTTTTGATTTAGTTTCCCTTGCCTAGTTG
AGTAGTTTCTTACCAACCTTATACATCTTCCTGGCCTGCGAAGTCCTTGATATTGTTTGTTCAGCCGTCACCAAAAGAGGTTGGTAATG
AGTTCTGGCAGACACTTGAGGACCATGTAAATCACAGTACTCGGTATTGACTAGCTATTGATGGTCTTTTGAGGATTTTAACATTCCTA
GTGATAGCATTTTGTTGCCGTTTCTTTTAAGGAAATAACACTATGTTACTACTGAACCAGAGAACATTTTACTGGGAGTATATTGGAGT
GCAGTCCCAACCAACCTGTTTTGAAATCTTGTCATGTGTGCGTGGCCACCTTCAGGTTGTGCAGATATCGCTCCCTACATGTCGCGGC
ACATAGCAAAGGTTATGATTCTTTTGATGACAAGGATCCCAGAGTTTTGCAACAAAACCGTCCTTATCCGTGGTAGACTTGGCTATTTT
TTGGCAGGGGTCGCTAGTTTGCGAGCACAGCGCGTCAGCGCCGATGGATCTGAATTCGTTTATGGACGGCGTTCCAATCGGTCGTCC
TGGATGCCACCGCGGTGAACGACATCAGTTTGGACTTATTGCTGCTGCTGTTGGTCAATCGATTGTGCTCATGCTGAGATCTTGCTCC
AGCGATCTACGGCTCTTATACCAGCATGGGACTCAAAAATCTCCTTACCTTGACGGCTTGGCAGTTTTTCTTTTTAGGAAAACGGCTTG
GCAGCTTTGGCCGGAAACTAACAAGCGTGTATGTCACGCCCCGATATGCCGTGCAGCAGACATGTTCCAAAATCCAGATTATCAAGG
GTGAACTGTAGATATTTGAACCTTTCCAAAAGTACGTCCCCGTCTTTTTTTTCCTCCCCTTTTCTTGAGATGTGATAATTACAGCGATTC
TCACGTCCAGACGCCCGATTCCTAGTAAAGAGTTTTCGCACGCTGAGAGGAAAATCTTTTGGCTTTTCGGAGGAAAACTCTTGTTTGA
ATTGTGGATTTTATTTCTGGTACAGTGACCAGCCAGCCCCCATGGTTGAATGGTTCTCAGCCAAGCGGGGCCTCCGGTCTCCGGCGTT
ATCTTCATTCGCGGCGTCGGACGTTATCCGTGTGAGCAACGGAGACGACGATCCAGCAAACTCCACGCAACGTTTTTTCTCCCCTTGC
ACAAGTCCTTCTCTCCTAAATCCCGTACTGTATACTGTAACAGATACTTCTTCGATGTTTTCCAAACGGAACGTGGGTCTCGTCGTTTCC
GAACCACAGATTTCGTAAAGCGAAATCGTTTTGTATTCTGGTGCGACGTGATGTGGCACCAATAAGGTCCAATACACCAATAAGAGC
CAGGTTTCTCTTTCATGATGTGTCCTCGTTGAGCTGTTTTTTTTACTCCAAATTAATTGTCTTCTCGTCTCTCATCCACATGGTTTTCCCA
TTGTTTTACTAGAACGGCATGCAAAGCAACAAAATCCATTCCGGAGAATCCTTCGTACATGGGAGTTTTTACTTGGTTCAGGTTATAG
GCCAGGAGAAAAAGTTCACGTTGGCCTCGTGGCGTCTGGGTCAGCTCAGACAGAGGAGGAGTAGCCATCAGTGCCGAAAAGGTAC
GCAAAATCTCAGCCAGGTCATCCCACGAGTCTTATCCCCTTAATTCATTCGCTTTTCTCCTCTTTTGGCTTTTGGTTGGCGTGGTGCTTG
CAAGATAGACCAATGCGCGTAAATTCAGATTTTTATTTCTTCTACTCCGTAAATTAAATAAAATGAAAGAGAAGAGACGTTTCATTTTT
GTCCATTCGTGGTGCGTTCTCTGGCAAACAAGATCACTTTGGAATTGGGCATGTACAAGTGCATGACATCAGCTTTCCTATTTGCGTG
TCTACCTATAAGATGGTATTTTAGATGTTTTCCACTTCTGCAGGCAAAACTGAAATAGATTTATTGTTTTCAAGAACAAGAATATCTTCT
CACAGAATTTACGCAGGGACACGGGATTTAGAGAAGTGTGTGCCTTCTCCCTGCTGCCATTGACAATTATTCGAGGATTAATTTGCAA
TATAAAGATTTCATTGAAAACAATTTCAGTTATTGCATAAACGTGAACAAACATATTTTGCTCCAAGGGCACCTTGAAACTCTTAACCG
TGTGATTAGATCCAGACTTGCAACCATACTGAGATCATTCAGCAGCAACCAGGCAGCTTCCTCATTTGACCATTCGCACGGTGTGGAA
TGGTGAATGTAGGAATATGTGCTACAGTAGTGTTGGTACAAATTAATTAAGGCCAAAGGGAGACAACGGGATGCAGAGACGCGTGG
TGAACACCGACCATATCCTTTCCTGATGCGCCTCGCGGGCACCGACTAACGGGGCCCATGGGACAGCTTCCATTCCTCTCTTTTTTTAC
CACAATTGGTACAGTTTTCTTTTTAATACTCCCGAGCAGACCAATGACTCCCTTTCGGATTAAACTTTGAAACGCTGGCTGGTTTGAAC
GATCTCCACGAACGCTGGACCCTGAGCCGTTCGATCAACATCCAACGCTAGGCTTCACGCTGGTTAAGAATTAACAGGGGTCAGGCC
ATTGGCGTGCGAGAGGTGGACGGTGCGCCCATGTGACCGTTCGTAAATGCTTCATACACAGAGAGATTATACAGACTGTTCACGGAC
ATATGGGCCGAGCAAGATTCATATACACCTTTTAGTCCACATGTCAGTGCGGGTGTTTTCCGGGCATGCGTCGGATATGTGGTGCGT
GGACGACCCTACATGTTTTCGATTATGAGTTTATGACGATCCACCGGCACTGCACGGTGTTGCCGGTGTCCAGGTTCCAGCGCTAGTT
GTCTTGCCCGGGGTGTAGTCAAAGTCGGAAATCTTACCCCCGATGAGTTTTAGAGAAAATTGCAAAGACATTGAATACAAAAGGAAA
TGGAGGAGAATTGAGATGAAAAAAGAGTTATGGAGCTCATTACTTAGAGGAGAGGGAGCACTCTTTTTTTTACGGCAGGGGAGGAA
CACTATTTTTTTTACGGTAGGAGAGGGCACACTCGAATTCTCTTGGAAAGTCAAGAGTCTAACTTATTCTTTTTGGCCACTAATTAATT

AAAACAATTTATGATGGTCAGATTAATGTCACCACTAAATTTGTCATCAGAATTGTTTTGCAATATTTTTTTACATTCACTTATATATGT
ATAAGAGAACTAATGATCATAGTTCCATACGTGACTCGATCTACGGATAATCAACAGCACATATGTTTTGAAACGAAGGTGTACATGC
CAATTCTTTTAGTAGTCTCACCGATCCATATTATTCGTCGCTGATTTGGTTACATGCTCCCTCCCATGCATATTATCTGTCGTTGATTTAC
TGGATAGTCACAAGTAATATGGATCGGATGTTCTGCTACATCGATATATTAAGAGATATATCCATATTAGAGCTCTAAACTAACTCCA
ATACCGGTTAGAACACAACCCTCAATTCTTAATACTGTGTAAATTATCCCCTGACCTAGCCAGAAGGTGGTTTTCACATTCCCGGCAGA
TTTTGACCGAGTCAACACACATTTTTCAGTCCAAAAAAGATAAAACGAAAAATCTTGTACACAACCTACTATTAAATTTGGGCTTCAGT
GAGCAAACGATAAGTACAACAAATGACATATTTTGTTTGAGACTCAAATTTTGAATTCAAAATTGAATTTCCAAATTTTTAATTATTTAT
AGGAAATCATAAACGTTGTTGAGAGCCTACTGTTAAAGTTTCAGCGTCTCGCATGTATGATGTGCCAGTCAACTTGGTCAAAACCTGC
CACGTACGTGTAAACAATCTTTTGGCTGGATCAAGGATAATTTAAGCGGTATGAATAGTTGAAGGATGTGTTTTAATTAGTATCGGAG
TTCGGTGTGTGTGAATTTAAACTATGTGGTTAGTTCAACGTTGTAATATGGACTTATCACATATATTAGTCTAGTTCGCATAATACATG
ATGCTGATTTTTCTAGTTTATCTCAACATACCATTATTATTTGTACTACGTGTGCTTGAATTTATTTGTATGCGTACGGAACATGTTTTG
GTTAAAATGAGTTTCATTTTTAACCTGGCGTTTGTGCGATTTGCTAGATCTGAAAATCTTAGCTGCACACATGTAAATGTTGACTACAA
ATAGTCGTTAAAATTTTTTGAATATGTAAAAAATTGCACAAGTTAGCTGAATTTAAAACTTATGAGGTAACAATATGAAGTGCAAACA
CCTTGGAAACCTAAAATTAAACCCACCTCTCTATGAACAGTCTACCAGCAAGGAAGAACAAAAATAGAAAGCACAACGCCGACAACG
GTAAATTCAGAAGCCCCAAAAAAATCCAAAGAAGGCCAGATTCGAAAATGCCCCGTTTAGGCCGAAGGGGCAGAGAAAAAAGAGAT
AAAAAAAGCATCGAAAACATGCACTTTAATATCGGGTGGGTCCCACCAACTACCCCCCGCACTCTATAAAGGTCGCCTCCCCTCAGAT
CTTCCCGTCCATTACGCCACCTCTCCACCTAAACCAAACCGTACCGAACCTTGCCATTTTGCAACTCACACCTCGCCGACCACGTATTTA
CTATAACCCGCCTTACCGTTTAGGCCTTTACTCTCCTTTTTTGCGCCTAGCCCCCTACAAAGTTTCCACATCTTGCGTCTGTGACCTTCAC
CGGAGTTGCCATATGAATGGTCAAACTACGATGGGGCTCGCCGCAGCCGCAGCCGCCGCCGTGAGGCCGTGCCGCCGCCGCCTACT
CTCGTCCGTCTCAGCCGGGGGCGCGAAGGCATCCGCGACCCCGCTCTTCCCGAGACGCCCCCACCCGCAGCACCACCACCACGGCCG
CCGCCTCCCCTTCCTCGCCTCGGCGGCGTCGCAGACCTCGCACTCCGACCCGAGCTCCACCCCAACAACCACCCCCACCCCTGTAAACT
CCGATCCCCGCTCCGCCGTCGCCGGGAACCTCCCTTTCTTTGACCGCGTGCTCTTCCCGGGCTCCTTCCCTCCTGTCGAGACCCCGCCT
GTGGAGGAGCCGGCGCCGCCGACCGATGAGGGTCTGGCTTCGCCGGTGAGGGAGGAGTCGGAGACGGAGAGGGAGGCCTGGAG
GCTACTGAGGAGGGCGGTGGTGAGCTACTGCGGCGAGCCGGTGGGCACGGTGGCGGCGGAGGACCCCGAGTGCACGGAGGTGGC
CAACTACGACCAGGTCTTCATCAGGGACTTCGTCCCCTCCGCGCTCGCCTTCCTCATGCGCGGAGAGACTGAAATCGTCCGCAATTTC
CTCCTCCACACCTTGCAGCTGCAGGTGAGCGCCTTGCCTATTGGGGATCCGGATTGCTTAGTTTGAGTTGAAAATTAGCGCGTTGTGA
CTACTAGTGTTGATTGCGTGCACGTCATTTGCTAGTTGGGGGGAAGTTAACACCGATTTTGCGTTGTTTGAATGTGTAGTACATCAGA
ATTTTCGGCATTAGTAAACGTAACATACCATTCCTTAATGAATTTGCTCTCAGCATGTGAATTTTTTTCTTCTAATTAATTCCTGTTCTAA
TTGGCTTGTATTGTATTCTATGGGTGAAATAGAGATAAGATTTCACTTTTGGAGTCTAATATGTCTCTTTCATACATGTACTAAGCAGT
ACTTGTTTGGTAGCGATTATACATCCTCATAGCTTAGCCTAAATTAGCCTAAACGGCAATTATGAGGTAACAATAGTTATCTGTATGCA
ATACTACCACAGAGTAAACTTCACTTTTTTATACGATTCGAAAAGCCTAGAACAGACGAGAAAAATCTACAACTTTTGTCCAAATTTCC
TTAAGATGTGATCACATTTACCATAAAACGCCACTAAACTGGCTTGCTAGTAATTGTTTGTGAACTTTAGTTTCTTATCTGGCACTTGA
ATCAAATTTTCTTCATTTACTGATCTTTCTCATTTATTTGATGATCAGAGCTGGGAGAAAACTGTTGACTGTTACAGCCCTGGGCAAGG
CTTGATGCCAGCCAGCTTTAAGATTAGGACTGTTCCTCTTGACGAAAACAACGAAGCATTTGAGGAGGTTTTGGACCCTGACTTTGGT
GAATCCGCTATTGGCCGTGTAGCTCCAGTTGATTCTGGTATATTTCCCTCCCCGCCTCTCGAACATAATCCCTTTCCTTTTGAAAATTAG
GTGTTAGTATTGAATGACATCAAGTTAGTAATTTGAAATGTTTATCCTTCTTCTTTAACCTAACCTAACCTAACCTAACCGTTTGCAGGA
CTTTGGTGGATTATCTTACTCAGAGCATACTGCAAGATTACAGGGGACTATTCTTTGCAAGAAAGAGTGGATGTCCAA

```
Alignment of Sequence_1:  [Untitled Sequence #1] with  Sequence_2: [Sequence Window #2]

Similarity : 6126/6440 (95.12 %)

Seq_1  1      CCAGAGGACCAGATGTTACATATGATGTGTACAAATTTATGGATAATATGATGTATTTTG  60
               ||||||||||||||||||||||||||||||||||||||||||||||||||||||||||||
Seq_2  1      CCAGAGGACCAGATGTTACATATGATGTGTACAAATTTATGGATAATATGATGTATTTTG  60


Seq_1  61     CTTTTGATTTAGTTTCCCTTGCCTAGTTGAGTAGTTTCTTACCAACCTTATACATCTTCC  120
               ||||||||||||||||||||||||||||||||||||||||||||||||||||||||||||
Seq_2  61     CTTTTGATTTAGTTTCCCTTGCCTAGTTGAGTAGTTTCTTACCAACCTTATACATCTTCC  120


Seq_1  121    TGGCCTGCGAAGTCCTTGATATTGTTTGTTCAGCCGTCACCAAAAGAGGTTGGTAATGAG  180
               ||||||||||||||||||||||||||||||||||||||||||||||||||||||||||||
Seq_2  121    TGGCCTGCGAAGTCCTTGATATTGTTTGTTCAGCCGTCACCAAAAGAGGTTGGTAATGAG  180
```

```
Seq_1  181   TTCTGGCAGACACTTGAGGACCATGTAAATCACAGTACTCGGTATTGACTAGCTATTGAT  240
             ||||||||||||||||||||||||||||||||||||||||||||||||||||||||||||
Seq_2  181   TTCTGGCAGACACTTGAGGACCATGTAAATCACAGTACTCGGTATTGACTAGCTATTGAT  240

Seq_1  241   GGTCTTTTGAGGATTTTAACATTCCTAGTGATAGCATTTTGTTGCCGTTTCTTTTAAGGA  300
             ||||||||||||||||||||||||||||||||||||||||||||||||||||||||||||
Seq_2  241   GGTCTTTTGAGGATTTTAACATTCCTAGTGATAGCATTTTGTTGCCGTTTCTTTTAAGGA  300

Seq_1  301   AATAACACTATGTTACTACTGAACCAGAGAACATTTTACTGGGAGTATATTGGAGTGCAG  360
             ||||||||||||||||||||||||||||||||||||||||||||||||||||||||||||
Seq_2  301   AATAACACTATGTTACTACTGAACCAGAGAACATTTTACTGGGAGTATATTGGAGTGCAG  360

Seq_1  361   TCCTAACCAACCTGTTTTGAAATCTTGTCATGTGTGCGTGGCCACCTTCAGGTTGTGCAG  420
             ||| ||||||||||||||||||||||||||||||||||||||||||||||||||||||||
Seq_2  361   TCCCAACCAACCTGTTTTGAAATCTTGTCATGTGTGCGTGGCCACCTTCAGGTTGTGCAG  420

Seq_1  421   ATATCGCTCCCTACATGTCGCGTCACATAGCAAAGGTTATGATTCTTTTGATGACAAGGA  480
             |||||||||||||||||||||| |||||||||||||||||||||||||||||||||||||
Seq_2  421   ATATCGCTCCCTACATGTCGCGGCACATAGCAAAGGTTATGATTCTTTTGATGACAAGGA  480

Seq_1  481   TCCCAGAGTTTTGCACCAAAACCACCCTTATCCGTGGTAGACTTGGCTATTTTTTGGCAG  540
             ||||||||||||||| |||||||  |||||||||||||||||||||||||||||||||||
Seq_2  481   TCCCAGAGTTTTGCAACAAAACCGTCCTTATCCGTGGTAGACTTGGCTATTTTTTGGCAG  540

Seq_1  541   GGGTCGCTAGTTTGCGAGCACAGCGCGTCAGCGCCGATGGATCTGAATTCGTTTATGGAC  600
             ||||||||||||||||||||||||||||||||||||||||||||||||||||||||||||
Seq_2  541   GGGTCGCTAGTTTGCGAGCACAGCGCGTCAGCGCCGATGGATCTGAATTCGTTTATGGAC  600

Seq_1  601   GGCGTTCCAATCGGTCGTCCTGGATGCCACCGCGGTGAACGACATGAGTTTGGACTTATT  660
             ||||||||||||||||||||||||||||||||||||||||||||| ||||||||||||||
Seq_2  601   GGCGTTCCAATCGGTCGTCCTGGATGCCACCGCGGTGAACGACATCAGTTTGGACTTATT  660

Seq_1  661   GCTGCTGCTGTTGGTCAATCGATTGTGCTCATGCTGAGATCTTGCTCCAGCGATCTACGG  720
             ||||||||||||||||||||||||||||||||||||||||||||||||||||||||||||
Seq_2  661   GCTGCTGCTGTTGGTCAATCGATTGTGCTCATGCTGAGATCTTGCTCCAGCGATCTACGG  720

Seq_1  721   CTCTTATACCAGCATGGGACTCAAAAATCTCCTTACCTTGACGGCTTGGCAGTTTTTCTT  780
             ||||||||||||||||||||||||||||||||||||||||||||||||||||||||||||
Seq_2  721   CTCTTATACCAGCATGGGACTCAAAAATCTCCTTACCTTGACGGCTTGGCAGTTTTTCTT  780

Seq_1  781   TTTAGGAAAACGGCTTGGCAGCTTTGGCCGGAAACTAACAAGCGTGTATGTCACACTCCG  840
             |||||||||||||||||||||||||||||||||||||||||||||||||||||| | |||
Seq_2  781   TTTAGGAAAACGGCTTGGCAGCTTTGGCCGGAAACTAACAAGCGTGTATGTCACGCCCCG  840

Seq_1  841   ATATGCCGTGCAGCAGACATGTTCCAAAATCCAGATTATCAAGGGTGAACTGTAGATATT  900
             ||||||||||||||||||||||||||||||||||||||||||||||||||||||||||||
Seq_2  841   ATATGCCGTGCAGCAGACATGTTCCAAAATCCAGATTATCAAGGGTGAACTGTAGATATT  900

Seq_1  901   TGAACCTTTCCAAAAGTACGTCCCCGTCTTTTTTTTCC-CCCCTTTTCTTGAGATGTGAT  959
             |||||||||||||||||||||||||||||||||||||| |||||||||||||||||||||
Seq_2  901   TGAACCTTTCCAAAAGTACGTCCCCGTCTTTTTTTTCCTCCCCTTTTCTTGAGATGTGAT  960

Seq_1  960   AATTACAGCGATTCTCACGTCCAGACGCCCGATTCCTAGTAAAGAGTTTTCGCACGCTGA  1019
             ||||||||||||||||||||||||||||||||||||||||||||||||||||||||||||
Seq_2  961   AATTACAGCGATTCTCACGTCCAGACGCCCGATTCCTAGTAAAGAGTTTTCGCACGCTGA  1020
```

```
Seq_1  1020  GAGGAAAATCTTTTGGCTTTTCGGA-------------------------TTTTATTTCT  1054
             |||||||||||||||||||||||||                         ||||||||||
Seq_2  1021  GAGGAAAATCTTTTGGCTTTTCGGAGGAAAACTCTTGTTTGAATTGTGGATTTTATTTCT  1080

Seq_1  1055  GGTACAGTGACCAGCCAGCCCCCATGGTTGAATGGTTCTCAGCCAAGCGGGGCCTCCGGT  1114
             ||||||||||||||||||||||||||||||||||||||||||||||||||||||||||||
Seq_2  1081  GGTACAGTGACCAGCCAGCCCCCATGGTTGAATGGTTCTCAGCCAAGCGGGGCCTCCGGT  1140

Seq_1  1115  CTCCGGCGTTATCTTCATTCGCCGCGTCGGACGTTATCCGTGTGAGCAACGGAGAGGACG  1174
             |||||||||||||||||||||| |||||||||||||||||||||||||||||||| ||||
Seq_2  1141  CTCCGGCGTTATCTTCATTCGCGGCGTCGGACGTTATCCGTGTGAGCAACGGAGACGACG  1200

Seq_1  1175  ATCCAGCAAACTCCACGCAACGTTTTTTCTCCCCTTGCACAAGTCCTTCTCTCGTAAATC  1234
             ||||||||||||||||||||||||||||||||||||||||||||||||||||| ||||||
Seq_2  1201  ATCCAGCAAACTCCACGCAACGTTTTTTCTCCCCTTGCACAAGTCCTTCTCTCCTAAATC  1260

Seq_1  1235  CCGTACTGTATACTGCAAGGGCATCTTCAATGGCAAGCCGTTACTAATTTTTATCTGTTT  1294
             |||||||||||||||                             |||
Seq_2  1261  CCGTACTGTATACTG-----------------------------TAAC------------  1279

Seq_1  1295  TTTTTATTTTTTTTTAATGGACTCACATGTCATTTTTTTTTTCATTCTTGGTATCCATGC  1354

Seq_2  1280  ------------------------------------------------------------  1279

Seq_1  1355  ACAAGCTGTTACTTCTGCAAGTAATTAGTACTAATTTTTTCTCTTCATCTCTCCTTCACG  1414

Seq_2  1280  ------------------------------------------------------------  1279

Seq_1  1415  TAGGACTAGTACCTGCTTAGTACCTTCTATTGGAAATGCCTAATAGATACTTCTTCGATG  1474
                                                         ||||||||||||||||
Seq_2  1280  --------------------------------------------AGATACTTCTTCGATG  1295

Seq_1  1475  TTTTCCAAACGGAACGTGGGTCTCGTCGTTTCCGAACCACAGATTTCGTAAAGCGAAATC  1534
             ||||||||||||||||||||||||||||||||||||||||||||||||||||||||||||
Seq_2  1296  TTTTCCAAACGGAACGTGGGTCTCGTCGTTTCCGAACCACAGATTTCGTAAAGCGAAATC  1355

Seq_1  1535  GTTTTGTATTCTGGTGCGACGTGATGTGGCACCAATAAGGTCCAAT---CCT---AGAGC  1588
             ||||||||||||||||||||||||||||||||||||||||||||||   ||    |||||
Seq_2  1356  GTTTTGTATTCTGGTGCGACGTGATGTGGCACCAATAAGGTCCAATACACCAATAAGAGC  1415

Seq_1  1589  CAGGTTTCTCTTTCATGATGTGTCCTCGTTGAGCTGTTTTTTTTTACTCCAAATTAATTG  1648
             |||||||||||||||||||||||||||||||||||||||||||| |||||||||||||||
Seq_2  1416  CAGGTTTCTCTTTCATGATGTGTCCTCGTTGAGCTGTTTTTTTT-ACTCCAAATTAATTG  1474

Seq_1  1649  TCTTCTCGTCTCTCATCCACATGGTTTTTCCCATTGTTTTACTAGAACGGCATGCAAAGC  1708
             ||||||||||||||||||||||||||||||||||||||||||||||||||||||||||||
Seq_2  1475  TCTTCTCGTCTCTCATCCACATGGTTTTTCCCATTGTTTTACTAGAACGGCATGCAAAGC  1534

Seq_1  1709  AACAAAATCCATTCCGGAGAATCCTTCGTACATGGGAGTTTTTACTTGGTTCAGGTTATA  1768
             ||||||||||||||||||||||||||||||||||||||||||||||||||||||||||||
Seq_2  1535  AACAAAATCCATTCCGGAGAATCCTTCGTACATGGGAGTTTTTACTTGGTTCAGGTTATA  1594

Seq_1  1769  GGCCAGGAGAAAAAGTTCACGTTGGCCTCGTGGCGTCTGGGTCAGCTCAGACAGAGGAGG  1828
             ||||||||||||||||||||||||||||||||||||||||||||||||||||||||||||
Seq_2  1595  GGCCAGGAGAAAAAGTTCACGTTGGCCTCGTGGCGTCTGGGTCAGCTCAGACAGAGGAGG  1654
```

```
Seq_1  1829  AGTAGCCATCAGTGCC--------------------AGCCAGGTCATCCCACGAGTCTTA  1868
             ||||||||||||||||                    ||||||||||||||||||||||||
Seq_2  1655  AGTAGCCATCAGTGCCGAAAAGGTACGCAAAATCTCAGCCAGGTCATCCCACGAGTCTTA  1714

Seq_1  1869  TCCCCTTAATTCATTCGCTTTTCTCCTGTTTTGGCTTTTGGTTGGCGTGGTGCTTGCAAG  1928
             ||||||||||||||||||||||||||| ||||||||||||||||||||||||||||||||
Seq_2  1715  TCCCCTTAATTCATTCGCTTTTCTCCTCTTTTGGCTTTTGGTTGGCGTGGTGCTTGCAAG  1774

Seq_1  1929  ATAGACCAATGCGCGTAAATTCAGATTTTTATTTCTTCTACTCCGTAAAT-----AAAAT  1983
             ||||||||||||||||||||||||||||||||||||||||||||||||||     |||||
Seq_2  1775  ATAGACCAATGCGCGTAAATTCAGATTTTTATTTCTTCTACTCCGTAAATTAAATAAAAT  1834

Seq_1  1984  GAAAGAGAAGAGACGTTTCATTTTTGTCCATTCGTGGTGCGTTCTCTGGCAAACAAGATC  2043
             ||||||||||||||||||||||||||||||||||||||||||||||||||||||||||||
Seq_2  1835  GAAAGAGAAGAGACGTTTCATTTTTGTCCATTCGTGGTGCGTTCTCTGGCAAACAAGATC  1894

Seq_1  2044  ACTTTGGAATTGGGCATGTACAAGTGCATGACATCAGCTTTCCTATTTGCGTGTCTACCT  2103
             ||||||||||||||||||||||||||||||||||||||||||||||||||||||||||||
Seq_2  1895  ACTTTGGAATTGGGCATGTACAAGTGCATGACATCAGCTTTCCTATTTGCGTGTCTACCT  1954

Seq_1  2104  ATAAGATGGTATTTTAGATGTTTTCCACTTCTGCAGGCAAAACTGAAATAGATTTATTGT  2163
             ||||||||||||||||||||||||||||||||||||||||||||||||||||||||||||
Seq_2  1955  ATAAGATGGTATTTTAGATGTTTTCCACTTCTGCAGGCAAAACTGAAATAGATTTATTGT  2014

Seq_1  2164  TTTCAAGAACAAGAATATCTTCTCACAGAATTTACGCAGGGACACGGGATTTAGAGAAGT  2223
             ||||||||||||||||||||||||||||||||||||||||||||||||||||||||||||
Seq_2  2015  TTTCAAGAACAAGAATATCTTCTCACAGAATTTACGCAGGGACACGGGATTTAGAGAAGT  2074

Seq_1  2224  GTGTGCCTTCTCCCTGCTGCCATTGACAATTATTCGAGGATTAATTTGCAATATAAAGAT  2283
             ||||||||||||||||||||||||||||||||||||||||||||||||||||||||||||
Seq_2  2075  GTGTGCCTTCTCCCTGCTGCCATTGACAATTATTCGAGGATTAATTTGCAATATAAAGAT  2134

Seq_1  2284  TTCATTGAAAACAATTTCAGTTATTGCATAAACGTGAACAAACATATTTTGCTCCAAGGG  2343
             ||||||||||||||||||||||||||||||||||||||||||||||||||||||||||||
Seq_2  2135  TTCATTGAAAACAATTTCAGTTATTGCATAAACGTGAACAAACATATTTTGCTCCAAGGG  2194

Seq_1  2344  CACCTTGAAACTCTTAACCGTGTGATTAGATCCAGACTTGCAACCATACTGAGATCATTC  2403
             ||||||||||||||||||||||||||||||||||||||||||||||||||||||||||||
Seq_2  2195  CACCTTGAAACTCTTAACCGTGTGATTAGATCCAGACTTGCAACCATACTGAGATCATTC  2254

Seq_1  2404  AGCAGCAACCAGGCAGCTTCCTCATTTGACCATTCGCACGGTGTGGAATGGTGAATGTAG  2463
             ||||||||||||||||||||||||||||||||||||||||||||||||||||||||||||
Seq_2  2255  AGCAGCAACCAGGCAGCTTCCTCATTTGACCATTCGCACGGTGTGGAATGGTGAATGTAG  2314

Seq_1  2464  GAATATGTGCTACAGTAGTGTTGGTACAAATTAATTAAGGCCAAAGGGAGACAACGGGAT  2523
             ||||||||||||||||||||||||||||||||||||||||||||||||||||||||||||
Seq_2  2315  GAATATGTGCTACAGTAGTGTTGGTACAAATTAATTAAGGCCAAAGGGAGACAACGGGAT  2374

Seq_1  2524  GCAGAGACGCGTGGTGAACACCGACCATATCCTTTCCTGATGCGCCTCGCGGGCACCGAC  2583
             ||||||||||||||||||||||||||||||||||||||||||||||||||||||||||||
Seq_2  2375  GCAGAGACGCGTGGTGAACACCGACCATATCCTTTCCTGATGCGCCTCGCGGGCACCGAC  2434

Seq_1  2584  TAACGGGGCCCATGGGACAGCTTCCATTCCTCTCTTTTTTTACCACAATTGGTACAGTTT  2643
             ||||||||||||||||||||||||||||||||||||||||||||||||||||||||||||
Seq_2  2435  TAACGGGGCCCATGGGACAGCTTCCATTCCTCTCTTTTTTTACCACAATTGGTACAGTTT  2494
```

```
Seq_1  2644  TTTTTTTAATACTCCCGAGCAGACCAATGACTCCCTTTCGGATTAAACTTTGAAACGCTG  2703
             | ||||||||||||||||||||||||||||||||||||||||||||||||||||||||||
Seq_2  2495  TCTTTTTAATACTCCCGAGCAGACCAATGACTCCCTTTCGGATTAAACTTTGAAACGCTG  2554

Seq_1  2704  GCTGGTTTGAACGATCTCCACGAACGCTGGACCCTGAGCCGTTCGATCAACATCCAACGC  2763
             ||||||||||||||||||||||||||||||||||||||||||||||||||||||||||||
Seq_2  2555  GCTGGTTTGAACGATCTCCACGAACGCTGGACCCTGAGCCGTTCGATCAACATCCAACGC  2614

Seq_1  2764  TAGGCTTCACGCTGGTTAAGAATTAACAGGGGTCAGGCCATTGGCGTGCGAGAGGTGGAC  2823
             ||||||||||||||||||||||||||||||||||||||||||||||||||||||||||||
Seq_2  2615  TAGGCTTCACGCTGGTTAAGAATTAACAGGGGTCAGGCCATTGGCGTGCGAGAGGTGGAC  2674

Seq_1  2824  GGTGCGCCCATGTGACCGTTCGTAAATGCTTCATACACAGAGAGATTATACAGACTGTTC  2883
             ||||||||||||||||||||||||||||||||||||||||||||||||||||||||||||
Seq_2  2675  GGTGCGCCCATGTGACCGTTCGTAAATGCTTCATACACAGAGAGATTATACAGACTGTTC  2734

Seq_1  2884  ACGGACATATGGGCCGAGCAAGATTCATATACACCTTTTGGTCCACATGTCAGTGCGGGT  2943
             |||||||||||||||||||||||||||||||||||||||| |||||||||||||||||||
Seq_2  2735  ACGGACATATGGGCCGAGCAAGATTCATATACACCTTTTAGTCCACATGTCAGTGCGGGT  2794

Seq_1  2944  GTTTTCCGGGCATGTGTCGGATATGTGGTGTGTGGACGACCCTACATGTTTTCGATTATG  3003
             |||||||||||||| ||||||||||||||| |||||||||||||||||||||||||||||
Seq_2  2795  GTTTTCCGGGCATGCGTCGGATATGTGGTGCGTGGACGACCCTACATGTTTTCGATTATG  2854

Seq_1  3004  AGTTTATGACGACCCACCGGCACTGCACGGTGTTGCCGGTGTCCAGACTCCAGCGCTAGT  3063
             |||||||||||| ||||||||||||||||||||||||||||||||  |||||||||||||
Seq_2  2855  AGTTTATGACGATCCACCGGCACTGCACGGTGTTGCCGGTGTCCAGGTTCCAGCGCTAGT  2914

Seq_1  3064  TGTCTTGCCCGGGGTGTAGTCAAAGTCGGAAATCTTACCCCCGATGAGTTTTAGAGAAAA  3123
             ||||||||||||||||||||||||||||||||||||||||||||||||||||||||||||
Seq_2  2915  TGTCTTGCCCGGGGTGTAGTCAAAGTCGGAAATCTTACCCCCGATGAGTTTTAGAGAAAA  2974

Seq_1  3124  TTGCAAAGACATTGAATACAAAAGGAAATGGAAGAGAATTGAGATGAAAAAAGAGTTATG  3183
             |||||||||||||||||||||||||||||||| |||||||||||||||||||||||||||
Seq_2  2975  TTGCAAAGACATTGAATACAAAAGGAAATGGAGGAGAATTGAGATGAAAAAAGAGTTATG  3034

Seq_1  3184  GAGCTCATTACTTAGAGGAGAGGGAGCACTCTTTTTTTTACGGCAGGGGAGGAACACTAT  3243
             ||||||||||||||||||||||||||||||||||||||||||||||||||||||||||||
Seq_2  3035  GAGCTCATTACTTAGAGGAGAGGGAGCACTCTTTTTTTTACGGCAGGGGAGGAACACTAT  3094

Seq_1  3244  TTTTTTTAAGGCAGGAGAGGGAACACTCGAATTCTCTTGGAAAGTCAAGAGTCTAACTTA  3303
             |||||||| || ||||||||| ||||||||||||||||||||||||||||||||||||||
Seq_2  3095  TTTTTTTACGGTAGGAGAGGGCACACTCGAATTCTCTTGGAAAGTCAAGAGTCTAACTTA  3154

Seq_1  3304  TTCTTTTTGGCCACTAATTAATTAAAACAATTTATGATGGTCAGATTAATGTCACCACTA  3363
             ||||||||||||||||||||||||||||||||||||||||||||||||||||||||||||
Seq_2  3155  TTCTTTTTGGCCACTAATTAATTAAAACAATTTATGATGGTCAGATTAATGTCACCACTA  3214

Seq_1  3364  AATTTGTCATCAGAATTGTTTTGCAATATTTTTTTACATTCACTTATATATGTATAAGAG  3423
             ||||||||||||||||||||||||||||||||||||||||||||||||||||||||||||
Seq_2  3215  AATTTGTCATCAGAATTGTTTTGCAATATTTTTTTACATTCACTTATATATGTATAAGAG  3274

Seq_1  3424  AACTAATGATCATAGTTCCATACGTGACTCGATCTACGGATAATCAACAGCACATATGTT  3483
             ||||||||||||||||||||||||||||||||||||||||||||||||||||||||||||
Seq_2  3275  AACTAATGATCATAGTTCCATACGTGACTCGATCTACGGATAATCAACAGCACATATGTT  3334
```

```
Seq_1  3484  TTGAAACCGAAGGTGTACATGCCAATTCTTTTAGTAGTCTCACCGATCCATATTATTCGT  3543
             ||||||| ||||||||||||||||||||||||||||||||||||||||||||||||||||
Seq_2  3335  TTGAAAC-GAAGGTGTACATGCCAATTCTTTTAGTAGTCTCACCGATCCATATTATTCGT  3393

Seq_1  3544  CGCTGATTTGGTTACATGCTCCCTCCCATGCATATTATCTGTCGTTGATTTACTGGATAG  3603
             ||||||||||||||||||||||||||||||||||||||||||||||||||||||||||||
Seq_2  3394  CGCTGATTTGGTTACATGCTCCCTCCCATGCATATTATCTGTCGTTGATTTACTGGATAG  3453

Seq_1  3604  TCACAAGTAATATGGATCGGATGTTCTGCTACATCGATATATTAAGAGATATATCCATAT  3663
             ||||||||||||||||||||||||||||||||||||||||||||||||||||||||||||
Seq_2  3454  TCACAAGTAATATGGATCGGATGTTCTGCTACATCGATATATTAAGAGATATATCCATAT  3513

Seq_1  3664  TAGAGCTCTAAACTAACTCCAATACCGGTTAGAACACAACCCTCAATTCTTAATACTGTG  3723
             ||||||||||||||||||||||||||||||||||||||||||||||||||||||||||||
Seq_2  3514  TAGAGCTCTAAACTAACTCCAATACCGGTTAGAACACAACCCTCAATTCTTAATACTGTG  3573

Seq_1  3724  TAAATTATCCCCTGACCTAGCCAGAAGGTGGTTTTCACATTCCCGGCAGATTTTGACCGA  3783
             ||||||||||||||||||||||||||||||||||||||||||||||||||||||||||||
Seq_2  3574  TAAATTATCCCCTGACCTAGCCAGAAGGTGGTTTTCACATTCCCGGCAGATTTTGACCGA  3633

Seq_1  3784  GTCAACACACATTTTTCAGTCCAAAAAAGATAAAACGAAAAATCTTGTACACAACCTACT  3843
             ||||||||||||||||||||||||||||||||||||||||||||||||||||||||||||
Seq_2  3634  GTCAACACACATTTTTCAGTCCAAAAAAGATAAAACGAAAAATCTTGTACACAACCTACT  3693

Seq_1  3844  ATTAAATTTGGGCTTCAGTGAGCAAACGATAAGTACAACAAATGACATATTTTGTTTGAG  3903
             ||||||||||||||||||||||||||||||||||||||||||||||||||||||||||||
Seq_2  3694  ATTAAATTTGGGCTTCAGTGAGCAAACGATAAGTACAACAAATGACATATTTTGTTTGAG  3753

Seq_1  3904  ACTCAAATTTTGAATTCAAAATTGAATTTCCAAATTTTTAATTATTTATAGGAAATCATA  3963
             ||||||||||||||||||||||||||||||||||||||||||||||||||||||||||||
Seq_2  3754  ACTCAAATTTTGAATTCAAAATTGAATTTCCAAATTTTTAATTATTTATAGGAAATCATA  3813

Seq_1  3964  AACGTTGTTGAGAGCCTACTGTTAAAGTTTCAGCGTCTCGCATGTATGATGTGCCAGTCA  4023
             ||||||||||||||||||||||||||||||||||||||||||||||||||||||||||||
Seq_2  3814  AACGTTGTTGAGAGCCTACTGTTAAAGTTTCAGCGTCTCGCATGTATGATGTGCCAGTCA  3873

Seq_1  4024  ACTTGGTCAAAACCTGCCACGTACGTGTAAACAATCTTTTGGCTGGATCAAGAATAATTT  4083
             |||||||||||||||||||||||||||||||||||||||||||||||||||| |||||||
Seq_2  3874  ACTTGGTCAAAACCTGCCACGTACGTGTAAACAATCTTTTGGCTGGATCAAGGATAATTT  3933

Seq_1  4084  AAGCGGTATGAATAGTTGAAGGATGTGTTTTAATTAGTATCGGAGTTCGGTGTGTGTGAA  4143
             ||||||||||||||||||||||||||||||||||||||||||||||||||||||||||||
Seq_2  3934  AAGCGGTATGAATAGTTGAAGGATGTGTTTTAATTAGTATCGGAGTTCGGTGTGTGTGAA  3993

Seq_1  4144  TTTAAACTATATGGTTAGTTCAACGTTGTAATATGGACTTATCACATATATTAGTCTAGT  4203
             |||||||||| |||||||||||||||||||||||||||||||||||||||||||||||||
Seq_2  3994  TTTAAACTATGTGGTTAGTTCAACGTTGTAATATGGACTTATCACATATATTAGTCTAGT  4053

Seq_1  4204  TCGCATAATACATGATGCTGATTTTTCTAGTTTATCTCAACATACCATTATTATTTGTAC  4263
             ||||||||||||||||||||||||||||||||||||||||||||||||||||||||||||
Seq_2  4054  TCGCATAATACATGATGCTGATTTTTCTAGTTTATCTCAACATACCATTATTATTTGTAC  4113

Seq_1  4264  TACGTGTGCTTGAATTTATTTGTATGCGTACAGAACATGTTTTGGTTAGAATGAGTTTCA  4323
             ||||||||||||||||||||||||||||||| |||||||||||||||| |||||||||||
Seq_2  4114  TACGTGTGCTTGAATTTATTTGTATGCGTACGGAACATGTTTTGGTTAAAATGAGTTTCA  4173
```

```
Seq_1  4324  TTTTTAACCTGGCGTTTGTGCGATTTGCTAGATCTGAAAATCTTAGCTGCACACATGTAA  4383
             ||||||||||||||||||||||||||||||||||||||||||||||||||||||||||||
Seq_2  4174  TTTTTAACCTGGCGTTTGTGCGATTTGCTAGATCTGAAAATCTTAGCTGCACACATGTAA  4233

Seq_1  4384  ATGTTGACTACAAATAGTCGTTAAAATTTTTTGAATATGTAAAAAATTGCACAAGTTAGC  4443
             ||||||||||||||||||||||||||||||||||||||||||||||||||||||||||||
Seq_2  4234  ATGTTGACTACAAATAGTCGTTAAAATTTTTTGAATATGTAAAAAATTGCACAAGTTAGC  4293

Seq_1  4444  TGAATTTAAAACTTATGAGGTAACAATATGAAGTGCAAACACCTTGGAAACCTAAAATTA  4503
             ||||||||||||||||||||||||||||||||||||||||||||||||||||||||||||
Seq_2  4294  TGAATTTAAAACTTATGAGGTAACAATATGAAGTGCAAACACCTTGGAAACCTAAAATTA  4353

Seq_1  4504  AACCCACCTCTCTATGAACAGTCTACCAGCAAGGAAGAACAAAAATAGAAAGCACAACGC  4563
             ||||||||||||||||||||||||||||||||||||||||||||||||||||||||||||
Seq_2  4354  AACCCACCTCTCTATGAACAGTCTACCAGCAAGGAAGAACAAAAATAGAAAGCACAACGC  4413

Seq_1  4564  CGACAACGGTAAATTCAGAAGCCCCAAAAAAATCCAAAGAAGGCCAGATTCGAAAATGCC  4623
             ||||||||||||||||||||||||||||||||||||||||||||||||||||||||||||
Seq_2  4414  CGACAACGGTAAATTCAGAAGCCCCAAAAAAATCCAAAGAAGGCCAGATTCGAAAATGCC  4473

Seq_1  4624  CCGTTTAGGCCGAAGGGGCAGAGAAAAAAGAGATAAAAAAAGCATTGAAAACATGCACTT  4683
             ||||||||||||||||||||||||||||||||||||||||||||| ||||||||||||||
Seq_2  4474  CCGTTTAGGCCGAAGGGGCAGAGAAAAAAGAGATAAAAAAAGCATCGAAAACATGCACTT  4533

Seq_1  4684  TAATATCGGGTGGGTCCCACCAACTACCCCCCGCACTCTATAAAGGTCGCCTCCCCTCAG  4743
             ||||||||||||||||||||||||||||||||||||||||||||||||||||||||||||
Seq_2  4534  TAATATCGGGTGGGTCCCACCAACTACCCCCCGCACTCTATAAAGGTCGCCTCCCCTCAG  4593

Seq_1  4744  ATCTTCCCGTCCATTACGCCACCTCTCCACCTAAACCAAACCGTACCGAACCTTGCCATT  4803
             ||||||||||||||||||||||||||||||||||||||||||||||||||||||||||||
Seq_2  4594  ATCTTCCCGTCCATTACGCCACCTCTCCACCTAAACCAAACCGTACCGAACCTTGCCATT  4653

Seq_1  4804  TTGCAACTCACACCTCGCCGACCACGTATTTACTATAACCCGCCTTACCGTTGAGGCCTT  4863
             |||||||||||||||||||||||||||||||||||||||||||||||||||| |||||||
Seq_2  4654  TTGCAACTCACACCTCGCCGACCACGTATTTACTATAACCCGCCTTACCGTTTAGGCCTT  4713

Seq_1  4864  TACTCTCCTTTTTTGCGCCTAGCCCCCTACAAAGTTTCCACATCTTGCGTCTGTGACCTT  4923
             ||||||||||||||||||||||||||||||||||||||||||||||||||||||||||||
Seq_2  4714  TACTCTCCTTTTTTGCGCCTAGCCCCCTACAAAGTTTCCACATCTTGCGTCTGTGACCTT  4773

Seq_1  4924  CACCGGAGTTGCCATATGAATGGTCAAACTACGATGGGGCTCGCCGCAGCCGCAGCCGCC  4983
             ||||||||||||||||||||||||||||||||||||||||||||||||||||||||||||
Seq_2  4774  CACCGGAGTTGCCATATGAATGGTCAAACTACGATGGGGCTCGCCGCAGCCGCAGCCGCC  4833

Seq_1  4984  GCCGTGAGGCCGTGCCGCCGCCGCCTACTCTCGTCCGTCTCAGCCGGGGGCGCGAAGGCA  5043
             ||||||||||||||||||||||||||||||||||||||||||||||||||||||||||||
Seq_2  4834  GCCGTGAGGCCGTGCCGCCGCCGCCTACTCTCGTCCGTCTCAGCCGGGGGCGCGAAGGCA  4893

Seq_1  5044  TCCGTGACCCCGCTCTTCCCGAGACGCCCCCACCCGCAGCACCACCACCACGGCCGCCGC  5103
             |||| |||||||||||||||||||||||||||||||||||||||||||||||||||||||
Seq_2  4894  TCCGCGACCCCGCTCTTCCCGAGACGCCCCCACCCGCAGCACCACCACCACGGCCGCCGC  4953

Seq_1  5104  CTCCCCTTCCTCGCCTCGGCGGCGTCGCAGACCTCGCACTCCGACCCGAGCTCCACCCCA  5163
             ||||||||||||||||||||||||||||||||||||||||||||||||||||||||||||
Seq_2  4954  CTCCCCTTCCTCGCCTCGGCGGCGTCGCAGACCTCGCACTCCGACCCGAGCTCCACCCCA  5013
```

```
Seq_1  5164  ACAACCACCCCCACCCCTGTAAACTCCGATCCCCGCTCCGCCGTCGCCGGGAACCTCCCT  5223
             ||||||||||||||||||||||||||||||||||||||||||||||||||||||||||||
Seq_2  5014  ACAACCACCCCCACCCCTGTAAACTCCGATCCCCGCTCCGCCGTCGCCGGGAACCTCCCT  5073

Seq_1  5224  TTCTTTGACCGCGTGCTCTTCCCGGGCTCCTTCCCTCCTGTCGAGACCCCGCCTGTGGAG  5283
             ||||||||||||||||||||||||||||||||||||||||||||||||||||||||||||
Seq_2  5074  TTCTTTGACCGCGTGCTCTTCCCGGGCTCCTTCCCTCCTGTCGAGACCCCGCCTGTGGAG  5133

Seq_1  5284  GAGCCGGCGCCGCCGACCGATGAGGGTCTGGCTTCGCCGGTGAGGGAGGAGTCGGAGACG  5343
             ||||||||||||||||||||||||||||||||||||||||||||||||||||||||||||
Seq_2  5134  GAGCCGGCGCCGCCGACCGATGAGGGTCTGGCTTCGCCGGTGAGGGAGGAGTCGGAGACG  5193

Seq_1  5344  GAGAGGGAGGCCTGGAGGCTACTGAGGAGGGCGGTGGTGAGCTACTGCGGCGAACCGGTG  5403
             ||||||||||||||||||||||||||||||||||||||||||||||||||||| ||||||
Seq_2  5194  GAGAGGGAGGCCTGGAGGCTACTGAGGAGGGCGGTGGTGAGCTACTGCGGCGAGCCGGTG  5253

Seq_1  5404  GGCACGGTGGCGGCGGAGGACCCCGAGTGCACGGAGGTGGCCAACTACGACCAGGTCTTC  5463
             ||||||||||||||||||||||||||||||||||||||||||||||||||||||||||||
Seq_2  5254  GGCACGGTGGCGGCGGAGGACCCCGAGTGCACGGAGGTGGCCAACTACGACCAGGTCTTC  5313

Seq_1  5464  ATCAGGGACTTCGTCCCCTCCGCGCTCGCCTTCCTCATGCGCGGAGAGACTGAAATCGTC  5523
             ||||||||||||||||||||||||||||||||||||||||||||||||||||||||||||
Seq_2  5314  ATCAGGGACTTCGTCCCCTCCGCGCTCGCCTTCCTCATGCGCGGAGAGACTGAAATCGTC  5373

Seq_1  5524  CGCAATTTCCTCCTCCACACCTTGCAGCTGCAGGTGAGCGCCTTGCCTATTGGGGATCCG  5583
             ||||||||||||||||||||||||||||||||||||||||||||||||||||||||||||
Seq_2  5374  CGCAATTTCCTCCTCCACACCTTGCAGCTGCAGGTGAGCGCCTTGCCTATTGGGGATCCG  5433

Seq_1  5584  GATTGCTTAGTTTGAGTTGAAAATTAGCGCGTTGTGACTACTAGTGTTGATTGCGTGCAC  5643
             ||||||||||||||||||||||||||||||||||||||||||||||||||||||||||||
Seq_2  5434  GATTGCTTAGTTTGAGTTGAAAATTAGCGCGTTGTGACTACTAGTGTTGATTGCGTGCAC  5493

Seq_1  5644  GTCACTTGCTAGTTGGGGGGAAGTTAACACCGATTTTGCGTTGTTTGAATGTGTAGTACA  5703
             |||| |||||||||||||||||||||||||||||||||||||||||||||||||||||||
Seq_2  5494  GTCATTTGCTAGTTGGGGGGAAGTTAACACCGATTTTGCGTTGTTTGAATGTGTAGTACA  5553

Seq_1  5704  TCAGAATTTTCGGCATTAGTAAACGTAACATACCATTCCTTAATGAATTTGCTCTCAGCA  5763
             ||||||||||||||||||||||||||||||||||||||||||||||||||||||||||||
Seq_2  5554  TCAGAATTTTCGGCATTAGTAAACGTAACATACCATTCCTTAATGAATTTGCTCTCAGCA  5613

Seq_1  5764  TGTGAATTTTTTTCTTCTAATTAATTCCTGTTCTAATTGGCTTGTATTGTATTCTATGGG  5823
             ||||||||||||||||||||||||||||||||||||||||||||||||||||||||||||
Seq_2  5614  TGTGAATTTTTTTCTTCTAATTAATTCCTGTTCTAATTGGCTTGTATTGTATTCTATGGG  5673

Seq_1  5824  TGAAATAGAGATAAGATTTCACTTTTGGAGTCTAATATGTCTCTTTCATACATGTACTAA  5883
             ||||||||||||||||||||||||||||||||||||||||||||||||||||||||||||
Seq_2  5674  TGAAATAGAGATAAGATTTCACTTTTGGAGTCTAATATGTCTCTTTCATACATGTACTAA  5733

Seq_1  5884  GCAGTACTTGTTTGGTAGCGATTATACATCCTCATAGCTTAGCCTAAATTAGCCTAAACG  5943
             ||||||||||||||||||||||||||||||||||||||||||||||||||||||||||||
Seq_2  5734  GCAGTACTTGTTTGGTAGCGATTATACATCCTCATAGCTTAGCCTAAATTAGCCTAAACG  5793

Seq_1  5944  GCAATTATGAGGTAACAATAGTTATCTGTATGCAATACTACCACACTGAGTAAACTTCAC  6003
             |||||||||||||||||||||||||||||||||||||||||||||  |||||||||||||
Seq_2  5794  GCAATTATGAGGTAACAATAGTTATCTGTATGCAATACTACCACA--GAGTAAACTTCAC  5851
```

```
Seq_1  6004  TTTTTTATACGATTCGAAAAGCCTAGAACAGACGAGAAAAATCTACAACTTTTGTCCAAA  6063
             ||||||||||||||||||||||||||||||||||||||||||||||||||||||||||||
Seq_2  5852  TTTTTTATACGATTCGAAAAGCCTAGAACAGACGAGAAAAATCTACAACTTTTGTCCAAA  5911

Seq_1  6064  TTTCCTTAAGATGTGATCACATTTACCATAAAACGCCACTAAACTGGCTTGCTAGTAATT  6123
             ||||||||||||||||||||||||||||||||||||||||||||||||||||||||||||
Seq_2  5912  TTTCCTTAAGATGTGATCACATTTACCATAAAACGCCACTAAACTGGCTTGCTAGTAATT  5971

Seq_1  6124  GTTTGTGAACTTTAGTTTCTTATCTGGCACTTGAATCAAATTTTCTTCATTTACTGATCT  6183
             ||||||||||||||||||||||||||||||||||||||||||||||||||||||||||||
Seq_2  5972  GTTTGTGAACTTTAGTTTCTTATCTGGCACTTGAATCAAATTTTCTTCATTTACTGATCT  6031

Seq_1  6184  TTCTCATTTATTTGATGATCAGAGCTGGGAGAAAACTGTTGACTGTTACAGCCCTGGGCA  6243
             ||||||||||||||||||||||||||||||||||||||||||||||||||||||||||||
Seq_2  6032  TTCTCATTTATTTGATGATCAGAGCTGGGAGAAAACTGTTGACTGTTACAGCCCTGGGCA  6091

Seq_1  6244  AGGCTTGATGCCAGCCAGCTTTAAGATTAGGACTGTTCCTCTTGACGAAAACAACGAAGC  6303
             ||||||||||||||||||||||||||||||||||||||||||||||||||||||||||||
Seq_2  6092  AGGCTTGATGCCAGCCAGCTTTAAGATTAGGACTGTTCCTCTTGACGAAAACAACGAAGC  6151

Seq_1  6304  ATTTGAGGAGGTTTTGGACCCTGACTTTGGTGAATCCGCTATTGGCCGTGTAGCTCCAGT  6363
             ||||||||||||||||||||||||||||||||||||||||||||||||||||||||||||
Seq_2  6152  ATTTGAGGAGGTTTTGGACCCTGACTTTGGTGAATCCGCTATTGGCCGTGTAGCTCCAGT  6211

Seq_1  6364  TGATTC------------------------------------------------------  6369
             ||||||
Seq_2  6212  TGATTCTGGTATATTTCCCTCCCCGCCTCTCGAACATAATCCCTTTCCTTTTGAAAATTA  6271

Seq_1  6370  ------------------------------------------------------------  6369

Seq_2  6272  GGTGTTAGTATTGAATGACATCAAGTTAGTAATTTGAAATGTTTATCCTTCTTCTTTAAC  6331

Seq_1  6370  ------------------------------------------------------------  6369

Seq_2  6332  CTAACCTAACCTAACCTAACCGTTTGCAGGACTTTGGTGGATTATCTTACTCAGAGCATA  6391

Seq_1  6370  -------------------------------------------------  6369

Seq_2  6392  CTGCAAGATTACAGGGGACTATTCTTTGCAAGAAAGAGTGGATGTCCAA  6440
```

BdindelWSU_7, upstream

>Bradi2g13600

TTCGTCGCGAACAGCCACTACGGGCTAAATAGTTATACCAACTAAGCTTGAGCAACTTAGCAATAGGAACAGGCTTTGTCATCATATC
AGCAATGTTGAAAAGTGATCCGAATCCGTTACCAACTCTTGTAGACATAGCAGGATAGGTTTGGGCTTTGCGTTGGTACTGACGTAA
ACTCGTTTTCCTTGTCCTACTAGGACTCATGTTTATTGCCCATTATATATGTCCCATGTAGTCGCACGTAAAGACGGCATGTCATCTCGT
GCCTGTAATACTCCAACACAAGTGAAGTTGCGACTTTGTGCATCCGTGATTTTTCCCGCAAGGGTTTCCACGTTAAAATTCATGTCTCT
CGGTTCTAGTTTAATCTCTATTTTTGTAACAAGCAGGATTATCATGAGTGCTAATCTTGTATACCTTCAATTTACCTTATGCAACAACGT
CGCGAACATAATGATACTTGACATCAATGTGCTTGGTTCTCTCATGTAGCATCTGATCTTTAGTAAGGTATATAGCACTTTGACTGTCA
CAAAACAGGTTAATGCAAGAAACATCCTGATAAAGCTCAGCATACAATCCCTTCAACCAAACAGATTCTTTGCAAGTTTTAGCAATAG
CCATATATTATGCTTCAGTTCTAGATTGAGTAACAACTGGTTGCGGTGTTGCCTTCTAACTCACAGCACATCCACCTACCGTGAACACA
TAACTTGTGAGGGATCTCCTCTTATCCAAGTCGGCAGCAAAATCTGAATTTACATAACCTGCGAGTCCCTCATCAGTCTTGCCAAACTT

CAAGCAAGCATTGCATGTGCCACGGAGGTATCTGAAAATCCAATGAACAACTTTCCAATGTTCTTTACCGGGAGATTAGGCATGTAAC
GACTGACCGCATACATCAAGGAACCAACAATAATAGAATAGGTAACTCGTGACATGTACTCAATATCTTCATCAGAAGTAGCACATTG
CAATGCTGAAAACTTGAAATGAAAAGCAATATGAGTACTAACAGACTTTGCATCATGCATATTAAAACGATGAAGAACTTTCTCAATG
TAACTTTGCTGACTAAGAAATAGCAACCTAAATTTTCTGTCCCTTTTAATTTCCATACCTAGGATTTTCTTAGCAGCACCAAGATTCTTC
ATCTCAAATTCACTACTTAGTTGAGCCTACAAAATAGTGATTACTTCCGTGCTCTTGGCAGTAATTAACATATCATCAACATATAACAG
CTGGTATATCGGTGATCCATCAACAAATTTAATGTAGACACAATTGTCATACTGAGATCTACTAAAGCCACGTGAGATCATAAATGAA
TCAAACCTTTTATATCACTGTCTAGAAGATTGCTTTAAACCATAAAGGGACTTCTTCAACTTGCATACAAACTCCTCTTTAATAGGAACC
ATGAACCCTTCAGATTGGTCTGTATATTTCCTCCTCAAGCTTTCCATGTGAAAACGCAGTCTTTACATCTAACTGCTCAAGCTCAAGATC
GTGCATAGCAACAATACCAAAGAAAGTATGAATGGAACTATACTTTACCACTGGAGAGAACACATCATTATAATCAATACCTGGTATT
TGACTGAAACTTTTTGCCACTAAACTTGCCTTTAAACCTCATAGGCTCATTAGGAGACGAGCCCTACTTTCTTTTGTATATCCATATGCA
GCGAACAACCTTCTTTTGTTCAGGCAAGGGCACAACATCCCATGTGCCATTTTTCTCGAGAGATTGCATCTCTTCTTGCATGGCAGACA
ACCACTTAACACGGTCATCGAAAACAACTGCTTCATTATATGTGGTCGGTTCACTATCATGATCCACCTGTTCTGCACAACTCAAAACA
TAATAAACAAGATCACACTCTTCAATTAAACGTTTGCGAGGACCCTTATTTCTCTTTGGACGGTTAGCTGCAATAGGCCGTGTTGGTTG
CTGCAAAACAGGTGGTGAGGGTGGAACACTATCATCATTATTATCATCATTGTTCCCTTCATCATTACCACGATCAACATGAGCATCAC
CGTTGCCAACAATTTCATCAACATGCTCCGCCTGCACTCTAACTCTCTCCTCTTCAATTCTAGAGGCATCAGTGGACAAAGTATTATGG
TACATAACTTTTTCATTAAAAACAACATTCCTGCTCAGTAAAATCTTTTTAGCTTCAGGATTCCACAACCTATAAGCTTTAACTCCAGAA
CCATAGCCAAGAAATATGCACTTAACGGCCCTAGGCTCTAGATTTCCATTATCAACGTGAGCATAAGCGGTGCAACCGAAAACTCTCA
ACTGTGAATAATCAGAAGGTTTAACAGACGATATCTCAATGGGAGTTTTCTTGTCAAGTGGAATAGAAGGTGACCTGCTTATGAGGT
AGCATGTTGTGAAGGCTGCTTCAGCCCAAAAATGTTTGACCAAACCTGCATTGGACAACATGCAACGAGCCCTCGAGATGATGGTTA
TGTTCATGCGCTCAGCCACGCCATTTTGCTGTGGAGTACGGACAGCCGTGTAGTGCCTAGCTACACCATCATTGCTGGAAAACTCCTC
AAACTCATCAGAACAGAGTTCAACACCATTGTCAGTACGTAATATTTTTACCTTCCTTCCAACTTTCTTTGCTACCATAGCCTTTCACTTC
TTGAACGCACCAAAAACATCAAATTTGTGTTTCAGAAAATAAGGCCAAACTTTTCTTGAGTAATCATCAATAATACTAAGCATGTAATT
AGCACCACCAATAGAAGTCTTACAGGAAGGTCCCCAAACATCGTAATGCACATAATATAATATGCTTTGGTGGTATGAGCGGAAGCA
ATGAACTTAACCCTTTTGTGCTTACCATAAATGCAATGCTCACAAAACTCAAACTTACTCAAGTTGCAGCCATCGATCAGCTCTCTCTTA
ACCTATTCTGACATGCCACGTTCACTCATATGCCCATGACGCATATGCCACAGATTCGTTTTAGCACAATTATCAGAATCATTAGGTGT
AACAGCAGCAGCAAAACCAGGTAAAGTGCTAGTAAGAACATACAATTTGGCAGAATTCATATCACCTATCATGTGAATAAGAGAACC
TCTTGCTACTTTCAAAAGTCTACGCCCTCCGGCTATTTGTACCCTTCATAATCTATGGTACATAGCGAGATCAAATTTCTTGCCATACTT
GGTATGTGGTTCACCTCATTCAACGTGCGTGTCATGCCATCATGCGTCTTGATCTGAATAGACCCAACGCCAACAATATGAATGGGAT
TGTCATTGCCCACACGCACAAACTCATAACAACTGAACCAATCTTTGTTACAACAAATATGAAATGAACATGCAGCATCAAGAATCCA
TTCATCATCATGACAAGCACAAGCAGCAAGAACAACGAGGCATTCACCCTCAGAGCTGTCGGTAGAAACGACAACAGCCTTACCAGC
ACCGTCGGACTTGTCTTTCGGTTTGTACGTACCGTTCCTTTTATCCTTGTTCTGCAGTTTATAACAATCCTCAATAACATTCTTTTTTTTCT
TACAATACCTGCGTAACTTATCTTTTTGCCTGTACTTTGAACGAGCTTTCCCGTCCCTGCTCTTATCTATGTTATTGCAGTTGTTGTTTCT
CTGCTCGGTCATCCCACAAACCTGCAGAGCTTCAGCCTTGGAAGATGACCCATCGGTCTGCACCATAGATTTCATCTTCTCTTTCTGTC
TAAGTGCCTCTTGGACTTCAACAAGGGTTAGTGTATCATGGCTCAAAAGAATTGAATCATGGAAATTTGTAAAGGAACTATGCAACG
AGCATAAGAGTAAAAGGCTAAATTCTCATCATCATATATTACTTCCAAAGACCGCAAGTCAGAAATGATCTCTCTAAAGGCAGCCATA
TGAGATATTTCTGATCCACCTTCTTGGAGCTTATGTAAGAACAACTTCATCTTACTTGCATCTTATTGGTTAGATCCTTTGTCATGCAGA
TCGATTCTAGTTTCACCCACATGGCTGCTGCGGATTTCTCCTCCAACACTTCCTGCAAAATAGTATTGGACAAATGAAGTTGAATCAAA
GACAAAGCCTTACCATCTTTTTGTTTCTCTTCGGCAGTCCACTCTTTTGCATCCTTCTTTCCGAAAGAATAAAGCGCTTCATCTAGATCT
GAAGTTTGGACGAGAATCGCACGTATCTTCACTTGCCACAACGAGAACCGTGTGGTGTAATTCAGCTGCAGCAGATCGAACTCAAAG
TAGACATGTCGCAAACTCTAGGCAGAACCTGGTGTTCTGATACCACTTGTTATGAACGCGACAAGCGAGAACGAAAAAGAAATCACA
GCACAAGTACACACGGATTTAACGTGAAAAAACCTCAACCAATGAGAGAAAAACCACGGACGTCAGACAGCGAAACTTCACTATATG
GAGAGTATTTACACAACGGCAGGGATGTTTCACGAATGCAACTCATCCCGAAACGACGGCTTACAAGGGGTATATATAGCAGGAAC
ATTAGGTTAAAATAGATCCGTATCGGTGCATCCGAAAATTCCGGGTGCACGACCGGAAGTTCCGGGTGCGTGGCCGGAAGTTTCACA
AATTTCTCGAAGAGTAACCGAGATTCACAAATTCCTCGAAGAGTAACCGAGAGTATCCCGAGGAACAACCGGACTTACGGTGGAAGT
TTTGTTCCTGGGAAAGCCTTGGCAATGAATATGGATCACAATTTCAACGCTAACATCGTTCCGCTGGCAAGGTTGTATCCGCCCTCCT
GTCTTGTACGGAGTACACCGTAAAGACACCTGATGCCAATCACAATTGAGCCAGATGGTAGATTGTCAAAAGAATCTAAAGAAACCG
CATTTCTCATTAGTTGGTCTTTAAGAATTTCTCTTTCACGCAATATTTCCTTCATTTTGAGAATCTGAAGACTTAACCTGCTCCTTCCTTG
CTGAGACCCGCGTAATCATTGCTTTGTGCTAGAACCTACTCCTGCTTTCTAGAAAGACAATATAAAAAAAAAACTGTAGTGCAAGAACG
GAAAGGAAATTCCACTCGTATGAGCGAGTGAAGGTGGTGTTGCAAACTCTACCTACAATACAAGCGGGTCAGACTAGGTGAAGCTG

ACCGATATATATTAGCTGTCTGGAAACACGTTTATACTACTAATATATAGCATAAACTCTATCAAAGAATATGAAGTAGTATATCACAC
AACTACTGGGCAGTCACATGCATTCTTGCAATGAATAGTATAGATCCAACGGTCTTGCAATCATCTCCCACATTTTTAAGTGATTTCAT
GATTGTGCAACCAAATACATACAGATTCTGTGACTCTAGAAGCAAATATTCTACATAGGGATTATTCAGAGTTGTTTTTTCTTAGAATT
ATTCAGAGTTGTTGACGTAAAGAACTTTTGCAAATACAAAAGGTTGGCCAGAATAAAAAAAACAAAGAACGAGGTGTAGTCCTGTTC
CCGATTGTACTCAACAGTAGGATAGGAGGGCCTTTCTTTTCCCTTTTTGAAAGTTAAGCAACGGAAGGAACGAAGGAGAAGGAAGG
ATTAACCGATGGATATGGATGGATGTAGCCCGAAGACGAGGTGGCTGTGTCTGAGTTGGCTGCCCTTGCTCCATCAGTCCCAACGGA
ATATCACGTTGGTGTAGCGTAACGCCGAGCCCCATACGTGACGTGATGCATGCACCAGCTGTGTACAGCTAACACCGCCACGCGTTC
CCATGCAACCCCCGGGAAACCAAAACCTGACAAAAAATATGAAGAAATTGCGTGGCCGGAACCTGACTGCGGCCGCCAACCGCGCC
ACGCAGGTTCGTTGGTTTCGGTGGCACCGGGTTGGGTTGGGTTGGGTTGCCAGTCCATGTCCAGCAGTCCAGGCGGTCAGCGGCGC
AGCCAGTTTCCCTTCCCTCGCAAGCCAGCCAGCCAGCCTCCCTCACCAGAACAGGACGCGGTTTTATTAGCAGAGCAAGCAAAGGAA
TTCCTACCCCTCCACATTTAAAAGACAGACGCCACCGAAACACCCCCACAACTCCATCCAACCCTGCTGAGGCCACAGCGGTTCCACG
CAAAACTCTTTGGTGCTGAAAGCTCTTTCGCTCGCTCTTGGCTTTGCTTTCCCTGGTTCCTGGTCGACTGGCTGAGTGGCTGGCTAGCG
GGC

>BdiBd21-3.2G0181400

TAAACTGAGCTTTTTCTTTCTGATGCATATGCTGTGTGTGACAGTGTGAGGCTTCCATTCATGGAAGAGTTGACTTCACTTTTTTTTTGT
GTGTGTGGGTCGGGGTTGGGTGGTTTATTAACCTCTGGCAACCTGAACTTCCTCCTGGCCGGCATTTCGCTGTGCTGAAGAGTGATC
GGTCAGTAGGGTGATCTGTCCTGATCGGATCGAGTCATATCTTATTTTGGCCAACGAAGCATGCATACGTACAGGTTGGGGTTGGTA
TCTGGGGAAAGTCAAATGGCACGTTGGCGCGTGGAAGGACGAAGCATCCTGGACGGCCACGAAACGGAGGCGTCACAGAGAATCC
GGATTTATTGATCCTCAAGCTTGAGAAACAAACATTCGAAGGGCCTCCCTAAAGCGATCGACACCTCCAAGAATCCTTCCTTCGTCGA
GAAAAGCTGAGGAGAAACACGAAAGAAACGAAAGGGAAGAAAGCCCATGTCGCTGTTGTCTTTGACGAGTCGGCCAATCCAGAAG
CGCCATGGTTTTGGCTGCTTGCCATGCCGTGATTAGTCGCCGACCCACCAAGACTGGCCACTACGACGTGATGCCGATGGTTGATGTC
TCTTCTTCACCGCACGCAAATTTAATTATAGAGCGGTAATTTTCAAGCTCCTTTTCGTAAACAATGGGGCGCAAGCTGTATTGGTTCGT
AGAAAACGCCTGATAAATTCATGTGAAGTGGTTCGATCTGAAAAAATAAATTCATGACGAGTTCGTACTATCGTCCCTGTCCATGGGC
GAAATAACAAGCAATTGTGGTTTCTCACCGGCGTATAGACAAAACATTTTTTTTTTTTAAAAGGATATAGACAAAACAAATTGAACCT
ATTTCAGGGGGGAAAAGTTGCGTGGCCACAAAATTCTCTGTAACGCACACGACTGTCGTAGAGTCGCCGAGAACAACCAATGATTCC
CCATCAGAGTGGCAGGTGAATGAGGCTAAACCAGAAATGGGGTAGTTCCTGACATGCATGTTGGAGGCATAATAATAATAGAGAAA
GGCATGTTTGATTTTGGTGGAAAACATGTCCAGGTATAAGTGTCATGTCGTCCTGATATGTGGGACCAACCTAGGACTACCTCATTTG
TAAAATGATATTCGAAAATAATATTCAGTGTGGAGTTAGGTGCAAAATATGGGTCAAGCTGTTGCAAAAACTGAGTTATGGTTCAAA
GTGATGTCCAACCAGGGATTAACATATCCATTTTGAGGTAGGGGAAAGAGGATCTCCAGCAAAATTTAGGAGGGTGAGGATACTTT
GCATAATGATGGAATTAGGATCTTTTAGCTGCCTGTTCTGCAGCCGACGTAAGGAGAAGCGTCGTACTCATCAATTTCCTCGACCGAC
TCAGTTTAACCCAAATTATATTGTTAAGTCTGTTTGTTGCAACAAATCTGCTACATCACCTACCTAAAAACACTACGTTAAAGCATTGCT
ATGATACGATGCTTTTACACTTTACGTAGCACACGTGTTGTTCTTTGGGAAAACGATCGCAGAGCACGCGTAAAATGCATATCTTTTTT
TTTCCTAAAGAAAAATGCGTAATTTTGTCCACTCAAAACCCAAACACTTTTTTATGTGAACTCCGCATGATTCTTTCCTACTGTATATGT
TTCAACGACGCAAACGGACCACTGTTCCTCGGAGCACCCGACGCCGAGCGCCGGTCCAAGGAAGGAAGGTCACCATCAGAAAATCC
AACTCGCGATTGCTAGTATTAGAACTAAAACTTATAGGGCACCATTTGAGATATATGCACCAAAACTGTTTTTGTTTTTTTGAGATGAT
GTATTTTAGGACACTGCTGCCGCACTAGATGATGTAAAATAGGGCACGGTTGCGCACCAAACACAACTATTTTTCCCGGGCGTGGTCT
TTTGCCTCTTCGGCTTTCCGTCGGTGGCTGGCGCATTTGTCCTCCTTCAATCGGCGCATTTGCCTGGCTGTGCCATGTCGCGGCACATT
CCCTCCCACGGCGACAGATCAGGGCACGTGTGAAGACCGAGCGTCAGGGGAGGGGAGAGGGGGTGGCACCCGGTTCATGGAGGC
AGCGGCGAGAGGGGTGGACTGGGTTTTGGCGCCACCGTACACGGCGTTCTGGTACCGGTGGAAGGCGGGGTGAAAAATTTCCGAG
TGGCTATTGAGGGCTTCGCACGCGCTGAGATTTTGTTTTGCGCTAAAAATTAGTTTCTACGATACTACTCTGTTTGATGTGTGCCAAAT
ACAGTCCTACCAAACGATAGTTTGGCACTCACTTTGCCAATTATTTGACAAGAATCCGTGAAAAAAGTGGGATGAACCAAACGACAA
CCAGATGACCATAGGTTAATTTTTTTTTAAAAAAAACAAATCCAGCTATTTTAGCTTGGCTACTAGTTGAAGGCTACTAAGGCCCTGTT
TGTTTGGACTTCTGTTTCAGCTTTTGATGCTTTGAGCAATCTTAAAAGCACTTCTTCTGTTTACACATGAAGCTTGAAAGCACCTCCAGA
CGTGCTTCTCAGCTAGAAGCACCAAAAACACGTCCGGAGATACTTCTCAGCTTCATGTATAAACAGGAGAAGTGCTTTTGAGATTGTT
AGAAGCACCAAAAGCTGAAGCTGAAACCCAAACAAACATGGCCTAAACCACGGCCAGCTATCGTTGGCACTAGGCACCCGCCACCG
CACGGAGCACCGGCCAGGCACCTTGGTCCCTCGCACTCGCAATCTACGCGCTTTCGTGGCAGTCCCATCCATCCAATCACACACCTCC
TCCTTTGCACGTAACCGATGACAGCACCCGGTTCCGCCGAGCGCGACGGCTGGGTCTCGGTCTCGGCCGCGGACCCGTTCCTAGCTA
GTTCCACCCCCAGGGTCGGATCGGAACTTTTCCTGTGGATCCATCCATCCATCCATCCAGGAGGAACACACAGCACAGTGCAGGCGC

TTGCGTTGCTCAGGTCGGGTCTCAGGTCCGCCGTGCCTGCCTGCCTGCATGCTGAAGTCCGATGCAAGCGAGAGCGAGAGAGCAAC
AGCTTATTTTCTTTCCCTCGCGTTATTGCTTGCTCAGAGTACCACTTCGAAGTGGCTGTGAAGTTTCCTTCTGCCTTGGCAAATAAAATC
AATCTGCGTGCGTACGCACCGGGTGACCGGGCCGGTCAGAACTCATTCGCGTTGAAGCATCGCCTTCCTTCCAAAGCCTACCTCTCGC
CTTTGATGGAAGCAAAGCGATTCGATCCGTTCGTAAACCGGTAAACGTCGACACGCTTCAGAGTAGGATTACGTAGAGCAGGTGGAT
TTCCTACCAAGGGAACGCTGTCAGTCTGTCACACCATGGGAGGGAGTTTTTGGGCTGACCGTAACCTTGAATTAGTCAGTCTCCATGC
ATGTCCGATGCTCTTATATATGTGACCGACCATCTCTTGCTCGTGGAATTGTATTGCCTCTGCTCGTGGGAATCAGATGGGAGGATTA
GGGCGACTGGGGATCAGGGGACGAAATTAGAGACTTAATTTATTTTTAAGTGATTTTTAATGAGCTAATTAGTATATTAAGTAAAATT
TATTTTCTTAGGAGGGCAAAGCCCCCCGCCGCTCCCCCCACCCCCTTCTCCATCCCTGAATGTGAGATGTGACCAACATCAAAATTCTT
GTTATAAACATTACTTTCTCAGTTCCATAATTCTACAGAGACAGGTAGATAGGGCTACAAAACTCTTCGACAAAAACAAATCTAAATA
AGTTCTTACCGAACTTATCTAGTTTTGTCTTAAGTCAAACTTGTTTAGGTTTGACCAACTTTGTTGAAAAGAGTAGCGACACATATGAC
ATCAAATCGTCATATTATGAAACTACATTTCATAATTGATCTAATGATGTTAATTTTGTGTCATAAATGTTGTTACGTTTTTCCATAAAA
TTGGTCAACTTCAACATCTTTGACTTATGACAAACCTATAAGTACACTTATTTTGCTCGGTCATGCCACGAACCTGCAGAGCTTCAGCC
TTGGAAGATGACCCATCGGTCTGCACCATAGATTTCATCTTCTCTTTCTGTCTAAGTGCCTCTTGGAGCTTATGTAAGAACAACTTCATC
TTACTTGCATCTTATTGGTTAGATCCTTTGTCATGCAGATCGATTCCAGTTTCAGCCACATGGCTGCTGCGGATTTCTCCTCCAACACTT
CCTGCAAAATAGTATTGGACAAATGAAGTTGAATCAAAGACAAAGCCTTACGATCTTTTTGTTTCTCTTCGGCAGTCCACTCTTTTGCA
TCCTTCTTTCCGAAAGAATAAAGCGCTTCATCTAGATCTGAAGTTTGGGCGAGAATCGCACGTATCTTCACTTGCCACAACGAGAACC
GTGTGGTGTAGTTCAGCTGCGGCAGATCGAACTTCAAGGTAGACATGTCGCAAACCCTAGGCGGAACCTGGTGTTCTGATACCACTT
GTTATGAACGCGACAAGCGAGAACGAAAAAAAATCACAGCACAAGTACACACGGATTTAACGTGAAAAAACCTCAACCAATGAGAG
AAAAACCACGGACGTCAGCCAACGAAACTTCACTATATGGAGAGTGTTTACACAACGGCAGGGATGTTTCACGAATGCAACTCATCC
CGAAACGGCGGCTTACAAGGGGTATATATAGCAGTAGCATTAGGTTAAAATTAGATCCGTATCGGTGCACCCGAAATTTCTGGGTGC
ACGACCGGAAGTTTCACAAATTCCTCAAAGAGTAACCGAGAGTATCCTGAGGAACAACCGGACTTACGGTGGAAGTTTCGTTCATGG
AAAAGCCTTGGCAATGAATATGGATCACAATTTCAACACTAACATCGTTCCGCTGGCAAGGTTGTATCCGCCCTCCTATCTTGTACGG
AATACACCGTAAAGACACCTGATGCCAATCACAATTGAGCCAGATGGTAGATTGTCAAAAGAATCTAAAGAAACCGCATTTCTCATTA
GTTGGTCTTTAAGAATTTCTCTTTCACGCAATATTTCCTTCATTTTGAGAATCTGAAGACTTTAACCTGCTCTTCCTTGCTGAGACCCGC
GTAATCATTGCTTTGTGCTAGAACCTACTCCTGCTTTCTAGAAAGACAATATAAAAAAAAAACTGTAGTGCAAGAACGGAAAGGAAA
TTCCACTCGTATGAGCGAGTGAAGGTGGTGTTGCAAACTCTACCTACAATACAAGCGGGTCAGACTAGGTGAAGCTGACCGATATAT
ATTAGCTGTCTGGAAACACGTTTATACTACTAATATATAGCATAAACTCTATCAAAGAATATGAAGTAGTATATCACACAACTACTGG
GCAGTCACATGCATTCTTGCAATGAATAGTATAGATCCAACGGTCTTGCAATCATCTCCCACATTTTTAAGTGATTTCATGATTGTGCA
ACCAAATACATACAGATTCTGTGACTCTAGAAGCAAATATTCTACATAGGGATTATTCAGAGTTGTTTTTTCTTAGAATTATTCAGAGT
TGTTGACGTAAAGAACTTTTGCAAATACAAAAAGTTGGCCAGAATAAAAAAAACAAAGAACGAGGTGTAGTCCTGTTCCCGATTGTA
CTCAACAGTAGGATAGGAGGGCCTTTCTTTTCCCTTTTTGAAAGTTAAGCAACGGAAGGAACGAAGGAGAAGGAAGGATTAACCGA
TGGATATGGATGGATGTAGCCCGAAGACGAGGTGGCTGTGTCTGAGTTGGCTGCCCTTGCTCCATCAGTCCCAACGGAATATCACGT
TGGTGTAGCGTAACGCCGAGCCCCATACGTGACGTGATGCATGCACCAGCTGTGTACAGCTAACACCGCCACGCGTTCCCATGCAAC
CCCCGGGAAACCAAAACCTGACAAAAAATATGAAGAAATTGCGTGGCCGGAACCTGACTGCGGCCGCCAACCGCGCCACGCAGGTT
CGTTGGTTTCGGTGGCACCGGGTTGGGTTGGGTTGGGTTGCCAGTCCATGTCCAGCAGTCCAGGCGGTCAGCGGCGCAGCCAGTTT
CCCTTCCCTCGCAAGCCAGCCAGCCAGCCTCCCTCACCAGAACAGGACGCGGTTTTATTAGCAGAGCAAGCAAAGGAATTCCTACCCC
TCCACATTTAAAAGACAGACGCCACCGAAACACCCCCACAACTCCATCCAACCCTGCTGAGGCCACAGCGGTTCCACGCAAAACTCTT
TGGTGCTGAAAGCTCTTTCGCTCGCTCTTGGCTTTGCTTTCCCTGGTTCCTGGTCGACTGGCTGAGTGGCTGGCTAGCGGGC

```
Alignment of Sequence_1:  [Untitled Sequence #1] with  Sequence_2: [Sequence Window #2]

Similarity : 3218/6312 (50.98 %)

Seq_1  1      ------------------------------------------------------------  0

Seq_2  1      TAAACTGAGCTTTTTCTTTCTGATGCATATGCTGTGTGTGACAGTGTGAGGCTTCCATTC  60


Seq_1  1      ------------------------------------------------------------  0

Seq_2  61     ATGGAAGAGTTGACTTCACTTTTTTTTTGTGTGTGTGGGTCGGGGTTGGGTGGTTTATTA  120


Seq_1  1      ------------------------------------------------------------  0
```

```
Seq_2  121   ACCTCTGGCAACCTGAACTTCCTCCTGGCCGGCATTTCGCTGTGCTGAAGAGTGATCGGT  180

Seq_1  1     ----------------------------------------TTCGTCGCGAACAGCCACTA  20
                                                      |  | || |||      |
Seq_2  181   CAGTAGGGTGATCTGTCCTGATCGGATCGAGTCATATCTTATTTTGGCCAACGAAGCATG  240

Seq_1  21    CGGGCTAAATAGTTATACCAACTAAGCTTGAGCAACTTAGCAATAGGAACAGGCTTTGTC  80
             |   |  |   |||          |   || | ||  |   |         |||
Seq_2  241   CATACGTACAGGTTGGGGTTGG-TA-TCTGGGGAAAGTCAAATGGCACGTTGGCGCGTGG  298

Seq_1  81    ATCATATCAGCAATGTTGAAAAGTGATCCGAATCCGTTACCAACTCTTGTAGACATAGCA  140
             |       ||||   | ||                |   |  |        |   |
Seq_2  299   AAGGACGAAGCATCCTGGACGGCCACGAAACGGAGGCGTCACAGAGAATCCGGATTTATT  358

Seq_1  141   GGATAGGTTTGGGCTTTGCGTTGGTACTGACGTAAACTCGTTTTCCTTGTCCTACTAGGA  200
             |                           || |      | |    |       ||    |
Seq_2  359   GATCCTCAAGCTTGAGAAACAAACATTCGAAGGGCCTCCCTAAAGCGATCGACACCTCCA  418

Seq_1  201   CTCATGTTTATTGCCCATTATATATGTCCCATGTAGTCGCACGTAAAGACGGCATGTCAT  260
                ||  ||  | |       | |  |     | |     |   |||           |
Seq_2  419   AGAATCCTTCCTTCGTCGAGAAAAGCTGAGGAGAAACACGAAAGAAACGAAAGGGAAGAA  478

Seq_1  261   CTCGTGCCTGTAATACTCCAACACAAGTGAAGTTGCGACTTTGTGCATCCGTGATTTTTC  320
               |     |         |            |      |           |   ||
Seq_2  479   AGCCCATGTCGCTGTTGTCTTTGACGAGTCGGCCAATCCAGAAGCGCCATGGTTTTGGCT  538

Seq_1  321   CCGCAAGGGTTTCCACGTTAAAATTCATGTCTCTCGGTTCTAGTTTAATCTCTATTTTTG  380
              |               |        |     |  |  |      ||     |  |   |
Seq_2  539   GCTTGCCATGCCGTGATTAGTCGCCGACCCACCAAGACTGGCCACTACGACGTGATGCCG  598

Seq_1  381   TAACAAGCAGGATTATCATGAGTGCTAATCTTGTATACCTTCAATTTACCTTATGCAACA  440
                   |  |  |  || | |  ||        |  |   |  |       | | |||
Seq_2  599   ATGGTTGATGTCTCTTCTTCACCGCACGCAAATTTAATTATAGAGCGGTAATTTTCAAGC  658

Seq_1  441   ACGTCGCGAACATAATGATACTTGACATCAATGTGCTTGGTTCTCTCATGTAGCATCTGA  500
              | |       |  |         |        || || ||           |
Seq_2  659   TCCTTTTCGTAAACAATGGGGCGCAAGCTGTATTGGTTCGTAGAAAACGCCTGATAAATT  718

Seq_1  501   TCTTTAGTAAGGTATATAGCACTTTGACTGTCACAAAACAGGTTAATGCAAGAAACATCC  560
               | |     |||             |      ||  ||  |||  | | |    |
Seq_2  719   CATGTGAAGTGGTTCGATCTGAAAAAATAAATTCATGACGAGTTCGTACTATCGTCCCTG  778

Seq_1  561   TGATAAAGCTCAGCATACAATCCCTTCAACCAAACAGATTCTTTGCAAGTTTTAGCAATA  620
             |      ||  |  |   |     |         ||    | |    |          |
Seq_2  779   TCCATGGGCGAAATAACAAGCAATTGTGGTTTCTCACCGGCGTATAGACAAAACATTTTT  838

Seq_1  621   GCCATATATTATGCTTCAGTTCTAGATTGAGTAACAACTGGTTGCGGTGTTGCCTTCTAA  680
                 | ||  | | |  ||    |        |||   |    | || |     ||
Seq_2  839   TTTTTTTAAAAGGATATAGACAAAACAAATTGAACCTATTTCAGGGGGGAAAAGTTGCGT  898

Seq_1  681   CTCACAGCACATCCACCTACCGTGAACACATAACTTGTGAGGGATCTCCTCTTATCCAAG  740
               |     |  ||    || ||   ||   |  | |      |       |
Seq_2  899   GGCCACAAAATTCTCTGTAACGCACACGACTGTCGTAGAGTCGCCGAGAACAACCAATGA  958

Seq_1  741   TCGGCAGCAAAATCTGAATTTACATAACCTGCGAGTCCCTCATCAGTCTTGCCAAACTTC  800
```

```
              |   |    | |   | |  |  || |      |           ||  | ||  || |
Seq_2  959    TTCCCCATCAGAGTGGCAGGTGAATGAGGCTAAACCAGAAATGGGGTAGTTCCTGACATG  1018

Seq_1  801    AAGCAAGCATTGCATGTGCCACGGAGGTATCTGAAAATCCAATGAACAACTTTCCAATGT  860
               |    | |       |   |     | |    |   |  | |           ||
Seq_2  1019   CATGTTGGAGGCATAATAATAATAGAGAAAGGCATGTTTGATTTTGGTGGAAAACATGTC  1078

Seq_1  861    TCTTTACCGGGAGATTAGGCATGTAACGACTGACCGCATACATCAAGGAACCAACAATAA  920
                  ||   |     |       | |    ||    ||  |    |  | |  |     |
Seq_2  1079   CAGGTATAAGTGTCATGTCGTCCTGATATGTGGGACCAACCTAGGACTACCTCATTTGTA  1138

Seq_1  921    TAGAATAGGTAACTCGTGACATGTACTCAATATCTTCATCAGAAGTAGCACATTGCAATG  980
               |       |      | | ||  | |    |  |   |   || ||
Seq_2  1139   AAATGATATTCGAAAATAATATTCAGTGTGGAGTTAGGTGCAAAATATGGGTCAAGCTGT  1198

Seq_1  981    CTGAAAACTTGAAATGAAAAGCAATATGAGTACTAACAGACTTTGCATCATGCATATTAA  1040
                 ||||  |||  |      |||  |||   | |||      |  |     |   ||
Seq_2  1199   TGCAAAAACTGAGTTATGGTTCAAAGTGATGTCCAACCAGGGATTAACATATCCATTTTG  1258

Seq_1  1041   AACGATGAAGAACTTTCTCAATGTAACTTTGCTGACTAAGAAATAGCAACCTAAATTTTC  1100
              |   | |   ||     ||      |   |   |       |  |        |   |
Seq_2  1259   AGGTAGGGGAAAGAGGATCTCCAGCAAAATTTAGGAGGGTGAGGATACTTTGCATAATGA  1318

Seq_1  1101   TGTCCCTTTTAATTTCCATACCTAGGATTTTCTTAGCAGCACCAAGATTCTTCATCTCAA  1160
              ||    |   |  ||  |       | | | |     |                   |
Seq_2  1319   TGGAATTAGGATCTTTTAGCTGCCTGTTCTGCAGCCGACGTAAGGAGAAGCGTCGTACTC  1378

Seq_1  1161   ATTCACTACTTAGTTGAGCCTACAAAATAGTGATTACTTCCGTGCTCTTGGCAGTAATTA  1220
              ||  | | | | |     |  |    |     | |  |   ||    |  |        |
Seq_2  1379   ATCAATTTCCTCGACCGACTCAGTTTAACCCAAATTATATTGTTAAGTCTGTTTGTTGCA  1438

Seq_1  1221   ACATATCATCAACATATAACAGCTGGTATATCGGTGATCCATCAACAAATTTAATGTAGA  1280
              ||| |||  | ||||     | ||   |   |   | |  | ||      | |    |
Seq_2  1439   ACAAATCTGCTACATCACCTACCTAAAAACACTACGTTAAAGCATTGCTATGATACGATG  1498

Seq_1  1281   CACAATTGTCATACTGAGATCTACTAAAGCCACGTGAGATCATAAATGAATCAAACCTTT  1340
              |          |||  ||  |   |   |     || ||  |  |  | |    ||   |
Seq_2  1499   CTTTTACACTTTACGTAGCACACGTGTTGTTCTTTGGGAAAACGATCGCAGAGCACGCGT  1558

Seq_1  1341   TATATCACTGTCTAGAAGATTGCTTTAAACCATAAAGGGACTTCTTCAACTTGCATACAA  1400
               | ||   | |||      || ||  | |  |    |  | ||  || |||   |  | |
Seq_2  1559   AAAATGCATATCTTTTTTTTTCCTAAAGAAAAATGCGTAATTTTGTCCACTCAAAACCCA  1618

Seq_1  1401   ACTCCTCTTTAATAGGAACCATGAACCCTTCAGATTGGTCTGTATATTTCCTCCTCAAGC  1460
              |   || ||| ||  ||||   | |   |||                ||  |  |
Seq_2  1619   AACACTTTTTTATGTGAACTCCGCATGATTCTTTCCTACTGTATATGTTTCAACGACGCA  1678

Seq_1  1461   TTTCCATGTGAAAACGCAGTCTTTACATCTAACTGCTCAAGCTCAAGATCGTGCATAGCA  1520
                   |        |   |      |      |       | || |      | |   ||
Seq_2  1679   AACGGACCACTGTTCCTCGGAGCACCCGACGCCGAGCGCCGGTCCAAGGAAGGAAGGTCA  1738

Seq_1  1521   ACAATACCAAAGAAAGTATGAATGGAACTATACTTTACCACTGGAGAGAACACATCATTA  1580
               ||  |  |||   |    |          || |  | |          |     |  |
Seq_2  1739   CCATCAGAAAATCCAACTCGCGATTGCTAGTATTAGAACTAAAACTTATAGGGCACCATT  1798

Seq_1  1581   TAATCAATACCTGGTATTTGACTGAAACTTTTTGCCACTAAACTTGCCTTTAAACCTCAT  1640
```

```
             | |  |||      |       |     |||||   |    |  |   ||   ||
Seq_2  1799  TGAGATATATGCACCAAAACTGTTTTTGTTTTTTTGAGATGATGTATTTTAGGACACTGC  1858

Seq_1  1641  AGGCTCATTAGGAGACGAGCCCTACTTTCTTTTGTATATCCATATGCAGCGAACAACCTT  1700
              | | || ||| || |     ||        |      ||| |  || | |     |
Seq_2  1859  TGCCGCACTAGATGATGTAAAATAGGGCACGGTTGCGCACCAAACACAACTATTTTTCCC  1918

Seq_1  1701  CTTTTGTTCAGGCAAGGGCACAACATCCCATGTGCCATTTTTCTCGAGAGATTGCATCTC  1760
                               |      |  |    |     | |  |    |    | |
Seq_2  1919  GGGCGTGGTCTTTTGCCTCTTCGGCTTTCCGTCGGTGGCTGGCGCATTTGTCCTCCTTCA  1978

Seq_1  1761  TTCTTGCATGGCAGACAACCACTTAACACGGTCATCGAAAACAACTGCTTCATTATATGT  1820
              ||               |      |    |      |   |  |
Seq_2  1979  ATCGGCGCATTTGCCTGGCTGTGCCATGTCGCGGCACATTCCCTCCCACGGCGACAGATC  2038

Seq_1  1821  GGTCGGTTCACTATCATGATCCACCTGTTCTGCACAACTCAAAACATAATAAACAAGATC  1880
              |          | |      |  | |    |   |           |
Seq_2  2039  AGGGCACGTGTGAAGACCGAGCGTCAGGGGAGGGGAGAGGGGGTGGCACCCGGTTCATGG  2098

Seq_1  1881  ACACTCTTCAATTAAACGTTTGCGAGGACCCTTATTTCTCTTTGGACGGTTAGCTGCAAT  1940
             |  |        |   ||    |  ||            | |     |  |    | |
Seq_2  2099  AGGCAGCGGCGAGAGGGGTGGACTGGGTTTTGGCGCCACCGTACACGGCGTTCTGGTACC  2158

Seq_1  1941  AGGCCGTGTTGGTTGCTGCAAAACAGGTGGTGAGGGTGGAACACTATCATCATTATTATC  2000
              |     |  ||       ||     | |  |    ||      |  ||
Seq_2  2159  GGTGGAAGGCGGGGTGAAAAATTTCCGAGTGGCTATTGAGGGCTTCGCACGCGCTGAGAT  2218

Seq_1  2001  ATCATTGTTCCCTTCATCATTACCACGATCAACATGAGCATCACCGTTGCCAACAATTTC  2060
              |  || | | ||  |   |     | |  |   |   |       |      ||| | |
Seq_2  2219  TTTGTTTTGCGCTAAAAATTAGTTTCTACGATACTACTCTGTTTGATGTGTGCCAAATAC  2278

Seq_1  2061  ATCAACATGCTCCGCCTGCACTCTAACTCTCTCCTCTTCAATTCTAGAGGCATCAGTGGA  2120
             |     |     ||   |      |      |   |    |||  | | | ||| ||| |
Seq_2  2279  AGTCCTACCAAACGATAGTTTGGCACTCACTTTGCCAATTATTTGACAAGAATCCGTGAA  2338

Seq_1  2121  CAAAGTATTATGGTACATAACTTTTTCATTAAAAACAACATTCCTGCTCAGTAAAATCTT  2180
              |||||   |||   || |       ||    |    |  ||  |  |   | |||
Seq_2  2339  AAAAGTGGGATGAACCAAACGACAACCAGATGACCATAGGTTAATTTTTTTTTAAAAAAA  2398

Seq_1  2181  TTTAGCTTCAGGATTCCACAACCTATAAGCTTTAACTCCAGAACCATAGCCAAGAAATAT  2240
                |        |||  |       ||    || |   |          |       |
Seq_2  2399  ACAAATCCAGCTATTTTAGCTTGGCTACTAGTTGAAGGCTACTAAGGCCCTGTTTGTTTG  2458

Seq_1  2241  GCACTTAACGGCCCTAGGCTCTAGATTTCCATTATCAACGTGAGCATAAGCGGTGCAACC  2300
             |   |      |         |  ||           |   |  | |   | || |  |
Seq_2  2459  GACTTCTGTTTCAGCTTTTGATGCTTTGAGCAATCTTAAAAGCACTTCTTCTGTTTACAC  2518

Seq_1  2301  GAAAACTCTCAACTGTGAATAATCAGAAGGTTTAACAGACGATATCTCAATGGGAGTTTT  2360
                ||   | ||         |   |    | | |   |  |   |  ||     ||
Seq_2  2519  ATGAAGCTTGAAAGCACCTCCAGACGTGCTTCTCAGCTAGAAGCACCAAAAACACGTCCG  2578

Seq_1  2361  CTTGTCAAGTGGAATAGAAGGTGACCTGCTTATGAGGTAGCATGTTGTGAAGGCTGCTTC  2420
                 |       |     | ||         |  ||      ||   |           |
Seq_2  2579  GAGATACTTCTCAGCTTCATGTATAAACAGGAGAAGTGCTTTTGAGATTGTTAGAAGCAC  2638

Seq_1  2421  AGCCCAAAAATGTTTGACCAAACCTGCATTGGACAACATGCAACGAGCCCTCGAGATGAT  2480
```

```
                      | | |  | |  |     |   | |   |  ||  |    ||   | ||
Seq_2  2639  CAAAAGCTGAAGCTGAAACCCAAACAAACATGGCCTAAACCACGGCCAGCTATCGTTGGC  2698

Seq_1  2481  GGTTATGTTCATGCGCTCAGCCACGCCATTTTGCTGTGGAGTACGGACAGCCGTGTAGTG  2540
               |      |   | |    |   ||         |     |     |   |
Seq_2  2699  ACTAGGCACCCGCCACCGCACGGAGCACCGGCCAGGCACCTTGGTCCCTCGCACTCGCAA  2758

Seq_1  2541  CCTAGCTACACCATCATTGCTGGAAAACTCCTCAAACTCATCAGAACAGAGTTCAACACC  2600
              |||    |           |   |  |  |  |       |         | ||
Seq_2  2759  TCTACGCGCTTTCGTGGCAGTCCCATCCATCCAATCACACACCTCCTCCTTTGCACGTAA  2818

Seq_1  2601  ATTGTCAGTACGTAATATTTTTACCTTCCTTCCAACTTTCTTTGCTACCATAGCCTTTCA  2660
                 | |   |      ||    |   |    |  ||   | |    |     |
Seq_2  2819  CCGATGACAGCACCCGGTTCCGCCGAGCGCGACGGCTGGGTCTCGGTCTCGGCCGCGGAC  2878

Seq_1  2661  CTTCTTGAACGCACCAAAAACATCAAATTTGTGTTTCAGAAAATAAGGCCAAACTTTTCT  2720
             |   |   |   |     | |  ||   | |  |   |     |   |   | |  | |
Seq_2  2879  CCGTTCCTAGCTAGTTCCACCCCCAGGGTCGGATCGGAACTTTTCCTGTGGATCCATCCA  2938

Seq_1  2721  TGAGTAATCATCAATAATACTAAGCATGTAATTAGCACCACCAATAGAAGTCTTACAGGA  2780
             |   |             ||  |      | |     |                 | ||
Seq_2  2939  TCCATCCATCCAGGAGGAACACACAGCACAGTGCAGGCGCTTGCGTTGCTCAGGTCGGGT  2998

Seq_1  2781  AGGTCCCCAAACATCGTAATGCACATAATATAATATGCTTTGGTGGTATGAGCGGAAGCA  2840
                    |   | |         | |        | |            ||| |  ||
Seq_2  2999  CTCAGGTCCGCCGTGCCTGCCTGCCTGCATGCTGAAGTCCGATGCAAGCGAGAGCGAGAG  3058

Seq_1  2841  ATGAACTTAACCCTTTTGTGCTTACCATAAATGCAATGCTCACAAAACTCAAACTTACTC  2900
             |  |||       |||  | |   |     ||    |||||| |  ||     |  | |
Seq_2  3059  AGCAACAGCTTATTTTCTTTCCCTCGCGTTATTGCTTGCTCAGAGTACCACTTCGAAGTG  3118

Seq_1  2901  AAGTTGCAGCCATCGATCAGCTCTCTCTTAACCTATTCTGACATGCCACGTTCACTCATA  2960
                 || ||    |      ||        |       |       |     |||
Seq_2  3119  GCTGTGAAGTTTCCTTCTGCCTTGGCAAATAAAATCAATCTGCGTGCGTACGCACCGGGT  3178

Seq_1  2961  TGCCCATGACGCATATGCCACAGATTCGTTTTAGCACAATTATCAGAATCATTAGGTGTA  3020
               ||      |      |          |       |   |  |      |       |
Seq_2  3179  GACCGGGCCGGTCAGAACTCATTCGCGTTGAAGCATCGCCTTCCTTCCAAAGCCTACCTC  3238

Seq_1  3021  ACAGCAGCAGCAAAACCAGGTAAAGTGCTAGTAAGAACATACAATTTGGCAGAATTCATA  3080
              |  |        || ||     | |            | ||   |   |
Seq_2  3239  TCGCCTTTGATGGAAGCAAAGCGATTCGATCCGTTCGTAAACCGGTAAACGTCGACACGC  3298

Seq_1  3081  TCACCTATCATGTGAATAAGAGAACCTCTTGCTACTTTCAAAAGTCTACGCCCTCCGGCT  3140
             |      |    | |   |||| |  |     | ||  |||  |    |   |      |
Seq_2  3299  TTCAGAGTAGGATTACGTAGAGCAGGTGGATTTCCTACCAAGGGAACGCTGTCAGTCTGT  3358

Seq_1  3141  ATTTGTACCCTTCATAATCTATGGTACATAGCGAGATCAAATTTCTTGCCATACTTGGTA  3200
                   |        | | | |||         |      | ||  |      |   | |
Seq_2  3359  CACACCATGGGAGGGAGTTTTTGGGCTGACCGTAACCTTGAATTAGTCAGTCTCCATGCA  3418

Seq_1  3201  TGTGGTTCACCTCATTCAACGTGCGTGTCATGCCATCATGCGTCTTGATCTGAATAGACC  3260
             |||      |     |  | |||   | |   |  |    |||     | |       |
Seq_2  3419  TGTCCGATGCTCTTATATATGTGACCGACCATCTCTTGCTCGTGGAATTGTATTGCCTCT  3478

Seq_1  3261  CAACGCCAACAATATGAATGGGATTGTCATTGCCCACACGCACAAACTCATAACAACTGA  3320
```

```
                ||     |  |     | |  |      | |              |  ||  |  ||
Seq_2  3479  GCTCGTGGGAATCAGATGGGAGGATTAGGGCGACTGGGGATCAGGGGACGAAATTAGAGA  3538

Seq_1  3321  ACCAATCTTTGTTACAACAAATATGAAATGAACATGCAGCATCAAGAATCCATTCATCAT  3380
                ||| | | ||  |   | | | ||      |   || ||    | |  | |     |
Seq_2  3539  CTTAATTTATTTTTAAGTGATTTTTAATGAGCTAATTAGTATATTAAGTAAAATTTATTT  3598

Seq_1  3381  CATGACAAGCACAAGCAGCAAGAACAACGAGGCATTCACCCTCAGAGCTGTCGGTAGAAA  3440
               | |  ||  |||    |     |       |   | || ||         |   |  |
Seq_2  3599  TCTTAGGAGGGCAAAGCCCCCCGCCGCTCCCCCCACCCCCTTCTCCATCCCTGAATGTGA  3658

Seq_1  3441  CGACAACAGCCTTACCAGCACCGTCGGACTTGTCTTTCGGTTTGTACGTACCGTTCCTTT  3500
                           | |    | |   |      || |  | |           | ||
Seq_2  3659  GATGTGACCAACATCAAAATTCTTGTTATAAACATTACTTTCTCAGTTCCATAATTCTAC  3718

Seq_1  3501  TATCCTTGTTCTGCAGTTTATAACAATCCTCAATAACATTCTTTTTTTTCTTACAATACC  3560
                    | |    ||      | ||  ||     | |  |   | |   | |
Seq_2  3719  AGAGACAGGTAGATAGGGCTACAAAACTCTTCGACAAAAACAAATCTAAATAAGTTCTTA  3778

Seq_1  3561  TGCGTAACTTATCTTTTTGCCTGTACTTTGAACGAGCTTTCCCGTCCCTGCTCTTATCTA  3620
                      | |  ||||| ||  | |   |      |        ||  || |  |  |
Seq_2  3779  CCGAACTTATCTAGTTTTGTCTTAAGTCAAACTTGTTTAGGTTTGACCAACTTTGTTGAA  3838

Seq_1  3621  TGTTATTGCAGTTGTTGTTTCTCTGCTCGGTCATCCCACA--------------------  3660
                  | ||      | |  |        |||||   |
Seq_2  3839  AAGAGTAGCGACACATATGACATCAAATCGTCATATTATGAAACTACATTTCATAATTGA  3898

Seq_1  3661  ------------------------------------------------------------  3660

Seq_2  3899  TCTAATGATGTTAATTTTGTGTCATAAATGTTGTTACGTTTTTCCATAAAATTGGTCAAC  3958

Seq_1  3661  ------------------------------------------------------------  3660

Seq_2  3959  TTCAACATCTTTGACTTATGACAAACCTATAAGTACACTTATTTTGCTCGGTCATGCCAC  4018

Seq_1  3661  -AACCTGCAGAGCTTCAGCCTTGGAAGATGACCCATCGGTCTGCACCATAGATTTCATCT  3719
              |||||||||||||||||||||||||||||||||||||||||||||||||||||||||||
Seq_2  4019  GAACCTGCAGAGCTTCAGCCTTGGAAGATGACCCATCGGTCTGCACCATAGATTTCATCT  4078

Seq_1  3720  TCTCTTTCTGTCTAAGTGCCTCTTGGACTTCAACAAGGGTTAGTGTATCATGGCTCAAAA  3779
             |||||||||||||||||||||||||||
Seq_2  4079  TCTCTTTCTGTCTAAGTGCCTCTTGGA---------------------------------  4105

Seq_1  3780  GAATTGAATCATGGAAATTTGTAAAGGAACTATGCAACGAGCATAAGAGTAAAAGGCTAA  3839

Seq_2  4106  ------------------------------------------------------------  4105

Seq_1  3840  ATTCTCATCATCATATATTACTTCCAAAGACCGCAAGTCAGAAATGATCTCTCTAAAGGC  3899

Seq_2  4106  ------------------------------------------------------------  4105

Seq_1  3900  AGCCATATGAGATATTTCTGATCCACCTTCTTGGAGCTTATGTAAGAACAACTTCATCTT  3959
                                                |||||||||||||||||||||||||
Seq_2  4106  -----------------------------------GCTTATGTAAGAACAACTTCATCTT  4130

Seq_1  3960  ACTTGCATCTTATTGGTTAGATCCTTTGTCATGCAGATCGATTCTAGTTTCACCCACATG  4019
```

```
                ||||||||||||||||||||||||||||||||||||||||||||| ||||||| |||||||
Seq_2   4131    ACTTGCATCTTATTGGTTAGATCCTTTGTCATGCAGATCGATTCCAGTTTCAGCCACATG   4190


Seq_1   4020    GCTGCTGCGGATTTCTCCTCCAACACTTCCTGCAAAATAGTATTGGACAAATGAAGTTGA   4079
                ||||||||||||||||||||||||||||||||||||||||||||||||||||||||||||
Seq_2   4191    GCTGCTGCGGATTTCTCCTCCAACACTTCCTGCAAAATAGTATTGGACAAATGAAGTTGA   4250


Seq_1   4080    ATCAAAGACAAAGCCTTACCATCTTTTTGTTTCTCTTCGGCAGTCCACTCTTTTGCATCC   4139
                ||||||||||||||||||| ||||||||||||||||||||||||||||||||||||||||
Seq_2   4251    ATCAAAGACAAAGCCTTACGATCTTTTTGTTTCTCTTCGGCAGTCCACTCTTTTGCATCC   4310


Seq_1   4140    TTCTTTCCGAAAGAATAAAGCGCTTCATCTAGATCTGAAGTTTGGACGAGAATCGCACGT   4199
                ||||||||||||||||||||||||||||||||||||||||||||| ||||||||||||||
Seq_2   4311    TTCTTTCCGAAAGAATAAAGCGCTTCATCTAGATCTGAAGTTTGGGCGAGAATCGCACGT   4370


Seq_1   4200    ATCTTCACTTGCCACAACGAGAACCGTGTGGTGTAA-TTCAGCTGCAGCAGATCGAACTC   4258
                |||||||||||||||||||||||||||||||||||  |                 |  ||
Seq_2   4371    ATCTTCACTTGCCACAACGAGAACCGTGTGGTGTAGTTCAGCTGCGGCAGATCGAACTTC   4430


Seq_1   4259    AAAGTAGACATGTCGCAAACTCTAGGCAGAACCTGGTGTTCTGATACCACTTGTTATGAA   4318
                || ||||||||||||||||| |||||| ||||||||||||||||||||||||||||||||
Seq_2   4431    AAGGTAGACATGTCGCAAACCCTAGGCGGAACCTGGTGTTCTGATACCACTTGTTATGAA   4490


Seq_1   4319    CGCGACAAGCGAGAACGAAAAAGAAATCACAGCACAAGTACACACGGATTTAACGTGAAA   4378
                |||||||||||||||||||||| |||||||||||||||||||||||||||||||||||||
Seq_2   4491    CGCGACAAGCGAGAACGAAAAA-AAATCACAGCACAAGTACACACGGATTTAACGTGAAA   4549


Seq_1   4379    AAACCTCAACCAATGAGAGAAAAACCACGGACGTCAGACAGCGAAACTTCACTATATGGA   4438
                ||||||||||||||||||||||||||||||||||||| || |||||||||||||||||||
Seq_2   4550    AAACCTCAACCAATGAGAGAAAAACCACGGACGTCAGCCAACGAAACTTCACTATATGGA   4609


Seq_1   4439    GAGTATTTACACAACGGCAGGGATGTTTCACGAATGCAACTCATCCCGAAACGACGGCTT   4498
                |||| |||||||||||||||||||||||||||||||||||||||||||||| ||||||
Seq_2   4610    GAGTGTTTACACAACGGCAGGGATGTTTCACGAATGCAACTCATCCCGAAACGGCGGCTT   4669


Seq_1   4499    ACAAGGGGTATATATAGCAGGAACATTAGGTTAAAA-TAGATCCGTATCGGTGCATCCGA   4557
                |||||||||||||||||||| | ||||||||||||| |||||||||||||||||| ||||
Seq_2   4670    ACAAGGGGTATATATAGCAGTAGCATTAGGTTAAAATTAGATCCGTATCGGTGCACCCGA   4729


Seq_1   4558    AAATTCCGGGTGCACGACCGGAAGTTCCGGGTGCGTGGCCGGAAGTTTCACAAATTTCTC   4617
                || ||| |||||||||||||||||||
Seq_2   4730    AATTTCTGGGTGCACGACCGGAAGTT----------------------------------   4755


Seq_1   4618    GAAGAGTAACCGAGATTCACAAATTCCTCGAAGAGTAACCGAGAGTATCCCGAGGAACAA   4677
                                ||||||||||||| |||||||||||||||||||| |||||||||
Seq_2   4756    ----------------TCACAAATTCCTCAAAGAGTAACCGAGAGTATCCTGAGGAACAA   4799


Seq_1   4678    CCGGACTTACGGTGGAAGTTTTGTTCCTGGGAAAGCCTTGGCAATGAATATGGATCACAA   4737
                ||||||||||||||||||||| |||| ||| |||||||||||||||||||||||||||||
Seq_2   4800    CCGGACTTACGGTGGAAGTTTCGTTCATGGAAAAGCCTTGGCAATGAATATGGATCACAA   4859


Seq_1   4738    TTTCAACGCTAACATCGTTCCGCTGGCAAGGTTGTATCCGCCCTCCTGTCTTGTACGGAG   4797
                ||||||| ||||||||||||||||||||||||||||||||||||||| |||||||||||
Seq_2   4860    TTTCAACACTAACATCGTTCCGCTGGCAAGGTTGTATCCGCCCTCCTATCTTGTACGGAA   4919


Seq_1   4798    TACACCGTAAAGACACCTGATGCCAATCACAATTGAGCCAGATGGTAGATTGTCAAAAGA   4857
```

```
                |||||||||||||||||||||||||||||||||||||||||||||||||||||||||||||
Seq_2  4920  TACACCGTAAAGACACCTGATGCCAATCACAATTGAGCCAGATGGTAGATTGTCAAAAGA  4979


Seq_1  4858  ATCTAAAGAAACCGCATTTCTCATTAGTTGGTCTTTAAGAATTTCTCTTTCACGCAATAT  4917
             ||||||||||||||||||||||||||||||||||||||||||||||||||||||||||||
Seq_2  4980  ATCTAAAGAAACCGCATTTCTCATTAGTTGGTCTTTAAGAATTTCTCTTTCACGCAATAT  5039


Seq_1  4918  TTCCTTCATTTTGAGAATCTGAAGACTT-AACCTGCTCCTTCCTTGCTGAGACCCGCGTA  4976
             |||||||||||||||||||||||||||| |||||||| ||||||||||||||||||||||
Seq_2  5040  TTCCTTCATTTTGAGAATCTGAAGACTTTAACCTGCT-CTTCCTTGCTGAGACCCGCGTA  5098


Seq_1  4977  ATCATTGCTTTGTGCTAGAACCTACTCCTGCTTTCTAGAAAGACAATATAAAAAAAAA-C  5035
             |||||||||||||||||||||||||||||||||||||||||||||||||||||||||| |
Seq_2  5099  ATCATTGCTTTGTGCTAGAACCTACTCCTGCTTTCTAGAAAGACAATATAAAAAAAAAAC  5158


Seq_1  5036  TGTAGTGCAAGAACGGAAAGGAAATTCCACTCGTATGAGCGAGTGAAGGTGGTGTTGCAA  5095
             ||||||||||||||||||||||||||||||||||||||||||||||||||||||||||||
Seq_2  5159  TGTAGTGCAAGAACGGAAAGGAAATTCCACTCGTATGAGCGAGTGAAGGTGGTGTTGCAA  5218


Seq_1  5096  ACTCTACCTACAATACAAGCGGGTCAGACTAGGTGAAGCTGACCGATATATATTAGCTGT  5155
             ||||||||||||||||||||||||||||||||||||||||||||||||||||||||||||
Seq_2  5219  ACTCTACCTACAATACAAGCGGGTCAGACTAGGTGAAGCTGACCGATATATATTAGCTGT  5278


Seq_1  5156  CTGGAAACACGTTTATACTACTAATATATAGCATAAACTCTATCAAAGAATATGAAGTAG  5215
             ||||||||||||||||||||||||||||||||||||||||||||||||||||||||||||
Seq_2  5279  CTGGAAACACGTTTATACTACTAATATATAGCATAAACTCTATCAAAGAATATGAAGTAG  5338


Seq_1  5216  TATATCACACAACTACTGGGCAGTCACATGCATTCTTGCAATGAATAGTATAGATCCAAC  5275
             ||||||||||||||||||||||||||||||||||||||||||||||||||||||||||||
Seq_2  5339  TATATCACACAACTACTGGGCAGTCACATGCATTCTTGCAATGAATAGTATAGATCCAAC  5398


Seq_1  5276  GGTCTTGCAATCATCTCCCACATTTTTAAGTGATTTCATGATTGTGCAACCAAATACATA  5335
             ||||||||||||||||||||||||||||||||||||||||||||||||||||||||||||
Seq_2  5399  GGTCTTGCAATCATCTCCCACATTTTTAAGTGATTTCATGATTGTGCAACCAAATACATA  5458


Seq_1  5336  CAGATTCTGTGACTCTAGAAGCAAATATTCTACATAGGGATTATTCAGAGTTGTTTTTTC  5395
             ||||||||||||||||||||||||||||||||||||||||||||||||||||||||||||
Seq_2  5459  CAGATTCTGTGACTCTAGAAGCAAATATTCTACATAGGGATTATTCAGAGTTGTTTTTTC  5518


Seq_1  5396  TTAGAATTATTCAGAGTTGTTGACGTAAAGAACTTTTGCAAATACAAAAGGTTGGCCAGA  5455
             ||||||||||||||||||||||||||||||||||||||||||||||||| ||||||||||
Seq_2  5519  TTAGAATTATTCAGAGTTGTTGACGTAAAGAACTTTTGCAAATACAAAAAGTTGGCCAGA  5578


Seq_1  5456  ATAAAAAAAACAAAGAACGAGGTGTAGTCCTGTTCCCGATTGTACTCAACAGTAGGATAG  5515
             ||||||||||||||||||||||||||||||||||||||||||||||||||||||||||||
Seq_2  5579  ATAAAAAAAACAAAGAACGAGGTGTAGTCCTGTTCCCGATTGTACTCAACAGTAGGATAG  5638


Seq_1  5516  GAGGGCCTTTCTTTTCCCTTTTTGAAAGTTAAGCAACGGAAGGAACGAAGGAGAAGGAAG  5575
             ||||||||||||||||||||||||||||||||||||||||||||||||||||||||||||
Seq_2  5639  GAGGGCCTTTCTTTTCCCTTTTTGAAAGTTAAGCAACGGAAGGAACGAAGGAGAAGGAAG  5698


Seq_1  5576  GATTAACCGATGGATATGGATGGATGTAGCCCGAAGACGAGGTGGCTGTGTCTGAGTTGG  5635
             ||||||||||||||||||||||||||||||||||||||||||||||||||||||||||||
Seq_2  5699  GATTAACCGATGGATATGGATGGATGTAGCCCGAAGACGAGGTGGCTGTGTCTGAGTTGG  5758


Seq_1  5636  CTGCCCTTGCTCCATCAGTCCCAACGGAATATCACGTTGGTGTAGCGTAACGCCGAGCCC  5695
```

```
          ||||||||||||||||||||||||||||||||||||||||||||||||||||||||||||
Seq_2  5759  CTGCCCTTGCTCCATCAGTCCCAACGGAATATCACGTTGGTGTAGCGTAACGCCGAGCCC  5818

Seq_1  5696  CATACGTGACGTGATGCATGCACCAGCTGTGTACAGCTAACACCGCCACGCGTTCCCATG  5755
             ||||||||||||||||||||||||||||||||||||||||||||||||||||||||||||
Seq_2  5819  CATACGTGACGTGATGCATGCACCAGCTGTGTACAGCTAACACCGCCACGCGTTCCCATG  5878

Seq_1  5756  CAACCCCCGGGAAACCAAAACCTGACAAAAAATATGAAGAAATTGCGTGGCCGGAACCTG  5815
             ||||||||||||||||||||||||||||||||||||||||||||||||||||||||||||
Seq_2  5879  CAACCCCCGGGAAACCAAAACCTGACAAAAAATATGAAGAAATTGCGTGGCCGGAACCTG  5938

Seq_1  5816  ACTGCGGCCGCCAACCGCGCCACGCAGGTTCGTTGGTTTCGGTGGCACCGGGTTGGGTTG  5875
             ||||||||||||||||||||||||||||||||||||||||||||||||||||||||||||
Seq_2  5939  ACTGCGGCCGCCAACCGCGCCACGCAGGTTCGTTGGTTTCGGTGGCACCGGGTTGGGTTG  5998

Seq_1  5876  GGTTGGGTTGCCAGTCCATGTCCAGCAGTCCAGGCGGTCAGCGGCGCAGCCAGTTTCCCT  5935
             ||||||||||||||||||||||||||||||||||||||||||||||||||||||||||||
Seq_2  5999  GGTTGGGTTGCCAGTCCATGTCCAGCAGTCCAGGCGGTCAGCGGCGCAGCCAGTTTCCCT  6058

Seq_1  5936  TCCCTCGCAAGCCAGCCAGCCAGCCTCCCTCACCAGAACAGGACGCGGTTTTATTAGCAG  5995
             ||||||||||||||||||||||||||||||||||||||||||||||||||||||||||||
Seq_2  6059  TCCCTCGCAAGCCAGCCAGCCAGCCTCCCTCACCAGAACAGGACGCGGTTTTATTAGCAG  6118

Seq_1  5996  AGCAAGCAAAGGAATTCCTACCCCTCCACATTTAAAAGACAGACGCCACCGAAACACCCC  6055
             ||||||||||||||||||||||||||||||||||||||||||||||||||||||||||||
Seq_2  6119  AGCAAGCAAAGGAATTCCTACCCCTCCACATTTAAAAGACAGACGCCACCGAAACACCCC  6178

Seq_1  6056  CACAACTCCATCCAACCCTGCTGAGGCCACAGCGGTTCCACGCAAAACTCTTTGGTGCTG  6115
             ||||||||||||||||||||||||||||||||||||||||||||||||||||||||||||
Seq_2  6179  CACAACTCCATCCAACCCTGCTGAGGCCACAGCGGTTCCACGCAAAACTCTTTGGTGCTG  6238

Seq_1  6116  AAAGCTCTTTCGCTCGCTCTTGGCTTTGCTTTCCCTGGTTCCTGGTCGACTGGCTGAGTG  6175
             ||||||||||||||||||||||||||||||||||||||||||||||||||||||||||||
Seq_2  6239  AAAGCTCTTTCGCTCGCTCTTGGCTTTGCTTTCCCTGGTTCCTGGTCGACTGGCTGAGTG  6298

Seq_1  6176  GCTGGCTAGCGGGC  6189
             ||||||||||||||
Seq_2  6299  GCTGGCTAGCGGGC  6312
```

BdindelWSU_8, upstream

>Bradi3g00757

TGCAACACCCCCAGCCATGAGTTCTGTCAATAGCTTCAGTCACCACCCTCTTCAGAAGAGATGCCGCGTCAACAAGCTGTCGTCGAGC
CTTAGGCTTCTGCAAGCCCGCCCAGTAGTCCAGCCAATGACAGATAAAGATAACCAGATTAGAAGGATCACTAGGAAATATACATTG
AAAACAAGCATTATTACGCGCCTTCCAGATAGACCAACAAAGTGCGGCTGTTCCTATTGCCGCTGCAGGGCGAATAGAAACCTAAAA
TTTGCTGGCTTTGCAGTCTTTCTCCATGGGAAAGAAGCAGGTTGGTAAACTCTGCTTCGGATTCAGCCAATGCTTGATGCAGTTGAAT
GAATTCATGTTGTTACCTTTGGGGTTGTGAAACCCAAGGAGTTTCCTTTCGCCCAATGCAGACGGTGGCGGCATCCTATCGTCGCATT
CCCACGGCCGGTGAGAGCTGCTGAAGTTCCCAAAATTCCTTAAAGACAAGATATCGATTTATATGATTGAAAGAACTCCTAAAATTCC
CATGAAACAAGTTGCAAGAATTCTGGAACTCCCATGAACTTCTACAGTTTGCGAAAGAACTTCCAGATTTTTTTAGAGAACTTTGAAAT
TTTGTGAGAGAGCTTTCGAAGTGGGGAAACTTTCAACTAAATTTACACACTTCCAACAAATTCCCATGATGCAAAAAAGATTTCCAGG
TGATTAATTGGCAACTTCTTATAATCTTCCAAAAAAAGGTTTAACGAAAACTTATTCCTCGCAAGACATAAGGAGAAAATTTCCCCATT
TACTCTAGTCTGCTTTTATTTTGACAGCCCCATTTACTCTAGTCTACTTCACTGTACAGCTATTATACGGCAGCAAGGCAGATAACACCA
AAACAAATCTGCCCCTTTCCCTCGCCACCGGCGCCGCCGAGGCCTCAATTTCCCCAAGCCGCGCCGGGTTCACCATGGAGGAATACCT

CGTCGACGCCTGCGGCCTGACCCGAGCCCAGGCGCTCAAGGCCTCCGCCAAGCTATCACACCTCAAGCCCCCCTCCAACCCCGACGCC
GTCGTCGCCTTCCTCTCCGGCGGCCTCGGCCTCTCCATCGCCGACATCGCCGCCGTCGTCGCCAAGGACCCCAGGTTCCTCTGCGCCA
GCGTGAAGAAAACCCTGGCGCCCAACGTCGCCGCGCTCACCGCCGGCGCCGGCGGCCTGTCGCGTCCCGAGGTCGCCAGCCTCGTC
GTCCTCGCCCCCAGCCACTTCCGCCGCTGGTCCATCGTCTCCAAGCTGCGCTACTACCTGCGCCTCCTGGGCTCCGCCGTGGACCTCCT
CCGGACTCTCAGGAAGAACTCCCGCCTCCTCTCGGCCGACCTCGACGCCGTCGTCAGGCCCAACGCCGCGTTCCTGCGGGAGCAGTG
CGGGCTAGGTGCTCGCGACATTGCCAAGCTGTGCAACCCCCTGCCGCTGCTGCTCTCGGCCGACCCGGAGCGCGTCCGGGCGATGG
CGGCTTGCGCCGAGGGCGTCGTGGGCGTGCCCCGCGGCTCCGGGATGTTCAGGCCGTCGCGTTCCTCAGCGAGAAGAAGACCGCCG
CCAAAGTCGAGAACTTGAAGGTGACGTTCGGGTGGTCGGACGACGAGGTGGCTGTTGCCGTGTCCAAGGCTCCGATGGTGCTGACG
AATTCCAAGGACATGCTGCGGCGCAGGGCAGAGTTCCTGATCTCCGAGGTGGGGTTGGAACCGGCGTACATTGCTCGCCGGCCCGT
CATGCTCAGTTATAGCTTGGAGGGCCGGCTCAAACCCCGGTACTGCGTCATAAAGTTTCTCAAGGAACATGGGTTGCTGGATCATGA
CAGGGACTACTACAAAACAGTCATGGTCAGCGAGAAGGTGTTCATGGAGAAGTTCATATGCCCTCACAGCGAAGCTGCGCCGCACCT
TGCCGAAGCCTATGATGCCGCTTGCAGAGCGGAAGTGCCTGCTAATTTTTGGCTTACATGAACCAAGAATGGACCATTGGAATTGGT
AACTACGTATGGCATGACAAAGTTATCACTCTGTTTAGTATGTTGCATGTTGATTTGGTATTTGCTAGCCCTGGATGAAACTGATAAAT
TGATTAGTGAGTCCAGGTATGTCGATTCATCATTCAGTAGAGAAGAAAGCAGAGAATGATATGTAATTCCATCCTCATGCCATATTGA
TAGACATGTACTTGACATGTTGCAACTTAAAGAGTGTGAGGTCAGGCCATTCTGCAAATGTGTTGAAGTATCAGTGAGTTGTATACTA
TATCTGCACACTTGATGTCCTTATGTTCTCCAATGGCCATTGCAGTTTTCAGGTCAATCCTTAATCATTTTAGTCTGCATAACCTGCCAG
GATTCAGAGAAGGAAAGCTTGAGAGAGTCGTGTTTTTCCTACGGAGTATGTTATATCTGCAGTTCTGATTGATTTACGGCTTCAGCCC
ATTTGGCATTTGTCTCCTCAGTTATGGCATTTCCATGTTAACATCTGAAAACCTTTTAGCTAATTTCTTAAGACATGTGCGCTGGAATTG
TATTTTACCTTGTACAACTTGTTGAATCTTATATGGGTAAAATTTTGCACTTACAAAAAACCATACACGTTAACAAATTATTGTTACCTA
GTAGTGTCATATATAGTGGCAGCATCATGCAAACACATACTGCAATGAGAGAACTGTAAACCATACCAAAAGTATAAAAATACATCA
GTCAATGTCGATGAGCCAAAATTTCATCTGTCGCTAGTCAGAGACTCAGAGTTTAGCGCAGCAGAAACATTTTACAGGTCTGCAAGA
GTGACAAAACTTATTGTTTTCCCATTCCTTCGGGACTATTTTGCAGCAAAAGATCCACCAGGGCTAGCCTTTAATCTTCCTCGTGTGGT
GAAAATGGCAGAGGTAGTTAGATAGGGACACCATAATTCACAATCTTCGTGAGGGCAGAGTTAAGCACACGCTTCTCCTTCTTGACC
TGGGAGGCTAGACCAGAAATCTGCAAGGAAAAGAAAAGACAGTAGTGACACGAATTTATGTTAGAGGATATTAGGTCAGGGCCATC
AATGGTTTGAGAAGATGGGTGCAATCTACTGTGGCCGGCTGAAGCACCTAGTTTGGAAAGTACAGGTTGCTACTAGTGGGCTGTGG
GCACATTTAGTAGAAGGCTACATCCTGATTTTATTTGCTGTACCTAGTTGAAGAGGTGGATAGTAGTACTGTTGCAGATACTAACCTC
TGATCGAAATTCAGATGCATGTGCTCTCGGAAGCTGATCAAGAATTCCTTTGAGACCTAAGAGGGAGCATTTTATTATATGTAAGATT
GAAGACAAGAAATTTCTACACTGAAATTCAGAGTCATAAGATAAAGCAGTGATCTCTCAAGGAATTGCTACACACCTACTACTTGGTT
TTCAATATCACTAATCTGCTTCAGCTCACTTTGAATGGCGCGCCCTTCTTCCCTGAGAAAGTATACATTCACCCCTATGTTATATTAAGC
CCCAAAGTTAACAGGTCGAAACAATGAAAAACATGCCTGAGTGTATTCCATCCTTTCTGGAATCTTTTTTCTGCCGTTGCTGCTCTATC
CTTGATTCCATATGCAATAATTTTAACCATGAGTAACATTAGTTCCCAAGTTGAAATAAATGTCTCCGCTTTCTAAATATACATACAGAA
AGTTACCATCAATTTTTGGCTTTCATTGGATACAAGATTGACAGTTTGCCGCATGTGATTCACTTCAGCTTGGATGCCGGAAAGTAGG
GTCTACAATATGGAAACTTGTGTTAGACAACAAGTCTATTTATCTTTGTCATTTCTAAGGAAAATCAACCATGCTTTTGAAAACTGTAT
GTAAAATAAATGAACTTCAGAAAACTGTTTCATTTTTGTTCTAAAAGGTACAAACAGAAGGAATCGTATCAGAACATGTAAATAGTAA
AACATGATGTACAATCAGTACAGAGTTAGCCACCTCGTAAGAGATGACAAGTTATAAGAAAAATGGTTGATGACTTGATGTAGCTAC
TGGAACATGAAGCTAGCAGATAGCATAGTCAAGGAGTCTCGCTAAAGTATGAACTAATTAAACTGACACATAGTGCTTACCTCCTTGC
TGACAAATATTCGTCGTACACGTTGTATTAAGTAGCTCCTTGGCCCTGGAATGACTCATTGTCAGCCATACTAAATTTAGCAGTATATA
AGAAAAACAATTTTTCAACATTCATGGAGCAAACTTCCACTGAACCAACAACCTTGTAATTCAGTACCAAGAAGTGCTAGGCATCTAT
GTCTACAGATTAGAAGTCGGATAATTTGAATTTCTTAGCAGCTTTAATGTTATTCGCCTAATCCGATATTCTAAGAACAATGCAATCAG
TTAATTTGAACCTACAGGAAAAGACGATCTGTGCATGACAATTTAATTAGGATGCAGGTAACTGGTTCGTGGAGGTCTCAAGATATA
CCAATCAACTGTAAGAAATGAGCAATAAATTTTTTAAATTCCGATATAAGGTCCATCGTGCGGCACTAAGAACAAAAACTCATTATCC
TCTACAAGAGCATTATTTCGCAAAGACTTAGACATGCAAGTAGCGTATTTCTTTGTGAGTAACAAACAAGAACCAACTAAATACTAAT
ACAAACAGCTTGAACTTATCAAACATAAGGCGTCGCCACAAAGCAAATGAGTTCAGACGAAATGGCATTACTTTTGAGCAGCACAAT
TCCTGCAACTGTGGCAGAGCCTGCGGCAATGCCTGGATGCTCGATTGCCATGATGACACCCTCTGCAAAGACAGCAAATTGAGTTTC
AAATAATTGATTACAAAATATACAGCCACACCAATGTGGCACTGAAAGTCATCAATCAGACATACATATATGAACAATGCTACATATC
AATCCATAGATACCCCCCAAACAATTAGACAACAAACACCAAAAGGCGCACCTTTGATCTTGCCCAGCGCCATCTGCTCACGAGCCAA
GTACTCCTTCTTGACCCATTCCAGCTCTTCCTACAATTAAACAACCAAGTTGGAGCTTGAGCGAATTTCGGTGTGACCGAATCGGAAG
AGTGAGGGGAATGGATGGTTCCAGAAGAGGGGGGGCACCTTCGCGGCGTTGGTGAGGGTGGGGAAGGCGGCGAGGGCGGCGGC
CATGGCATCGTTGGCGGCATCAGAAGCGGCGATGGCGGACGAGTGCGCGTGGGCGGTGGCCGTGTCGACGGTGGCCCTGGCGGCA

GCGACCATGTCGGCGCTGAATGCCTCACCCCGCCGCGTGAAATCCGCGGCGGCCGCCGCCGCGGAGGTCGCGGACGAGGCGACCAT
GGAGGAGATGGATCCGCCTTCGCTCTCCGGCTGCGGCTGCGGCGGCGGGGAGCCGCCGGGGGAGGAGGAGGAAGCGGAGGGCG
CGTCGGCCATGGCGGTAGGATCCAGAGAGAGGGAGAGGGGGGCAGAGGAGAGAGACCTATTTCCGGGAAATGAGATAAAAAAAG
AAGAAGCTTTCGCGTATTCCATGATTCCGCTTTCATCAAGTGAAAACGTTTTCGTTCAACGAGGCAACACGGTTGACAGACTTGACGT
GTTCCATAAGCTATAGCGGAGAGTTTATGCCCGGCAACTAGTGGAGGAGATCGAGGCCGAAAATGGAAATGTCTTGGAACGATGCA
AAAGGAAAAATGCCTTCGGATTCAATTTAAGTGTTCAAACGAGGAGCACATGTTTTGGGTTGTGGAACATGTGAAAAAACAGACGAA
AAATCTCCGAAAACAAAAAGACCTCGCGCCAATGTTTTCATATCACACATTTTGATGGCGACCATATATAAGCTGATTTCACTTTTTCG
AGATATTCTACATGACGAGGAGGAGGAGATATTCTACCGGGAGATATTCGACGGTCCTGAAGAAAATTGATTGTGGTTCTGTCTACT
TGTCTCAGCGCCTGCGTCCTCTGTCCTCTACTCCGGCGCATGCACAGTGAGTAGTGACAGTGCGCAATTAAGGAGCGATGGTACCAG
GCATGTGCACAGCCAGCTCACTGTGGAAGCCAATAATTCAGCAGGCTGTACTGGGAGCTAGTAAGCTACTGTAGAAGCCAATAATTC
AGCAGCTTCTCTGTTCGTTCCTTCTCACAATCTCACCCGGCCGTTTCCTCCACCGCATCCGGACCTTAACCATCCTCTTCCCTTCTTCGTT
CCATTCTCCTCCTGCCGTCATGAGGAACAGAGATTCAGACTTGAGAGATCTGTTGTGATCCTGCCTGCGCGACAAAGATTCAGACTTG
AGGTACGTGCGATGTTACGTTAGAGCGTATGGATCTCAGAGATTTTCTCAGCCTTCAAGTTGTTTTTAGAGCGTATGAATCTCAGCAG
TTTCTTCAGCCTTTCAAGTTGTTTTTAGAGTGTATGAATCTCAGCAGTCTCTTTTTAAAACTGTATGGGTATGTCCTAGAGTGTATGTAT
GGACCTTCAAGTGCTTTCCTACATACTATTTCATTCGATCCAAGAGGCTATGGCTATCTAAATCCATCAGGAGAGTATTAAATTTACAG
TACCTCTTCCTCTGTCTACCCAAACCCCCACCACCCCTGCCTTCTTTCGCGCCCGGCGTCAGATCTCGCGCGTCTAATCGTGTAGTGATA
ATTGATTCAAGTTGCATGGCTGTTCCTGTTCACATGGTAGACGGATGGCAATAAGCTCCAGTATGAAGAGTCCATGATGGATCGATTC
ACTGTAAAAGAAAATGCTTGATGCATGGATCGATTTACTATCTTCCATTCCATGGGCAAGGCAATCGCAGGCTGCAGGCCTTGCTCTC
TTTCCAATTCCATCCAGATCTGCCATGGCACAGCACACGCCAACTGCCGAGGAGGGCTGCGGCAGTGATGAGAAAGAAAAAATAAAT
CTGATTTCCTTTTAAAAAATTTACCAACGTGAAAGTTCATAATACCCTTTTCAAATTGGGAGGGGGTTTATACAGAAGCAGACCTCAAA
TCTTAATTTGGTCTCTCGGTCTCTTCTTGCCTTTGCAGCACAAATTTCTAAAGCCAGCCATGAATGCACTCATGGTTTACTCCCTGTGTA
CACAACATATGTATAGTAGGCAAGGTGTCATCTAAAATCACAAAACCATTGGGATTTCGTATTCAAAACACTTCCGTACAGCTAGAGC
ATCTCCACTCACGTCCCCCCATTCGGCGTCTGGCGCTGCCGTTTTGTAGGCCGGATGGGGGGCACAAATATTTTAATCTGGGGATCCG
CAGCCCTGCCCCCAATACCAAATCCAAAATAAATAAATCCAAAAAGGGATAAATAAATTTAAAAAGGGATAATTTAACTAGTTGGGG
CGAAATTTATACAAAATTCTAAAAAAACAACTAATCTACTCGTCGTCGTCCCACTCCTCCCCATCCCCGTCCTTGTCGAGCTCGCTGGT
GAGGCCAAGGCAGCGCGTGCGTTCGGCTTACTCCTTCTTGTGCTCCCTCGCCGACCAGGCGCGGAAATGGTCAAAGTTTGACATAAA
ATTCCGCGTAGGCCTTACAAATCTGGACGAGGGAGTATCTTGTTACTACGAGTCTAGGACTAACACTATTGTGCAAAAAGTATTGTTG
TCAAAGCTGCTTACTGAACTTGGAGTCTAGTTAAAATACTCTTTATCATTTCTAAATGAAGAGAAGAGAGTATTACATAAAAATTCCAA
ATTTAAGAAAAACATATGGGGTAATCCAAGTATATCTACCCCGGCCTATGTTGATTTTGGTTGTATCATCTCATTTAAACTTGCTAGGG
ATACATGCATGCATAGAAGTATGTCGTTTTATAATATGAGATATAGATCGAAAGCTCAAAGCTCTGGTCTATTTAACGCCCACGTTGC
AGGTACCTTACAAGCATAATTAGGATCCAAGATACACAATTGTCCAGCAAGAGATCC

>BdiBd21-3.3G0008500

TTCCAAAAAAAAGGTTTAACGAAAACTTATTCCTCGCAAGACATAAGGAGAAAATTTCCCTATTTACTCTAGTCTGCTTTTATTTTGACA
GCCCCATTTACTCTAGTCTACTTCACTGTACAGCTATTATACGGCAGCAAGGCAGATAACACCAAAACAAATCTGCCCCCTTTCCCTCG
CTGCCGGCGCCGCCGAGGCCTCAATTTCCCCAAGCCGCGCCAGGTTCACCATGGAGGAATACCTCGTCGACGCCTGCGGCCTGACCC
GAGCCCAGGCGCTCAAGGCCTCCGCCAAGCTATCACACCTCAAGCCCCCCTCCAACCCCGACGCCGTCGTCGCCTTCCTCTCCGGCGG
CCTCGGCCTCTACATCGCCGATGTTGGAGAATGCAATGCAAGCTTCTAGAGTGTTCTCAATATTACAAGAAAGAAGACATTACTATGG
AATACTCTAGAATATAAAATAAATAAAAGAGGGAACCTTCTAGTGTTTAGATATGCATGAAGAATTATGTAGAAGCTTTGGAATACTC
TAGAATAGGAGTTTAAAATAAATAAATAGGAAACCTTCTAATACCTAGATATTTATGTAGAAGTGTGTGGAAGGCTTTAGATATTTTG
TATCAATGGCTAAGATTTTGACACATGTCAAAGATCCAATGGCCATGTAGTCTTATAAATAGAGGCCCTTGCCTCACAACTTGTGTAG
TAGAGAATAAGAAAGTGAAGAAGGTGGAGAACAAGGTGTGAGCAAGTGTAAGGTGTGTATGCTCTCTCTTGTACTAATATTCCTAAA
GCAATATAGTACTACTTTGTTCATATAAGTCTCCCGGTTAGGCCTTGTTATTTTTAAGTACTTAGTGCATATAAGTTGTGATTTAACGTG
AGCGGATTCGTCCGGAATCACAACGGTCTTGTTTCAACGTGAGTGGCATAGCCGCCCGGATTCAAGACTTGCTTTAACGTGAGTGGT
TCTGCCGCCCGGAAGCTTGCACTAAGTAAGTAGAAATAAAAAGGGACTAATCAACTACACCAAAGTAGTTGGTATAGGTGAATAGG
GCCTCTCGCAGTGTGTTAGGTCCACCTCGAATTCTCTGACAGCCGACATCGCCGCCGTCGTCGCCAAGGACCCCAGGTTCCTCTGCGC
CAGCGTGAAGAAAACCCTGGCGCCCAACGTCGCCGCGCTCACCGCCGGCGCCGGCGGCCTGTCGCGTCCCGAGGTCGCCAGCCTCG
TCGTCCTCGCCCCCAGCCACTTCCGCCGCCGGTCCATCGTCTCCAAGCTGCGCTACTACCTGCGCCTCCTGGGCTCCGCCGGGGACCTC
CTCCGGACTCTCAGGAAGAACTCCCGCCTCCTCTCGGCCGACCTCGACGCCGTCGTCAGGCCCAACGCCGCGTTCCTGCGGGAGCAG

TGCGGGCTAGGTGCTCGCGACATTGCCAAGCTGTGCAACCCCCTGCCGCTGCTGCTCTCGGCCGACCCGGAGCGCGTCCGGGCGATG
GCGGCTTGCGCCGAGGGCGTCGTGGGCGTGCCCCGCGGCTCCGGGATGTTCAGGCCGTCGCGTTCCTCAGCGAGAAGAAGGCCGCC
GCCAAAGTCGAGTACTTGAAGGTGACGTTCGGGTGGTCGGACGACGAGGTGGCTGTTGCCGTGTCCAAGGCTCCGATGGTGCTGAC
GAATTCCAAGGACATGCTGCGGCGCAGGGCAGAGTTCCTGATCTCCGAGGTGGGTTGGAACCGGCGTACGTTGCTCGCCGGCCCGT
CATGCTCAGTTATAGCTTGGAGGGCCGGCTCAAACCCCGGTACTGCGTCATAAAGTTTCTCAAGGAACATGGGTTGCTGGATCATGA
CAGGGACTACTACAAAACAGTCATGGTCAGCGAGAAGGTGTTCATGGAGAAGTTCATATGCCCTCGCAGCGAAGCTGCGCCGCACCT
TGCTGAAGCCTATGATGCCGCTTGCAGAGCGGAAGTGCCTGCTAATTTTTGGCTTACATGAACCAAGAATGGACCATTGGAATTGGT
AACTACGTATGGCATGACAAAGTTATCACTCTGTTTAGTATGTTGCATGTTGATTTGGTATTTGCTAGCCCTGGATGAAACTGATAAAT
TGATTAGTGAGTCCAGGTATGTCGATTCATCATTCAGTAGAGAAGAAAGCAGAGAATGATATGTAATTCCATCCTCATGCCATATTGA
TAGACATGTGCTTGAGATGTTGCAACTTAAAGAGTGTGAGGTCAGGCCATTCTGCAAATGTGTTGAAGTATCAGTGAGTTGTATACT
ATATCTGCACACTTGATGTCCTTATGTTCTCCAATGGCCATTGCAGTTTTCAGGTCAATCCTTAATCATTTTAGTCTGCATAACCTGCCA
GGATTCAGAGAAGGAAAGCTTGAGAGAGTCGTGTTTTTCCTACGGAGTATGTTATATCTGCAGTTTTGATTGATTTACGGCTTCAGCC
CATTTGGCATTTGTCTCCTCAGTTATGGCATTTCCATGTTAACATCTGCAAAACCTTTTAGCTAATTTCTTAAGACATGTGCGCTGGAAT
TGTATTTTACCTTGTACAACTTGTTGAATCTTATATGGGTAAAATTTTGCACTTACAAAAAACCATACACCTTAACAAATTATTGTTACC
TAGTAGTGTCATATATAGTGGCAGCATCATGCAAACACATACTGCAATGAGAGAACTGTAAACCATACCAAAAGTATAAAAATACAT
CAGTCAATGTCGATGAGCCAAAATTTCATCTGTCGCTAGTCAGAGACTCAGAGTTTAGCGCAGCAGAAACACTTTTACAGGTCTGCAA
GAGTGACAAAACTTATTGTTTTCCCATTCCTTCGGGACTATTTTGCAGCAAAAGATCCACCAGGGCTAGCCTTTAATCTTCCTCGTGTG
CCGCGGTGAAAATGGCAGAGGTAGTTAGATAGGGACACCATAATTCACAATCTTCGTGAGGGCAGAGTTAAGCACACGCTTCTCCTT
CTTGACCTGGGAGGCTAGACCAGAAATCTGCAAGGAAAAGAAAAGACAGTAGTGACACGAATTTATGTTAGAGGATATTAGGTCAG
GGCCATCAATGGTTTGAGAAGATGGGTGCAATCTACTGTGGCCGGCTGAAGCACCTAGTTTGGAAAGTACAGGTTGCTACTAGTGG
GCTGTGGGCACATTTAGTAGAAGGCTACATCCTGATTTTATTTGCTGTACCTAGTTGAAGAGGTGAATAGTAGTACTGTTGCAGATAC
TAACCTCTGATCGAAATTCAGATGCATGTGCTCTCGGAAGCTGATCAAGAATTCCTTTGAGACCTAAGAGGGAGCATTTTATTATATG
TAAGATTGAAGACAAGAAATTTCTACACTGAAATTCAGAGTCATAAGATAAAGCAGTGATCTCTCAAGGAATTGCTACACACCTACTG
CTTGGTTTTCAATATCACTAATCTGCTTCAGCTCACTTTGAATGGCGCGCCCTTCTTCCCTGAGAAAGTATACATTCACCCCTATGTTAT
ATTAAGCCCCAAAGTTAACAGGTCGAAACAATGAAAAACATGCCTGAGTGTATTCCATCCTTTCTGGAATCTTTTTTCTGCTGTTGCTG
CTCTATCCTTGATTCCATATGCAATAATTTTAACCATGAGTAACATTAGTTCCCAAGTTGAAATAAATGTCTCCGCTTTCTAAATATACA
TACAGAAAGTTACCATCAATTTTTGGCTTTCATTGGATACAAGATTGACAGTTTGCCGCATGTGATTCACTTCAGCTTGGATGCCGGAA
AGTAGGGTCTACAATATGGAAACTTGTGTTAGACAACAAGTCTATTTATCTTTGTCATTTCTAAGGAAAATCAACCATGCTTTTGAAAA
CTGTATGAAATAAATGAACTTCAGAAAACTGTTTCATTTTTGTTCTAAAAGGTACAAACAGAAGGAATCGTATCAGAACATGTAAATA
GTAAAACATGATGTACAATCAGTACAGAGTTAGCCACCTCGTAAGAGATGACAAGTTATAAGAAAAATGGTTGATGACTTGATGTAG
CTACCGGAACATGAAGCTAGCAGATAGCATAGTCAAGGAGTCTCGCTAAAGTATGAACTAATTAAACTGACACATAGTGCTTACCTCC
TTGCTGACAAATATTCGTCGTACACGTTGTATTAAGTAGCTCCTTGGCCCTGGAATGACTCATTGTCAGCCATACTAAATTTAGCAGTA
TATAAGAAAAACAATTTTTCAACATTCATGGAGCAAACTTCCACTGAACCAACAACCTTGTAATTCAGTACCAAGAAGTGCTAGGCAT
CTATGTCTACAGATTAGAAGTCGGATAATTTGAATTTCTTAGCAGCTTTAATGTTATTCGCCTAATCCGATATTCTAAGAACAATGCAA
TCAGTTAATTTGAACCTACAGGAAAAGACGATCTGTGCATGACAATTTAATTAGGATGCAGGTATTGGTTCGTGGAGGTCTCAAGAT
ATACCAATCAACTGTAAGAAATGAGCAATAAATTTTTTAAATTCCGATATAAGGTCCATCGTGCGGCACTAAGAACAAAAACTCATTA
TCCTCTACAAGAGCATTATTTCGCAAAGACTTAGACATGCAAGTAGCGTATTTCTTTGTGAGTAACAAACAAGAACCAACTAAATACT
AATACAAACAGCTTGAACTTATCAAACATAACGCGTCGCCACAAAGCAAATGAGTTCAGACGAAATGGCATTACTTTTGAGCAGCAC
AATTCCTGCAACTGTGGCAGAGCCTGCGGCAATGCCTGGATGCTCGATTGCCATGATGACACCCTCTGCAAAGACAGCAAATTGAGT
TTCAAATAATTGATTACAAAATATACAGCCACACCAATGCGGCACTGAAAGTCATCAATCAGACATACATATATGAACAATGCTACAT
ATCAATCCATAGATACCCCCCAAACAATTAGACAACAAACACCAAAAGGCGCACCTTTGATCTTGCCCAGCGCCATCTGCTCACGAGC
CAAGTACTCCTTCTTGACCCATTCCAGCTCTTCCTACAATTAAACAACCAAGTTGGAGCTTGAGCGAATTTCGGTGTGACCGAATCGG
AAGAGTGAGGGGAATGGATGGTTCCAGAAGAGGGGGGGCACCTTCGCGGCGTTGGTGAGGGTGGGGAAGGCGGCGAGGGCGGC
GGCCATGGCATCGTTGGCGGCATCAGAAACGGCGATGGCGGACGAGTGGGCGTGGGCGGTGGCCGTGTCGACGGTGGCCCTGGC
GGCAGCGACCATGTCGGCGCTGAATGCCTCACCCCGCCGCGTGAAATCCGCGGCGGCCGCCGCCGCGGAGGTCGCGGACGAGGCG
ACCATGGAGGAGATGGATCCGCCTTCGCTCTCCGGCTGCGGCTGCGGCGGCGGGGAGCCGCCGGGGGAGGAGGAGGAAGCGGAG
GGCGCGTCGGCCATGGCGGTAGGATCCAGAGAGAGGGAGAGGGGGCAGAGGAGAGAGACCTGTTTCCGGGAAATGAGATAAAAA
AAGAAGAAGCTCTCGCGTATTCCGTGATTCCGCTTTCATCAAGTGGAAACGTATTCGTTCAACGAAGCTACACGGTTGACAGACTTGA
CGTGTTCCATAAGCTATAGCGGAGAGTTTATGCCCGGCAACTAGTGGAGGAGATCGAGGCCGAAAATGGAAATGTCTTGGAACGAT

GCAAAAGGAAAAATGCCTTCGGATTCAATTTAAGTGTTCAAACGAGGAGCACATGTTTTGGGTTGTGGAACATGTGAAAAAACAGAC
GAAAAATCTCCGAAAACAAAAAGACCTCGCGCCAATGTTTTCATATCACACATTTTGATGGCGACCATATATAAGCTGATTTCACTTTT
TCGAGATATTCTACATGACGAGGAGGAGGAGATATTCTACCGGGAGATATTCGACGGTCCTGAAGAAAATTGATTGTGGTTCTGTCT
ACTTGTCTCAGCGCCTGCGTCCTCTGTCCTCTACTCCGGCGCATGCACAGTGAGTAGTGACAGTGCGCAATTAAGGAGCGATGGTACC
AGGCATGTGCACAGCCAGCTCACTGTGGAAGCCAATAATTCAGCAGGCTGTACTGGGAGCTAGTAAGCTACTGTAGAAGCCAATAAT
TCAGCAGCTTCTCTGTTCGTTCCTTCTCACAATCTCACCCGGCCGTTTCCTCCACCGCATCCGGACCTTAACCATCCTCTTCCCTTCTTCG
TTCCATTCTCCTCCTGCCGTCATGAGGAACAGAGATTCAGACTTGAGAGATCTGTTGTGATCCTGCCTGCGCGACAAAGATTCAGACT
TGAGGTACGTGCGATGTTACGTTAGAGCGTATGGATCTCAGAGATTTTCTCAGCCTTCAAGTTGTTTTTAGAGCGTATGAATCTCAGC
AGTTTCTTCAGCCTTTCAAGTTGTTTTTAGAGTGTATGAATCTCAGCAGTCTCTTTTTAAAACTGTATGGGTATGTCCTTGAGTGTATGT
ATGGACCTTCAAGTGCTTTCCTACATACTATTTCATTCGATCCAAGAGGCTATGGCTATCTAAATCCATCAGGAGAGTATTAAATTTAC
AGTACCTCTTCCTCTGTCTACCCAAACCCCCACCACCCCTGCCTTCTTTCGCGCCCGGCGTCAGATCTCGCGCGTCTAATCGTGTAGTG
ATAATTGATTCAAGTTGCATGGCTGTTCCTGTTCACATGGTTAGACGAATGGCAATAAGCTCCAGTATGAAGAGTCCATGATGGATCG
ATTCACTGTAAAAGAAAATGCTTGATGCATGGATCGATTTACTATCTTCCATTCCATGGGCAAGGCAATCGCAGGCTGCAGGCCTTGC
TCTCTTTCCAATTCCATCCAGATCTGCCATGGCACAGCACACGCCAACTGCCGAGGAAGGCTGCGGCAGTGATGAGAAAGAAAAAAA
AAATCTGATTTTCTTTTAAAAAATTTACCAACGTGAAAAGTTCATAATACCCTTTTCAAATTGGGAGGGGGTTTATACAGAAGCAGACC
TCAAATCTTAATTTGGTCTCTCGGTCTCTTCTTGCCTTTGTAGCACAAATTTCTAAAACCAGCCATGAATGACAACACATGTATAGTAG
GCAAGGTGTCATCTAAAATCACAAAACCATTGGGATTTCGTATTCAAAACACTTCCGTACAGCTAGAGCATCTCCACTCGCGTCCCCCC
ATTCGGCGTCTGGCGCTGCCGTTTTGTAGGCCGGATGGGGGGCACAAATATTTTAATTTGGGGATCCGCAGCCCTGCCCCCAATACC
AAATCTAAAATAAATAAATCCAAAAAGGGATAAATAAATTTAAAAAGGGATAAATTTAACTAGTTGGGGCGAAATTTATACAAAATT
CTAAAAAAACAACTAATCTACTCGTTGTCGTCCCACTCCTCCCCATCCCCGTCCTTGTCGAGCTCGCTGGTGAGGCCAAGGCAGCGCG
TGCGTTCGGCTTACTCCTTCTTGTGCTCCCTCGCCGACCAGGCGGAAATGGTCAAAGTTTGACATAAAATTCCGCGTAGGCCTTACAA
ACCTGGACAAGGGAGTATCTTGTTACTACGAGTCTAGGACTAACACTATTGTGCAAAAAATATTGTTGTCAAAGCTGCTTGCTGAACT
TGGAGTCTAGTTAAAATACTCTTTATCATTTCTAAATGAAGAGAAGAGAGTATTACATAAAAATTCCAAATTTAAGAAAAACATATGG
GGTAATCCAAGTATATCTACCCCGGCCTATGTTGATTTTGGTTGTATCATCTCATTTAAACTTGCTAGGGATACATGCATGCATAGAAG
TATGTCGTTTTATAATATGAGATAGATCGAAAGCTCAAAGCTTTGGTCTATTTAACACCCACGTTGCAGGTACCTTACAAGCATAACTA
GGATCCAAGATACACAATTGTCCAGCAAGAGATCC

```
Alignment of Sequence_1:  [Untitled Sequence #1] with  Sequence_2: [Sequence Window #2]

Similarity : 7107/7958 (89.31 %)

Seq_1  1      TGCAACACCCCCAGCCATGAGTTCTGTCAATAGCTTCAGTCACCACCCTCTTCAGAAGAG  60

Seq_2  1      ------------------------------------------------------------  0


Seq_1  61     ATGCCGCGTCAACAAGCTGTCGTCGAGCCTTAGGCTTCTGCAAGCCCGCCCAGTAGTCCA  120

Seq_2  1      ------------------------------------------------------------  0


Seq_1  121    GCCAATGACAGATAAAGATAACCAGATTAGAAGGATCACTAGGAAATATACATTGAAAAC  180

Seq_2  1      ------------------------------------------------------------  0


Seq_1  181    AAGCATTATTACGCGCCTTCCAGATAGACCAACAAAGTGCGGCTGTTCCTATTGCCGCTG  240

Seq_2  1      ------------------------------------------------------------  0


Seq_1  241    CAGGGCGAATAGAAACCTAAAATTTGCTGGCTTTGCAGTCTTTCTCCATGGGAAAGAAGC  300

Seq_2  1      ------------------------------------------------------------  0


Seq_1  301    AGGTTGGTAAACTCTGCTTCGGATTCAGCCAATGCTTGATGCAGTTGAATGAATTCATGT  360

Seq_2  1      ------------------------------------------------------------  0
```

```
Seq_1  361   TGTTACCTTTGGGGTTGTGAAACCCAAGGAGTTTCCTTTCGCCCAATGCAGACGGTGGCG  420

Seq_2  1     ------------------------------------------------------------  0


Seq_1  421   GCATCCTATCGTCGCATTCCCACGGCCGGTGAGAGCTGCTGAAGTTCCCAAAATTCCTTA  480

Seq_2  1     ------------------------------------------------------------  0


Seq_1  481   AAGACAAGATATCGATTTATATGATTGAAAGAACTCCTAAAATTCCCATGAAACAAGTTG  540

Seq_2  1     ------------------------------------------------------------  0


Seq_1  541   CAAGAATTCTGGAACTCCCATGAACTTCTACAGTTTGCGAAAGAACTTCCAGATTTTTTT  600

Seq_2  1     ------------------------------------------------------------  0


Seq_1  601   AGAGAACTTTGAAATTTTGTGAGAGAGCTTTCGAAGTGGGGAAACTTTCAACTAAATTTA  660

Seq_2  1     ------------------------------------------------------------  0


Seq_1  661   CACACTTCCAACAAATTCCCATGATGCAAAAAAGATTTCCAGGTGATTAATTGGCAACTT  720

Seq_2  1     ------------------------------------------------------------  0


Seq_1  721   CTTATAATCTTCCAAAAAAAGGTTTAACGAAAACTTATTCCTCGCAAGACATAAGGAGAA  780
                      |||||||||||||||||||||||||||||||||||||||||||||||||||
Seq_2  1     ---------TTCCAAAAAAAGGTTTAACGAAAACTTATTCCTCGCAAGACATAAGGAGAA  51


Seq_1  781   AATTTCCC----------------------------------CATTTACTCTAGTCTGCT  806
             ||||||||                                  |||||||||||||||
Seq_2  52    AATTTCCCTATTTACTCTAGTCTGCTTTTATTTTGACAGCCCCATTTACTCTAGTCT---  108


Seq_1  807   TTTATTTTGACAGCCCCATTTACTCTAGTCTACTTCACTGTACAGCTATTATACGGCAGC  866
                                            |||||||||||||||||||||||||||||
Seq_2  109   -------------------------------ACTTCACTGTACAGCTATTATACGGCAGC  137


Seq_1  867   AAGGCAGATAACACCAAAACAAATCTGCCCC-TTTCCCTCGCCACCGGCGCCGCCGAGGC  925
             ||||||||||||||||||||||||||||||| ||||||||||  ||||||||||||||||
Seq_2  138   AAGGCAGATAACACCAAAACAAATCTGCCCCCTTTCCCTCGCTGCCGGCGCCGCCGAGGC  197


Seq_1  926   CTCAATTTCCCCAAGCCGCGCCGGGTTCACCATGGAGGAATACCTCGTCGACGCCTGCGG  985
             |||||||||||||||||||||| |||||||||||||||||||||||||||||||||||||
Seq_2  198   CTCAATTTCCCCAAGCCGCGCCAGGTTCACCATGGAGGAATACCTCGTCGACGCCTGCGG  257


Seq_1  986   CCTGACCCGAGCCCAGGCGCTCAAGGCCTCCGCCAAGCTATCACACCTCAAGCCCCCCTC  1045
             ||||||||||||||||||||||||||||||||||||||||||||||||||||||||||||
Seq_2  258   CCTGACCCGAGCCCAGGCGCTCAAGGCCTCCGCCAAGCTATCACACCTCAAGCCCCCCTC  317


Seq_1  1046  CAACCCCGACGCCGTCGTCGCCTTCCTCTCCGGCGGCCTCGGCCTCT-------------  1092
             |||||||||||||||||||||||||||||||||||||||||||||||
Seq_2  318   CAACCCCGACGCCGTCGTCGCCTTCCTCTCCGGCGGCCTCGGCCTCTACATCGCCGATGT  377


Seq_1  1093  ------------------------------------------------------------  1092

Seq_2  378   TGGAGAATGCAATGCAAGCTTCTAGAGTGTTCTCAATATTACAAGAAAGAAGACATTACT  437
```

```
Seq_1  1093  ------------------------------------------------------------  1092

Seq_2  438   ATGGAATACTCTAGAATATAAAATAAATAAAAGAGGGAACCTTCTAGTGTTTAGATATGC  497


Seq_1  1093  ------------------------------------------------------------  1092

Seq_2  498   ATGAAGAATTATGTAGAAGCTTTGGAATACTCTAGAATAGGAGTTTAAAATAAATAAATA  557


Seq_1  1093  ------------------------------------------------------------  1092

Seq_2  558   GGAAACCTTCTAATACCTAGATATTTATGTAGAAGTGTGTGGAAGGCTTTAGATATTTTG  617


Seq_1  1093  ------------------------------------------CCATC-------------  1097
                                                       ||||
Seq_2  618   TATCAATGGCTAAGATTTTGACACATGTCAAAGATCCAATGGCCATGTAGTCTTATAAAT  677


Seq_1  1098  ------------------------------------------------------------  1097

Seq_2  678   AGAGGCCCTTGCCTCACAACTTGTGTAGTAGAGAATAAGAAAGTGAAGAAGGTGGAGAAC  737


Seq_1  1098  ------------------------------------------------------------  1097

Seq_2  738   AAGGTGTGAGCAAGTGTAAGGTGTGTATGCTCTCTCTTGTACTAATATTCCTAAAGCAAT  797


Seq_1  1098  ------------------------------------------------------------  1097

Seq_2  798   ATAGTACTACTTTGTTCATATAAGTCTCCCGGTTAGGCCTTGTTATTTTTAAGTACTTAG  857


Seq_1  1098  ------------------------------------------------------------  1097

Seq_2  858   TGCATATAAGTTGTGATTTAACGTGAGCGGATTCGTCCGGAATCACAACGGTCTTGTTTC  917


Seq_1  1098  ------------------------------------------------------------  1097

Seq_2  918   AACGTGAGTGGCATAGCCGCCCGGATTCAAGACTTGCTTTAACGTGAGTGGTTCTGCCGC  977


Seq_1  1098  ------------------------------------------------------------  1097

Seq_2  978   CCGGAAGCTTGCACTAAGTAAGTAGAAATAAAAAGGGACTAATCAACTACACCAAAGTAG  1037


Seq_1  1098  -----------------------------------------------------------G  1098
                                                                        |
Seq_2  1038  TTGGTATAGGTGAATAGGGCCTCTCGCAGTGTGTTAGGTCCACCTCGAATTCTCTGACAG  1097


Seq_1  1099  CCGACATCGCCGCCGTCGTCGCCAAGGACCCCAGGTTCCTCTGCGCCAGCGTGAAGAAAA  1158
             ||||||||||||||||||||||||||||||||||||||||||||||||||||||||||||
Seq_2  1098  CCGACATCGCCGCCGTCGTCGCCAAGGACCCCAGGTTCCTCTGCGCCAGCGTGAAGAAAA  1157


Seq_1  1159  CCCTGGCGCCCAACGTCGCCGCGCTCACCGCCGGCGCCGGCGGCCTGTCGCGTCCCGAGG  1218
             ||||||||||||||||||||||||||||||||||||||||||||||||||||||||||||
Seq_2  1158  CCCTGGCGCCCAACGTCGCCGCGCTCACCGCCGGCGCCGGCGGCCTGTCGCGTCCCGAGG  1217


Seq_1  1219  TCGCCAGCCTCGTCGTCCTCGCCCCCAGCCACTTCCGCCGCTGGTCCATCGTCTCCAAGC  1278
             ||||||||||||||||||||||||||||||||||||||||| ||||||||||||||||||
Seq_2  1218  TCGCCAGCCTCGTCGTCCTCGCCCCCAGCCACTTCCGCCGCCGGTCCATCGTCTCCAAGC  1277
```

```
Seq_1  1279  TGCGCTACTACCTGCGCCTCCTGGGCTCCGCCGTGGACCTCCTCCGGACTCTCAGGAAGA  1338
             ||||||||||||||||||||||||||||||||| ||||||||||||||||||||||||||
Seq_2  1278  TGCGCTACTACCTGCGCCTCCTGGGCTCCGCCGGGGACCTCCTCCGGACTCTCAGGAAGA  1337

Seq_1  1339  ACTCCCGCCTCCTCTCGGCCGACCTCGACGCCGTCGTCAGGCCCAACGCCGCGTTCCTGC  1398
             ||||||||||||||||||||||||||||||||||||||||||||||||||||||||||||
Seq_2  1338  ACTCCCGCCTCCTCTCGGCCGACCTCGACGCCGTCGTCAGGCCCAACGCCGCGTTCCTGC  1397

Seq_1  1399  GGGAGCAGTGCGGGCTAGGTGCTCGCGACATTGCCAAGCTGTGCAACCCCCTGCCGCTGC  1458
             ||||||||||||||||||||||||||||||||||||||||||||||||||||||||||||
Seq_2  1398  GGGAGCAGTGCGGGCTAGGTGCTCGCGACATTGCCAAGCTGTGCAACCCCCTGCCGCTGC  1457

Seq_1  1459  TGCTCTCGGCCGACCCGGAGCGCGTCCGGGCGATGGCGGCTTGCGCCGAGGGCGTCGTGG  1518
             ||||||||||||||||||||||||||||||||||||||||||||||||||||||||||||
Seq_2  1458  TGCTCTCGGCCGACCCGGAGCGCGTCCGGGCGATGGCGGCTTGCGCCGAGGGCGTCGTGG  1517

Seq_1  1519  GCGTGCCCCGCGGCTCCGGGATGTTCAGGCCGTCGCGTTCCTCAGCGAGAAGAAGACCGC  1578
             ||||||||||||||||||||||||||||||||||||||||||||||||||||||| ||||
Seq_2  1518  GCGTGCCCCGCGGCTCCGGGATGTTCAGGCCGTCGCGTTCCTCAGCGAGAAGAAGGCCGC  1577

Seq_1  1579  CGCCAAAGTCGAGAACTTGAAGGTGACGTTCGGGTGGTCGGACGACGAGGTGGCTGTTGC  1638
             ||||||||||||| ||||||||||||||||||||||||||||||||||||||||||||||
Seq_2  1578  CGCCAAAGTCGAGTACTTGAAGGTGACGTTCGGGTGGTCGGACGACGAGGTGGCTGTTGC  1637

Seq_1  1639  CGTGTCCAAGGCTCCGATGGTGCTGACGAATTCCAAGGACATGCTGCGGCGCAGGGCAGA  1698
             ||||||||||||||||||||||||||||||||||||||||||||||||||||||||||||
Seq_2  1638  CGTGTCCAAGGCTCCGATGGTGCTGACGAATTCCAAGGACATGCTGCGGCGCAGGGCAGA  1697

Seq_1  1699  GTTCCTGATCTCCGAGGTGGGGTTGGAACCGGCGTACATTGCTCGCCGGCCCGTCATGCT  1758
             ||||||||||||||||||||| ||||||||||||||| ||||||||||||||||||||||
Seq_2  1698  GTTCCTGATCTCCGAGGTGGG-TTGGAACCGGCGTACGTTGCTCGCCGGCCCGTCATGCT  1756

Seq_1  1759  CAGTTATAGCTTGGAGGGCCGGCTCAAACCCCGGTACTGCGTCATAAAGTTTCTCAAGGA  1818
             ||||||||||||||||||||||||||||||||||||||||||||||||||||||||||||
Seq_2  1757  CAGTTATAGCTTGGAGGGCCGGCTCAAACCCCGGTACTGCGTCATAAAGTTTCTCAAGGA  1816

Seq_1  1819  ACATGGGTTGCTGGATCATGACAGGGACTACTACAAAACAGTCATGGTCAGCGAGAAGGT  1878
             ||||||||||||||||||||||||||||||||||||||||||||||||||||||||||||
Seq_2  1817  ACATGGGTTGCTGGATCATGACAGGGACTACTACAAAACAGTCATGGTCAGCGAGAAGGT  1876

Seq_1  1879  GTTCATGGAGAAGTTCATATGCCCTCACAGCGAAGCTGCGCCGCACCTTGCCGAAGCCTA  1938
             |||||||||||||||||||||||||| |||||||||||||||||||||||| ||||||||
Seq_2  1877  GTTCATGGAGAAGTTCATATGCCCTCGCAGCGAAGCTGCGCCGCACCTTGCTGAAGCCTA  1936

Seq_1  1939  TGATGCCGCTTGCAGAGCGGAAGTGCCTGCTAATTTTTGGCTTACATGAACCAAGAATGG  1998
             ||||||||||||||||||||||||||||||||||||||||||||||||||||||||||||
Seq_2  1937  TGATGCCGCTTGCAGAGCGGAAGTGCCTGCTAATTTTTGGCTTACATGAACCAAGAATGG  1996

Seq_1  1999  ACCATTGGAATTGGTAACTACGTATGGCATGACAAAGTTATCACTCTGTTTAGTATGTTG  2058
             ||||||||||||||||||||||||||||||||||||||||||||||||||||||||||||
Seq_2  1997  ACCATTGGAATTGGTAACTACGTATGGCATGACAAAGTTATCACTCTGTTTAGTATGTTG  2056

Seq_1  2059  CATGTTGATTTGGTATTTGCTAGCCCTGGATGAAACTGATAAATTGATTAGTGAGTCCAG  2118
             ||||||||||||||||||||||||||||||||||||||||||||||||||||||||||||
Seq_2  2057  CATGTTGATTTGGTATTTGCTAGCCCTGGATGAAACTGATAAATTGATTAGTGAGTCCAG  2116
```

```
Seq_1  2119  GTATGTCGATTCATCATTCAGTAGAGAAGAAAGCAGAGAATGATATGTAATTCCATCCTC  2178
             ||||||||||||||||||||||||||||||||||||||||||||||||||||||||||||
Seq_2  2117  GTATGTCGATTCATCATTCAGTAGAGAAGAAAGCAGAGAATGATATGTAATTCCATCCTC  2176

Seq_1  2179  ATGCCATATTGATAGACATGTACTTGACATGTTGCAACTTAAAGAGTGTGAGGTCAGGCC  2238
             ||||||||||||||||||||| ||||| ||||||||||||||||||||||||||||||||
Seq_2  2177  ATGCCATATTGATAGACATGTGCTTGAGATGTTGCAACTTAAAGAGTGTGAGGTCAGGCC  2236

Seq_1  2239  ATTCTGCAAATGTGTTGAAGTATCAGTGAGTTGTATACTATATCTGCACACTTGATGTCC  2298
             ||||||||||||||||||||||||||||||||||||||||||||||||||||||||||||
Seq_2  2237  ATTCTGCAAATGTGTTGAAGTATCAGTGAGTTGTATACTATATCTGCACACTTGATGTCC  2296

Seq_1  2299  TTATGTTCTCCAATGGCCATTGCAGTTTTCAGGTCAATCCTTAATCATTTTAGTCTGCAT  2358
             ||||||||||||||||||||||||||||||||||||||||||||||||||||||||||||
Seq_2  2297  TTATGTTCTCCAATGGCCATTGCAGTTTTCAGGTCAATCCTTAATCATTTTAGTCTGCAT  2356

Seq_1  2359  AACCTGCCAGGATTCAGAGAAGGAAAGCTTGAGAGAGTCGTGTTTTTCCTACGGAGTATG  2418
             ||||||||||||||||||||||||||||||||||||||||||||||||||||||||||||
Seq_2  2357  AACCTGCCAGGATTCAGAGAAGGAAAGCTTGAGAGAGTCGTGTTTTTCCTACGGAGTATG  2416

Seq_1  2419  TTATATCTGCAGTTCTGATTGATTTACGGCTTCAGCCCATTTGGCATTTGTCTCCTCAGT  2478
             |||||||||||||| |||||||||||||||||||||||||||||||||||||||||||||
Seq_2  2417  TTATATCTGCAGTTTTGATTGATTTACGGCTTCAGCCCATTTGGCATTTGTCTCCTCAGT  2476

Seq_1  2479  TATGGCATTTCCATGTTAACATCTG-AAAACCTTTTAGCTAATTTCTTAAGACATGTGCG  2537
             ||||||||||||||||||||||||| ||||||||||||||||||||||||||||||||||
Seq_2  2477  TATGGCATTTCCATGTTAACATCTGCAAAACCTTTTAGCTAATTTCTTAAGACATGTGCG  2536

Seq_1  2538  CTGGAATTGTATTTTACCTTGTACAACTTGTTGAATCTTATATGGGTAAAATTTTGCACT  2597
             ||||||||||||||||||||||||||||||||||||||||||||||||||||||||||||
Seq_2  2537  CTGGAATTGTATTTTACCTTGTACAACTTGTTGAATCTTATATGGGTAAAATTTTGCACT  2596

Seq_1  2598  TACAAAAAACCATACACGTTAACAAATTATTGTTACCTAGTAGTGTCATATATAGTGGCA  2657
             ||||||||||||||||| ||||||||||||||||||||||||||||||||||||||||||
Seq_2  2597  TACAAAAAACCATACACCTTAACAAATTATTGTTACCTAGTAGTGTCATATATAGTGGCA  2656

Seq_1  2658  GCATCATGCAAACACATACTGCAATGAGAGAACTGTAAACCATACCAAAAGTATAAAAAT  2717
             ||||||||||||||||||||||||||||||||||||||||||||||||||||||||||||
Seq_2  2657  GCATCATGCAAACACATACTGCAATGAGAGAACTGTAAACCATACCAAAAGTATAAAAAT  2716

Seq_1  2718  ACATCAGTCAATGTCGATGAGCCAAAATTTCATCTGTCGCTAGTCAGAGACTCAGAGTTT  2777
             ||||||||||||||||||||||||||||||||||||||||||||||||||||||||||||
Seq_2  2717  ACATCAGTCAATGTCGATGAGCCAAAATTTCATCTGTCGCTAGTCAGAGACTCAGAGTTT  2776

Seq_1  2778  AGCGCAGCAGAAACA-TTTTACAGGTCTGCAAGAGTGACAAAACTTATTGTTTTCCCATT  2836
             ||||||||||||||| ||||||||||||||||||||||||||||||||||||||||||||
Seq_2  2777  AGCGCAGCAGAAACACTTTTACAGGTCTGCAAGAGTGACAAAACTTATTGTTTTCCCATT  2836

Seq_1  2837  CCTTCGGGACTATTTTGCAGCAAAAGATCCACCAGGGCTAGCCTTTAATCTTCCTCGTGT  2896
             ||||||||||||||||||||||||||||||||||||||||||||||||||||||||||||
Seq_2  2837  CCTTCGGGACTATTTTGCAGCAAAAGATCCACCAGGGCTAGCCTTTAATCTTCCTCGTGT  2896

Seq_1  2897  G-----GTGAAAATGGCAGAGGTAGTTAGATAGGGACACCATAATTCACAATCTTCGTGA  2951
             |     ||||||||||||||||||||||||||||||||||||||||||||||||||||||
Seq_2  2897  GCCGCGGTGAAAATGGCAGAGGTAGTTAGATAGGGACACCATAATTCACAATCTTCGTGA  2956
```

```
Seq_1  2952  GGGCAGAGTTAAGCACACGCTTCTCCTTCTTGACCTGGGAGGCTAGACCAGAAATCTGCA  3011
             ||||||||||||||||||||||||||||||||||||||||||||||||||||||||||||
Seq_2  2957  GGGCAGAGTTAAGCACACGCTTCTCCTTCTTGACCTGGGAGGCTAGACCAGAAATCTGCA  3016

Seq_1  3012  AGGAAAAGAAAAGACAGTAGTGACACGAATTTATGTTAGAGGATATTAGGTCAGGGCCAT  3071
             ||||||||||||||||||||||||||||||||||||||||||||||||||||||||||||
Seq_2  3017  AGGAAAAGAAAAGACAGTAGTGACACGAATTTATGTTAGAGGATATTAGGTCAGGGCCAT  3076

Seq_1  3072  CAATGGTTTGAGAAGATGGGTGCAATCTACTGTGGCCGGCTGAAGCACCTAGTTTGGAAA  3131
             ||||||||||||||||||||||||||||||||||||||||||||||||||||||||||||
Seq_2  3077  CAATGGTTTGAGAAGATGGGTGCAATCTACTGTGGCCGGCTGAAGCACCTAGTTTGGAAA  3136

Seq_1  3132  GTACAGGTTGCTACTAGTGGGCTGTGGGCACATTTAGTAGAAGGCTACATCCTGATTTTA  3191
             ||||||||||||||||||||||||||||||||||||||||||||||||||||||||||||
Seq_2  3137  GTACAGGTTGCTACTAGTGGGCTGTGGGCACATTTAGTAGAAGGCTACATCCTGATTTTA  3196

Seq_1  3192  TTTGCTGTACCTAGTTGAAGAGGTGGATAGTAGTACTGTTGCAGATACTAACCTCTGATC  3251
             ||||||||||||||||||||||||| ||||||||||||||||||||||||||||||||||
Seq_2  3197  TTTGCTGTACCTAGTTGAAGAGGTGAATAGTAGTACTGTTGCAGATACTAACCTCTGATC  3256

Seq_1  3252  GAAATTCAGATGCATGTGCTCTCGGAAGCTGATCAAGAATTCCTTTGAGACCTAAGAGGG  3311
             ||||||||||||||||||||||||||||||||||||||||||||||||||||||||||||
Seq_2  3257  GAAATTCAGATGCATGTGCTCTCGGAAGCTGATCAAGAATTCCTTTGAGACCTAAGAGGG  3316

Seq_1  3312  AGCATTTTATTATATGTAAGATTGAAGACAAGAAATTTCTACACTGAAATTCAGAGTCAT  3371
             ||||||||||||||||||||||||||||||||||||||||||||||||||||||||||||
Seq_2  3317  AGCATTTTATTATATGTAAGATTGAAGACAAGAAATTTCTACACTGAAATTCAGAGTCAT  3376

Seq_1  3372  AAGATAAAGCAGTGATCTCTCAAGGAATTGCTACACACCTACTACTTGGTTTTCAATATC  3431
             ||||||||||||||||||||||||||||||||||||||||||| ||||||||||||||||
Seq_2  3377  AAGATAAAGCAGTGATCTCTCAAGGAATTGCTACACACCTACTGCTTGGTTTTCAATATC  3436

Seq_1  3432  ACTAATCTGCTTCAGCTCACTTTGAATGGCGCGCCCTTCTTCCCTGAGAAAGTATACATT  3491
             ||||||||||||||||||||||||||||||||||||||||||||||||||||||||||||
Seq_2  3437  ACTAATCTGCTTCAGCTCACTTTGAATGGCGCGCCCTTCTTCCCTGAGAAAGTATACATT  3496

Seq_1  3492  CACCCCTATGTTATATTAAGCCCCAAAGTTAACAGGTCGAAACAATGAAAAACATGCCTG  3551
             ||||||||||||||||||||||||||||||||||||||||||||||||||||||||||||
Seq_2  3497  CACCCCTATGTTATATTAAGCCCCAAAGTTAACAGGTCGAAACAATGAAAAACATGCCTG  3556

Seq_1  3552  AGTGTATTCCATCCTTTCTGGAATCTTTTTTCTGCCGTTGCTGCTCTATCCTTGATTCCA  3611
             ||||||||||||||||||||||||||||||||||| ||||||||||||||||||||||||
Seq_2  3557  AGTGTATTCCATCCTTTCTGGAATCTTTTTTCTGCTGTTGCTGCTCTATCCTTGATTCCA  3616

Seq_1  3612  TATGCAATAATTTTAACCATGAGTAACATTAGTTCCCAAGTTGAAATAAATGTCTCCGCT  3671
             ||||||||||||||||||||||||||||||||||||||||||||||||||||||||||||
Seq_2  3617  TATGCAATAATTTTAACCATGAGTAACATTAGTTCCCAAGTTGAAATAAATGTCTCCGCT  3676

Seq_1  3672  TTCTAAATATACATACAGAAAGTTACCATCAATTTTTGGCTTTCATTGGATACAAGATTG  3731
             ||||||||||||||||||||||||||||||||||||||||||||||||||||||||||||
Seq_2  3677  TTCTAAATATACATACAGAAAGTTACCATCAATTTTTGGCTTTCATTGGATACAAGATTG  3736

Seq_1  3732  ACAGTTTGCCGCATGTGATTCACTTCAGCTTGGATGCCGGAAAGTAGGGTCTACAATATG  3791
             ||||||||||||||||||||||||||||||||||||||||||||||||||||||||||||
Seq_2  3737  ACAGTTTGCCGCATGTGATTCACTTCAGCTTGGATGCCGGAAAGTAGGGTCTACAATATG  3796
```

```
Seq_1  3792  GAAACTTGTGTTAGACAACAAGTCTATTTATCTTTGTCATTTCTAAGGAAAATCAACCAT  3851
             ||||||||||||||||||||||||||||||||||||||||||||||||||||||||||||
Seq_2  3797  GAAACTTGTGTTAGACAACAAGTCTATTTATCTTTGTCATTTCTAAGGAAAATCAACCAT  3856


Seq_1  3852  GCTTTTGAAAACTGTATGTAAAATAAATGAACTTCAGAAAACTGTTTCATTTTTGTTCTA  3911
             ||||||||||||||||||  ||||||||||||||||||||||||||||||||||||||||
Seq_2  3857  GCTTTTGAAAACTGTATG--AAATAAATGAACTTCAGAAAACTGTTTCATTTTTGTTCTA  3914


Seq_1  3912  AAAGGTACAAACAGAAGGAATCGTATCAGAACATGTAAATAGTAAAACATGATGTACAAT  3971
             ||||||||||||||||||||||||||||||||||||||||||||||||||||||||||||
Seq_2  3915  AAAGGTACAAACAGAAGGAATCGTATCAGAACATGTAAATAGTAAAACATGATGTACAAT  3974


Seq_1  3972  CAGTACAGAGTTAGCCACCTCGTAAGAGATGACAAGTTATAAGAAAAATGGTTGATGACT  4031
             ||||||||||||||||||||||||||||||||||||||||||||||||||||||||||||
Seq_2  3975  CAGTACAGAGTTAGCCACCTCGTAAGAGATGACAAGTTATAAGAAAAATGGTTGATGACT  4034


Seq_1  4032  TGATGTAGCTACTGGAACATGAAGCTAGCAGATAGCATAGTCAAGGAGTCTCGCTAAAGT  4091
             |||||||||||| |||||||||||||||||||||||||||||||||||||||||||||||
Seq_2  4035  TGATGTAGCTACCGGAACATGAAGCTAGCAGATAGCATAGTCAAGGAGTCTCGCTAAAGT  4094


Seq_1  4092  ATGAACTAATTAAACTGACACATAGTGCTTACCTCCTTGCTGACAAATATTCGTCGTACA  4151
             ||||||||||||||||||||||||||||||||||||||||||||||||||||||||||||
Seq_2  4095  ATGAACTAATTAAACTGACACATAGTGCTTACCTCCTTGCTGACAAATATTCGTCGTACA  4154


Seq_1  4152  CGTTGTATTAAGTAGCTCCTTGGCCCTGGAATGACTCATTGTCAGCCATACTAAATTTAG  4211
             ||||||||||||||||||||||||||||||||||||||||||||||||||||||||||||
Seq_2  4155  CGTTGTATTAAGTAGCTCCTTGGCCCTGGAATGACTCATTGTCAGCCATACTAAATTTAG  4214


Seq_1  4212  CAGTATATAAGAAAAACAATTTTTCAACATTCATGGAGCAAACTTCCACTGAACCAACAA  4271
             ||||||||||||||||||||||||||||||||||||||||||||||||||||||||||||
Seq_2  4215  CAGTATATAAGAAAAACAATTTTTCAACATTCATGGAGCAAACTTCCACTGAACCAACAA  4274


Seq_1  4272  CCTTGTAATTCAGTACCAAGAAGTGCTAGGCATCTATGTCTACAGATTAGAAGTCGGATA  4331
             ||||||||||||||||||||||||||||||||||||||||||||||||||||||||||||
Seq_2  4275  CCTTGTAATTCAGTACCAAGAAGTGCTAGGCATCTATGTCTACAGATTAGAAGTCGGATA  4334


Seq_1  4332  ATTTGAATTTCTTAGCAGCTTTAATGTTATTCGCCTAATCCGATATTCTAAGAACAATGC  4391
             ||||||||||||||||||||||||||||||||||||||||||||||||||||||||||||
Seq_2  4335  ATTTGAATTTCTTAGCAGCTTTAATGTTATTCGCCTAATCCGATATTCTAAGAACAATGC  4394


Seq_1  4392  AATCAGTTAATTTGAACCTACAGGAAAAGACGATCTGTGCATGACAATTTAATTAGGATG  4451
             ||||||||||||||||||||||||||||||||||||||||||||||||||||||||||||
Seq_2  4395  AATCAGTTAATTTGAACCTACAGGAAAAGACGATCTGTGCATGACAATTTAATTAGGATG  4454


Seq_1  4452  CAGGTAACTGGTTCGTGGAGGTCTCAAGATATACCAATCAACTGTAAGAAATGAGCAATA  4511
             ||||||  ||||||||||||||||||||||||||||||||||||||||||||||||||||
Seq_2  4455  CAGGTAT-TGGTTCGTGGAGGTCTCAAGATATACCAATCAACTGTAAGAAATGAGCAATA  4513


Seq_1  4512  AATTTTTTAAATTCCGATATAAGGTCCATCGTGCGGCACTAAGAACAAAAACTCATTATC  4571
             ||||||||||||||||||||||||||||||||||||||||||||||||||||||||||||
Seq_2  4514  AATTTTTTAAATTCCGATATAAGGTCCATCGTGCGGCACTAAGAACAAAAACTCATTATC  4573


Seq_1  4572  CTCTACAAGAGCATTATTTCGCAAAGACTTAGACATGCAAGTAGCGTATTTCTTTGTGAG  4631
             ||||||||||||||||||||||||||||||||||||||||||||||||||||||||||||
Seq_2  4574  CTCTACAAGAGCATTATTTCGCAAAGACTTAGACATGCAAGTAGCGTATTTCTTTGTGAG  4633
```

```
Seq_1  4632  TAACAAACAAGAACCAACTAAATACTAATACAAACAGCTTGAACTTATCAAACATAAGGC  4691
             ||||||||||||||||||||||||||||||||||||||||||||||||||||||||| ||
Seq_2  4634  TAACAAACAAGAACCAACTAAATACTAATACAAACAGCTTGAACTTATCAAACATAACGC  4693

Seq_1  4692  GTCGCCACAAAGCAAATGAGTTCAGACGAAATGGCATTACTTTTGAGCAGCACAATTCCT  4751
             ||||||||||||||||||||||||||||||||||||||||||||||||||||||||||||
Seq_2  4694  GTCGCCACAAAGCAAATGAGTTCAGACGAAATGGCATTACTTTTGAGCAGCACAATTCCT  4753

Seq_1  4752  GCAACTGTGGCAGAGCCTGCGGCAATGCCTGGATGCTCGATTGCCATGATGACACCCTCT  4811
             ||||||||||||||||||||||||||||||||||||||||||||||||||||||||||||
Seq_2  4754  GCAACTGTGGCAGAGCCTGCGGCAATGCCTGGATGCTCGATTGCCATGATGACACCCTCT  4813

Seq_1  4812  GCAAAGACAGCAAATTGAGTTTCAAATAATTGATTACAAAATATACAGCCACACCAATGT  4871
             ||||||||||||||||||||||||||||||||||||||||||||||||||||||||||| 
Seq_2  4814  GCAAAGACAGCAAATTGAGTTTCAAATAATTGATTACAAAATATACAGCCACACCAATGC  4873

Seq_1  4872  GGCACTGAAAGTCATCAATCAGACATACATATATGAACAATGCTACATATCAATCCATAG  4931
             ||||||||||||||||||||||||||||||||||||||||||||||||||||||||||||
Seq_2  4874  GGCACTGAAAGTCATCAATCAGACATACATATATGAACAATGCTACATATCAATCCATAG  4933

Seq_1  4932  ATACCCCCCAAACAATTAGACAACAAACACCAAAAGGCGCACCTTTGATCTTGCCCAGCG  4991
             ||||||||||||||||||||||||||||||||||||||||||||||||||||||||||||
Seq_2  4934  ATACCCCCCAAACAATTAGACAACAAACACCAAAAGGCGCACCTTTGATCTTGCCCAGCG  4993

Seq_1  4992  CCATCTGCTCACGAGCCAAGTACTCCTTCTTGACCCATTCCAGCTCTTCCTACAATTAAA  5051
             ||||||||||||||||||||||||||||||||||||||||||||||||||||||||||||
Seq_2  4994  CCATCTGCTCACGAGCCAAGTACTCCTTCTTGACCCATTCCAGCTCTTCCTACAATTAAA  5053

Seq_1  5052  CAACCAAGTTGGAGCTTGAGCGAATTTCGGTGTGACCGAATCGGAAGAGTGAGGGGAATG  5111
             ||||||||||||||||||||||||||||||||||||||||||||||||||||||||||||
Seq_2  5054  CAACCAAGTTGGAGCTTGAGCGAATTTCGGTGTGACCGAATCGGAAGAGTGAGGGGAATG  5113

Seq_1  5112  GATGGTTCCAGAAGAGGGGGGGCACCTTCGCGGCGTTGGTGAGGGTGGGGAAGGCGGCGA  5171
             ||||||||||||||||||||||||||||||||||||||||||||||||||||||||||||
Seq_2  5114  GATGGTTCCAGAAGAGGGGGGGCACCTTCGCGGCGTTGGTGAGGGTGGGGAAGGCGGCGA  5173

Seq_1  5172  GGGCGGCGGCCATGGCATCGTTGGCGGCATCAGAAGCGGCGATGGCGGACGAGTGCGCGT  5231
             ||||||||||||||||||||||||||||||||||| ||||||||||||||||||| ||||
Seq_2  5174  GGGCGGCGGCCATGGCATCGTTGGCGGCATCAGAAACGGCGATGGCGGACGAGTGGGCGT  5233

Seq_1  5232  GGGCGGTGGCCGTGTCGACGGTGGCCCTGGCGGCAGCGACCATGTCGGCGCTGAATGCCT  5291
             ||||||||||||||||||||||||||||||||||||||||||||||||||||||||||||
Seq_2  5234  GGGCGGTGGCCGTGTCGACGGTGGCCCTGGCGGCAGCGACCATGTCGGCGCTGAATGCCT  5293

Seq_1  5292  CACCCCGCCGCGTGAAATCCGCGGCGGCCGCCGCCGCGGAGGTCGCGGACGAGGCGACCA  5351
             ||||||||||||||||||||||||||||||||||||||||||||||||||||||||||||
Seq_2  5294  CACCCCGCCGCGTGAAATCCGCGGCGGCCGCCGCCGCGGAGGTCGCGGACGAGGCGACCA  5353

Seq_1  5352  TGGAGGAGATGGATCCGCCTTCGCTCTCCGGCTGCGGCTGCGGCGGCGGGGAGCCGCCGG  5411
             ||||||||||||||||||||||||||||||||||||||||||||||||||||||||||||
Seq_2  5354  TGGAGGAGATGGATCCGCCTTCGCTCTCCGGCTGCGGCTGCGGCGGCGGGGAGCCGCCGG  5413

Seq_1  5412  GGGAGGAGGAGGAAGCGGAGGGCGCGTCGGCCATGGCGGTAGGATCCAGAGAGAGGGAGA  5471
             ||||||||||||||||||||||||||||||||||||||||||||||||||||||||||||
Seq_2  5414  GGGAGGAGGAGGAAGCGGAGGGCGCGTCGGCCATGGCGGTAGGATCCAGAGAGAGGGAGA  5473
```

```
Seq_1  5472  GGGGGGCAGAGGAGAGAGACCTATTTCCGGGAAATGAGATAAAAAAAGAAGAAGCTTTCG  5531
             ||||| |||||||||||||||| ||||||||||||||||||||||||||||||||| |||
Seq_2  5474  GGGGG-CAGAGGAGAGAGACCTGTTTCCGGGAAATGAGATAAAAAAAGAAGAAGCTCTCG  5532


Seq_1  5532  CGTATTCCATGATTCCGCTTTCATCAAGTGAAAACGTTTTCGTTCAACGAGGCAACACGG  5591
             |||||||| ||||||||||||||||||||| |||||| |||||||||||| || ||||||
Seq_2  5533  CGTATTCCGTGATTCCGCTTTCATCAAGTGGAAACGTATTCGTTCAACGAAGCTACACGG  5592


Seq_1  5592  TTGACAGACTTGACGTGTTCCATAAGCTATAGCGGAGAGTTTATGCCCGGCAACTAGTGG  5651
             ||||||||||||||||||||||||||||||||||||||||||||||||||||||||||||
Seq_2  5593  TTGACAGACTTGACGTGTTCCATAAGCTATAGCGGAGAGTTTATGCCCGGCAACTAGTGG  5652


Seq_1  5652  AGGAGATCGAGGCCGAAAATGGAAATGTCTTGGAACGATGCAAAAGGAAAAATGCCTTCG  5711
             ||||||||||||||||||||||||||||||||||||||||||||||||||||||||||||
Seq_2  5653  AGGAGATCGAGGCCGAAAATGGAAATGTCTTGGAACGATGCAAAAGGAAAAATGCCTTCG  5712


Seq_1  5712  GATTCAATTTAAGTGTTCAAACGAGGAGCACATGTTTTGGGTTGTGGAACATGTGAAAAA  5771
             ||||||||||||||||||||||||||||||||||||||||||||||||||||||||||||
Seq_2  5713  GATTCAATTTAAGTGTTCAAACGAGGAGCACATGTTTTGGGTTGTGGAACATGTGAAAAA  5772


Seq_1  5772  ACAGACGAAAAATCTCCGAAAACAAAAAGACCTCGCGCCAATGTTTTCATATCACACATT  5831
             ||||||||||||||||||||||||||||||||||||||||||||||||||||||||||||
Seq_2  5773  ACAGACGAAAAATCTCCGAAAACAAAAAGACCTCGCGCCAATGTTTTCATATCACACATT  5832


Seq_1  5832  TTGATGGCGACCATATATAAGCTGATTTCACTTTTTCGAGATATTCTACATGACGAGGAG  5891
             ||||||||||||||||||||||||||||||||||||||||||||||||||||||||||||
Seq_2  5833  TTGATGGCGACCATATATAAGCTGATTTCACTTTTTCGAGATATTCTACATGACGAGGAG  5892


Seq_1  5892  GAGGAGATATTCTACCGGGAGATATTCGACGGTCCTGAAGAAAATTGATTGTGGTTCTGT  5951
             ||||||||||||||||||||||||||||||||||||||||||||||||||||||||||||
Seq_2  5893  GAGGAGATATTCTACCGGGAGATATTCGACGGTCCTGAAGAAAATTGATTGTGGTTCTGT  5952


Seq_1  5952  CTACTTGTCTCAGCGCCTGCGTCCTCTGTCCTCTACTCCGGCGCATGCACAGTGAGTAGT  6011
             ||||||||||||||||||||||||||||||||||||||||||||||||||||||||||||
Seq_2  5953  CTACTTGTCTCAGCGCCTGCGTCCTCTGTCCTCTACTCCGGCGCATGCACAGTGAGTAGT  6012


Seq_1  6012  GACAGTGCGCAATTAAGGAGCGATGGTACCAGGCATGTGCACAGCCAGCTCACTGTGGAA  6071
             ||||||||||||||||||||||||||||||||||||||||||||||||||||||||||||
Seq_2  6013  GACAGTGCGCAATTAAGGAGCGATGGTACCAGGCATGTGCACAGCCAGCTCACTGTGGAA  6072


Seq_1  6072  GCCAATAATTCAGCAGGCTGTACTGGGAGCTAGTAAGCTACTGTAGAAGCCAATAATTCA  6131
             ||||||||||||||||||||||||||||||||||||||||||||||||||||||||||||
Seq_2  6073  GCCAATAATTCAGCAGGCTGTACTGGGAGCTAGTAAGCTACTGTAGAAGCCAATAATTCA  6132


Seq_1  6132  GCAGCTTCTCTGTTCGTTCCTTCTCACAATCTCACCCGGCCGTTTCCTCCACCGCATCCG  6191
             ||||||||||||||||||||||||||||||||||||||||||||||||||||||||||||
Seq_2  6133  GCAGCTTCTCTGTTCGTTCCTTCTCACAATCTCACCCGGCCGTTTCCTCCACCGCATCCG  6192


Seq_1  6192  GACCTTAACCATCCTCTTCCCTTCTTCGTTCCATTCTCCTCCTGCCGTCATGAGGAACAG  6251
             ||||||||||||||||||||||||||||||||||||||||||||||||||||||||||||
Seq_2  6193  GACCTTAACCATCCTCTTCCCTTCTTCGTTCCATTCTCCTCCTGCCGTCATGAGGAACAG  6252


Seq_1  6252  AGATTCAGACTTGAGAGATCTGTTGTGATCCTGCCTGCGCGACAAAGATTCAGACTTGAG  6311
             ||||||||||||||||||||||||||||||||||||||||||||||||||||||||||||
Seq_2  6253  AGATTCAGACTTGAGAGATCTGTTGTGATCCTGCCTGCGCGACAAAGATTCAGACTTGAG  6312
```

```
Seq_1  6312  GTACGTGCGATGTTACGTTAGAGCGTATGGATCTCAGAGATTTTCTCAGCCTTCAAGTTG  6371
             ||||||||||||||||||||||||||||||||||||||||||||||||||||||||||||
Seq_2  6313  GTACGTGCGATGTTACGTTAGAGCGTATGGATCTCAGAGATTTTCTCAGCCTTCAAGTTG  6372

Seq_1  6372  TTTTTAGAGCGTATGAATCTCAGCAGTTTCTTCAGCCTTTCAAGTTGTTTTTAGAGTGTA  6431
             ||||||||||||||||||||||||||||||||||||||||||||||||||||||||||||
Seq_2  6373  TTTTTAGAGCGTATGAATCTCAGCAGTTTCTTCAGCCTTTCAAGTTGTTTTTAGAGTGTA  6432

Seq_1  6432  TGAATCTCAGCAGTCTCTTTTTAAAACTGTATGGGTATGTCCTAGAGTGTATGTATGGAC  6491
             ||||||||||||||||||||||||||||||||||||||||||| ||||||||||||||||
Seq_2  6433  TGAATCTCAGCAGTCTCTTTTTAAAACTGTATGGGTATGTCCTTGAGTGTATGTATGGAC  6492

Seq_1  6492  CTTCAAGTGCTTTCCTACATACTATTTCATTCGATCCAAGAGGCTATGGCTATCTAAATC  6551
             ||||||||||||||||||||||||||||||||||||||||||||||||||||||||||||
Seq_2  6493  CTTCAAGTGCTTTCCTACATACTATTTCATTCGATCCAAGAGGCTATGGCTATCTAAATC  6552

Seq_1  6552  CATCAGGAGAGTATTAAATTTACAGTACCTCTTCCTCTGTCTACCCAAACCCCCACCACC  6611
             ||||||||||||||||||||||||||||||||||||||||||||||||||||||||||||
Seq_2  6553  CATCAGGAGAGTATTAAATTTACAGTACCTCTTCCTCTGTCTACCCAAACCCCCACCACC  6612

Seq_1  6612  CCTGCCTTCTTTCGCGCCCGGCGTCAGATCTCGCGCGTCTAATCGTGTAGTGATAATTGA  6671
             ||||||||||||||||||||||||||||||||||||||||||||||||||||||||||||
Seq_2  6613  CCTGCCTTCTTTCGCGCCCGGCGTCAGATCTCGCGCGTCTAATCGTGTAGTGATAATTGA  6672

Seq_1  6672  TTCAAGTTGCATGGCTGTTCCTGTTCACATGGT-AGACGGATGGCAATAAGCTCCAGTAT  6730
             ||||||||||||||||||||||||||||||||| ||||| ||||||||||||||||||||
Seq_2  6673  TTCAAGTTGCATGGCTGTTCCTGTTCACATGGTTAGACGAATGGCAATAAGCTCCAGTAT  6732

Seq_1  6731  GAAGAGTCCATGATGGATCGATTCACTGTAAAAGAAAATGCTTGATGCATGGATCGATTT  6790
             ||||||||||||||||||||||||||||||||||||||||||||||||||||||||||||
Seq_2  6733  GAAGAGTCCATGATGGATCGATTCACTGTAAAAGAAAATGCTTGATGCATGGATCGATTT  6792

Seq_1  6791  ACTATCTTCCATTCCATGGGCAAGGCAATCGCAGGCTGCAGGCCTTGCTCTCTTTCCAAT  6850
             ||||||||||||||||||||||||||||||||||||||||||||||||||||||||||||
Seq_2  6793  ACTATCTTCCATTCCATGGGCAAGGCAATCGCAGGCTGCAGGCCTTGCTCTCTTTCCAAT  6852

Seq_1  6851  TCCATCCAGATCTGCCATGGCACAGCACACGCCAACTGCCGAGGAGGGCTGCGGCAGTGA  6910
             ||||||||||||||||||||||||||||||||||||||||||||| ||||||||||||||
Seq_2  6853  TCCATCCAGATCTGCCATGGCACAGCACACGCCAACTGCCGAGGAAGGCTGCGGCAGTGA  6912

Seq_1  6911  TGAGAAAGAAAAAATAAATCTGATTTCCTTTTAAAAAATTTACCAACGTGAAA-GTTCAT  6969
             |||||||||||||| ||||||||||| |||||||||||||||||||||||||| ||||||
Seq_2  6913  TGAGAAAGAAAAAAAAAATCTGATTTTCTTTTAAAAAATTTACCAACGTGAAAAGTTCAT  6972

Seq_1  6970  AATACCCTTTTCAAATTGGGAGGGGGTTTATACAGAAGCAGACCTCAAATCTTAATTTGG  7029
             ||||||||||||||||||||||||||||||||||||||||||||||||||||||||||||
Seq_2  6973  AATACCCTTTTCAAATTGGGAGGGGGTTTATACAGAAGCAGACCTCAAATCTTAATTTGG  7032

Seq_1  7030  TCTCTCGGTCTCTTCTTGCCTTTGCAGCACAAATTTCTAAAGCCAGCCATGAATGCACTC  7089
             |||||||||||||||||||||||| |||||||||||||||| |||||||||||||    |
Seq_2  7033  TCTCTCGGTCTCTTCTTGCCTTTGTAGCACAAATTTCTAAAACCAGCCATGAATGACAAC  7092

Seq_1  7090  ATGGTTTACTCCCTGTGTACACAACATATGTATAGTAGGCAAGGTGTCATCTAAAATCAC  7149
             |                          |||||||||||||||||||||||||||||||||
Seq_2  7093  AC-------------------------ATGTATAGTAGGCAAGGTGTCATCTAAAATCAC  7127
```

```
Seq_1  7150  AAAACCATTGGGATTTCGTATTCAAAACACTTCCGTACAGCTAGAGCATCTCCACTCACG  7209
             ||||||||||||||||||||||||||||||||||||||||||||||||||||||||| ||
Seq_2  7128  AAAACCATTGGGATTTCGTATTCAAAACACTTCCGTACAGCTAGAGCATCTCCACTCGCG  7187

Seq_1  7210  TCCCCCCATTCGGCGTCTGGCGCTGCCGTTTTGTAGGCCGGATGGGGGGCACAAATATTT  7269
             ||||||||||||||||||||||||||||||||||||||||||||||||||||||||||||
Seq_2  7188  TCCCCCCATTCGGCGTCTGGCGCTGCCGTTTTGTAGGCCGGATGGGGGGCACAAATATTT  7247

Seq_1  7270  TAATCTGGGGATCCGCAGCCCTGCCCCCAATACCAAATCCAAAATAAATAAATCCAAAAA  7329
             |||| |||||||||||||||||||||||||||||||||| ||||||||||||||||||||
Seq_2  7248  TAATTTGGGGATCCGCAGCCCTGCCCCCAATACCAAATCTAAAATAAATAAATCCAAAAA  7307

Seq_1  7330  GGGATAAATAAATTTAAAAAGGGATAA-TTTAACTAGTTGGGGCGAAATTTATACAAAAT  7388
             ||||||||||||||||||||||||||| ||||||||||||||||||||||||||||||||
Seq_2  7308  GGGATAAATAAATTTAAAAAGGGATAAATTTAACTAGTTGGGGCGAAATTTATACAAAAT  7367

Seq_1  7389  TCTAAAAAAACAACTAATCTACTCGTCGTCGTCCCACTCCTCCCCATCCCCGTCCTTGTC  7448
             |||||||||||||||||||||||||| |||||||||||||||||||||||||||||||||
Seq_2  7368  TCTAAAAAAACAACTAATCTACTCGTTGTCGTCCCACTCCTCCCCATCCCCGTCCTTGTC  7427

Seq_1  7449  GAGCTCGCTGGTGAGGCCAAGGCAGCGCGTGCGTTCGGCTTACTCCTTCTTGTGCTCCCT  7508
             ||||||||||||||||||||||||||||||||||||||||||||||||||||||||||||
Seq_2  7428  GAGCTCGCTGGTGAGGCCAAGGCAGCGCGTGCGTTCGGCTTACTCCTTCTTGTGCTCCCT  7487

Seq_1  7509  CGCCGACCAGGCGCGGAAATGGTCAAAGTTTGACATAAAATTCCGCGTAGGCCTTACAAA  7568
             |||||||||||||  |||||||||||||||||||||||||||||||||||||||||||||
Seq_2  7488  CGCCGACCAGGCG--GAAATGGTCAAAGTTTGACATAAAATTCCGCGTAGGCCTTACAAA  7545

Seq_1  7569  TCTGGACGAGGGAGTATCTTGTTACTACGAGTCTAGGACTAACACTATTGTGCAAAAAGT  7628
              |||||| |||||||||||||||||||||||||||||||||||||||||||||||||| |
Seq_2  7546  CCTGGACAAGGGAGTATCTTGTTACTACGAGTCTAGGACTAACACTATTGTGCAAAAAAT  7605

Seq_1  7629  ATTGTTGTCAAAGCTGCTTACTGAACTTGGAGTCTAGTTAAAATACTCTTTATCATTTCT  7688
             ||||||||||||||||||| ||||||||||||||||||||||||||||||||||||||||
Seq_2  7606  ATTGTTGTCAAAGCTGCTTGCTGAACTTGGAGTCTAGTTAAAATACTCTTTATCATTTCT  7665

Seq_1  7689  AAATGAAGAGAAGAGAGTATTACATAAAAATTCCAAATTTAAGAAAAACATATGGGGTAA  7748
             ||||||||||||||||||||||||||||||||||||||||||||||||||||||||||||
Seq_2  7666  AAATGAAGAGAAGAGAGTATTACATAAAAATTCCAAATTTAAGAAAAACATATGGGGTAA  7725

Seq_1  7749  TCCAAGTATATCTACCCCGGCCTATGTTGATTTTGGTTGTATCATCTCATTTAAACTTGC  7808
             ||||||||||||||||||||||||||||||||||||||||||||||||||||||||||||
Seq_2  7726  TCCAAGTATATCTACCCCGGCCTATGTTGATTTTGGTTGTATCATCTCATTTAAACTTGC  7785

Seq_1  7809  TAGGGATACATGCATGCATAGAAGTATGTCGTTTTATAATATGAGATATAGATCGAAAGC  7868
             ||||||||||||||||||||||||||||||||||||||||||||||||  ||||||||||
Seq_2  7786  TAGGGATACATGCATGCATAGAAGTATGTCGTTTTATAATATGAGATA--GATCGAAAGC  7843

Seq_1  7869  TCAAAGCTCTGGTCTATTTAACGCCCACGTTGCAGGTACCTTACAAGCATAATTAGGATC  7928
             |||||||| ||||||||||||| ||||||||||||||||||||||||||||| |||||||
Seq_2  7844  TCAAAGCTTTGGTCTATTTAACACCCACGTTGCAGGTACCTTACAAGCATAACTAGGATC  7903

Seq_1  7929  CAAGATACACAATTGTCCAGCAAGAGATCC  7958
             ||||||||||||||||||||||||||||||
Seq_2  7904  CAAGATACACAATTGTCCAGCAAGAGATCC  7933
```

BdindelWSU_9, UPSTREAM

>Bradi3g03831

TTTCAATATTCCTAGGATATATCTGATTATGATGACGTTCGGTACTGCTGCTCTGGAGCCGACACATGTCTGGACTGCTGGCCGTTAAT
GACCATTGTTATCAAAATCCTGGATACTTCACTTAGAGGCATGTTGGTTTGTTTATGCAATTACTTTGAGCACACATGACAATCACATG
TTGCTATGAAAATTCTGGATAATTCACTCCCATTGCAGTCTATACTATCTTGATCTGCTTTACTTTAAGTTACTGAAATTATGTGCTTGC
AGGTGATTTTGGTTTCAATCACATACTGTGGGTATATAGTGGTCGCCGTGGTGTCCATTGCTGGGTTTGTGATAGTAGAGCAAGAAA
GTATGTTTAAGAGAACCTTTTTGTACCTGAAGTGAATGTTCTCTTTGTTTCTTGTCTGGTGATCATGAAATTCATCTCTGTTCTCATACT
AGGCTCAGCAATGAACAAAGGTCTGCAATTGCTGACTACTTCCGCGTCTATAAGGTGGTACATCTTGATTCATATCTCTAGCCCTTTTC
CACGCCTGTATTCCAAAATCTGTTGATCTCCATTACCTTTTTCAGGGTGGTGAGAATACATCGAAAAAGGTGTCGTTGACTGGGCCT
GTTCTCCACCCTTTCCTTGCGTATGCTCTAAACATAACCATCTCCTACTTGCATGTTGTATGTGTGAATTGTGGGAAACAAATATGTTAT
ATTGAAGCTTTATGCTGCTGCAGACGGTCATACACTGATGTTCTGAAATGCTTCTTTGAAGACAAGCTATTACATAGCCAACAACTATT
TTCATCAGAGGAGAGATGCAACAAGATACTTGAACTTATTCCTGATGAAAGTAAGAAATAAACCTTAAGAAGCTGAGACAATGTTCT
CCAAATATCACTTCTATCAACAAATTCTCATTCATCAAGTACGAGATGTGTTCAACATCATCCTAAACGGGTGCCTTTTCTAGATGTTGC
TAGTGAGCTCCATGACAAATGGCAAGGAAATAGACGCTCCTCTATTTCAAAGGAAGATGTAAATGCAGCAAGATGGGAGCAGTTAA
AGATGACTTTGCAGTCAGGAAAACACAAGGTTAGTGTTTAATCTCATGTCGCTCTATTCACTTCTATTGCATATTGTTAATTTCTACTGC
AGTAAGTATATATGTCTCTTGAGTATTCAAGGGGGATGCTGTGTGTAGTGTTCACTCTTCATAAATCACAGTTGCATCTTGTGATTCAG
CTTGCCGTGTCAAAATGTAGGATGAATAATAGTGGTAAATCATGTTTTTGTCTCAGGCGCAGGGGCTTCGCAGATGTGTAGAAGAGA
TTGTCTTCTCATACACGTATCCTAGACTTGACATGGAGGTAACAGAAAAATCATCACGAATACATGTTCATAGCAAGTGGATTATTAAT
ATCTTTTTCCTCTATTGATCAGACTTGATGTTGTATATCAGGTGTCAAAACACATGAATCATTTACTGAAAGCTCCGTTCTGCATACACC
CAAAGACAGGTAAAAACTAAGTAGATTTCTGGTTTAGTTTTCTAATGTAATAAGGTGACACGATTGCATGGACGAAAAAGCAGCGTG
GAGCTTGTTATCCATTAGAAGTTTCTACCGTGACAGTTTCTTGAATGATGCACACTTTTAGCACAGCACTGATTTACTTAATGCGTACA
CTTATGACTTTTGGAGGAGTCAAACCGTGCTCTCTTTTCTTTGTACAACTGTACATATGATTAATCTGTCACCTCATTAGTTCTAACGAC
CATTCTAACGTTTCTCCAGGACGTGTTTCGTTCCAATTGATCCCAATAATTGTGATGATTTTGATCCTGCGGCTGTTCCAACTCTATCAC
AGGTATTGTCTATAGAGCCAAACATAAAATAAAATAAAAAACATGCTAAAAAATTATTTGCGCCTTTTGATGTGTGATCATGCAATCC
TGACTTGTTGAAATTACCATACACGCCCAGCTATTAGGAGAGCTCAATGCTGCTGGTATGCAGATTGATTCTGAGAGTGGTAAGTGG
TTCTAGAAACTTGCACCTCTTGGGATTTGTGTTAAAGTGCTGCATCATTTTATAACTTGCAAATTACATTTTTGTAACATACAGCCTTGT
CTTGCATACTCCTCATAATCCTGTTCTGAATTGCTCAATTGCAGATTGGGAAAGAACATCCCTCGAGAAATCCATCAGATTTTTCAGAA
CATCCTTTCTGCAGCCAATGCTGAAAGCTTGCAAGGTATGCAGGGTGTTCGAAGTTCAGGGGGTTAAGTAAGGCAAAATACTCAGGG
CTGGTCCTGAGATTTCAGGGGCCCGGGGCGAAAATCAGAACCTGGACCCTTAATATAAACACTAAATAATAATCAATATATGTACTA
GTATAAAATTTCGTCAATAAAAATTTCTCGAACTCTTTAAAAATTTATCAACCAACCCTCTTTGCGATTCTATCAACTCATCTGCTAGTTT
CCTCTTTTTCATTTTTCACCATCGGATGCATAATTTTTAGATGACACGACGATGCTGAGACTTTAACAAACAATCTGCTTTTGAAGAAAT
GTTACTCCCTCCGGCCAGAATTACTTGTCGAAATATTACATGTATCTAGACGCTTTTTGCACATAGATACATTCAAATTTGGGCAAATT
TGAGACAAGTAATACCGGTCGGAGGGAGTAACAAACAATTGACCAATTTAGAACTAGTGCAAACCTACAAAATACTAAATCAGTTGA
GTAAAATGTACTGGTTAATCACGATTACTTGCCGACTCGGACGATGTGAAAATTGTGCTTGAGACGGTTTGCATATTGGTGAAGGTG
TCTATCTTGTCTCCTGATCCAGAGCCCTATCCTTAGGTTTGTTGTCTGCTCGCGCACTAGAAGCCTCCAGTAGGCATGGCTGTGTCAGT
CGACAGATTGTGCTTGAGATGGTTTGCATTTGTGCTGGAATAGAGTTGTATTGTTGAGCCTAAACCCTGTATACTTGGTTGGTAGACA
ATTTTTATCAACATGTTTTAATCTTCAAAAAAAAAAATAGCACTTTGCTATCTTGTGGTCCTGTCATTTTTGTAGTGCTGAAAAAAATGTC
AGTTTTTAATAACCATGTAAGTTTTTGCAGGAGGAACTGGAAAGTGCTTATAACGCCAAGCTCCAGCAGTCCAAGAATGCCTTGAATT
GGTAAAACTTACAGACACACACAAAGGCGGCATGCCATACAGTTATCCTAGTATCGCCCCCATGGATGACCATGAAATTGTTCGAGCT
TGCTCACAATCTTCTTTTGTGGGCATACTCGGCACAGCCAAATGTTAATGTATTGCAAGTTGTAACTATGATGTTAAAGATGAATTGCT
CTGAGACTTCATGCTTGGTGGTTTGAGCGACGCCGGATACGTTTCGCTGTGCATATAGAATATGTTTGGTCCTTGAATTTCTATATGCA
TAGCGAGACTTGTTTAGAGTCGCTGAAACTACCAAGCAAAAAGCCTTGGAGCACCAAGAGTATACTACTCGGGAAAAGAGATGTTA
GTTTGACGCATGTGATGTGAGTCATGGACCAAAATAGTGCTAGCCATGGGCTTGCTTTTATTTGACACTCCCAAGGTCCAACTGCGTA
TTGGTTGCGGAGTTACCCTGGTGGGATCGCAGAGGCAATAAATGTTAAATTTTATGAGTGTTTAATGAAAGGGCAATCTCTTTTTTTT
CACTTCTTTTTGAAAAGCACCTTTGAAATCTTCATCTTTACTCTTATAAAGAGCTGGGTTGGTGGTTATCTGTTCACCCTCAGTGAGGTT
GCCCCTTGAGTCGTCCAGAAATTGTTGCTCGGAAATAGTCTGTTCACAATCTTTTGACCAGGGTTTAAAATTTTCGTGAAATTCTGTTG

AAATTTTGAAGAAATACGGTCAAAAATTCAGTCTAGATCAAATAAAATATGAAATTGGTCAAAATGATTTAAAAAATCCTGGACGTTT
TTGTATGGTGTGAAATTTGAAACCCTGCTTTTGATTAGTTCATCTGATTGATATATATTTTGAGATATTTTTTTATTTTGCACAAATTTAG
CGACTATGATGACACGATCGTCTGAAATTCAAACAATTTACGCACGGAAATTTTAATTCCGTGGCAACCGCCAGCATTCGCAAAAGAA
GAATGCAAACTTGCAAACTTCACCCTTCAAATGTTCTGTATTCTATTATCACCCTCGCTGCCTCCTTCCGTCCTTGGGTGCTCCAGTCGT
TCTCCTTCCCCACGCGCGTTCATCCGTCGCCGCCGCGTCCAACCACGACCAGAGCAACGTGGGGACGCCAAGGCGTGAGCAATGGCG
GCGGGGGACGAGGAGCTGGAGTCGCTGCTCCGGAACTTCCACCGCTTCTCCCAGGTCAGCAGCACCCGCACCGCACCTCACCGCGG
CATCCCTCCCTCCCTACCACCGTCCCGCCGCTCCCCCGACTCGTTCGTTCGCCCTTCCAGTTCGTTTCGGGTCTTAGGTTCGGTTTGATG
CCACGTTCTGGTTCCGATGGAGCTCACCCTAGTCGTCGTAGCAATCGCCTGAACCTTCCGTGCTCGGCTCAGGTGAATTGGATGATGA
AATGCCTGCGAGATGGCGCGTGTCGAACTGAATAGATAATTCTCCGGCGACATTTGCGCAATCGAGGGAATTCACGTAGTAGGCCGT
CCATCTTCTGTGCCTATTTTGTTTTTAACTTTTGATCTTCCGGTTGGATGCCATGCTTGGCCAATGCTGTGGACCGTGGTGGTTAATTGG
TTGGTTATGCTGAAGAATGTCTCGTCGTATTGATCATTGAGAGATGATTTTGGTGGGGGGGATTCCACACTAGTTCAGCCTCAGTGCT
CGATTCTCACTGTAGGGCTCGAATCCCCTGCAATCGATTTCTAGAATTGGGATCCCAAGTGCTCAGCAAACCAAAATATATGGGTTGT
CTGCATGGCAGGAGCGTGCTTCTGTTGCAATGTTTACTGCTTCCAGAGCTCAAGTGTTGCCATAGATGTTCAATCTACCAGTTTTAATT
GCCTAAAGATGAATGATGACCTATATCCAAGCATTTTTCTTATAGTGTGTTGATTTTCTTTTCTATTGTGTGTTCACTGGTTCCTTGGTTT
TGAACAGGGTTATAAAGATGCACTAATGGAGGCCCAGGCTTTAAGGGTGGATTGCAACTCTGAATCCAAGAAGCGTGAAGCTCTTG
AGTCGCATATTGCAGATCTCAATAGAGGTTAACTGATGTTTCTGCGTTCTTCCGCTGTTCAGCTTCAGTTTGTTGTTTTCTGTTAATGTA
TATAGCGTAGTAGGCGATGATTTTCATTGCTTGCTCAGGTTGAAACAAGAATTGAGTTGGGCTTTTTTCTGTCGTGCAGATAATGAGC
GATTAAGAAGGCTGTACACTGAAACTTTATTCAAGTTCACCAACAAGGTAGGTGGCTACTTACTATAATGCTATGTGCAGTTACATGA
AGGCTGATGAATTTCTAATACTAAGATGATGTCACGATTAAATATATTTTTTCATCATCCGACGTTTTTCATTGTGGGGTCATCATCCG
AGTTAACTATTTGATACAGATACTCAGTTGTATTGATGGTATCAGCCTGAGTTCAATGCTCTCCTTTTTCACCTGTGAGATTGAGTTGA
TGGAAGGACTATAGTGTCTTGTCCTTGGGCTTCAGTGTACTCTTTTTTCATGGAAAAACAGGTTGAACGCGTGTTGTGGAGCTGTTGA
TTCCGATTGCCATTCATACTATATTAACATCTGGATTGTAAACTTCTTATGTTGGAATTTGGAAGTTGATAATAGATCAAGTTTAATGA
GAGATCAAGTGCTAGGTTTTATATGCAGCATTAATCATATCTGTCAAACTCTGGACAATTTTTTATGTGCTTGCTATTATTCAGATGAA
ATTTCACACAGAATCTCAAAATCTTAAGGAAGAATTGGGGAAAGCAAATAGCAGGTTGCTATCC

>BdiBd21-3.3G0047900

AAGCCGCAGCCGCCGCTTCCGTCAGCCGCAGCCGCCGGGCGCTGGATCCGCTTCGCCGGGTGCCATCGCCGCCCCGTCCATATCGGT
ACGCCTCCCACTCTGTATCTCCTCCTGCTCTCGCCTTCTCGTCCCTCGTCAAACGGCGGCGGCCCGGTCCGTGTAAGCTGCGTCAGCGG
CAGCGCGAGCCAGACAGAGGGGAAGGGATGGGCAGCCCCGAGCTGCCTGCCGAGTCTTGGGGGACCCCGGAAGGGATTTGGGGA
GCGGTTGAATTTGGGGATTCCCATTTGCAGCAATTAGTCCACAATAGCAATTAGTTTGTTCTTTATTTAGTTAGGAAACCTTGCTTTAT
TGGGTGGAATTTCCGAAGAGTCTGTTCTGTTTTGGGGGATTCTTTCTTCGCTATCCTGGTGAGCACTCAAAACCAAAATTGTTCTTTTC
TATTTGTTCAGTCAGATGCCGGTTTGCTTGCTTGCTATGTGGCACCTTTGTAATCTAATAAATGCATCCATTTTTTGTCTAGGTTCTTCTT
CAGCACACAAGGTTGATCTAGTCCTCCTCCTCTGGTATGGAGGATGAGAGGGCCATGGAGATCGATGGGCAGCAGCAGCAAGAAGA
CACCGCTGCTGTGCCCGAAGGCTTCAACGCCGATTACCTCCGAATATACTATGGTGAAGTGCTGTAGTCATTTTGTCTATCCAAAATCA
TAATGCGAGGAACTGATAGGGTAAAGAAGCTGAAATGAGAGGCAGATTTGAATTCCTTGTACTACCTCTGTTACAAAATACAAGAGT
ATGGTGTAATACTCAACACAAAGATGCCTCAAATTTGGAAGCAGTTTGCTAAACAATTCTCACCCCCAAGAGTTCTTCTTCACCAATTT
CTTGTACTTGCTTTCTTGCAAAAAAATAGAATAAAATTGGATGAATGTAAATAATTATGTCATTAGGTTCAAACTTAGTCTATTATAGA
TTATAGATCTGGTGTAGTTAAGAGGCTCTTTAACTGAGAAAAGCTTACAACTCATCTAGTGCTGATGGTTATTTTACAACTGCAGGAA
AGCTATTCCCATATGGTGATTTCTTCAAGTGGCTTTGCTATGGAAATGGTCTGTGGTCACACTTTACTACTTCAGTGTTGGTTCTAATT
GTCTTGCTCTGTGCTAACTGATTTTTTTTCTGTAGATGGAAAACATCCAGGATGTGATCAGTCATACATTGGGCGTCGGGAGCTTTCGT
TTACACTGGAGAATGACATCTATCTCCGGTTCCAGTCCTTTGACAGTGCAGCTGAACTGGAGAGCTCTATCAAAGAGAAGTGCCCTTT
CAAGATTGACATTGGACCTGTTTACAGCGTAGATGTATGATATAGCATTATCGAACATGCCAAATATTTAACTTGATTTAATTGTCTAT
ATTAAGCTTTGTGTGCTATTGCCTAATGAATTAAGTTTTGTAGCCTGCAAAGCGACATGCCTATGCCCAGTCTGGCAATAATGTCTTTG
CACCAGTTGAGAGAGAACTTATTTTTGATATTGTAAGAAATATGTCATGTTTTTTCTTCATCATCTGATGTGCGTGTCCTGTGTTATCTT
TGTTCAACTGTTGCTAACTGTGAGCATTTTCAATATTCCTAGGATATATCTGATTATGATGATGTTCGGTACTGCTGCTCTGGAGCCGA
CACATGTCTGGACTGCTGGCCGTTAATGACCATTGTTATCAAAATCCTGGATACTTCACTTAGAGGCATGTTGGTTTGTTTATGTAATT
ACTTTGAGCACACATGACAATCACATGTTGCTATGAAAATTCTGGATAATTCACTCCCATTGCAGTCTATACTATCTTGATCTGCTTTAC
TTTAAGTTACTGAAATTATGTGCTTGCAGGTGATTTTGGTTTCAATCACATACTGTGGGTATATAGTGGTCGCCGTGGTGTCCATTGCT
GGGTTTGTGATAGTAGAGCAAGAAAGTATGTTTAAGAGAACCTTTTTGTACCTGAAGTGAATGTTCTCTTTGTTTCTTGTCTGGTGATC

ATGAAATTCATCTCTGTTCTCGTACTAGGCTCAGCAATGAACAAAGGTCTGCAATTGCTGACTACTTCCGCGTCTATAAGGTGGTACAT
CTTGATTCATATCTCTAGCCCTTTTCCACACCTGTATTCCAAAATCTGTTGATCTCCATTACCTTTTTTCAGGGTGGTGAGAATACATCG
AAAAAGGTGTCGTTGACTGGGCCTGTTCTCCACCCTTTCCTTGCGTATGCTCTAAACATAACCATCTCTCCTACTTGCATGTTGTATGTG
TGAATTGTGGAAAACAAATATGTTATATTGAAGCTTTATGCTGCTGCAGACGGTCATACACTGATGTTCTGAAATGCTTCTTTGAAGA
CAAGCTATTACATAGCCAACAACTATTTTCATCAGAGGAGAGATGCAACAAGATACTTGAACTTATTCCTGATGAAAGTAAGAAATAA
ACCTTAAGAAGCTGACAATGTTCTCCAAATATCACTTCTATCAACAAATTCTCATTCATCAAGTACGAGATGTGTTCAACATCATCCTAA
ACGGGTGCCTTTTCTAGATGTTGCTAGTGAGCTCCATGACAAATGGCAAGGAAATAGACGCTCCTCTATTTCAAAGGAAGATGTAAA
TGCAGCAAGATGGGAGCAGTTAAAGACGACTTTGCAGTCAGGAAAACACAAGGTTAGCGTTTAATATCATGTCGCTCTATTCACTTCT
ATTGCATATTGTTAATTTCTACTGCAGTAAGTATATATGTCTCTTGAGTATTCAAGGGGGATGCTGTGTGTAGTATTCACTCTTCATGA
ATCACAGTTGCATCTTGTGATTCAGCTTGTCGTGTCAAAATGTAGGATGAATAATAGTGGTAAATCATGTTTTTATCTCAGGCGCAGG
GGCTTCGCAGATGTGTAGAAGAGATTGTCTTCTCATACACGTATCCTAGACTTGACATGGAGGTAACAGAAAAATCATCACGAATAC
ATGTTCATAGCAAGTGGATTATTAATATCTTTTTCCTCTATTGATCAGACTTGATGTTGTATATCAGGTGTCAAAACACATGAATCATTT
ACTGAAAGCCCCGTTCTGCATACACCCAAAGACAGGTAAAAACTAAGTAGATTTCTGGTTTAGTTTTCTAATGTAATAAGGTGACACG
ATTGCATGGACGAAAAAGCAGCGTGGAGCTTGTTATCCATTAGAAGTTTCTACCGTGACAGTTTCTTGAATGATGCACACTTTTAGCA
CAGCACTGATTCACTTAATGCGTACACTTATGACTTTTGGAGGAGTCAAACCATGCTCTCTTTTCTTTGTACAACTGTACATATGATTAA
TCTGTCACCTCATTAGTTCTAACGACCATTCTAACGTTTCTCCAGGACGTGTTTGCGTTCCAATTGATCCCAATAATTGTGATGATTTTG
ATCCTGCGGCTGTTCCAACTCTATCACAGGTATTGTCTATAGAGCCAAACATAAAATAAAATAAAAAACATGCTAAAAAATTATTTGG
GCCTTTTGATGTGTGATCATGCAATCCTGACTTGTTGAAATTACCATACGCGCCCAGCTATTAGGAGAGCTCAATGCTGCTGGTATGC
AGATTGATTCTGAGAGTGGTAAGTGGTTCTAGAAACTTGCACCTCTTGGGATTTGTGTTAAAGTGCTGCATCATTTTATAACTTGCAA
ATTACATTTTTGTAACATACAGCCTTGTCTTACATACTCCTCATAATCCTGTTCTGAATTGCTCAATTGCAGATTGGGAAAGAACATCCC
TCGAGAAATCCATCAGATTTTTCAGAACATCCTTTCTGCAGCCAATGCTGAAAGCTTGCAAGGTATGCAGGGTGTTCGAAGTTCAGGG
GGTTAAGTAAGGCAAAATACTCAGGGCCGGTCCTGAGATTTCAGGGGCCCGGGGCGAAAATCAGAACCTGGACCCTTAATATAAAC
ACTAAATAATAATCAATATATGTACTAGTATAAAATTTCGTCAATAAAAATTTCTCGAACTCTTTAAAAATTTATCAACCAACCCTCTTT
GCGATTCTATCAACTCATCTGCTAGTTTCCTCTTTTTCATTTTTCACCATCGGATGCATAATTTTTAGATGACACGACGATGCTGAAACT
TTAACAAACAATCTGCTTTTGAAGAAATGTTACTCCCTCCGGCCGGAATTACTTGTCGAAATATTACATGTATCTAGACGCTTTTTGCA
CATAGATACATTCAAATTTGAGACAAGTAATACCGGTCGGAGGGAGTAACAAACAATTGACCAATTTAGAACTAGTGCAAACCTACA
AAATACTAAATCAGTTGAGTAAAATGTGCTGGTTAATCACGATTACTTGCCTACTCGGACGATGTGAAAATTGTGCTTGAGACGGTTT
GCATATTGGTGAAGGTGTCTATCTTGTCTCCTGATCCAGAGCCCTATCCTTAGGTTTGTTGTCTGCTAGCGCACTAGAAGCCTCCAGTA
GGCATGGCTGTGTCAGTCGACAGATTGTGCTTGAGATGGTTTGCATTTGTGCTGGAATAGAGTTGTATTGTTGAGCCTAAACCCTGTA
TTCTTGGCTGGTAGACAATTTTTATCAACATGTTTTAATCTTCAAAAAAAAAAATACTTTGCTATCTTGTGGTCCTGTCATTTTTGTAGTG
CTGAAAAAAAATGTCAGTTTTTAATAACCATGTAAGTTTTTGCAGGAGGAACTGGAAAGCGCCTATAACGCCAAGCTCCAGCAGTCCA
AGAATGCCTTGAATTGGTAAAACTTACAGATACACACAAAGGCGGCATGCCATACAGTTATCCTAGTATCGCCCCCATGGATGACCAT
GAAATTGTTCGAGCTTGCTCACAATCTTCTTTTGTGGGCATACTCGGCACAGCCAAATGTTAATGTATTGCAAGTTGTAACTATGATGT
TAAAGATGAATTGCTCTGAGACTTCATGCTTGGTGGACTGAGCGACGCAGGATACGTTTCGCTGTGCATATAGAATAAGTTTGGTTCT
TGAATTTCTATATGCATAGCGAGACTTGTTTAGAGTCGCTGAAACCACCAAGCAAAAAGCCTTGGAGCACCAAGAGTATACTACTCG
GGAAAAGAGATGTTAGTTTGACGCATGTGATGTGAGTCATGGACCAAAATAGTGCTAGCCATGGGCTTGCTTTTATTTGACACTCCC
GAGGGCCAACTGCGTATTGGTTGCGGAGTTACCCTGGTGGGATCGCAGAGGCAATAAATGTTAAATTTTATGAGTGTTTAATGAAAG
GGAAATCTCTTTTTTTTTCCACTTCTTTTTGAAAAGCACCTTTGAAATCTTCATCTTTACTCTTATAAAGAGCTGGGTTGGTGGTTATCTG
TTCACCCTCAGGCCTTGTTCGGTTTCATGCAAATCCCCATGGATTGAGGGGGATTAGAGGGGATTGAGGGGAAAATGAACTAAAATC
TTGCTCAATCCCTGCCATTCCCCCTGGATTGAACGGGTTGGGGCATAAACGAACTAGCCCTCAGTGAGGTTGCCCCTTGAGTCGTCCA
GAAATTGTAGCTCGGAAATAGTTTGTTCACAATCTTTTGACCAGGGTTTAAAATTTTCATGAAATTCTGTTGAAATTTTGAAGAAATAC
GGTCAAAAATTCAGTCTAGATCAAATAAAATATGAAGTTGGTCAAAATTCATTCGAATTCGCTTGAAGCGGTACTAAAATGATTTAAA
AAATCCTGGACGTTTTTGTATGGTGTGAAATTTGAAACCCTGCTTTTGATTAGTTCATCTGATTGATATATATTTTGAGATATTTTTTTA
TTTTGCACAAATTTAGCGACTATGATGACACGATCGTCTGAAATTCAAACAATTTACGCACGGAAATTTTAATTCCGTAGCAACCGCCA
GCATTCGCAAAAGAAGAATGCAAACTTGCAAACTTCACCCTTCAAATGTTCTGTATTCTATGATCACCCTCGCTGCCTCCTTCCGTCCTT
GGGTGCTCCAGTCGTTCTCCTTCCCCACGCGCGTTCATCCGTGGCCGCCGCGTCCAACCACGACCAGAGCAACGTGGGGACGCCAAG
GCGTGAGCA

Alignment of Sequence_1: [Untitled Sequence #1] with Sequence_2: [Sequence Window #2]

Similarity : 4277/6117 (69.92 %)

```
Seq_1  1    ------------------------------------------------------------  0

Seq_2  1    AAGCCGCAGCCGCCGCTTCCGTCAGCCGCAGCCGCCGGGCGCTGGATCCGCTTCGCCGGG  60

Seq_1  1    ------------------------------------------------------------  0

Seq_2  61   TGCCATCGCCGCCCCGTCCATATCGGTACGCCTCCCACTCTGTATCTCCTCCTGCTCTCG  120

Seq_1  1    ------------------------------------------------------------  0

Seq_2  121  CCTTCTCGTCCCTCGTCAAACGGCGGCGGCCCGGTCCGTGTAAGCTGCGTCAGCGGCAGC  180

Seq_1  1    ------------------------------------------------------------  0

Seq_2  181  GCGAGCCAGACAGAGGGGAAGGGATGGGCAGCCCCGAGCTGCCTGCCGAGTCTTGGGGGA  240

Seq_1  1    ------------------------------------------------------------  0

Seq_2  241  CCCCGGAAGGGATTTGGGGAGCGGTTGAATTTGGGGATTCCCATTTGCAGCAATTAGTCC  300

Seq_1  1    ------------------------------------------------------------  0

Seq_2  301  ACAATAGCAATTAGTTTGTTCTTTATTTAGTTAGGAAACCTTGCTTTATTGGGTGGAATT  360

Seq_1  1    ------------------------------------------------------------  0

Seq_2  361  TCCGAAGAGTCTGTTCTGTTTTGGGGGATTCTTTCTTCGCTATCCTGGTGAGCACTCAAA  420

Seq_1  1    ------------------------------------------------------------  0

Seq_2  421  ACCAAAATTGTTCTTTTCTATTTGTTCAGTCAGATGCCGGTTTGCTTGCTTGCTATGTGG  480

Seq_1  1    ------------------------------------------------------------  0

Seq_2  481  CACCTTTGTAATCTAATAAATGCATCCATTTTTTGTCTAGGTTCTTCTTCAGCACACAAG  540

Seq_1  1    ------------------------------------------------------------  0

Seq_2  541  GTTGATCTAGTCCTCCTCCTCTGGTATGGAGGATGAGAGGGCCATGGAGATCGATGGGCA  600

Seq_1  1    ------------------------------------------------------------  0

Seq_2  601  GCAGCAGCAAGAAGACACCGCTGCTGTGCCCGAAGGCTTCAACGCCGATTACCTCCGAAT  660

Seq_1  1    ------------------------------------------------------------  0

Seq_2  661  ATACTATGGTGAAGTGCTGTAGTCATTTTGTCTATCCAAAATCATAATGCGAGGAACTGA  720

Seq_1  1    ------------------------------------------------------------  0

Seq_2  721  TAGGGTAAAGAAGCTGAAATGAGAGGCAGATTTGAATTCCTTGTACTACCTCTGTTACAA  780

Seq_1  1    ------------------------------------------------------------  0

Seq_2  781  AATACAAGAGTATGGTGTAATACTCAACACAAAGATGCCTCAAATTTGGAAGCAGTTTGC  840
```

```
Seq_1  1     ------------------------------------------------------------  0

Seq_2  841   TAAACAATTCTCACCCCCAAGAGTTCTTCTTCACCAATTTCTTGTACTTGCTTTCTTGCA  900

Seq_1  1     ------------------------------------------------------------  0

Seq_2  901   AAAAAATAGAATAAAATTGGATGAATGTAAATAATTATGTCATTAGGTTCAAACTTAGTC  960

Seq_1  1     ------------------------------------------------------------  0

Seq_2  961   TATTATAGATTATAGATCTGGTGTAGTTAAGAGGCTCTTTAACTGAGAAAAGCTTACAAC  1020

Seq_1  1     ------------------------------------------------------------  0

Seq_2  1021  TCATCTAGTGCTGATGGTTATTTTACAACTGCAGGAAAGCTATTCCCATATGGTGATTTC  1080

Seq_1  1     ------------------------------------------------------------  0

Seq_2  1081  TTCAAGTGGCTTTGCTATGGAAATGGTCTGTGGTCACACTTTACTACTTCAGTGTTGGTT  1140

Seq_1  1     ------------------------------------------------------------  0

Seq_2  1141  CTAATTGTCTTGCTCTGTGCTAACTGATTTTTTTTCTGTAGATGGAAAACATCCAGGATG  1200

Seq_1  1     ------------------------------------------------------------  0

Seq_2  1201  TGATCAGTCATACATTGGGCGTCGGGAGCTTTCGTTTACACTGGAGAATGACATCTATCT  1260

Seq_1  1     ------------------------------------------------------------  0

Seq_2  1261  CCGGTTCCAGTCCTTTGACAGTGCAGCTGAACTGGAGAGCTCTATCAAAGAGAAGTGCCC  1320

Seq_1  1     ------------------------------------------------------------  0

Seq_2  1321  TTTCAAGATTGACATTGGACCTGTTTACAGCGTAGATGTATGATATAGCATTATCGAACA  1380

Seq_1  1     ------------------------------------------------------------  0

Seq_2  1381  TGCCAAATATTTAACTTGATTTAATTGTCTATATTAAGCTTTGTGTGCTATTGCCTAATG  1440

Seq_1  1     ------------------------------------------------------------  0

Seq_2  1441  AATTAAGTTTTGTAGCCTGCAAAGCGACATGCCTATGCCCAGTCTGGCAATAATGTCTTT  1500

Seq_1  1     ------------------------------------------------------------  0

Seq_2  1501  GCACCAGTTGAGAGAGAACTTATTTTTGATATTGTAAGAAATATGTCATGTTTTTTCTTC  1560

Seq_1  1     ----------------------------------------------------------TT  2
                                                                       ||
Seq_2  1561  ATCATCTGATGTGCGTGTCCTGTGTTATCTTTGTTCAACTGTTGCTAACTGTGAGCATTT  1620

Seq_1  3     TCAATATTCCTAGGATATATCTGATTATGATGACGTTCGGTACTGCTGCTCTGGAGCCGA  62
             |||||||||||||||||||||||||||||||||| |||||||||||||||||||||||||
Seq_2  1621  TCAATATTCCTAGGATATATCTGATTATGATGATGTTCGGTACTGCTGCTCTGGAGCCGA  1680
```

```
Seq_1  63    CACATGTCTGGACTGCTGGCCGTTAATGACCATTGTTATCAAAATCCTGGATACTTCACT  122
             ||||||||||||||||||||||||||||||||||||||||||||||||||||||||||||
Seq_2  1681  CACATGTCTGGACTGCTGGCCGTTAATGACCATTGTTATCAAAATCCTGGATACTTCACT  1740

Seq_1  123   TAGAGGCATGTTGGTTTGTTTATGCAATTACTTTGAGCACACATGACAATCACATGTTGC  182
             |||||||||||||||||||||||| |||||||||||||||||||||||||||||||||||
Seq_2  1741  TAGAGGCATGTTGGTTTGTTTATGTAATTACTTTGAGCACACATGACAATCACATGTTGC  1800

Seq_1  183   TATGAAAATTCTGGATAATTCACTCCCATTGCAGTCTATACTATCTTGATCTGCTTTACT  242
             ||||||||||||||||||||||||||||||||||||||||||||||||||||||||||||
Seq_2  1801  TATGAAAATTCTGGATAATTCACTCCCATTGCAGTCTATACTATCTTGATCTGCTTTACT  1860

Seq_1  243   TTAAGTTACTGAAATTATGTGCTTGCAGGTGATTTTGGTTTCAATCACATACTGTGGGTA  302
             ||||||||||||||||||||||||||||||||||||||||||||||||||||||||||||
Seq_2  1861  TTAAGTTACTGAAATTATGTGCTTGCAGGTGATTTTGGTTTCAATCACATACTGTGGGTA  1920

Seq_1  303   TATAGTGGTCGCCGTGGTGTCCATTGCTGGGTTTGTGATAGTAGAGCAAGAAAGTATGTT  362
             ||||||||||||||||||||||||||||||||||||||||||||||||||||||||||||
Seq_2  1921  TATAGTGGTCGCCGTGGTGTCCATTGCTGGGTTTGTGATAGTAGAGCAAGAAAGTATGTT  1980

Seq_1  363   TAAGAGAACCTTTTTGTACCTGAAGTGAATGTTCTCTTTGTTTCTTGTCTGGTGATCATG  422
             ||||||||||||||||||||||||||||||||||||||||||||||||||||||||||||
Seq_2  1981  TAAGAGAACCTTTTTGTACCTGAAGTGAATGTTCTCTTTGTTTCTTGTCTGGTGATCATG  2040

Seq_1  423   AAATTCATCTCTGTTCTCATACTAGGCTCAGCAATGAACAAAGGTCTGCAATTGCTGACT  482
             |||||||||||||||||| |||||||||||||||||||||||||||||||||||||||||
Seq_2  2041  AAATTCATCTCTGTTCTCGTACTAGGCTCAGCAATGAACAAAGGTCTGCAATTGCTGACT  2100

Seq_1  483   ACTTCCGCGTCTATAAGGTGGTACATCTTGATTCATATCTCTAGCCCTTTTCCACGCCTG  542
             ||||||||||||||||||||||||||||||||||||||||||||||||||||||| ||||
Seq_2  2101  ACTTCCGCGTCTATAAGGTGGTACATCTTGATTCATATCTCTAGCCCTTTTCCACACCTG  2160

Seq_1  543   TATTCCAAAATCTGTTGATCTCCATTACCTTTTTTCAGGGTGGTGAGAATACATCGAAAA  602
             ||||||||||||||||||||||||||||||||||||||||||||||||||||||||||||
Seq_2  2161  TATTCCAAAATCTGTTGATCTCCATTACCTTTTTTCAGGGTGGTGAGAATACATCGAAAA  2220

Seq_1  603   AGGTGTCGTTGACTGGGCCTGTTCTCCACCCTTTCCTTGCGTATGCTCTAAACATAACCA  662
             ||||||||||||||||||||||||||||||||||||||||||||||||||||||||||||
Seq_2  2221  AGGTGTCGTTGACTGGGCCTGTTCTCCACCCTTTCCTTGCGTATGCTCTAAACATAACCA  2280

Seq_1  663   TCTC--CTACTTGCATGTTGTATGTGTGAATTGTGGGAAACAAATATGTTATATTGAAGC  720
             ||||  |||||||||||||||||||||||||||||| |||||||||||||||||||||||
Seq_2  2281  TCTCTCCTACTTGCATGTTGTATGTGTGAATTGTGGAAAACAAATATGTTATATTGAAGC  2340

Seq_1  721   TTTATGCTGCTGCAGACGGTCATACACTGATGTTCTGAAATGCTTCTTTGAAGACAAGCT  780
             ||||||||||||||||||||||||||||||||||||||||||||||||||||||||||||
Seq_2  2341  TTTATGCTGCTGCAGACGGTCATACACTGATGTTCTGAAATGCTTCTTTGAAGACAAGCT  2400

Seq_1  781   ATTACATAGCCAACAACTATTTTCATCAGAGGAGAGATGCAACAAGATACTTGAACTTAT  840
             ||||||||||||||||||||||||||||||||||||||||||||||||||||||||||||
Seq_2  2401  ATTACATAGCCAACAACTATTTTCATCAGAGGAGAGATGCAACAAGATACTTGAACTTAT  2460

Seq_1  841   TCCTGATGAAAGTAAGAAATAAACCTTAAGAAGCTGAGACAATGTTCTCCAAATATCACT  900
             |||||||||||||||||||||||||||||||||||||  |||||||||||||||||||||
Seq_2  2461  TCCTGATGAAAGTAAGAAATAAACCTTAAGAAGCTGA--CAATGTTCTCCAAATATCACT  2518
```

```
Seq_1  901   TCTATCAACAAATTCTCATTCATCAAGTACGAGATGTGTTCAACATCATCCTAAACGGGT  960
             ||||||||||||||||||||||||||||||||||||||||||||||||||||||||||||
Seq_2  2519  TCTATCAACAAATTCTCATTCATCAAGTACGAGATGTGTTCAACATCATCCTAAACGGGT  2578


Seq_1  961   GCCTTTTCTAGATGTTGCTAGTGAGCTCCATGACAAATGGCAAGGAAATAGACGCTCCTC  1020
             ||||||||||||||||||||||||||||||||||||||||||||||||||||||||||||
Seq_2  2579  GCCTTTTCTAGATGTTGCTAGTGAGCTCCATGACAAATGGCAAGGAAATAGACGCTCCTC  2638


Seq_1  1021  TATTTCAAAGGAAGATGTAAATGCAGCAAGATGGGAGCAGTTAAAGATGACTTTGCAGTC  1080
             ||||||||||||||||||||||||||||||||||||||||||||||| ||||||||||||
Seq_2  2639  TATTTCAAAGGAAGATGTAAATGCAGCAAGATGGGAGCAGTTAAAGACGACTTTGCAGTC  2698


Seq_1  1081  AGGAAAACACAAGGTTAGTGTTTAATCTCATGTCGCTCTATTCACTTCTATTGCATATTG  1140
             |||||||||||||||||| ||||||| |||||||||||||||||||||||||||||||||
Seq_2  2699  AGGAAAACACAAGGTTAGCGTTTAATATCATGTCGCTCTATTCACTTCTATTGCATATTG  2758


Seq_1  1141  TTAATTTCTACTGCAGTAAGTATATATGTCTCTTGAGTATTCAAGGGGGATGCTGTGTGT  1200
             ||||||||||||||||||||||||||||||||||||||||||||||||||||||||||||
Seq_2  2759  TTAATTTCTACTGCAGTAAGTATATATGTCTCTTGAGTATTCAAGGGGGATGCTGTGTGT  2818


Seq_1  1201  AGTGTTCACTCTTCATAAATCACAGTTGCATCTTGTGATTCAGCTTGCCGTGTCAAAATG  1260
             ||| |||||||||||| |||||||||||||||||||||||||||||| ||||||||||||
Seq_2  2819  AGTATTCACTCTTCATGAATCACAGTTGCATCTTGTGATTCAGCTTGTCGTGTCAAAATG  2878


Seq_1  1261  TAGGATGAATAATAGTGGTAAATCATGTTTTTGTCTCAGGCGCAGGGGCTTCGCAGATGT  1320
             |||||||||||||||||||||||||||||||| |||||||||||||||||||||||||||
Seq_2  2879  TAGGATGAATAATAGTGGTAAATCATGTTTTTATCTCAGGCGCAGGGGCTTCGCAGATGT  2938


Seq_1  1321  GTAGAAGAGATTGTCTTCTCATACACGTATCCTAGACTTGACATGGAGGTAACAGAAAAA  1380
             ||||||||||||||||||||||||||||||||||||||||||||||||||||||||||||
Seq_2  2939  GTAGAAGAGATTGTCTTCTCATACACGTATCCTAGACTTGACATGGAGGTAACAGAAAAA  2998


Seq_1  1381  TCATCACGAATACATGTTCATAGCAAGTGGATTATTAATATCTTTTTCCTCTATTGATCA  1440
             ||||||||||||||||||||||||||||||||||||||||||||||||||||||||||||
Seq_2  2999  TCATCACGAATACATGTTCATAGCAAGTGGATTATTAATATCTTTTTCCTCTATTGATCA  3058


Seq_1  1441  GACTTGATGTTGTATATCAGGTGTCAAAACACATGAATCATTTACTGAAAGCTCCGTTCT  1500
             |||||||||||||||||||||||||||||||||||||||||||||||||||| |||||||
Seq_2  3059  GACTTGATGTTGTATATCAGGTGTCAAAACACATGAATCATTTACTGAAAGCCCCGTTCT  3118


Seq_1  1501  GCATACACCCAAAGACAGGTAAAAACTAAGTAGATTTCTGGTTTAGTTTTCTAATGTAAT  1560
             ||||||||||||||||||||||||||||||||||||||||||||||||||||||||||||
Seq_2  3119  GCATACACCCAAAGACAGGTAAAAACTAAGTAGATTTCTGGTTTAGTTTTCTAATGTAAT  3178


Seq_1  1561  AAGGTGACACGATTGCATGGACGAAAAAGCAGCGTGGAGCTTGTTATCCATTAGAAGTTT  1620
             ||||||||||||||||||||||||||||||||||||||||||||||||||||||||||||
Seq_2  3179  AAGGTGACACGATTGCATGGACGAAAAAGCAGCGTGGAGCTTGTTATCCATTAGAAGTTT  3238


Seq_1  1621  CTACCGTGACAGTTTCTTGAATGATGCACACTTTTAGCACAGCACTGATTTACTTAATGC  1680
             |||||||||||||||||||||||||||||||||||||||||||||||||| |||||||||
Seq_2  3239  CTACCGTGACAGTTTCTTGAATGATGCACACTTTTAGCACAGCACTGATTCACTTAATGC  3298


Seq_1  1681  GTACACTTATGACTTTTGGAGGAGTCAAACCGTGCTCTCTTTTCTTTGTACAACTGTACA  1740
             ||||||||||||||||||||||||||||||| ||||||||||||||||||||||||||||
Seq_2  3299  GTACACTTATGACTTTTGGAGGAGTCAAACCATGCTCTCTTTTCTTTGTACAACTGTACA  3358
```

```
Seq_1  1741  TATGATTAATCTGTCACCTCATTAGTTCTAACGACCATTCTAACGTTTCTCCAGGACGTG  1800
             ||||||||||||||||||||||||||||||||||||||||||||||||||||||||||||
Seq_2  3359  TATGATTAATCTGTCACCTCATTAGTTCTAACGACCATTCTAACGTTTCTCCAGGACGTG  3418

Seq_1  1801  TTT-CGTTCCAATTGATCCCAATAATTGTGATGATTTTGATCCTGCGGCTGTTCCAACTC  1859
             ||| ||||||||||||||||||||||||||||||||||||||||||||||||||||||||
Seq_2  3419  TTTGCGTTCCAATTGATCCCAATAATTGTGATGATTTTGATCCTGCGGCTGTTCCAACTC  3478

Seq_1  1860  TATCACAGGTATTGTCTATAGAGCCAAACATAAAATAAAATAAAAAACATGCTAAAAAAT  1919
             ||||||||||||||||||||||||||||||||||||||||||||||||||||||||||||
Seq_2  3479  TATCACAGGTATTGTCTATAGAGCCAAACATAAAATAAAATAAAAAACATGCTAAAAAAT  3538

Seq_1  1920  TATTTGCGCCTTTTGATGTGTGATCATGCAATCCTGACTTGTTGAAATTACCATACACGC  1979
             |||||| ||||||||||||||||||||||||||||||||||||||||||||||||| |||
Seq_2  3539  TATTTGGGCCTTTTGATGTGTGATCATGCAATCCTGACTTGTTGAAATTACCATACGCGC  3598

Seq_1  1980  CCAGCTATTAGGAGAGCTCAATGCTGCTGGTATGCAGATTGATTCTGAGAGTGGTAAGTG  2039
             ||||||||||||||||||||||||||||||||||||||||||||||||||||||||||||
Seq_2  3599  CCAGCTATTAGGAGAGCTCAATGCTGCTGGTATGCAGATTGATTCTGAGAGTGGTAAGTG  3658

Seq_1  2040  GTTCTAGAAACTTGCACCTCTTGGGATTTGTGTTAAAGTGCTGCATCATTTTATAACTTG  2099
             ||||||||||||||||||||||||||||||||||||||||||||||||||||||||||||
Seq_2  3659  GTTCTAGAAACTTGCACCTCTTGGGATTTGTGTTAAAGTGCTGCATCATTTTATAACTTG  3718

Seq_1  2100  CAAATTACATTTTTGTAACATACAGCCTTGTCTTGCATACTCCTCATAATCCTGTTCTGA  2159
             |||||||||||||||||||||||||||||||||| |||||||||||||||||||||||||
Seq_2  3719  CAAATTACATTTTTGTAACATACAGCCTTGTCTTACATACTCCTCATAATCCTGTTCTGA  3778

Seq_1  2160  ATTGCTCAATTGCAGATTGGGAAAGAACATCCCTCGAGAAATCCATCAGATTTTTCAGAA  2219
             ||||||||||||||||||||||||||||||||||||||||||||||||||||||||||||
Seq_2  3779  ATTGCTCAATTGCAGATTGGGAAAGAACATCCCTCGAGAAATCCATCAGATTTTTCAGAA  3838

Seq_1  2220  CATCCTTTCTGCAGCCAATGCTGAAAGCTTGCAAGGTATGCAGGGTGTTCGAAGTTCAGG  2279
             ||||||||||||||||||||||||||||||||||||||||||||||||||||||||||||
Seq_2  3839  CATCCTTTCTGCAGCCAATGCTGAAAGCTTGCAAGGTATGCAGGGTGTTCGAAGTTCAGG  3898

Seq_1  2280  GGGTTAAGTAAGGCAAAATACTCAGGGCTGGTCCTGAGATTTCAGGGGCCCGGGGCGAAA  2339
             |||||||||||||||||||||||||||| |||||||||||||||||||||||||||||||
Seq_2  3899  GGGTTAAGTAAGGCAAAATACTCAGGGCCGGTCCTGAGATTTCAGGGGCCCGGGGCGAAA  3958

Seq_1  2340  ATCAGAACCTGGACCCTTAATATAAACACTAAATAATAATCAATATATGTACTAGTATAA  2399
             ||||||||||||||||||||||||||||||||||||||||||||||||||||||||||||
Seq_2  3959  ATCAGAACCTGGACCCTTAATATAAACACTAAATAATAATCAATATATGTACTAGTATAA  4018

Seq_1  2400  AATTTCGTCAATAAAAAATTTCTCGAACTCTTTAAAAATTTATCAACCAACCCTCTTTGCG  2459
             ||||||||||||||||||||||||||||||||||||||||||||||||||||||||||||
Seq_2  4019  AATTTCGTCAATAAAAAATTTCTCGAACTCTTTAAAAATTTATCAACCAACCCTCTTTGCG  4078

Seq_1  2460  ATTCTATCAACTCATCTGCTAGTTTCCTCTTTTTCATTTTTCACCATCGGATGCATAATT  2519
             ||||||||||||||||||||||||||||||||||||||||||||||||||||||||||||
Seq_2  4079  ATTCTATCAACTCATCTGCTAGTTTCCTCTTTTTCATTTTTCACCATCGGATGCATAATT  4138

Seq_1  2520  TTTAGATGACACGACGATGCTGAGACTTTAACAAACAATCTGCTTTTGAAGAAATGTTAC  2579
             ||||||||||||||||||||||| ||||||||||||||||||||||||||||||||||||
Seq_2  4139  TTTAGATGACACGACGATGCTGAAACTTTAACAAACAATCTGCTTTTGAAGAAATGTTAC  4198
```

```
Seq_1  2580  TCCCTCCGGCCAGAATTACTTGTCGAAATATTACATGTATCTAGACGCTTTTTGCACATA  2639
             ||||||||||| ||||||||||||||||||||||||||||||||||||||||||||||||
Seq_2  4199  TCCCTCCGGCCGGAATTACTTGTCGAAATATTACATGTATCTAGACGCTTTTTGCACATA  4258

Seq_1  2640  GATACATTCAAATTTGGGCAAATTTGAGACAAGTAATACCGGTCGGAGGGAGTAACAAAC  2699
             ||||||||||||||||          ||||||||||||||||||||||||||||||||||
Seq_2  4259  GATACATTCAAATTTG----------AGACAAGTAATACCGGTCGGAGGGAGTAACAAAC  4308

Seq_1  2700  AATTGACCAATTTAGAACTAGTGCAAACCTACAAAATACTAAATCAGTTGAGTAAAATGT  2759
             ||||||||||||||||||||||||||||||||||||||||||||||||||||||||||||
Seq_2  4309  AATTGACCAATTTAGAACTAGTGCAAACCTACAAAATACTAAATCAGTTGAGTAAAATGT  4368

Seq_1  2760  ACTGGTTAATCACGATTACTTGCCGACTCGGACGATGTGAAAATTGTGCTTGAGACGGTT  2819
              ||||||||||||||||||||||| |||||||||||||||||||||||||||||||||||
Seq_2  4369  GCTGGTTAATCACGATTACTTGCCTACTCGGACGATGTGAAAATTGTGCTTGAGACGGTT  4428

Seq_1  2820  TGCATATTGGTGAAGGTGTCTATCTTGTCTCCTGATCCAGAGCCCTATCCTTAGGTTTGT  2879
             ||||||||||||||||||||||||||||||||||||||||||||||||||||||||||||
Seq_2  4429  TGCATATTGGTGAAGGTGTCTATCTTGTCTCCTGATCCAGAGCCCTATCCTTAGGTTTGT  4488

Seq_1  2880  TGTCTGCTCGCGCACTAGAAGCCTCCAGTAGGCATGGCTGTGTCAGTCGACAGATTGTGC  2939
             |||||||| |||||||||||||||||||||||||||||||||||||||||||||||||||
Seq_2  4489  TGTCTGCTAGCGCACTAGAAGCCTCCAGTAGGCATGGCTGTGTCAGTCGACAGATTGTGC  4548

Seq_1  2940  TTGAGATGGTTTGCATTTGTGCTGGAATAGAGTTGTATTGTTGAGCCTAAACCCTGTATA  2999
             ||||||||||||||||||||||||||||||||||||||||||||||||||||||||||| 
Seq_2  4549  TTGAGATGGTTTGCATTTGTGCTGGAATAGAGTTGTATTGTTGAGCCTAAACCCTGTATT  4608

Seq_1  3000  CTTGGTTGGTAGACAATTTTTATCAACATGTTTTAATCTTCAAAAAAAAAATAGCACTTT  3059
             ||||| |||||||||||||||||||||||||||||||||||||||||||   ||||
Seq_2  4609  CTTGGCTGGTAGACAATTTTTATCAACATGTTTTAATCTTCAAAAAAAAAATA---CTTT  4665

Seq_1  3060  GCTATCTTGTGGTCCTGTCATTTTTGTAGTGCTGAAAAAAATGTCAGTTTTTAATAACCA  3119
             ||||||||||||||||||||||||||||||||||||||||||||||||||||||||||||
Seq_2  4666  GCTATCTTGTGGTCCTGTCATTTTTGTAGTGCTGAAAAAAATGTCAGTTTTTAATAACCA  4725

Seq_1  3120  TGTAAGTTTTTGCAGGAGGAACTGGAAAGTGCTTATAACGCCAAGCTCCAGCAGTCCAAG  3179
             ||||||||||||||||||||||||||||| || |||||||||||||||||||||||||||
Seq_2  4726  TGTAAGTTTTTGCAGGAGGAACTGGAAAGCGCCTATAACGCCAAGCTCCAGCAGTCCAAG  4785

Seq_1  3180  AATGCCTTGAATTGGTAAAACTTACAGACACACACAAAGGCGGCATGCCATACAGTTATC  3239
             |||||||||||||||||||||||||||| |||||||||||||||||||||||||||||||
Seq_2  4786  AATGCCTTGAATTGGTAAAACTTACAGATACACACAAAGGCGGCATGCCATACAGTTATC  4845

Seq_1  3240  CTAGTATCGCCCCCATGGATGACCATGAAATTGTTCGAGCTTGCTCACAATCTTCTTTTG  3299
             ||||||||||||||||||||||||||||||||||||||||||||||||||||||||||||
Seq_2  4846  CTAGTATCGCCCCCATGGATGACCATGAAATTGTTCGAGCTTGCTCACAATCTTCTTTTG  4905

Seq_1  3300  TGGGCATACTCGGCACAGCCAAATGTTAATGTATTGCAAGTTGTAACTATGATGTTAAAG  3359
             ||||||||||||||||||||||||||||||||||||||||||||||||||||||||||||
Seq_2  4906  TGGGCATACTCGGCACAGCCAAATGTTAATGTATTGCAAGTTGTAACTATGATGTTAAAG  4965

Seq_1  3360  ATGAATTGCTCTGAGACTTCATGCTTGGTGG-TTTGAGCGACGCCGGATACGTTTCGCTG  3418
             |||||||||||||||||||||||||||||||   |||||||||| |||||||||||||||
Seq_2  4966  ATGAATTGCTCTGAGACTTCATGCTTGGTGGAC-TGAGCGACGCAGGATACGTTTCGCTG  5024
```

```
Seq_1  3419  TGCATATAGAATATGTTTGGTCCTTGAATTTCTATATGCATAGCGAGACTTGTTTAGAGT  3478
             ||||||||||||| ||||||| ||||||||||||||||||||||||||||||||||||||
Seq_2  5025  TGCATATAGAATAAGTTTGGTTCTTGAATTTCTATATGCATAGCGAGACTTGTTTAGAGT  5084

Seq_1  3479  CGCTGAAACTACCAAGCAAAAAGCCTTGGAGCACCAAGAGTATACTACTCGGGAAAAGAG  3538
             ||||||||| ||||||||||||||||||||||||||||||||||||||||||||||||||
Seq_2  5085  CGCTGAAACCACCAAGCAAAAAGCCTTGGAGCACCAAGAGTATACTACTCGGGAAAAGAG  5144

Seq_1  3539  ATGTTAGTTTGACGCATGTGATGTGAGTCATGGACCAAAATAGTGCTAGCCATGGGCTTG  3598
             ||||||||||||||||||||||||||||||||||||||||||||||||||||||||||||
Seq_2  5145  ATGTTAGTTTGACGCATGTGATGTGAGTCATGGACCAAAATAGTGCTAGCCATGGGCTTG  5204

Seq_1  3599  CTTTTATTTGACACTCCCAAGGTCCAACTGCGTATTGGTTGCGGAGTTACCCTGGTGGGA  3658
             |||||||||||||||||| ||| |||||||||||||||||||||||||||||||||||||
Seq_2  5205  CTTTTATTTGACACTCCCGAGGGCCAACTGCGTATTGGTTGCGGAGTTACCCTGGTGGGA  5264

Seq_1  3659  TCGCAGAGGCAATAAATGTTAAATTTTATGAGTGTTTAATGAAAGGGCAATCTCTTTTTT  3718
             ||||||||||||||||||||||||||||||||||||||||||||||| ||||||||||||
Seq_2  5265  TCGCAGAGGCAATAAATGTTAAATTTTATGAGTGTTTAATGAAAGGGAAATCTCTTTTTT  5324

Seq_1  3719  TT--CACTTCTTTTTGAAAAGCACCTTTGAAATCTTCATCTTTACTCTTATAAAGAGCTG  3776
             ||  ||||||||||||||||||||||||||||||||||||||||||||||||||||||||
Seq_2  5325  TTTCCACTTCTTTTTGAAAAGCACCTTTGAAATCTTCATCTTTACTCTTATAAAGAGCTG  5384

Seq_1  3777  GGTTGGTGGTTATCTGTTCACCCTCAG---------------------------------  3803
             |||||||||||||||||||||||||||
Seq_2  5385  GGTTGGTGGTTATCTGTTCACCCTCAGGCCTTGTTCGGTTTCATGCAAATCCCCATGGAT  5444

Seq_1  3804  ------------------------------------------------------------  3803

Seq_2  5445  TGAGGGGGATTAGAGGGGATTGAGGGGAAAATGAACTAAAATCTTGCTCAATCCCTGCCA  5504

Seq_1  3804  -----------------------------------------------TGAGGTTGCCCCT  3816
                                                            |||||||||||||
Seq_2  5505  TTCCCCCTGGATTGAACGGGTTGGGGCATAAACGAACTAGCCCTCAGTGAGGTTGCCCCT  5564

Seq_1  3817  TGAGTCGTCCAGAAATTGTTGCTCGGAAATAGTCTGTTCACAATCTTTTGACCAGGGTTT  3876
             ||||||||||||||||||| ||||||||||||| ||||||||||||||||||||||||||
Seq_2  5565  TGAGTCGTCCAGAAATTGTAGCTCGGAAATAGTTTGTTCACAATCTTTTGACCAGGGTTT  5624

Seq_1  3877  AAAATTTTCGTGAAATTCTGTTGAAATTTTGAAGAAATACGGTCAAAAATTCAGTCTAGA  3936
             ||||||||| ||||||||||||||||||||||||||||||||||||||||||||||||||
Seq_2  5625  AAAATTTTCATGAAATTCTGTTGAAATTTTGAAGAAATACGGTCAAAAATTCAGTCTAGA  5684

Seq_1  3937  TCAAATAAAATATGAAATTGGTC--------------------------------AAAAT  3964
             |||||||||||||||| ||||||                                |||||
Seq_2  5685  TCAAATAAAATATGAAGTTGGTCAAAATTCATTCGAATTCGCTTGAAGCGGTACTAAAAT  5744

Seq_1  3965  GATTTAAAAAATCCTGGACGTTTTTGTATGGTGTGAAATTTGAAACCCTGCTTTTGATTA  4024
             ||||||||||||||||||||||||||||||||||||||||||||||||||||||||||||
Seq_2  5745  GATTTAAAAAATCCTGGACGTTTTTGTATGGTGTGAAATTTGAAACCCTGCTTTTGATTA  5804

Seq_1  4025  GTTCATCTGATTGATATATATATTTGAGATATTTTTTTATTTTGCACAAATTTAGCGACTA  4084
             ||||||||||||||||||||||||||||||||||||||||||||||||||||||||||||
Seq_2  5805  GTTCATCTGATTGATATATATATTTGAGATATTTTTTTATTTTGCACAAATTTAGCGACTA  5864
```

```
Seq_1  4085  TGATGACACGATCGTCTGAAATTCAAACAATTTACGCACGGAAATTTTAATTCCGTGGCA  4144
             |||||||||||||||||||||||||||||||||||||||||||||||||||||||| |||
Seq_2  5865  TGATGACACGATCGTCTGAAATTCAAACAATTTACGCACGGAAATTTTAATTCCGTAGCA  5924

Seq_1  4145  ACCGCCAGCATTCGCAAAAGAAGAATGCAAACTTGCAAACTTCACCCTTCAAATGTTCTG  4204
             ||||||||||||||||||||||||||||||||||||||||||||||||||||||||||||
Seq_2  5925  ACCGCCAGCATTCGCAAAAGAAGAATGCAAACTTGCAAACTTCACCCTTCAAATGTTCTG  5984

Seq_1  4205  TATTCTATTATCACCCTCGCTGCCTCCTTCCGTCCTTGGGTGCTCCAGTCGTTCTCCTTC  4264
             |||||||| |||||||||||||||||||||||||||||||||||||||||||||||||||
Seq_2  5985  TATTCTATGATCACCCTCGCTGCCTCCTTCCGTCCTTGGGTGCTCCAGTCGTTCTCCTTC  6044

Seq_1  4265  CCCACGCGCGTTCATCCGTCGCCGCCGCGTCCAACCACGACCAGAGCAACGTGGGGACGC  4324
             ||||||||||||||||||| ||||||||||||||||||||||||||||||||||||||||
Seq_2  6045  CCCACGCGCGTTCATCCGTGGCCGCCGCGTCCAACCACGACCAGAGCAACGTGGGGACGC  6104

Seq_1  4325  CAAGGCGTGAGCAATGGCGGCGGGGGACGAGGAGCTGGAGTCGCTGCTCCGGAACTTCCA  4384
             |||||||||||||
Seq_2  6105  CAAGGCGTGAGCA-----------------------------------------------  6117

Seq_1  4385  CCGCTTCTCCCAGGTCAGCAGCACCCGCACCGCACCTCACCGCGGCATCCCTCCCTCCCT  4444

Seq_2  6118  ------------------------------------------------------------  6117

Seq_1  4445  ACCACCGTCCCGCCGCTCCCCCGACTCGTTCGTTCGCCCTTCCAGTTCGTTTCGGGTCTT  4504

Seq_2  6118  ------------------------------------------------------------  6117

Seq_1  4505  AGGTTCGGTTTGATGCCACGTTCTGGTTCCGATGGAGCTCACCCTAGTCGTCGTAGCAAT  4564

Seq_2  6118  ------------------------------------------------------------  6117

Seq_1  4565  CGCCTGAACCTTCCGTGCTCGGCTCAGGTGAATTGGATGATGAAATGCCTGCGAGATGGC  4624

Seq_2  6118  ------------------------------------------------------------  6117

Seq_1  4625  GCGTGTCGAACTGAATAGATAATTCTCCGGCGACATTTGCGCAATCGAGGGAATTCACGT  4684

Seq_2  6118  ------------------------------------------------------------  6117

Seq_1  4685  AGTAGGCCGTCCATCTTCTGTGCCTATTTTGTTTTTAACTTTTGATCTTCCGGTTGGATG  4744

Seq_2  6118  ------------------------------------------------------------  6117

Seq_1  4745  CCATGCTTGGCCAATGCTGTGGACCGTGGTGGTTAATTGGTTGGTTATGCTGAAGAATGT  4804

Seq_2  6118  ------------------------------------------------------------  6117

Seq_1  4805  CTCGTCGTATTGATCATTGAGAGATGATTTTGGTGGGGGGGATTCCACACTAGTTCAGCC  4864

Seq_2  6118  ------------------------------------------------------------  6117

Seq_1  4865  TCAGTGCTCGATTCTCACTGTAGGGCTCGAATCCCCTGCAATCGATTTCTAGAATTGGGA  4924

Seq_2  6118  ------------------------------------------------------------  6117
```

```
Seq_1  4925  TCCCAAGTGCTCAGCAAACCAAAATATATGGGTTGTCTGCATGGCAGGAGCGTGCTTCTG  4984

Seq_2  6118  ------------------------------------------------------------  6117

Seq_1  4985  TTGCAATGTTTACTGCTTCCAGAGCTCAAGTGTTGCCATAGATGTTCAATCTACCAGTTT  5044

Seq_2  6118  ------------------------------------------------------------  6117

Seq_1  5045  TAATTGCCTAAAGATGAATGATGACCTATATCCAAGCATTTTTCTTATAGTGTGTTGATT  5104

Seq_2  6118  ------------------------------------------------------------  6117

Seq_1  5105  TTCTTTTCTATTGTGTGTTCACTGGTTCCTTGGTTTTGAACAGGGTTATAAAGATGCACT  5164

Seq_2  6118  ------------------------------------------------------------  6117

Seq_1  5165  AATGGAGGCCCAGGCTTTAAGGGTGGATTGCAACTCTGAATCCAAGAAGCGTGAAGCTCT  5224

Seq_2  6118  ------------------------------------------------------------  6117

Seq_1  5225  TGAGTCGCATATTGCAGATCTCAATAGAGGTTAACTGATGTTTCTGCGTTCTTCCGCTGT  5284

Seq_2  6118  ------------------------------------------------------------  6117

Seq_1  5285  TCAGCTTCAGTTTGTTGTTTTCTGTTAATGTATATAGCGTAGTAGGCGATGATTTTCATT  5344

Seq_2  6118  ------------------------------------------------------------  6117

Seq_1  5345  GCTTGCTCAGGTTGAAACAAGAATTGAGTTGGGCTTTTTTCTGTCGTGCAGATAATGAGC  5404

Seq_2  6118  ------------------------------------------------------------  6117

Seq_1  5405  GATTAAGAAGGCTGTACACTGAAACTTTATTCAAGTTCACCAACAAGGTAGGTGGCTACT  5464

Seq_2  6118  ------------------------------------------------------------  6117

Seq_1  5465  TACTATAATGCTATGTGCAGTTACATGAAGGCTGATGAATTTCTAATACTAAGATGATGT  5524

Seq_2  6118  ------------------------------------------------------------  6117

Seq_1  5525  CACGATTAAATATATTTTTTCATCATCCGACGTTTTTCATTGTGGGGTCATCATCCGAGT  5584

Seq_2  6118  ------------------------------------------------------------  6117

Seq_1  5585  TAACTATTTGATACAGATACTCAGTTGTATTGATGGTATCAGCCTGAGTTCAATGCTCTC  5644

Seq_2  6118  ------------------------------------------------------------  6117

Seq_1  5645  CTTTTTCACCTGTGAGATTGAGTTGATGGAAGGACTATAGTGTCTTGTCCTTGGGCTTCA  5704

Seq_2  6118  ------------------------------------------------------------  6117

Seq_1  5705  GTGTACTCTTTTTTCATGGAAAAACAGGTTGAACGCGTGTTGTGGAGCTGTTGATTCCGA  5764

Seq_2  6118  ------------------------------------------------------------  6117
```

```
Seq_1  5765  TTGCCATTCATACTATATTAACATCTGGATTGTAAACTTCTTATGTTGGAATTTGGAAGT  5824

Seq_2  6118  ------------------------------------------------------------  6117

Seq_1  5825  TGATAATAGATCAAGTTTAATGAGAGATCAAGTGCTAGGTTTTATATGCAGCATTAATCA  5884

Seq_2  6118  ------------------------------------------------------------  6117

Seq_1  5885  TATCTGTCAAACTCTGGACAATTTTTTATGTGCTTGCTATTATTCAGATGAAATTTCACA  5944

Seq_2  6118  ------------------------------------------------------------  6117

Seq_1  5945  CAGAATCTCAAAATCTTAAGGAAGAATTGGGGAAAGCAAATAGCAGGTTGCTATCC  6000

Seq_2  6118  --------------------------------------------------------  6117
```

BdindelWSU_10, UPSTREAM

>Bradi3g08040

ATCAGCGACACTTATTATGAATAAGAGGGAGTAGTAACCTAGTAACCTTTGAGTGGTCTCAGTTACTCATTAATTGACGAGACATTCG
TTTTGATGTTTGAATAACTCTGTGTAACGAATTGTTTTGAATAGTTCTTGTCCAATAACAATCTACATATATTTTGTTTACACTTACACTA
AAAAAATAATACATCACTTCTAAATTGTTCAGCGCTTTCGGTACTTTGTTTAGTCGTATATGTATCTTTTCAATAATCTACTTATCACTAA
AAAGATATATTATGACATTTGATTACATATTTTTCCTCCTTTTTTTTACAAAATGCTAATGGTCAAATCATGTACCATGGACTCTACTTTT
AAAAAAAGTCTTGCGTGTCTTAAACTCGGTATGAAAAAATTAAAGAGAATCGTCTGCATGTTATTTTGAACTTGAGATCTTAAATTAA
AATATATATATCAATACTGCATTATACTCCCTCCGTCCCATAATAAGTGACTCAAATTTGTCCAAATATGAATGTATCTATGTCTAAAAA
GAGTCTAGATACATGTAATAGAAAGTCACTTAATATGGGACGGAGGGAGTAGATATTTACTCTTACAACTCGTTTGATTCCCATCATC
GGGGCTTGAAATTCATTTCATATTCCAAAAAACAACATGTTTGGTTGTTACAGAATTGAGTGAAATAAAATCAATCTGAGATTTCATA
AGATCACCAATATCAATCTGAAATGAACAATGGCTAAGCTACCACACGTCCACACGGGCTGTTAAATTTTGACTGATGCTTAAACTGA
GGTTCATGTAGGGCTCATTTCCCCCCTCCTCTATTCCTTCTTCCACATCCTTGCTACAGCTCCGAGCCCTCCCTCGGCCGCTTCCTTCCCT
CCCCCTTCTCTTCGGCCGGCCAAAGAGAAGGGGGAGAGGAGGTCCGGCCTGGTCCTCTCCCATATATGTATATTAAGGTAGTTGCCT
TAGAGGCTTGTTCCTGTGGATCTGCATCGCCGGATTTAAGGCCCCGGTGGTCCCTTCGAAGAGGATTTTTTGCCGTCGCTACCAATAC
TGAGTTTGGTAATGGTAGCCTCGGTTCCATTTCTTCCCTTGATCCCCTCCTAAATAATTGCCTCCTTCTAGATCTGGCTCCGTATGGTTT
CTTGCTTCTCTTCAATTTCGGAACCAGATCTGTGGTTTGTTTCTTTTTTCTCATCAAATCCCCTGATTCTTGGTGTCCCTTGATCTTCCGG
AGTGGTTATAGTGGATATATTCTCTCTGGAGATCCCCTTTTATTAGTGCATATCTCTCCACCTAAGAGATCTTGCGGGCTGGCTGGGA
GGGAAGGCATGCTTCCCCATCGCATCTCTTGGTCTTCCTTCTGGTCTGCTCTGGTGAAGTTCCTACTTTCAATTTCATGCACTGGAGCA
AGGCTGGTCTTTGTCACTGGATCTATTCGTTTGCTGTTCTTTGTTCCTCTCAACCTCCAGATCGGAGGCCTGCCAGGCGGTCTTGGAAC
AAGACATCAACGGATCGGAGACGGGGACTTTGCTTTGATCTCGATCTGGTGGTTGCTGCCCATTTCAGGGGCTATGATACTTTGGTT
GTCGATGATGGAGGTTATTCAAGATATATGGCATCGTTAACATCCTCTGCCTCCCGAGTTCGTGAAGGCATTTTGTTCTCGTGGACAC
CTCGCTTGCTGGTGGCGGCTGGCAGTTCGCTCCTCTACGACGGCGTGTGACTCAGTCGGCGACGAGGAAGAAGCGGGAGCACGTGT
CAACCCGAAGCGTGTATGTGGACGCATGGATCTTGCCCGTCTGGGAGTTAGGCAGGTGTCCTTAGACATGGTTGTAAATCTTCTATTT
CTTTGTGTCCTTTCATGTATCGGGTTGTAATCCGTAGGTCTTTTGTACCTACTTGGTTTTAATGAAATGCAGGGTTCTTTAAAAAAAAA
ACTGAGGTTCACGTTGAAACACACGTTGCAAATTACGACTCCACGAGGGTTCTCTGCACAAAAGTCGGAATTCGGACCAACGGCGAT
CCACGAGGTAGCTCGTCGTCTTCCTGGCAGAAGCTGCCCTTCCTCTGTTCCCGGCTTCTCGCCCCGGCCCCAAGCTCAGCCATCTGCCG
GAATCCAGGGAACTTCGTCTCGAACCTTCTCGGAAGGGACCACAACCCCATTCTTCCTCCTCCTCCCCAAAACCCTAGATCCCCAAACC
CACCCGGCCACCGCTTC

>BdiBd21-3.3G0108000

TACCCTTTTTTTTGGTACGTATACATGCCTGCTCTCTTTTTAGTTTCAGTGTGCTCAGATAAAAACTTAAGCAAGTTACTGAAATGAATA
TATGGTGTATGTACTTCCATTTATGTATGACTGCATCGGAGCTACAGATGGTACACATATTCCATTAAAACTACCATTGCATATGCAAG
AAGAATATAAAGCAAACCCTATCGCAAAATGCTATAGTGACCTATGATTTTGATCTGAAGTTTGTGCATGTGCATGCTGGTTGGGAG

GGATCGTCTTCATATGCAAGAGTCCTGCAAGATAAATCTTGTGAAATACACTAGGGAAACCCCATGGGAAATAATACTGAGGATTAG
AATCCTGCAAATCAATGGACGTCCATAGAAATGCAATCCTTCAAAAATCCTGTGAAATCCCATGAATCAAAGGAGGCCTTAAAGTTGG
GAAGTTGGAAAACCAGCACTCTTTCGTAAAAGAAACAAGTTTATGGCCAGAATTTTTGTTCCTGGTGCCACGTTAACTTTCTAAACATT
ACATATGGTCAAACCAGCCCTTAAGATGAACCCACACATCAGAAAATAATTGGCAAAAGACAAGTGTCCCCGTGTACTACGTTATAAA
GACAACAACATTTTATGCCCTATGTGCTGTCCATAATGTTATGGCTTGCTCTTAAATCTTTGAACTTGTGAACAAACACAACAATGTAT
TAAGCCGTCCTTAAGGAGTGTCTTTTTTTCTTATGGACAAGTTTTTCAGATTCATAGACGCTTATACAGTTATACTCCCTCTTCCCTGAG
CGTATTCTTGGGTTGTACATTGGTGATAATTTTGAAGTGCACGAATGAAAAATATTAGTTCATCTAATACAAGAGCATATTTTGTTTAT
TTTGTTTTCCTAAATTACTAAATCAGCGACACTTATTATGAATAAGAGGGAGTAGTAACCTAGTAACCTTTGAGTGGTCTCAGTTACTC
ATTAAATTGACGAGACATTCGTTTTGATGTTTGAATAACTCTGTGTACCGAATTGTTTTGAATAGTTCTTGTCCAATAACAATTAACAAT
CTACATATATTTTGTTTACACTAACACTAAAAAAATAATACATCACTTCTAAATTGTTCAGCGCTTTCGGTACTTTGTTTAGTCGTATAT
GTATCTTTTCAATAATCTACTTATCCCCTCATTCTTGGTGTCCCTTGATCTTCCGGAGTGGTTATAGTGGATATATTCTCTCTGGAGATC
CCCTTTTATTAGTGCATATCTCTCCACCTAAGAGATCTTGCGGGCTGGCTGGGAGGGAAGGCATGCTTCCCCATCGCATCTCTTGGTCT
TCCTTCTGGTCTGCTCTGGTGAAGTTCCTACTTTCAATTTCATGCACTGGAGCAAGGCTGGTCTTTGTCACTGGATCTATTCGTTTGCTG
TTCTTTGTTCCTCTCAACCTCCAGATCGGAGGCCCGCCAGGCGGTCTTGGAACAAGACATCAACGGATCGGAGACGGGGACTTTGCT
TTGATCTCGATCTGGTGGTTGCTACCCATTTCAGGGGCTATGATACTTTGGTTGTCGATGATGGAGGTTATTCAAGATATATGGCATC
GTTAACATCCTCTGCCTCCCGAGTTCGTGGAGGCGTTTTGTTCTCGTGGACACCTCGCTTGCTGGTGGCGGCTGGCAGTTCGCTCGTC
TACGACGGCGTGTGACTCAGTCGGCGACGAGAAAGAAGCGGGAGCACGTGTCAACCCGAAGCGTGTATGTGGACGCGTGGATCTT
GCCCGTCTGGGAGTTAGGCAGGTGTCCTTAGACATGGTTGTAAATCTTCTATTTCTTTGTGTCATTTCATGTATCGGGTTGTAATCTGC
AGGTCTTTTGTACCTACTTGGTTTTAATGAAATGCAGGGGTTCTTTCAAAAAAAAAAAAAAACTGAGGTTCACGTTGAAACACACGTT
GCAAATTACGACTCCACGAGGGTTCTCTGCACAAAAGTCGGAATTCGGACCAACGGCGATCCACGAGGTAGCTCGTCGTCTTCCTGG
CAGAAGCTGCCCTTCCTCTGTTCCCGGCTTCTCGCCCCGGCCCCAAGCTCCCCAACAGGCGCAGCCATCTGCCGGAATCCAGGGAACT
TCGTCTCGAACCTTCTCGGAAGGGACCACAACCCCATTCTTCCTCCTCCTCCCCAAAACCCTAGATCCCCAAACCCACCCGGCCACCGC
TTC

Alignment of Sequence_1: [Untitled Sequence #1] with Sequence_2: [Sequence Window #2]

Similarity : 1270/2236 (56.80 %)

```
Seq_1  1      ------------------------------------------------------------  0

Seq_2  1      TACCCTTTTTTTTGGTACGTATACATGCCTGCTCTCTTTTTAGTTTCAGTGTGCTCAGAT  60

Seq_1  1      ------------------------------------------------------------  0

Seq_2  61     AAAAACTTAAGCAAGTTACTGAAATGAATATATGGTGTATGTACTTCCATTTATGTATGA  120

Seq_1  1      ------------------------------------------------------------  0

Seq_2  121    CTGCATCGGAGCTACAGATGGTACACATATTCCATTAAAACTACCATTGCATATGCAAGA  180

Seq_1  1      ------------------------------------------------------------  0

Seq_2  181    AGAATATAAAGCAAACCCTATCGCAAAATGCTATAGTGACCTATGATTTTGATCTGAAGT  240

Seq_1  1      ------------------------------------------------------------  0

Seq_2  241    TTGTGCATGTGCATGCTGGTTGGGAGGGATCGTCTTCATATGCAAGAGTCCTGCAAGATA  300

Seq_1  1      ------------------------------------------------------------  0

Seq_2  301    AATCTTGTGAAATACACTAGGGAAACCCCATGGGAAATAATACTGAGGATTAGAATCCTG  360

Seq_1  1      ------------------------------------------------------------  0

Seq_2  361    CAAATCAATGGACGTCCATAGAAATGCAATCCTTCAAAAATCCTGTGAAATCCCATGAAT  420
```

```
Seq_1  1     ------------------------------------------------------------  0

Seq_2  421   CAAAGGAGGCCTTAAAGTTGGGAAGTTGGAAAACCAGCACTCTTTCGTAAAAGAAACAAG  480

Seq_1  1     ------------------------------------------------------------  0

Seq_2  481   TTTATGGCCAGAATTTTTGTTCCTGGTGCCACGTTAACTTTCTAAACATTACATATGGTC  540

Seq_1  1     ------------------------------------------------------------  0

Seq_2  541   AAACCAGCCCTTAAGATGAACCCACACATCAGAAAATAATTGGCAAAAGACAAGTGTCCC  600

Seq_1  1     ------------------------------------------------------------  0

Seq_2  601   CGTGTACTACGTTATAAAGACAACAACATTTTATGCCCTATGTGCTGTCCATAATGTTAT  660

Seq_1  1     ------------------------------------------------------------  0

Seq_2  661   GGCTTGCTCTTAAATCTTTGAACTTGTGAACAAACACAACAATGTATTAAGCCGTCCTTA  720

Seq_1  1     ------------------------------------------------------------  0

Seq_2  721   AGGAGTGTCTTTTTTTCTTATGGACAAGTTTTTCAGATTCATAGACGCTTATACAGTTAT  780

Seq_1  1     ------------------------------------------------------------  0

Seq_2  781   ACTCCCTCTTCCCTGAGCGTATTCTTGGGTTGTACATTGGTGATAATTTTGAAGTGCACG  840

Seq_1  1     ------------------------------------------------------------  0

Seq_2  841   AATGAAAAATATTAGTTCATCTAATACAAGAGCATATTTTGTTTATTTTGTTTTCCTAAA  900

Seq_1  1     -------ATCAGCGACACTTATTATGAATAAGAGGGAGTAGTAACCTAGTAACCTTTGAG  53
                    |||||||||||||||||||||||||||||||||||||||||||||||||||||
Seq_2  901   TTACTAAATCAGCGACACTTATTATGAATAAGAGGGAGTAGTAACCTAGTAACCTTTGAG  960

Seq_1  54    TGGTCTCAGTTACTCATTAA-TTGACGAGACATTCGTTTTGATGTTTGAATAACTCTGTG  112
             |||||||||||||||||||| |||||||||||||||||||||||||||||||||||||||
Seq_2  961   TGGTCTCAGTTACTCATTAAATTGACGAGACATTCGTTTTGATGTTTGAATAACTCTGTG  1020

Seq_1  113   TAACGAATTGTTTTGAATAGTTCTTGTCCAATAACAAT-------CTACATATATTTTGT  165
             || |||||||||||||||||||||||||||||||||||       |||||||||||||||
Seq_2  1021  TACCGAATTGTTTTGAATAGTTCTTGTCCAATAACAATTAACAATCTACATATATTTTGT  1080

Seq_1  166   TTACACTTACACTAAAAAAATAATACATCACTTCTAAATTGTTCAGCGCTTTCGGTACTT  225
             ||||||| ||||||||||||||||||||||||||||||||||||||||||||||||||||
Seq_2  1081  TTACACTAACACTAAAAAAATAATACATCACTTCTAAATTGTTCAGCGCTTTCGGTACTT  1140

Seq_1  226   TGTTTAGTCGTATATGTATCTTTTCAATAATCTACTTATCACTAAAAAGATATATTATGA  285
             ||||||||||||||||||||||||||||||||||||||||
Seq_2  1141  TGTTTAGTCGTATATGTATCTTTTCAATAATCTACTTATC--------------------  1180

Seq_1  286   CATTTGATTACATATTTTTCCTCCTTTTTTTTACAAAATGCTAATGGTCAAATCATGTAC  345

Seq_2  1181  ------------------------------------------------------------  1180
```

```
Seq_1  346   CATGGACTCTACTTTTAAAAAAAGTCTTGCGTGTCTTAAACTCGGTATGAAAAAATTAAA  405

Seq_2  1181  ------------------------------------------------------------  1180

Seq_1  406   GAGAATCGTCTGCATGTTATTTTGAACTTGAGATCTTAAATTAAAATATATATATCAATA  465

Seq_2  1181  ------------------------------------------------------------  1180

Seq_1  466   CTGCATTATACTCCCTCCGTCCCATAATAAGTGACTCAAATTTGTCCAAATATGAATGTA  525
                         |||||
Seq_2  1181  ------------CCCTC-------------------------------------------  1185

Seq_1  526   TCTATGTCTAAAAAGAGTCTAGATACATGTAATAGAAAGTCACTTAATATGGGACGGAGG  585

Seq_2  1186  ------------------------------------------------------------  1185

Seq_1  586   GAGTAGATATTTACTCTTACAACTCGTTTGATTCCCATCATCGGGGCTTGAAATTCATTT  645

Seq_2  1186  ------------------------------------------------------------  1185

Seq_1  646   CATATTCCAAAAAACAACATGTTTGGTTGTTACAGAATTGAGTGAAATAAAATCAATCTG  705

Seq_2  1186  ------------------------------------------------------------  1185

Seq_1  706   AGATTTCATAAGATCACCAATATCAATCTGAAATGAACAATGGCTAAGCTACCACACGTC  765

Seq_2  1186  ------------------------------------------------------------  1185

Seq_1  766   CACACGGGCTGTTAAATTTTGACTGATGCTTAAACTGAGGTTCATGTAGGGCTCATTTCC  825

Seq_2  1186  ------------------------------------------------------------  1185

Seq_1  826   CCCCTCCTCTATTCCTTCTTCCACATCCTTGCTACAGCTCCGAGCCCTCCCTCGGCCGCT  885

Seq_2  1186  ------------------------------------------------------------  1185

Seq_1  886   TCCTTCCCTCCCCCTTCTCTTCGGCCGGCCAAAGAGAAGGGGGAGAGGAGGTCCGGCCTG  945

Seq_2  1186  ------------------------------------------------------------  1185

Seq_1  946   GTCCTCTCCCATATATGTATATTAAGGTAGTTGCCTTAGAGGCTTGTTCCTGTGGATCTG  1005

Seq_2  1186  ------------------------------------------------------------  1185

Seq_1  1006  CATCGCCGGATTTAAGGCCCCGGTGGTCCCTTCGAAGAGGATTTTTTGCCGTCGCTACCA  1065

Seq_2  1186  ------------------------------------------------------------  1185

Seq_1  1066  ATACTGAGTTTGGTAATGGTAGCCTCGGTTCCATTTCTTCCCTTGATCCCCTCCTAAATA  1125

Seq_2  1186  ------------------------------------------------------------  1185

Seq_1  1126  ATTGCCTCCTTCTAGATCTGGCTCCGTATGGTTTCTTGCTTCTCTTCAATTTCGGAACCA  1185

Seq_2  1186  ------------------------------------------------------------  1185
```

```
Seq_1  1186  GATCTGTGGTTTGTTTCTTTTTTCTCATCAAATCCCCTGATTCTTGGTGTCCCTTGATCT  1245
                                                    |||||||||||||||||||||
Seq_2  1186  ---------------------------------------ATTCTTGGTGTCCCTTGATCT  1206


Seq_1  1246  TCCGGAGTGGTTATAGTGGATATATTCTCTCTGGAGATCCCCTTTTATTAGTGCATATCT  1305
             ||||||||||||||||||||||||||||||||||||||||||||||||||||||||||||
Seq_2  1207  TCCGGAGTGGTTATAGTGGATATATTCTCTCTGGAGATCCCCTTTTATTAGTGCATATCT  1266


Seq_1  1306  CTCCACCTAAGAGATCTTGCGGGCTGGCTGGGAGGGAAGGCATGCTTCCCCATCGCATCT  1365
             ||||||||||||||||||||||||||||||||||||||||||||||||||||||||||||
Seq_2  1267  CTCCACCTAAGAGATCTTGCGGGCTGGCTGGGAGGGAAGGCATGCTTCCCCATCGCATCT  1326


Seq_1  1366  CTTGGTCTTCCTTCTGGTCTGCTCTGGTGAAGTTCCTACTTTCAATTTCATGCACTGGAG  1425
             ||||||||||||||||||||||||||||||||||||||||||||||||||||||||||||
Seq_2  1327  CTTGGTCTTCCTTCTGGTCTGCTCTGGTGAAGTTCCTACTTTCAATTTCATGCACTGGAG  1386


Seq_1  1426  CAAGGCTGGTCTTTGTCACTGGATCTATTCGTTTGCTGTTCTTTGTTCCTCTCAACCTCC  1485
             ||||||||||||||||||||||||||||||||||||||||||||||||||||||||||||
Seq_2  1387  CAAGGCTGGTCTTTGTCACTGGATCTATTCGTTTGCTGTTCTTTGTTCCTCTCAACCTCC  1446


Seq_1  1486  AGATCGGAGGCCTGCCAGGCGGTCTTGGAACAAGACATCAACGGATCGGAGACGGGGACT  1545
             |||||||||||| |||||||||||||||||||||||||||||||||||||||||||||||
Seq_2  1447  AGATCGGAGGCCCGCCAGGCGGTCTTGGAACAAGACATCAACGGATCGGAGACGGGGACT  1506


Seq_1  1546  TTGCTTTGATCTCGATCTGGTGGTTGCTGCCCATTTCAGGGGCTATGATACTTTGGTTGT  1605
             |||||||||||||||||||||||||||| |||||||||||||||||||||||||||||||
Seq_2  1507  TTGCTTTGATCTCGATCTGGTGGTTGCTACCCATTTCAGGGGCTATGATACTTTGGTTGT  1566


Seq_1  1606  CGATGATGGAGGTTATTCAAGATATATGGCATCGTTAACATCCTCTGCCTCCCGAGTTCG  1665
             ||||||||||||||||||||||||||||||||||||||||||||||||||||||||||||
Seq_2  1567  CGATGATGGAGGTTATTCAAGATATATGGCATCGTTAACATCCTCTGCCTCCCGAGTTCG  1626


Seq_1  1666  TGAAGGCATTTTGTTCTCGTGGACACCTCGCTTGCTGGTGGCGGCTGGCAGTTCGCTCCT  1725
             || |||| |||||||||||||||||||||||||||||||||||||||||||||||||| |
Seq_2  1627  TGGAGGCGTTTTGTTCTCGTGGACACCTCGCTTGCTGGTGGCGGCTGGCAGTTCGCTCGT  1686


Seq_1  1726  CTACGACGGCGTGTGACTCAGTCGGCGACGAGGAAGAAGCGGGAGCACGTGTCAACCCGA  1785
             |||||||||||||||||||||||||||||||| |||||||||||||||||||||||||||
Seq_2  1687  CTACGACGGCGTGTGACTCAGTCGGCGACGAGAAAGAAGCGGGAGCACGTGTCAACCCGA  1746


Seq_1  1786  AGCGTGTATGTGGACGCATGGATCTTGCCCGTCTGGGAGTTAGGCAGGTGTCCTTAGACA  1845
             ||||||||||||||||| ||||||||||||||||||||||||||||||||||||||||||
Seq_2  1747  AGCGTGTATGTGGACGCGTGGATCTTGCCCGTCTGGGAGTTAGGCAGGTGTCCTTAGACA  1806


Seq_1  1846  TGGTTGTAAATCTTCTATTTCTTTGTGTCCTTTCATGTATCGGGTTGTAATC--CGTAGG  1903
             ||||||||||||||||||||||||||||| ||||||||||||||||||||||  |  |||
Seq_2  1807  TGGTTGTAAATCTTCTATTTCTTTGTGTCATTTCATGTATCGGGTTGTAATCTGC--AGG  1864


Seq_1  1904  TCTTTTGTACCTACTTGGTTTTAATGAAATGCAGGG-TTCTTT------AAAAAAAAAAC  1956
             |||||||||||||||||||||||||||||||||||| ||||||      |||||||||||
Seq_2  1865  TCTTTTGTACCTACTTGGTTTTAATGAAATGCAGGGGTTCTTTCAAAAAAAAAAAAAAAC  1924


Seq_1  1957  TGAGGTTCACGTTGAAACACACGTTGCAAATTACGACTCCACGAGGGTTCTCTGCACAAA  2016
             ||||||||||||||||||||||||||||||||||||||||||||||||||||||||||||
Seq_2  1925  TGAGGTTCACGTTGAAACACACGTTGCAAATTACGACTCCACGAGGGTTCTCTGCACAAA  1984
```

```
Seq_1  2017  AGTCGGAATTCGGACCAACGGCGATCCACGAGGTAGCTCGTCGTCTTCCTGGCAGAAGCT  2076
             ||||||||||||||||||||||||||||||||||||||||||||||||||||||||||||
Seq_2  1985  AGTCGGAATTCGGACCAACGGCGATCCACGAGGTAGCTCGTCGTCTTCCTGGCAGAAGCT  2044

Seq_1  2077  GCCCTTCCTCTGTTCCCGGCTTCTCGCCCCGGCCCCAAGCTC------------AGCCAT  2124
             ||||||||||||||||||||||||||||||||||||||||||            ||||||
Seq_2  2045  GCCCTTCCTCTGTTCCCGGCTTCTCGCCCCGGCCCCAAGCTCCCCAACAGGCGCAGCCAT  2104

Seq_1  2125  CTGCCGGAATCCAGGGAACTTCGTCTCGAACCTTCTCGGAAGGGACCACAACCCCATTCT  2184
             ||||||||||||||||||||||||||||||||||||||||||||||||||||||||||||
Seq_2  2105  CTGCCGGAATCCAGGGAACTTCGTCTCGAACCTTCTCGGAAGGGACCACAACCCCATTCT  2164

Seq_1  2185  TCCTCCTCCTCCCCAAAACCCTAGATCCCCAAACCCACCCGGCCACCGCTTC  2236
             ||||||||||||||||||||||||||||||||||||||||||||||||||||
Seq_2  2165  TCCTCCTCCTCCCCAAAACCCTAGATCCCCAAACCCACCCGGCCACCGCTTC  2216
```

BdindelWSU_11, DOWNSTREAM

>Bradi3g32820

AGTCTGATCTACCCAAGTAAGACGAGGTTCTTTTCACAGTCATGGTGCAGGACTGGGCTCCCAATTTTGCTATCATGAGCTTCTTAAA
GATGGGCTAGCCGATGGAGTCTCGGAAATACATTTGATCCAGGTGTTCTGACTGTTGTTAACTGCTAACAAACTACTAGCTTATGATT
ATTAGTGATGATTACTGGAAAGTAGATAAGTAGATGGCCCTGGAATTTTGTGAGTGGAAAAAACAATTTTCAAATGTCAAAAGAAAT
AGACATATAAGTTGGAGTTATTTGTGTGGCTTGTAAAATTTTGTGAAAAAAGATGAGGTCTACGTTCTGTAAAATTTTGTGGAAAAAG
ATGAGGCCCACGTCTTCACCAGAATATGCGCAGTGTTCCATCTACATTGTACACCAGACTATAGTATTCCATCTGTTTCTATTCTAAATA
TAAGATGTTCTAGCTTTTCAGCAGCATGTTGTTTGCCATGATGCCATCCAACATCCGAAGTTTGGCTGAAAGGAGAAACTCTTTCCCAC
CCGGGTATGCTTGCAGCGCCTGCGGCACTTCAGTGCAATCATCCTTCTGCTAGCTATCTACAATTCAGCACATGATCGTCCGGAATCCT
GATGGCGGTCAACGTAGAGTTTGGCATGTGAATAGACTGCTGCTTTAAAGTTGCTCATGATTCTGCAATTTGGAGAAGAATAAGTGG
TGATAAATGCCTTTTGATCTACACTGAGGAAATGTAAGACAAAACCATACCTGTTCATTGCCATATTATATACTCCAAGTCACCCTGCT
TGATAACATGTGCAATTTGAGCAGGACACTAAAATTCTCTGAGGTTTTTTATGCAGGCAAACATGCTTCCAAATCATAGTACTTCCTAG
CTGCAGCATCATTGAGAGCACCGGACAGGACAGAAACGACTTGATATAGTAGGCCAATCTGCGAGATGGACATGGAAGAAAAGATT
ACACATGTTTAAAAAGAATCTATACCTATCTAAAAAATACGGACCGTTGGCCTCCCGTAACCAAGCATACGGCCTCCCGTAACCAAGC
ATGTAGCAATATTGGAATGGAGTATGGATCGTACGAAAAAAAGTCATAAAGCAATCTCATGAAAATCAATCCAATCCGAGCCAGAAA
AACTGATACATCCCTCTCTCCCAGCGAAGCTCCACGCCATGGCCCATGGATCGTTAGTCGGGATAGGATTGATCAATGTACTTGCTCG
TAAATTATGCCTACGGCCTACAAGCGTCCCGCCGCCGGCCGCCTCCGCATGCCCGTACCAGCGCCCACGCCCGCGTGCAAGAAAATA
ATCGCTGCTGATTTCCCCCGCTAGGGATGGGCCTCTGCTCGCCGACACCCCCTGCGCGACCTTCGAGATTTCGCTCGGCCGAGCGGCC
GAGGCGCCGACGAAGGGCTGCACAACACCCGTTCCCGTAACCTCTGCTGCATCGCCATCGCCTCCCGCCGCCTCTACCTCCATGCCTC
CCTCTGGTCGGGACTCCTTTCCTGTTGGAAATATGCCCTAGAGGCAATAATAAATATGTTATTATCACATTTTCTTGTTCTTGATAAATG
TTTATTATCCATGCTATAATTGTATTAACAGGAAACTAAATACATGCGTGGATAAATAAACAAATACCGTGTCCCTAATACGCCTCTAC
TAGATTAGCTCGTTTATTAAAAGATGGTTAAGGTTTTCTAACCATAGACATGTGTGGTCATTTAACAACGAGGTCATATCATTAGGAA
AATGATGTGATGGACAGGCCCAAACCTAAGCATAGCTTTTGATCGTGTCACTTAAGTTTAAATTGCTAATGCTTTTATTATGTCAAGTA
TCATTTCCTTAGACCATGAGATCATGCCACTCCCTAGTACCGGAAGAATACTTTGTGGACATCAATCGTCATCTCGTAACTGGGTGATC
ATAAAGATGTTCTTCAGCTCGGAAGGTGCTTGTTGGGTTGTATGGATCAAGAGTAGGATTTGTCACTCCATGTGACGGAGAGATATC
TCTGGGCCCTCTCGGTAATATAACATCCTAATGAGCTTGCAAACATGTGACTAATGTGTTTAGTCACAGGATATTATATTGCGGTACG
AGTAAAGAGAACTTGCCGGTAACGAAATTGAACTAGGTATGTTGATACCGACGATCAAATTTCGGGCAAGTAATATATCGCGAGACA
AAGAAAATTGAATACCGGATTAATTGAATCATCGACATCGTGGTTCAACTGATGAGATTTTCGTGGAATATGTGGGAACCATTATGAA
CATCTAGGTCCCGCTATTGGTTATTGATCGGAGAGGTGTCTCGGTCATGTCCACATGTTCTCGAACCCGTAGGGTCACACACTTAATG
CTTAAATAGATGGTGATATCGAATATTGATTCGAAGTCTCGGATGGGATCCAGGGCATCACGAGGAGCTTCGGAATGGTCCGGAGA
TAAAGAATTATATATGGGAAAGCTGTTTGAGGGTTTCGGAAAAGTTTGGGATATTTTACGGTATCGACCGGGAATGTTCTAGAAGGT
TCCAAAAGTTCTCAAAAGGTTCTAGAATGTTCCAGAATATTCCTAAGAATTTAATTGGGCCTTTATAGTAATTAATTAGAGCAATCTAA

TTATTAGGGGGAAAGGGGAGGAACAAACCGTACGTGGGGGAGTTCGTGGGGAGGGAGTCCTCCCGGGACTCCTTTCCCTCCCCTGG
CCGGCCCTAGCTCTTTCTTGGAGGACGGGCAAACCCTGGCCAGCACCCCCTATATAAATAG

>BdiBd21-3.3G0429200

AGTCTGATCTACCCAAGTAAGACGAGGTTCTTTTCACAGTCATGGTGCAGGACTGGACTCCCAATTTTGCTATCATGAGCTTCTTAAA
GATGGGCTAGCCGATGGAGTCTCGGAAATACATTTGATCCAGGTGTTCTGACTGTTGTTAACTGCTAACAAACTACTAGCTTATGATT
ATTAGTGATGATTACTGGAAAGTAGATAAGTAGATGGCCCTGGAATTTTGTGAGTGGAAAAAACAATTTTCAAATGTCAAAAGAAAT
AGACATATAAGTTGGAGTTATTTGTGTGGCTTGTAAAATTTTGTGAAAACAGATGAGGTCCACGTTCTGTAAAATTTTGTGGAAAAAG
ATGAGGCCCACGTCTTCACCAGAATATGCGCAGTGTTCCATCTACATTGTACACCAGACTATAGTATTCCATCTGTTTCTATTCTAAATA
TAAGATGTTCTAACTTTTCAGCAGCATGTTGTTTGCCATGATGCCATCCAACATCCGAAGTTTGGCTGAAAGGAGAAACTCTTTCCCAC
CCGGGTATGCTTGCAGCGCCTGCGGCACTTCAGTGCAATCATCCTTCTGCTAGCTATCTACAATTCAGCACATGATCGTCCGGAATCCT
GATGGCGGTCAACGTAGAGTTTGGCATGTGAATAGACTGCTGCTTTAAAGTTGCTCATGATTCTGCAATTTGGAGAAGAATAAGTGG
TGATAAATGCCTTTTGATCTACACTGAGGAAATGTAAGACAAAACCATACCTGTTCGTCGCCATATTATATACTCCAAGTCTCCCTGCT
TGATAACATGTGCAATTTGAGCAGGACACTAAAATTCTCTGAGGTTTTTATGCAGGCAAACATGCTTCCAAATCATAGTACTTCCTAG
CTGCAGCATCATTGAGAGCACCGGACAGGACAGAAACGACTTGATATAGTAGGCCAATCTGCCAGATGGACATGGAAGAAAAGATT
ACACATGTCTAAAAAGAATCTATACCTATCTAAAAAAATACGGACCGTTTTCTTCACCGTCGTCTGTCAAACTTTTCATAAAATTTGTGG
GCCCCCTAGGGTTGCATGTATATTAGGCCTACTAAGCCGGCTTGCCCAAATTGGACTGAAACTCCACCAGATCGGTCCAGATTCAATC
AATCACGAATACAACACAACTTCAAACTTAACGTATCAATCACGATCGGTCCAATACGGCCTCCCGTAACCAAGCATGCATCAATATC
GGAATGGAGCATGGATCGTATGAAAAAAAGTCATAAAGCAATCTCATGAAAATCAATCCAATCCGAGCCAGAAAAACTGATACATCC
CTCTCTCCCAGCGAAGCTCCACGCCATGGCCCATGGATCGTTAGTCGAGATAGGATTGATCAATGTACTTGCTCGTAAATTATGCCTA
CGGCCTACAAGCGTCCCGCCGCCGGCTGCCTCCGCATGCCCGTACCAGCGCCCACGCCCGCGTGCAAGAAAATAATCACTGCTGATT
TCCCCCGCTAGGGACGGGCCTCTGCTCGCCGACACCCCCTGCGCGACCTTTGAGATTTCGCTCGGCCGAGCGGCCGAGGCGCCGACG
AAGGCTGCACAACACCCGTTCCGGCGACCTCTGCTGCATCGCCATCGCCTCCCGCCGCCTCTACCTCCATGCCTCCCTCTAGTCGAGAC
TCCTTTCCTATTGGAAATATGCCCTAGAGGCAATAATAAATATGTTATTATCACATTTTCTTGTTCTTGATAAATGTTTATTATACATGCT
ATAATTGTATTAACATGAAACTAAATACATGCGTGGATAAATAAACAAATACCGTGTCCCTAATACGCCTCTACTAGATTAGCTCGTTG
ATTAAAAGATGGTTAAGGTTTTCTAACCATAGACATGTGTGGTCATTTAACAACGAGGTCATATCATTAGGAGAATGATGTGATGGA
CAGGCCCAAACCTAAGCATAGCTTTTGATCGTGTCACTTAAGTTTAAATTGCTAATGCTTTTATTATGTCAAGTATCATTTTCTTAAACC
ATGAGATCATGCCACTCCCTAGTACCGGAAGAATACTTTGTGGACATCAATCGTCATCTCGTAACTGGGTGATCATAAAAGTGTTCTT
CAGCTCGGAAGGTGCTTGTTGGGTTGTATGGATCAAGAGTGGGATTTGTCACTCCATGTGACGGAGAGATATCTCTGGGCCCTCTCG
GTAATATAACATCCTAATGAGCTTGCAAGCATGTGACTAATGTGTTTAGTCACATGATATTATATTGCGGTACGAGTAAAGAGAACTT
GCCGGTAACGAAATTGAACTAGGTATGGTGATACCGACGATCAAATTTCGGGCAAGTAATATATCGCGAGACAAAGAAAATTGAAT
ACCGGATTAATTGAATCATCGACATCGTGGTTCAACCGATGAGATTTTCGTGGAATATGTGGGAACCATTATGGACATCTAGGTCCCG
CTATTGGTTATTGATCGGAGAGGTGTCTCGGTCATGTCCACATGTTCTCGAACCCGTAGGGTCACACACTCAATGCTTAAATAGATGG
TGATATCGAATATTGATTCGAAGTCTTGGATGGGATTCAGGACATCACGAGGAGCTTCGGAATGGTCCGGAGATAAAGAATTATATA
TGGGAAAGCTGTTTGAGGGTTTCGGAAAAGTTTGGGATATTTTACGGTATCGACCGGGAATGTTCTAGAAGGTTCCGTAAGTTCTCA
AAAGGTTCTAGAATGTTCCAGAATATTCCTGAGAATTTAATTGGACCTTTATAGTAATTAATTAGAGCAATCTAATTATTAGGGGGAA
AGGGGAGGAACAAACCGTACATGGGGGAGTTCGTGGGGAGGGAGTCCTCCCGGGACTCCTTTCCCTCCCCTGGCCGGCCCTAGCTC
TTTCTTGCAGGAGGGGCAAACCCTGGCCACC

```
Alignment of Sequence_1:  [Untitled Sequence #1] with  Sequence_2: [Sequence Window #2]

Similarity : 2699/2934 (91.99 %)

Seq_1  1      AGTCTGATCTACCCAAGTAAGACGAGGTTCTTTTCACAGTCATGGTGCAGGACTGGGCTC  60
              ||||||||||||||||||||||||||||||||||||||||||||||||||||||| |||
Seq_2  1      AGTCTGATCTACCCAAGTAAGACGAGGTTCTTTTCACAGTCATGGTGCAGGACTGGACTC  60


Seq_1  61     CCAATTTTGCTATCATGAGCTTCTTAAAGATGGGCTAGCCGATGGAGTCTCGGAAATACA  120
              ||||||||||||||||||||||||||||||||||||||||||||||||||||||||||||
Seq_2  61     CCAATTTTGCTATCATGAGCTTCTTAAAGATGGGCTAGCCGATGGAGTCTCGGAAATACA  120


Seq_1  121    TTTGATCCAGGTGTTCTGACTGTTGTTAACTGCTAACAAACTACTAGCTTATGATTATTA  180
```

```
                ||||||||||||||||||||||||||||||||||||||||||||||||||||||||||||
Seq_2  121   TTTGATCCAGGTGTTCTGACTGTTGTTAACTGCTAACAAACTACTAGCTTATGATTATTA  180

Seq_1  181   GTGATGATTACTGGAAAGTAGATAAGTAGATGGCCCTGGAATTTTGTGAGTGGAAAAAAC  240
             ||||||||||||||||||||||||||||||||||||||||||||||||||||||||||||
Seq_2  181   GTGATGATTACTGGAAAGTAGATAAGTAGATGGCCCTGGAATTTTGTGAGTGGAAAAAAC  240

Seq_1  241   AATTTTCAAATGTCAAAAGAAATAGACATATAAGTTGGAGTTATTTGTGTGGCTTGTAAA  300
             ||||||||||||||||||||||||||||||||||||||||||||||||||||||||||||
Seq_2  241   AATTTTCAAATGTCAAAAGAAATAGACATATAAGTTGGAGTTATTTGTGTGGCTTGTAAA  300

Seq_1  301   ATTTTGTGAAAAAAGATGAGGTCTACGTTCTGTAAAATTTTGTGGAAAAAGATGAGGCCC  360
             |||||||||||| |||||||||| ||||||||||||||||||||||||||||||||||||
Seq_2  301   ATTTTGTGAAAACAGATGAGGTCCACGTTCTGTAAAATTTTGTGGAAAAAGATGAGGCCC  360

Seq_1  361   ACGTCTTCACCAGAATATGCGCAGTGTTCCATCTACATTGTACACCAGACTATAGTATTC  420
             ||||||||||||||||||||||||||||||||||||||||||||||||||||||||||||
Seq_2  361   ACGTCTTCACCAGAATATGCGCAGTGTTCCATCTACATTGTACACCAGACTATAGTATTC  420

Seq_1  421   CATCTGTTTCTATTCTAAATATAAGATGTTCTAGCTTTTCAGCAGCATGTTGTTTGCCAT  480
             ||||||||||||||||||||||||||||||||| ||||||||||||||||||||||||||
Seq_2  421   CATCTGTTTCTATTCTAAATATAAGATGTTCTAACTTTTCAGCAGCATGTTGTTTGCCAT  480

Seq_1  481   GATGCCATCCAACATCCGAAGTTTGGCTGAAAGGAGAAACTCTTTCCCACCCGGGTATGC  540
             ||||||||||||||||||||||||||||||||||||||||||||||||||||||||||||
Seq_2  481   GATGCCATCCAACATCCGAAGTTTGGCTGAAAGGAGAAACTCTTTCCCACCCGGGTATGC  540

Seq_1  541   TTGCAGCGCCTGCGGCACTTCAGTGCAATCATCCTTCTGCTAGCTATCTACAATTCAGCA  600
             ||||||||||||||||||||||||||||||||||||||||||||||||||||||||||||
Seq_2  541   TTGCAGCGCCTGCGGCACTTCAGTGCAATCATCCTTCTGCTAGCTATCTACAATTCAGCA  600

Seq_1  601   CATGATCGTCCGGAATCCTGATGGCGGTCAACGTAGAGTTTGGCATGTGAATAGACTGCT  660
             ||||||||||||||||||||||||||||||||||||||||||||||||||||||||||||
Seq_2  601   CATGATCGTCCGGAATCCTGATGGCGGTCAACGTAGAGTTTGGCATGTGAATAGACTGCT  660

Seq_1  661   GCTTTAAAGTTGCTCATGATTCTGCAATTTGGAGAAGAATAAGTGGTGATAAATGCCTTT  720
             ||||||||||||||||||||||||||||||||||||||||||||||||||||||||||||
Seq_2  661   GCTTTAAAGTTGCTCATGATTCTGCAATTTGGAGAAGAATAAGTGGTGATAAATGCCTTT  720

Seq_1  721   TGATCTACACTGAGGAAATGTAAGACAAAACCATACCTGTTCATTGCCATATTATATACT  780
             |||||||||||||||||||||||||||||||||||||||||| | |||||||||||||||
Seq_2  721   TGATCTACACTGAGGAAATGTAAGACAAAACCATACCTGTTCGTCGCCATATTATATACT  780

Seq_1  781   CCAAGTCACCCTGCTTGATAACATGTGCAATTTGAGCAGGACACTAAAATTCTCTGAGGT  840
             ||||||| ||||||||||||||||||||||||||||||||||||||||||||||||||||
Seq_2  781   CCAAGTCTCCCTGCTTGATAACATGTGCAATTTGAGCAGGACACTAAAATTCTCTGAGGT  840

Seq_1  841   TTTTTATGCAGGCAAACATGCTTCCAAATCATAGTACTTCCTAGCTGCAGCATCATTGAG  900
             ||||||||||||||||||||||||||||||||||||||||||||||||||||||||||||
Seq_2  841   TTTTTATGCAGGCAAACATGCTTCCAAATCATAGTACTTCCTAGCTGCAGCATCATTGAG  900

Seq_1  901   AGCACCGGACAGGACAGAAACGACTTGATATAGTAGGCCAATCTGCGAGATGGACATGGA  960
             |||||||||||||||||||||||||||||||||||||||||||||| |||||||||||||
Seq_2  901   AGCACCGGACAGGACAGAAACGACTTGATATAGTAGGCCAATCTGCCAGATGGACATGGA  960

Seq_1  961   AGAAAAGATTACACATGTTTAAAAAGAATCTATACCTATCTAAAAAATACGGACCGTT--  1018
```

```
                  |||||||||||||||||| |||||||||||||||||||||||||||||||||||||||  
Seq_2   961    AGAAAAGATTACACATGTCTAAAAAGAATCTATACCTATCTAAAAAATACGGACCGTTTT  1020

Seq_1   1019   ------------------------------------------------------------  1018

Seq_2   1021   CTTCACCGTCGTCTGTCAAACTTTTCATAAAATTTGTGGGCCCCCTAGGGTTGCATGTAT  1080

Seq_1   1019   ------------------------------------------------------------  1018

Seq_2   1081   ATTAGGCCTACTAAGCCGGCTTGCCCAAATTGGACTGAAACTCCACCAGATCGGTCCAGA  1140

Seq_1   1019   ------------------------------------------------------------  1018

Seq_2   1141   TTCAATCAATCACGAATACAACACAACTTCAAACTTAACGTATCAATCACGATCGGTCCA  1200

Seq_1   1019   ----GGCCTCCCGTAACCAAGCATACGGCCTCCCGTAACCAAGCATGTAGCAATATTGGA  1074
                   ||||||||||||||||||||                      | | |||||| |||
Seq_2   1201   ATACGGCCTCCCGTAACCAAGCAT----------------------GCATCAATATCGGA  1238

Seq_1   1075   ATGGAGTATGGATCGTACGAAAAAAAGTCATAAAGCAATCTCATGAAAATCAATCCAATC  1134
               |||||| |||||||||| ||||||||||||||||||||||||||||||||||||||||||
Seq_2   1239   ATGGAGCATGGATCGTATGAAAAAAAGTCATAAAGCAATCTCATGAAAATCAATCCAATC  1298

Seq_1   1135   CGAGCCAGAAAAACTGATACATCCCTCTCTCCCAGCGAAGCTCCACGCCATGGCCCATGG  1194
               ||||||||||||||||||||||||||||||||||||||||||||||||||||||||||||
Seq_2   1299   CGAGCCAGAAAAACTGATACATCCCTCTCTCCCAGCGAAGCTCCACGCCATGGCCCATGG  1358

Seq_1   1195   ATCGTTAGTCGGGATAGGATTGATCAATGTACTTGCTCGTAAATTATGCCTACGGCCTAC  1254
               ||||||||||| ||||||||||||||||||||||||||||||||||||||||||||||||
Seq_2   1359   ATCGTTAGTCGAGATAGGATTGATCAATGTACTTGCTCGTAAATTATGCCTACGGCCTAC  1418

Seq_1   1255   AAGCGTCCCGCCGCCGGCCGCCTCCGCATGCCCGTACCAGCGCCCACGCCCGCGTGCAAG  1314
               |||||||||||||||||| |||||||||||||||||||||||||||||||||||||||||
Seq_2   1419   AAGCGTCCCGCCGCCGGCTGCCTCCGCATGCCCGTACCAGCGCCCACGCCCGCGTGCAAG  1478

Seq_1   1315   AAAATAATCGCTGCTGATTTCCCCCGCTAGGGATGGGCCTCTGCTCGCCGACACCCCCTG  1374
               ||||||||| ||||||||||||||||||||||| ||||||||||||||||||||||||||
Seq_2   1479   AAAATAATCACTGCTGATTTCCCCCGCTAGGGACGGGCCTCTGCTCGCCGACACCCCCTG  1538

Seq_1   1375   CGCGACCTTCGAGATTTCGCTCGGCCGAGCGGCCGAGGCGCCGACGAAGGGCTGCACAAC  1434
               ||||||||| |||||||||||||||||||||||||||||||||||||||| |||||||||
Seq_2   1539   CGCGACCTTTGAGATTTCGCTCGGCCGAGCGGCCGAGGCGCCGACGAAGG-CTGCACAAC  1597

Seq_1   1435   ACCCGTTCC--CGTAACCTCTGCTGCATCGCCATCGCCTCCCGCCGCCTCTACCTCCATG  1492
               |||||||||  ||  |||||||||||||||||||||||||||||||||||||||||||||
Seq_2   1598   ACCCGTTCCGGCG--ACCTCTGCTGCATCGCCATCGCCTCCCGCCGCCTCTACCTCCATG  1655

Seq_1   1493   CCTCCCTCTGGTCGGGACTCCTTTCCTGTTGGAAATATGCCCTAGAGGCAATAATAAATA  1552
               ||||||||| |||| |||||||||||| ||||||||||||||||||||||||||||||||
Seq_2   1656   CCTCCCTCTAGTCGAGACTCCTTTCCTATTGGAAATATGCCCTAGAGGCAATAATAAATA  1715

Seq_1   1553   TGTTATTATCACATTTTCTTGTTCTTGATAAATGTTTATTATCCATGCTATAATTGTATT  1612
               |||||||||||||||||||||||||||||||||||||||||| |||||||||||||||||
Seq_2   1716   TGTTATTATCACATTTTCTTGTTCTTGATAAATGTTTATTATACATGCTATAATTGTATT  1775

Seq_1   1613   AACAGGAAACTAAATACATGCGTGGATAAATAAACAAATACCGTGTCCCTAATACGCCTC  1672
```

```
              |||| |||||||||||||||||||||||||||||||||||||||||||||||||||||||
Seq_2  1776   AACATGAAACTAAATACATGCGTGGATAAATAAACAAATACCGTGTCCCTAATACGCCTC  1835

Seq_1  1673   TACTAGATTAGCTCGTTTATTAAAAGATGGTTAAGGTTTTCTAACCATAGACATGTGTGG  1732
              ||||||||||||||||| ||||||||||||||||||||||||||||||||||||||||||
Seq_2  1836   TACTAGATTAGCTCGTTGATTAAAAGATGGTTAAGGTTTTCTAACCATAGACATGTGTGG  1895

Seq_1  1733   TCATTTAACAACGAGGTCATATCATTAGGAAAATGATGTGATGGACAGGCCCAAACCTAA  1792
              |||||||||||||||||||||||||||||| |||||||||||||||||||||||||||||
Seq_2  1896   TCATTTAACAACGAGGTCATATCATTAGGAGAATGATGTGATGGACAGGCCCAAACCTAA  1955

Seq_1  1793   GCATAGCTTTTGATCGTGTCACTTAAGTTTAAATTGCTAATGCTTTTATTATGTCAAGTA  1852
              ||||||||||||||||||||||||||||||||||||||||||||||||||||||||||||
Seq_2  1956   GCATAGCTTTTGATCGTGTCACTTAAGTTTAAATTGCTAATGCTTTTATTATGTCAAGTA  2015

Seq_1  1853   TCATTTCCTTAGACCATGAGATCATGCCACTCCCTAGTACCGGAAGAATACTTTGTGGAC  1912
              |||||| |||| ||||||||||||||||||||||||||||||||||||||||||||||||
Seq_2  2016   TCATTTTCTTAAACCATGAGATCATGCCACTCCCTAGTACCGGAAGAATACTTTGTGGAC  2075

Seq_1  1913   ATCAATCGTCATCTCGTAACTGGGTGATCATAAAGATGTTCTTCAGCTCGGAAGGTGCTT  1972
              ||||||||||||||||||||||||||||||||||  ||||||||||||||||||||||||
Seq_2  2076   ATCAATCGTCATCTCGTAACTGGGTGATCATAAAAGTGTTCTTCAGCTCGGAAGGTGCTT  2135

Seq_1  1973   GTTGGGTTGTATGGATCAAGAGTAGGATTTGTCACTCCATGTGACGGAGAGATATCTCTG  2032
              ||||||||||||||||||||||| ||||||||||||||||||||||||||||||||||||
Seq_2  2136   GTTGGGTTGTATGGATCAAGAGTGGGATTTGTCACTCCATGTGACGGAGAGATATCTCTG  2195

Seq_1  2033   GGCCCTCTCGGTAATATAACATCCTAATGAGCTTGCAAACATGTGACTAATGTGTTTAGT  2092
              |||||||||||||||||||||||||||||||||||||| |||||||||||||||||||||
Seq_2  2196   GGCCCTCTCGGTAATATAACATCCTAATGAGCTTGCAAGCATGTGACTAATGTGTTTAGT  2255

Seq_1  2093   CACAGGATATTATATTGCGGTACGAGTAAAGAGAACTTGCCGGTAACGAAATTGAACTAG  2152
              |||| |||||||||||||||||||||||||||||||||||||||||||||||||||||||
Seq_2  2256   CACATGATATTATATTGCGGTACGAGTAAAGAGAACTTGCCGGTAACGAAATTGAACTAG  2315

Seq_1  2153   GTATGTTGATACCGACGATCAAATTTCGGGCAAGTAATATATCGCGAGACAAAGAAAATT  2212
              ||||| ||||||||||||||||||||||||||||||||||||||||||||||||||||||
Seq_2  2316   GTATGGTGATACCGACGATCAAATTTCGGGCAAGTAATATATCGCGAGACAAAGAAAATT  2375

Seq_1  2213   GAATACCGGATTAATTGAATCATCGACATCGTGGTTCAACTGATGAGATTTTCGTGGAAT  2272
              |||||||||||||||||||||||||||||||||||||||| |||||||||||||||||||
Seq_2  2376   GAATACCGGATTAATTGAATCATCGACATCGTGGTTCAACCGATGAGATTTTCGTGGAAT  2435

Seq_1  2273   ATGTGGGAACCATTATGAACATCTAGGTCCCGCTATTGGTTATTGATCGGAGAGGTGTCT  2332
              ||||||||||||||||| ||||||||||||||||||||||||||||||||||||||||||
Seq_2  2436   ATGTGGGAACCATTATGGACATCTAGGTCCCGCTATTGGTTATTGATCGGAGAGGTGTCT  2495

Seq_1  2333   CGGTCATGTCCACATGTTCTCGAACCCGTAGGGTCACACACTTAATGCTTAAATAGATGG  2392
              |||||||||||||||||||||||||||||||||||||||||| |||||||||||||||||
Seq_2  2496   CGGTCATGTCCACATGTTCTCGAACCCGTAGGGTCACACACTCAATGCTTAAATAGATGG  2555

Seq_1  2393   TGATATCGAATATTGATTCGAAGTCTCGGATGGGATCCAGGGCATCACGAGGAGCTTCGG  2452
              |||||||||||||||||||||||||| ||||||||| |||| ||||||||||||||||||
Seq_2  2556   TGATATCGAATATTGATTCGAAGTCTTGGATGGGATTCAGGACATCACGAGGAGCTTCGG  2615

Seq_1  2453   AATGGTCCGGAGATAAAGAATTATATATGGGAAAGCTGTTTGAGGGTTTCGGAAAAGTTT  2512
```

```
                ||||||||||||||||||||||||||||||||||||||||||||||||||||||||||||
Seq_2  2616  AATGGTCCGGAGATAAAGAATTATATATGGGAAAGCTGTTTGAGGGTTTCGGAAAAGTTT  2675


Seq_1  2513  GGGATATTTTACGGTATCGACCGGGAATGTTCTAGAAGGTTCCAAAAGTTCTCAAAAGGT  2572
             |||||||||||||||||||||||||||||||||||||||||||  |||||||||||||||
Seq_2  2676  GGGATATTTTACGGTATCGACCGGGAATGTTCTAGAAGGTTCCGTAAGTTCTCAAAAGGT  2735


Seq_1  2573  TCTAGAATGTTCCAGAATATTCCTAAGAATTTAATTGGGCCTTTATAGTAATTAATTAGA  2632
             |||||||||||||||||||||||| ||||||||||||| |||||||||||||||||||||
Seq_2  2736  TCTAGAATGTTCCAGAATATTCCTGAGAATTTAATTGGACCTTTATAGTAATTAATTAGA  2795


Seq_1  2633  GCAATCTAATTATTAGGGGGAAAGGGGAGGAACAAACCGTACGTGGGGGAGTTCGTGGGG  2692
             |||||||||||||||||||||||||||||||||||||||||| |||||||||||||||||
Seq_2  2796  GCAATCTAATTATTAGGGGGAAAGGGGAGGAACAAACCGTACATGGGGGAGTTCGTGGGG  2855


Seq_1  2693  AGGGAGTCCTCCCGGGACTCCTTTCCCTCCCCTGGCCGGCCCTAGCTCTTTCTTGGAGGA  2752
             ||||||||||||||||||||||||||||||||||||||||||||||||||||||| ||||
Seq_2  2856  AGGGAGTCCTCCCGGGACTCCTTTCCCTCCCCTGGCCGGCCCTAGCTCTTTCTTGCAGGA  2915


Seq_1  2753  CGGGCAAACCCTGGCCAGCACCCCCTATATAAATAG  2788
              ||||||||||||||||   ||
Seq_2  2916  GGGGCAAACCCTGGCCA---CC--------------  2934
```

BdindelWSU_12, DOWNSTREAM

>Bradi3g34310

GCATTGTGGTGTGACTTGTGCTTCTCTTTCATGCGTGGTAGTGGCACGGTGAGAATACTCGAGGAAATTTTAGATGGCTCAGATGTAT
GTAACCTATCTGCTCCCTCCGTTCCATAATTTTTGTCTCAAATTCAACCAAAAATGGATGTATCTAATCTTACAAAGTGTCTAGATACAT
GTAAGATTCCGACAAGAATTATGGAACGAAGGGAGTACTACATATGCAAGTATAAATCAATGCAGTTGATAATCACAAGTCTTCAGG
AGTTCTTATTTACAGGGCTTACAGTCACGCTGACATTCAAATTTGCATTGAATTTATCTTTATGTTGAGTTTATAATGTGGTCGTGGGA
TCGAATTCTGCGCTTCGCACTGTAATGATGGTTGATCAATTTCTTTTGGGTGCGCTCGCTTGAGAATGGATAGATAGATGGGTTTATC
GGACGCAAATCGTCATGTGTGAAGTAATTTCGATGATTATGCGGTGCGTTGCGGCGATACAGCACTTGAACTGCACCTAGCGACCGT
GCTATACCGGTGCATGGCCAATGTTGGTCAACGCCAACCTTGGAGCACCATGGTGCTAAGAAAATTTCCTTGGAACGGAATCAAGTT
GTTGCTCCGGTTCTAAGAAGGTCGTAGACAATTTGGTGGACCCCAGCTGCAGCCACGCGCCTCGTCAGTCGTCGCCCACGGTGCGTC
TCGTTGCGATCAGATCTGAAGGAGGCCGAGGCGGGAGGAGGAGGGCCTCACCTCCAGACGTCGCGAATCTGGGAGGAGGCCGACT
CGGGGAGGGGAGGGGGCGGACACGGCGCCGTCGGGAGGAGGAGGCCTACTGGAGGGAGAGAGGCCGCGCTGCCGGTAGCGGCC
GGGAGGAGGAGGCAGACGATGAGGATGGGAATCCAGCGTGGTGGCTGTAGGGATGACGAGGAGGCCAATCAGGATGCCGCTGGC
ATAATCCGGCGGTGGAGATGAGGGGAACTCAAGAAAATCTGGCAGTGGACGATCGGCGGAGGAAACGAAGCAGGGGAGGAAGTA
GCAAGCAACAGAGAAGAAAAGCAGGAGGCGGCCAGGACTGGGGAAAGTGACGTCCGGTGCTTTTTCTTCTTCTTTCTTTATTAGGA
GAGGTCCGGTGCTAAGAAAACTTCACGTTGCAGCAGTTTTGTGGTTTTGAGGCCCGGTACTTGTGTCCGCCCACTAGAACTGATACTT
CACCTTACATTGCTCTTGCTCCTCTGTTGAACTTGTGTAGCTAAGGATTTCATCGTTTTAGTCTGACATCTCTACTTTGCTGTTAGTTCG
GTAAACTGTTTGCTTACCGCTGAAGTGGTTAGCGAGGCGTCAATGGTCTTCTGCAGATGTTGTGTACAGTGCAATTGATGTTTCTGAA
AGAAAAAAATTGCGTAGGTCTTGTTCGGTTGTTAAATTCCTTGCAAGTATCAAGTATGGAAGTTAGAAGATTGAAGAAAATTTATAGTC
CCATTGTTCCCTCGATCACTACCGGGGTGAGATTAAAACTTGGCCTTAAAAGAAGATAATAAACCACGGTTTGATATCTGCTGAAGTC
CAAGTGAATCTTTTCAGTGTCAGGTCGGACAGTCCATTTTTATATGGACTTGGACTACATGGATCGAGTTACCCCAATGCCATCACTG
GACTGAAACGACAAACTACACAGCTCATCACTCACTGGACCATTTGTCTGCCGCAGCCCGGACTAGCTAGCACGTTGCGCGAGGCAC
GTACGTACGTACGTGTCCTGCATGCACCTTTCCCAACTTAAATTAACCATGCGCGATCGCCGATCATCAGCCCGGCCGTACATGATAA
TGGCGCTACTGGCCCATCAACCAGGACTAGGAGGAAGAGGGAGAGAGACCAGCTGTGTCGGACTCTCGATGGATCGAACAATATGC
ATGGGTCCATGGGTCCCTGGATAAGCTCGTTTTGGATCTACGATAACTGTCATTCATGGGCCATGCCCAGGAGGCGTGCACCATGCC
ACCATGGAGGACGCGGCCGGGTCGGATAGCTAAGCAGCCAAGCTTGGTTGGACATATGCGCGCCGAAATCACAACCATTCTGGGCC
GTCGGATATTCTTTCATTTCCTGTCTTTAATCACAAATCTGAGACAATTTCTCCCGAATTTGAGAGAGCGACAATGATGAACCCGACTT
GCAGACGCAGGGTCCCGATCGAGACGCGCGGTAACCCATGTCCCGCCACCGGAAAACCAGCAGCGCTCGCCGTGGTCTCGCGTCGT

GGGTCGTGGCCGCCCACCCGCGCGCGGCGCCGCCGGCCGCAAACACGTGTCCCCCCCAGTCCCCTACACTCACGTGCTCTCCTCTTTC
GATCTCTTCCCTCTCCTTCACGGACGGGACGGGCACAGTGGACGCGTGGAATATAACTCGAGTGAGTGACGCCGTCAATCTCGCCGT
CGTCTTCTGCCTCTCGTACGTCACGCCTCGCTGTATATAAAAGAGAGCGGGCACCCATCCTCCGAGTACCCCAGGACACCGCACGCGC
AGGCAGGCAGCAATGGAAGGCGCCACATCGTCCCTCACGCGCTCCCTCTCCCTCGCCCCCCGCCCACGCCGCCGCTCTCACCACAACC
TGCCCACCCTCTCCCCCGCGCC

>BdiBd21-3.3G0450100

GCATTGTGGTGTGACTTGTGCTTCTCTTTCATGCGTGGTAGTGGCACGGTGAGAATACACGAGGAAATTTTAGATGGTTCAGATGTAT
GTAACCTATCTGCTCCCTCCGTTCCATAATTTTTGTCTCAAATTCAACCAAAAATGGATGTATCTAATCCTACAAAGTGTCTAGATACAT
GTAAGATTCCGACAAGAATTATGGAATGAAGGGAGTACTACATATGCAAGTATAAATCAATGCAGTTGATAATCAAGTCTTCAGGAG
TTCTTATTTACAGGGCTTACAGTCACGCTGACATTCAAATTTGCATCGAATTTATCTTTATGTTGAGTTTATAATGTGGTCGTGGGATC
GAATTCTGCGCTTCGCACTGTAATGATGGTTGATCAATTTCTTTTGGGTGCGCTCGCTTGAGAATGGATAGATAGATGGGTTTATCGG
ACGCAAATCGTCATGTGTGAAGTAATTTCGATGATTATGCGGTGCGTTGCGGCGATACAGCACTTGAACTGCACCTAGCGACCGTGC
TATACCGGTGCCTGGCCAATGTTGGTCAACACCAACCTTGGAGCACCATGGTGCTAAGAAAATTTCCTTGGAACGGAATCAAGTAGT
TGCTCCGGTTCTAAGAAGGTCGTAGACAATTTGGTGGACCCCAGCTGCAGCCACGCGCCTCGTCAGTCGTCGCCCACGGTGCGTCTC
GTTGCGATCAGATCTGAAGGAGGCCGAGGCGGGAGGAGGAGGGCCTCACCTCCAGACGTCGCGAATCTGGGAGGAGGCCGACTCG
GGGAGGGGAGGGGGCGGACACGGCGCCGTCGGGAGGAGGAGGCCTACTGGAGGGAGAGAGGCCGCGCTGCCGGTAGCGGCCG
GGAGGAGGAGGCAGACGATGAGGATGGGAATCCAGCGTGGTGGCTGTAGGGAAGACGAGGAGGCCAATCAGGATGCCGCTGGCA
TAATCCGGCGGTGGAGATGAGGGGAACTCAAGAAAATCCGGCAGTGGACGATCGGCGGAGGAAACGAAGCAGGGGAGGAAGTAG
GCAAGCAACAGAGAAGAAAAGCAGGAGGCGGCCAGGACTGGGGAAAGTGACGTCCGGTGCTTTTTTTTCTTCTTTCTTTATTAGGAG
AGTTCCGGTGCTAAGAAAACTTCACGTTGCAGCAGTTTTGTGGTTTTGAGGCCCGGTACTTGTGTCCGCCCACTAGAACTGATACTTC
ACCTTACATTGCTCTTGCTCCTCTGTTGAACTTCTGTAGCTAAGGATTTCATCGTTTTAGTCTGATATCTCTACTTTGCTGTTAGTTCGGT
AAACTGTTTGCTTACCGCTGAAGTGGTTAGCGAGGCGTCAATGGTCTTCTGCAGATGTTGTGTACAGTGCAATTGATGTTTCTGAAAG
AAAAAATTGCGTAGGTCTTGTTCGGTTGTAAAATTCCTTGCAAGTATCAAGTATGGAAGTTAGAAGATTCAAGAAAATTTATACAGTC
CCGTTGTTCCTTCGATCACTACCGGGGTGAGATTAAAACTTGGCCGGTGCCTGTGTTCTATAAAACAGGTGCTTAATTAGACACTTCTA
GTATAAAAATAAGCACTGATGTTCGAGAATAAATCGGTTTAATTTTATAACATTGTGTTTAACGGTTTGCATTGGACATTCTCTAAGAA
GATAATAAACCACGGTTTGATATCTGCTGAAGTCCAAGTGAATCTTTTCAGTGTCAGGTCGGACCGTCCATTTTTATATGGACTTGGA
CTACATGGATCGAGTTACCCCAATGCCATCACTGGACTGAAACGACAAACTACACAGCTCATCACTCACTGGCGAATCGCATCCAAAA
GTCCAGATAAGAAGATGATGGGCACGGCCACTGGCCTGGACTGGACCATTTGTCTGCCGCAGCCCGGACTAGCTAGCACGTTGCGC
GAGGCACGTACGTACGTGTCCTGCATGCACCTTTCTCAACTTAAATTAACCATGCGCGATCGCCGATCATCAGCCCGGCCGTACATGA
TAATGGCGCTACTAGCCCATCAACCAGGACTAGGAGGAAGAGGGAGAGAGACCAGCTGTGTCGGACTCTCGATGGATCGAACAATA
TGCATGGGTCCATGGGTCCCTGGATAAGCTCGTTTTGGATCTACGATAACTGTGATTCATGGGCCATGCCCAGGAGGCGTGCACCAT
GCCACCATGGAGGACGCGGCCGGGTCGGATAGCTAAGCAGCCAAGCTTGGTTGGACATATGCGCGCCGAAATCACAACCATTCTGG
GCCGTCGGATATTCTTTCATTTCCTGTCTTTAATCACAAATCTGAGACAATTTCTCCCGAATTTGAGAGAGCGACAATGATGAACCCGA
CTTGCAGACGCAGGGTCCCGATCGAGGCGCGCGGTAAACCATGTCCCGCCACCGGAAAACCAGCAGCGCTCGCCGTGGTCTCGCGT
CGTGGGTCGTGGCCGCCCACCCGCGCGCGGCGCCGCCGGCCGCAAACACGTGTCCCCCCCAGTCCCCTACACTCACGTGCTCTTCCCT
CTCCTTCACGGACGGGACGGGCACAGTGGACGCGTGGAATATAACTCGAGTGAGTGACGCCGTCAATCTCGCCGTCGTCTTCTGCCT
CTCGTACGTCACGCCTCGCTGTATATAAAAGAGAGCGGGCACCCATCCTCAGAGTAGCCCAGGACGCCGCACGCGCAGGCAGGCAG
CAATGGAAGGCGCCACATCGTCCCTCACGCGCTCCCTCTCCCTCGCCCCCCGCCCACGCCGCCGCTCTCACCACAACCTGCCCACCCTC
TCCCCCGCGCCCCGCGTGCTCCCGCTCCGCCGCGCCAGATCTGACGCCCACGACCTCCTCGGATCCATGGCCACCGCCGCCGCAGCG
GCAACATCCTCCGCCGCCACCGTCCTCCCCCGCCCACGGACCCTCGACAGGGACAACGACTGCCTCTTCGCCGGCCCGGGCAACGGC
AACGGCAACAACGACAACAGATCCGGCAACGGCGGCGGCGGGGGCGGCAACGGCGGCGGGAGCGGCCAGAGCGCCGGCATGGG
GGAGCACTACCGGCGCGCGCTGAGCCTGGACCCGTCCAACCCGCTGCTGCTGCGCAACTACGGCAAGTTCCTCCACGACGTGCAGCG
CGACCTGCCCGGTGCCCAGGACTGCTACGCGCGCGCCATGCTCGCCTCCCCCGCCGACGCCGACCTGCTCAGCCTCTACGGCCGCGC
GCTCTGGGAGGCCGGCCAGGGCCACGGCCAGGCGGACAGGGACGGCAGCAAGGACCGCGCCGAGGGATACTTCCAGCGCGCCGT
CCAGGCCGCGCCCGACGACTGCCACGTGCTCGCCTCCTACGCCAGCTTCCTCTGGGACGCCGAGGAAGACGACGTGGAGGACCAGG
TCGCCTGTGGCTCTCCGGCGTCCTTTGTGCCGGCGTGCTGACACAACCTTGATCTCGGCCATCTCATCTGCGGTCTGCTGTACTGTAAC
ATAGGAGGCGCGCCGGCTGGAAGCAGGGTGATGGAGA

Alignment of Sequence_1: [Untitled Sequence #1] with Sequence_2: [Sequence Window #2]

```
Similarity : 2583/3519 (73.40 %)

Seq_1  1     GCATTGTGGTGTGACTTGTGCTTCTCTTTCATGCGTGGTAGTGGCACGGTGAGAATACTC  60
             |||||||||||||||||||||||||||||||||||||||||||||||||||||||||| |
Seq_2  1     GCATTGTGGTGTGACTTGTGCTTCTCTTTCATGCGTGGTAGTGGCACGGTGAGAATACAC  60


Seq_1  61    GAGGAAATTTTAGATGGCTCAGATGTATGTAACCTATCTGCTCCCTCCGTTCCATAATTT  120
             ||||||||||||||||| ||||||||||||||||||||||||||||||||||||||||||
Seq_2  61    GAGGAAATTTTAGATGGTTCAGATGTATGTAACCTATCTGCTCCCTCCGTTCCATAATTT  120


Seq_1  121   TTGTCTCAAATTCAACCAAAAATGGATGTATCTAATCTTACAAAGTGTCTAGATACATGT  180
             ||||||||||||||||||||||||||||||||||||| ||||||||||||||||||||||
Seq_2  121   TTGTCTCAAATTCAACCAAAAATGGATGTATCTAATCCTACAAAGTGTCTAGATACATGT  180


Seq_1  181   AAGATTCCGACAAGAATTATGGAACGAAGGGAGTACTACATATGCAAGTATAAATCAATG  240
             |||||||||||||||||||||||| |||||||||||||||||||||||||||||||||||
Seq_2  181   AAGATTCCGACAAGAATTATGGAATGAAGGGAGTACTACATATGCAAGTATAAATCAATG  240


Seq_1  241   CAGTTGATAATCACAAGTCTTCAGGAGTTCTTATTTACAGGGCTTACAGTCACGCTGACA  300
             |||||||||||||  |||||||||||||||||||||||||||||||||||||||||||||
Seq_2  241   CAGTTGATAATCA--AGTCTTCAGGAGTTCTTATTTACAGGGCTTACAGTCACGCTGACA  298


Seq_1  301   TTCAAATTTGCATTGAATTTATCTTTATGTTGAGTTTATAATGTGGTCGTGGGATCGAAT  360
             ||||||||||||| ||||||||||||||||||||||||||||||||||||||||||||||
Seq_2  299   TTCAAATTTGCATCGAATTTATCTTTATGTTGAGTTTATAATGTGGTCGTGGGATCGAAT  358


Seq_1  361   TCTGCGCTTCGCACTGTAATGATGGTTGATCAATTTCTTTTGGGTGCGCTCGCTTGAGAA  420
             ||||||||||||||||||||||||||||||||||||||||||||||||||||||||||||
Seq_2  359   TCTGCGCTTCGCACTGTAATGATGGTTGATCAATTTCTTTTGGGTGCGCTCGCTTGAGAA  418


Seq_1  421   TGGATAGATAGATGGGTTTATCGGACGCAAATCGTCATGTGTGAAGTAATTTCGATGATT  480
             ||||||||||||||||||||||||||||||||||||||||||||||||||||||||||||
Seq_2  419   TGGATAGATAGATGGGTTTATCGGACGCAAATCGTCATGTGTGAAGTAATTTCGATGATT  478


Seq_1  481   ATGCGGTGCGTTGCGGCGATACAGCACTTGAACTGCACCTAGCGACCGTGCTATACCGGT  540
             ||||||||||||||||||||||||||||||||||||||||||||||||||||||||||||
Seq_2  479   ATGCGGTGCGTTGCGGCGATACAGCACTTGAACTGCACCTAGCGACCGTGCTATACCGGT  538


Seq_1  541   GCATGGCCAATGTTGGTCAACGCCAACCTTGGAGCACCATGGTGCTAAGAAAATTTCCTT  600
             || |||||||||||||||||| ||||||||||||||||||||||||||||||||||||||
Seq_2  539   GCCTGGCCAATGTTGGTCAACACCAACCTTGGAGCACCATGGTGCTAAGAAAATTTCCTT  598


Seq_1  601   GGAACGGAATCAAGTTGTTGCTCCGGTTCTAAGAAGGTCGTAGACAATTTGGTGGACCCC  660
             ||||||||||||||| ||||||||||||||||||||||||||||||||||||||||||||
Seq_2  599   GGAACGGAATCAAGTAGTTGCTCCGGTTCTAAGAAGGTCGTAGACAATTTGGTGGACCCC  658


Seq_1  661   AGCTGCAGCCACGCGCCTCGTCAGTCGTCGCCCACGGTGCGTCTCGTTGCGATCAGATCT  720
             ||||||||||||||||||||||||||||||||||||||||||||||||||||||||||||
Seq_2  659   AGCTGCAGCCACGCGCCTCGTCAGTCGTCGCCCACGGTGCGTCTCGTTGCGATCAGATCT  718


Seq_1  721   GAAGGAGGCCGAGGCGGGAGGAGGAGGGCCTCACCTCCAGACGTCGCGAATCTGGGAGGA  780
             ||||||||||||||||||||||||||||||||||||||||||||||||||||||||||||
Seq_2  719   GAAGGAGGCCGAGGCGGGAGGAGGAGGGCCTCACCTCCAGACGTCGCGAATCTGGGAGGA  778


Seq_1  781   GGCCGACTCGGGGAGGGGAGGGGGCGGACACGGCGCCGTCGGGAGGAGGAGGCCTACTGG  840
             ||||||||||||||||||||||||||||||||||||||||||||||||||||||||||||
```

```
Seq_2  779   GGCCGACTCGGGGAGGGGAGGGGGCGGACACGGCGCCGTCGGGAGGAGGAGGCCTACTGG  838


Seq_1  841   AGGGAGAGAGGCCGCGCTGCCGGTAGCGGCCGGGAGGAGGAGGCAGACGATGAGGATGGG  900
             ||||||||||||||||||||||||||||||||||||||||||||||||||||||||||||
Seq_2  839   AGGGAGAGAGGCCGCGCTGCCGGTAGCGGCCGGGAGGAGGAGGCAGACGATGAGGATGGG  898


Seq_1  901   AATCCAGCGTGGTGGCTGTAGGGATGACGAGGAGGCCAATCAGGATGCCGCTGGCATAAT  960
             |||||||||||||||||||||||| |||||||||||||||||||||||||||||||||||
Seq_2  899   AATCCAGCGTGGTGGCTGTAGGGAAGACGAGGAGGCCAATCAGGATGCCGCTGGCATAAT  958


Seq_1  961   CCGGCGGTGGAGATGAGGGGAACTCAAGAAAATCTGGCAGTGGACGATCGGCGGAGGAAA  1020
             |||||||||||||||||||||||||||||||||| |||||||||||||||||||||||||
Seq_2  959   CCGGCGGTGGAGATGAGGGGAACTCAAGAAAATCCGGCAGTGGACGATCGGCGGAGGAAA  1018


Seq_1  1021  CGAAGCAGGGGAGGAAGTAG-CAAGCAACAGAGAAGAAAAGCAGGAGGCGGCCAGGACTG  1079
             |||||||||||||||||||| |||||||||||||||||||||||||||||||||||||||
Seq_2  1019  CGAAGCAGGGGAGGAAGTAGGCAAGCAACAGAGAAGAAAAGCAGGAGGCGGCCAGGACTG  1078


Seq_1  1080  GGGAAAGTGACGTCCGGTGCTTTTTCTTCTTCTTTCTTTATTAGGAGAGGTCCGGTGCTA  1139
             ||||||||||||||||||||||||| ||||||||||||||||||||||| ||||||||||
Seq_2  1079  GGGAAAGTGACGTCCGGTGCTTTTTTTTCTTCTTTCTTTATTAGGAGAGTTCCGGTGCTA  1138


Seq_1  1140  AGAAAACTTCACGTTGCAGCAGTTTTGTGGTTTTGAGGCCCGGTACTTGTGTCCGCCCAC  1199
             ||||||||||||||||||||||||||||||||||||||||||||||||||||||||||||
Seq_2  1139  AGAAAACTTCACGTTGCAGCAGTTTTGTGGTTTTGAGGCCCGGTACTTGTGTCCGCCCAC  1198


Seq_1  1200  TAGAACTGATACTTCACCTTACATTGCTCTTGCTCCTCTGTTGAACTTGTGTAGCTAAGG  1259
             |||||||||||||||||||||||||||||||||||||||||||||||| |||||||||||
Seq_2  1199  TAGAACTGATACTTCACCTTACATTGCTCTTGCTCCTCTGTTGAACTTCTGTAGCTAAGG  1258


Seq_1  1260  ATTTCATCGTTTTAGTCTGACATCTCTACTTTGCTGTTAGTTCGGTAAACTGTTTGCTTA  1319
             |||||||||||||||||||| |||||||||||||||||||||||||||||||||||||||
Seq_2  1259  ATTTCATCGTTTTAGTCTGATATCTCTACTTTGCTGTTAGTTCGGTAAACTGTTTGCTTA  1318


Seq_1  1320  CCGCTGAAGTGGTTAGCGAGGCGTCAATGGTCTTCTGCAGATGTTGTGTACAGTGCAATT  1379
             ||||||||||||||||||||||||||||||||||||||||||||||||||||||||||||
Seq_2  1319  CCGCTGAAGTGGTTAGCGAGGCGTCAATGGTCTTCTGCAGATGTTGTGTACAGTGCAATT  1378


Seq_1  1380  GATGTTTCTGAAAGAAAAAATTGCGTAGGTCTTGTTCGGTTGTTAAATTCCTTGCAAGTA  1439
             ||||||||||||||||||||||||||||||||||||||||||| ||||||||||||||||
Seq_2  1379  GATGTTTCTGAAAGAAAAAATTGCGTAGGTCTTGTTCGGTTGTAAAATTCCTTGCAAGTA  1438


Seq_1  1440  TCAAGTATGGAAGTTAGAAGATT--GAAGAAAATTTATAGTCCCATTGTTCCCTCGATCA  1497
             |||||||||||||||||||||||    | ||  | || |||||| ||||||| |||||||
Seq_2  1439  TCAAGTATGGAAGTTAGAAGATTCAAGAAAATTTATACAGTCCCGTTGTTCCTTCGATCA  1498


Seq_1  1498  CTACCGGGGTGAGATTAAAACTTGGCC---------------------------TTAA--  1528
             |||||||||||||||||||||||||||                           ||||
Seq_2  1499  CTACCGGGGTGAGATTAAAACTTGGCCGGTGCCTGTGTTCTATAAAACAGGTGCTTAATT  1558


Seq_1  1529  ------------------------------------------------------------  1528

Seq_2  1559  AGACACTTCTAGTATAAAAATAAGCACTGATGTTCGAGAATAAATCGGTTTAATTTTATA  1618


Seq_1  1529  -----------------------------------AAGAAGATAATAAACCACGGTTTGA  1553
                                                |||||||||||||||||||||||||
```

```
Seq_2  1619   ACATTGTGTTTAACGGTTTGCATTGGACATTCTCTAAGAAGATAATAAACCACGGTTTGA  1678


Seq_1  1554   TATCTGCTGAAGTCCAAGTGAATCTTTTCAGTGTCAGGTCGGACAGTCCATTTTTATATG  1613
              |||||||||||||||||||||||||||||||||||||||||||| |||||||||||||||
Seq_2  1679   TATCTGCTGAAGTCCAAGTGAATCTTTTCAGTGTCAGGTCGGACCGTCCATTTTTATATG  1738


Seq_1  1614   GACTTGGACTACATGGATCGAGTTACCCCAATGCCATCACTGGACTGAAACGACAAACTA  1673
              ||||||||||||||||||||||||||||||||||||||||||||||||||||||||||||
Seq_2  1739   GACTTGGACTACATGGATCGAGTTACCCCAATGCCATCACTGGACTGAAACGACAAACTA  1798


Seq_1  1674   CACAGCTCATCACTCACTGG----------------------------------------  1693
              ||||||||||||||||||||
Seq_2  1799   CACAGCTCATCACTCACTGGCGAATCGCATCCAAAAGTCCAGATAAGAAGATGATGGGCA  1858


Seq_1  1694   --------------------ACCATTTGTCTGCCGCAGCCCGGACTAGCTAGCACGTTGC  1733
                                  ||||||||||||||||||||||||||||||||||||||||
Seq_2  1859   CGGCCACTGGCCTGGACTGGACCATTTGTCTGCCGCAGCCCGGACTAGCTAGCACGTTGC  1918


Seq_1  1734   GCGAGGCACGTACGTACGTACGTGTCCTGCATGCACCTTTCCCAACTTAAATTAACCATG  1793
              |||||||||||||||||||    |||||||||||||||||| ||||||||||||||||||
Seq_2  1919   GCGAGGCACGTACGTACGT----GTCCTGCATGCACCTTTCTCAACTTAAATTAACCATG  1974


Seq_1  1794   CGCGATCGCCGATCATCAGCCCGGCCGTACATGATAATGGCGCTACTGGCCCATCAACCA  1853
              ||||||||||||||||||||||||||||||||||||||||||||||| ||||||||||||
Seq_2  1975   CGCGATCGCCGATCATCAGCCCGGCCGTACATGATAATGGCGCTACTAGCCCATCAACCA  2034


Seq_1  1854   GGACTAGGAGGAAGAGGGAGAGAGACCAGCTGTGTCGGACTCTCGATGGATCGAACAATA  1913
              ||||||||||||||||||||||||||||||||||||||||||||||||||||||||||||
Seq_2  2035   GGACTAGGAGGAAGAGGGAGAGAGACCAGCTGTGTCGGACTCTCGATGGATCGAACAATA  2094


Seq_1  1914   TGCATGGGTCCATGGGTCCCTGGATAAGCTCGTTTTGGATCTACGATAACTGTCATTCAT  1973
              ||||||||||||||||||||||||||||||||||||||||||||||||||||| ||||||
Seq_2  2095   TGCATGGGTCCATGGGTCCCTGGATAAGCTCGTTTTGGATCTACGATAACTGTGATTCAT  2154


Seq_1  1974   GGGCCATGCCCAGGAGGCGTGCACCATGCCACCATGGAGGACGCGGCCGGGTCGGATAGC  2033
              ||||||||||||||||||||||||||||||||||||||||||||||||||||||||||||
Seq_2  2155   GGGCCATGCCCAGGAGGCGTGCACCATGCCACCATGGAGGACGCGGCCGGGTCGGATAGC  2214


Seq_1  2034   TAAGCAGCCAAGCTTGGTTGGACATATGCGCGCCGAAATCACAACCATTCTGGGCCGTCG  2093
              ||||||||||||||||||||||||||||||||||||||||||||||||||||||||||||
Seq_2  2215   TAAGCAGCCAAGCTTGGTTGGACATATGCGCGCCGAAATCACAACCATTCTGGGCCGTCG  2274


Seq_1  2094   GATATTCTTTCATTTCCTGTCTTTAATCACAAATCTGAGACAATTTCTCCCGAATTTGAG  2153
              ||||||||||||||||||||||||||||||||||||||||||||||||||||||||||||
Seq_2  2275   GATATTCTTTCATTTCCTGTCTTTAATCACAAATCTGAGACAATTTCTCCCGAATTTGAG  2334


Seq_1  2154   AGAGCGACAATGATGAACCCGACTTGCAGACGCAGGGTCCCGATCGAGACGCGCGGTAAC  2213
              |||||||||||||||||||||||||||||||||||||||||||||||| ||||||||||
Seq_2  2335   AGAGCGACAATGATGAACCCGACTTGCAGACGCAGGGTCCCGATCGAGGCGCGCGGTAAA  2394


Seq_1  2214   CCATGTCCCGCCACCGGAAAACCAGCAGCGCTCGCCGTGGTCTCGCGTCGTGGGTCGTGG  2273
              ||||||||||||||||||||||||||||||||||||||||||||||||||||||||||||
Seq_2  2395   CCATGTCCCGCCACCGGAAAACCAGCAGCGCTCGCCGTGGTCTCGCGTCGTGGGTCGTGG  2454


Seq_1  2274   CCGCCCACCCGCGCGCGGCGCCGCCGGCCGCAAACACGTGTCCCCCCCAGTCCCCTACAC  2333
              ||||||||||||||||||||||||||||||||||||||||||||||||||||||||||||
```

```
Seq_2  2455  CCGCCCACCCGCGCGCGGCGCCGCCGGCCGCAAACACGTGTCCCCCCCAGTCCCCTACAC  2514

Seq_1  2334  TCACGTGCTCTCCTCTTTCGATCTCTTCCCTCTCCTTCACGGACGGGACGGGCACAGTGG  2393
             |||||||||||               ||||||||||||||||||||||||||||||||||
Seq_2  2515  TCACGTGCTCT---------------TCCCTCTCCTTCACGGACGGGACGGGCACAGTGG  2559

Seq_1  2394  ACGCGTGGAATATAACTCGAGTGAGTGACGCCGTCAATCTCGCCGTCGTCTTCTGCCTCT  2453
             ||||||||||||||||||||||||||||||||||||||||||||||||||||||||||||
Seq_2  2560  ACGCGTGGAATATAACTCGAGTGAGTGACGCCGTCAATCTCGCCGTCGTCTTCTGCCTCT  2619

Seq_1  2454  CGTACGTCACGCCTCGCTGTATATAAAAGAGAGCGGGCACCCATCCTCCGAGTACCCCAG  2513
             |||||||||||||||||||||||||||||||||||||||||||||||| ||||| |||||
Seq_2  2620  CGTACGTCACGCCTCGCTGTATATAAAAGAGAGCGGGCACCCATCCTCAGAGTAGCCCAG  2679

Seq_1  2514  GACACCGCACGCGCAGGCAGGCAGCAATGGAAGGCGCCACATCGTCCCTCACGCGCTCCC  2573
             ||| ||||||||||||||||||||||||||||||||||||||||||||||||||||||||
Seq_2  2680  GACGCCGCACGCGCAGGCAGGCAGCAATGGAAGGCGCCACATCGTCCCTCACGCGCTCCC  2739

Seq_1  2574  TCTCCCTCGCCCCCCGCCCACGCCGCCGCTCTCACCACAACCTGCCCACCCTCTCCCCCG  2633
             ||||||||||||||||||||||||||||||||||||||||||||||||||||||||||||
Seq_2  2740  TCTCCCTCGCCCCCCGCCCACGCCGCCGCTCTCACCACAACCTGCCCACCCTCTCCCCCG  2799

Seq_1  2634  CGCC--------------------------------------------------------  2637
             ||||
Seq_2  2800  CGCCCCGCGTGCTCCCGCTCCGCCGCGCCAGATCTGACGCCCACGACCTCCTCGGATCCA  2859

Seq_1  2638  ------------------------------------------------------------  2637

Seq_2  2860  TGGCCACCGCCGCCGCAGCGGCAACATCCTCCGCCGCCACCGTCCTCCCCCGCCCACGGA  2919

Seq_1  2638  ------------------------------------------------------------  2637

Seq_2  2920  CCCTCGACAGGGACAACGACTGCCTCTTCGCCGGCCCGGGCAACGGCAACGGCAACAACG  2979

Seq_1  2638  ------------------------------------------------------------  2637

Seq_2  2980  ACAACAGATCCGGCAACGGCGGCGGCGGGGGCGGCAACGGCGGCGGGAGCGGCCAGAGCG  3039

Seq_1  2638  ------------------------------------------------------------  2637

Seq_2  3040  CCGGCATGGGGGAGCACTACCGGCGCGCGCTGAGCCTGGACCCGTCCAACCCGCTGCTGC  3099

Seq_1  2638  ------------------------------------------------------------  2637

Seq_2  3100  TGCGCAACTACGGCAAGTTCCTCCACGACGTGCAGCGCGACCTGCCCGGTGCCCAGGACT  3159

Seq_1  2638  ------------------------------------------------------------  2637

Seq_2  3160  GCTACGCGCGCGCCATGCTCGCCTCCCCCGCCGACGCCGACCTGCTCAGCCTCTACGGCC  3219

Seq_1  2638  ------------------------------------------------------------  2637

Seq_2  3220  GCGCGCTCTGGGAGGCCGGCCAGGGCCACGGCCAGGCGGACAGGGACGGCAGCAAGGACC  3279

Seq_1  2638  ------------------------------------------------------------  2637
```

```
Seq_2  3280  GCGCCGAGGGATACTTCCAGCGCGCCGTCCAGGCCGCGCCCGACGACTGCCACGTGCTCG  3339

Seq_1  2638  ------------------------------------------------------------  2637

Seq_2  3340  CCTCCTACGCCAGCTTCCTCTGGGACGCCGAGGAAGACGACGTGGAGGACCAGGTCGCCT  3399

Seq_1  2638  ------------------------------------------------------------  2637

Seq_2  3400  GTGGCTCTCCGGCGTCCTTTGTGCCGGCGTGCTGACACAACCTTGATCTCGGCCATCTCA  3459

Seq_1  2638  ------------------------------------------------------------  2637

Seq_2  3460  TCTGCGGTCTGCTGTACTGTAACATAGGAGGCGCGCCGGCTGGAAGCAGGGTGATGGAGA  3519
```

BdindelWSU_13, DOWNSTREAM

>Bradi3g50320

AACAAGACTTTCCGAGTTACTGAGTTCCTCTGGTTGTAACAGTGAACTCAAAGCATGCCAACTGTTCCGGCCAGATCTGGGGGCTCGT
TGATTCGCAATATTTTCAAAACACAAGAATAGAAAAGCCATAGAACTGAATGCCATCAATGTCATGCTAAGATTATGTGCTAGTCATA
ATTGTCATAGAAACAGAGTTTTTTTTTCATGAGGGGTGACCTCATGAAAAATTTCCTTTTGCTTGCAAAGTACAATGTACCTATATTCCT
TACTGTACGTTTGGTTATGCTACATTCAGGAACTGAGGTAGTAGACCATAGGAAAAAATCACATGGTTTTAAATCCTACAAAATCAAA
CAAGTTGTATAGAAAGAAGTTCCTTTAAAATATGGTAATCCTCTAAAGTCTTACAATTTTTGGTGCAAGTTGAGCTGTCCTCGTGTTTG
AATTTTGAAGAGGGGTGTATCGAAGCAAAAGTGTAATTTTTTTTCACACTTTTTCATGATTCTTGAATAACATGTTTGGAATGTAGTGC
TAATAAACCATTGGTGCGTCCAAAATGTCGCTCACGATCTAATATGCAGGTTCGATAGAAAGAGCTCAGCTCCGCAATTTGATTGGTG
AAATTGTGGCCACACAAGACACGTATCCAGGTTGGCAGCAAACAAAAGAAAAAAGATGAAATTGTGGCCACACAAGACACGTATCC
AGGTTGGCAGCAAACAAAAGAAAAAAGATGTCAACCAAGATGCACAACCACATCTTCGAGCAAAAGACGGGTAAATGACAACCACT
GACAAGTCAACAACAATTACATGCCCTATAGACATCCAAGCATCTGACGGTAGGTCTCCAACCATGTCGGTTCAAGAGAGTTTCAAAC
GCTACCACGAACGCAGTCGATGGTGTGCATTCGAGTACTCTACGGACTGCGATGGACTACATTTCCCTGCTATGGAGGTACATATATT
TATTAGAGAAATCAATAATAGACAATCTCACTATTTAGGTCATCGGGTGGATGAGAAAGCGGGTTTGACGAGACATAAAAGTAATTA
TCGCCAAACTGATGGCACGACGATTGAATTTCTAGCGTCGAAAAAGTGACAACATGAAATGTAGAAACCAAAGCATGAGCAATGGTC
TAATATAAACAATACACAAAGGGTGTCACCGGGGGATTGAACTAGCCAAGTGATAAGGGATTGATTGCATGGCTTACTTGATAGTGC
TCGAATAAACGTGACAGGGACCGTTCAGATTGTTAGATGGAAGATGCATGTGAACTAGGTCAATTCAAGTCACGTTGTAAACTTGTA
ATCGTATAACAACACTAGTGTTAAGGGGTGTCAATGTTCGAGGGTGCATTAGTATGGCATCCACACTAATCCTATTAGAGATAGTCGT
TGTACACTCGATAGATTGACACATAGATGTTTTAGGATATGTTTGGTCGCCTCATGGGACGCAACATACTCATATGTTAGAAGAGTGG
GTGAGTGAAAGTTGGATTGGTCATTCGGGTTGTACAAAACTAACCGGGCCGACATTAATTATTCCTGACGCTCTTCCATCAAACTGGA
GGGGTACTCGACCCTTATGACCTTGTTGACTCTAGGAGCTCCATGCCTATGTGAACCTTTGAGCTCTCTAGGCATCTTTCCTTGAATTT
ATTGAAGTGACAATGGAATGAGCTACATCCTACGAGTGTGGTTATAGCAAAAGGTTCATGTCAAATGACCATGCATGGAGTTTTTGT
ATTAATGCAGTGTCCTCCTACTTATTATCCTCCTGTGCTTAGGTCCTTTCAATTCAGTTTCATTTAATCCATTTCTTGACTTGTTCGGCGT
ATTAATTTGCATAGTGGCTTGACCAAACCTCTTAGTCATTTTTGACCGGATGAATATTTGTGTTTACTTATTCTCTTGCCTGGTTAGAGA
CCGAATCGTGTAAAAATTATTTACATCCAAAAAGTTATTTATAAAACAAAACCAAGGAAAAGGAAAATATTAATAAGCAAAAGGATG
TATGCACATGCACTTTTCTTGACAGCTATGCACATGCACTAATACTTCATAGCTCTATTGATCGATCTCTGTTAAAACAAAAACCCTTTG
AGACAGTCGCGTAATCTTCGTTGTCACCATACACCACTAATCCCTTGAAGCTTTGAATAATCCACATGCGCCATGATTGTTTTCTCAATC
GACGATTTGCTAATCCGGAAGAAGATGTACGTACTATTGGAAGTTGAAATCGTTGTCCCACCAACTGCCGGTACGACGACAGCTAG

>BdiBd21-3.3G0666900

AACAAGACTTTCCGAGTTACTGAGTTCCTCTTGGTTGTAACAGTGAACTCAAAGCATGCCAACTGTTCCGGCCAGATCTGGGGGCTCG
TTGATTCGCAATATTTTCAAAACACAAGAATAGAAAAGCCATAGAACTGAATGCCATCAATGTCATGCTAAGATTATGTGCTAGTCAT
AATTGTCATAGAAACAGAGTTTTTTTTTTCATGAGGGGTGACCTCATGAAAAATTTCCTTTTGCTTGCAAAGTACAATGTACCTATATT
CCTTACTGTACGTTTGGTTATGCTACATTCAGGAACTGAGGTAGTAGACCATAGGAAAAAATCATATGGTTTTAAATCCTACAAAATC
AAACAAGTTGTATAGAAAGAAGTTTCTTTAAAATATGGTAATCCTCTAAAGTCTTACGATTTTTGAATCAAAGAGAGGCCTTTGTTCGT

CTTTGGAGTACATAGAACTATGAAAATAGAATGCGTACAATTTTGGAATAGCAAGTACGTCAATTTTATCTCAGGACACTACCGGTGG
CAAGTGTACAACAGGTTACGTGTTACTCCGTAATAACTATGCTGGGAGGTTAACTGGTTACAAATACTGTTTTTCGTCCTGCTTACAG
GATGCGGACAGCTGTCTAGAACTCTAGATGGTTGAAGCTGCCAGCTTGCCAGGCACTCACCGCACGATCGACCAATCGTACACACCG
CGCTGGCAACCTCCGGTCATCAAAGCCGGAGAACCAAGTGGCTGGCAACAATGATGAGGCGCCAAGGAGGCAACGTCTGCATTCAG
CACTTGCCTTTCAAAGAGGCCTTTGTTGGTCTTTCAAAAATTGTTTATTTTGTACAGCGTTCACACATGTGTTCATCACACACACCAGCG
CCAGTAAACCGCGACCTCACCCGTCCTGCGCCTCGGGAAGGTGAAGCGGCATGGGGAACATACCCTCTCCGTGAACTATCAGCTAAT
TGGGGAGCAGCGGCAGCTTAGTTGGCGCTAGCAATTTCTAATCCCGCTTGACCAGGCTCGGAATAAGTGCGATTAGAGGTTCAATTG
ATGAGAAATTTTAGAGAAATAGTATTAGGATATAAGAGTGACAGAGAGAGGCGAGGTGAGGAACGGAGCCATTGCCGCCGAGAGC
ATGCACAGAAGAGAGAGGTAGGCAGAGAGCAGGCGTTGGACGGAGAATGAGCCACAAAGGAGAGCTGGACTTATAAATTCTAAAT
TTACCATCATGCAATTCTTATAGAAGGGGCATGGTGCAAGTTGAGCCGTCCTCGTGTTTGAATTTTGAAGAGGGGTGTATCGAAGCA
AAAGTGTAATTTTTTTTCACACTTTTTCATGATTCTTGAATAACATGTTTGGCATGTAGTGCTAATAAACCATTGGTGCGTCCAAAATGT
CGCTCACGATCTAATATGCAGGTTCGATAAAAAGAGCTCAGCTCCGCAATTTGATTGGTGAAATTGTGGCCACACAAGACACGCATCC
AGGTTGGCAGCAAACAAAAGAAAAAAGATGTCAACCAAGATGCACAACCACATCTTCGAGCAAAAGACGGGTAAATGACAACCACT
GACAAGTCAACAACAATTACATGCCCTATAGACATCCAAGCATCTGACGGTAGGTCTCCAACCATGTCGGTTCAAGAGAGTTTCAAAC
GCTACCACGAACGCAGTCGATGGTGTGCATTCGAGTACTCTACGGACTGCGATGGACTACATTTCCCTGCTATGGAGGTACATATATT
TATTAGAGAAATCAATAATAGACAATCTCACTATTTAGGTCATCGGGTGGATGAGAAAGCGGGTTTGACGAGACATAAAAGTAATTA
TCGCCAAACTGATGGCACGACGATTGAATTTCTAGCGTCCAAAAAGTGACAACATGAAATGTAGAAACCAAAGCATGAGCAATGGTC
TAATATAAACAATACACAAAGGGTGTCACCGGGGGATTGAACTAGCCAAGTGATAAGGGATTGATTGCATGGCTTACTTGATAGTGC
TCGAATAAACGTGACAAGGGACCGTTCAGATTGTTAGATGGAAGATGCATGTGAACTAGGTCAATTCAAGTCACGTTGTAAACTTGT
AATCGTATAACAACACTAGTGTTAAGGGGTGTCAATGTTCGAGGGTGTATTAGTATGGCATCCACACTAATCCTATTAGAGATAGTCG
TTGTACACTCGATAGATTGACACATAGATGTTTTAGGATATGTTTGGTCGCCTCATGGGACGCAACATACTCATATGTTAGAAGAGTG
GGTGAGTGAAAGTTGGATTGGTCATTCGGGTTGTACAAAACTAACCGGGCCGACATTAATTATTCCTGACGCTCTTCCATCAAACTGG
AGGGGTACTCGACCCTTATGACGTTGTTGACTCTAGGAGCTCCATGCCTATGTGAACCTTTGAGCTCTCTAGGCATCTTTC

```
Alignment of Sequence_1:  [Untitled Sequence #1] with  Sequence_2: [Sequence Window #2]

Similarity : 1593/2445 (65.15 %)

Seq_1  1      AACAAGACTTTCCGAGTTACTGAGTTCCTCT-GGTTGTAACAGTGAACTCAAAGCATGCC  59
              ||||||||||||||||||||||||||||||| ||||||||||||||||||||||||||||
Seq_2  1      AACAAGACTTTCCGAGTTACTGAGTTCCTCTTGGTTGTAACAGTGAACTCAAAGCATGCC  60


Seq_1  60     AACTGTTCCGGCCAGATCTGGGGGCTCGTTGATTCGCAATATTTTCAAAACACAAGAATA  119
              ||||||||||||||||||||||||||||||||||||||||||||||||||||||||||||
Seq_2  61     AACTGTTCCGGCCAGATCTGGGGGCTCGTTGATTCGCAATATTTTCAAAACACAAGAATA  120


Seq_1  120    GAAAAGCCATAGAACTGAATGCCATCAATGTCATGCTAAGATTATGTGCTAGTCATAATT  179
              ||||||||||||||||||||||||||||||||||||||||||||||||||||||||||||
Seq_2  121    GAAAAGCCATAGAACTGAATGCCATCAATGTCATGCTAAGATTATGTGCTAGTCATAATT  180


Seq_1  180    GTCATAGAAACAGAGTTTTTTTTT-CATGAGGGGTGACCTCATGAAAAATTTCCTTTTGC  238
              |||||||||||||||||||||||| |||||||||||||||||||||||||||||||||||
Seq_2  181    GTCATAGAAACAGAGTTTTTTTTTTCATGAGGGGTGACCTCATGAAAAATTTCCTTTTGC  240


Seq_1  239    TTGCAAAGTACAATGTACCTATATTCCTTACTGTACGTTTGGTTATGCTACATTCAGGAA  298
              ||||||||||||||||||||||||||||||||||||||||||||||||||||||||||||
Seq_2  241    TTGCAAAGTACAATGTACCTATATTCCTTACTGTACGTTTGGTTATGCTACATTCAGGAA  300


Seq_1  299    CTGAGGTAGTAGACCATAGGAAAAAATCACATGGTTTAAATCCTACAAAATCAAACAAG  358
              ||||||||||||||||||||||||||||| ||||||||||||||||||||||||||||||
Seq_2  301    CTGAGGTAGTAGACCATAGGAAAAAATCATATGGTTTAAATCCTACAAAATCAAACAAG  360


Seq_1  359    TTGTATAGAAAGAAGTTCCTTTAAAATATGGTAATCCTCTAAAGTCTTAC----------  408
              ||||||||||||||||| ||||||||||||||||||||||||||||||||
Seq_2  361    TTGTATAGAAAGAAGTTTCTTTAAAATATGGTAATCCTCTAAAGTCTTACGATTTTTGAA  420
```

```
Seq_1  409   ------------------------------------------------------------  408

Seq_2  421   TCAAAGAGAGGCCTTTGTTCGTCTTTGGAGTACATAGAACTATGAAAATAGAATGCGTAC  480

Seq_1  409   AATTTT------------------------------------------------------  414
             ||||||
Seq_2  481   AATTTTGGAATAGCAAGTACGTCAATTTTATCTCAGGACACTACCGGTGGCAAGTGTACA  540

Seq_1  415   ------------------------------------------------------------  414

Seq_2  541   ACAGGTTACGTGTTACTCCGTAATAACTATGCTGGGAGGTTAACTGGTTACAAATACTGT  600

Seq_1  415   ------------------------------------------------------------  414

Seq_2  601   TTTTCGTCCTGCTTACAGGATGCGGACAGCTGTCTAGAACTCTAGATGGTTGAAGCTGCC  660

Seq_1  415   ------------------------------------------------------------  414

Seq_2  661   AGCTTGCCAGGCACTCACCGCACGATCGACCAATCGTACACACCGCGCTGGCAACCTCCG  720

Seq_1  415   ------------------------------------------------------------  414

Seq_2  721   GTCATCAAAGCCGGAGAACCAAGTGGCTGGCAACAATGATGAGGCGCCAAGGAGGCAACG  780

Seq_1  415   ------------------------------------------------------------  414

Seq_2  781   TCTGCATTCAGCACTTGCCTTTCAAAGAGGCCTTTGTTGGTCTTTCAAAAATTGTTTATT  840

Seq_1  415   ------------------------------------------------------------  414

Seq_2  841   TTGTACAGCGTTCACACATGTGTTCATCACACACACCAGCGCCAGTAAACCGCGACCTCA  900

Seq_1  415   ------------------------------------------------------------  414

Seq_2  901   CCCGTCCTGCGCCTCGGGAAGGTGAAGCGGCATGGGGAACATACCCTCTCCGTGAACTAT  960

Seq_1  415   ------------------------------------------------------------  414

Seq_2  961   CAGCTAATTGGGGAGCAGCGGCAGCTTAGTTGGCGCTAGCAATTTCTAATCCCGCTTGAC  1020

Seq_1  415   ------------------------------------------------------------  414

Seq_2  1021  CAGGCTCGGAATAAGTGCGATTAGAGGTTCAATTGATGAGAAATTTTAGAGAAATAGTAT  1080

Seq_1  415   ------------------------------------------------------------  414

Seq_2  1081  TAGGATATAAGAGTGACAGAGAGAGGCGAGGTGAGGAACGGAGCCATTGCCGCCGAGAGC  1140

Seq_1  415   ------------------------------------------------------------  414

Seq_2  1141  ATGCACAGAAGAGAGAGGTAGGCAGAGAGCAGGCGTTGGACGGAGAATGAGCCACAAAGG  1200

Seq_1  415   --------------------------------------------------------TGGT  418
                                                                     ||||
Seq_2  1201  AGAGCTGGACTTATAAATTCTAAATTTACCATCATGCAATTCTTATAGAAGGGGCATGGT  1260
```

```
Seq_1  419   GCAAGTTGAGCTGTCCTCGTGTTTGAATTTTGAAGAGGGGTGTATCGAAGCAAAAGTGTA  478
             ||||||||||| ||||||||||||||||||||||||||||||||||||||||||||||||
Seq_2  1261  GCAAGTTGAGCCGTCCTCGTGTTTGAATTTTGAAGAGGGGTGTATCGAAGCAAAAGTGTA  1320

Seq_1  479   ATTTTTTTTCACACTTTTTCATGATTCTTGAATAACATGTTTGGAATGTAGTGCTAATAA  538
             |||||||||||||||||||||||||||||||||||||||||||| |||||||||||||||
Seq_2  1321  ATTTTTTTTCACACTTTTTCATGATTCTTGAATAACATGTTTGGCATGTAGTGCTAATAA  1380

Seq_1  539   ACCATTGGTGCGTCCAAAATGTCGCTCACGATCTAATATGCAGGTTCGATAGAAAGAGCT  598
             ||||||||||||||||||||||||||||||||||||||||||||||||||| ||||||||
Seq_2  1381  ACCATTGGTGCGTCCAAAATGTCGCTCACGATCTAATATGCAGGTTCGATAAAAAGAGCT  1440

Seq_1  599   CAGCTCCGCAATTTGATTGGTGAAATTGTGGCCACACAAGACACGTATCCAGGTTGGCAG  658
             ||||||||||||||||||||||||||||||||||||||||||||| ||||||||||||||
Seq_2  1441  CAGCTCCGCAATTTGATTGGTGAAATTGTGGCCACACAAGACACGCATCCAGGTTGGCAG  1500

Seq_1  659   CAAACAAAAGAAAAAAGATGAAATTGTGGCCACACAAGACACGTATCCAGGTTGGCAGCA  718
             ||||||||||||||||||||
Seq_2  1501  CAAACAAAAGAAAAAAGATG----------------------------------------  1520

Seq_1  719   AACAAAAGAAAAAAGATGTCAACCAAGATGCACAACCACATCTTCGAGCAAAAGACGGGT  778
                               ||||||||||||||||||||||||||||||||||||||||||
Seq_2  1521  ------------------TCAACCAAGATGCACAACCACATCTTCGAGCAAAAGACGGGT  1562

Seq_1  779   AAATGACAACCACTGACAAGTCAACAACAATTACATGCCCTATAGACATCCAAGCATCTG  838
             ||||||||||||||||||||||||||||||||||||||||||||||||||||||||||||
Seq_2  1563  AAATGACAACCACTGACAAGTCAACAACAATTACATGCCCTATAGACATCCAAGCATCTG  1622

Seq_1  839   ACGGTAGGTCTCCAACCATGTCGGTTCAAGAGAGTTTCAAACGCTACCACGAACGCAGTC  898
             ||||||||||||||||||||||||||||||||||||||||||||||||||||||||||||
Seq_2  1623  ACGGTAGGTCTCCAACCATGTCGGTTCAAGAGAGTTTCAAACGCTACCACGAACGCAGTC  1682

Seq_1  899   GATGGTGTGCATTCGAGTACTCTACGGACTGCGATGGACTACATTTCCCTGCTATGGAGG  958
             ||||||||||||||||||||||||||||||||||||||||||||||||||||||||||||
Seq_2  1683  GATGGTGTGCATTCGAGTACTCTACGGACTGCGATGGACTACATTTCCCTGCTATGGAGG  1742

Seq_1  959   TACATATATTTATTAGAGAAATCAATAATAGACAATCTCACTATTTAGGTCATCGGGTGG  1018
             ||||||||||||||||||||||||||||||||||||||||||||||||||||||||||||
Seq_2  1743  TACATATATTTATTAGAGAAATCAATAATAGACAATCTCACTATTTAGGTCATCGGGTGG  1802

Seq_1  1019  ATGAGAAAGCGGGTTTGACGAGACATAAAAGTAATTATCGCCAAACTGATGGCACGACGA  1078
             ||||||||||||||||||||||||||||||||||||||||||||||||||||||||||||
Seq_2  1803  ATGAGAAAGCGGGTTTGACGAGACATAAAAGTAATTATCGCCAAACTGATGGCACGACGA  1862

Seq_1  1079  TTGAATTTCTAGCGTCGAAAAAGTGACAACATGAAATGTAGAAACCAAAGCATGAGCAAT  1138
             |||||||||||||||| |||||||||||||||||||||||||||||||||||||||||||
Seq_2  1863  TTGAATTTCTAGCGTCCAAAAAGTGACAACATGAAATGTAGAAACCAAAGCATGAGCAAT  1922

Seq_1  1139  GGTCTAATATAAACAATACACAAAGGGTGTCACCGGGGGATTGAACTAGCCAAGTGATAA  1198
             ||||||||||||||||||||||||||||||||||||||||||||||||||||||||||||
Seq_2  1923  GGTCTAATATAAACAATACACAAAGGGTGTCACCGGGGGATTGAACTAGCCAAGTGATAA  1982

Seq_1  1199  GGGATTGATTGCATGGCTTACTTGATAGTGCTCGAATAAACGTGACA-GGGACCGTTCAG  1257
             ||||||||||||||||||||||||||||||||||||||||||||||| ||||||||||||
Seq_2  1983  GGGATTGATTGCATGGCTTACTTGATAGTGCTCGAATAAACGTGACAAGGGACCGTTCAG  2042
```

```
Seq_1  1258  ATTGTTAGATGGAAGATGCATGTGAACTAGGTCAATTCAAGTCACGTTGTAAACTTGTAA  1317
             ||||||||||||||||||||||||||||||||||||||||||||||||||||||||||||
Seq_2  2043  ATTGTTAGATGGAAGATGCATGTGAACTAGGTCAATTCAAGTCACGTTGTAAACTTGTAA  2102

Seq_1  1318  TCGTATAACAACACTAGTGTTAAGGGGTGTCAATGTTCGAGGGTGCATTAGTATGGCATC  1377
             ||||||||||||||||||||||||||||||||||||||||||||| ||||||||||||||
Seq_2  2103  TCGTATAACAACACTAGTGTTAAGGGGTGTCAATGTTCGAGGGTGTATTAGTATGGCATC  2162

Seq_1  1378  CACACTAATCCTATTAGAGATAGTCGTTGTACACTCGATAGATTGACACATAGATGTTTT  1437
             ||||||||||||||||||||||||||||||||||||||||||||||||||||||||||||
Seq_2  2163  CACACTAATCCTATTAGAGATAGTCGTTGTACACTCGATAGATTGACACATAGATGTTTT  2222

Seq_1  1438  AGGATATGTTTGGTCGCCTCATGGGACGCAACATACTCATATGTTAGAAGAGTGGGTGAG  1497
             ||||||||||||||||||||||||||||||||||||||||||||||||||||||||||||
Seq_2  2223  AGGATATGTTTGGTCGCCTCATGGGACGCAACATACTCATATGTTAGAAGAGTGGGTGAG  2282

Seq_1  1498  TGAAAGTTGGATTGGTCATTCGGGTTGTACAAAACTAACCGGGCCGACATTAATTATTCC  1557
             ||||||||||||||||||||||||||||||||||||||||||||||||||||||||||||
Seq_2  2283  TGAAAGTTGGATTGGTCATTCGGGTTGTACAAAACTAACCGGGCCGACATTAATTATTCC  2342

Seq_1  1558  TGACGCTCTTCCATCAAACTGGAGGGGTACTCGACCCTTATGACCTTGTTGACTCTAGGA  1617
             |||||||||||||||||||||||||||||||||||||||||||| |||||||||||||||
Seq_2  2343  TGACGCTCTTCCATCAAACTGGAGGGGTACTCGACCCTTATGACGTTGTTGACTCTAGGA  2402

Seq_1  1618  GCTCCATGCCTATGTGAACCTTTGAGCTCTCTAGGCATCTTTCCTTGAATTTATTGAAGT  1677
             |||||||||||||||||||||||||||||||||||||||||||
Seq_2  2403  GCTCCATGCCTATGTGAACCTTTGAGCTCTCTAGGCATCTTTC-----------------  2445

Seq_1  1678  GACAATGGAATGAGCTACATCCTACGAGTGTGGTTATAGCAAAAGGTTCATGTCAAATGA  1737

Seq_2  2446  ------------------------------------------------------------  2445

Seq_1  1738  CCATGCATGGAGTTTTTGTATTAATGCAGTGTCCTCCTACTTATTATCCTCCTGTGCTTA  1797

Seq_2  2446  ------------------------------------------------------------  2445

Seq_1  1798  GGTCCTTTCAATTCAGTTTCATTTAATCCATTTCTTGACTTGTTCGGCGTATTAATTTGC  1857

Seq_2  2446  ------------------------------------------------------------  2445

Seq_1  1858  ATAGTGGCTTGACCAAACCTCTTAGTCATTTTTGACCGGATGAATATTTGTGTTTACTTA  1917

Seq_2  2446  ------------------------------------------------------------  2445

Seq_1  1918  TTCTCTTGCCTGGTTAGAGACCGAATCGTGTAAAAATTATTTACATCCAAAAAGTTATTT  1977

Seq_2  2446  ------------------------------------------------------------  2445

Seq_1  1978  ATAAAACAAAACCAAGGAAAAGGAAAATATTAATAAGCAAAAGGATGTATGCACATGCAC  2037

Seq_2  2446  ------------------------------------------------------------  2445

Seq_1  2038  TTTTCTTGACAGCTATGCACATGCACTAATACTTCATAGCTCTATTGATCGATCTCTGTT  2097

Seq_2  2446  ------------------------------------------------------------  2445
```

```
Seq_1  2098  AAAACAAAAACCCTTTGAGACAGTCGCGTAATCTTCGTTGTCACCATACACCACTAATCC  2157

Seq_2  2446  ------------------------------------------------------------  2445

Seq_1  2158  CTTGAAGCTTTGAATAATCCACATGCGCCATGATTGTTTTCTCAATCGACGATTTGCTAA  2217

Seq_2  2446  ------------------------------------------------------------  2445

Seq_1  2218  TCCGGAAGAAGATGTACGTACTATTGGAAGTTGAAATCGTTGTCCCACCAACTGCCGGTA  2277

Seq_2  2446  ------------------------------------------------------------  2445

Seq_1  2278  CGACGACAGCTAG  2290

Seq_2  2446  -------------  2445
```

BdindelWSU_14, UPSTREAM

>Bradi3g60610

GCTTCTCCTCCGCGCAGCCGACGCCGCCATTGGACGCGGCGGACGAGTCCCCGCCATTGGACTCCGTGGTGGTCTCCTCGTCGCGGG
CGGCGCGGACCCAGGCCGGGATGTCGTCGGCCTGGAGCATGTCCTGGAACGGCGCCCGGCCCGTCAGCAGCTCCAGCAGCAGCACC
CCGAAGCTGTACACGTCCGACGCCGCCGTGAAGAGCCCACCGCCGGCGGAGCGGGTCTCCGGGGCGCGGTAGAGGAGCGAGGCG
GCGGCGGCGTCGGCGGAGGAGGGGCTGTGGAGAGCCGGGACGAGGCCGTAGTCGGTGAGGCAGGACTCGAAGTCGGGGCCCAG
GAGCACGTTGGACGGCTTCAGGTTGCCGTGCACGATGCCGATGCCGGCAGGAGAAGAGTGCGTGTGCAGGTGCAGGAGGCCGGCG
GCGATGTCCTCGGCGATCTTCATGCACGACGTCCAGTGCAGCGGCTTCCCCTTGCTCGACGGCCGAGACCCTGCATCCAACCAACCAT
GCATCAAGCGCCAACAGTTTCAGAATTATTTACACAAGCTGCTTGCTTTAGCACAAGTAAAAAAAATTAGAAGGCGACAGGTGAAAA
TTAACGGAGCAGGTGGTACATGGAGGCCTGGTTATTTATTTACTGATTTACAGTCACTGATCATCATCAGGAGGGCATGCTTTTGCAT
TGCTAGATGGAGTTGCAGCCATGGCGAAAAACAGAGCAGGGGAGGTCCTACCATCTCATGGCTTTTTGACTGGCTGGTATAACTAAT
TCTGGGACAGCTACACAGACTGCACGAGCACCATCATGGTCATGGACCCCGCTACACAATACATTGACACCAAGATAGCTAGGCATG
GAGTTGAGAGCATCATGGGTTTCTGAACAGCAGAATTATGTAGGACAACCAATACTAGATTAAGACAACACCTTCTTTACTTGCAAGA
AATAAATTTATTACTACTGTGACAGTGTGACTCACATGAGCCTCCCCCCATGTTCATGTGTCCAAGGTTCAGTCAGAGCTAGTGGTTTA
TTTATTTTTGGTCCCTAGATTCATGGCAGCGTGTCTCCATGAGAACATGTTCCCGGGATCCTCTCCTCTGCGGGCAACTAATGGTGTAA
GGTTTCTTGTCGATCTTGTTTGTCATGTATGGGAACCAATACGGGGATTCGCATCAATCGGAGGCCGGAGATTATCCTCATTTCCGGG
GGAAAGATGAAGCTAATAAAGCTAATTTTGTTCTCTTCTTGCACTTCACAAATCTCACAACTCTGTGATACGGAGAATTTACCGCTGTT
GGTGCTGGATCGATGAAGAATTTACCCGTCCAATATAGCACTCAAACAGCACAGTACTGGCTAGAACTTCACTTCATGTGGGACACT
GGGACTCAACCTTATGCATGGAGGATGAATAAAGTTGGCTTTTAAGCTAGGGCCATGCCGGGTCTTGTTGTGTTCCATTTTTTCACAT
GGCTTGGCACTGTACTCCTGGTACACATGGTTTGAATTCTTTCATCTGTTTCTGTAAATAAATTCAGAGGTGGACCAAACTAGAGAAG
GTACTAGCAACAGCTGGAGCATGTATACATACGTACGTATGTACGGGGTATAATGTACGTGCAGAAGAGCCCAGATTAAGCTTTGGA
TTTGGAATGTAGCAGCAGCAGCAGCAAGCTAATAAGCTCCATTAAGCTATGGAACGGAGGCAACAGAAGAGTCCAAATCAAGAGCC
CTTCTTCTGCTTCTTCCTTGCAGACCCGGCCTTTTTGTTTGCAGTGATTGCAATGCACGTACGGCAGATGACAGATCCATATATGCTGC
TAGATACTCCCCAGCAAGAAAGACAGGCAAACATATATGCAATGCAAATGCAGCTTTAACTTATGCATTGCTCTATACCGTGACACGA
AGAAATTTAAATTTTCACCCCCTTTTCGTGATCCGATTTGACATTTTGATTCGTACGAACGAGAGCACGGGCAGGGAGGATCAGCTGC
CTGCCAGCACTTAATCAGCAGGACTTGTTCGTGTGTGTCGACATGAATGGAATGGAAATTAAATCAAGCCGTGTGGATTAGCAGTAG
GAATTTCCGCATTTGGTTCGTAACAATTAAGGAGGAAGAAGAAGGCAATGGCAATGGAGACATGGAGTACTGACCGTGGATGAGG
GAGAAGAGGCTGCCGTTGGGGTAGTAGTCGTAGACGAGGAGCCGCTCCTCCTTGGCCTGGAAGTAGGCCCGGAGCGCCACCACGTT
GGGGTGCCGCACCCGCCCCAGCTCCTCCGCGCGCCGCCCGAACCCCGCCGCGTCGCCATGGCCGTCGCCGCCGCCGCAACGCATCCG
CTTCACCGTCACGATGAACCCCGTCTCCATCACCGCCTTGTACGTGCTCCCGGCTTCCCCTCTCCCCAGCGTCTCCGCCGACGCCCGCA
GCAGCTCCTCCAGGCTGTACATCCCGCCGCCGCCGCAGAACACCAGCTTCCCCATCCCCCCCTCCCGCTCCCACGAGAACTCCCTGGC
CGCCATTGCTGCTGATGGTGCTGCTGGTGGCGGCAATGGCGCGCTCGCCGCCGGCTGCTCCGACGATGAAGGAATTGCTTCGGCCTT
GTTGTTGTGACCCTCGTCGCCGGCCACGCGCTGCTTGCTCCCGCGCCCGCGCCGCGACGCCATTATCACCGCCGCTGCTAGGACTCCC

AGCAGCACCGCTCCGGCCACCGTGGAGCCTGCAACAATCGCCGCCTTCTTGCGGCGACGGCTGCTACTGGAGGAGCCGCCGCCGGG
CGGAGGGAGCGGCGAGAAGGCCGCCCTGGCAGGGGACAGCGGCGCTGCGGCGTCGCAAAGCGTGGGGAGCGGAGGCCCGCAGA
GCCCGCCGCCATTACCGGCGAATGACGACGCGTTAAACCTCGCGGCTAGCACGGGCGGGATGCGGCCGGAGAGCCGGTTCCCGGA
GACATCGAGCACCCGAAGCGTCCGCTGCGAGAACCCCGGGACAGGGCCGGCGAGGAGGTTCCCGTCGAGCATTAGCGCGGTGAGC
CGGGGCGGCGCGTCCGCGAGCTCCTCCGGGATCTCCCCCGTGAGGCGGTTGCCGGAGAGGACGAGGACGGTGGCGCGGCGGAGC
GAGGCGAGGTCCGGCGGGATCTCGCCCGTGAGGCGGTTGCCGGAGAGGTAGAGCAGCTTGAGGTTGGGGAGCGCGGCGGGGAG
GCCCGCCGGGACCGGGCCGCTCAGGGCGTTGTCCTTGAGGCTGAGGACGCGGAGCTCCGGGAACGGGGAGAGGAGCGCCGCGGT
GAGGAAGCCCGTGAGGTTGAGGTTTTCCAGGACGAGCTTCGTCACGCGCCCGCCATGGCCGCACTGGCGGATGCCCGGCCAGGAG
GTGCAGAGGGTGGAGGCCGTGTCGGGCCGCCATGGGAGGCGGTTGGAGTTGTCCAGGGCGGATTTTAGGGCTAGCAGCGCTTCCG
CTTCGCCGCCGGTGACCGGCGGGGCGAGGTGGTGGAGGAAGAGGAGAATGAGCAGCGGGGAGATGGCTCTTGGGTGGAGCATGG
TGGAGTGAGCTTTGGCGGTAGAGCAAGAGGAGTGAGCAAGTCAGCAGCAATGGCCGGTGGGCGGCATTGATTATTTTCTTTGCTTG
TTTGTTTGTTGGTGGAAGGACGGATTGAGTGAAGGGAGCTGTAAAAGAGGGAAGAAGAAAAGGGGAAGGAGGACGAAGCAGAGG
CAGAGGAAACCTGAGTCGGGAAAGGAAAGGAAACCGCTTTGAGGTCGCTGCTCAACTAAATGCAGCTGTGTCCTGTGTGCGTGTGG
TGTGGCTGGCGGCGGGAGGAGGAAGAAGAAGAAGAAGAAGAAGAAGACAGCTAGCTAGCCGGGTGGGATTCAGACGAAGAGCG
TCAGTTATTGAGTTCGCAGTTACTGTATTTGTTTCAGACGAAGAGCGTCAGAAACTGATGGGAAAATAAACCGACAGCTTTGGTCTTG
GTCTGAATATCTGATGGCGATGCAGCAAAGCAAGCAAGCTCGGCTGCTGAGGTGCAGCAATGGCGGCTAGCGAAGGGAGAAAGAA
TGGGGAATCAAGTGTTGGAGTCTGAGCTACAGCGAGCTTTCTCTGTGTCGCTTTAGCTAGCTGATGATTTCTACTCTTGATAACTTGG
CGGTAAGCTAAGCTAAAGCAGCATGCACAGCGATTATACCCTCCAGTGGGCAGTCAGCGCAGCTCCTCGGGGTCGTGAGGGAAAAA
AAAGAAAAGCCTGTGTCCTGGCCATTTTACTAGTCGCGTATGTGGCCGGCAAAGAGCAGCAGAGGGAAGGATTACTATCATCCCTTC
TCTGCTCTAGCCCTTGACTGACAAGCTCCATTATTTAGTCTCCGGTGTTGTTTTTCGATTTGAAAGCTTGTTTTGACGGTACGGAGAGC
GAGCACGGAGTAGTAAATCAATCTTATCTGGGTGAGAGATACTGAGATGCTCAGTCAATGCCATTACGCACGTCGTAGCACCCTTTA
CTTAATGATTAACCATGCTGCTGCTAGTTAACTCGATGGGCTTCGGAGTTGAGACGAGCGAGAGAGAGAAATCCAGATCGTGATGCA
ACATGGAGAGTACTACTCCCACTATGTTTATGTATGCCCGTGAGAGAGAGAATGAGATTGGGTCGCAGGAGGAGGAAGAAGAAGG
AGGACGAAATGGGTGAAAGAATTCAATGGTGCCACGTGCATACAGTGCACTGCTGCTCTCTCTCTCTGCAGATCCAACGCACTTCAAT
AGGACACTGCCTGTACTATTAACGCCACCAACAAGATGAAGCTATAGAGTTTTTTGAGAAACAGTTTTCCTTCTTTTGCAAAAAGAAA
AAACTTTCGATGGGCCTCTCTCTCGCCCGGGGCTTTCTTCTTTGTGGAGCAGGCCTTATTGACTGCTTCGTGGTGTCCGGCCCAACGG
CCCGTATATAACACGAGAACATTCTACCAGGCCCCAAAAGCATTGCCATTGTCCGGCCCAGTGGCAACGTGCGAACACTAACATATG
GGACCCCATGTTACCCTGAGTACGTCATAAAATCTTCCTCCTCCCAATGGAGCCAAAAGATAGTGAACCACGATCAGAGTTTTGCTCT
TAGACGAACAACTTGCAGCGTTCACATATTTCTTTCCTCCTTGTCGCTCATCTCCAATCTTGTTTTGTTTTGCATGGCCTGCATCTCATCT
CATGACGTCCGCTGAAGATCCATTATATCGTCGCTAGTAGTGATAGCCTTTGTTTAAGCCTGGTGCGAGTTTGTAATCGAAGGATTAT
GCTCACTAGTATCTTGTTAAGACATATATCGTCTCTTGGATTGGCATCGGTCTTAATGAGCTCGGAATCGCTGATCGACACAATCTATG
GCAGATCTATAATGTTATTCCGAGATAATAAAAATAGTTGCAACCTACATTGATCCCCAAAAAATAGATCAGTATACTGAAACAGTGA
GCAAATGGAACACAAGGTATGAATGAAAACAAAGGGTGTGGGGTTGCCATTTTAGGCTAACTAAGCAGGCTGTCAGGCAGAAAGA
AATACTCTTGACAGATTAGCACACCACAGCGAGAGAGAGAGGGGAAGGGGAAGGAGGGGGAACCCATCGGCTGCGGCGTGGCGA
TCTCGAGCTGGCGACCCCGCGCTCCTCCCACCACCTCGCCTCCGCCGCCGCCCTCGCGGCAGCGGGGGTCTCCTACCCGCCGCCGCTG
CCCCCCACCACGCCCAGCGCCGCCGCGCCGCCGCACGACCCCGGCGCCGGCGGCGCCGCCAAGCAGATCGTCGACTCCCTCCTCGCC
CGCTTCCTCCCGCTCGCCCGCCGCCGCATCGAGACCGCGCAGGCCCAGGTGATTCTGCCTGCTCCTCTCCCCCCCAACTCCTCTCCCCC
ACGTTCCGAATTCCGGCTGATTCCCGTTCCCGCGAGAGGTCAACGAAAATCGCACCACCTAGGGCTGCCTGTCCGCATCGCGTCCATG
TAGATTCGGGGGAGCATCGCTTAGGGCACGCTGGTGGGGTTCAATTTAAGAGGCACCTTCTACTTTGCTCAAGCACACGTCAATTCA
ATTAGGTTAGAGGTCTGACCAATGCTCCAAGATATCGCCTGCCCGCCTGCCCATGGTGCTACATTTCATTTGCTCACTGCCACGCACCC
ACCAATTTGGAA

>BdiBd21-3.3G0798600

GAGTCCAAATCAAGAGCCCTTCTTCTGCTTCTTCCTTGCAGACCCGGCCTTTTTGTTTGCAGTGATTGCAATGCACGTACGGCAGATGA
CAGATCCATATATGCTGCTAGATACTCCCCAGCAAGAAAGACAGGCAAACATATATGCAATGCAAATGCAGCTTTAACTTATGCATTG
CTCTATACCGTGACACGAAGAAATTTAAATTTTCACCCCCTTTTCGTGATCCGATTTGACATTTTGATTCGTACGAACGAGAGCACGGG
CAGGGAGGATCAGCTGCCTGCCAGCACTTAATCAGCAGGACTTGTTCGTGTGTGTCGACATGAATGGAATGGAAATTAAATCAAGCC
GTGTGGATTAGCAGTAGGAATTTCCGCATTTGGTTCGTAACAATTAAGGAGGAAGAAGAAGGCAATGGCAATGGAGACATGGAGTA
CTGACCGTGGATGAGGGAGAAGAGGCTGCCGTTGGGGTAGTAGTCGTAGACGAGGAGCCGCTCCTCCTTGGCCTGGAAGTAGGCC

CGGAGCGCCACCACGTTGGGGTGCCGCACCCGCCCCAGCTCCTCCGCGCGCCGCCCGAACCCCGCCGCGTCGCCATGGCCGTCGCCG
CCGCCGCAACGCATCCGCTTCACCGTCACGATGAACCCCGTCTCCATCACCGCCTTGTACGTGCTCCCGGCTTCCCCTCTCCCCAGCGT
CTCCGCCGACGCCCGCAGCAGCTCCTCCAGGCTGTACATCCCGCCGCCGCCGCAGAACACCAGCTTCCCCATCCCCCCCTCCCGCTCCC
ACGAGAACTCCCTGGCCGCCATTGCTGCTGATGGTGCTGCTGGTGGCGGCAATGGCGCGCTCGCCGCCGGCTGCTCCGACGATGAA
GGAATTGCTTCGGCCTTGTTGTTGTGACCCTCGTCGCCGGCCACGCGCTGCTTGCTCCCGCGCCCGCGCCGCGACGCCATTATCACCG
CCGCTGCTAGGACTCCCAGCAGCACCGCTCCGGCCACCGTGGAGCCTGCAACAATCGCCGCCTTCTTGCGGCGACGGCTGCTACTGG
AGGAGCCGCCGCCGGGCGGAGGGAGCGGCGAGAAGGCCGCCCTGGCAGGGGACAGCGGCGCTGCGGCGTCGCAAAGCGTGGGG
AGCGGAGGCCCGCAGAGCCCGCCGCCATTACCGGCGAATGACGACGCGTTAAACCTCGCGGCTAGCACGGGCGGGATGCGGCCGG
AGAGCCGGTTCCCGGAGACATCGAGCACCCGAAGCGTCCGCTGCGAGAACCCCGGGACAGGGCCGGCGAGGAGGTTCCCGTCGAG
CATTAGCGCGGTGAGCCGGGGCGGCGCGTCCGCGAGCTCCTCCGGGATCTCCCCCGTGAGGCGGTTGCCGGAGAGGACGAGGACG
GTGGCGCGGCGGAGCGAGGCGAGGTCCGGCGGGATCTCGCCCGTGAGGCGGTTGCCGGAGAGGTAGAGCAGCTTGAGGTTGGGG
AGCGCGGCGGGGAGGCCCGCCGGGACCGGGCCGCTCAGGGCGTTGTCCTTGAGGCTGAGGACGCGGAGCTCCGGGAACGGGGAG
AGGAGCGCCGCGGTGAGGAAGCCCGTGAGGTTGAGGTTTTCCAGGACGAGCTTCGTCACGCGCCCGCCATGGCCGCACTGGCGGAT
GCCCGGCCAGGAGGTGCAGAGGGTGGAGGCCGTGTCGGGCCGCCATGGGAGGCGGTTGGAGTTGTCCAGGGCGGATTTTAGGGC
TAGCAGCGCTTCCGCTTCGCCGCCGGTGACCGGCGGGGCGAGGTGGTGGAGGAAGAGGAGAATGAGCAGCGGGGAGATGGCTCT
TGGGTGGAGCATGGTGGAGTGAGCTTTGGCGGTAGAGCAAGAGGAGTGAGCAAGTCAGCAGCAATGGCCGGTGGGCGGCATTGA
TTATTTTCTTTGCTTGTTTGTTTGTTGGTGGAAGGACGGATTGAGTGAAGGGAGCTGTAAAAGAGGGAAGAAGAAAAGGGGAAGGA
GGACGAAGCAGAGGCAGAGGAAACCTGAGTCGGGAAAGGAAAGGAAACCGCTTTGAGGTCGCTGCTCAACTAAATGCAGCTGTGT
CCTGTGTGCGTGTGGTGTGGCTGGCGGCGGGAGGAGGAAGAAGAAGAAGAAGAAGAAGAAGACAGCTAGCTAGCCGGGTGG
GATTCAGACGAAGAGCGTCAGTTATTGAGTTCGCAGTTACTGTATTTGTTTCAGACGAAGAGCGTCAGAAACTGATGGGAAAATAAA
CCGACAGCTTTGGTCTTGGTCTGAATATCTGATGGCGATGCAGCAAAGCAAGCAAGCTCGGCTGCTGAGGTGCAGCAATGGCGGCT
AGCGAAGGGAGAAAGAATGGGGAATCAAGTGTTGGAGTCTGAGCTACAGCGAGCTTTCTCTGTGTCGCTTTAGCTAGCTGATGATTT
CTACTCTTGATAACTTGGCGGTAAGCTAAGCTAAAGCAGCATGCACAGCGATTATACCCTCCACTGGGCAGTCAGCGCAGCTCCTCG
GGGTTGTGAGGGAAAAAAAAGAAAAGCCTGTGTCCTGGCCATTTTACTAGTCGCGTATGTGGCCGGCAAAGAGCAGCAGAGGGAA
GCTCTGCTCTAGCCCTTGACTGACAAGCTCCATTATTTAGTCTCCGGTGTTGTTTTTCGATTTGAAAGCTTGTTTTGACGGTACGGAGA
GCGAGCACGGAGTAGTAAATCAATCTTATCTGGGTGAGAGATACTGAGAGGCTCAGTCAATGCCATTACGCACGTCGTAGCACCCTT
TACTTAATGATTAACCATGCTGCTGCTAGTTAACTCGATGGGCTTCGGAGTTGAGACGAGCGAGAGAGAGAAATCCAGATCGTGATG
CAACATGGAGAGTACTACTCCCACTATGTTTATGTATGCCCGTGAGAGAGAGAATGAGATTGGGTCGCAGGAGGAGGAAGAAGGA
GGAGGACGAAATGGGTGAAAGAATTCAATGGTGCCACGTGCATACAGTGCACTGCTGCTCTCTCTCTGCAGATCCAACGCACTTCAA
TAGGACACTGCCTGTCCTATTAACGCCACCAACAAGATGAAGCTATAGAGAGACAGGGAGCTTTTTTTTTCATTTTTTGAGAAACAGT
TTTCCTTCTTTTGCAAAAAGAAAAAACTTTCGATGGGCCTCTCTCTCGCCCGGGGCTTTCTTCTTTGTGGAGCAGGCCTTATTGACTGC
TTCGTGGTGTCCGGCCCAACGGCCCGTATATAACACGAGAACATTCTACCAGGCCCCAAAAGCATTGCCATTGTCCGGCCCAGTGGC
AACGTGGGAACACTAACATATGGGACCCCATGTTACCCTGAGTACGTCATAAAATCTTCCTCCTCCCAATGGAGCCAAAAGATAGTGA
ACCACGATCAGAGTTTTGCTCTTAGACGAACAACTTGCAGCATTCACATATTTCTTTCCAATCTTGTTTTGTTTTGCATGGCCTGCATCT
CGTCTCATGACATCCGCTAAAGATTCAGAGAACATATCCAGTGAAAATGGCTATTGGGTGTAAATCTGCAAATTTCGACCAACAGCCA
TCGGATCCAGAATAGACGGCATGGATGAAACGTGGGAACCAACAGATCACTTAACCCACTTTTACAAATAACCCCTGCTAAAAACAC
CGATCAAGTTTTTCCTTCCAAACTCAAGTTCGACCTTCACAGATGGAGATCCGCCACCCGTCCTCCACCAGCGCCGCCCGCTTGCGCTG
GACCACCGTCGTCCGCCCGCCCTAGAACACCGCTGTTTCCTCGGGCAAGACCACCGCTGTACGCCTGCCCTCGACCACCGCCGTCCAC
CAACCCTTAACCACCTTCGCGCATGTGCCCTGGCACTCGCAAGGCGTCGGATCCCCACGCATGAACGACCAGGAATTTCCCTCACGGG
ACGGAGGCCCGGCAGGCACGACGATGGCCCGCGCAACGGCGACCTGGCATGGCAGTGCCCCAACGCAACGGAGACCCGCTGGCCA
CGGCACTGCTTCATGCGACGGCCACCCGACGGTCTGGGTGAAGCTACGTTGCGACGGCCACCCGACGGTCTGGGTGAAGCTACGTG
GCAACGGCCACCTGTTCCTGTACCTCAACCTGAAGCTGCAGTCTGCTGTTTGGGACAAAATGTTCTGTACCTGAACCTGAATGTTTTGC
TTTACTGTTCTTGAATGGTGGATGTGCAGGCAAATTGATGCTTCTTTGTTGCTGTGTTGTTGTTGTACATACGAAATTGTGGATGTTAA
AGTAGTTTTGTTACATTATGACAATGAATCTGAATTCAGTGTATCATGCAAATGAAAAGTTCAAACAGATAGTCTCCAGTTCATCTATC
TTTCAGTTTTTTTTTCTTCTCTGATATTGAAAAGAAGTTTACCTGAATTTTGTCACTATAGTTGTTCTGAGAACATGTTGAATTCATTTTT
TTAAGCATTCGAGGTGATGCTGATGGCTCTTTTGGTAGGTTGTAGCGGAACAAGAAGGTTGAGGTCACTAGATCTTGATCGTAGTTTT
ACCTCTATGTGGTTTCAGTGGGTATTCTCTCCGGTCTTGTTCATGGCAAGGCCGCAGGGCGCAGGGGACGGAGGAGCAGCAGAGGC
CACCGCTTACGAGTGGCAGCAGTGACACGTGCTCGTGAGGGAGAATCATGGCCGCTGGAGGACAGTGCATCATTTCCGGACCCAGC
TGAGGCCGGTGTCTCACAGCCGCCGGACGGAGTGCGCTGGATGGCGGGCCTCGCGGAGAGCTAGGACACACGCGCGGCGGCCGTT

GAGGATGGGCGGACACCGCTCCGTGGTCCAGGAGAGCAGACGTAAGTGGTCCGGGATGGGCGGCGGGCAGGGGGCATCCGGCGC
GGCCAGCCGGATTCTCCATGGCAGTGAAGGTTGAAGACGAAACGTTTCTGGAAGACTGAAATCGAGAGAAAAACTTGATCGGTGTT
TTTAGTTGGGAGTTATTTGTAAAATCGAGTTTAAGTGACCGGCTCGTTCACACGTTTCATCTGTGCCGTCTATTCTAGATCCGATGGCT
GACGGACGATTTGCGGATTTGCATCCAATAGCCATTTTCACTGGATACGTTGTCAAAGATACATTATATCGTCGCTAGTAGCGATAGC
CTTTGTTTAAGCCTGGTGCGAGTTTGTAATCGAAGGATTATGCTCGCTACTATCTTGTTAAGACATATATCGTCTCTTGGATTGGCATC
GGTCTTAATGAGCTCGGAATCGCTGATCGACACAATCTATGGCAGATCTATAATGTCATTCCGAGATAATAAAAATAGTTGCAACCTA
CATTGATCCCCAAAAAATAGATCAGTATACTGAAACAGTGAGCAAATGGAACACAAGGTATGAATGAAAACAAAGGGTGTGGGGTT
GCCATTTTAGGCTAACTAAGCAGGCTGTCAGGCAGAAAGAAATACTCTTGACAGATTAGCACACCACAGCGAGAGAGAGAGGGGAA
GGGGAAGGAGGGGGAATCCATCGGCTGCGGCGTGGCGATCTCGAGCTGGCGACCCCGCGCTCCTCCCACCACCTCGCCTCCGCCGC
CGCCCTCGCGGCAGCGGGGGTCTCCTACCCGCCGCCGCTGCCCCCCACCACGCCCAGCGCCGCCGCGCCGCCGCACGACCCCGGCGC
CGGCGGCGCCGCCAAGCAGATCGTCGACTCCCTCCTCGCCCGCTTCCTCCCGCTCGCCCGCCGCCGCATCGAGACCGCGCAGGCCCA
GGTGATTCTGCCTGCTCTTCTCCCCCCTAACTCCTCTCCCCCACGTTCCGAATTCCGGCTGATTCCCGTTCCCGCGAGAGGTCAACGAA
AATCGCACCACCTAGGGCTGCCTGTCCGCATCGCGTCCATGTAGATTCGGGGGAGCCTCGCTTAGGGCACGCTGATGGGGTTCAATT
TAAGAGGCACCTTCTACTTTGCTCAAGCACACGTCAATTCAATTAGGTTAGAGGTCTGACCAATGCTCCAAGAAATCGCCTGCCCGCC
TGCCCATGGTGCTACATTTCATTTGCTCACTGCCACGCACCCACCAATTTGGAA

Alignment of Sequence_1: [Untitled Sequence #1] with Sequence_2: [Sequence Window #2]

Similarity : 4391/6172 (71.14 %)

```
Seq_1  1     GCTTCTCCTCCGCGCAGCCGACGCCGCCATTGGACGCGGCGGACGAGTCCCCGCCATTGG  60

Seq_2  1     ------------------------------------------------------------  0


Seq_1  61    ACTCCGTGGTGGTCTCCTCGTCGCGGGCGGCGCGGACCCAGGCCGGGATGTCGTCGGCCT  120

Seq_2  1     ------------------------------------------------------------  0


Seq_1  121   GGAGCATGTCCTGGAACGGCGCCCGGCCCGTCAGCAGCTCCAGCAGCAGCACCCCGAAGC  180

Seq_2  1     ------------------------------------------------------------  0


Seq_1  181   TGTACACGTCCGACGCCGCCGTGAAGAGCCCACCGCCGGCGGAGCGGGTCTCCGGGGCGC  240

Seq_2  1     ------------------------------------------------------------  0


Seq_1  241   GGTAGAGGAGCGAGGCGGCGGCGGCGTCGGCGGAGGAGGGGCTGTGGAGAGCCGGGACGA  300

Seq_2  1     ------------------------------------------------------------  0


Seq_1  301   GGCCGTAGTCGGTGAGGCAGGACTCGAAGTCGGGGCCCAGGAGCACGTTGGACGGCTTCA  360

Seq_2  1     ------------------------------------------------------------  0


Seq_1  361   GGTTGCCGTGCACGATGCCGATGCCGGCAGGAGAAGAGTGCGTGTGCAGGTGCAGGAGGC  420

Seq_2  1     ------------------------------------------------------------  0


Seq_1  421   CGGCGGCGATGTCCTCGGCGATCTTCATGCACGACGTCCAGTGCAGCGGCTTCCCCTTGC  480

Seq_2  1     ------------------------------------------------------------  0


Seq_1  481   TCGACGGCCGAGACCCTGCATCCAACCAACCATGCATCAAGCGCCAACAGTTTCAGAATT  540

Seq_2  1     ------------------------------------------------------------  0
```

```
Seq_1  541   ATTTACACAAGCTGCTTGCTTTAGCACAAGTAAAAAAAATTAGAAGGCGACAGGTGAAAA  600

Seq_2  1     ------------------------------------------------------------  0

Seq_1  601   TTAACGGAGCAGGTGGTACATGGAGGCCTGGTTATTTATTTACTGATTTACAGTCACTGA  660

Seq_2  1     ------------------------------------------------------------  0

Seq_1  661   TCATCATCAGGAGGGCATGCTTTTGCATTGCTAGATGGAGTTGCAGCCATGGCGAAAAAC  720

Seq_2  1     ------------------------------------------------------------  0

Seq_1  721   AGAGCAGGGGAGGTCCTACCATCTCATGGCTTTTTGACTGGCTGGTATAACTAATTCTGG  780

Seq_2  1     ------------------------------------------------------------  0

Seq_1  781   GACAGCTACACAGACTGCACGAGCACCATCATGGTCATGGACCCCGCTACACAATACATT  840

Seq_2  1     ------------------------------------------------------------  0

Seq_1  841   GACACCAAGATAGCTAGGCATGGAGTTGAGAGCATCATGGGTTTCTGAACAGCAGAATTA  900

Seq_2  1     ------------------------------------------------------------  0

Seq_1  901   TGTAGGACAACCAATACTAGATTAAGACAACACCTTCTTTACTTGCAAGAAATAAATTTA  960

Seq_2  1     ------------------------------------------------------------  0

Seq_1  961   TTACTACTGTGACAGTGTGACTCACATGAGCCTCCCCCCATGTTCATGTGTCCAAGGTTC  1020

Seq_2  1     ------------------------------------------------------------  0

Seq_1  1021  AGTCAGAGCTAGTGGTTTATTTATTTTTGGTCCCTAGATTCATGGCAGCGTGTCTCCATG  1080

Seq_2  1     ------------------------------------------------------------  0

Seq_1  1081  AGAACATGTTCCCGGGATCCTCTCCTCTGCGGGCAACTAATGGTGTAAGGTTTCTTGTCG  1140

Seq_2  1     ------------------------------------------------------------  0

Seq_1  1141  ATCTTGTTTGTCATGTATGGGAACCAATACGGGGATTCGCATCAATCGGAGGCCGGAGAT  1200

Seq_2  1     ------------------------------------------------------------  0

Seq_1  1201  TATCCTCATTTCCGGGGGAAAGATGAAGCTAATAAAGCTAATTTTGTTCTCTTCTTGCAC  1260

Seq_2  1     ------------------------------------------------------------  0

Seq_1  1261  TTCACAAATCTCACAACTCTGTGATACGGAGAATTTACCGCTGTTGGTGCTGGATCGATG  1320

Seq_2  1     ------------------------------------------------------------  0

Seq_1  1321  AAGAATTTACCCGTCCAATATAGCACTCAAACAGCACAGTACTGGCTAGAACTTCACTTC  1380

Seq_2  1     ------------------------------------------------------------  0
```

```
Seq_1  1381  ATGTGGGACACTGGGACTCAACCTTATGCATGGAGGATGAATAAAGTTGGCTTTTAAGCT  1440

Seq_2  1     ------------------------------------------------------------  0

Seq_1  1441  AGGGCCATGCCGGGTCTTGTTGTGTTCCATTTTTTCACATGGCTTGGCACTGTACTCCTG  1500

Seq_2  1     ------------------------------------------------------------  0

Seq_1  1501  GTACACATGGTTTGAATTCTTTCATCTGTTTCTGTAAATAAATTCAGAGGTGGACCAAAC  1560

Seq_2  1     ------------------------------------------------------------  0

Seq_1  1561  TAGAGAAGGTACTAGCAACAGCTGGAGCATGTATACATACGTACGTATGTACGGGGTATA  1620

Seq_2  1     ------------------------------------------------------------  0

Seq_1  1621  ATGTACGTGCAGAAGAGCCCAGATTAAGCTTTGGATTTGGAATGTAGCAGCAGCAGCAGC  1680

Seq_2  1     ------------------------------------------------------------  0

Seq_1  1681  AAGCTAATAAGCTCCATTAAGCTATGGAACGGAGGCAACAGAAGAGTCCAAATCAAGAGC  1740
                                                        |||||||||||||||||
Seq_2  1     -------------------------------------------GAGTCCAAATCAAGAGC  17

Seq_1  1741  CCTTCTTCTGCTTCTTCCTTGCAGACCCGGCCTTTTTGTTTGCAGTGATTGCAATGCACG  1800
             ||||||||||||||||||||||||||||||||||||||||||||||||||||||||||||
Seq_2  18    CCTTCTTCTGCTTCTTCCTTGCAGACCCGGCCTTTTTGTTTGCAGTGATTGCAATGCACG  77

Seq_1  1801  TACGGCAGATGACAGATCCATATATGCTGCTAGATACTCCCCAGCAAGAAAGACAGGCAA  1860
             ||||||||||||||||||||||||||||||||||||||||||||||||||||||||||||
Seq_2  78    TACGGCAGATGACAGATCCATATATGCTGCTAGATACTCCCCAGCAAGAAAGACAGGCAA  137

Seq_1  1861  ACATATATGCAATGCAAATGCAGCTTTAACTTATGCATTGCTCTATACCGTGACACGAAG  1920
             ||||||||||||||||||||||||||||||||||||||||||||||||||||||||||||
Seq_2  138   ACATATATGCAATGCAAATGCAGCTTTAACTTATGCATTGCTCTATACCGTGACACGAAG  197

Seq_1  1921  AAATTTAAATTTTCACCCCCTTTTCGTGATCCGATTTGACATTTTGATTCGTACGAACGA  1980
             ||||||||||||||||||||||||||||||||||||||||||||||||||||||||||||
Seq_2  198   AAATTTAAATTTTCACCCCCTTTTCGTGATCCGATTTGACATTTTGATTCGTACGAACGA  257

Seq_1  1981  GAGCACGGGCAGGGAGGATCAGCTGCCTGCCAGCACTTAATCAGCAGGACTTGTTCGTGT  2040
             ||||||||||||||||||||||||||||||||||||||||||||||||||||||||||||
Seq_2  258   GAGCACGGGCAGGGAGGATCAGCTGCCTGCCAGCACTTAATCAGCAGGACTTGTTCGTGT  317

Seq_1  2041  GTGTCGACATGAATGGAATGGAAATTAAATCAAGCCGTGTGGATTAGCAGTAGGAATTTC  2100
             ||||||||||||||||||||||||||||||||||||||||||||||||||||||||||||
Seq_2  318   GTGTCGACATGAATGGAATGGAAATTAAATCAAGCCGTGTGGATTAGCAGTAGGAATTTC  377

Seq_1  2101  CGCATTTGGTTCGTAACAATTAAGGAGGAAGAAGAAGGCAATGGCAATGGAGACATGGAG  2160
             ||||||||||||||||||||||||||||||||||||||||||||||||||||||||||||
Seq_2  378   CGCATTTGGTTCGTAACAATTAAGGAGGAAGAAGAAGGCAATGGCAATGGAGACATGGAG  437

Seq_1  2161  TACTGACCGTGGATGAGGGAGAAGAGGCTGCCGTTGGGGTAGTAGTCGTAGACGAGGAGC  2220
             ||||||||||||||||||||||||||||||||||||||||||||||||||||||||||||
Seq_2  438   TACTGACCGTGGATGAGGGAGAAGAGGCTGCCGTTGGGGTAGTAGTCGTAGACGAGGAGC  497
```

```
Seq_1  2221  CGCTCCTCCTTGGCCTGGAAGTAGGCCCGGAGCGCCACCACGTTGGGGTGCCGCACCCGC  2280
             ||||||||||||||||||||||||||||||||||||||||||||||||||||||||||||
Seq_2  498   CGCTCCTCCTTGGCCTGGAAGTAGGCCCGGAGCGCCACCACGTTGGGGTGCCGCACCCGC  557

Seq_1  2281  CCCAGCTCCTCCGCGCGCCGCCCGAACCCCGCCGCGTCGCCATGGCCGTCGCCGCCGCCG  2340
             ||||||||||||||||||||||||||||||||||||||||||||||||||||||||||||
Seq_2  558   CCCAGCTCCTCCGCGCGCCGCCCGAACCCCGCCGCGTCGCCATGGCCGTCGCCGCCGCCG  617

Seq_1  2341  CAACGCATCCGCTTCACCGTCACGATGAACCCCGTCTCCATCACCGCCTTGTACGTGCTC  2400
             ||||||||||||||||||||||||||||||||||||||||||||||||||||||||||||
Seq_2  618   CAACGCATCCGCTTCACCGTCACGATGAACCCCGTCTCCATCACCGCCTTGTACGTGCTC  677

Seq_1  2401  CCGGCTTCCCCTCTCCCCAGCGTCTCCGCCGACGCCCGCAGCAGCTCCTCCAGGCTGTAC  2460
             ||||||||||||||||||||||||||||||||||||||||||||||||||||||||||||
Seq_2  678   CCGGCTTCCCCTCTCCCCAGCGTCTCCGCCGACGCCCGCAGCAGCTCCTCCAGGCTGTAC  737

Seq_1  2461  ATCCCGCCGCCGCCGCAGAACACCAGCTTCCCCATCCCCCCCTCCCGCTCCCACGAGAAC  2520
             ||||||||||||||||||||||||||||||||||||||||||||||||||||||||||||
Seq_2  738   ATCCCGCCGCCGCCGCAGAACACCAGCTTCCCCATCCCCCCCTCCCGCTCCCACGAGAAC  797

Seq_1  2521  TCCCTGGCCGCCATTGCTGCTGATGGTGCTGCTGGTGGCGGCAATGGCGCGCTCGCCGCC  2580
             ||||||||||||||||||||||||||||||||||||||||||||||||||||||||||||
Seq_2  798   TCCCTGGCCGCCATTGCTGCTGATGGTGCTGCTGGTGGCGGCAATGGCGCGCTCGCCGCC  857

Seq_1  2581  GGCTGCTCCGACGATGAAGGAATTGCTTCGGCCTTGTTGTTGTGACCCTCGTCGCCGGCC  2640
             ||||||||||||||||||||||||||||||||||||||||||||||||||||||||||||
Seq_2  858   GGCTGCTCCGACGATGAAGGAATTGCTTCGGCCTTGTTGTTGTGACCCTCGTCGCCGGCC  917

Seq_1  2641  ACGCGCTGCTTGCTCCCGCGCCCGCGCCGCGACGCCATTATCACCGCCGCTGCTAGGACT  2700
             ||||||||||||||||||||||||||||||||||||||||||||||||||||||||||||
Seq_2  918   ACGCGCTGCTTGCTCCCGCGCCCGCGCCGCGACGCCATTATCACCGCCGCTGCTAGGACT  977

Seq_1  2701  CCCAGCAGCACCGCTCCGGCCACCGTGGAGCCTGCAACAATCGCCGCCTTCTTGCGGCGA  2760
             ||||||||||||||||||||||||||||||||||||||||||||||||||||||||||||
Seq_2  978   CCCAGCAGCACCGCTCCGGCCACCGTGGAGCCTGCAACAATCGCCGCCTTCTTGCGGCGA  1037

Seq_1  2761  CGGCTGCTACTGGAGGAGCCGCCGCCGGGCGGAGGGAGCGGCGAGAAGGCCGCCCTGGCA  2820
             ||||||||||||||||||||||||||||||||||||||||||||||||||||||||||||
Seq_2  1038  CGGCTGCTACTGGAGGAGCCGCCGCCGGGCGGAGGGAGCGGCGAGAAGGCCGCCCTGGCA  1097

Seq_1  2821  GGGGACAGCGGCGCTGCGGCGTCGCAAAGCGTGGGGAGCGGAGGCCCGCAGAGCCCGCCG  2880
             ||||||||||||||||||||||||||||||||||||||||||||||||||||||||||||
Seq_2  1098  GGGGACAGCGGCGCTGCGGCGTCGCAAAGCGTGGGGAGCGGAGGCCCGCAGAGCCCGCCG  1157

Seq_1  2881  CCATTACCGGCGAATGACGACGCGTTAAACCTCGCGGCTAGCACGGGCGGGATGCGGCCG  2940
             ||||||||||||||||||||||||||||||||||||||||||||||||||||||||||||
Seq_2  1158  CCATTACCGGCGAATGACGACGCGTTAAACCTCGCGGCTAGCACGGGCGGGATGCGGCCG  1217

Seq_1  2941  GAGAGCCGGTTCCCGGAGACATCGAGCACCCGAAGCGTCCGCTGCGAGAACCCCGGGACA  3000
             ||||||||||||||||||||||||||||||||||||||||||||||||||||||||||||
Seq_2  1218  GAGAGCCGGTTCCCGGAGACATCGAGCACCCGAAGCGTCCGCTGCGAGAACCCCGGGACA  1277

Seq_1  3001  GGGCCGGCGAGGAGGTTCCCGTCGAGCATTAGCGCGGTGAGCCGGGGCGGCGCGTCCGCG  3060
             ||||||||||||||||||||||||||||||||||||||||||||||||||||||||||||
Seq_2  1278  GGGCCGGCGAGGAGGTTCCCGTCGAGCATTAGCGCGGTGAGCCGGGGCGGCGCGTCCGCG  1337
```

```
Seq_1  3061  AGCTCCTCCGGGATCTCCCCCGTGAGGCGGTTGCCGGAGAGGACGAGGACGGTGGCGCGG  3120
             ||||||||||||||||||||||||||||||||||||||||||||||||||||||||||||
Seq_2  1338  AGCTCCTCCGGGATCTCCCCCGTGAGGCGGTTGCCGGAGAGGACGAGGACGGTGGCGCGG  1397

Seq_1  3121  CGGAGCGAGGCGAGGTCCGGCGGGATCTCGCCCGTGAGGCGGTTGCCGGAGAGGTAGAGC  3180
             ||||||||||||||||||||||||||||||||||||||||||||||||||||||||||||
Seq_2  1398  CGGAGCGAGGCGAGGTCCGGCGGGATCTCGCCCGTGAGGCGGTTGCCGGAGAGGTAGAGC  1457

Seq_1  3181  AGCTTGAGGTTGGGGAGCGCGGCGGGGAGGCCCGCCGGGACCGGGCCGCTCAGGGCGTTG  3240
             ||||||||||||||||||||||||||||||||||||||||||||||||||||||||||||
Seq_2  1458  AGCTTGAGGTTGGGGAGCGCGGCGGGGAGGCCCGCCGGGACCGGGCCGCTCAGGGCGTTG  1517

Seq_1  3241  TCCTTGAGGCTGAGGACGCGGAGCTCCGGGAACGGGGAGAGGAGCGCCGCGGTGAGGAAG  3300
             ||||||||||||||||||||||||||||||||||||||||||||||||||||||||||||
Seq_2  1518  TCCTTGAGGCTGAGGACGCGGAGCTCCGGGAACGGGGAGAGGAGCGCCGCGGTGAGGAAG  1577

Seq_1  3301  CCCGTGAGGTTGAGGTTTTCCAGGACGAGCTTCGTCACGCGCCCGCCATGGCCGCACTGG  3360
             ||||||||||||||||||||||||||||||||||||||||||||||||||||||||||||
Seq_2  1578  CCCGTGAGGTTGAGGTTTTCCAGGACGAGCTTCGTCACGCGCCCGCCATGGCCGCACTGG  1637

Seq_1  3361  CGGATGCCCGGCCAGGAGGTGCAGAGGGTGGAGGCCGTGTCGGGCCGCCATGGGAGGCGG  3420
             ||||||||||||||||||||||||||||||||||||||||||||||||||||||||||||
Seq_2  1638  CGGATGCCCGGCCAGGAGGTGCAGAGGGTGGAGGCCGTGTCGGGCCGCCATGGGAGGCGG  1697

Seq_1  3421  TTGGAGTTGTCCAGGGCGGATTTTAGGGCTAGCAGCGCTTCCGCTTCGCCGCCGGTGACC  3480
             ||||||||||||||||||||||||||||||||||||||||||||||||||||||||||||
Seq_2  1698  TTGGAGTTGTCCAGGGCGGATTTTAGGGCTAGCAGCGCTTCCGCTTCGCCGCCGGTGACC  1757

Seq_1  3481  GGCGGGGCGAGGTGGTGGAGGAAGAGGAGAATGAGCAGCGGGGAGATGGCTCTTGGGTGG  3540
             ||||||||||||||||||||||||||||||||||||||||||||||||||||||||||||
Seq_2  1758  GGCGGGGCGAGGTGGTGGAGGAAGAGGAGAATGAGCAGCGGGGAGATGGCTCTTGGGTGG  1817

Seq_1  3541  AGCATGGTGGAGTGAGCTTTGGCGGTAGAGCAAGAGGAGTGAGCAAGTCAGCAGCAATGG  3600
             ||||||||||||||||||||||||||||||||||||||||||||||||||||||||||||
Seq_2  1818  AGCATGGTGGAGTGAGCTTTGGCGGTAGAGCAAGAGGAGTGAGCAAGTCAGCAGCAATGG  1877

Seq_1  3601  CCGGTGGGCGGCATTGATTATTTTCTTTGCTTGTTTGTTTGTTGGTGGAAGGACGGATTG  3660
             ||||||||||||||||||||||||||||||||||||||||||||||||||||||||||||
Seq_2  1878  CCGGTGGGCGGCATTGATTATTTTCTTTGCTTGTTTGTTTGTTGGTGGAAGGACGGATTG  1937

Seq_1  3661  AGTGAAGGGAGCTGTAAAAGAGGGAAGAAGAAAAGGGGAAGGAGGACGAAGCAGAGGCAG  3720
             ||||||||||||||||||||||||||||||||||||||||||||||||||||||||||||
Seq_2  1938  AGTGAAGGGAGCTGTAAAAGAGGGAAGAAGAAAAGGGGAAGGAGGACGAAGCAGAGGCAG  1997

Seq_1  3721  AGGAAACCTGAGTCGGGAAAGGAAAGGAAACCGCTTTGAGGTCGCTGCTCAACTAAATGC  3780
             ||||||||||||||||||||||||||||||||||||||||||||||||||||||||||||
Seq_2  1998  AGGAAACCTGAGTCGGGAAAGGAAAGGAAACCGCTTTGAGGTCGCTGCTCAACTAAATGC  2057

Seq_1  3781  AGCTGTGTCCTGTGTGCGTGTGGTGTGGCTGGCGGCGGGAGGAGGAAGAAGAAGAAGAAG  3840
             ||||||||||||||||||||||||||||||||||||||||||||||||||||||||||||
Seq_2  2058  AGCTGTGTCCTGTGTGCGTGTGGTGTGGCTGGCGGCGGGAGGAGGAAGAAGAAGAAGAAG  2117

Seq_1  3841  AAGAAGAAGA---CAGCTAGCTAGCCGGGTGGGATTCAGACGAAGAGCGTCAGTTATTGA  3897
             ||||||||||   |||||||||||||||||||||||||||||||||||||||||||||||
Seq_2  2118  AAGAAGAAGAAGACAGCTAGCTAGCCGGGTGGGATTCAGACGAAGAGCGTCAGTTATTGA  2177
```

```
Seq_1  3898  GTTCGCAGTTACTGTATTTGTTTCAGACGAAGAGCGTCAGAAACTGATGGGAAAATAAAC  3957
             ||||||||||||||||||||||||||||||||||||||||||||||||||||||||||||
Seq_2  2178  GTTCGCAGTTACTGTATTTGTTTCAGACGAAGAGCGTCAGAAACTGATGGGAAAATAAAC  2237

Seq_1  3958  CGACAGCTTTGGTCTTGGTCTGAATATCTGATGGCGATGCAGCAAAGCAAGCAAGCTCGG  4017
             ||||||||||||||||||||||||||||||||||||||||||||||||||||||||||||
Seq_2  2238  CGACAGCTTTGGTCTTGGTCTGAATATCTGATGGCGATGCAGCAAAGCAAGCAAGCTCGG  2297

Seq_1  4018  CTGCTGAGGTGCAGCAATGGCGGCTAGCGAAGGGAGAAAGAATGGGGAATCAAGTGTTGG  4077
             ||||||||||||||||||||||||||||||||||||||||||||||||||||||||||||
Seq_2  2298  CTGCTGAGGTGCAGCAATGGCGGCTAGCGAAGGGAGAAAGAATGGGGAATCAAGTGTTGG  2357

Seq_1  4078  AGTCTGAGCTACAGCGAGCTTTCTCTGTGTCGCTTTAGCTAGCTGATGATTTCTACTCTT  4137
             ||||||||||||||||||||||||||||||||||||||||||||||||||||||||||||
Seq_2  2358  AGTCTGAGCTACAGCGAGCTTTCTCTGTGTCGCTTTAGCTAGCTGATGATTTCTACTCTT  2417

Seq_1  4138  GATAACTTGGCGGTAAGCTAAGCTAAAGCAGCATGCACAGCGATTATACCCTCCAGTGGG  4197
             ||||||||||||||||||||||||||||||||||||||||||||||||||||||| ||||
Seq_2  2418  GATAACTTGGCGGTAAGCTAAGCTAAAGCAGCATGCACAGCGATTATACCCTCCACTGGG  2477

Seq_1  4198  CAGTCAGCGCAGCTCCTCGGGGTCGTGAGGGAAAAAAAAGAAAAGCCTGTGTCCTGGCCA  4257
             ||||||||||||||||||||||| ||||||||||||||||||||||||||||||||||||
Seq_2  2478  CAGTCAGCGCAGCTCCTCGGGGTTGTGAGGGAAAAAAAAGAAAAGCCTGTGTCCTGGCCA  2537

Seq_1  4258  TTTTACTAGTCGCGTATGTGGCCGGCAAAGAGCAGCAGAGGGAAGGATTACTATCATCCC  4317
             |||||||||||||||||||||||||||||||||||||||||||||
Seq_2  2538  TTTTACTAGTCGCGTATGTGGCCGGCAAAGAGCAGCAGAGGGAAG---------------  2582

Seq_1  4318  TTCTCTGCTCTAGCCCTTGACTGACAAGCTCCATTATTTAGTCTCCGGTGTTGTTTTTCG  4377
               ||||||||||||||||||||||||||||||||||||||||||||||||||||||||||
Seq_2  2583  --CTCTGCTCTAGCCCTTGACTGACAAGCTCCATTATTTAGTCTCCGGTGTTGTTTTTCG  2640

Seq_1  4378  ATTTGAAAGCTTGTTTTGACGGTACGGAGAGCGAGCACGGAGTAGTAAATCAATCTTATC  4437
             ||||||||||||||||||||||||||||||||||||||||||||||||||||||||||||
Seq_2  2641  ATTTGAAAGCTTGTTTTGACGGTACGGAGAGCGAGCACGGAGTAGTAAATCAATCTTATC  2700

Seq_1  4438  TGGGTGAGAGATACTGAGATGCTCAGTCAATGCCATTACGCACGTCGTAGCACCCTTTAC  4497
             ||||||||||||||||||| ||||||||||||||||||||||||||||||||||||||||
Seq_2  2701  TGGGTGAGAGATACTGAGAGGCTCAGTCAATGCCATTACGCACGTCGTAGCACCCTTTAC  2760

Seq_1  4498  TTAATGATTAACCATGCTGCTGCTAGTTAACTCGATGGGCTTCGGAGTTGAGACGAGCGA  4557
             ||||||||||||||||||||||||||||||||||||||||||||||||||||||||||||
Seq_2  2761  TTAATGATTAACCATGCTGCTGCTAGTTAACTCGATGGGCTTCGGAGTTGAGACGAGCGA  2820

Seq_1  4558  GAGAGAGAAATCCAGATCGTGATGCAACATGGAGAGTACTACTCCCACTATGTTTATGTA  4617
             ||||||||||||||||||||||||||||||||||||||||||||||||||||||||||||
Seq_2  2821  GAGAGAGAAATCCAGATCGTGATGCAACATGGAGAGTACTACTCCCACTATGTTTATGTA  2880

Seq_1  4618  TGCCCGTGAGAGAGAGAATGAGATTGGGTCGCAGGAGGAGGAAGAAGAAGGAGGACGAAA  4677
             ||||||||||||||||||||||||||||||||||||||||||||||| ||||||||||||
Seq_2  2881  TGCCCGTGAGAGAGAGAATGAGATTGGGTCGCAGGAGGAGGAAGAAGGAGGAGGACGAAA  2940

Seq_1  4678  TGGGTGAAAGAATTCAATGGTGCCACGTGCATACAGTGCACTGCTGCTCTCTCTCTCTGC  4737
             ||||||||||||||||||||||||||||||||||||||||||||||||||||||||  ||
Seq_2  2941  TGGGTGAAAGAATTCAATGGTGCCACGTGCATACAGTGCACTGCTGCTCTCTCTCT--GC  2998
```

```
Seq_1  4738  AGATCCAACGCACTTCAATAGGACACTGCCTGTACTATTAACGCCACCAACAAGATGAAG  4797
             ||||||||||||||||||||||||||||||||| ||||||||||||||||||||||||||
Seq_2  2999  AGATCCAACGCACTTCAATAGGACACTGCCTGTCCTATTAACGCCACCAACAAGATGAAG  3058

Seq_1  4798  CTATAGAG----------------------TTTTTTGAGAAACAGTTTTCCTTCTTTTGC  4835
             ||||||||                      ||||||||||||||||||||||||||||||
Seq_2  3059  CTATAGAGAGACAGGGAGCTTTTTTTTTCATTTTTTGAGAAACAGTTTTCCTTCTTTTGC  3118

Seq_1  4836  AAAAAGAAAAAACTTTCGATGGGCCTCTCTCTCGCCCGGGGCTTTCTTCTTTGTGGAGCA  4895
             ||||||||||||||||||||||||||||||||||||||||||||||||||||||||||||
Seq_2  3119  AAAAAGAAAAAACTTTCGATGGGCCTCTCTCTCGCCCGGGGCTTTCTTCTTTGTGGAGCA  3178

Seq_1  4896  GGCCTTATTGACTGCTTCGTGGTGTCCGGCCCAACGGCCCGTATATAACACGAGAACATT  4955
             ||||||||||||||||||||||||||||||||||||||||||||||||||||||||||||
Seq_2  3179  GGCCTTATTGACTGCTTCGTGGTGTCCGGCCCAACGGCCCGTATATAACACGAGAACATT  3238

Seq_1  4956  CTACCAGGCCCCAAAAGCATTGCCATTGTCCGGCCCAGTGGCAACGTGCGAACACTAACA  5015
             |||||||||||||||||||||||||||||||||||||||||||||||| |||||||||||
Seq_2  3239  CTACCAGGCCCCAAAAGCATTGCCATTGTCCGGCCCAGTGGCAACGTGGGAACACTAACA  3298

Seq_1  5016  TATGGGACCCCATGTTACCCTGAGTACGTCATAAAATCTTCCTCCTCCCAATGGAGCCAA  5075
             ||||||||||||||||||||||||||||||||||||||||||||||||||||||||||||
Seq_2  3299  TATGGGACCCCATGTTACCCTGAGTACGTCATAAAATCTTCCTCCTCCCAATGGAGCCAA  3358

Seq_1  5076  AAGATAGTGAACCACGATCAGAGTTTTGCTCTTAGACGAACAACTTGCAGCGTTCACATA  5135
             ||||||||||||||||||||||||||||||||||||||||||||||||||| ||||||||
Seq_2  3359  AAGATAGTGAACCACGATCAGAGTTTTGCTCTTAGACGAACAACTTGCAGCATTCACATA  3418

Seq_1  5136  TTTCTTTCCTCCTTGTCGCTCATCTCCAATCTTGTTTTGTTTTGCATGGCCTGCATCTCA  5195
             |||||||||                  |||||||||||||||||||||||||||||||| 
Seq_2  3419  TTTCTTTCC------------------AATCTTGTTTTGTTTTGCATGGCCTGCATCTCG  3460

Seq_1  5196  TCTCATGACGTCCGCTGAAGATC-------------------------------------  5218
             ||||||||| |||||| |||||
Seq_2  3461  TCTCATGACATCCGCTAAAGATTCAGAGAACATATCCAGTGAAAATGGCTATTGGGTGTA  3520

Seq_1  5219  ------------------------------------------------------------  5218

Seq_2  3521  AATCTGCAAATTTCGACCAACAGCCATCGGATCCAGAATAGACGGCATGGATGAAACGTG  3580

Seq_1  5219  ------------------------------------------------------------  5218

Seq_2  3581  GGAACCAACAGATCACTTAACCCACTTTTACAAATAACCCCTGCTAAAAACACCGATCAA  3640

Seq_1  5219  ------------------------------------------------------------  5218

Seq_2  3641  GTTTTTCCTTCCAAACTCAAGTTCGACCTTCACAGATGGAGATCCGCCACCCGTCCTCCA  3700

Seq_1  5219  ------------------------------------------------------------  5218

Seq_2  3701  CCAGCGCCGCCCGCTTGCGCTGGACCACCGTCGTCCGCCCGCCCTAGAACACCGCTGTTT  3760

Seq_1  5219  ------------------------------------------------------------  5218

Seq_2  3761  CCTCGGGCAAGACCACCGCTGTACGCCTGCCCTCGACCACCGCCGTCCACCAACCCTTAA  3820
```

```
Seq_1  5219  ------------------------------------------------------------  5218

Seq_2  3821  CCACCTTCGCGCATGTGCCCTGGCACTCGCAAGGCGTCGGATCCCCACGCATGAACGACC  3880

Seq_1  5219  ------------------------------------------------------------  5218

Seq_2  3881  AGGAATTTCCCTCACGGGACGGAGGCCCGGCAGGCACGACGATGGCCCGCGCAACGGCGA  3940

Seq_1  5219  ------------------------------------------------------------  5218

Seq_2  3941  CCTGGCATGGCAGTGCCCCAACGCAACGGAGACCCGCTGGCCACGGCACTGCTTCATGCG  4000

Seq_1  5219  ------------------------------------------------------------  5218

Seq_2  4001  ACGGCCACCCGACGGTCTGGGTGAAGCTACGTTGCGACGGCCACCCGACGGTCTGGGTGA  4060

Seq_1  5219  ------------------------------------------------------------  5218

Seq_2  4061  AGCTACGTGGCAACGGCCACCTGTTCCTGTACCTCAACCTGAAGCTGCAGTCTGCTGTTT  4120

Seq_1  5219  ------------------------------------------------------------  5218

Seq_2  4121  GGGACAAAATGTTCTGTACCTGAACCTGAATGTTTTGCTTTACTGTTCTTGAATGGTGGA  4180

Seq_1  5219  ------------------------------------------------------------  5218

Seq_2  4181  TGTGCAGGCAAATTGATGCTTCTTTGTTGCTGTGTTGTTGTTGTACATACGAAATTGTGG  4240

Seq_1  5219  ------------------------------------------------------------  5218

Seq_2  4241  ATGTTAAAGTAGTTTTGTTACATTATGACAATGAATCTGAATTCAGTGTATCATGCAAAT  4300

Seq_1  5219  ------------------------------------------------------------  5218

Seq_2  4301  GAAAAGTTCAAACAGATAGTCTCCAGTTCATCTATCTTTCAGTTTTTTTTTCTTCTCTGA  4360

Seq_1  5219  ------------------------------------------------------------  5218

Seq_2  4361  TATTGAAAAGAAGTTTACCTGAATTTTGTCACTATAGTTGTTCTGAGAACATGTTGAATT  4420

Seq_1  5219  ------------------------------------------------------------  5218

Seq_2  4421  CATTTTTTTAAGCATTCGAGGTGATGCTGATGGCTCTTTTGGTAGGTTGTAGCGGAACAA  4480

Seq_1  5219  ------------------------------------------------------------  5218

Seq_2  4481  GAAGGTTGAGGTCACTAGATCTTGATCGTAGTTTTACCTCTATGTGGTTTCAGTGGGTAT  4540

Seq_1  5219  ------------------------------------------------------------  5218

Seq_2  4541  TCTCTCCGGTCTTGTTCATGGCAAGGCCGCAGGGCGCAGGGGACGGAGGAGCAGCAGAGG  4600

Seq_1  5219  ------------------------------------------------------------  5218

Seq_2  4601  CCACCGCTTACGAGTGGCAGCAGTGACACGTGCTCGTGAGGGAGAATCATGGCCGCTGGA  4660
```

```
Seq_1  5219  ------------------------------------------------------------  5218

Seq_2  4661  GGACAGTGCATCATTTCCGGACCCAGCTGAGGCCGGTGTCTCACAGCCGCCGGACGGAGT  4720


Seq_1  5219  ------------------------------------------------------------  5218

Seq_2  4721  GCGCTGGATGGCGGGCCTCGCGGAGAGCTAGGACACACGCGCGGCGGCCGTTGAGGATGG  4780


Seq_1  5219  ------------------------------------------------------------  5218

Seq_2  4781  GCGGACACCGCTCCGTGGTCCAGGAGAGCAGACGTAAGTGGTCCGGGATGGGCGGCGGGC  4840


Seq_1  5219  ------------------------------------------------------------  5218

Seq_2  4841  AGGGGGCATCCGGCGCGGCCAGCCGGATTCTCCATGGCAGTGAAGGTTGAAGACGAAACG  4900


Seq_1  5219  ------------------------------------------------------------  5218

Seq_2  4901  TTTCTGGAAGACTGAAATCGAGAGAAAAACTTGATCGGTGTTTTTAGTTGGGAGTTATTT  4960


Seq_1  5219  ------------------------------------------------------------  5218

Seq_2  4961  GTAAAATCGAGTTTAAGTGACCGGCTCGTTCACACGTTTCATCTGTGCCGTCTATTCTAG  5020


Seq_1  5219  ------------------------------------------------------------  5218

Seq_2  5021  ATCCGATGGCTGACGGACGATTTGCGGATTTGCATCCAATAGCCATTTTCACTGGATACG  5080


Seq_1  5219  ------------CATTATATCGTCGCTAGTAGTGATAGCCTTTGTTTAAGCCTGGTGCGA  5266
                         |||||||||||||||||||| |||||||||||||||||||||||||||
Seq_2  5081  TTGTCAAAGATACATTATATCGTCGCTAGTAGCGATAGCCTTTGTTTAAGCCTGGTGCGA  5140


Seq_1  5267  GTTTGTAATCGAAGGATTATGCTCACTAGTATCTTGTTAAGACATATATCGTCTCTTGGA  5326
             |||||||||||||||||||||||| ||| |||||||||||||||||||||||||||||||
Seq_2  5141  GTTTGTAATCGAAGGATTATGCTCGCTACTATCTTGTTAAGACATATATCGTCTCTTGGA  5200


Seq_1  5327  TTGGCATCGGTCTTAATGAGCTCGGAATCGCTGATCGACACAATCTATGGCAGATCTATA  5386
             ||||||||||||||||||||||||||||||||||||||||||||||||||||||||||||
Seq_2  5201  TTGGCATCGGTCTTAATGAGCTCGGAATCGCTGATCGACACAATCTATGGCAGATCTATA  5260


Seq_1  5387  ATGTTATTCCGAGATAATAAAAATAGTTGCAACCTACATTGATCCCCAAAAAATAGATCA  5446
             |||| |||||||||||||||||||||||||||||||||||||||||||||||||||||||
Seq_2  5261  ATGTCATTCCGAGATAATAAAAATAGTTGCAACCTACATTGATCCCCAAAAAATAGATCA  5320


Seq_1  5447  GTATACTGAAACAGTGAGCAAATGGAACACAAGGTATGAATGAAAACAAAGGGTGTGGGG  5506
             ||||||||||||||||||||||||||||||||||||||||||||||||||||||||||||
Seq_2  5321  GTATACTGAAACAGTGAGCAAATGGAACACAAGGTATGAATGAAAACAAAGGGTGTGGGG  5380


Seq_1  5507  TTGCCATTTTAGGCTAACTAAGCAGGCTGTCAGGCAGAAAGAAATACTCTTGACAGATTA  5566
             ||||||||||||||||||||||||||||||||||||||||||||||||||||||||||||
Seq_2  5381  TTGCCATTTTAGGCTAACTAAGCAGGCTGTCAGGCAGAAAGAAATACTCTTGACAGATTA  5440


Seq_1  5567  GCACACCACAGCGAGAGAGAGAGGGGAAGGGGAAGGAGGGGGAACCCATCGGCTGCGGCG  5626
             |||||||||||||||||||||||||||||||||||||||||||| |||||||||||||||
Seq_2  5441  GCACACCACAGCGAGAGAGAGAGGGGAAGGGGAAGGAGGGGGAATCCATCGGCTGCGGCG  5500
```

```
Seq_1  5627  TGGCGATCTCGAGCTGGCGACCCCGCGCTCCTCCCACCACCTCGCCTCCGCCGCCGCCCT  5686
             ||||||||||||||||||||||||||||||||||||||||||||||||||||||||||||
Seq_2  5501  TGGCGATCTCGAGCTGGCGACCCCGCGCTCCTCCCACCACCTCGCCTCCGCCGCCGCCCT  5560

Seq_1  5687  CGCGGCAGCGGGGGTCTCCTACCCGCCGCCGCTGCCCCCCACCACGCCCAGCGCCGCCGC  5746
             ||||||||||||||||||||||||||||||||||||||||||||||||||||||||||||
Seq_2  5561  CGCGGCAGCGGGGGTCTCCTACCCGCCGCCGCTGCCCCCCACCACGCCCAGCGCCGCCGC  5620

Seq_1  5747  GCCGCCGCACGACCCCGGCGCCGGCGGCGCCGCCAAGCAGATCGTCGACTCCCTCCTCGC  5806
             ||||||||||||||||||||||||||||||||||||||||||||||||||||||||||||
Seq_2  5621  GCCGCCGCACGACCCCGGCGCCGGCGGCGCCGCCAAGCAGATCGTCGACTCCCTCCTCGC  5680

Seq_1  5807  CCGCTTCCTCCCGCTCGCCCGCCGCCGCATCGAGACCGCGCAGGCCCAGGTGATTCTGCC  5866
             ||||||||||||||||||||||||||||||||||||||||||||||||||||||||||||
Seq_2  5681  CCGCTTCCTCCCGCTCGCCCGCCGCCGCATCGAGACCGCGCAGGCCCAGGTGATTCTGCC  5740

Seq_1  5867  TGCTCCTCTCCCCCCCAACTCCTCTCCCCCACGTTCCGAATTCCGGCTGATTCCCGTTCC  5926
             ||||| ||||||||| ||||||||||||||||||||||||||||||||||||||||||||
Seq_2  5741  TGCTCTTCTCCCCCCTAACTCCTCTCCCCCACGTTCCGAATTCCGGCTGATTCCCGTTCC  5800

Seq_1  5927  CGCGAGAGGTCAACGAAAATCGCACCACCTAGGGCTGCCTGTCCGCATCGCGTCCATGTA  5986
             ||||||||||||||||||||||||||||||||||||||||||||||||||||||||||||
Seq_2  5801  CGCGAGAGGTCAACGAAAATCGCACCACCTAGGGCTGCCTGTCCGCATCGCGTCCATGTA  5860

Seq_1  5987  GATTCGGGGGAGCATCGCTTAGGGCACGCTGGTGGGGTTCAATTTAAGAGGCACCTTCTA  6046
             ||||||||||||| ||||||||||||||||| ||||||||||||||||||||||||||||
Seq_2  5861  GATTCGGGGGAGCCTCGCTTAGGGCACGCTGATGGGGTTCAATTTAAGAGGCACCTTCTA  5920

Seq_1  6047  CTTTGCTCAAGCACACGTCAATTCAATTAGGTTAGAGGTCTGACCAATGCTCCAAGATAT  6106
             ||||||||||||||||||||||||||||||||||||||||||||||||||||||||| ||
Seq_2  5921  CTTTGCTCAAGCACACGTCAATTCAATTAGGTTAGAGGTCTGACCAATGCTCCAAGAAAT  5980

Seq_1  6107  CGCCTGCCCGCCTGCCCATGGTGCTACATTTCATTTGCTCACTGCCACGCACCCACCAAT  6166
             ||||||||||||||||||||||||||||||||||||||||||||||||||||||||||||
Seq_2  5981  CGCCTGCCCGCCTGCCCATGGTGCTACATTTCATTTGCTCACTGCCACGCACCCACCAAT  6040

Seq_1  6167  TTGGAA  6172
             ||||||
Seq_2  6041  TTGGAA  6046
```

BdindelWSU_15, downstream

> Bradi4g12750

TTTGTCCTGTGTTGTGGATTGAGATGTCTGGCTGCAGTTTTAGTGTTTCTTAAGTTTTGTCTATGTGCATTGCTCTTATTGCTGAGACTC
TTGGAATGCAAGCAATGTTCATGTCTGTCGTTTAAATACTGTACTCCTATTTCGGTTCGGTTAAAACCTGTGTGCTCTGATGCTTGTGC
CCTTGTTTAACTTCTCTGGGATGCGAGTTCTTAGATTGCAACTTATTATCTGTTCTCTCATGTTCTTGTTCCCTTGATTTGTTCGCAATTG
GTTTTTGTTCTGCTACTTCCTATTATATACTCTCTCTGTCCCATATAAAAATGACATTCTATTACATGTATCTAGACGTCTGTTACATCCA
TAGTTGGACAAATTTGAGTCACTTAACATGAAACGGAGGGAGTAGCAGCGTAGCCTCCGCCTCTAGTCATGTCATAAGTTTAGGTCTT
CTTTCTTTTTTGCACAGTATAGGTTTCTCCGGTTCTTTTGTCCGAAGCAGATTTTCCCCTTGTTGCCGCTGAGTTAGAGCATGGTCATTT
CAACCTAAATTTCCCTTTGTTCGTACTCCTATAGCATTAGCCACTGCAACTCTACTTTCTTTCGCTATCTTGACTCCAAAGTTTTCGTTTG
GATTCCCTGTTCTCGCATTTTTGCTGCATACGGCGATCAACTCCGCAGTAGATATTCTCTCCTCGAAGCACTCTGTCCTCTCAGAGGTA
GCATTTGTGCACTTAGAGTTCCCGATTCTAACTAAAATTGGAGTAGGGAGTATGTTATTCTGGCCCCGAAGCATTCTTTGTCTCAGTGT
AAGCATTTGTGCACCTGGGTTCCAGATCCTAACCAAAAAATTGTTATTCGGCCCGAAGCACTCTTTGTCCTCTCAGAATGCAGCATTTG

TGCACTTGGGTTTCAGATTGTAACTAAAAAAGAAAAATTAACGTCGCCTTGCTTGAATGCACTTTCCTTACCACTAAAATAAGCAGAA
GCTCTTTACCTTATAAAGAAAAAGAAGAAGCTTACTTCATCTCAAGGATTAAAAAACCTAGTTACTCAACTAAATATTCAAATCGGCCG
GAGTTGACTCCAACATGCGAGGCTGACTGTTGTACTTTGTAAGCAACCCGGTGCATTTTCATTTACTAGTTTGTGCAAACGCACATATT
GACTTGTATATTTTCATCATTTTTAAACTTATGTGTCACCTCGCAACTATTATCAAGGCTCACAACAATAATCATTGTAGATCTTATTCTT
GGCCAAGTATACCAAAGTGGGATTTTGTTGACACTAGGACCGGTACTACTACCTCTAGTAGATCACGGCAAAGCCAGATTGCAACAA
AAAGGGATCTTTTTATTACTTCCTCAAAACAACTTGCAACATAAACTCTCACTAAATAAACTTAAGTTTCAGTATCTACTTAATAACCCA
AAACTATACACATGTGCCATTTTCATGGTATGACGCTCGGCCAGAGTTAAAAACAGAAGTCATATAAATTCTCAGTATAATTTTCTGCC
AAGTTGACCAGTATAGAAAAGAGCGTACACGCCGAATGAAGTTCCACAAATAAAGAGGCAACAACATAACAACTTGCGGCACCGCC
ATCGGCTGCTTGTGACAACCTCTCATATCCACTTGTTACTTACTGAAGAAATGTGGATACATCAGCTAATCTAAGAGATGGATTTTGCG
ACTTAGGTAGCTGCCACAGCAAATGTATATATAAAGAAGGAAATGTGTGATAAGGAGGTTTCTGATTCACACGCTCATCAAGATCCCT
TACATTTCAAGTCGGTCAATTTCAGAAGTCCCAGAATATCAGATGGCAGGAACAACAAAGATTTGGAAAAAACACGGTTAACGAGTT
ATCAGTCAAAAGGTGTTCTGACTTCAGAGAGAGCTTGATGTCATTTTTAGCCGAAGAAGAAACAGCAAACAAGTCTGATACAAAGAG
TCGGACAACATGGAAGGAAAGATCTCGACCAGAACATAAGATATCTTATCCAGTACAAACAGCAAGGTTGGCCTTATCCTAATCATCT
GCTCCAGTTTACATGACATAGTCTATCCGAATACAGTACAAAACATTTGCATGCCCTGATCCAGTCAAACACCACACCCTTCTCCCCTT
ACGGCTAACAAACTATTAGTTTAGACATATTGTTAGATCAGATTATACACTTGTCATATACAGAGTATATAGGAGGAAAATCACCATC
AAAATATATCCAAATCGGAAAAGTTGCATAAAGCCAAGTGTGGCAGTGTGAAACATAAGGATCTACAATTGAAATCTGTGTGCAAAA
TGGCATGCTTCAGCATACGGAACCATAGAAGTTGGTTCCATTATACAACAACATTCTGAGAATAACAAGTATTAACGATCTGAGACAT
GATAGTTTGAGGTAAAGAGTTCAAAGGCAGAACGAGTTCATATAATGCAACATATATGACCATAGGAGAAACAAAGATGAAAGAGA
TAAACTCTAACACATTCAATTGCTGACCCCTATGTAATTTTTCTTATGCATATCTCCATTTTCCTTCTCCACTCACAAACCAATGACCACA
CACCATGCGCAGGGGATGCCCTTATCATACAGTAGTCCCATGCTACACCCATATCCTGCAAGTTTCTCCTTGATAATAACTCAAATAAA
CAACTTCTATTATGACGAAACACAAATAATCTGTAGTGTTTATAAATTATCACTCGACATGTGGCAGAAATATTGTTGCTGAAGTGGTC
GTCTTAAAGTGTCCTAAGGAATCAATCATATGCCTACACCATTACAGTTATACCAAACAATGTATCATTACGCACCACTGGGAAAAGT
AATGAAGCATTGTCAGTACAGAAATGCTGATACTGTTCACAACTAATTGATCACAATGGTGTATTGATCAAAACAGAATTGTCAGCCA
CTACTAAGCATAAAGCACATGCCAAAACAAAGCAACAAACCAAAGGAAATTTAAACAGTTCACATTTCTTTTGGAAAAAAATGATAATC
TAAACAAGAGCAGTAATAACCCAAAGACCAAATACAAACCTTGTAGAATCATCCTTGAGCACTGAGAATGCGGGAGGCCAAATCATC
ATGCAAGGATGGATGTGACCAACGTATCGATCCTTCTCTGTCATCTCATCATACACTTTACGCAGCACCCTGCGCCTGATATCCAAATA
GAGAGTGTATGGACCCATCTGCAAGAACACAAACTCAGCATTGTCTCCCACCTGGGTTATACTCACAGCAGTAGTATGCTCATCTTCA
ACCATGACATCAGACATCTGGAAAGCAGCACACATCTGACGCAAACATATGGTATCCACCAGCAACCAGTTATCATCCTTGTGGAGCC
AGATGCGAAACTGAAGTTCCTTCAAATCGATGAGGTAAACTCCGGAATCATCTGCCCGTGAGAATACAGCATCTCTACTGTGAAATCC
CATCCCATATTTATATCCAGAAGACCACTCCACCCCTTCTGGGAGCTGAATAGTGAAGAAACTGGAGCTCGCCAAATCCAACACAAGA
ATGTCGTTACAGGTGCCCCCAGCCATGTAGATCCTATAATTGATGAGTAGAGGTTTTGGCCTCCATAGCGGATGGGGGAGTTGTGTC
ATGGCTTTGGTAAGCATGACCCAGACACCATCTCACAACATGTGTACCTTCGCGGTAGATCTTCCAACAATGACATTGATCTCCATTGC
CATCCACATGTACGATAAGCAGCCGTCGCCTCCTGTGGAAAGGAAGCAAGAGAATGTAGGTTTATGGCTGTCGTCTGTCATGGCACG
TGTTGGTGCTGGGAGGACCACCACGCCTCTGTCAGGGAACAGCGGGTTGTGCACTTCATATGTGTATTTGTGGTTCTGGAATCTGGA
GATGAAGACACTGTCGTTCCGGCATTGCTCAATCATTATTCTGTTGATGGTGTCCAAGCTTAAGGTTGCCATGCGGCGGAGGACGGA
GGCGAGCTCCGGCTCTTGGGTCTGGGTCAGCAACGGCACGAAGCGAGGAAGAACCTTGGCCGACTCGGGTGTATCGGTAAAGAAC
ACGCCATTGACGTACAAGCCGAGGAGGCGGGGCGGGTGGCGCTGGCGGAATTGGCGGAGGAAGGCCCGGTTGGCAGCGTTGTGG
AACCAGCGCTTGCAGACGAGCGCGGCGCGGGCGAGTGTGGTGGGGAGGCCGACGCGGAGGAGGTTGTCGTCGTCGAGCACCTTG
GACACGAGGGCCGCGGCCGCGGCCGGCGAGGGCAGGAAATCCATTGTTGGCCGATCTTTGAATAAGGAAGAGAAATCAAAATCTTA
CACTTATGGTATCATTCCCCTCCTCTGCTGCTGGAACAAGGAAAGGTAGGAGTTTAAGAAGAAAAGTCAAGCTAGGGTTCCGTGCAA
GAAACGAAATTGGGGCAAAATAAGACTTACCTGGAATCGCGAGAGGGGAAGGTGGATTTGTGGGGGCGCAGGCACCGACCGCCGT
CGGGGGTTCAATTCGGACCGCCGGCAGAAGTCCCGTGCCAGGGTGGATGCCGGTCTGGCTCGGTCCGAGCTCGCCGCGAGCTGTGC
GGCAGCGGCTGCCTAGGAGAGTCAGGGTTCGTAGGAACTGAGAAGCAGTTAATTTTGACCTAGGCAAATGGGACTGGACTGGGCTC
GAGGCATCTATGGGCATTGAAAACTGAGCGTGGGCCGCCAGTGGGCCAAAATGGCCAGCTCGCCGAGCTGGACCGAGCTCAGCTCG
GCTCGGCCTGAAGATGTGACGAGCCGTTTTCGTGGGCTAGGCTTGGTCTAAAAAAATCAGGCTCGGGGCGAACTGGCTCGGCTCGC
CACGGGCTCGGGCCAGCTCACCGAGCCCAGCTCGAACGTCCACCCTGAGTCCCATCAATGCGGCGCTTCGTGGTGGTGTGGAGCAG
AGCAGGCAGCCCACCAGGACAGGGTGAGAAAAGGTAATCTCCCATGGAATTCATTCACATGACACCTAGCCGTACTCCATTTCTATCT
GCCTGTTTCCAAAAAAAAATCACTGTGAACTTCAAAGATATTTTTCAGAGAAATAAAATAAATAAATCCTGCGCACAGTTTGAGATCC
CAAACTAGAATATTTTCCAACATGCAAAGAATAACAGAAATAGCAAGGAGGATGTCATTACATGCTTAATCAGGAAGCTTGTTATTTA

ACTGTAACCACTCTGAAAATACTTCTCTTGCCATCAGCACCTGCTCCTTTCTACAGTAATTAACAGGCTCAGTGATTGGCTTCACATTGA
TTAAAGCCACACAACGGGCAAGTCAAGATTACAAGATCCTCCCATTTGGTCCAAGTACACAGCCCCAGTCCCGACTGACAAGTCAATA
ACTGCTCTTCTTGACAGTAAGATGCTGAAAAGCATGGGACGAGCAATTTGTGATGACAATGACTTTGAACTGCCAATAAATGACATTT
TGGGACTCGTTTGGACAGTCCAAGCGCCTAAAGCTCATAAAATCATGCGGAGAGACTCAAAAGACGAAGACCAAGGCTTCGAGTCTT
TGGATCCAGGTAGCTTCCGCTGTGGTGAGGCCACCTTTCTGGCTATCACATGCATCCGCTCCGTATCTGCAGGTCTCCTTGATCTGCAA
GCCTTCCTGACTGCAACACCTACAGTTTTTTCCTACCTAGCTTACCGAATATTCCAAGACCTAAATCATCAAGTGATTCTTCTCGCAGCA
TTCTTATTCGTGCAGGCACCGCACCTTGGGTTCTGCCAATTTCTTCTTCATCGTGCTTGCAGCTGCAGCATCGTTGCTGGCCATGCAGG
GATCCAACTATGGCGTCTATCCCATTGTCAACCTCCCAACACTGTTGATCAATAAAAAACCTCCCTCCCAAACGGCTTGAGCTACATGG
ACGGTACAACCGTCCGTGCCATCGTCCTCCGTTCACCAAGGGCACGGCCTTTTTCTTTTGATGGGTAGTCCTTTGCTGAAGGGTTCTTC
TGTGCCTCCCTCTGTGATGTCTTCCTCTTTTCTGTCTTCATTGTAAGCACTGACAGTGGCGGCTGGTTCTACGACATGGCAACTGGACC
GCAGGTTATCTGGTTGGCCAACCGGGCTGGCCCTGTCAGGGAGAACACCACCCTTGAGCTTACTAGGAATGGCAACCTCATCCTCCG
TGATGCCAATGGGATCATGGGAGGATGGTCTGGTCTAGTGGCACTTCAGACAAATCCATGATTGGCATGGAGATCACGAAGTTCGG
CAACCTGGTGCTATTTGATCAGAGGAATGCAACTGTGTGGCAGTCATTTGATCATCCGACTGATTCATTGGCCCCCGTGCAATCACT

>BdiBd21-3.4G0184100

TTTGTCCTTTGTTGCGGATAGAGATGTCTGGCTGCAGTTTTAGTGTTCCTTAAGTTTTGTCTATGTGCATTGCTCTTATTGCTGAGACTC
TTGGAACTATGCAAGCAATGCTCATGTCTGTCGTTTAAATACTGTACTCCTATTTCGGTTCGGTTAAGACCTGTGTGCTCTGATGCTTG
TGCCCTTGTTTAACTTCTCTGGGATGCGAGTTCTTAGATTGCAACTTATTATCTGTTCTCTCATGTTCTTGTTCCCTTGATTTGTTCGCAA
TTGGTTTTTGTTCTGCTACTTACTATTATGTACTTCCTCCGTCCCATATAAAATGACCTTCTATTACATGTATCTAGATGTCTTCTAGGCA
TAGATGCATGCATATTTGGACAAATTTGAGTCACTTGATATGGGATGGAGGGAGTAGCAGCGTAGACTCTAATCATGTCATAAGTTT
AGGTCTTCCTTCTTTTTTTCACAGTATAGGTTTCTCCGGTTCTTTTGTCTGAAGCAGATTTTCCCCTTGTTGCCGCTGAGTTAGAGCATG
GTCATTTCAACCTAAATTTCCCTTTTTTTCGTACTCCTATAGCATTAGCCACTGCAACTCTACTTTGTTTGGCTATCTTGACTCCAAAGTT
TTCGTTTGGATTCCCTGTTCTAGCATTTGCTGCATACGGCGATCAACTTCGCAGTAGATATTCTCGCCTCGAAGCAGTCTGTCCTCTCA
GAGGGAGCATTTGTGCACTTGAGTTCCCGATTCTGACTAAAATTGGAGTACGGAGTATGTTATTCTGGCCCCGAAGCATTCTTTGTCT
CAGTGGAAGCATTTGTGCACCTGGGTTCCAGATCCTAACCAAAAAATTGTTATTCGGCCCCGAAGCACTCTTTGTCCTCTCAGAATGC
AGCATTTGTGCACTTGGGTTTCAGATTGTAACTAAAAAAAAAATTAACGTCTCCTTGCTTGAATGCACTTTCCTTACCACTAAAATAAT
CAGAAGCTCTTTACCCTTATAAAGAAAAAGAAGAAGCTAACTTCATCTCAAGAATTAAAAAACCTAGTTACTCATCTAAATATTCAAAT
CGGCCGGAGTTGACTTCCAACACGCGAGGCTTCGACTGTTGTATTCTGTAAGCCTCCCGGTGCATTTTCATTTACTAGCTTGTGCAACG
CACATATTGACTTGTATATTTTCATCATTTTTAAGCTTATGTGTCACCACGCAACTATTATCAAGGCTCACAAAAATAATCATTGTAGAT
CTTATTGTTGGCCAAGTATACCAAAGTGGGATTTTGTTGACACTAGGACCGGTACTAACAGGGGCAGAGCCGGGGTATGGCCGAGC
CCGAGGCCAACTTCGTTAAACAGTGTTAAATAGTGCTGAGCAGTGCTAAAAGTATGAAGAAAAACATGCCCACGCCCCAGGCCACGG
CCCAGTGTGCCTGGGACCTGTCTCCGCCTCTGGGTACTACCTCTAGTAGATCATGGCAAAGCCAGATTGCAACAAAAAGGGATCTTTT
TATTACTTCCTCAAAACAACTTGCAACATAAAGTCTCACTAAATAAACTTAAGTTTCAATATCTACTTAATAACCCAAAACTACACACAT
GTGCCATTTTCCTTGTATGGTGATCGGCCAGAGTTGAAAAAACAGAAGTCATATAAATTGTCAGTATAATTTTCTGCCAAGTTGACCA
ATATAGAAAAGAGCGTACAAGCTGAATGAAGCTTCATAAATAAAGAGGCAACAACATATCAACTTGCGGCCCCGCCATCGGCTGCTT
GTGACAACCTCTCATATCCACTTGTTACTTACTGAAGAAATGTGGATACATCAGCTAATCCAAGAGATGGATTTTCCGACTTAGGTAG
CTGCCACAGCAAATATATATATATAAAGAAGGAAATGTGTGATAAGGAGGTTTCTGATTCATACGCTCATCAAGATCCCTTACATTTC
AAGTTGGTCAATTTCAGAAGTCCCAGAATATCAGATGGCAAGAACAACAAATATTTGGAAAAAAACACGGTTTAACGAGTTATCAGTC
AAAAGGTGTTCTGACTTCAGAGAGAGCTTGATGTCATTTTCAGCCAAAGAAGAAACAGCAAATAAGTCTGATACAAAGAGTCGGACA
TGGAAGGAAAGATCTCGACCAGAACATAAGATATCTTATCCAGTACAAACAGCAAGGTTGGCCTTATCCTAATCATCTGCTCCAGTTT
ACATGACATAGTCTATCCGAATACAGTACAAAACATTTGCATGCCCTAATCCAGTTAAACACCACACCCTTCTCCCCTTACGGCTAACA
AATTATTAGTTTAGACAGCCAACATAAGAGGTTTTGGCTATCAGAAAGGAAGAAAATGCAGCCCACTGATCTTCCTTGATGGTGTCCT
TGTCACTGGCGTACACAACATCCAAGATCCTCCTGCAACCCTTGTTCTTCAAGCGGCACTTCTGTTTAAAGTAAAGCACGACAGGGAT
TAGGATGATTCTCGCAAAAAAAAAGGAAAAAAAGACAGGGATTAGGATGAAAATAACTAAGAACAGGATATCAAATGAACTAATAAG
ACAAGTGGCTACTACAATGGGCAATGGCTATCAACTATTATGTGGCACTCAAAGGCCAAAAATCAATGTATTTGTTTCAGCAAAACGG
ATAAAACTATATATCCTAAACCACTAGTCAAATAGAACAAACAACTGCTCTCATAAGGTTAAAACGTCACCCAGGAAACATATTGTTA
GATCAGATTATACCCTTGTCATATATATAGGAGGAAAATTACCACCAAAATATATCCAAATCGGAGAAGTTGCGAGAAGCCAAGTGT
GGCAGTGTGAAATATAAGGATCTACCATTGAAATCTGTGTGCAAAATGGCATGCTTCAGCATACTGAACCATAGAAGTCAGTTCCATT
ATACAACAACATTCTGAGAATAACAAGTATTAACGATCTGCGACATGATAGTTTGAGGTAAAGAGTTCAAAGGCAGAACGAGTTCAT

ATAATGCAACATATATGACCGTAGGAGAAACAAAGATGAAAGAGATAAACTCTAACACATTCAATTGCTGACCCCTATGTGTGTAATT
TTTCTTATGCATATCTCCATTTTCCTTCTCCACTCACAAACCAATGACCACACACCATGCGCAGGGGACGCCCGTATCATACAGTAGTC
CCATGCTACACCCATATCCTGCAAGTTTCTCCTTGATAATAACTCAAACAGACAACTTCTATTATGACGAAACACAAATAATCTGTAGT
GTTTATAAATTATCACTCGACATGTGGCAGAAATATTGTTGCTGAAGTGGTAGTCTTAAAGTGTCCTAAGGAATCAATCATATACCTA
CACCATTACAGTTATACCAAACAATGTATCATTACGCACCGGGGGAAAAGTAATGAAGCATTGTCAGTACAGAAATCCTGATACTTTT
CACAACTAATTGATCACAATGGTGTATTGATCAAAACAGAATTGTCAACTACTGCTAAGCATAAAGCACATGCCAAAACAAAGCAACA
AATCAAAGGAAATTTAAAAAGTTCACATTTCATTTGGAGAAAATGATAATCGAAGCAAGAGCAGTAATAACCCAAAGACCAAATACA
AACCTTGTAGAATCATCCTTGAGTGCTGGGAATGCGGGAGGCCAAATCATCATGCAAGGATGGATGTGACCAACGTATCGATCCTTC
TCTGTCATCTCATCATACACTTTACGCAGCACCCTGCGCCTGATATCCAAGTAGAGAGTGTATGGACCCATCTGCAAGAACACAAACT
CAGCATTGTCTCCCACCTGGGTTATACTCACAGCAGTAGTATGCTCATCCTCAACCTGACATCAGACATCTCGAAAGCAGCACACATCT
GACGCAAACATATGGTGGTATCCACCAGCAACCAGTTATCATCCTTGTGGATCCAGATGCGAAACCGAAGTTCCTTCAAATCGATGAG
GTAAACTCCGGAATCATCTGCCCGCGAGAATACAACATCTCTACTGTGAAATCCCATCCCATATTTATATCCAGAAGACCACTCCACCC
CTTCTGGGAGCTGAATAGTGAAGAAACTGGAGCTCGCCAAATCCAACACAAGAATGTCGTTACAGGTGCCCCCAGCCATGTAGATCC
TATCATTGATGAGGAGAGGTTTTGGCCTCCATAGTGGATGGGGGAGTTGTGTCGTGGCTTTGGTAAGCATGACCCAGAAACCATCTC
GCAACATGTGTACCTTCGCGGTAGATCTTGCAACAATGACATTGATCTCCATACATTGATCTCCATTGCCATCCACATGTACGATAAGC
AGCCGTCGCCTCCTGTTGAAAGGAAGCAAGAGAAGGTAGGTTTATGGCCGTCGTCTGTCATGGCATGTGTTGGTGCCTGGAGGACC
ACCACACCTCTGTCAGGGAACAGCGGGTTATGCACTTCATATCTGTATTTGTGGTTCTGGAATCTGGAGATGAAGACACTGTCATTCT
GGCATTGCTCAATCATTATTCTGTCGATGGTGTCCAAGCTTAAGGTTGACATGCGATGGAGGACGGTGGCGAGCTCCGGGTCTTGGG
TCTGGGTCAGCATCAGCACGAAGCGAGGAAGAACCTTTGCCGACTCGGGTGTATCAGTAAAGAACATGCCATTGACGTACAAGCCG
AGGAGGCGGGGCGGGTGGCGCTGGCAGAATTGGCGGAGGAAGGCCCGGTTGGCAGCGTTGTGGAACCAGCGCTTGCAGACGAGC
GTGGCGCGGACGAGGGTGGTGGGGAGACCGATGCGGAGGAGGATCTCGCGGAGGAGGTCGTCGTCGTCGAGCACCTTGGACACG
AGGGTCGCGGCCGCGGCCGGCGAGGGCGGGGAATCCATTGTTGGCCGATCTCTTGAATAAGGAAGAGAAATCAAAATCTTACACTT
ATGGTATCATTCCCCTCCTCTGCTGCTGGAACAAGGAAAGGTAGGAATTTAAGAAGAAAAGTCAAGCTAGGGTTCCGCACAAGAAAC
GAAATTGGGGCAAAATAAGACTTACCTGGAATCGCGAGAGGGGAAGGCGGATTTGTGGGGGCGTAGGCACCGACCGCCGTCGGG
GGTTCAATTCGGACCTCCGGCGGAAGTCCTGTCCCAGGGTGGACGCCGGTCTGGCTCGGTCCAAGCTCGTCGCGAGCTGTGCAGCG
GCCGCTGCCTAGGAGAGTCAGGGTTCGTAGGAACTAAGTAGCAGTTAATTTTGACCTAGGCAAATGGGACTGGACTGGGCTCGAGG
CATCTATGAGCAGTGAAAACTGAGCGTGGGCCGCTAGTGGGCCAAAATGGCCAGCTCGCCAAGCTGGACCGAGCTCAGCTTGGCTC
GGCCTGAAGATGTGACGAGCCGTTTCCGTGGGCTCGGCTTGGTCTAAAAAAATCAGGCTCGGGCCGAGCTGGCTCGGCTCGCCACA
GGCTCGGGCCAGCTCGCCGAGCCCAGCTCGAACGTCCACCCTGAGTCCCGTCAATGTGGCGTTTCGTGGTGGTGTGGAGCAGAGCA
GAGCAGGCAGCCCACCAGGACAGGGTGAGAAAAGGTAATCACCCATGGAATTCATTCACATGACACCTAGCCGTTCTCGATTTCTAT
CTGCCTGTTTCCAAAAAAAAGATCACTGTGAACTTCAAAGATATTTTTCAGAGAAATAAAATAAATAAATCCTGCACACAGTTTGAGA
TCCCAAACTAGAATATTTTCCAACATGCAAAGAATAACAGAAATAGCAGGGAGGATGCCATTACATGCTTAATCAGGAAGCTTGTTAT
TTAACTGTAACCACTCTGAAAATACTTCTCTTGCCATCAGCACCTGCTCCTTTCTACAGTAATTAACAGGCTCAGTGATTGGCTTCACAT
TGATTAAAGCGACACAACGGGCAAGTTAAGATTACAAGATCCTCCCATTTGGTCCACGTACACAGCCCCAGTCCCGACAGACAAGTC
AATGACTCCTCTTCTTGACAGTAAGATGCTGAAAAGCATGGGACGAGCAATTTGTGATGACAATGACTTTGAACTGCCAATCAGTGAC
ATTTTGGGACTCTGTTGGACAGTCCAGGCGCCTAAAGCTCATAAATCATGCGGAGAGACTCAAAAAACGAAGACCAAGACTTCCGAG
TCTTCGGATCCAGGCAGCTTCCGCTGTGGCGAGGCCACCTTTCTGGCTATCACATGCATCCGCTCCGTATCTGCAGGTCTCCTTGATCC
GCAAGCCTTC

```
Alignment of Sequence_1:  [Untitled Sequence #1] with  Sequence_2: [Sequence Window #2]

Similarity : 5350/6334 (84.46 %)

Seq_1  1      TTTGTCCTGTGTTGTGGATTGAGATGTCTGGCTGCAGTTTTAGTGTTTCTTAAGTTTTGT  60
              |||||||| ||||| |||| ||||||||||||||||||||||||||| ||||||||||||
Seq_2  1      TTTGTCCTTTGTTGCGGATAGAGATGTCTGGCTGCAGTTTTAGTGTTCCTTAAGTTTTGT  60


Seq_1  61     CTATGTGCATTGCTCTTATTGCTGAGACTCTTGGAA---TGCAAGCAATGTTCATGTCTG  117
              ||||||||||||||||||||||||||||||||||||   ||||||||||| |||||||||
Seq_2  61     CTATGTGCATTGCTCTTATTGCTGAGACTCTTGGAACTATGCAAGCAATGCTCATGTCTG  120
```

```
Seq_1  118   TCGTTTAAATACTGTACTCCTATTTCGGTTCGGTTAAAACCTGTGTGCTCTGATGCTTGT  177
             ||||||||||||||||||||||||||||||||||||| ||||||||||||||||||||||
Seq_2  121   TCGTTTAAATACTGTACTCCTATTTCGGTTCGGTTAAGACCTGTGTGCTCTGATGCTTGT  180

Seq_1  178   GCCCTTGTTTAACTTCTCTGGGATGCGAGTTCTTAGATTGCAACTTATTATCTGTTCTCT  237
             ||||||||||||||||||||||||||||||||||||||||||||||||||||||||||||
Seq_2  181   GCCCTTGTTTAACTTCTCTGGGATGCGAGTTCTTAGATTGCAACTTATTATCTGTTCTCT  240

Seq_1  238   CATGTTCTTGTTCCCTTGATTTGTTCGCAATTGGTTTTTGTTCTGCTACTTCCTATTATA  297
             ||||||||||||||||||||||||||||||||||||||||||||||||||| |   |   
Seq_2  241   CATGTTCTTGTTCCCTTGATTTGTTCGCAATTGGTTTTTGTTCTGCTACTTAC-TATTAT  299

Seq_1  298   TACTCTCTCTGTCCCATATAAAAATGACATTCTATTACATGTATCTAGA-----------  346
                  ||      ||     |||||||| ||||||||||||||||||||           
Seq_2  300   GTACTTCCTCCGTCCCATATAAAATGACCTTCTATTACATGTATCTAGATGTCTTCTAGG  359

Seq_1  347   CGTCTGTTACATCCATAGTTGGACAAATTTGAGTCACTTAACATGAAACGGAGGGAGTAG  406
             |    | | ||| |||| ||||||||||||||||||||| | |||  | |||||||||||
Seq_2  360   CA-TAGATGCATGCATATTTGGACAAATTTGAGTCACTTGATATGGGATGGAGGGAGTAG  418

Seq_1  407   CAGCGTAGCCTCCGCCTCTAGTCATGTCATAAGTTTAGGTCTTCTTTCTTTTTTGCACAG  466
             ||||||||       ||||| ||||||||||||||||||||||| ||||||||| |||||
Seq_2  419   CAGCGTAG-----A-CTCTAATCATGTCATAAGTTTAGGTCTTCCTTCTTTTTTTCACAG  472

Seq_1  467   TATAGGTTTCTCCGGTTCTTTTGTCCGAAGCAGATTTTCCCCTTGTTGCCGCTGAGTTAG  526
             ||||||||||||||||||||||||| ||||||||||||||||||||||||||||||||||
Seq_2  473   TATAGGTTTCTCCGGTTCTTTTGTCTGAAGCAGATTTTCCCCTTGTTGCCGCTGAGTTAG  532

Seq_1  527   AGCATGGTCATTTCAACCTAAATTTCCCTTTG-TTCGTACTCCTATAGCATTAGCCACTG  585
             |||||||||||||||||||||||||||||||  |||||||||||||||||||||||||||
Seq_2  533   AGCATGGTCATTTCAACCTAAATTTCCCTTTTTTTCGTACTCCTATAGCATTAGCCACTG  592

Seq_1  586   CAACTCTACTTTCTTTCGCTATCTTGACTCCAAAGTTTTCGTTTGGATTCCCTGTTCTCG  645
             |||||||||||| ||| ||||||||||||||||||||||||||||||||||||||||| |
Seq_2  593   CAACTCTACTTTGTTTGGCTATCTTGACTCCAAAGTTTTCGTTTGGATTCCCTGTTCTAG  652

Seq_1  646   CATTTTTGCTGCATACGGCGATCAACTCCGCAGTAGATATTCTCTCCTCGAAGCACTCTG  705
             ||  ||||||||||||||||||||||| |||||||||||||||| |||||||||| ||||
Seq_2  653   CA--TTTGCTGCATACGGCGATCAACTTCGCAGTAGATATTCTCGCCTCGAAGCAGTCTG  710

Seq_1  706   TCCTCTCAGAGGTAGCATTTGTGCACTTAGAGTTCCCGATTCTAACTAAAATTGGAGTAG  765
             |||||||||||| ||||||||||||||| |||||||||||||| ||||||||||||||| 
Seq_2  711   TCCTCTCAGAGGGAGCATTTGTGCACTT-GAGTTCCCGATTCTGACTAAAATTGGAGTAC  769

Seq_1  766   GGAGTATGTTATTCTGGCCCCGAAGCATTCTTTGTCTCAGTGTAAGCATTTGTGCACCTG  825
             |||||||||||||||||||||||||||||||||||||||||| |||||||||||||||||
Seq_2  770   GGAGTATGTTATTCTGGCCCCGAAGCATTCTTTGTCTCAGTGGAAGCATTTGTGCACCTG  829

Seq_1  826   GGTTCCAGATCCTAACCAAAAAATTGTTATTCGGCCC-GAAGCACTCTTTGTCCTCTCAG  884
             ||||||||||||||||||||||||||||||||||||| ||||||||||||||||||||||
Seq_2  830   GGTTCCAGATCCTAACCAAAAAATTGTTATTCGGCCCCGAAGCACTCTTTGTCCTCTCAG  889

Seq_1  885   AATGCAGCATTTGTGCACTTGGGTTTCAGATTGTAACTAAAAAAGAAAAATTAACGTCGC  944
             ||||||||||||||||||||||||||||||||||||||||||||  |||||||||||| |
Seq_2  890   AATGCAGCATTTGTGCACTTGGGTTTCAGATTGTAACTAAAAAA--AAAATTAACGTCTC  947
```

```
Seq_1  945   CTTGCTTGAATGCACTTTCCTTACCACTAAAATAAGCAGAAGCTCTTTACC-TTATAAAG  1003
             ||||||||||||||||||||||||||||||||||| ||||||||||||||| ||||||||
Seq_2  948   CTTGCTTGAATGCACTTTCCTTACCACTAAAATAATCAGAAGCTCTTTACCCTTATAAAG  1007


Seq_1  1004  AAAAAGAAGAAGCTTACTTCATCTCAAGGATTAAAAAACCTAGTTACTCAACTAAATATT  1063
             |||||||||||||| ||||||||||||| ||||||||||||||||||||| |||||||||
Seq_2  1008  AAAAAGAAGAAGCTAACTTCATCTCAAGAATTAAAAAACCTAGTTACTCATCTAAATATT  1067


Seq_1  1064  CAAATCGGCCGGAGTTGACT--C-CAACATGCGAGGCTGACTGTTGTACTTTGTAAGCAA  1120
             ||||||||||||||||||||  |   ||  | |     |||||||||| | |||||||
Seq_2  1068  CAAATCGGCCGGAGTTGACTTCCAACACGCGAGGCTTCGACTGTTGTATTCTGTAAGCCT  1127


Seq_1  1121  CCCGGTGCATTTTCATTTACTAGTTTGTGCAAACGCACATATTGACTTGTATATTTTCAT  1180
             ||||||||||||||||||||||| |||||| |||||||||||||||||||||||||||||
Seq_2  1128  CCCGGTGCATTTTCATTTACTAGCTTGTGC-AACGCACATATTGACTTGTATATTTTCAT  1186


Seq_1  1181  CATTTTTAAACTTATGTGTCACCTCGCAACTATTATCAAGGCTCACAACAATAATCATTG  1240
             ||||||||| ||||||||||||| |||||||||||||||||||||||| |||||||||||
Seq_2  1187  CATTTTTAAGCTTATGTGTCACCACGCAACTATTATCAAGGCTCACAAAAATAATCATTG  1246


Seq_1  1241  TAGATCTTATTCTTGGCCAAGTATACCAAAGTGGGATTTTGTTGACACTAGGACCGGTAC  1300
             ||||||||||| ||||||||||||||||||||||||||||||||||||||||||||||||
Seq_2  1247  TAGATCTTATTGTTGGCCAAGTATACCAAAGTGGGATTTTGTTGACACTAGGACCGGTAC  1306


Seq_1  1301  TA----------------------------------------------------------  1302
             ||
Seq_2  1307  TAACAGGGGCAGAGCCGGGGTATGGCCGAGCCCGAGGCCAACTTCGTTAAACAGTGTTAA  1366


Seq_1  1303  ------------------------------------------------------------  1302

Seq_2  1367  ATAGTGCTGAGCAGTGCTAAAAGTATGAAGAAAAACATGCCCACGCCCCAGGCCACGGCC  1426


Seq_1  1303  ----------------------------------CTACCTCTAGTAGATCACGGCAAAGC  1328
                                               ||||||||||||||||| ||||||||
Seq_2  1427  CAGTGTGCCTGGGACCTGTCTCCGCCTCTGGGTACTACCTCTAGTAGATCATGGCAAAGC  1486


Seq_1  1329  CAGATTGCAACAAAAAGGGATCTTTTTATTACTTCCTCAAAACAACTTGCAACATAAACT  1388
             |||||||||||||||||||||||||||||||||||||||||||||||||||||||||| |
Seq_2  1487  CAGATTGCAACAAAAAGGGATCTTTTTATTACTTCCTCAAAACAACTTGCAACATAAAGT  1546


Seq_1  1389  CTCACTAAATAAACTTAAGTTTCAGTATCTACTTAATAACCCAAAACTATACACATGTGC  1448
             |||||||||||||||||||||||| |||||||||||||||||||||||| ||||||||||
Seq_2  1547  CTCACTAAATAAACTTAAGTTTCAATATCTACTTAATAACCCAAAACTACACACATGTGC  1606


Seq_1  1449  CATTTTCATGGTATGACGCTCGGCCAGAGTT--AAAAACAGAAGTCATATAAATTCTCAG  1506
             ||||||| | |||||  | ||||||||||||  |||||||||||||||||||||| ||||
Seq_2  1607  CATTTTCCTTGTATGGTGATCGGCCAGAGTTGAAAAAACAGAAGTCATATAAATTGTCAG  1666


Seq_1  1507  TATAATTTTCTGCCAAGTTGACCAGTATAGAAAAGAGCGTACACGCCGAATGAAGTTCCA  1566
             |||||||||||||||||||||||| |||||||||||||||||| || |||||||| | ||
Seq_2  1667  TATAATTTTCTGCCAAGTTGACCAATATAGAAAAGAGCGTACAAGCTGAATGAAGCTTCA  1726


Seq_1  1567  CAAATAAAGAGGCAACAACATAACAACTTGCGGCACCGCCATCGGCTGCTTGTGACAACC  1626
              ||||||||||||||||||||| ||||||||||| |||||||||||||||||||||||||
Seq_2  1727  TAAATAAAGAGGCAACAACATATCAACTTGCGGCCCCGCCATCGGCTGCTTGTGACAACC  1786
```

```
Seq_1  1627  TCTCATATCCACTTGTTACTTACTGAAGAAATGTGGATACATCAGCTAATCTAAGAGATG  1686
             ||||||||||||||||||||||||||||||||||||||||||||||||||| ||||||||
Seq_2  1787  TCTCATATCCACTTGTTACTTACTGAAGAAATGTGGATACATCAGCTAATCCAAGAGATG  1846

Seq_1  1687  GATTTTGCGACTTAGGTAGCTGCCACAGCAAATG--TATATATAAAGAAGGAAATGTGTG  1744
             |||||| ||||||||||||||||||||||||||   ||||||||||||||||||||||||
Seq_2  1847  GATTTTCCGACTTAGGTAGCTGCCACAGCAAATATATATATATAAAGAAGGAAATGTGTG  1906

Seq_1  1745  ATAAGGAGGTTTCTGATTCACACGCTCATCAAGATCCCTTACATTTCAAGTCGGTCAATT  1804
             |||||||||||||||||||| |||||||||||||||||||||||||||||| ||||||||
Seq_2  1907  ATAAGGAGGTTTCTGATTCATACGCTCATCAAGATCCCTTACATTTCAAGTTGGTCAATT  1966

Seq_1  1805  TCAGAAGTCCCAGAATATCAGATGGCAGGAACAACAAAGATTTGGAAAAAACACGGTT-A  1863
             ||||||||||||||||||||||||||| |||||||||| ||||||||||||||||||| |
Seq_2  1967  TCAGAAGTCCCAGAATATCAGATGGCAAGAACAACAAATATTTGGAAAAAACACGGTTTA  2026

Seq_1  1864  ACGAGTTATCAGTCAAAAGGTGTTCTGACTTCAGAGAGAGCTTGATGTCATTTTTAGCCG  1923
             |||||||||||||||||||||||||||||||||||||||||||||||||||||| |||| 
Seq_2  2027  ACGAGTTATCAGTCAAAAGGTGTTCTGACTTCAGAGAGAGCTTGATGTCATTTTCAGCCA  2086

Seq_1  1924  AAGAAGAAACAGCAAACAAGTCTGATACAAAGAGTCGGACAACATGGAAGGAAAGATCTC  1983
             |||||||||||||||| ||||||||||||||||||||||||   ||||||||||||||||
Seq_2  2087  AAGAAGAAACAGCAAATAAGTCTGATACAAAGAGTCGGACA---TGGAAGGAAAGATCTC  2143

Seq_1  1984  GACCAGAACATAAGATATCTTATCCAGTACAAACAGCAAGGTTGGCCTTATCCTAATCAT  2043
             ||||||||||||||||||||||||||||||||||||||||||||||||||||||||||||
Seq_2  2144  GACCAGAACATAAGATATCTTATCCAGTACAAACAGCAAGGTTGGCCTTATCCTAATCAT  2203

Seq_1  2044  CTGCTCCAGTTTACATGACATAGTCTATCCGAATACAGTACAAAACATTTGCATGCCCTG  2103
             ||||||||||||||||||||||||||||||||||||||||||||||||||||||||||| 
Seq_2  2204  CTGCTCCAGTTTACATGACATAGTCTATCCGAATACAGTACAAAACATTTGCATGCCCTA  2263

Seq_1  2104  ATCCAGTCAAACACCACACCCTTCTCCCCTTACGGCTAACAAACTATTAGTTTAG-----  2158
             ||||||| ||||||||||||||||||||||||||||||||||| |||||||||||     
Seq_2  2264  ATCCAGTTAAACACCACACCCTTCTCCCCTTACGGCTAACAAATTATTAGTTTAGACAGC  2323

Seq_1  2159  ------------------------------------------------------------  2158

Seq_2  2324  CAACATAAGAGGTTTTGGCTATCAGAAAGGAAGAAAATGCAGCCCACTGATCTTCCTTGA  2383

Seq_1  2159  ------------------------------------------------------------  2158

Seq_2  2384  TGGTGTCCTTGTCACTGGCGTACACAACATCCAAGATCCTCCTGCAACCCTTGTTCTTCA  2443

Seq_1  2159  ------------------------------------------------------------  2158

Seq_2  2444  AGCGGCACTTCTGTTTAAAGTAAAGCACGACAGGGATTAGGATGATTCTCGCAAAAAAAA  2503

Seq_1  2159  ------------------------------------------------------------  2158

Seq_2  2504  GGAAAAAAAGACAGGGATTAGGATGAAAATAACTAAGAACAGGATATCAAATGAACTAAT  2563

Seq_1  2159  ------------------------------------------------------------  2158

Seq_2  2564  AAGACAAGTGGCTACTACAATGGGCAATGGCTATCAACTATTATGTGGCACTCAAAGGCC  2623
```

```
Seq_1  2159  ------------------------------------------------------------  2158

Seq_2  2624  AAAAATCAATGTATTTGTTTCAGCAAAACGGATAAAACTATATATCCTAAACCACTAGTC  2683


Seq_1  2159  ------------------------------------------------ACATATTGTTAG  2170
                                                             ||||||||||||
Seq_2  2684  AAATAGAACAAACAACTGCTCTCATAAGGTTAAAACGTCACCCAGGAAACATATTGTTAG  2743


Seq_1  2171  ATCAGATTATACACTTGTCATATACAGAGTATATAGGAGGAAAATCACCATCAAAATATA  2230
             |||||||||||| |||||||||       |||||||||||||||| |||| |||||||||
Seq_2  2744  ATCAGATTATACCCTTGTCATA-------TATATAGGAGGAAAATTACCACCAAAATATA  2796


Seq_1  2231  TCCAAATCGGAAAAGTTGCATAAAGCCAAGTGTGGCAGTGTGAAACATAAGGATCTACAA  2290
             ||||||||||| |||||||   ||||||||||||||||||||||| |||||||||||| |
Seq_2  2797  TCCAAATCGGAGAAGTTGCGAGAAGCCAAGTGTGGCAGTGTGAAATATAAGGATCTACCA  2856


Seq_1  2291  TTGAAATCTGTGTGCAAAATGGCATGCTTCAGCATACGGAACCATAGAAGTTGGTTCCAT  2350
             ||||||||||||||||||||||||||||||||||||| |||||||||||||  |||||||
Seq_2  2857  TTGAAATCTGTGTGCAAAATGGCATGCTTCAGCATACTGAACCATAGAAGTCAGTTCCAT  2916


Seq_1  2351  TATACAACAACATTCTGAGAATAACAAGTATTAACGATCTGAGACATGATAGTTTGAGGT  2410
             ||||||||||||||||||||||||||||||||||||||||| ||||||||||||||||||
Seq_2  2917  TATACAACAACATTCTGAGAATAACAAGTATTAACGATCTGCGACATGATAGTTTGAGGT  2976


Seq_1  2411  AAAGAGTTCAAAGGCAGAACGAGTTCATATAATGCAACATATATGACCATAGGAGAAACA  2470
             |||||||||||||||||||||||||||||||||||||||||||||||| |||||||||||
Seq_2  2977  AAAGAGTTCAAAGGCAGAACGAGTTCATATAATGCAACATATATGACCGTAGGAGAAACA  3036


Seq_1  2471  AAGATGAAAGAGATAAACTCTAACACATTCAATTGCTGACCCCTATGT----AATTTTTC  2526
             ||||||||||||||||||||||||||||||||||||||||||||||||    ||||||||
Seq_2  3037  AAGATGAAAGAGATAAACTCTAACACATTCAATTGCTGACCCCTATGTGTGTAATTTTTC  3096


Seq_1  2527  TTATGCATATCTCCATTTTCCTTCTCCACTCACAAACCAATGACCACACACCATGCGCAG  2586
             ||||||||||||||||||||||||||||||||||||||||||||||||||||||||||||
Seq_2  3097  TTATGCATATCTCCATTTTCCTTCTCCACTCACAAACCAATGACCACACACCATGCGCAG  3156


Seq_1  2587  GGGATGCCCTTATCATACAGTAGTCCCATGCTACACCCATATCCTGCAAGTTTCTCCTTG  2646
             |||| |||| ||||||||||||||||||||||||||||||||||||||||||||||||||
Seq_2  3157  GGGACGCCCGTATCATACAGTAGTCCCATGCTACACCCATATCCTGCAAGTTTCTCCTTG  3216


Seq_1  2647  ATAATAACTCAAATAAACAACTTCTATTATGACGAAACACAAATAATCTGTAGTGTTTAT  2706
             ||||||||||||| | ||||||||||||||||||||||||||||||||||||||||||||
Seq_2  3217  ATAATAACTCAAACAGACAACTTCTATTATGACGAAACACAAATAATCTGTAGTGTTTAT  3276


Seq_1  2707  AAATTATCACTCGACATGTGGCAGAAATATTGTTGCTGAAGTGGTCGTCTTAAAGTGTCC  2766
             ||||||||||||||||||||||||||||||||||||||||||||| ||||||||||||||
Seq_2  3277  AAATTATCACTCGACATGTGGCAGAAATATTGTTGCTGAAGTGGTAGTCTTAAAGTGTCC  3336


Seq_1  2767  TAAGGAATCAATCATATGCCTACACCATTACAGTTATACCAAACAATGTATCATTACGCA  2826
             ||||||||||||||||| ||||||||||||||||||||||||||||||||||||||||||
Seq_2  3337  TAAGGAATCAATCATATACCTACACCATTACAGTTATACCAAACAATGTATCATTACGCA  3396


Seq_1  2827  CCACTGGGAAAAGTAATGAAGCATTGTCAGTACAGAAATGCTGATACTGTTCACAACTAA  2886
             ||   |||||||||||||||||||||||||||||||||| |||||||| |||||||||||
Seq_2  3397  CCGG-GGGAAAAGTAATGAAGCATTGTCAGTACAGAAATCCTGATACTTTTCACAACTAA  3455
```

```
Seq_1  2887  TTGATCACAATGGTGTATTGATCAAAACAGAATTGTCAGCCACTA---CTAAGCATAAAG  2943
             ||||||||||||||||||||||||||||||||||||||   ||||   ||||||||||||
Seq_2  3456  TTGATCACAATGGTGTATTGATCAAAACAGAATTGTCA---ACTACTGCTAAGCATAAAG  3512

Seq_1  2944  CACATGCCAAAACAAAGCAACAAACCAAAGGAAATTTAAACAGTTCACATTTCTTTTGGA  3003
             |||||||||||||||||||||||| ||||||||||||||| |||||||||||| ||||||
Seq_2  3513  CACATGCCAAAACAAAGCAACAAATCAAAGGAAATTTAAAAAGTTCACATTTCATTTGGA  3572

Seq_1  3004  AAAAATGATAATCTAAACAAGAGCAGTAATAACCCAAAGACCAAATACAAACCTTGTAGA  3063
              |||||||||||| || |||||||||||||||||||||||||||||||||||||||||||
Seq_2  3573  GAAAATGATAATCGAAGCAAGAGCAGTAATAACCCAAAGACCAAATACAAACCTTGTAGA  3632

Seq_1  3064  ATCATCCTTGAGCACTGAGAATGCGGGAGGCCAAATCATCATGCAAGGATGGATGTGACC  3123
             ||||||||||||  ||| ||||||||||||||||||||||||||||||||||||||||||
Seq_2  3633  ATCATCCTTGAGTGCTGGGAATGCGGGAGGCCAAATCATCATGCAAGGATGGATGTGACC  3692

Seq_1  3124  AACGTATCGATCCTTCTCTGTCATCTCATCATACACTTTACGCAGCACCCTGCGCCTGAT  3183
             ||||||||||||||||||||||||||||||||||||||||||||||||||||||||||||
Seq_2  3693  AACGTATCGATCCTTCTCTGTCATCTCATCATACACTTTACGCAGCACCCTGCGCCTGAT  3752

Seq_1  3184  ATCCAAATAGAGAGTGTATGGACCCATCTGCAAGAACACAAACTCAGCATTGTCTCCCAC  3243
             |||||| |||||||||||||||||||||||||||||||||||||||||||||||||||||
Seq_2  3753  ATCCAAGTAGAGAGTGTATGGACCCATCTGCAAGAACACAAACTCAGCATTGTCTCCCAC  3812

Seq_1  3244  CTGGGTTATACTCACAGCAGTAGTATGCTCATCTTCAACCATGACATCAGACATCTGGAA  3303
             ||||||||||||||||||||||||||||||||| |||||| ||||||||||||||| |||
Seq_2  3813  CTGGGTTATACTCACAGCAGTAGTATGCTCATCCTCAACC-TGACATCAGACATCTCGAA  3871

Seq_1  3304  AGCAGCACACATCTGACGCAAACATATGGT---ATCCACCAGCAACCAGTTATCATCCTT  3360
             ||||||||||||||||||||||||||||||   |||||||||||||||||||||||||||
Seq_2  3872  AGCAGCACACATCTGACGCAAACATATGGTGGTATCCACCAGCAACCAGTTATCATCCTT  3931

Seq_1  3361  GTGGAGCCAGATGCGAAACTGAAGTTCCTTCAAATCGATGAGGTAAACTCCGGAATCATC  3420
             ||||| ||||||||||||| ||||||||||||||||||||||||||||||||||||||||
Seq_2  3932  GTGGATCCAGATGCGAAACCGAAGTTCCTTCAAATCGATGAGGTAAACTCCGGAATCATC  3991

Seq_1  3421  TGCCCGTGAGAATACAGCATCTCTACTGTGAAATCCCATCCCATATTTATATCCAGAAGA  3480
             |||||| ||||||||| |||||||||||||||||||||||||||||||||||||||||||
Seq_2  3992  TGCCCGCGAGAATACAACATCTCTACTGTGAAATCCCATCCCATATTTATATCCAGAAGA  4051

Seq_1  3481  CCACTCCACCCCTTCTGGGAGCTGAATAGTGAAGAAACTGGAGCTCGCCAAATCCAACAC  3540
             ||||||||||||||||||||||||||||||||||||||||||||||||||||||||||||
Seq_2  4052  CCACTCCACCCCTTCTGGGAGCTGAATAGTGAAGAAACTGGAGCTCGCCAAATCCAACAC  4111

Seq_1  3541  AAGAATGTCGTTACAGGTGCCCCCAGCCATGTAGATCCTATAATTGATGAGTAGAGGTTT  3600
             ||||||||||||||||||||||||||||||||||||||||| ||||||||| ||||||||
Seq_2  4112  AAGAATGTCGTTACAGGTGCCCCCAGCCATGTAGATCCTATCATTGATGAGGAGAGGTTT  4171

Seq_1  3601  TGGCCTCCATAGCGGATGGGGGAGTTGTGTCATGGCTTTGGTAAGCATGACCCAGACACC  3660
             |||||||||||| |||||||||||||||||| |||||||||||||||||||||||| |||
Seq_2  4172  TGGCCTCCATAGTGGATGGGGGAGTTGTGTCGTGGCTTTGGTAAGCATGACCCAGAAACC  4231

Seq_1  3661  ATCTCACAACATGTGTACCTTCGCGGTAGATCTTCCAACAATGACATTGATCTCCAT---  3717
             ||||| |||||||||||||||||||||||||||| ||||||||||||||||||||||
Seq_2  4232  ATCTCGCAACATGTGTACCTTCGCGGTAGATCTTGCAACAATGACATTGATCTCCATACA  4291
```

```
Seq_1  3718  -----------TGCCATCCACATGTACGATAAGCAGCCGTCGCCTCCTGTGGAAAGGAAG  3766
                        |||||||||||||||||||||||||||||||||||||||| |||||||||
Seq_2  4292  TTGATCTCCATTGCCATCCACATGTACGATAAGCAGCCGTCGCCTCCTGTTGAAAGGAAG  4351


Seq_1  3767  CAAGAGAATGTAGGTTTATGGCTGTCGTCTGTCATGGCACGTGTTGGTGCTGGGAGGACC  3826
             |||||||| ||||||||||||| ||||||||||||||| ||||||||||  ||||||||
Seq_2  4352  CAAGAGAAGGTAGGTTTATGGCCGTCGTCTGTCATGGCATGTGTTGGTGCCTGGAGGACC  4411


Seq_1  3827  ACCACGCCTCTGTCAGGGAACAGCGGGTTGTGCACTTCATATGTGTATTTGTGGTTCTGG  3886
             ||||| ||||||||||||||||||||||| |||||||||||| |||||||||||||||||
Seq_2  4412  ACCACACCTCTGTCAGGGAACAGCGGGTTATGCACTTCATATCTGTATTTGTGGTTCTGG  4471


Seq_1  3887  AATCTGGAGATGAAGACACTGTCGTTCCGGCATTGCTCAATCATTATTCTGTTGATGGTG  3946
             ||||||||||||||||||||||| ||| ||||||||||||||||||||||||| |||||||
Seq_2  4472  AATCTGGAGATGAAGACACTGTCATTCTGGCATTGCTCAATCATTATTCTGTCGATGGTG  4531


Seq_1  3947  TCCAAGCTTAAGGTTGCCATGCGGCGGAGGACGGAGGCGAGCTCCGGCTCTTGGGTCTGG  4006
             |||||||||||||||| ||||||  ||||||||| |||||||||||| ||||||||||||
Seq_2  4532  TCCAAGCTTAAGGTTGACATGCGATGGAGGACGGTGGCGAGCTCCGGGTCTTGGGTCTGG  4591


Seq_1  4007  GTCAGCA--ACGGCACGAAGCGAGGAAGAACCTTGGCCGACTCGGGTGTATCGGTAAAGA  4064
             |||||||  |  |||||||||||||||||||||| ||||||||||||||||| |||||||
Seq_2  4592  GTCAGCATCA--GCACGAAGCGAGGAAGAACCTTTGCCGACTCGGGTGTATCAGTAAAGA  4649


Seq_1  4065  ACACGCCATTGACGTACAAGCCGAGGAGGCGGGGCGGGTGGCGCTGGCGGAATTGGCGGA  4124
             ||| |||||||||||||||||||||||||||||||||||||||||||| |||||||||||
Seq_2  4650  ACATGCCATTGACGTACAAGCCGAGGAGGCGGGGCGGGTGGCGCTGGCAGAATTGGCGGA  4709


Seq_1  4125  GGAAGGCCCGGTTGGCAGCGTTGTGGAACCAGCGCTTGCAGACGAGCGCGGCGCGGGCGA  4184
             |||||||||||||||||||||||||||||||||||||||||||||| ||||||| |||
Seq_2  4710  GGAAGGCCCGGTTGGCAGCGTTGTGGAACCAGCGCTTGCAGACGAGCGTGGCGCGGACGA  4769


Seq_1  4185  GTGTGGTGGGGAGGCCGACGCGGAGGAGGTT---------------GTCGTCGTCGAGCA  4229
             | ||||||||||| |||| |||||||||| |               ||||||||||||||
Seq_2  4770  GGGTGGTGGGGAGACCGATGCGGAGGAGGATCTCGCGGAGGAGGTCGTCGTCGTCGAGCA  4829


Seq_1  4230  CCTTGGACACGAGGGCCGCGGCCGCGGCCGGCGAGGGCAG-GAAATCCATTGTTGGCCGA  4288
             ||||||||||||||| |||||||||||||||||||||| | | |||||||||||||||||
Seq_2  4830  CCTTGGACACGAGGGTCGCGGCCGCGGCCGGCGAGGGC-GGGGAATCCATTGTTGGCCGA  4888


Seq_1  4289  TCT-TTGAATAAGGAAGAGAAATCAAAATCTTACACTTATGGTATCATTCCCCTCCTCTG  4347
             ||| ||||||||||||||||||||||||||||||||||||||||||||||||||||||||
Seq_2  4889  TCTCTTGAATAAGGAAGAGAAATCAAAATCTTACACTTATGGTATCATTCCCCTCCTCTG  4948


Seq_1  4348  CTGCTGGAACAAGGAAAGGTAGGAGTTTAAGAAGAAAAGTCAAGCTAGGGTTCCGTGCAA  4407
             ||||||||||||||||||||||| ||||||||||||||||||||||||||||||  |||
Seq_2  4949  CTGCTGGAACAAGGAAAGGTAGGAATTTAAGAAGAAAAGTCAAGCTAGGGTTCCGCACAA  5008


Seq_1  4408  GAAACGAAATTGGGGCAAAATAAGACTTACCTGGAATCGCGAGAGGGGAAGGTGGATTTG  4467
             |||||||||||||||||||||||||||||||||||||||||||||||||||| |||||||
Seq_2  5009  GAAACGAAATTGGGGCAAAATAAGACTTACCTGGAATCGCGAGAGGGGAAGGCGGATTTG  5068


Seq_1  4468  TGGGGGCGCAGGCACCGACCGCCGTCGGGGGTTCAATTCGGACCGCCGGCAGAAGTCCCG  4527
             |||||||| ||||||||||||||||||||||||||||||||||| ||||| ||||||| |
Seq_2  5069  TGGGGGCGTAGGCACCGACCGCCGTCGGGGGTTCAATTCGGACCTCCGGCGGAAGTCCTG  5128
```

```
Seq_1  4528  TGCCAGGGTGGATGCCGGTCTGGCTCGGTCCGAGCTCGCCGCGAGCTGTGCGGCAGCGGC  4587
             | |||||||||| |||||||||||||||||| |||||| |||||||||||| || || ||
Seq_2  5129  TCCCAGGGTGGACGCCGGTCTGGCTCGGTCCAAGCTCGTCGCGAGCTGTGCAGCGGCCGC  5188

Seq_1  4588  TGCCTAGGAGAGTCAGGGTTCGTAGGAACTGAGAAGCAGTTAATTTTGACCTAGGCAAAT  4647
             |||||||||||||||||||||||||||||| || ||||||||||||||||||||||||||
Seq_2  5189  TGCCTAGGAGAGTCAGGGTTCGTAGGAACTAAGTAGCAGTTAATTTTGACCTAGGCAAAT  5248

Seq_1  4648  GGGACTGGACTGGGCTCGAGGCATCTATGGGCATTGAAAACTGAGCGTGGGCCGCCAGTG  4707
             ||||||||||||||||||||||||||||| ||| ||||||||||||||||||||| ||||
Seq_2  5249  GGGACTGGACTGGGCTCGAGGCATCTATGAGCAGTGAAAACTGAGCGTGGGCCGCTAGTG  5308

Seq_1  4708  GGCCAAAATGGCCAGCTCGCCGAGCTGGACCGAGCTCAGCTCGGCTCGGCCTGAAGATGT  4767
             ||||||||||||||||||||| ||||||||||||||||||| ||||||||||||||||||
Seq_2  5309  GGCCAAAATGGCCAGCTCGCCAAGCTGGACCGAGCTCAGCTTGGCTCGGCCTGAAGATGT  5368

Seq_1  4768  GACGAGCCGTTTTCGTGGGCTAGGCTTGGTCTAAAAAAATCAGGCTCGGGGCGAACTGGC  4827
             |||||||||||| |||||||| |||||||||||||||||||||||||||| ||| |||||
Seq_2  5369  GACGAGCCGTTTCCGTGGGCTCGGCTTGGTCTAAAAAAATCAGGCTCGGGCCGAGCTGGC  5428

Seq_1  4828  TCGGCTCGCCACGGGCTCGGGCCAGCTCACCGAGCCCAGCTCGAACGTCCACCCTGAGTC  4887
             |||||||||||| ||||||||||||||| |||||||||||||||||||||||||||||||
Seq_2  5429  TCGGCTCGCCACAGGCTCGGGCCAGCTCGCCGAGCCCAGCTCGAACGTCCACCCTGAGTC  5488

Seq_1  4888  CCATCAATGCGGCGCTTCGTGGTGGTGTGGAGCAGAGCAG-----GCAGCCCACCAGGAC  4942
             || |||||| |||| |||||||||||||||||||||||||     |||||||||||||||
Seq_2  5489  CCGTCAATGTGGCGTTTCGTGGTGGTGTGGAGCAGAGCAGAGCAGGCAGCCCACCAGGAC  5548

Seq_1  4943  AGGGTGAGAAAAGGTAATCTCCCATGGAATTCATTCACATGACACCTAGCCGTACTCCAT  5002
             ||||||||||||||||||| ||||||||||||||||||||||||||||||||| ||| ||
Seq_2  5549  AGGGTGAGAAAAGGTAATCACCCATGGAATTCATTCACATGACACCTAGCCGTTCTCGAT  5608

Seq_1  5003  TTCTATCTGCCTGTTTCCAAAAAAAA-ATCACTGTGAACTTCAAAGATATTTTTCAGAGA  5061
             |||||||||||||||||||||||||| |||||||||||||||||||||||||||||||||
Seq_2  5609  TTCTATCTGCCTGTTTCCAAAAAAAAGATCACTGTGAACTTCAAAGATATTTTTCAGAGA  5668

Seq_1  5062  AATAAAATAAATAAATCCTGCGCACAGTTTGAGATCCCAAACTAGAATATTTTCCAACAT  5121
             ||||||||||||||||||||| ||||||||||||||||||||||||||||||||||||||
Seq_2  5669  AATAAAATAAATAAATCCTGCACACAGTTTGAGATCCCAAACTAGAATATTTTCCAACAT  5728

Seq_1  5122  GCAAAGAATAACAGAAATAGCAAGGAGGATGTCATTACATGCTTAATCAGGAAGCTTGTT  5181
             |||||||||||||||||||||| |||||||| ||||||||||||||||||||||||||||
Seq_2  5729  GCAAAGAATAACAGAAATAGCAGGGAGGATGCCATTACATGCTTAATCAGGAAGCTTGTT  5788

Seq_1  5182  ATTTAACTGTAACCACTCTGAAAATACTTCTCTTGCCATCAGCACCTGCTCCTTTCTACA  5241
             ||||||||||||||||||||||||||||||||||||||||||||||||||||||||||||
Seq_2  5789  ATTTAACTGTAACCACTCTGAAAATACTTCTCTTGCCATCAGCACCTGCTCCTTTCTACA  5848

Seq_1  5242  GTAATTAACAGGCTCAGTGATTGGCTTCACATTGATTAAAGCCACACAACGGGCAAGTCA  5301
             |||||||||||||||||||||||||||||||||||||||||| ||||||||||||||| |
Seq_2  5849  GTAATTAACAGGCTCAGTGATTGGCTTCACATTGATTAAAGCGACACAACGGGCAAGTTA  5908

Seq_1  5302  AGATTACAAGATCCTCCCATTTGGTCCAAGTACACAGCCCCAGTCCCGACTGACAAGTCA  5361
             |||||||||||||||||||||||||||| ||||||||||||||||||||| |||||||||
Seq_2  5909  AGATTACAAGATCCTCCCATTTGGTCCACGTACACAGCCCCAGTCCCGACAGACAAGTCA  5968
```

```
Seq_1  5362  ATAACTGCTCTTCTTGACAGTAAGATGCTGAAAAGCATGGGACGAGCAATTTGTGATGAC  5421
             || ||| |||||||||||||||||||||||||||||||||||||||||||||||||||||
Seq_2  5969  ATGACTCCTCTTCTTGACAGTAAGATGCTGAAAAGCATGGGACGAGCAATTTGTGATGAC  6028

Seq_1  5422  AATGACTTTGAACTGCCAATAAATGACATTTTGGGACTCGTTTGGACAGTCCAAGCGCCT  5481
             |||||||||||||||||||| | ||||||||||||||||  |||||||||||| ||||||
Seq_2  6029  AATGACTTTGAACTGCCAATCAGTGACATTTTGGGACTCTGTTGGACAGTCCAGGCGCCT  6088

Seq_1  5482  AAAGCTCATAAAATCATGCGGAGAGACTCAAAAGAC-GAAGACCAAGGCTTCGAGTCTTT  5540
             |||||||||||| |||||||||||||||||||| ||  |   | |    | |||||||| 
Seq_2  6089  AAAGCTCATAAA-TCATGCGGAGAGACTCAAAAAACGAAGACCAAGACTTCCGAGTCTTC  6147

Seq_1  5541  GGATCCAGGTAGCTTCCGCTGTGGTGAGGCCACCTTTCTGGCTATCACATGCATCCGCTC  5600
             ||||||||| |||||||||||||| |||||||||||||||||||||||||||||||||||
Seq_2  6148  GGATCCAGGCAGCTTCCGCTGTGGCGAGGCCACCTTTCTGGCTATCACATGCATCCGCTC  6207

Seq_1  5601  CGTATCTGCAGGTCTCCTTGATCTGCAAGCCTTCCTGACTGCAACACCTACAGTTTTTTC  5660
             ||||||||||||||||||||||| ||||||||||
Seq_2  6208  CGTATCTGCAGGTCTCCTTGATCCGCAAGCCTTC--------------------------  6241

Seq_1  5661  CTACCTAGCTTACCGAATATTCCAAGACCTAAATCATCAAGTGATTCTTCTCGCAGCATT  5720

Seq_2  6242  ------------------------------------------------------------  6241

Seq_1  5721  CTTATTCGTGCAGGCACCGCACCTTGGGTTCTGCCAATTTCTTCTTCATCGTGCTTGCAG  5780

Seq_2  6242  ------------------------------------------------------------  6241

Seq_1  5781  CTGCAGCATCGTTGCTGGCCATGCAGGGATCCAACTATGGCGTCTATCCCATTGTCAACC  5840

Seq_2  6242  ------------------------------------------------------------  6241

Seq_1  5841  TCCCAACACTGTTGATCAATAAAAAACCTCCCTCCCAAACGGCTTGAGCTACATGGACGG  5900

Seq_2  6242  ------------------------------------------------------------  6241

Seq_1  5901  TACAACCGTCCGTGCCATCGTCCTCCGTTCACCAAGGGCACGGCCTTTTTCTTTTGATGG  5960

Seq_2  6242  ------------------------------------------------------------  6241

Seq_1  5961  GTAGTCCTTTGCTGAAGGGTTCTTCTGTGCCTCCCTCTGTGATGTCTTCCTCTTTTCTGT  6020

Seq_2  6242  ------------------------------------------------------------  6241

Seq_1  6021  CTTCATTGTAAGCACTGACAGTGGCGGCTGGTTCTACGACATGGCAACTGGACCGCAGGT  6080

Seq_2  6242  ------------------------------------------------------------  6241

Seq_1  6081  TATCTGGTTGGCCAACCGGGCTGGCCCTGTCAGGGAGAACACCACCCTTGAGCTTACTAG  6140

Seq_2  6242  ------------------------------------------------------------  6241

Seq_1  6141  GAATGGCAACCTCATCCTCCGTGATGCCAATGGGATCATGGGAGGATGGTCTGGTCTAGT  6200

Seq_2  6242  ------------------------------------------------------------  6241
```

```
Seq_1  6201  GGCACTTCAGACAAATCCATGATTGGCATGGAGATCACGAAGTTCGGCAACCTGGTGCTA  6260

Seq_2  6242  ------------------------------------------------------------  6241


Seq_1  6261  TTTGATCAGAGGAATGCAACTGTGTGGCAGTCATTTGATCATCCGACTGATTCATTGGCC  6320

Seq_2  6242  ------------------------------------------------------------  6241


Seq_1  6321  CCCGTGCAATCACT  6334

Seq_2  6242  --------------  6241
```

BdindelWSU_16, upstream

>Bradi5g00810

GTCAACGGGTGTCACCCATGGGGTAAGCCAATTAGTAATGGGCAGGGCATGAGTACAACTTGGCGCCCATGGGTTGCCTAATTATTT
TTTTTAATAATGTTTATAAGCCAAACTACAATTATGTAATTGCACATGTAATTGCACTTTTTCATGTGAATTATGATCTTATATTGATGC
ATGCATAATACATATATTATCATTTCTTTCATGTTTATGATGTTCTTATATTAATGCACAACTAGAGTGACCCACACGTTTGCACGACTA
GAGTTTCTACAGATTATCACCATCTTTGAGCCGGGGGTTCATCACCTTTCGTTTTGGGAAACTAAGCTCCCTGATATGGATGGCAATG
GGTACTCACTACCCGTGCACTTACCCTCTAGGTATTTTTGTGTTCTTGTTACATGCTCATGCCGGCAAGGTTTTACATGGTCTTAGCACA
TGCTTTACAAAATGGACTATCTCTGATACATTTTCATAACTTAGGGTTTTAACTTTGTGATTTGAAGCATCGGACCAACTTTTGTAATTT
TTTTAGTTGATATTTTTATTGGGCCTTCATTTAGCATTAATAACGAGTAATGTGAATGAAACAACAGCAAAATTGAAATAATTCTTAAA
GTTGTCAAAACTTATTTATTTATTGGCATGAAGCCATACTTTTGTATACAAGATTGAGATAAATAAGTCAAGTCAATACACTAAATTTT
AAATCGGTACACACTTCACAAATACACTTTGGGTATATAAGAATGGCCCGGCAAAGGTTTCTTCCTTGCAGATCTCCACCAATCAGCA
ATACAAATAAGCAGCTACGGGTGGTCTGAACTTAATTGGGTAAACTCGCCTTGTCTCTAAAGTCCGACGAAGATCTGGCCCACTCTCG
CGCCCCTCGTTCTTTCCGGATGTCTGCTCCCTCACACGCGACAACTGTCGGTTCAGAAATCTTGGTCATAATAACCGTTGATAACTTAA
CTGAGTGTGTATTTTTTCCAAAATCATTAAATCATTATTATCATTAGAATAAATAAATAATTGGTCGGTGGTGCGTACGTGGACAAACA
AATTGCGTCCGAAAAGAACACGTCTCGTCGTCCGACATCGATCAGAGTCCAAACCGAGACTGACTAGGGTTTGGTTGATCGACTATA
TATATCTGAACCCAGAGCTAGCCGCCGCCTCCAGTCCAGATCGATCTATAGAAAGAGAAGGATATATCGAGATCCGGCGAGATGAAG
CATGATAGGGCTGAGGAGGTTGCGTCCGACGGCGACGGAGAGATCAAGTCGCAGAATCCGGCGGAGGCAGCAGAAGCAGCGCCG
CAAACAGAGGGCCTGTCGTCCAAGAGAGCATCATCCGCCGGACCGAGCGGCGACGGCGACGCCATGATCAAGTCGCAGAAGGTGG
CGGAGGGCGACAAGGCGGAGGCGGGGAAGGCGAAGAGGATCGCCAAGGTGCCCCAGAAGTACATCGATCTGCTGCGCAATGGCG
GCTTCCCCAGGCTTCCCACCTTCGACGGCCCCAGCAAATCCCGATCTCCTGCCGTCCAGGCACGCGTGGCTCACTGCAAAGCTCTGGT
GGACGAACTCCGAGCCTACAACGCCGGCATCCTGGCGCAGTACGACGAGCTCGGCCACGCCTACCATGAGGTCGAGGAGGAGCCGT
GGATCGACGAAGCCATGGCCCGCCAGCTGGCCAGGAAGAAGCCGATCCCGCCGCCGGCTTCATCTTGATCGAAGCAGGAATTTCAT
CAAGAACATGCTTATCCATCCCCTGCCAAAACTATAGAGTTTATACGCGCGAGAGTCGACTTTGTATAATTAGAATTTAAGTTAATGTC
CGTGATGTCCATCTGGGACAAGTTAATGCTCTGGTAATCGATAGTATAATCTGTCTCACAGTCCGGTTCAACTTGCCCTGCAGATGGT
CATAAAAGACCTCATAATCTGTTAGTTAATGATCGTGACTTCATGAATAACTCAAGCTATTAGTACAGTTTCTTTTCCTTGCGTCAGTG
ACTTAATCTTAGATGAATTTAGCTGTGTTCATGTCGTGGTGGTATCACGGAACCGGAGGAGAGAGGACGTAAAGGGAACCACGCAC
GATTCACACAAGAACACACAGATTTACCCAGGTTCAGAGCCCTCTTGTCGAGGTAAGACTCTTACTCCTGATTTGTTGTGTTAGCCGA
GATAGGCAAGGTCTACAATGGCGCTCCTTGAGCTGTATTCTTGAGGAAAAAGAAGAAAGGGGAAACCTTAGATGCCTAGAATGCTC
CGTCCATCTCTACAGAGGGGTAAGATTCTATTTATAGGGGGTTACTTGGGCCTTGCCAGGCACGCGGCTTGATTCTCTCCAGTCAGTA
CCGCAGGGGACAAGACAGCTTTATTTTTCCCTGCCAGCCTGCAACGGGTACAGCTATCCTCTGCCGGCTTCTGTCATAGATTCTCTTTC
GGTGCTTCTCTAGCGTGGTCTCCTGACACCGGTACGCAGGCGCTGACTGCTTTTCGGGAAATAATGATGGCGTTGCTTTACTTTGCAC
TGCGTGGTCAGGATAAGACCGTTGAGGGATCTCGGCTATCGCCGAGACCAATGTGCCCATCCTTATCCTGCAAACTCATAAGACAAG
ATAGGCCTGCCGGCATACACCCGGGATGCCGGCTTAGCGTTAGTTGTACTTTACGATGCCGACATACGTAACTATGCCGGCTTTGCCT
CCGTCATCTCGGGTAGAGCTGTGTCGCCATGACCCTATCCGAGGTCATCCCCCCCGACAGTTCATGTATATGCAATGTGATACCTCCG
ACACTTGCTACGGAAGATGCGTCAGAGGTGGCGCATCACCACTTGACGCATAGTTTTTGACGGTTCCCTGTTTCGTCAGAAGAGAGG
GTTTGAGGGTCGTCAAAGATAAACTGTACTGTAGTAGTGAGTAAACATCACGGGATAGAGCTTACAAATCAACAAGAAACTAAAAAT
CAACAAAGGTTTCTGAACTCTTGATCTGTTACAAGGAATCTCACAAACAGCTAAAGTAATCAACAGTTGTATGTCTCAGATGTCTCAG

ATCACAGGAACATCAAGATAAGCAGGAAGCTGAGAATTAAAGGAAGAGAGCGATTCGGTTTTTTTCTCAGTTAAGTCACTGTGTGGG
CTGTCTTTCTTCTTATGGCAATGAGTTGGTGACATTAATTTTTTTTGTCAAAGTCTCAATGGTTGATGGACTTATGACACGTCCATCCTA
TCTAATTCCCTATCTACTATGCACTTTAATCCTCTAGAGATGATGTATGACAGACTCTTTGATCCTCCGGTATCTTCAGTTCATTTGGAG
TTGGAAAGTATGTCGCCATGACCCTACCCGGGGTCATCCCCCGACAGTTCATCATGTATATGCAATGTGATACCTCCGACACTTGCTA
CGGAAGATGCGTATGAGGTGGCGCATCACCACTTGACGCATAGTTTTTGACGGTTCCCCGTTTCGTCAGAAGAGATGCGTCAGAACA
CCTTTATGATCACTCAGTTACGAGATGACGGTTGATATTCACAAAGTATTCTTCCGGTACTAGAGAATGACATGATCTCATGGTCTAA
GGAAATGATACTTGACATAATAAAAGTTTTAGCAATTTAAACTTAAGTGACACGATCAAAAGTTATGTTTAGGTTTGGGTCTGTCCAT
CACATCATTCTCCTGATGATATGACCTCGTTATTAAATGACAACACATGTCTATGGTTAGAAAACCTTAACCATCTTTTAACCAACGAG
CTAATCTAGTAGAGGCGTATTAGGACACGGTATTTATTTATTTACCATACATGTATTTAGTTTCATGTTAATCCATGCAAAGCACCTTA
ATCCGGCGTTGCAAGGCGAACGCGCGTCGAGGAAGATTCTGTGCGAGCACCTGCGCGGCGCGGCGGAGCCAGGGGAGGAATCTTT
CTTCTAGTGTTGCGGATTACTCCGCTTCCATTTTCAAGGACAGACCACGAGAAGGAGGCCATTGAAAAAAAGAGAGCAGCTATGAGA
AACATGCTGCTACGAGTGTGTACAAGGGTCAGTATAGTTCTCCATAGAGAATTTTACTTTATGTAAGCCAGATTAAGAGTAAATTCCA
CTTTTAACCTCAAGTTGCATTTTATTGACAACTTTTACCCCATTTAGTGAGAGTTCCAATTTTTTTACCCCATTTCAGAAAAGTTCGTCCA
CAAATTACCCATTTAAGATGTTTGGTGAATTCCCTTCGTTCTTGCTTGCGGGGCTCCCTTGCGGGGTTGACGTGGCGTGGCCAACGCG
GATACGTATTTGGCTCCCACGTGCGTCCTTGTTGCCGGTTCGCAGACCGGCGACCGAACCAGTCAAGCTAGCGAGGGCTTGGGCGAT
GGCACGACCATGGACAAAGCAGAGGTGGCCGGCGCGGAGCAGCAGGCCGACTGGAGGAGGTCGGGCTAGCGAGAGAGAGATGG
CAAACAACAAGGGACGCGCATGGCGGGACCCAATACGTGTCCGAGCTGGCATGCCAGGTCGGCCCTACAACTGGGGCCCAACGGTC
AGAACGATGGAGATTTACTGAAAAATCTAAAATGGGGTAATATGTGGAACAACTTTTCTTAAATGGGGTAAAAAAATGAAATTCCCA
ATAAATGTGGTAAAAGTTGTCAAAAAAAATTGATGGTAAAAACTAGAATCTACTCACAGATTATTTAGCATTGTACCATTGCTTCCCTG
GTCAGATTTCCTTTGCTACCTAATCTTGTGATCTACATGTGAAATTTAAGCAGAACCTACATAAATGACCAAATGCGGTCCAAGTACCG
AGCTAGCAAGTCTCATGGGCCAATCCACTACAGGCCCACATATATTTTCTAGTTTCTCTTCTGAAAATAGAAAAGAACATTTTACAATT
ATAATTTAATGAAGCCTTCCAGAATAAGTAGTCCATTGCTGGGATGGATTACCAAATCACAATTTCACAACTCACAAGGCATTGCCAC
TTTACTATTTGTGCTGGTCACTGTATTGAGAGTTTCGGATGGTGAAAGAGGAGAAAATTGAAGGTGGAGCGTTGTTTTGGTGCTTTAT
ATCTTAGCTCACTATAAGAATCCACCGCTGACTCTTACGTTGCTGGATATTATAATGCAAACTGCTAGAAGACCTAGCAATAAGTTGTT
GGACTGATATGTTTTTTGTCACATGCACAATTAGACTTGTAGGAATGGTACTATTTTCTTGTCCATCCGAAAGTAAGCCAAGATTTTTT
TTTGAAAGTGTAAGCCAAGATTTTTGTGAAAGTGAGCTAAGATATTTGAGTGAATCTGGAGAGCTCAGTTGTGAAGCATGTATGATA
TGATTGGTCACAGTTAAGCTGCCATTTTCAGTAAATTTTGGAGCTCACGGCTGCTTGTGATAAATAATTTGATGGTCATTTTCTAATTT
CTTGTAATAATAAGTTGATGTTTATTTATGTTATGCTCACTCCTGGCCGGCCTGCTTTTTGCTGCATTTCAGTATTCTTTTTGTGGATAAT
ATTTTTCTTGTAATAAAAACTGTACTACAACTTAGGAAGAACAGAGGGAGTAATTGTTCACATCGGAGGTTCGCTTGCAAGTTGCATG
CGGCTCCCTGATTTTGACCAGTGCATGGTCCTAGCAGCCATAGCCCACCAAACGCCTAGAATTGACCAACAAGCTACTAAGCTTTGTT
GACCATATTGTTCAGATTAGCAGCCATTACAGTTAGGTCTCTGTTTCTTCACTACTCAAATAGGATAATGAAGGGATTACTAGTATGG
ATAGGACGTCGACAGCTAAAAAGAAAGATATGTTAAGGGCGTAGCCGTGCTACAATTGGCAAGTTGCATACATGCAGATATGTCCGC
AAGACAGGATTTGATGCGAGACGGCGATTGATCTGCGATGGCTTTGCAAGTTGCAGATGCAGAATTTGGAGCAGCCTAGCCTAGGC
AGTCCAGTTGACCAATAAAAATCCCCTTTGTTGGCCACATGATCCAACTCTGCTCAGCTTACTTAAAACGGAGAACTGGACGTGGAGC
AATCTCAATCTCACATCATTTCATATAATACTACATATAGATTCCATTCATGTGATGTATCAATGGAGACCTTCATTTCACTTGCAAGTA
CGCCTGACCTCCATTAGACCAAAACTAACTCAGAAAGCTTGGAGCATAGAGCCAGGCGCAAGAAAGAATCA

>BdiBd21-3.5G0007000

GAATGTATCTAGACATGACTTAGTGTATAGATGCATTCAAATTTTGTCAAAGTTGAGACATCTTTTGTTGGACGGAGGGGGTATAATG
TGTTTGGTATCGTTCATTCGTTCTAATAAAAATATGGCGGTGCGCATAAGACCTGGAGGTTTTGCCTTCATTCTTTGAAAAAAATAAAT
TTGCAGAGTGTCATCATCAATGTTTGATTTGGGCATGTTCACATTTTTATCCATTGCATTTTGTTTTCTGAACTCGCAAGTTATTGTACT
CCCTCCGTCCAACAAAAGATGTCTCAAGTTTGTCCAAATTTGGATGTATCTAGACATGACTTAGTGTATAGATGCATTCAAAATTGGTC
AAAGTTGAGACATCTTTGTTGGACGGAGGGAGTACTCAATATTCATTGATTAATTGTGACAACTCAACAAAAAAATTCTGATAGCAAT
CTGCTCATAAAACCACCCATGGATACCTGACCTGAATGGGGGTATGGATCGTCGTTTTCGTCAACGGGTGTCACCCATGGGGTAAGC
CAATTAGTAATGGGTAGGGTATGAGTACAACTTGGCGCCCATGGGTTGCCTAATTATTTTTTTAATAATGTTTATAAGCCAAACTACA
ATTATGTAATTGCACATGTAATTGCACTTTTTCATGTGAATTATGATCTTCTATTGATGCATGCATAATACATATATTATCATTTCTTTCA
TGTTTATGATGTTCTTATATTAATGCACAACTAGCAGAGTGACCCACACGTTTGCACGACTAGAGTTTCTACAGATTATCACCATCTTT
GAGCCGGGGGTTCATCACCTTTCGTTTTGGGAAACTAAGCTCTCTGATATGGATGGCAATGGGTACTCACTACCCGTGCACGTACCCT
CTAGGTATTTTTGTGTTCTTGTTACATGCTCATGCCGGCAAGGTTTTACATGGTCTTGGCACATGCTTTACAAAATGGACTATCTCTGA

TACATTTTCATAACTTAGGGTTTTAACTTTGTGATTTGAAGCATCGGACCAACTTTTGTAAATTTTTTAGTTGATATTTTTATTGGGCCTT
CATTTAGCATTAATAACGAGTAATGTGAATGAAACAACAGGAAAATTGAAATAATACTTAAAGTTGTCAAAACTTATTTATTTATTGG
CATGAAGCCATACTTTTGTATACAAGATTGAGATAAATAAGTCAAGTCAATACACTAAATTTTAAATCGGTACACACTTCACAAATACA
CTTTGGGTATATAAGAATGGCCCGGCAAAGGTTTCTTCCTTGCAGATCTCCACCAATCAGCAATACAAATAAGCAGCTACGGGTGGTC
TGAACTTAATTGGGTAAACTCGCCTTGTCTCCAAAGTCCGACGAAGATCTGGCCCACTCTCGCGCCCCTCACTCTTTCCGGATGTCTGC
GCCCTCACACGCGACAACTGTTGGTTCAGAAATCTTGGTCATAATAACCGTTGATAACTTAACTGAGTGTGTATTTTTTCCAAAATCAT
TAACTCATTAAATTATTATTATCATTAAAATAAATAAATAATTGGTCGGTGGTGCGTACGTGGACAAACAAATTGCGTCCGAAAAGAA
CACGTCTCGTCGTCCGACATCGATCAGAGTCCAAACCGAGACTGACTAGGGTTTGGTTGATCGACTATATATATCTGAACCCAGAGCT
AGCCGCCGCCTCCAGTCCAGATCGATCTAGAGAAAGAGAAGGATATATCGAGAACCGGCGCGATGAAGCATGATAGGGCTGAGGA
GGTTGCGTCCGACGGCGACGGAGAGATCAAGTCGCAGAATCCGGCGGAGGCAGCAGAAGCAGCGCCGCAAACAGAGGGCCTGTC
GTCCAAGAGAGCATCATCCGCCGGACCGAGCGGCGACGGCGACGCCATGATCAAGTCGCAGAAGGTGGCGGAGGGCGACAAGGC
GGAGTCGGGGAAGGCGAAGAGGATCGCCAAGGTGCCCCAGAAGTACATCGATCTGCTGCGCAATGGCGGCTTCCCCAGGCTTCCCA
CCTTCGACGGCCCCAGCAAATCCCGATCTCCTGCCGTCCAGGCACGCGTGGCTCACTGCAAAGCTCTCGTGGACGAACTCCGAGCCTA
CAACGCCGGCATCCTGGCGCAGTACGACGAGCTCGGCCACGCCTACCATGAGGTCGAGGAGGAGCCGTGGATCGACGAAGCCATG
GCCCGCCAGCTGGCCAGGAAGGAGCCGATCCCGCCGCCGGCTTCATCTTGATCGAAGCAGGAATTTCATCAAGAACATGCTGATCCA
TCCCCTGCCAAAACTATAGAGTTTATACGCGCGAGAGTCGACTTTGTATAATTAGAATTTAAGTTAATGTCCGTGATGTCCATCTGGG
ACAAGTTAATGCTCTGATAATCGATAGTATAATCTGTCTTACAGTCCGGTTCAACTTGCCCTGCAGATGGTCATAAAAGACCTCATAAT
CTGTTAGTTAATGATCGTGACTTCATGAATAACTCAAGCTATTAGTACAGTTTCTTTTCCTCGCGTCAATGACTTAATCTTACATGAATT
TAGCTGTGTTCATGTCGTGGTGGTATCACGGCAGATGCCATAAGATGGTTTAACTTGGAGCCGATGGACGAAGGATTAACCGGAGG
AGGGAGGACGTGAAGGGAAACATGCACGATTCACACAAGAACACACAGATTTATCCAGGTTCAGAACCCTCTTGTCGAGGTAAGAC
TCTTACTTCTGATTTGTTGTGTTAGCCGAGATAGGCAAGGTCTACAATGACGCTTCTTGAACTGTATTCTTGAGGAAAAAGAAGAAAG
AGGAAACCCTAGATGCCTAGAATGCTCCGTCCATCTCCACAGAGGGTAAGGTTCTATTTATAGGGGGTTCCTTGGGCCCCGCCAGGC
ACGCGGCTTGGTTCTCTCCAGGCAGTACCGCAGGGGACAGCTTTACTTTTCCTTGTCAGCCTGCAACGGGTACATCTATCCTCTGCCG
GCTTCCGTCATAGATTCTCTTTCGGTGCTTCTCTAGCGTGGTCTCCTGACACCGGTACGCAGGTGCTGACTGTTTTTCGGGAGATAATG
ATGGCGTTGCTTTACTTTGCACTGCGTGGTCAGGATAAGACCGTTTAGAGATCTCGGCTATCGCCAAAACCAATGTGCCCATTCTTATC
CTGCAAACTCATAAGACAAGATAGGCCTGCCGGCATACACCCGGGATGCCGGCTTAGCGTTAGTTGTACTTTACGATGCCGGCATAC
GTAACTATGCCGGCTTTGCCTTCGTCATCTTGGTTAGAGCCGTGTCGCCATGACCCTACCCGGGGTCACCCCCCCCCCCCCGACAGTT
CATGTATATGCAATGTGATACCTCCGACACTTGCTACGGAAGATGCGTCAGAGGTGGCGCATCACCACTTGACGCATAGTTTTTGACG
GTTCCCTATTTCGTCAGAAGAGATGCGTCAGAACACCTTTATGATCACCCAGTTACGAAATGACGGTTGATATCTACAAAATATTTTTC
CGGTACTAGAGAATGGCATGATCTCATGGTCTAAGGAAATGATACTTGACATAATAAAAGCATTATTAATTTAAACTTAAGTGACACG
ATCAAAAGCTATGCTTAGGTTTGGGTCTGTCCATCACATCATTCTCCTAATGATATGACCTCGTTATTAAATGACAACACATGTTTATG
GTTAGGAAATCTTAACCAATTTTTAACCAACGAGCTAATCTAGTAGAGACGTATTAGGAACACGGTATTTATTTATTTATCCATACATG
TCTTTACTTTCCTGTTAATCCATGCAAAGCACCTTAATCCGGCGTTGCAAGGCGAGCGCGCGTCGAGGAAGATTCTGTGCGAGCACCT
GCGCGGCGGGAGGAATCTGTGGTACTTGGTTCCCTAGGACGTGTTTCTTCTAGTGTTGCGGATTACTCCGCTTCCATTTTCAAGGACA
GACCACGAGAAGGAGGCCATTGAAAAAAAGAGAGCAGCTATGAGAAACATGCTGCTACGAGTGTGTACAAGGGTCAGTATAGTTCT
CCATAGAGAATTTTACTTTATGTAAGGCAGATTAAGAGTAAATTTCACTTTTAACCTCAAGTTGCATTTTATTGACAACTTTTACCCTAT
TTAGTGAGAGTTCCAATTTTTTTACCCCATTTCAGAAAAGTTCGTCCACAAATTACCCATTTAAGATGTTTGGTGAATTCCCTTCGTTCT
TACTTGCGGGGCTCCCTTGCAGGGCTGACGTGGCGTGGCCAACGCGGATACGTATTTGGCTCCCACGCGCGTCCTTGTTGCCGGTTC
GCAGACCGGCGACCGAACCAGTCAAGCTAGCAAGGGCTTGGGCGATGGCGCGACCATGGACAAAGCAGAGGTGGCCGGCGCGGA
GCAGCAGGCCGACTGGAGGAGGTCGGGCTAGCGAGAGAGAGAGATGGCAAACAACAAGGGACGCGCATGGCGGGACCCAATACGTG
TCCGAGCTGGCATGCCAGGTCGGCCCTACAACTGGGGCCCAACGGTCAGAACGATGGAGATTTACTGAAAAATCTAAAATGGGGTA
ATACGTGGAACAACTTTTCTTAAATGGGGTAAAAAAAAATGAAATTCCCAGTAAATGTGGTAAAAGTTGTCAAAAAAAAATTGATGGTA
AAAACTAGAATCTACTCACAGATTATTTAGCATTGTACCATTGCTTCCCTGGTCAGATTTCCTTTGCTACCTAATCTTGTGATCTACATG
TGAAATTTAAGCAGAACCTACATAAATGACCAAATGCGGTCCAAGTACCGAGCTAGCAAGTCTCATGGGCCAATCCACTACAGGCCC
ACATATATTTTCTAGTTTCTCTTCTGAAAATAGAAAAGAACATTTTACAATTATAGTTTAATGAAGCCTTCCAGAATAAGTAGTCCATTG
CTGGGATGGATTACCAAATCACAATTTCACAACTCACAAGGCATTGCCACTTTACTATTTGTGCTGGTCACTGTATTGAGAGTTTCGGA
TGGTGAAAGAGGAGAAAATTGAAGGTGGAGCGTTGTTTTGGTGCTTTATATCTTAGCTAACTATAAGAATCCACCGCTGACTCTTACG
TTGCTGGATATTATAATGCAAACTGCTAGAAGACCTAGCAATGAGTTGTTGGACTGATATGTTTTTTGTCACATGCACAATTAGACTTG
TAGGAATGGTGCTATTTTCTTGTCCATCCGAAAGTAAGCCAAGATTTTTTTTGAAAGTGTAAGCCAAGATTTTTGTGAAAGTGAGCT

AAGATATTTGAGTGAATCTGGAGAGCTCAGTTGTGAAGCATGTATGATATGATCGGTCACAGTTAAGCTGCCATTTTCAGTAAATTTT
GGAGCTCACGGCTGCTTGTGATAAATAAGTTGATGGTCATTTTCTAATTTCTTGTAATAATAAGTTGATGGTTTATTTATGTTATGCTC
ACTCCTGGCCGGCCTGCTTTCTGCTGCATTTCAGTATTCTTTTTGTGGATAATATTTTTCTTGTAATAAAAACTGTACTACAACTTAGGA
AGAACAGAGGGAGTAATTGTTCACATCGGAGGTTCGCTTGCAAGTTGCATGCGGCTCCCTGATTTTGACCAGTGCATGGTCCTAGCA
GCCATAGCCCACCAAACGCCTAGAATTGACCAACAAGCTACTAAGCTTTGTTGACCTTATTGTTCAGATTAGCAGCCATTACAGTTAG
GTCTCTGTTTCTTCACTGCTCAAATAGGATAATGAAAGGATTACTAGTATGGATAGGACGTCGACAGCTAAAAAGAAAGATATGTTAA
GGGCGTAGCCGTGCTACAATTGGCAAGTTGCATACATGCAGATATGTCCGCAAGACAAGATTTGATGCGAGACGGCGATTGATCTGC
GATGGCTTTGCAAGTTGCAGATGCAGAATTTGGAGCAGCCTAGCCTAGGCAGTCCAGTTGACCAATAAAAAATCCCCTTTGTTGGCC
ACATGATCCAACTCTGCTCAGCTTACTTAAAACGGAGAACTGGACGTGGAGCAATCTCAATCTCACATCATTTCATATAATACTACATA
TAGATTCCATTCATGTGATGTATCAATGGAGACCTTCATTTCACTTGCAAGTACGCCTGACCTCCATTAGACCAAAACTAACTCAGAAA
GCTTGGAGCATAGAGCCAGGCGCAAGAAAGAATCA

```
Alignment of Sequence_1:  [Untitled Sequence #1] with  Sequence_2: [Sequence Window #2]

Similarity : 5475/6222 (87.99 %)

Seq_1  1      ------------------------------------------------------------  0

Seq_2  1      GAATGTATCTAGACATGACTTAGTGTATAGATGCATTCAAATTTTGTCAAAGTTGAGACA  60


Seq_1  1      ------------------------------------------------------------  0

Seq_2  61     TCTTTTGTTGGACGGAGGGGGTATAATGTGTTTGGTATCGTTCATTCGTTCTAATAAAAA  120


Seq_1  1      ------------------------------------------------------------  0

Seq_2  121    TATGGCGGTGCGCATAAGACCTGGAGGTTTTGCCTTCATTCTTTGAAAAAAATAAATTTG  180


Seq_1  1      ------------------------------------------------------------  0

Seq_2  181    CAGAGTGTCATCATCAATGTTTGATTTGGGCATGTTCACATTTTTATCCATTGCATTTTG  240


Seq_1  1      ------------------------------------------------------------  0

Seq_2  241    TTTTCTGAACTCGCAAGTTATTGTACTCCCTCCGTCCAACAAAAGATGTCTCAAGTTTGT  300


Seq_1  1      ------------------------------------------------------------  0

Seq_2  301    CCAAATTTGGATGTATCTAGACATGACTTAGTGTATAGATGCATTCAAAATTGGTCAAAG  360


Seq_1  1      ------------------------------------------------------------  0

Seq_2  361    TTGAGACATCTTTGTTGGACGGAGGGAGTACTCAATATTCATTGATTAATTGTGACAACT  420


Seq_1  1      ------------------------------------------------------------  0

Seq_2  421    CAACAAAAAAATTCTGATAGCAATCTGCTCATAAAACCACCCATGGATACCTGACCTGAA  480


Seq_1  1      -----------------------GTCAACGGGTGTCACCCATGGGGTAAGCCAATTAGTA  37
                                     |||||||||||||||||||||||||||||||||||||
Seq_2  481    TGGGGGTATGGATCGTCGTTTTCGTCAACGGGTGTCACCCATGGGGTAAGCCAATTAGTA  540


Seq_1  38     ATGGGCAGGGCATGAGTACAACTTGGCGCCCATGGGTTGCCTAATTATTTTTTTTAATAA  97
              ||||| |||| ||||||||||||||||||||||||||||||||||||||||||| |||||
Seq_2  541    ATGGGTAGGGTATGAGTACAACTTGGCGCCCATGGGTTGCCTAATTATTTTTTT-AATAA  599
```

```
Seq_1  98    TGTTTATAAGCCAAACTACAATTATGTAATTGCACATGTAATTGCACTTTTTCATGTGAA  157
             ||||||||||||||||||||||||||||||||||||||||||||||||||||||||||||
Seq_2  600   TGTTTATAAGCCAAACTACAATTATGTAATTGCACATGTAATTGCACTTTTTCATGTGAA  659


Seq_1  158   TTATGATCTTATATTGATGCATGCATAATACATATATTATCATTTCTTTCATGTTTATGA  217
             |||||||||| |||||||||||||||||||||||||||||||||||||||||||||||||
Seq_2  660   TTATGATCTTCTATTGATGCATGCATAATACATATATTATCATTTCTTTCATGTTTATGA  719


Seq_1  218   TGTTCTTATATTAATGCACAACTAG---AGTGACCCACACGTTTGCACGACTAGAGTTTC  274
             |||||||||||||||||||||||||   ||||||||||||||||||||||||||||||||
Seq_2  720   TGTTCTTATATTAATGCACAACTAGCAGAGTGACCCACACGTTTGCACGACTAGAGTTTC  779


Seq_1  275   TACAGATTATCACCATCTTTGAGCCGGGGGTTCATCACCTTTCGTTTTGGGAAACTAAGC  334
             ||||||||||||||||||||||||||||||||||||||||||||||||||||||||||||
Seq_2  780   TACAGATTATCACCATCTTTGAGCCGGGGGTTCATCACCTTTCGTTTTGGGAAACTAAGC  839


Seq_1  335   TCCCTGATATGGATGGCAATGGGTACTCACTACCCGTGCACTTACCCTCTAGGTATTTTT  394
             || |||||||||||||||||||||||||||||||||||||| ||||||||||||||||||
Seq_2  840   TCTCTGATATGGATGGCAATGGGTACTCACTACCCGTGCACGTACCCTCTAGGTATTTTT  899


Seq_1  395   GTGTTCTTGTTACATGCTCATGCCGGCAAGGTTTTACATGGTCTTAGCACATGCTTTACA  454
             ||||||||||||||||||||||||||||||||||||||||||||| ||||||||||||||
Seq_2  900   GTGTTCTTGTTACATGCTCATGCCGGCAAGGTTTTACATGGTCTTGGCACATGCTTTACA  959


Seq_1  455   AAATGGACTATCTCTGATACATTTTCATAACTTAGGGTTTTAACTTTGTGATTTGAAGCA  514
             ||||||||||||||||||||||||||||||||||||||||||||||||||||||||||||
Seq_2  960   AAATGGACTATCTCTGATACATTTTCATAACTTAGGGTTTTAACTTTGTGATTTGAAGCA  1019


Seq_1  515   TCGGACCAACTTTTGTAATTTTTTTAGTTGATATTTTTATTGGGCCTTCATTTAGCATTA  574
             |||||||||||||||||| |||||||||||||||||||||||||||||||||||||||||
Seq_2  1020  TCGGACCAACTTTTGTAAATTTTTTAGTTGATATTTTTATTGGGCCTTCATTTAGCATTA  1079


Seq_1  575   ATAACGAGTAATGTGAATGAAACAACAGCAAAATTGAAATAATTCTTAAAGTTGTCAAAA  634
             |||||||||||||||||||||||||||| |||||||||||||| ||||||||||||||||
Seq_2  1080  ATAACGAGTAATGTGAATGAAACAACAGGAAAATTGAAATAATACTTAAAGTTGTCAAAA  1139


Seq_1  635   CTTATTTATTTATTGGCATGAAGCCATACTTTTGTATACAAGATTGAGATAAATAAGTCA  694
             ||||||||||||||||||||||||||||||||||||||||||||||||||||||||||||
Seq_2  1140  CTTATTTATTTATTGGCATGAAGCCATACTTTTGTATACAAGATTGAGATAAATAAGTCA  1199


Seq_1  695   AGTCAATACACTAAATTTTAAATCGGTACACACTTCACAAATACACTTTGGGTATATAAG  754
             ||||||||||||||||||||||||||||||||||||||||||||||||||||||||||||
Seq_2  1200  AGTCAATACACTAAATTTTAAATCGGTACACACTTCACAAATACACTTTGGGTATATAAG  1259


Seq_1  755   AATGGCCCGGCAAAGGTTTCTTCCTTGCAGATCTCCACCAATCAGCAATACAAATAAGCA  814
             ||||||||||||||||||||||||||||||||||||||||||||||||||||||||||||
Seq_2  1260  AATGGCCCGGCAAAGGTTTCTTCCTTGCAGATCTCCACCAATCAGCAATACAAATAAGCA  1319


Seq_1  815   GCTACGGGTGGTCTGAACTTAATTGGGTAAACTCGCCTTGTCTCTAAAGTCCGACGAAGA  874
             |||||||||||||||||||||||||||||||||||||||||||| |||||||||||||||
Seq_2  1320  GCTACGGGTGGTCTGAACTTAATTGGGTAAACTCGCCTTGTCTCCAAAGTCCGACGAAGA  1379


Seq_1  875   TCTGGCCCACTCTCGCGCCCCTCGTTCTTTCCGGATGTCTGCTCCCTCACACGCGACAAC  934
             |||||||||||||||||||||||  ||||||||||||||||| |||||||||||||||||
Seq_2  1380  TCTGGCCCACTCTCGCGCCCCTCACTCTTTCCGGATGTCTGCGCCCTCACACGCGACAAC  1439
```

```
Seq_1  935   TGTCGGTTCAGAAATCTTGGTCATAATAACCGTTGATAACTTAACTGAGTGTGTATTTTT  994
             ||| ||||||||||||||||||||||||||||||||||||||||||||||||||||||||
Seq_2  1440  TGTTGGTTCAGAAATCTTGGTCATAATAACCGTTGATAACTTAACTGAGTGTGTATTTTT  1499

Seq_1  995   TCCAAAATCATTAA--------ATCATTATTATCATTAGAATAAATAAATAATTGGTCGG  1046
             ||||||||||||||        || ||||||||||||| |||||||||||||||||||||
Seq_2  1500  TCCAAAATCATTAACTCATTAAATTATTATTATCATTAAAATAAATAAATAATTGGTCGG  1559

Seq_1  1047  TGGTGCGTACGTGGACAAACAAATTGCGTCCGAAAAGAACACGTCTCGTCGTCCGACATC  1106
             ||||||||||||||||||||||||||||||||||||||||||||||||||||||||||||
Seq_2  1560  TGGTGCGTACGTGGACAAACAAATTGCGTCCGAAAAGAACACGTCTCGTCGTCCGACATC  1619

Seq_1  1107  GATCAGAGTCCAAACCGAGACTGACTAGGGTTTGGTTGATCGACTATATATATCTGAACC  1166
             ||||||||||||||||||||||||||||||||||||||||||||||||||||||||||||
Seq_2  1620  GATCAGAGTCCAAACCGAGACTGACTAGGGTTTGGTTGATCGACTATATATATCTGAACC  1679

Seq_1  1167  CAGAGCTAGCCGCCGCCTCCAGTCCAGATCGATCTATAGAAAGAGAAGGATATATCGAGA  1226
             |||||||||||||||||||||||||||||||||||| |||||||||||||||||||||||
Seq_2  1680  CAGAGCTAGCCGCCGCCTCCAGTCCAGATCGATCTAGAGAAAGAGAAGGATATATCGAGA  1739

Seq_1  1227  TCCGGCGAGATGAAGCATGATAGGGCTGAGGAGGTTGCGTCCGACGGCGACGGAGAGATC  1286
              |||||| ||||||||||||||||||||||||||||||||||||||||||||||||||||
Seq_2  1740  ACCGGCGCGATGAAGCATGATAGGGCTGAGGAGGTTGCGTCCGACGGCGACGGAGAGATC  1799

Seq_1  1287  AAGTCGCAGAATCCGGCGGAGGCAGCAGAAGCAGCGCCGCAAACAGAGGGCCTGTCGTCC  1346
             ||||||||||||||||||||||||||||||||||||||||||||||||||||||||||||
Seq_2  1800  AAGTCGCAGAATCCGGCGGAGGCAGCAGAAGCAGCGCCGCAAACAGAGGGCCTGTCGTCC  1859

Seq_1  1347  AAGAGAGCATCATCCGCCGGACCGAGCGGCGACGGCGACGCCATGATCAAGTCGCAGAAG  1406
             ||||||||||||||||||||||||||||||||||||||||||||||||||||||||||||
Seq_2  1860  AAGAGAGCATCATCCGCCGGACCGAGCGGCGACGGCGACGCCATGATCAAGTCGCAGAAG  1919

Seq_1  1407  GTGGCGGAGGGCGACAAGGCGGAGGCGGGGAAGGCGAAGAGGATCGCCAAGGTGCCCCAG  1466
             |||||||||||||||||||||||| |||||||||||||||||||||||||||||||||||
Seq_2  1920  GTGGCGGAGGGCGACAAGGCGGAGTCGGGGAAGGCGAAGAGGATCGCCAAGGTGCCCCAG  1979

Seq_1  1467  AAGTACATCGATCTGCTGCGCAATGGCGGCTTCCCCAGGCTTCCCACCTTCGACGGCCCC  1526
             ||||||||||||||||||||||||||||||||||||||||||||||||||||||||||||
Seq_2  1980  AAGTACATCGATCTGCTGCGCAATGGCGGCTTCCCCAGGCTTCCCACCTTCGACGGCCCC  2039

Seq_1  1527  AGCAAATCCCGATCTCCTGCCGTCCAGGCACGCGTGGCTCACTGCAAAGCTCTGGTGGAC  1586
             ||||||||||||||||||||||||||||||||||||||||||||||||||||| ||||||
Seq_2  2040  AGCAAATCCCGATCTCCTGCCGTCCAGGCACGCGTGGCTCACTGCAAAGCTCTCGTGGAC  2099

Seq_1  1587  GAACTCCGAGCCTACAACGCCGGCATCCTGGCGCAGTACGACGAGCTCGGCCACGCCTAC  1646
             ||||||||||||||||||||||||||||||||||||||||||||||||||||||||||||
Seq_2  2100  GAACTCCGAGCCTACAACGCCGGCATCCTGGCGCAGTACGACGAGCTCGGCCACGCCTAC  2159

Seq_1  1647  CATGAGGTCGAGGAGGAGCCGTGGATCGACGAAGCCATGGCCCGCCAGCTGGCCAGGAAG  1706
             ||||||||||||||||||||||||||||||||||||||||||||||||||||||||||||
Seq_2  2160  CATGAGGTCGAGGAGGAGCCGTGGATCGACGAAGCCATGGCCCGCCAGCTGGCCAGGAAG  2219

Seq_1  1707  AAGCCGATCCCGCCGCCGGCTTCATCTTGATCGAAGCAGGAATTTCATCAAGAACATGCT  1766
              |||||||||||||||||||||||||||||||||||||||||||||||||||||||||||
Seq_2  2220  GAGCCGATCCCGCCGCCGGCTTCATCTTGATCGAAGCAGGAATTTCATCAAGAACATGCT  2279
```

```
Seq_1  1767  TATCCATCCCCTGCCAAAACTATAGAGTTTATACGCGCGAGAGTCGACTTTGTATAATTA  1826
              |||||||||||||||||||||||||||||||||||||||||||||||||||||||||||
Seq_2  2280  GATCCATCCCCTGCCAAAACTATAGAGTTTATACGCGCGAGAGTCGACTTTGTATAATTA  2339

Seq_1  1827  GAATTTAAGTTAATGTCCGTGATGTCCATCTGGGACAAGTTAATGCTCTGGTAATCGATA  1886
             |||||||||||||||||||||||||||||||||||||||||||||||||| |||||||||
Seq_2  2340  GAATTTAAGTTAATGTCCGTGATGTCCATCTGGGACAAGTTAATGCTCTGATAATCGATA  2399

Seq_1  1887  GTATAATCTGTCTCACAGTCCGGTTCAACTTGCCCTGCAGATGGTCATAAAAGACCTCAT  1946
             ||||||||||||| ||||||||||||||||||||||||||||||||||||||||||||||
Seq_2  2400  GTATAATCTGTCTTACAGTCCGGTTCAACTTGCCCTGCAGATGGTCATAAAAGACCTCAT  2459

Seq_1  1947  AATCTGTTAGTTAATGATCGTGACTTCATGAATAACTCAAGCTATTAGTACAGTTTCTTT  2006
             ||||||||||||||||||||||||||||||||||||||||||||||||||||||||||||
Seq_2  2460  AATCTGTTAGTTAATGATCGTGACTTCATGAATAACTCAAGCTATTAGTACAGTTTCTTT  2519

Seq_1  2007  TCCTTGCGTCAGTGACTTAATCTTAGATGAATTTAGCTGTGTTCATGTCGTGGTGGTATC  2066
             |||| |||||| ||||||||||||| ||||||||||||||||||||||||||||||||||
Seq_2  2520  TCCTCGCGTCAATGACTTAATCTTACATGAATTTAGCTGTGTTCATGTCGTGGTGGTATC  2579

Seq_1  2067  ACGG----------------------------------------------AACCGGAGGA  2080
             ||||                                              ||||||||||
Seq_2  2580  ACGGCAGATGCCATAAGATGGTTTAACTTGGAGCCGATGGACGAAGGATTAACCGGAGGA  2639

Seq_1  2081  GAGAGGACGTAAAGGGAACCACGCACGATTCACACAAGAACACACAGATTTACCCAGGTT  2140
             | |||||||| ||||||| || |||||||||||||||||||||||||||||| |||||||
Seq_2  2640  GGGAGGACGTGAAGGGAAACATGCACGATTCACACAAGAACACACAGATTTATCCAGGTT  2699

Seq_1  2141  CAGAGCCCTCTTGTCGAGGTAAGACTCTTACTCCTGATTTGTTGTGTTAGCCGAGATAGG  2200
             |||| ||||||||||||||||||||||||||| |||||||||||||||||||||||||||
Seq_2  2700  CAGAACCCTCTTGTCGAGGTAAGACTCTTACTTCTGATTTGTTGTGTTAGCCGAGATAGG  2759

Seq_1  2201  CAAGGTCTACAATGGCGCTCCTTGAGCTGTATTCTTGAGGAAAAAGAAGAAAGGGGAAAC  2260
             |||||||||||||| |||| ||||| ||||||||||||||||||||||||||| ||||||
Seq_2  2760  CAAGGTCTACAATGACGCTTCTTGAACTGTATTCTTGAGGAAAAAGAAGAAAGAGGAAAC  2819

Seq_1  2261  CTTAGATGCCTAGAATGCTCCGTCCATCTCTACAGAGGGGTAAGATTCTATTTATAGGGG  2320
             | ||||||||||||||||||||||||||||       ||||||| |||||||||||||||
Seq_2  2820  CCTAGATGCCTAGAATGCTCCGTCCATCTC-CACAGAGGGTAAGGTTCTATTTATAGGGG  2878

Seq_1  2321  GTTACTTGGGCCTTGCCAGGCACGCGGCTTGATTCTCTCCAGTCAGTACCGCAGGGGACA  2380
             ||| ||||||||  ||||||||||||||||| |||||||||| |||||||||||||||||
Seq_2  2879  GTTCCTTGGGCCCCGCCAGGCACGCGGCTTGGTTCTCTCCAGGCAGTACCGCAGGGGACA  2938

Seq_1  2381  AGACAGCTTTATTTTTCCCTGCCAGCCTGCAACGGGTACAGCTATCCTCTGCCGGCTTCT  2440
                  |||||| |||||| || |||||||||||||||||| ||||||||||||||||||
Seq_2  2939  -----GCTTTACTTTTCCTTGTCAGCCTGCAACGGGTACATCTATCCTCTGCCGGCTTCC  2993

Seq_1  2441  GTCATAGATTCTCTTTCGGTGCTTCTCTAGCGTGGTCTCCTGACACCGGTACGCAGGCGC  2500
             ||||||||||||||||||||||||||||||||||||||||||||||||||||||||| ||
Seq_2  2994  GTCATAGATTCTCTTTCGGTGCTTCTCTAGCGTGGTCTCCTGACACCGGTACGCAGGTGC  3053

Seq_1  2501  TGACTGCTTTTCGGGAAATAATGATGGCGTTGCTTTACTTTGCACTGCGTGGTCAGGATA  2560
             |||||| ||||||||| |||||||||||||||||||||||||||||||||||||||||||
Seq_2  3054  TGACTGTTTTTCGGGAGATAATGATGGCGTTGCTTTACTTTGCACTGCGTGGTCAGGATA  3113
```

```
Seq_1  2561  AGACCGTT--GAGGGATCTCGGCTATCGCCGAGACCAATGTGCCCATCCTTATCCTGCAA  2618
             ||||||||  ||  |||||||||||||||| | |||||||||||||| ||||||||||||
Seq_2  3114  AGACCGTTTAGA--GATCTCGGCTATCGCCAAAACCAATGTGCCCATTCTTATCCTGCAA  3171

Seq_1  2619  ACTCATAAGACAAGATAGGCCTGCCGGCATACACCCGGGATGCCGGCTTAGCGTTAGTTG  2678
             ||||||||||||||||||||||||||||||||||||||||||||||||||||||||||||
Seq_2  3172  ACTCATAAGACAAGATAGGCCTGCCGGCATACACCCGGGATGCCGGCTTAGCGTTAGTTG  3231

Seq_1  2679  TACTTTACGATGCCGACATACGTAACTATGCCGGCTTTGCCTCCGTCATCTCGGGTAGAG  2738
             ||||||||||||||| |||||||||||||||||||||||||| |||||||| || |||||
Seq_2  3232  TACTTTACGATGCCGGCATACGTAACTATGCCGGCTTTGCCTTCGTCATCTTGGTTAGAG  3291

Seq_1  2739  CTGTGTCGCCATGACCCTATCCGAGGTCAT------CCCCCCCGACAGTTCATGTATATG  2792
             | ||||||||||||||||| ||| |||||       ||||||||||||||||||||||||
Seq_2  3292  CCGTGTCGCCATGACCCTACCCGGGGTCACCCCCCCCCCCCCGACAGTTCATGTATATG  3351

Seq_1  2793  CAATGTGATACCTCCGACACTTGCTACGGAAGATGCGTCAGAGGTGGCGCATCACCACTT  2852
             ||||||||||||||||||||||||||||||||||||||||||||||||||||||||||||
Seq_2  3352  CAATGTGATACCTCCGACACTTGCTACGGAAGATGCGTCAGAGGTGGCGCATCACCACTT  3411

Seq_1  2853  GACGCATAGTTTTTGACGGTTCCCTGTTTCGTCAGAAGAGAGGGTTTGAGGGTCGTCAAA  2912
             ||||||||||||||||||||||||| |||||||||||||||
Seq_2  3412  GACGCATAGTTTTTGACGGTTCCCTATTTCGTCAGAAGAGA-------------------  3452

Seq_1  2913  GATAAACTGTACTGTAGTAGTGAGTAAACATCACGGGATAGAGCTTACAAATCAACAAGA  2972

Seq_2  3453  ------------------------------------------------------------  3452

Seq_1  2973  AACTAAAAATCAACAAAGGTTTCTGAACTCTTGATCTGTTACAAGGAATCTCACAAACAG  3032

Seq_2  3453  ------------------------------------------------------------  3452

Seq_1  3033  CTAAAGTAATCAACAGTTGTATGTCTCAGATGTCTCAGATCACAGGAACATCAAGATAAG  3092

Seq_2  3453  ------------------------------------------------------------  3452

Seq_1  3093  CAGGAAGCTGAGAATTAAAGGAAGAGAGCGATTCGGTTTTTTTCTCAGTTAAGTCACTGT  3152

Seq_2  3453  ------------------------------------------------------------  3452

Seq_1  3153  GTGGGCTGTCTTTCTTCTTATGGCAATGAGTTGGTGACATTAATTTTTTTTGTCAAAGTC  3212

Seq_2  3453  ------------------------------------------------------------  3452

Seq_1  3213  TCAATGGTTGATGGACTTATGACACGTCCATCCTATCTAATTCCCTATCTACTATGCACT  3272

Seq_2  3453  ------------------------------------------------------------  3452

Seq_1  3273  TTAATCCTCTAGAGATGATGTATGACAGACTCTTTGATCCTCCGGTATCTTCAGTTCATT  3332

Seq_2  3453  ------------------------------------------------------------  3452

Seq_1  3333  TGGAGTTGGAAAGTATGTCGCCATGACCCTACCCGGGGTCATCCCCCGACAGTTCATCAT  3392

Seq_2  3453  ------------------------------------------------------------  3452
```

```
Seq_1  3393  GTATATGCAATGTGATACCTCCGACACTTGCTACGGAAGATGCGTATGAGGTGGCGCATC  3452

Seq_2  3453  ------------------------------------------------------------  3452


Seq_1  3453  ACCACTTGACGCATAGTTTTTGACGGTTCCCCGTTTCGTCAGAAGAGATGCGTCAGAACA  3512
                                                             ||||||||||||
Seq_2  3453  ------------------------------------------------TGCGTCAGAACA  3464


Seq_1  3513  CCTTTATGATCACTCAGTTACGAGATGACGGTTGATATTCACAAAGTATTCTTCCGGTAC  3572
             ||||||||||||| ||||||||| ||||||||||||||  ||||| |||| |||||||||
Seq_2  3465  CCTTTATGATCACCCAGTTACGAAATGACGGTTGATATCTACAAAATATTTTTCCGGTAC  3524


Seq_1  3573  TAGAGAATGACATGATCTCATGGTCTAAGGAAATGATACTTGACATAATAAAAG-TTTTA  3631
             ||||||||| ||||||||||||||||||||||||||||||||||||||||||||   |||
Seq_2  3525  TAGAGAATGGCATGATCTCATGGTCTAAGGAAATGATACTTGACATAATAAAAGCA-TTA  3583


Seq_1  3632  GCAATTTAAACTTAAGTGACACGATCAAAAGTTATGTTTAGGTTTGGGTCTGTCCATCAC  3691
               ||||||||||||||||||||||||||||| |||| |||||||||||||||||||||||
Seq_2  3584  TTAATTTAAACTTAAGTGACACGATCAAAAGCTATGCTTAGGTTTGGGTCTGTCCATCAC  3643


Seq_1  3692  ATCATTCTCCTGATGATATGACCTCGTTATTAAATGACAACACATGTCTATGGTTAGAAA  3751
             ||||||||||| ||||||||||||||||||||||||||||||||||| ||||||||| ||
Seq_2  3644  ATCATTCTCCTAATGATATGACCTCGTTATTAAATGACAACACATGTTTATGGTTAGGAA  3703


Seq_1  3752  ACCTTAACCATCTTTTAACCAACGAGCTAATCTAGTAGAGGCGTATTAGG-ACACGGTAT  3810
             | ||||||||  |||||||||||||||||||||||||||| ||||||||| |||||||||
Seq_2  3704  ATCTTAACCAATTTTTAACCAACGAGCTAATCTAGTAGAGACGTATTAGGAACACGGTAT  3763


Seq_1  3811  TTATTTATTTA-CCATACATGTATTTAGTTTCATGTTAATCCATGCAAAGCACCTTAATC  3869
             ||||||||||| |||||||||| |||| |||| |||||||||||||||||||||||||||
Seq_2  3764  TTATTTATTTATCCATACATGTCTTTACTTTCCTGTTAATCCATGCAAAGCACCTTAATC  3823


Seq_1  3870  CGGCGTTGCAAGGCGAACGCGCGTCGAGGAAGATTCTGTGCGAGCACCTGCGCGGCGCGG  3929
             |||||||||||||||| ||||||||||||||||||||||||||||||||||||||||
Seq_2  3824  CGGCGTTGCAAGGCGAGCGCGCGTCGAGGAAGATTCTGTGCGAGCACCTGCGCGGCG---  3880


Seq_1  3930  CGGAGCCAGGGGAGGAATC--------------------------TTTCTTCTAGTGTTG  3963
                       |||||||||                          |||||||||||||||
Seq_2  3881  ----------GGAGGAATCTGTGGTACTTGGTTCCCTAGGACGTGTTTCTTCTAGTGTTG  3930


Seq_1  3964  CGGATTACTCCGCTTCCATTTTCAAGGACAGACCACGAGAAGGAGGCCATTGAAAAAAAG  4023
             ||||||||||||||||||||||||||||||||||||||||||||||||||||||||||||
Seq_2  3931  CGGATTACTCCGCTTCCATTTTCAAGGACAGACCACGAGAAGGAGGCCATTGAAAAAAAG  3990


Seq_1  4024  AGAGCAGCTATGAGAAACATGCTGCTACGAGTGTGTACAAGGGTCAGTATAGTTCTCCAT  4083
             ||||||||||||||||||||||||||||||||||||||||||||||||||||||||||||
Seq_2  3991  AGAGCAGCTATGAGAAACATGCTGCTACGAGTGTGTACAAGGGTCAGTATAGTTCTCCAT  4050


Seq_1  4084  AGAGAATTTTACTTTATGTAAGCCAGATTAAGAGTAAATTCCACTTTTAACCTCAAGTTG  4143
             |||||||||||||||||||||| ||||||||||||||||| |||||||||||||||||||
Seq_2  4051  AGAGAATTTTACTTTATGTAAGGCAGATTAAGAGTAAATTTCACTTTTAACCTCAAGTTG  4110


Seq_1  4144  CATTTTATTGACAACTTTTACCCCATTTAGTGAGAGTTCCAATTTTTTTACCCCATTTCA  4203
             ||||||||||||||||||||||| ||||||||||||||||||||||||||||||||||||
Seq_2  4111  CATTTTATTGACAACTTTTACCCTATTTAGTGAGAGTTCCAATTTTTTTACCCCATTTCA  4170
```

```
Seq_1  4204  GAAAAGTTCGTCCACAAATTACCCATTTAAGATGTTTGGTGAATTCCCTTCGTTCTTGCT  4263
             ||||||||||||||||||||||||||||||||||||||||||||||||||||||||| ||
Seq_2  4171  GAAAAGTTCGTCCACAAATTACCCATTTAAGATGTTTGGTGAATTCCCTTCGTTCTTACT  4230

Seq_1  4264  TGCGGGGCTCCCTTGC-GGGGTTGACGTGGCGTGGCCAACGCGGATACGTATTTGGCTCC  4322
             |||||||||||||||| || | ||||||||||||||||||||||||||||||||||||||
Seq_2  4231  TGCGGGGCTCCCTTGCAGG-GCTGACGTGGCGTGGCCAACGCGGATACGTATTTGGCTCC  4289

Seq_1  4323  CACGTGCGTCCTTGTTGCCGGTTCGCAGACCGGCGACCGAACCAGTCAAGCTAGCGAGGG  4382
             |||| ||||||||||||||||||||||||||||||||||||||||||||||||||| ||||
Seq_2  4290  CACGCGCGTCCTTGTTGCCGGTTCGCAGACCGGCGACCGAACCAGTCAAGCTAGCAAGGG  4349

Seq_1  4383  CTTGGGCGATGGCACGACCATGGACAAAGCAGAGGTGGCCGGCGCGGAGCAGCAGGCCGA  4442
             ||||||||||||| ||||||||||||||||||||||||||||||||||||||||||||||
Seq_2  4350  CTTGGGCGATGGCGCGACCATGGACAAAGCAGAGGTGGCCGGCGCGGAGCAGCAGGCCGA  4409

Seq_1  4443  CTGGAGGAGGTCGGGCTAGCGAGAGAGAGATGGCAAACAACAAGGGACGCGCATGGCGGG  4502
             ||||||||||||||||||||||||||||||||||||||||||||||||||||||||||||
Seq_2  4410  CTGGAGGAGGTCGGGCTAGCGAGAGAGAGATGGCAAACAACAAGGGACGCGCATGGCGGG  4469

Seq_1  4503  ACCCAATACGTGTCCGAGCTGGCATGCCAGGTCGGCCCTACAACTGGGGCCCAACGGTCA  4562
             ||||||||||||||||||||||||||||||||||||||||||||||||||||||||||||
Seq_2  4470  ACCCAATACGTGTCCGAGCTGGCATGCCAGGTCGGCCCTACAACTGGGGCCCAACGGTCA  4529

Seq_1  4563  GAACGATGGAGATTTACTGAAAAATCTAAAATGGGGTAATATGTGGAACAACTTTTCTTA  4622
             ||||||||||||||||||||||||||||||||||||||||| ||||||||||||||||||
Seq_2  4530  GAACGATGGAGATTTACTGAAAAATCTAAAATGGGGTAATACGTGGAACAACTTTTCTTA  4589

Seq_1  4623  AATGGGGTAAAAAAA-TGAAATTCCCAATAAATGTGGTAAAAGTTGTCAAAAAAAAATTGA  4681
             ||||||||||||||| ||||||||||| ||||||||||||||||||||||||||||||||
Seq_2  4590  AATGGGGTAAAAAAAATGAAATTCCCAGTAAATGTGGTAAAAGTTGTCAAAAAAAAATTGA  4649

Seq_1  4682  TGGTAAAAACTAGAATCTACTCACAGATTATTTAGCATTGTACCATTGCTTCCCTGGTCA  4741
             ||||||||||||||||||||||||||||||||||||||||||||||||||||||||||||
Seq_2  4650  TGGTAAAAACTAGAATCTACTCACAGATTATTTAGCATTGTACCATTGCTTCCCTGGTCA  4709

Seq_1  4742  GATTTCCTTTGCTACCTAATCTTGTGATCTACATGTGAAATTTAAGCAGAACCTACATAA  4801
             ||||||||||||||||||||||||||||||||||||||||||||||||||||||||||||
Seq_2  4710  GATTTCCTTTGCTACCTAATCTTGTGATCTACATGTGAAATTTAAGCAGAACCTACATAA  4769

Seq_1  4802  ATGACCAAATGCGGTCCAAGTACCGAGCTAGCAAGTCTCATGGGCCAATCCACTACAGGC  4861
             ||||||||||||||||||||||||||||||||||||||||||||||||||||||||||||
Seq_2  4770  ATGACCAAATGCGGTCCAAGTACCGAGCTAGCAAGTCTCATGGGCCAATCCACTACAGGC  4829

Seq_1  4862  CCACATATATTTTCTAGTTTCTCTTCTGAAAATAGAAAAGAACATTTTACAATTATAATT  4921
             ||||||||||||||||||||||||||||||||||||||||||||||||||||||||| ||
Seq_2  4830  CCACATATATTTTCTAGTTTCTCTTCTGAAAATAGAAAAGAACATTTTACAATTATAGTT  4889

Seq_1  4922  TAATGAAGCCTTCCAGAATAAGTAGTCCATTGCTGGGATGGATTACCAAATCACAATTTC  4981
             ||||||||||||||||||||||||||||||||||||||||||||||||||||||||||||
Seq_2  4890  TAATGAAGCCTTCCAGAATAAGTAGTCCATTGCTGGGATGGATTACCAAATCACAATTTC  4949

Seq_1  4982  ACAACTCACAAGGCATTGCCACTTTACTATTTGTGCTGGTCACTGTATTGAGAGTTTCGG  5041
             ||||||||||||||||||||||||||||||||||||||||||||||||||||||||||||
Seq_2  4950  ACAACTCACAAGGCATTGCCACTTTACTATTTGTGCTGGTCACTGTATTGAGAGTTTCGG  5009
```

```
Seq_1  5042  ATGGTGAAAGAGGAGAAAATTGAAGGTGGAGCGTTGTTTTGGTGCTTTATATCTTAGCTC  5101
             ||||||||||||||||||||||||||||||||||||||||||||||||||||||||||| 
Seq_2  5010  ATGGTGAAAGAGGAGAAAATTGAAGGTGGAGCGTTGTTTTGGTGCTTTATATCTTAGCTA  5069

Seq_1  5102  ACTATAAGAATCCACCGCTGACTCTTACGTTGCTGGATATTATAATGCAAACTGCTAGAA  5161
             ||||||||||||||||||||||||||||||||||||||||||||||||||||||||||||
Seq_2  5070  ACTATAAGAATCCACCGCTGACTCTTACGTTGCTGGATATTATAATGCAAACTGCTAGAA  5129

Seq_1  5162  GACCTAGCAATAAGTTGTTGGACTGATATGTTTTTTGTCACATGCACAATTAGACTTGTA  5221
             ||||||||||| ||||||||||||||||||||||||||||||||||||||||||||||||
Seq_2  5130  GACCTAGCAATGAGTTGTTGGACTGATATGTTTTTTGTCACATGCACAATTAGACTTGTA  5189

Seq_1  5222  GGAATGGTACTATTTTCTTGTCCATCCGAAAGTAAGCCAAGATTTTTTTTGAAAGTGTA  5281
             |||||||| ||||||||||||||||||||||||||||||||||||||||||||||||||
Seq_2  5190  GGAATGGTGCTATTTTCTTGTCCATCCGAAAGTAAGCCAAGATTTTTTTTGAAAGTGTA  5249

Seq_1  5282  AGCCAAGATTTTTGTGAAAGTGAGCTAAGATATTTGAGTGAATCTGGAGAGCTCAGTTGT  5341
             ||||||||||||||||||||||||||||||||||||||||||||||||||||||||||||
Seq_2  5250  AGCCAAGATTTTTGTGAAAGTGAGCTAAGATATTTGAGTGAATCTGGAGAGCTCAGTTGT  5309

Seq_1  5342  GAAGCATGTATGATATGATTGGTCACAGTTAAGCTGCCATTTTCAGTAAATTTTGGAGCT  5401
             ||||||||||||||||||| ||||||||||||||||||||||||||||||||||||||||
Seq_2  5310  GAAGCATGTATGATATGATCGGTCACAGTTAAGCTGCCATTTTCAGTAAATTTTGGAGCT  5369

Seq_1  5402  CACGGCTGCTTGTGATAAATAATTTGATGGTCATTTTCTAATTTCTTGTAATAATAAGTT  5461
             |||||||||||||||||||||| |||||||||||||||||||||||||||||||||||||
Seq_2  5370  CACGGCTGCTTGTGATAAATAAGTTGATGGTCATTTTCTAATTTCTTGTAATAATAAGTT  5429

Seq_1  5462  GATG-TTTATTTATGTTATGCTCACTCCTGGCCGGCCTGCTTTTTGCTGCATTTCAGTAT  5520
             |||| |||||||||||||||||||||||||||||||||||||| ||||||||||||||||
Seq_2  5430  GATGGTTTATTTATGTTATGCTCACTCCTGGCCGGCCTGCTTTCTGCTGCATTTCAGTAT  5489

Seq_1  5521  TCTTTTTGTGGATAATATTTTTCTTGTAATAAAAACTGTACTACAACTTAGGAAGAACAG  5580
             ||||||||||||||||||||||||||||||||||||||||||||||||||||||||||||
Seq_2  5490  TCTTTTTGTGGATAATATTTTTCTTGTAATAAAAACTGTACTACAACTTAGGAAGAACAG  5549

Seq_1  5581  AGGGAGTAATTGTTCACATCGGAGGTTCGCTTGCAAGTTGCATGCGGCTCCCTGATTTTG  5640
             ||||||||||||||||||||||||||||||||||||||||||||||||||||||||||||
Seq_2  5550  AGGGAGTAATTGTTCACATCGGAGGTTCGCTTGCAAGTTGCATGCGGCTCCCTGATTTTG  5609

Seq_1  5641  ACCAGTGCATGGTCCTAGCAGCCATAGCCCACCAAACGCCTAGAATTGACCAACAAGCTA  5700
             ||||||||||||||||||||||||||||||||||||||||||||||||||||||||||||
Seq_2  5610  ACCAGTGCATGGTCCTAGCAGCCATAGCCCACCAAACGCCTAGAATTGACCAACAAGCTA  5669

Seq_1  5701  CTAAGCTTTGTTGACCATATTGTTCAGATTAGCAGCCATTACAGTTAGGTCTCTGTTTCT  5760
             |||||||||||||||| |||||||||||||||||||||||||||||||||||||||||||
Seq_2  5670  CTAAGCTTTGTTGACCTTATTGTTCAGATTAGCAGCCATTACAGTTAGGTCTCTGTTTCT  5729

Seq_1  5761  TCACTACTCAAATAGGATAATGAAGGGATTACTAGTATGGATAGGACGTCGACAGCTAAA  5820
             ||||| |||||||||||||||||| |||||||||||||||||||||||||||||||||||
Seq_2  5730  TCACTGCTCAAATAGGATAATGAAAGGATTACTAGTATGGATAGGACGTCGACAGCTAAA  5789

Seq_1  5821  AAGAAAGATATGTTAAGGGCGTAGCCGTGCTACAATTGGCAAGTTGCATACATGCAGATA  5880
             ||||||||||||||||||||||||||||||||||||||||||||||||||||||||||||
Seq_2  5790  AAGAAAGATATGTTAAGGGCGTAGCCGTGCTACAATTGGCAAGTTGCATACATGCAGATA  5849
```

```
Seq_1  5881  TGTCCGCAAGACAGGATTTGATGCGAGACGGCGATTGATCTGCGATGGCTTTGCAAGTTG  5940
             ||||||||||||| ||||||||||||||||||||||||||||||||||||||||||||||
Seq_2  5850  TGTCCGCAAGACAAGATTTGATGCGAGACGGCGATTGATCTGCGATGGCTTTGCAAGTTG  5909

Seq_1  5941  CAGATGCAGAATTTGGAGCAGCCTAGCCTAGGCAGTCCAGTTGACCAATAAAAA-TCCCC  5999
             |||||||||||||||||||||||||||||||||||||||||||||||||||||| |||||
Seq_2  5910  CAGATGCAGAATTTGGAGCAGCCTAGCCTAGGCAGTCCAGTTGACCAATAAAAAATCCCC  5969

Seq_1  6000  TTTGTTGGCCACATGATCCAACTCTGCTCAGCTTACTTAAAACGGAGAACTGGACGTGGA  6059
             ||||||||||||||||||||||||||||||||||||||||||||||||||||||||||||
Seq_2  5970  TTTGTTGGCCACATGATCCAACTCTGCTCAGCTTACTTAAAACGGAGAACTGGACGTGGA  6029

Seq_1  6060  GCAATCTCAATCTCACATCATTTCATATAATACTACATATAGATTCCATTCATGTGATGT  6119
             ||||||||||||||||||||||||||||||||||||||||||||||||||||||||||||
Seq_2  6030  GCAATCTCAATCTCACATCATTTCATATAATACTACATATAGATTCCATTCATGTGATGT  6089

Seq_1  6120  ATCAATGGAGACCTTCATTTCACTTGCAAGTACGCCTGACCTCCATTAGACCAAAACTAA  6179
             ||||||||||||||||||||||||||||||||||||||||||||||||||||||||||||
Seq_2  6090  ATCAATGGAGACCTTCATTTCACTTGCAAGTACGCCTGACCTCCATTAGACCAAAACTAA  6149

Seq_1  6180  CTCAGAAAGCTTGGAGCATAGAGCCAGGCGCAAGAAAGAATCA  6222
             |||||||||||||||||||||||||||||||||||||||||||
Seq_2  6150  CTCAGAAAGCTTGGAGCATAGAGCCAGGCGCAAGAAAGAATCA  6192
```

BdindelWSU_17, DOWNSTREAM

>Bradi5g01050

TGATTAATCTGTATCCTTTGCTACTATGTTCACCGACTTCCCAATAACATGTTATTCTATTATACTTGATTGATAACTGAACATGCATTTG
CAATATATATTCTGTGGAAATGTACTCCTGATCACGGTGAGTCTGATTATTGGATGATATACTAGTCAGACTTCACATGTAATATATCT
AGCAGTACAATTGTACCACCAAGAATGAAGTTTGAGAAAAATAATACAAATTGAAGTGGCAAGGACGATATCCTAGCTAGATTTACA
GCTCATCCCTCTCCTCTCCTTCTGTGTGCTTCTTACCCCTCTTGATCCCCCAAGAAGGATGGATTACAAATTATAATGCACCTGTTCCTT
GTCTTGATCACCCGAGATAGTGAAATGAAACTAGTTAGTTGTAGTTCCCATGATCATGCACTGTGTGGACTAATTAAACAGACGTGG
GAGGAAATTAATATGACAAACTATGTATATGGACTAATGGACAGAGGAGCCATGATATAGACTCTGACATGACGAGCAGTTGTGTAC
TTGTGTTAATTTACATCCAGATTTAGACTGATGGATGGCATGCTGGTAAGTGTTGGGACATCATATCGCTCGCTCTGGCAGTGTCCTC
ATCTGCTAACTGGCGCCCTTGCTGACTGCAATTCCCCTCTTGTTAGCTCGGCATCGCCGAACCATCCTCCATATCTATACTACCGTGTTG
TGCAATTATCAGCATTCATAAATGATCAAAGTTAGCTTGTTCAGGTTGTCCGAGTGCCATGTCGCTCCGAGCAGGTATTCCCATGTGTC
TTTGAACCTTGCCATCGCCTTTAGCACGATGGTTGTGGTTGCGAAGCGTCTCCTGTTGAACTCTACTTTGGAGTCTGAGAGGTTCATCT
CCTCCTTCTCCGACAGGTAAGTTGTGAGAAGAGAACTGGGAAACCAAGAGAGAAGAGGGTATCCTGCATTACTCATTAAGCAACACT
CATAGTAGTAGACTAGTAGTTGTCAAGTCTTTGTTGATATTCATGTGGACAGGGAAAGAACGAAATGGATGCAAAATCTATCCACAG
TTACGACGTGGACTGAACTGAACCAATTTTCTCATCTCCAAAAGTGCAATCAATATTTCTTCTGGCTGCCCATCAGAAGAAGGCGGAG
CAGAGCAAGTGAACAACCCTGGGCGGCGCCCCCGTCGTCAGGGAGGAGGAGGAAGGCGGCAAGCGATCGTTCAACGCGGTGCCAC
TGGCCGGCGTGCGCGTGGACGTCGCCGAGCGCGGCCGCGCCCTCTGCTCGATGCGCATCCCCGCCCAACCTCACGGTACGCACCACC
CTGATGCCTGATCCCCCTCCATCTCTGCATCTCGATCTGATTCCCCTGGTACGATCCGCTGGGCCGCTCCACATTCAGTGGAGGGAAA
AGGAGAAGAAACCAAAATGATCACTCATTTTAAGGGCCTATCCGGTATCACTGTGAATTCTAAAACTCCGCGGATCTCGAAATGCGG
ATCGGACCACCTGCTAGCCTATTTTTGCGGAGCGATGATTTGCGGCCTCTAGTTCTGGCTTCTGACCCCGCTGCATCGTTCCAAACCTC
ACCACGCAGCCGCCGCCTCCATCCTGCCCACCTTCAACCACAGCCGTCCCGTCTCTATCCTGTCCATTCCCGCTCCCCATTCGACCGAG
AGGAACCTAGCGAGGTAAAAAAAAGCCGTGAACCCCACCAGCACCGGCCGGGGAGGATGAGTGCCAGCGCGGGCTTCGTCGATGA
GGGGCCGGGAGGAGGTGGGCGAGCTCAGGGCACCCACGGCGGGGACAGCCCAACAATCCTCCCTCGCCGCCACGCTCACCAGGGG
GACCTGTCAACCAAGCAGGCCTCCTGGAACAGCGTGCCACGGCTTTGGAGAAGCGAGGAGGAGGTACAGGCCATGTGTGGGACGA
GAGTGGCAGGGAGAGAGGCATAATCGTGGTGCTGAGGAATGTCCGTTGTTACAAGCACACGCACGGCATCCTCCTCCCTGTGGATA
TCAGCAATGTTGCGTGCATAAATGAGCCCAGGAACTCAGGAATGTTCCCCGGTTCAACCTCCTTTTCAGGCCCAGAAGATTTCAAGCC
ATAGCGTGGTCGGAACAGTTTCTTGAGCGCATTGCAGACATTACCAGGAGAATCCACGAGGTGTTTTTAATCCCATCCAGATTGTTGC

ACGTTTGCTTGTACTCCCCTCCTCCCTTGCTTCTTCCCTCATCACTGCTGAGCAAAGCCCGTGCGGTGCGGTGAGGTGCCAGCACCCCT
TACTCCTGGGGGGGCTCTTCTAAATTAAAGCGCATATAAGAGAATCTGAGACCTCTCTCCTGAAAGTAGTTCACAAGAGGTAAAGCT
AATTCTAATGCAAAAGATAGCCAGGTAAATCGTTCACATTTGGAGAAAGCAAATCTGTACCATACATGTCTTTGTTTCCAGCGAAACC
ATGAGATTCAGGAACCAGCTTTGGCTAGTGCTGATGGGGATACAGTAATGTTATGTCCACGCTTTATCGATCTCCACGTGTACATGAT
TCATGAATCTTCTGGTGCTTTGAGGACAGACTGTTTGTGAAAGTAGTCCGCCATAAGCCACAAGACGGTTGTCAGCTCACCGCCACTG
CCGAGTCTCTTGGCATGGGACTCCCTGCTGCATCTATTGGCAGTGTAGACAAGAAAATCAGTCCACACATGTCGGATCAGTTCCAAGA
CAGCCATTCTGTCCTTCTCCTCTTCTTGTTTGAGTAGTGCTCGAGTAAGCCAGTTAGCATAACATAGGCGAGGAACATCTGTGCTGTAG
CCTGGCTTCTCTGTGTTGAGCATGATGGCAAGATCGTCTCTTTCCAATAGTCGAGAAGGATTAGGGTCGTCATGCAGCCAGAATAATT
CCTTCACCTTAGTCCAGCTGATACTGCGACTTGAGGTCTCTGCAGTACACATGTCAACCAGATTCTGGCAGGTCTGTTGGTACAGTCT
GGTCTGAGCAAGGCCTGGTAGCATTTCGGGCCGCTCAACAAGGAGGAACATCATGTAGTTGGACATTGTCCTAATGATCTCCACCAC
TTTCTCCTGGTCTGCATCATATGCCTTGTCTCGACTGCTTTTGGCAAGGAACACATCTGTGCCAATGTGCCAGATAATGATGCCCTCTT
GGAGCTCAATGCCAAGAGAGTCTTTGAAGTCTTTGAATTCGACACCATCATAACATTTATAATACTGCAATGCCTCTACGCCCCAATTT
GTCCTGATCATGCCTTGCGTGTTCACCTTGTTTTTCCTGTGCAATCGTTGTATGTACCAGTGCAGCCATAGCCTTAGCTCATCTGAAATC
TGAATGGTCCCTGAGTAATGCTTTCTGTTCCACCACTCATTTTGTCCCACCATCTTTGCCAATCTACCGAGAAGAGGACTGAAGGCTGT
GTCACGACGGCTGCAAAGTTGCAGCATGTTGTATTGCCCCATTTCACCGGACCACCTCCTTGCCTGACCACCAACTTGTATTCTCGTGA
TCTTCTTGACAAGCTGACGAAGCTGATCCCACCTTCCACTGCATAGAGCAGTTTTACGGAGCCATCTCCAACGTGTGGTGCACAAGAA
GGTGTATGTCCAACTTGACCCTAGCGCGCTCAACAAGGATGTTGCTTCCAGGAGGAAAGCCCCAGACAGCAAGGTGTATGTGATACC
AACATCAACTTTGCTGTCACCATCGTTGTCACTGAAGTAGAACAGCAGCAAGGAGGCGACAGTGGCAAGTGGCGAGACAATACGGA
TGCTGTAGCCAGGCCAAGTGTGGATCACAGCTGCCTTGGTATACAGGATGTCATACAACAGGGAGAGCTCCACCTCCATCAACATCC
ACATACCCTTGTATTTTTCATTTTCGATGTCTCTAAGCATCTCGCCACCACGGTTTTCAAAATCCTTTTCAATCCAAGAATCAACTATGG
CACGCTTGCATACGTGGAACATGGAGTGAGCAAGACGCACATAGTGTTCTTCCTTGTTGGCGGCCCCCTCTCTGAGCCCTTGATCCAG
AGTGCGGAACTGTTTCCGTTGGACATATGGCTCATTCTTGAGAGAGCTCCGGATGGTATCCATGTTCCCCCGCTTGAGTGCACATGTC
CTCTCCACATACTTGACAAAACCGACGGTGAACATCAGGATAGAAGCCAGCATGACAAAGAACCCGTTGTCAGCAATGCGTTTGTAC
AGAACATAGCCTGCTCCGAGGACTTGAACGACGAGAACCTGCAGGTGACGCAACCAGAGCTGGTTATCCTGCAGGGCATAGGCAGT
TATGCTGTCAGGACCGCCTAGGTGCAGCAGGAGGAACGGCGCCCAGAAGGCGACAAGCTTCTCATTCTCGCGTGCTTTGCTGAGGG
AGAGGTGGCCGAGGGCATATATCGCAGTGGAGTCAGCCTGCATGTACGCCAACCACAGCAGGATCCTCAGCACAGGATTGGCTCCG
CGTCGACGGATCCTGGCGAAGACGAAGAGGAAGATCTGGAGTGCGAGGCTCAAGAGCACAAGGATCTGGATCGACCAATGATTCC
AGAGGTCCAGCGGCCCTCCAGACATTGTTCCGGCGTGCATGCAATGACCCTGTTTCATGAAATATACATACGTGTATAACTGACGCAG
CTAAACTACATATTATCTCATATTCCTTGTTGAGAAATAGCAATGCATACTTGAGAATATTCTGGACTACTCTGGATAATTGGAGAGTG
TTGGGAATTGGTTATGCATATTTGAGAAGATTCCAGAATAGTTTGGATTAGTGGAGGAGAAGATATTTACAAGGAGCTTTCCAGTGA
ATATGAGAAGATATGAATATTCTAGAAGCCTTGAGAGGTTACTTGAAGAGTGTTGCAATTTACCATGAAAGGACATAATTAAGATAA
GTTTCAAGAATAAGTGAGAATTCTCTAGAACTAGGATACATGTAGAGTATTCTAGAGAATTGTAGTGGGATGTACTACATGGTCTTGT
ACAAGTATAAGTAGAGGTGCCCCCACCTCATAAGTGTACCACAATCACCACAAGTAGTGTATCACCACACCAAGTAGAGAAGTGAGC
TACCATGGTAGGGTTGTGTGTAGGGTAGCCACTAAAGAGAGTGTTGTACCAACATTAAAATAGTGAATAAAGTGTTGAGTTTCCACA
AGTTTGATATCCCAAGTAGTGTAGTGTGCAAAGGTCCTCAAGAGTGGGTGTTAGGGTCGCCGCCCGTAAAATCTCTACATTCCTCAAC
AAAAGATGTCTTAAGTTTGTCAAAATTTGGATGTATCTAGACATGATTTAGTGTATAGGTGCATTCAAATTTGGTCAAAGTTGAGATA
TCTTTTGTTGGACGGAGGGAGCACATGATTCACAAGGGACGATGTATAGTACCTGGCAGTAGTAATGTTGCAGCTAGCAAAGACGG
ATAAAACCAGTCAATCGTCCAAGATAGGGGATGCAGTGATGAACTGGAAACAAGAAGGCAGACAGTAACTTGAGTCTGGCAAGGA
AGAGGCAGGTAATAACTCATCATCTCATACTACACATGAGTATTTGCTGTGTAAGCATACAAGCTGCATTCAGGTATTCAACTGACCA
GCAATAGTATATGTGCGCAATTTTTATGGTTCCATCCAATAGATGACACTACAATTTCCAATTTCAGACCATGTAGACCAGCAAGAAA
GCCAAGTCGGAAGCTTTTGGGACCCTTTTTAGCTAGTACAGAAGAAGACAAGGAAGCTATGTGATCTATTGCAAATAGTTGCCAGCA
GGTTCCAATTAATATACTCTCAGAAACTTCTCTTTGTGTCCATGAGAACTATGGCATTACACAACTATAATATAGACACAGTACTAGGT
TTACAGTGATCCAAGTTGCATCGCCAGGATGAGATTAAAGTAAATGCAAATTAGTAATGGGACTGACTATAGCTCAGTCGATTTCTCA
GTCAAACAGGCAATCAATGGGTTTGTGTCCTCTAATTGGTACGGTGTTGGTTCTATTTATTTATTTTTGTCTCAAGCAATAGCAGGCTG
GTAGCTTAGATTTTTCTTTTGGGTTTCTTTGTTGAAGGTCAATCTTGGCCTGAGCGTGTGGAGTTTCAAATTACGCTCTAAAATAAGTC
CCCTTTGGTGTGGGGCTTTCGTACACTAATAATTTTTGTACGGTGCTAAAAAAAACTTGAATTTTTTTTCAATTTTTTTTGACAAATTTG
AAGAAGGTTAT

>BdiBd21-3.5G0010200

TGATTAATCTGTATCCTTTGCTACTATGTTCACCGACTTCCCAATAACATGTTATTCTATTATACTTGATTGATAACTGAACATGCATTTG
CAATATATATTCTGTGGAAATGTACTCCTGATCACGGTGAGTCTGATTATTGGATGATATACTAGTCAGACTTCACATGTAATATATCT
AGCAGTACAATTGTACCACCAAGAATGAAGTTTGAGAAAAATAATACAAATTGAAGTGGCAAGGACGATATCCTAGCTAGATTTACA
GCTCATCCCTCTCCTCTCCTTCTGTGTGCTTCTTACCCCTCTTGATCCCCCAAGAAGGATGGATTACAAATTATAATGCACCTGTTCCTT
GTCTTGATCACCCGAGATAGTGAAATGAAACTAGTTAGTTGTAGTTCCCATGATCATGCACTGTGTGGACTAATTAAACAGACGTGG
GAGGAAATTAATATGACAAACTATGTATATGGACTAATGGACAGAGGAGCCATGATATAGACTCTGACATGACGAGCAGTTGTGTAC
TTGTGTTAATTTACATCCAGATTTAGACTGATGGATGGCATGCTGGTAAGTGTTGGGACATCATATCGCTCGCTCTGGCAGTGTCCTC
ATCTGCTAACTGGCGCCCTTGCTGACTGCAATTCCCCTCTTGTTAGCTCGGCATCGCCGAACCATCCTCCATATCTATACTACCGTGTTG
TGCAATTATCAGCATTCATAAATGATCAAAGTTAGCTTGTTCAGGTTGTCCGAGTGCCATGTCGCTCCGAGCAGGTATTCCCATGTGTC
TTTGAACCTTGCCATCGCCTTTAGCACGATGGTTGTGGTTGCGAAGCGTCTCCTGTTGAACTCTACTTTGGAGTCTGAGAGGTTCATCT
CCTCCTTCTCCGACAGGTAAGTTGTGAGAAGAGAACTGGGAAACCAAGAGAGAAGAGGGTATCCTGCATTACTCATTAAGCAACACT
CATAGTAGTAGACTAGTAGTTGTCAAGTCTTTGTTGATATTCATGTGGACAGGGAAAGAACGAAATGGATGCAAAATCTATCCACAG
TTACGACGTGGACTGAACTGAACCAATTTTCTCATCTCCAAAAGTGCAATCAATATTTCTGCTGGCTGCCCATCAGAAGAAGGCGGAG
CAGAGCAAGTGAACAACCCTGGGCGGCGCCCCCGTCGTCAGGGAGGAGGAGGAAGGCGGCAAGCGATCGTTCAACGCGGTGCCAC
TGGCCGGCGTGCGCGTGGACGTCGCCGAGCGCGGCCGCGCCCTCTGCTCGATGCGCATCCCCGCCCAACCTCACGGTACGCACCACC
CTGATGCCTGATCCCCCTCCATCTCTGCATCTCGATCTGATTCCCCTGGTACGATCCGCTGGGCCTCTCCACATTCAGTGGAGGGAAAA
GGAGAAGAAACCAAAATGATCACTCATTTTAAGGGCCTATTCGGCATCACTGTGAATTCTAAAACTCCGCGGATCTCGAAATGCGGA
TCGGACCACCTGCTAGCCTAGTTCTGGCTTCTGACCCCGCTGCATCGTTCCAAACCTCACCACGCAGCCGCCGCCTCCATCCTGCCCAC
CTTCAACCACAGCCGTCCCACGGCGAGGTAAAGAAAAGCCGTGAACCCCACCAGCACCGGCCGGGGAGGATGAGTGCCAGCGCGG
GCTTCGTCGATGAGGGGCCGGGAGGAGGTGGGCGAGCTCAGGGCACCCACGGCGGGGACAGCCCAACAATCCTCCCTCGCCGCCA
CGCTCACCAGGGGGACCTGTCAACCAAGCAGGCCTCCTGGAACAGCGTGCCACGGCTTGGGGAAGCGAGGAGGAGGTACAGGCCA
TGTGTGGGACGAGAGTGGCAGGGAGAGAGGCATAATCGTGGTGCTGAGGAATGTCCGTTGTTACAAGCACACGCATGGCATCCTCC
TCCCTGTGGATATCAGCAATGTTGCGTGCATAAATGAGCCCAGGAACTCAGGAATGTTCCCCGGTTCAACCTCTTTTTCAGGCCCAGA
AGATTTCAAGCCATAGCGTGGTCGGAACAGTTTCTTGAGCGCATTGCAGACATTACCAGGAGAATCCACGAGGTGTTTTTAATCCCAT
CCAGATTGTTGCACGTTTGCTTGTACTCCCCTCCTCCCTTGCTTCTTCCCTCACCACTGCTGAGCAAAGCCCGTGCGGTGCGGTGAGGT
GCCAGCACCCCTTACTCCTGGGGGGCCTCTTCTAAATTAAAGCGCATATAAGAGAATCTGAGACCTCTCTCCTGAAAGTAGTTCACAA
GAGGTAAAGCTAATTCTAATGCAAAAGATAGCCAGGTAAATCGTTCACATTTGGAGAAAGCAAATCTGTACCATACATGTCTTTGTTT
CCAGCGAAACCATGAGATTCAGGAACCAGCTTTGGCTAGTGCTGATGGGGATACAGTAATGTTATGTCCACGCTTTATCGATCTCCAC
GTGTACATGATTCATGAATCTTCTGGTGCTTTGAGGACAGACTGTTTGTGAAAGTAGTCCGCCATAAGCCACAAGACGGTTGTCAGCT
CACCGCCACTGCCGAGTCTCTTGGCATGGGACTCCCTGCTGCATCTATTGGCAGTGTAGACAAGAAAATCAGTCCACACATGTCGGAT
CAGTTCCAAGACAGCCATTCTGTCCTTCTCCTCTTCTTGTTTGAGTAGTGCTCGAGTAAGCCAGTTAGCATAACATAGGCGAGGAACA
TCTGTGCTGTAGCCTGGCTTCTCTGTGTTGAGCATGATGGCAAGATCGTCTCTTTCCAATAGTCGAGAAGGATTAGGGTCGTCATGCA
GCCAGAATAATTCCTTCACCTTAGTCCAGCTGATACTGCGACTTGAGGTCTCTGCAGTACACATGTCAACCAGATTCTGGCAGGTCTG
TTGGTACAGTCTGGTCTGAGCAAGGCCTGGTAGCATTTCGGGCCGCTCAACAAGGAGGAACATCATGTAGTTGGACATTGTCCTAAT
GATCTCCACCACTTTCTCCTGGTCTGCATCATATGCCTTGTCTCGACTGCTTTTGGCAAGGAACACATCTGTGCCAATGTGCCAGATAA
TGATGCCCTCTTGGAGCTCAATGCCAAGAGAGTCTTTGAAGTCTTTGAATTCGACACCATCATAACATTTATAATACTGCAATGCCTCT
ACGCCCCAATTTGTCCTGATCATGCCTTGCGTGTTCACCTTGTTTTTCCTGTGCAATCGTTGTATGTACCAGTGCAGCCATAGCCTTAGC
TCATCTGAAATCTGAATGGTCCCTGAGTAATGCTTTCTGTTCCACCACTCATTTTGTCCCACCATCTTTGCCAATCTACCGAGAAGAGG
ACTGAAGGCTGTGTCACGACGGCTGCAAAGTTGCAGCATGTTGTATTGCCCCATTTCACCGGACCACCTCCTTGCCTGACCACCAACT
TGTATTCTCGTGATCTTCTTGACAAGCTGACGAAGCTGATCCCACCTTCCACTGCATAGAGCAGTTTTACGGAGCCATCTCCAACGTGT
GGTGCACAAGAAGGTGTATGTCCAACTTGACCCTAGCGCGCTCAACAAGGATGTTGCTTCCAGGATGAAAGCCCCAGACAGCAAGG
TGTATGTGATACCAACATCAACTTTGCTGTCACCATCGTTGTCACTGAAGTAGAACAGCAGCAAGGAGGCGACAGTGGCAAGTGGCG
AGACAATACGGATGCTGTAGCCAGGCCAAGTGTGGATCACAGCTGCCTTGGTATACAGGATGTCATACAACAGGGAGAGCTCCACCT
CCATCAACATCCACATACCCTTGTATTTTTCATTTTCGATGTCTCTAAGCATCTCGCCACCACGGTTTTCAAAATCCTTTTCAATCCAAGA
ATCAACTATGGCACGCTTGCATACGTGGAACATGGAGTGAGCAAGACGCACATAGTGTTCTTCCTTGTTGGCGGCCCCCTCTCTGAGC
CCTTGATCCAGAGTGCGGAACTGTTTACGTTGGACATATGGCTCATTCTTGAGAGAGCTCCGGATGGTATCCATGTTCCCCCGCTTGA
GTGCACATGTCCTCTCCACATACTTGACAAAACCGACGGTGAACATCAGGATAGAAGCCAGCATGACAAAGAACCCGTTGTCAGCAA
TGCGTTTGTACAGAACATAGCCTGCTCCGAGGACTTGAACGACGAGAACCTGCAGGTGACGCAACCAGAGCTGGTTATCCTGCAGG
GCATAGGCAGTTATGCTGTCAGGACCGCCTAGGTGCAGCAGGAGGAACGGCGCCCAGAAGGCGACAAGCTTCTCATTCTCGCGTGC

TTTGCTGAGGGAGAGGTGGCCGAGGGCATATATCGCAGTGGAGTCAGCCTGCATGTACGCCAACCACAGCAGGATCCTCAGCACAG
GATTGGCTCCGCGTCGACGGATCCTGGCGAAGACGAAGAGGAAGATCTGGAGTGCGAGGCTCAAGAGCACAAGGATCTGGATCGA
CCAATGATTCCAGAGGTCCAGCGGCCCTCCAGACATTGTTCCGGCGTGCATGCAATGACCCTGTTTCATGAAATATACATACGTGTAT
AACTAACGCAGCTAAACTACATATTATCTCATATTCCTCTATCCAACAAAAGATGTCTTAAGTTTGTCAAAATTTGGATGTATCTAGAC
ATGATTTAGTGTATAGGTGCATTCAAATTTGATCAAAGTTGAGACATGTTTTGTTGGACGGAAGGAGTACATGATTCACAAGGGACG
ATGTATAGTACCTGGTAGTAGTAATGTTGCAGCTAGTACAGAAGGATAAAACCAGTCAATCGTCCAAGATAGGGGATGCAGTGATG
AACTGGAAACAAGAAGGCAGACAGTAACTTGAGTCTGGCAAGGAAGAGGCAGGTAATAACTCATCATCTCATACTACACATGAGTA
TTTGCTGTGTAAGCATACAAGCTGCATTCAGGTATTCAACTGACCAGCAATAGTATATGTGCGCAATTTTTATGGTTCCATCCAATAGA
TGACACTACAATTTCCAATTTCAGACCATGTAGACCAGCAAGAAAGCCAAGTCGGAAGCTTTTGGGACCCTTTTTAGCTAGTACAGAA
GAAGACAAGGAAGCTACGTGATCTATTGCAAATAGTTGCCAGCAGGTTCCAATTAATATACTCTCAGAAACTTCTCTTTGTGTCCATG
AGAACTATGGCATTACACAACTATAATATAGACACAGTACTAGGTTTACAGTGATCCAAAGTTGCATCGCCAGGATGAGATTAAAGT
AAATGCAAATTAGTAATGGAACTGACTATAGCTCAGTCGATTTCTCAGTCAAACAGGCAATCAATGGGTTTGTGTCCTCTAATTGGTA
CGGTGTTGGTTCTATTTATTTATTTTTGTCTCAAGCAATAGCAGGCTGGTAGCTTAGATTTGTCTTTTGGGTTTCTTTGTTGAAGGTCAA
TCTTGGCCTGAGCGTGTGGAGTTTCAAATTACGTTCCACTATACCATTTTGTACATGCACGAAAATAAACATAAATCCAGTCCCCTTTG
GTGTGGGGCTTTCGTACACTAGTATTTTTTGTACGGTGCTAAAAAAAACTTGAATTTTATTTCAATTTTTTTTTGACAAATTTGAAGAAG
GTTATTTGAACGCCTTTATTCAAGTATATAAAGAAGAAAGCATGAAAAAAGTATTTTAATAATGACAGCACATTTGATCAAATCAAGT
TGCTCAAAATAGTAAGTGCTAGCCTGAGTGCTTTGCAACGTGCAAACCTAAGTTGCGGTTATAAAAACATCAAGTTTAAATTTTTTAG
CAAAGTTGGACTATCATGGTTGTTGGGTCCACATGTACATACGAGGATAGGCCAAGTGCCTGCAAATTTTTATTACGTAATACTCTCT
CCGTTTCATAATTTTTGTCGAAATATTACATGTATCTAGACACGTTTTCTAAGAATAGATACATTCATTTTAGAGCAAATTTGAGACAA
GAATTATGAAACATTTTTGTCAGGGAGTAGGTCTCCTTCCTCCCTAGGCCCGACTACTCTCTACAAGAAGCTTATCCTAGGGATGGATT
TAGCTATAGCTAGGCCAAGAATACCAACTCTAATTGCAGATCAATCAGTAAACGTGCATTGGCCTACAGCCCGGCTGGCCGGCCGTA
CGCTGTAAGCCTGTAATACTGATACATATCATACGGATCAGAATACATACCTTGCCGGCTACACGATGGATGGTTGGCCAAGCCGTAT
ACCCTCGTGACATCGATCAGTCTAGCTATCTTACTAGCTTGCTTGCTGCTCATGCAGGACGCCATTGCGGCCGGGTCAATATATATAA
AGCTTGTATAGCTGCAGTTGACTTCGTCCCGTCAACGCCTCCAGCTTTTATTTTGTTTTCTTCTCCTTACTAGTTGGTAATATGTTTTCAA
TAAAATTAGTTTTTACGAGTGCCGCACACTTTTTAGTGGACCTAGGAATTACTGGCGAGCCGGCAGGCCGCTCCCGAATTGCGAGCG
ACCAACGATTTGCGGCGGCCATCGTCGTGATTTGCGGCGGCCGGCAACTGCAACTGGGGCACACAAGTTGTACGACGAGATGGGTT
TG

```
Alignment of Sequence_1:  [Untitled Sequence #1] with  Sequence_2: [Sequence Window #2]

Similarity : 5554/6590 (84.28 %)

Seq_1  1      TGATTAATCTGTATCCTTTGCTACTATGTTCACCGACTTCCCAATAACATGTTATTCTAT  60
              ||||||||||||||||||||||||||||||||||||||||||||||||||||||||||||
Seq_2  1      TGATTAATCTGTATCCTTTGCTACTATGTTCACCGACTTCCCAATAACATGTTATTCTAT  60


Seq_1  61     TATACTTGATTGATAACTGAACATGCATTTGCAATATATATTCTGTGGAAATGTACTCCT  120
              ||||||||||||||||||||||||||||||||||||||||||||||||||||||||||||
Seq_2  61     TATACTTGATTGATAACTGAACATGCATTTGCAATATATATTCTGTGGAAATGTACTCCT  120


Seq_1  121    GATCACGGTGAGTCTGATTATTGGATGATATACTAGTCAGACTTCACATGTAATATATCT  180
              ||||||||||||||||||||||||||||||||||||||||||||||||||||||||||||
Seq_2  121    GATCACGGTGAGTCTGATTATTGGATGATATACTAGTCAGACTTCACATGTAATATATCT  180


Seq_1  181    AGCAGTACAATTGTACCACCAAGAATGAAGTTTGAGAAAAATAATACAAATTGAAGTGGC  240
              ||||||||||||||||||||||||||||||||||||||||||||||||||||||||||||
Seq_2  181    AGCAGTACAATTGTACCACCAAGAATGAAGTTTGAGAAAAATAATACAAATTGAAGTGGC  240


Seq_1  241    AAGGACGATATCCTAGCTAGATTTACAGCTCATCCCTCTCCTCTCCTTCTGTGTGCTTCT  300
              ||||||||||||||||||||||||||||||||||||||||||||||||||||||||||||
Seq_2  241    AAGGACGATATCCTAGCTAGATTTACAGCTCATCCCTCTCCTCTCCTTCTGTGTGCTTCT  300


Seq_1  301    TACCCCTCTTGATCCCCCAAGAAGGATGGATTACAAATTATAATGCACCTGTTCCTTGTC  360
              ||||||||||||||||||||||||||||||||||||||||||||||||||||||||||||
```

```
Seq_2  301   TACCCCTCTTGATCCCCCAAGAAGGATGGATTACAAATTATAATGCACCTGTTCCTTGTC  360


Seq_1  361   TTGATCACCCGAGATAGTGAAATGAAACTAGTTAGTTGTAGTTCCCATGATCATGCACTG  420
             ||||||||||||||||||||||||||||||||||||||||||||||||||||||||||||
Seq_2  361   TTGATCACCCGAGATAGTGAAATGAAACTAGTTAGTTGTAGTTCCCATGATCATGCACTG  420


Seq_1  421   TGTGGACTAATTAAACAGACGTGGGAGGAAATTAATATGACAAACTATGTATATGGACTA  480
             ||||||||||||||||||||||||||||||||||||||||||||||||||||||||||||
Seq_2  421   TGTGGACTAATTAAACAGACGTGGGAGGAAATTAATATGACAAACTATGTATATGGACTA  480


Seq_1  481   ATGGACAGAGGAGCCATGATATAGACTCTGACATGACGAGCAGTTGTGTACTTGTGTTAA  540
             ||||||||||||||||||||||||||||||||||||||||||||||||||||||||||||
Seq_2  481   ATGGACAGAGGAGCCATGATATAGACTCTGACATGACGAGCAGTTGTGTACTTGTGTTAA  540


Seq_1  541   TTTACATCCAGATTTAGACTGATGGATGGCATGCTGGTAAGTGTTGGGACATCATATCGC  600
             ||||||||||||||||||||||||||||||||||||||||||||||||||||||||||||
Seq_2  541   TTTACATCCAGATTTAGACTGATGGATGGCATGCTGGTAAGTGTTGGGACATCATATCGC  600


Seq_1  601   TCGCTCTGGCAGTGTCCTCATCTGCTAACTGGCGCCCTTGCTGACTGCAATTCCCCTCTT  660
             ||||||||||||||||||||||||||||||||||||||||||||||||||||||||||||
Seq_2  601   TCGCTCTGGCAGTGTCCTCATCTGCTAACTGGCGCCCTTGCTGACTGCAATTCCCCTCTT  660


Seq_1  661   GTTAGCTCGGCATCGCCGAACCATCCTCCATATCTATACTACCGTGTTGTGCAATTATCA  720
             ||||||||||||||||||||||||||||||||||||||||||||||||||||||||||||
Seq_2  661   GTTAGCTCGGCATCGCCGAACCATCCTCCATATCTATACTACCGTGTTGTGCAATTATCA  720


Seq_1  721   GCATTCATAAATGATCAAAGTTAGCTTGTTCAGGTTGTCCGAGTGCCATGTCGCTCCGAG  780
             ||||||||||||||||||||||||||||||||||||||||||||||||||||||||||||
Seq_2  721   GCATTCATAAATGATCAAAGTTAGCTTGTTCAGGTTGTCCGAGTGCCATGTCGCTCCGAG  780


Seq_1  781   CAGGTATTCCCATGTGTCTTTGAACCTTGCCATCGCCTTTAGCACGATGGTTGTGGTTGC  840
             ||||||||||||||||||||||||||||||||||||||||||||||||||||||||||||
Seq_2  781   CAGGTATTCCCATGTGTCTTTGAACCTTGCCATCGCCTTTAGCACGATGGTTGTGGTTGC  840


Seq_1  841   GAAGCGTCTCCTGTTGAACTCTACTTTGGAGTCTGAGAGGTTCATCTCCTCCTTCTCCGA  900
             ||||||||||||||||||||||||||||||||||||||||||||||||||||||||||||
Seq_2  841   GAAGCGTCTCCTGTTGAACTCTACTTTGGAGTCTGAGAGGTTCATCTCCTCCTTCTCCGA  900


Seq_1  901   CAGGTAAGTTGTGAGAAGAGAACTGGGAAACCAAGAGAGAAGAGGGTATCCTGCATTACT  960
             ||||||||||||||||||||||||||||||||||||||||||||||||||||||||||||
Seq_2  901   CAGGTAAGTTGTGAGAAGAGAACTGGGAAACCAAGAGAGAAGAGGGTATCCTGCATTACT  960


Seq_1  961   CATTAAGCAACACTCATAGTAGTAGACTAGTAGTTGTCAAGTCTTTGTTGATATTCATGT  1020
             ||||||||||||||||||||||||||||||||||||||||||||||||||||||||||||
Seq_2  961   CATTAAGCAACACTCATAGTAGTAGACTAGTAGTTGTCAAGTCTTTGTTGATATTCATGT  1020


Seq_1  1021  GGACAGGGAAAGAACGAAATGGATGCAAAATCTATCCACAGTTACGACGTGGACTGAACT  1080
             ||||||||||||||||||||||||||||||||||||||||||||||||||||||||||||
Seq_2  1021  GGACAGGGAAAGAACGAAATGGATGCAAAATCTATCCACAGTTACGACGTGGACTGAACT  1080


Seq_1  1081  GAACCAATTTTCTCATCTCCAAAAGTGCAATCAATATTTCTTCTGGCTGCCCATCAGAAG  1140
             ||||||||||||||||||||||||||||||||||||||||| ||||||||||||||||||
Seq_2  1081  GAACCAATTTTCTCATCTCCAAAAGTGCAATCAATATTTCTGCTGGCTGCCCATCAGAAG  1140


Seq_1  1141  AAGGCGGAGCAGAGCAAGTGAACAACCCTGGGCGGCGCCCCCGTCGTCAGGGAGGAGGAG  1200
             ||||||||||||||||||||||||||||||||||||||||||||||||||||||||||||
```

```
Seq_2  1141  AAGGCGGAGCAGAGCAAGTGAACAACCCTGGGCGGCGCCCCCGTCGTCAGGGAGGAGGAG  1200

Seq_1  1201  GAAGGCGGCAAGCGATCGTTCAACGCGGTGCCACTGGCCGGCGTGCGCGTGGACGTCGCC  1260
             ||||||||||||||||||||||||||||||||||||||||||||||||||||||||||||
Seq_2  1201  GAAGGCGGCAAGCGATCGTTCAACGCGGTGCCACTGGCCGGCGTGCGCGTGGACGTCGCC  1260

Seq_1  1261  GAGCGCGGCCGCGCCCTCTGCTCGATGCGCATCCCCGCCCAACCTCACGGTACGCACCAC  1320
             ||||||||||||||||||||||||||||||||||||||||||||||||||||||||||||
Seq_2  1261  GAGCGCGGCCGCGCCCTCTGCTCGATGCGCATCCCCGCCCAACCTCACGGTACGCACCAC  1320

Seq_1  1321  CCTGATGCCTGATCCCCCTCCATCTCTGCATCTCGATCTGATTCCCCTGGTACGATCCGC  1380
             ||||||||||||||||||||||||||||||||||||||||||||||||||||||||||||
Seq_2  1321  CCTGATGCCTGATCCCCCTCCATCTCTGCATCTCGATCTGATTCCCCTGGTACGATCCGC  1380

Seq_1  1381  TGGGCCGCTCCACATTCAGTGGAGGGAAAAGGAGAAGAAACCAAAATGATCACTCATTTT  1440
             |||||| |||||||||||||||||||||||||||||||||||||||||||||||||||||
Seq_2  1381  TGGGCCTCTCCACATTCAGTGGAGGGAAAAGGAGAAGAAACCAAAATGATCACTCATTTT  1440

Seq_1  1441  AAGGGCCTATCCGGTATCACTGTGAATTCTAAAACTCCGCGGATCTCGAAATGCGGATCG  1500
             |||||||||| ||| |||||||||||||||||||||||||||||||||||||||||||||
Seq_2  1441  AAGGGCCTATTCGGCATCACTGTGAATTCTAAAACTCCGCGGATCTCGAAATGCGGATCG  1500

Seq_1  1501  GACCACCTGCTAGCCTATTTTTGCGGAGCGATGATTTGCGGCCTCTAGTTCTGGCTTCTG  1560
             |||||||||||||||||                              |||||||||||||
Seq_2  1501  GACCACCTGCTAGCCTA------------------------------GTTCTGGCTTCTG  1530

Seq_1  1561  ACCCCGCTGCATCGTTCCAAACCTCACCACGCAGCCGCCGCCTCCATCCTGCCCACCTTC  1620
             ||||||||||||||||||||||||||||||||||||||||||||||||||||||||||||
Seq_2  1531  ACCCCGCTGCATCGTTCCAAACCTCACCACGCAGCCGCCGCCTCCATCCTGCCCACCTTC  1590

Seq_1  1621  AACCACAGCCGTCCCGTCTCTATCCTGTCCATTCCCGCTCCCCATTCGACCGAGAGGAAC  1680
             |||||||||||||||
Seq_2  1591  AACCACAGCCGTCCC---------------------------------------------  1605

Seq_1  1681  CTA--GCGAGGTAAAAAAAAGCCGTGAACCCCACCAGCACCGGCCGGGGAGGATGAGTGC  1738
               |  |||||||||| ||||||||||||||||||||||||||||||||||||||||||||
Seq_2  1606  --ACGGCGAGGTAAAGAAAAGCCGTGAACCCCACCAGCACCGGCCGGGGAGGATGAGTGC  1663

Seq_1  1739  CAGCGCGGGCTTCGTCGATGAGGGGCCGGGAGGAGGTGGGCGAGCTCAGGGCACCCACGG  1798
             ||||||||||||||||||||||||||||||||||||||||||||||||||||||||||||
Seq_2  1664  CAGCGCGGGCTTCGTCGATGAGGGGCCGGGAGGAGGTGGGCGAGCTCAGGGCACCCACGG  1723

Seq_1  1799  CGGGGACAGCCCAACAATCCTCCCTCGCCGCCACGCTCACCAGGGGGACCTGTCAACCAA  1858
             ||||||||||||||||||||||||||||||||||||||||||||||||||||||||||||
Seq_2  1724  CGGGGACAGCCCAACAATCCTCCCTCGCCGCCACGCTCACCAGGGGGACCTGTCAACCAA  1783

Seq_1  1859  GCAGGCCTCCTGGAACAGCGTGCCACGGCTTTGGAGAAGCGAGGAGGAGGTACAGGCCAT  1918
             ||||||||||||||||||||||||||||||| || ||||||||||||||||||||||||
Seq_2  1784  GCAGGCCTCCTGGAACAGCGTGCCACGGCTT-GGGGAAGCGAGGAGGAGGTACAGGCCAT  1842

Seq_1  1919  GTGTGGGACGAGAGTGGCAGGGAGAGAGGCATAATCGTGGTGCTGAGGAATGTCCGTTGT  1978
             ||||||||||||||||||||||||||||||||||||||||||||||||||||||||||||
Seq_2  1843  GTGTGGGACGAGAGTGGCAGGGAGAGAGGCATAATCGTGGTGCTGAGGAATGTCCGTTGT  1902

Seq_1  1979  TACAAGCACACGCACGGCATCCTCCTCCCTGTGGATATCAGCAATGTTGCGTGCATAAAT  2038
             |||||||||||||| |||||||||||||||||||||||||||||||||||||||||||||
```

```
Seq_2  1903  TACAAGCACACGCATGGCATCCTCCTCCCTGTGGATATCAGCAATGTTGCGTGCATAAAT  1962

Seq_1  2039  GAGCCCAGGAACTCAGGAATGTTCCCCGGTTCAACCTCCTTTTCAGGCCCAGAAGATTTC  2098
             |||||||||||||||||||||||||||||||||||||| |||||||||||||||||||||
Seq_2  1963  GAGCCCAGGAACTCAGGAATGTTCCCCGGTTCAACCTCTTTTTCAGGCCCAGAAGATTTC  2022

Seq_1  2099  AAGCCATAGCGTGGTCGGAACAGTTTCTTGAGCGCATTGCAGACATTACCAGGAGAATCC  2158
             ||||||||||||||||||||||||||||||||||||||||||||||||||||||||||||
Seq_2  2023  AAGCCATAGCGTGGTCGGAACAGTTTCTTGAGCGCATTGCAGACATTACCAGGAGAATCC  2082

Seq_1  2159  ACGAGGTGTTTTTAATCCCATCCAGATTGTTGCACGTTTGCTTGTACTCCCCTCCTCCCT  2218
             ||||||||||||||||||||||||||||||||||||||||||||||||||||||||||||
Seq_2  2083  ACGAGGTGTTTTTAATCCCATCCAGATTGTTGCACGTTTGCTTGTACTCCCCTCCTCCCT  2142

Seq_1  2219  TGCTTCTTCCCTCATCACTGCTGAGCAAAGCCCGTGCGGTGCGGTGAGGTGCCAGCACCC  2278
             |||||||||||||| |||||||||||||||||||||||||||||||||||||||||||||
Seq_2  2143  TGCTTCTTCCCTCACCACTGCTGAGCAAAGCCCGTGCGGTGCGGTGAGGTGCCAGCACCC  2202

Seq_1  2279  CTTACTCCTGGGGGGGCTCTTCTAAATTAAAGCGCATATAAGAGAATCTGAGACCTCTCT  2338
             ||||||||||||||| ||||||||||||||||||||||||||||||||||||||||||||
Seq_2  2203  CTTACTCCTGGGGGGCCTCTTCTAAATTAAAGCGCATATAAGAGAATCTGAGACCTCTCT  2262

Seq_1  2339  CCTGAAAGTAGTTCACAAGAGGTAAAGCTAATTCTAATGCAAAAGATAGCCAGGTAAATC  2398
             ||||||||||||||||||||||||||||||||||||||||||||||||||||||||||||
Seq_2  2263  CCTGAAAGTAGTTCACAAGAGGTAAAGCTAATTCTAATGCAAAAGATAGCCAGGTAAATC  2322

Seq_1  2399  GTTCACATTTGGAGAAAGCAAATCTGTACCATACATGTCTTTGTTTCCAGCGAAACCATG  2458
             ||||||||||||||||||||||||||||||||||||||||||||||||||||||||||||
Seq_2  2323  GTTCACATTTGGAGAAAGCAAATCTGTACCATACATGTCTTTGTTTCCAGCGAAACCATG  2382

Seq_1  2459  AGATTCAGGAACCAGCTTTGGCTAGTGCTGATGGGGATACAGTAATGTTATGTCCACGCT  2518
             ||||||||||||||||||||||||||||||||||||||||||||||||||||||||||||
Seq_2  2383  AGATTCAGGAACCAGCTTTGGCTAGTGCTGATGGGGATACAGTAATGTTATGTCCACGCT  2442

Seq_1  2519  TTATCGATCTCCACGTGTACATGATTCATGAATCTTCTGGTGCTTTGAGGACAGACTGTT  2578
             ||||||||||||||||||||||||||||||||||||||||||||||||||||||||||||
Seq_2  2443  TTATCGATCTCCACGTGTACATGATTCATGAATCTTCTGGTGCTTTGAGGACAGACTGTT  2502

Seq_1  2579  TGTGAAAGTAGTCCGCCATAAGCCACAAGACGGTTGTCAGCTCACCGCCACTGCCGAGTC  2638
             ||||||||||||||||||||||||||||||||||||||||||||||||||||||||||||
Seq_2  2503  TGTGAAAGTAGTCCGCCATAAGCCACAAGACGGTTGTCAGCTCACCGCCACTGCCGAGTC  2562

Seq_1  2639  TCTTGGCATGGGACTCCCTGCTGCATCTATTGGCAGTGTAGACAAGAAAATCAGTCCACA  2698
             ||||||||||||||||||||||||||||||||||||||||||||||||||||||||||||
Seq_2  2563  TCTTGGCATGGGACTCCCTGCTGCATCTATTGGCAGTGTAGACAAGAAAATCAGTCCACA  2622

Seq_1  2699  CATGTCGGATCAGTTCCAAGACAGCCATTCTGTCCTTCTCCTCTTCTTGTTTGAGTAGTG  2758
             ||||||||||||||||||||||||||||||||||||||||||||||||||||||||||||
Seq_2  2623  CATGTCGGATCAGTTCCAAGACAGCCATTCTGTCCTTCTCCTCTTCTTGTTTGAGTAGTG  2682

Seq_1  2759  CTCGAGTAAGCCAGTTAGCATAACATAGGCGAGGAACATCTGTGCTGTAGCCTGGCTTCT  2818
             ||||||||||||||||||||||||||||||||||||||||||||||||||||||||||||
Seq_2  2683  CTCGAGTAAGCCAGTTAGCATAACATAGGCGAGGAACATCTGTGCTGTAGCCTGGCTTCT  2742

Seq_1  2819  CTGTGTTGAGCATGATGGCAAGATCGTCTCTTTCCAATAGTCGAGAAGGATTAGGGTCGT  2878
             ||||||||||||||||||||||||||||||||||||||||||||||||||||||||||||
```

```
Seq_2  2743  CTGTGTTGAGCATGATGGCAAGATCGTCTCTTTCCAATAGTCGAGAAGGATTAGGGTCGT  2802

Seq_1  2879  CATGCAGCCAGAATAATTCCTTCACCTTAGTCCAGCTGATACTGCGACTTGAGGTCTCTG  2938
             ||||||||||||||||||||||||||||||||||||||||||||||||||||||||||||
Seq_2  2803  CATGCAGCCAGAATAATTCCTTCACCTTAGTCCAGCTGATACTGCGACTTGAGGTCTCTG  2862

Seq_1  2939  CAGTACACATGTCAACCAGATTCTGGCAGGTCTGTTGGTACAGTCTGGTCTGAGCAAGGC  2998
             ||||||||||||||||||||||||||||||||||||||||||||||||||||||||||||
Seq_2  2863  CAGTACACATGTCAACCAGATTCTGGCAGGTCTGTTGGTACAGTCTGGTCTGAGCAAGGC  2922

Seq_1  2999  CTGGTAGCATTTCGGGCCGCTCAACAAGGAGGAACATCATGTAGTTGGACATTGTCCTAA  3058
             ||||||||||||||||||||||||||||||||||||||||||||||||||||||||||||
Seq_2  2923  CTGGTAGCATTTCGGGCCGCTCAACAAGGAGGAACATCATGTAGTTGGACATTGTCCTAA  2982

Seq_1  3059  TGATCTCCACCACTTTCTCCTGGTCTGCATCATATGCCTTGTCTCGACTGCTTTTGGCAA  3118
             ||||||||||||||||||||||||||||||||||||||||||||||||||||||||||||
Seq_2  2983  TGATCTCCACCACTTTCTCCTGGTCTGCATCATATGCCTTGTCTCGACTGCTTTTGGCAA  3042

Seq_1  3119  GGAACACATCTGTGCCAATGTGCCAGATAATGATGCCCTCTTGGAGCTCAATGCCAAGAG  3178
             ||||||||||||||||||||||||||||||||||||||||||||||||||||||||||||
Seq_2  3043  GGAACACATCTGTGCCAATGTGCCAGATAATGATGCCCTCTTGGAGCTCAATGCCAAGAG  3102

Seq_1  3179  AGTCTTTGAAGTCTTTGAATTCGACACCATCATAACATTTATAATACTGCAATGCCTCTA  3238
             ||||||||||||||||||||||||||||||||||||||||||||||||||||||||||||
Seq_2  3103  AGTCTTTGAAGTCTTTGAATTCGACACCATCATAACATTTATAATACTGCAATGCCTCTA  3162

Seq_1  3239  CGCCCCAATTTGTCCTGATCATGCCTTGCGTGTTCACCTTGTTTTTCCTGTGCAATCGTT  3298
             ||||||||||||||||||||||||||||||||||||||||||||||||||||||||||||
Seq_2  3163  CGCCCCAATTTGTCCTGATCATGCCTTGCGTGTTCACCTTGTTTTTCCTGTGCAATCGTT  3222

Seq_1  3299  GTATGTACCAGTGCAGCCATAGCCTTAGCTCATCTGAAATCTGAATGGTCCCTGAGTAAT  3358
             ||||||||||||||||||||||||||||||||||||||||||||||||||||||||||||
Seq_2  3223  GTATGTACCAGTGCAGCCATAGCCTTAGCTCATCTGAAATCTGAATGGTCCCTGAGTAAT  3282

Seq_1  3359  GCTTTCTGTTCCACCACTCATTTTGTCCCACCATCTTTGCCAATCTACCGAGAAGAGGAC  3418
             ||||||||||||||||||||||||||||||||||||||||||||||||||||||||||||
Seq_2  3283  GCTTTCTGTTCCACCACTCATTTTGTCCCACCATCTTTGCCAATCTACCGAGAAGAGGAC  3342

Seq_1  3419  TGAAGGCTGTGTCACGACGGCTGCAAAGTTGCAGCATGTTGTATTGCCCCATTTCACCGG  3478
             ||||||||||||||||||||||||||||||||||||||||||||||||||||||||||||
Seq_2  3343  TGAAGGCTGTGTCACGACGGCTGCAAAGTTGCAGCATGTTGTATTGCCCCATTTCACCGG  3402

Seq_1  3479  ACCACCTCCTTGCCTGACCACCAACTTGTATTCTCGTGATCTTCTTGACAAGCTGACGAA  3538
             ||||||||||||||||||||||||||||||||||||||||||||||||||||||||||||
Seq_2  3403  ACCACCTCCTTGCCTGACCACCAACTTGTATTCTCGTGATCTTCTTGACAAGCTGACGAA  3462

Seq_1  3539  GCTGATCCCACCTTCCACTGCATAGAGCAGTTTTACGGAGCCATCTCCAACGTGTGGTGC  3598
             ||||||||||||||||||||||||||||||||||||||||||||||||||||||||||||
Seq_2  3463  GCTGATCCCACCTTCCACTGCATAGAGCAGTTTTACGGAGCCATCTCCAACGTGTGGTGC  3522

Seq_1  3599  ACAAGAAGGTGTATGTCCAACTTGACCCTAGCGCGCTCAACAAGGATGTTGCTTCCAGGA  3658
             ||||||||||||||||||||||||||||||||||||||||||||||||||||||||||||
Seq_2  3523  ACAAGAAGGTGTATGTCCAACTTGACCCTAGCGCGCTCAACAAGGATGTTGCTTCCAGGA  3582

Seq_1  3659  GGAAAGCCCCAGACAGCAAGGTGTATGTGATACCAACATCAACTTTGCTGTCACCATCGT  3718
              |||||||||||||||||||||||||||||||||||||||||||||||||||||||||||
```

```
Seq_2  3583  TGAAAGCCCCAGACAGCAAGGTGTATGTGATACCAACATCAACTTTGCTGTCACCATCGT  3642

Seq_1  3719  TGTCACTGAAGTAGAACAGCAGCAAGGAGGCGACAGTGGCAAGTGGCGAGACAATACGGA  3778
             ||||||||||||||||||||||||||||||||||||||||||||||||||||||||||||
Seq_2  3643  TGTCACTGAAGTAGAACAGCAGCAAGGAGGCGACAGTGGCAAGTGGCGAGACAATACGGA  3702

Seq_1  3779  TGCTGTAGCCAGGCCAAGTGTGGATCACAGCTGCCTTGGTATACAGGATGTCATACAACA  3838
             ||||||||||||||||||||||||||||||||||||||||||||||||||||||||||||
Seq_2  3703  TGCTGTAGCCAGGCCAAGTGTGGATCACAGCTGCCTTGGTATACAGGATGTCATACAACA  3762

Seq_1  3839  GGGAGAGCTCCACCTCCATCAACATCCACATACCCTTGTATTTTTCATTTTCGATGTCTC  3898
             ||||||||||||||||||||||||||||||||||||||||||||||||||||||||||||
Seq_2  3763  GGGAGAGCTCCACCTCCATCAACATCCACATACCCTTGTATTTTTCATTTTCGATGTCTC  3822

Seq_1  3899  TAAGCATCTCGCCACCACGGTTTTCAAAATCCTTTTCAATCCAAGAATCAACTATGGCAC  3958
             ||||||||||||||||||||||||||||||||||||||||||||||||||||||||||||
Seq_2  3823  TAAGCATCTCGCCACCACGGTTTTCAAAATCCTTTTCAATCCAAGAATCAACTATGGCAC  3882

Seq_1  3959  GCTTGCATACGTGGAACATGGAGTGAGCAAGACGCACATAGTGTTCTTCCTTGTTGGCGG  4018
             ||||||||||||||||||||||||||||||||||||||||||||||||||||||||||||
Seq_2  3883  GCTTGCATACGTGGAACATGGAGTGAGCAAGACGCACATAGTGTTCTTCCTTGTTGGCGG  3942

Seq_1  4019  CCCCCTCTCTGAGCCCTTGATCCAGAGTGCGGAACTGTTTCCGTTGGACATATGGCTCAT  4078
             |||||||||||||||||||||||||||||||||||||||| |||||||||||||||||||
Seq_2  3943  CCCCCTCTCTGAGCCCTTGATCCAGAGTGCGGAACTGTTTACGTTGGACATATGGCTCAT  4002

Seq_1  4079  TCTTGAGAGAGCTCCGGATGGTATCCATGTTCCCCCGCTTGAGTGCACATGTCCTCTCCA  4138
             ||||||||||||||||||||||||||||||||||||||||||||||||||||||||||||
Seq_2  4003  TCTTGAGAGAGCTCCGGATGGTATCCATGTTCCCCCGCTTGAGTGCACATGTCCTCTCCA  4062

Seq_1  4139  CATACTTGACAAAACCGACGGTGAACATCAGGATAGAAGCCAGCATGACAAAGAACCCGT  4198
             ||||||||||||||||||||||||||||||||||||||||||||||||||||||||||||
Seq_2  4063  CATACTTGACAAAACCGACGGTGAACATCAGGATAGAAGCCAGCATGACAAAGAACCCGT  4122

Seq_1  4199  TGTCAGCAATGCGTTTGTACAGAACATAGCCTGCTCCGAGGACTTGAACGACGAGAACCT  4258
             ||||||||||||||||||||||||||||||||||||||||||||||||||||||||||||
Seq_2  4123  TGTCAGCAATGCGTTTGTACAGAACATAGCCTGCTCCGAGGACTTGAACGACGAGAACCT  4182

Seq_1  4259  GCAGGTGACGCAACCAGAGCTGGTTATCCTGCAGGGCATAGGCAGTTATGCTGTCAGGAC  4318
             ||||||||||||||||||||||||||||||||||||||||||||||||||||||||||||
Seq_2  4183  GCAGGTGACGCAACCAGAGCTGGTTATCCTGCAGGGCATAGGCAGTTATGCTGTCAGGAC  4242

Seq_1  4319  CGCCTAGGTGCAGCAGGAGGAACGGCGCCCAGAAGGCGACAAGCTTCTCATTCTCGCGTG  4378
             ||||||||||||||||||||||||||||||||||||||||||||||||||||||||||||
Seq_2  4243  CGCCTAGGTGCAGCAGGAGGAACGGCGCCCAGAAGGCGACAAGCTTCTCATTCTCGCGTG  4302

Seq_1  4379  CTTTGCTGAGGGAGAGGTGGCCGAGGGCATATATCGCAGTGGAGTCAGCCTGCATGTACG  4438
             ||||||||||||||||||||||||||||||||||||||||||||||||||||||||||||
Seq_2  4303  CTTTGCTGAGGGAGAGGTGGCCGAGGGCATATATCGCAGTGGAGTCAGCCTGCATGTACG  4362

Seq_1  4439  CCAACCACAGCAGGATCCTCAGCACAGGATTGGCTCCGCGTCGACGGATCCTGGCGAAGA  4498
             ||||||||||||||||||||||||||||||||||||||||||||||||||||||||||||
Seq_2  4363  CCAACCACAGCAGGATCCTCAGCACAGGATTGGCTCCGCGTCGACGGATCCTGGCGAAGA  4422

Seq_1  4499  CGAAGAGGAAGATCTGGAGTGCGAGGCTCAAGAGCACAAGGATCTGGATCGACCAATGAT  4558
             ||||||||||||||||||||||||||||||||||||||||||||||||||||||||||||
```

```
Seq_2  4423  CGAAGAGGAAGATCTGGAGTGCGAGGCTCAAGAGCACAAGGATCTGGATCGACCAATGAT  4482

Seq_1  4559  TCCAGAGGTCCAGCGGCCCTCCAGACATTGTTCCGGCGTGCATGCAATGACCCTGTTTCA  4618
             ||||||||||||||||||||||||||||||||||||||||||||||||||||||||||||
Seq_2  4483  TCCAGAGGTCCAGCGGCCCTCCAGACATTGTTCCGGCGTGCATGCAATGACCCTGTTTCA  4542

Seq_1  4619  TGAAATATACATACGTGTATAACTGACGCAGCTAAACTACATATTATCTCATATTCCTTG  4678
             |||||||||||||||||||||||| |||||||||||||||||||||||||||||||||
Seq_2  4543  TGAAATATACATACGTGTATAACTAACGCAGCTAAACTACATATTATCTCATATTCCT--  4600

Seq_1  4679  TTGAGAAATAGCAATGCATACTTGAGAATATTCTGGACTACTCTGGATAATTGGAGAGTG  4738

Seq_2  4601  ------------------------------------------------------------  4600

Seq_1  4739  TTGGGAATTGGTTATGCATATTTGAGAAGATTCCAGAATAGTTTGGATTAGTGGAGGAGA  4798

Seq_2  4601  ------------------------------------------------------------  4600

Seq_1  4799  AGATATTTACAAGGAGCTTTCCAGTGAATATGAGAAGATATGAATATTCTAGAAGCCTTG  4858

Seq_2  4601  ------------------------------------------------------------  4600

Seq_1  4859  AGAGGTTACTTGAAGAGTGTTGCAATTTACCATGAAAGGACATAATTAAGATAAGTTTCA  4918

Seq_2  4601  ------------------------------------------------------------  4600

Seq_1  4919  AGAATAAGTGAGAATTCTCTAGAACTAGGATACATGTAGAGTATTCTAGAGAATTGTAGT  4978

Seq_2  4601  ------------------------------------------------------------  4600

Seq_1  4979  GGGATGTACTACATGGTCTTGTACAAGTATAAGTAGAGGTGCCCCCACCTCATAAGTGTA  5038

Seq_2  4601  ------------------------------------------------------------  4600

Seq_1  5039  CCACAATCACCACAAGTAGTGTATCACCACACCAAGTAGAGAAGTGAGCTACCATGGTAG  5098
                                  ||||
Seq_2  4601  --------------------CTATC-----------------------------------  4605

Seq_1  5099  GGTTGTGTGTAGGGTAGCCACTAAAGAGAGTGTTGTACCAACATTAAAATAGTGAATAAA  5158

Seq_2  4606  ------------------------------------------------------------  4605

Seq_1  5159  GTGTTGAGTTTCCACAAGTTTGATATCCCAAGTAGTGTAGTGTGCAAAGGTCCTCAAGAG  5218

Seq_2  4606  ------------------------------------------------------------  4605

Seq_1  5219  TGGGTGTTAGGGTCGCCGCCCGTAAAATCTCTACATTCCTCAACAAAAGATGTCTTAAGT  5278
                                                     ||||||||||||||||||||
Seq_2  4606  ----------------------------------------CAACAAAAGATGTCTTAAGT  4625

Seq_1  5279  TTGTCAAAATTTGGATGTATCTAGACATGATTTAGTGTATAGGTGCATTCAAATTTGGTC  5338
             ||||||||||||||||||||||||||||||||||||||||||||||||||||||||| ||
Seq_2  4626  TTGTCAAAATTTGGATGTATCTAGACATGATTTAGTGTATAGGTGCATTCAAATTTGATC  4685

Seq_1  5339  AAAGTTGAGATATCTTTTGTTGGACGGAGGGAGCACATGATTCACAAGGGACGATGTATA  5398
             |||||||||| || |||||||||||||| |||| ||||||||||||||||||||||||||
```

```
Seq_2  4686  AAAGTTGAGACATGTTTTGTTGGACGGAAGGAGTACATGATTCACAAGGGACGATGTATA  4745

Seq_1  5399  GTACCTGGCAGTAGTAATGTTGCAGCTAGCAAAGACGGATAAAACCAGTCAATCGTCCAA  5458
             |||||||| |||||||||||||||||||| | ||| ||||||||||||||||||||||||
Seq_2  4746  GTACCTGGTAGTAGTAATGTTGCAGCTAGTACAGAAGGATAAAACCAGTCAATCGTCCAA  4805

Seq_1  5459  GATAGGGGATGCAGTGATGAACTGGAAACAAGAAGGCAGACAGTAACTTGAGTCTGGCAA  5518
             ||||||||||||||||||||||||||||||||||||||||||||||||||||||||||||
Seq_2  4806  GATAGGGGATGCAGTGATGAACTGGAAACAAGAAGGCAGACAGTAACTTGAGTCTGGCAA  4865

Seq_1  5519  GGAAGAGGCAGGTAATAACTCATCATCTCATACTACACATGAGTATTTGCTGTGTAAGCA  5578
             ||||||||||||||||||||||||||||||||||||||||||||||||||||||||||||
Seq_2  4866  GGAAGAGGCAGGTAATAACTCATCATCTCATACTACACATGAGTATTTGCTGTGTAAGCA  4925

Seq_1  5579  TACAAGCTGCATTCAGGTATTCAACTGACCAGCAATAGTATATGTGCGCAATTTTTATGG  5638
             ||||||||||||||||||||||||||||||||||||||||||||||||||||||||||||
Seq_2  4926  TACAAGCTGCATTCAGGTATTCAACTGACCAGCAATAGTATATGTGCGCAATTTTTATGG  4985

Seq_1  5639  TTCCATCCAATAGATGACACTACAATTTCCAATTTCAGACCATGTAGACCAGCAAGAAAG  5698
             ||||||||||||||||||||||||||||||||||||||||||||||||||||||||||||
Seq_2  4986  TTCCATCCAATAGATGACACTACAATTTCCAATTTCAGACCATGTAGACCAGCAAGAAAG  5045

Seq_1  5699  CCAAGTCGGAAGCTTTTGGGACCCTTTTTAGCTAGTACAGAAGAAGACAAGGAAGCTATG  5758
             |||||||||||||||||||||||||||||||||||||||||||||||||||||||||| |
Seq_2  5046  CCAAGTCGGAAGCTTTTGGGACCCTTTTTAGCTAGTACAGAAGAAGACAAGGAAGCTACG  5105

Seq_1  5759  TGATCTATTGCAAATAGTTGCCAGCAGGTTCCAATTAATATACTCTCAGAAACTTCTCTT  5818
             ||||||||||||||||||||||||||||||||||||||||||||||||||||||||||||
Seq_2  5106  TGATCTATTGCAAATAGTTGCCAGCAGGTTCCAATTAATATACTCTCAGAAACTTCTCTT  5165

Seq_1  5819  TGTGTCCATGAGAACTATGGCATTACACAACTATAATATAGACACAGTACTAGGTTTACA  5878
             ||||||||||||||||||||||||||||||||||||||||||||||||||||||||||||
Seq_2  5166  TGTGTCCATGAGAACTATGGCATTACACAACTATAATATAGACACAGTACTAGGTTTACA  5225

Seq_1  5879  GTGATCCAA-GTTGCATCGCCAGGATGAGATTAAAGTAAATGCAAATTAGTAATGGGACT  5937
             ||||||||| |||||||||||||||||||||||||||||||||||||||||||||| |||
Seq_2  5226  GTGATCCAAAGTTGCATCGCCAGGATGAGATTAAAGTAAATGCAAATTAGTAATGGAACT  5285

Seq_1  5938  GACTATAGCTCAGTCGATTTCTCAGTCAAACAGGCAATCAATGGGTTTGTGTCCTCTAAT  5997
             ||||||||||||||||||||||||||||||||||||||||||||||||||||||||||||
Seq_2  5286  GACTATAGCTCAGTCGATTTCTCAGTCAAACAGGCAATCAATGGGTTTGTGTCCTCTAAT  5345

Seq_1  5998  TGGTACGGTGTTGGTTCTATTTATTTATTTTTGTCTCAAGCAATAGCAGGCTGGTAGCTT  6057
             ||||||||||||||||||||||||||||||||||||||||||||||||||||||||||||
Seq_2  5346  TGGTACGGTGTTGGTTCTATTTATTTATTTTTGTCTCAAGCAATAGCAGGCTGGTAGCTT  5405

Seq_1  6058  AGATTTTTCTTTTGGGTTTCTTTGTTGAAGGTCAATCTTGGCCTGAGCGTGTGGAGTTTC  6117
             |||||| |||||||||||||||||||||||||||||||||||||||||||||||||||||
Seq_2  5406  AGATTTGTCTTTTGGGTTTCTTTGTTGAAGGTCAATCTTGGCCTGAGCGTGTGGAGTTTC  5465

Seq_1  6118  AAATTACG------------------------CTCTAAAATA-----------AGTCCCC  6142
             ||||||||                        | | ||||||           |||||||
Seq_2  5466  AAATTACGTTCCACTATACCATTTTGTACATGCACGAAAATAAACATAAATCCAGTCCCC  5525

Seq_1  6143  TTTGGTGTGGGGCTTTCGTACACTA--ATAATTTTTGTACGGTGCTAAAAAAAACTTGAA  6200
             |||||||||||||||||||||||||  ||  |||||||||||||||||||||||||||||
```

```
Seq_2  5526  TTTGGTGTGGGGCTTTCGTACACTAGTAT--TTTTTGTACGGTGCTAAAAAAAACTTGAA  5583

Seq_1  6201  TTTTTTTTCAA-TTTTTTTTGACAAATTTGAAGAAGGTTAT-------------------  6240
             |||| |||||| ||||||||||||||||||||||||||||||
Seq_2  5584  TTTTATTTCAATTTTTTTTTGACAAATTTGAAGAAGGTTATTTGAACGCCTTTATTCAAG  5643

Seq_1  6241  ------------------------------------------------------------  6240

Seq_2  5644  TATATAAAGAAGAAAGCATGAAAAAAGTATTTTAATAATGACAGCACATTTGATCAAATC  5703

Seq_1  6241  ------------------------------------------------------------  6240

Seq_2  5704  AAGTTGCTCAAAATAGTAAGTGCTAGCCTGAGTGCTTTGCAACGTGCAAACCTAAGTTGC  5763

Seq_1  6241  ------------------------------------------------------------  6240

Seq_2  5764  GGTTATAAAAACATCAAGTTTAAATTTTTTAGCAAAGTTGGACTATCATGGTTGTTGGGT  5823

Seq_1  6241  ------------------------------------------------------------  6240

Seq_2  5824  CCACATGTACATACGAGGATAGGCCAAGTGCCTGCAAATTTTTATTACGTAATACTCTCT  5883

Seq_1  6241  ------------------------------------------------------------  6240

Seq_2  5884  CCGTTTCATAATTTTTGTCGAAATATTACATGTATCTAGACACGTTTTCTAAGAATAGAT  5943

Seq_1  6241  ------------------------------------------------------------  6240

Seq_2  5944  ACATTCATTTTAGAGCAAATTTGAGACAAGAATTATGAAACATTTTTGTCAGGGAGTAGG  6003

Seq_1  6241  ------------------------------------------------------------  6240

Seq_2  6004  TCTCCTTCCTCCCTAGGCCCGACTACTCTCTACAAGAAGCTTATCCTAGGGATGGATTTA  6063

Seq_1  6241  ------------------------------------------------------------  6240

Seq_2  6064  GCTATAGCTAGGCCAAGAATACCAACTCTAATTGCAGATCAATCAGTAAACGTGCATTGG  6123

Seq_1  6241  ------------------------------------------------------------  6240

Seq_2  6124  CCTACAGCCCGGCTGGCCGGCCGTACGCTGTAAGCCTGTAATACTGATACATATCATACG  6183

Seq_1  6241  ------------------------------------------------------------  6240

Seq_2  6184  GATCAGAATACATACCTTGCCGGCTACACGATGGATGGTTGGCCAAGCCGTATACCCTCG  6243

Seq_1  6241  ------------------------------------------------------------  6240

Seq_2  6244  TGACATCGATCAGTCTAGCTATCTTACTAGCTTGCTTGCTGCTCATGCAGGACGCCATTG  6303

Seq_1  6241  ------------------------------------------------------------  6240

Seq_2  6304  CGGCCGGGTCAATATATATAAAGCTTGTATAGCTGCAGTTGACTTCGTCCCGTCAACGCC  6363

Seq_1  6241  ------------------------------------------------------------  6240
```

```
Seq_2  6364  TCCAGCTTTTATTTTGTTTTCTTCTCCTTACTAGTTGGTAATATGTTTTCAATAAAATTA  6423

Seq_1  6241  ------------------------------------------------------------  6240

Seq_2  6424  GTTTTTACGAGTGCCGCACACTTTTTAGTGGACCTAGGAATTACTGGCGAGCCGGCAGGC  6483

Seq_1  6241  ------------------------------------------------------------  6240

Seq_2  6484  CGCTCCCGAATTGCGAGCGACCAACGATTTGCGGCGGCCATCGTCGTGATTTGCGGCGGC  6543

Seq_1  6241  -----------------------------------------------  6240

Seq_2  6544  CGGCAACTGCAACTGGGGCACACAAGTTGTACGACGAGATGGGTTTG  6590
```

BdindelWSU_18, downstream

>Bradi5g10600

AGCGAGTAGTCCAGCGAGTATGATGTTCCTACCTCCAGGTGGGATGGGATCTGTGTTCAGGGATGCTTTGGGGCTCGTGTTAAGTGA
GGAATTAGATCAGGTCTCAGTAATTTCATTATGGTTGGGTACAATTCTTCCAGGTGTATGGTTTATAGTAGTGTACTAATTAATCAGAT
TCTGTTATGAACTTATGATGCTGGCCTGCCTAGCAGTTTCCGGCGAAAGTCAAATTCAGACTGTTGTTCTGGGACAGTGGACTTTCCA
TTGGGTATTCTTGTTATGACTTGGTGTTAGGAACAGCTTTATGTCTAGCTGAAGTTGCCACTGGGTTATGGGCTGAGCTCTCAGTGAG
ATGTCCACTGTTATTACGTGTATAACTAACCAGTTTGTGATACTCGTAAGATTTTGGAAGACGATACAACTGAGCCGAGGTTCAGTGT
GGCGGCTTGGTGAAAATTGCAGTATAAATTCTTGCTTTCTGGCTCCTTGGCATGTTTTGGATTTTTAGTTTTGCACTGTGCAAATCCTTT
GATGTTTTTTCAGCGTGGGTCGGGGCAAATCACTCTCTTAGCATTTTCTGCACTTGTATATCAATGAATGGCTAGGCATATTCACAGTG
CGTTACAGAAGCTAGGAGGCTGTTAGTACTAACCCCTCAGTTTTAAGTGACAGAAATACGTTAGCGGCGCATTGCCCAAAGTGACAG
AAGTTAGCGGCGCATTGCCCATTTGCCCGACATCGCAAGCCTGCTGGACATGATGACTGAGGCCACAGCTATGGATGGAAGAAGGG
GGGTTGATCCGTGGGGTGTTTGCCCCTCCTTGGGCTTGCACGTGGGCTGACACATGACACATGACTCTTCTTATAATCAAAAACATAG
GTGAACATGTAGCAACGCCATCTCGATTGCAAGATAAAAATGACCTATTAGCTTCTTACCTTTCGATCATCTCCCTAATATAGAAATGT
AACTGCGTGTATTGCGCAAAAAAAAGGGTTGAATTTAATGTAAGTGTGTATTAATGTGACTGTTTTTAGGAGTATATGGGTATCGACT
TGTAGCTCCACATGTCAGCCTATTAAAGTCTCAACCATTATTCCCTCACACTGCAGTCTCAACTCTCAAGTCGTCTTCTCCGCAAGGAA
AGTCTTGTACTACTTTGGGCTTGCGTGTCACTGTCAGTGACCACCGAACCGACCCAACCTAGAAGAGACCGGTGCTAGGATTCTTTTT
TTGTCGGATTCAGGTGCGGCTGCGAAATCCTCACCGGCTGTAGAACCGCCGCCGGCCATGGACCCTGAAGCGGTGCGGCGGACCAT
CGAGCCCACCGCCTTCCCCGCGGACATCACGGGCTCCACCCGCTACGACGCCCTCGTCGTGAGCGGCGTCCGCCTCGACGCCGCCGA
GCACGGCCGCGTCCTCTTCTCCTTCGTCGTCACCCCCCGCCTCGCCGTAAGACACGCGCTCCCACCCCGCAGAAACCTTAAGCTCAAG
CTTCCTTACGCGATTTTGATTTCGAGACGGTGACGGACGGCGACGCTTGGTTCGAATTGGCGGTGGTTTTGCAGAGCCCCCAAGGAT
ATCTCCTCAGCGGCGTCACGGCAACGCTCGCGGACCAATTGGGGTCAGCCGCGTTCTACAGCAGTGGGGTCGGTTTGATCGGGGTC
AGCAGCGGTGTCGGGTTGAGCGGGGTCTCTCTCGAGATCAACGTGTCCTATGTTGACACAGCCACCGTCGGCGTGAGACCACTATCT
CTCTATATATTTGCACTTAGAAACGCTTTACTGTTTGGTCCTGCTTGTCATTGACTACTTTGGTTTATGCGGATTGGGAAATTCTGTGAT
GGAAATGCAATTCTGTCGAACTTGGCTGTGTATTATTTCGTGGTAGAATTGGGCAATTGACAAATTAGGCCTAGCTATGTGGCTCCTT
CCTCCAGGTGGGGTTTGTGTTCAAGAATGCTTTAGGGATCATGTTGAGCGAGCGAGGAAGTAGATCGGGTCTCGTTAATTGCACTGT
GGTTGGTTAAAATTAATGCAGGTGTCTGGTTTCCCTGTCTTGCACTCTTGCAGTTTCAGGTGAACCTCTGGATCTTGCAGTTAGGTATG
CTTGTCATGACTTGGTGTTATGAACAGCTTTATGTGTAGCTGAAGTTGACACTGAGTTGTGGGCTGGGCTCTTAGTGCTCAGAGGTAC
CTCCACTGTTATCACGCGTACAACTAGCCAGTTTGTAATAGTAAGAAGTTGTTGGAAGACGACAGGAGTGAACTGGGGTTAGGTGTT
GCAATCACTTTAAACTTGAAAGTTGATTAGAAATTCTTGTTTCCTGGCTTGACATAGCTAGGATTTGTACCCGGAGCAGAAACAACAT
TGGTTTTAACTGTTATGGGATAACAACCGTAGAACGTTTTATAGTGCAAATGCTTTGACATTTTTAGGTGTGGGAAAGCGTTCTTTCA
GAACTTCCGATGCTTCTCCATGAATGATTAGGCATACTCAATTAATCAAAACGGGTGACAGAAGCTAGGAAGCTCTAAATATTTATGC
ATCAGTTTTCTGTAAGGATGCCATGGCTATTTAAAGTTTTGGCCTTTATATACTTTTAGAAGATCTCATCTTCTCATGCTATGTTGATGT

AGTTTCTGTTTTCTCGGTGGGTTCTTCTCATCTTGGATTTTGGTTATGTTTCTCATCATTCTTCATCATGTGACAATTTTTGTTGATATTTT
GATACCTTATAAACAGGGTACTCATAATTGTATTCTTTTCTCCATTTATATTCACTTTACTTGTTTTGAGACGCGTTGAGTGATGTTCAT
ATTGTATGCCAAAGTACATAATTTGATGAAAATAATTTCAGGATTGGCTTGTTATTGAGCTTTCCTTGCTCAAGTATAATACTTACGCTT
ACTGTCAGTTCAGTGTCATTAGGGGTTAAATACTTACAGAGGTGGAAGTGAGATTCATAAAAGGATACTGGGGAAATAATGTGACGT
GGAATAGATAAATATTCATTAGTTAAGAATATCGTAAAGCTTGAATGCATGCATATTGATAGTCTTCTACTCCCTCCGTTTAACAAAAG
ATGTCTCAAGTTTGTCAAAATTTGGATGTATCTAGACATGACTTAGTGTATAGATGCATTCAAATTTAGTCAAAGTTGAGACATCCTTT
GTTGGACGGAGGAAGTAAAAAGATTCAAGTTGGTATTCTCATAAGTTAACATTGGTGAACATGGAAGAATGGCATCTGAATAGTTAA
AATAAAAGGACCACTATTTCTTGGTTCTTAGCTTCTTACCTTTCTGTCATTTTTCTATTATAGAAATATAAGTGTGTATTAAGAAGAAAA
GATGTTTGAATTTAGTGTGTGTGCATTCATTTTGCTTCCTTTGATGCCCTAAGTTCAGCTTGAGCTTGCTGGCTACAACATGGTTCCTA
GACAGAGTATAGGATCAGAATTGCTGTCTTCGTGTCCATACATTGCTGTGAAGAATCTAAATGTCTTTTCATGGTTGCTTTGATGGAAT
TTTAACCAGAAAAAAAGCAGCTCTAGGCAATTTCTTGCTAGAGTTGTATGAGAAAGCTTCACTAACTTGAGCCTATCTTTGTATTTTTC
TGGTATTTTAATTTTAAAAGTTATTTGCCATGATTTAAACAATTTATTTAATTTCCAGGAAGAAATAGAGGTTGAGGCAAAGCTGTTGC
GTGCTGGAAAATCAGTCGGTGTTGTCTCTGTTGATTTCAGGAAGAAAAGGACCGGGAAATTGATGGCTCAGGCCCGTCACACAAAGT
ACCTTGCTCTATCTAGCAAATTGTGAGACTCATCTTCTTTTGCAAGATAACACATGGATCAGCCCAACTCTTCGTTAGTGTTCTACATTG
GCAACTTCAGCCACACAAATTACCTTTTGCCTTCATCAATAGGCAAGAAAGAATGACAAGATGTTAATGTTTTACATTGTTAATGAAAA
AAGACCAGCTGTCCTATATCAAATTCATGTATCAATGGCTATTTTGTGATTAGTAATTTAGTATAGTTCTGCTCATTTAATCATTTTATTT
TCGGGTTTCAGCCGTTGCCAAATAATGTTTTGACAATTGCAAGGCACAATTCCTGTTTGGAGATCACGAACTTCACAGGAAAACGTAG
TGATTCCTCCTGAATTCCAATAAAGGCCCTTAGGATAAGTGGCCGCTGGAATTGAACAGTACTTAGATGTTACTGTACTGGTGCTAGA
TCTTCAGTTGATTGGCAACTGTATTCTGCAGTCATCTTGGGGGAGATCTTCTCTGCTGAAACGAGTGGATCATTGTTGTATTATTGGTT
TAGTGTCTTTAGATAGTTGGTGAAAACAGGCCCAGCTGCAATCATGCTCGCGTATGAGTGTACAGACGATGTGTTCCTCTGCGCTGTC
CCTGAGTGTCTCTAGCTGTTTGGCTGACTTCAGTAGGGAAACAAAAACAAAACTGTCCAACTCTGAACTTTCTTCCTGTGCTCATTTGG
CTGTGACGCAGGTAGGTGCTAAACAAGACAAAAATCACACATCAAATGTCCATTTTGGCATTCTGAATTTCTTTCTGCCAAAAGTGAC
AGCCCCAGACCAGAATCAAAAGTAAACTTTACTTACATTTACATCAATTGGTTCATAGAATCACAATGCCACTATGTTTACGGTGCGG
GGTAATCTACCCGAGTCACCGAATTGGAAGAATCACAATTCACAAAGATGTTCTTGTGTGAGTGTTGATGACACACAGCTGGCTTGTA
GAGAGGAGGAGACGCCTATACTGTTTCTACTAATGAGGGAGGCGGTTGGATGTCCCTGTCCTGGCTCTCCTTTCCCCTTTATTAGTGC
GAGCTAACCTTGTGTCGCCATGCGAGTGAGCCAGCCCGATAAAATCACAAAACACGCACGCGTCCTCCGATATTTTTCTCCCACTAAA
ACACAGCACAGCACACGCTTTATTTTTACCCCCACAAACGCACACGCAAAAGGCGTCCCAGAAAGATGCCGTACACCTTTCTGTCTTT
GAGATCTAGAGATGATATCATGCATGCATCATTACTTAGCCATGACACTTGACACAAACAACATCTCGTGACCAATGGACACGATCGA
ATTTTGTACGGGTCTTGATTCTTTATTTACTTATTCACACCTTTACTAAATCCCTTGCTAAATAATTATGAAAACAAGTGACAATGAAGA
AGATCGATGGAGAGAAAGAAAAAATAAATATGGAATGATGGTATGCTGCATTTCTCTGTACTCTAATTACATACACCCATGAATAACA
CCACTTAAATTACTCGTATGCGACATCTAGTATAATTTACTTGGCATTCGTTGAAGCATGACACTTGGACTTGCTGGCGATCGATGATG
ACCCGGTCAGGAATAAATTCACCGTCCAGCCGCGTTTGGTTGGCGGTGGCGAGCGGAGGCGGAGGAGGAGAAGGTCTTCTCGACG
ACGAAGAGCCTCCTGAAGATCCTCATGAGCAGCACCCAGCTGCTGGCCCGGCGGTAGTACTGCCCCGTCCCGCCGGCCCTCGACGAC
GCCTGCAGCATCGGCATCGGCGCGCACCCCCTTCCCCGCTCCTCCGACTCCGTGTCCGACTCCACCTCGCACGCCGCCGCCTTCTGCTT
GGAGCAACGGCGCAGCGCCTCCAGCTCCTCGTTGAAGTTCTCCCACAGCATGTCCATCTTCTCCTGATCCCCTTCCACGCCTCCGCGGA
CGAAGCCCCTTTTAATGTCATCCAGATCTTCTCCTCCGGCTCCTCTTTCCCCCTCGCCCTCCTCGGCGTCAGCCCAAGCCGGCTCTTGTT
CTTCTTCTTCTTCCATGTCGTCTTCAGTTCGGCTGGTCATCAGCGGCGGTGGCGCGACGGCGACGGCGGCGTCGACGGGGAACCAGA
GCGGGGAATTGGCGAAGTGTGGGAAGGGAAGGAGGCTGTGGTGGCGGTGGTGATGTAGGCACAGCGTGTCCGCGGGGGACGAC
GACGACTCTGCCGGGGCAGCGGTTGCGGGGAAGGTGAAGTCGTCCATTATCTGCCGCGGCAGCTCTCTCTATACCATACTCTGTTTCT
AGCTCCGATCAATAATACTCCTGGCTGTTGCTGACAAGGATACGTTGCCAGTCGGGTTAATTGGCTTCTGCCTGCCGCTCGCTCTCTC
GCTATAGCTGCTAACTGGCCCGGGTCATTATATACTTGGAGCGTGGTAGCAGGGCACGGGAAGGCCGGGGGTAGATCGGCGCGGG
GATCTATCTATCTACCGACAGAAGGCAAGGAGCCAAGGACGGCGGTACGTGACGGCCGTTGGAAGCGTACAAACTAAGCTTAAGAA
ACGTACGTACAACGTACGTACGTGCATTTGGCGTGTATTGGATGAAGTCAGCATGTGACTGTTTTGTGGCTCGTCTCGCCTGAATTAA
GGGCGAGCCCTGTTTTTTGCCGGGGCCAGTGAGCGAGCGATCGACCGGATCCGACCAACGCTGTACGGAGTAGGTGGCCAGCCGG
AAGCGCGGTTTACATGGATATACAATGGTGATTTTGGTCGATCGATCGGCCTCCGGCGGGTCGTTTTCGGCCGTCAAGTGGGGCGCG
CCACCATATATGGCACCGCATCTCGCACTTGGAGATATGGCATGTTTAGTTTTTTTATTTTATAATCACCTGTAATTTTATTCAAACTCG
ACGTTCATCACACCTACATTTGGATAAAACAATCCGCAAAACTACAAACTAACTCTATAAGTAAGAATTACATTCAAGTCTTTCAAAGT
AATCTTTTTCATGATATCAATCTTCGCAACACAAAGTACCGCCAAGACTTCAGTAAAAATGTGGCATGTTTAGTTCTGCGGGGCCATC
GGGAGGTAATCTTAACTAGAGAGGTAGGCCGGCCGGGCCGGCCCAAAACTGTAAATAGTACTCCGATTAATACCTTCAAAATATTCA

CATCCAACGAGAGCGCTGCCCTAGCCGTCATCGGTTGAAGATTTAGCGGCCCCCTCGCCTCTTGCCGCCGTCTTGGCGTTGCGAGGTA
GGGGGAACCTCGGTTTCTCCTAAGAGTATCCTGAAGTGTTTGCTTGTTTCAATTGTTTGGTTCTAATCGGCAACGCAATGGCGGAGGT
TGCAATACCGTCGCGAATAAATATACTATGCTTTTCCTCTGCTGTGGTGGTGCCTCAACGGGCTGTTGCTGCGGAGTTAGAGTTTTTA
GTCCCCGTAAGTTTAGTTACCCGATCTTGCGGATCTCGAGTTTCTATACTCGCAGATCCGTTTAAATATATCTGCTGAGTTTTTGTCAGT
GTGTCATGATGCTTTTGTTGGTTTTGTCTTTTACTATAATTTTGGAATCCTTCTCTGGCGATTCGACGACCTGCACCTTTAGGGGATCAT
TCTCGGTCCAAATACGTTCAATGCTTACGGCTTCTCACCTTTTTGGATGAGCGACTCAAGGGCATTTTCAAAACCTTTAAAGGTAATCA
AGTTCGTGTAGGGATACTAGTGACGATGTAACTGCTCCAGTCTGATTGCAATCTCTATGTTGAGGCCTCAACAGTATTTCATGCTGCA
AATGATGATATCATGGAGAAGATGTATTTAAGAGATTTCATTATAATTCTTAATTATAAGAGTTACCTTGTAGTTTTTAGAACCTATGA
TCGAGAGCATTGTACATGCACTAGTTCTCGCGTTTGAATAAAATTACAGTTATTCTAAAAAAAAAGCTAATATATACATATCTAAAAAA
TACGGACCGTTCCCTTCACCATCGTCTGTCAAACTTTCCATAAAATTTGTGGGCCCACTAGGGTTGCATGTATATTAGGCTTACTAAGC
AGTTGGCCAAATTGGACTAGAACTCCACCAGATCAGTCCAGATTCAATCAATCACGAATACAACACAACTTCAAACTTAACGTATCAA
TCACGATCGGTCCAATACAGCCTCCCATAACGAAGCATGTATCAATATCGGTATGGAGTACGGATCGTATGAAAAAAAGTCATAAAG
CAATCTCAGGAAAATCAATCCAATCCGAAATAGAAAAACTGATACATCCCTCTCTCGCAGCGAAGCTCCACGCCATGGCCCATGAATC
GTTACGATAGTCGGGATAGGATCGATCAATGTACTTGCTCGTAAATTATGCCTACGACCTGCAAGCGTCCCTAAAAAAATTATCACTC
CCTCTGATCAATATTAATTGTGTCAAATTTGCCCAAATATGGATGTATTTATGCCTAAAAAGCGTCTAGATACATGTAATATTTCGGAG
GGAGTACGTCTACGGATGGACAGAAACTACAAGTCCGAACGCATGGCACAAGCCGCTGCACAACGCAATCACCCATGAAGATGCAA
ATCTGCACAGGCGCACAATGACAGTTCAACTGTCGGCAGATCGGCCTATACGAGGGATTCTACACTAGACTCTAGACATCGCTGACG
CCTTGAAGCCAAAATTTATTTAGGTGGATCTATTTGTAAGCTAGCCTTTTGTGATAGGTTTAATTAGCCCTAATTGTTGGTTAAAGTTTT
GAGATTGTCTTATCGACTAAGAAAAACGCATCCGAGAAAAGAAAAAAAAGAGAAAGCGAGTAGATGACTTGATAAAATCACAGAGA
GGATAAATTTATAAAATTTTATGTGTTGTCACGGGTTTTTGACATATTTTATACATACATATTTGGATATTATTTAGATGTTTATATTAG
GGGCCCGGTTTTTTAAGTTCGCCCGAGGCCCCCGAAATCACAGGAGCGGCCCTGGCCGGATGCCACCCAGTTACCTACCATATCATT
GGCAGCCGCCTTCATTGCTCCCATCAACACAACGCCAGGGCCACCGCCGCATCCACCCTCCAATCCGTGACCACCAGGTCCACCACCA
CGCTGCTGCCTATCATTGTTTCTCCTCACAGGTGAGCCCCAGGATCGTTCCCTCCTCTTATCTGAGCAATCCGGCCATCGCCTCACCTC
GAGCCGCCTTGCTCCCTTCTGTTTCCGCGCGAGGGGTTCCGTCGAATCAGGCCGCCCTGGTGTGTCAGTACTTTGCCGGAAAGTGTGC
ACTTGCTAGCTAAAAGCTCCATCCATTAATTCAGAGAGATACATCGTTTGTGGATTGAATTTAGCTAGCTACTAAATACCATGTTTTAA
TTTTCTCAGTTGATCGGTGTATATACATTGCTTATGCTGCTATAGGTTTTTTTTATGGAATCTTGCAATGTGAGAGAAGGCTTATGGC
CCGTATGGAAAGTCGGAAACGCAGCAACAAAGATTTTTGTGATAGTGGTCCTCTGTCTTGCGAGCTTCTGGTTTCCGGAGGTCTTCAA
CCGAGGGGTCGTCTCACTCACATGCATAATCAGGAAGAGGAACAGCATGACCGGGCTGTCATTCACCTACTAGACGGCATCTACCTT
CTGTGGACAAGCTCAGTAGGCCGTGGCCGTGGCAGCGCACGGGCATTTCGGCTAGTACCTTAAAAAAAACTAAAAAACTGATACGCC
AATTTCGCTGCATGACGTTCTTCTTGAGAGGAGATGATCCCATGTCCATTGGGCATGCTGTTGCTCAAACCATGGCAACCCAGAAGGT
TGAGCTAGGTGAATTCACGATTTACACGGAGAAGGAGGACACAGTTTGCTGCGCTGATGATCTGATCCACTACGGGAGACAATTCAT
GTTGTTCTTTGATGCCTGTCCAATTGCATTCGGTGGTCTCACACGTCTTGACTTAGAGAATTTGAGATTTGGTTAATCTGACATCCGTA
ACGTCCTCAACACTTGCAAACGGTTAAAGCATCTACGCCTGTTCAATTGTGACTCTGGCAGTAGTCAGACCGTGCTGCAAGTTGAACA
CTCACAACTTTGTGAGCTTGCTATTGTTGATTGTTCTATTGAAAGAGTCGAGCTCACCTCACTTCCCAAACTCACACGGTTCATATTTGA
GTGTTGGATCGCTTTCGAAGATCCCCTATCTTTTGGTTATGTCCCGTTGCTCGAGGCTGTAAGCCTCGCCAATATTTGTCTTAGTTGGC
ACCAGATGGTCAAGTTAAGTAAGTTTCTTTGGGTACCTCTGTGCGAGATTTGAAGTTGGGATTTGATTTCGAAAGGGTAAGTAAAGA
ATATGGTTTTGCTAGCTCTGGTGACAACTTCTTGTCTGCTCATCATCATGTGTTTACTTGTTGCAGATTTGGGTTCAACCAGAATGTCTG
ACGGTAAGGCTGGCATCTGTGTTCAACCAAATAAGGTTTGTGAATCTAGCTGATATTCCTGAAGGTTATGATCTCACCTGGACATTAT
TCTTTCTTAAA

>BdiBd21-3.5G0131800

GACTCATCTTCTTTTGCAAGATAACACATGGATCAGCCCAACTCTTCGTTAGTGTTCTACATTGGCAACTTCAGCCACACAAATTACCTT
TTGCCTTCATCAATAGGCAAGAAAGAATGACAAGATGTTAATGTTTTACATTGTTAATGAAAAAAGACCAGCTGTCCTATATCAAATT
CATGTATCAATGGCTATTTTGTGATTAGTAATTTAGTATAGTTCTGCTCATTTAATCATTTTATTTTCGGGTTTCAGCCGTTGCCAAATA
ATGTTTTGACAATCGCAAGGCACAATTCCTGTTTGGAGATCACGAACTTCACAGGAAAACGTAGTGATTCCTCCTGAATTCCAATAAA
GGCCCTTAGGATAAGTGGCCGCTGGAATTGAACAGTACTTAGATGTTACTGTACCGGTGCTAGATCTTCAGTTGATTGGCAACTGTAT
TCTGCAGTCATCTTGGGGGAGATCTTCTCTGCTGAAACGAGTGGATCATTGTTGTATTATTGGTTTAGTGTCTTTAGATAGTTGGTGA
AAACAGGCCCAGCTGCAATCATGCTCGCGTATGAGTGTACAGACGATGTGTTCCTCTGCGCTGTCCCTGAGTGTCTCTAGCTGTTTGG
CTGACTTCAGTAGGGAAACAAAAACAAAACTGTCCAACTCTGAACTTTCTTCCTGTGCTCATTTGGCTGTGACGCAGGTAGGTGCTAA

ACAAGACAAAAATCACACATCAAATGTCCATTTTGGCATTCTGAATTTCTTTCTGCCAAAAGTGACAGCCCCAGACCAGAATCAAAAG
TAAACTTTACTTGCATTTACATCAATTGGTTCATAGAATCACAATGCCACTATGTTTACGGTGCGGGGTAATCTACCCGAGTCACCGAA
TTGGAAGAATCACAATTCACAAAGATGTTCTTGTGTGAGTGTTGATGACACACAGCTGGCTTGTAGAGAGGAGGAGACGCCTATACT
GTTTCTACTAATGAGGGAGGCGGTTGGATGTCCCTGTCCTGGCTCTCCTTTCCCCTTTATTAGTGCGAGCTAACCTTGTGTCGCCATGC
GAGTGAGCCAGCCCGATAAAATCACAAAACACGCACGCGTCCTCCGATATTTTTCTCCCACTAAAACACAGCACAGCACACGCTTTAT
TTTTACCCCCACAAACGCACACGCAAAAGGCGTCCCAGAAAGATGCCGTACACCTTTCTGTCTTTGAGATCTAGAGATGATATCATGC
ATGCATCATTACTTAGCCATGACACTTGACACAAACAACATCTCGTGACCAATGGACACGATCGAATTTTGTACGGGTCTTGATTCTTT
ATTTACTTATTCACACCTTTACTAAATCCCTTGCTAAATAATTATGAAAACAAGTGACAATGAAGAAGATCGATGGAGAGAAAGAAAA
AATAAATATGGAATGATGGTATGCTGCATTTCTCTGTACTCTAATTACATACACCCATGAATAACACCACTTAAATTACTCGTATGCGA
CATCTAGTATAATTTACTTGGCATTCGTTGAAGCATGACACTTGGACTTGCTGGCGATCGATGATGACCCGGTCAGGAATAAATTCAC
CGTCCAGCCGCGTTTGGTTGGCGGTGGCGAGCGGAGGCGGAGGAGGAGGCAGAGGAGGAGAAGGTCTTCTCGACGACGAAGAGC
CTCCTGAAGATCCTCATGAGCAGCACCCAGCTGCTGGCCCGGCGGTAGTACTGCCCCGTCCCGCCGGCCCTCGACGACGCCTGCAGC
ATCGGCATCGGCGCGCACCCCCTTCCCCGCTCCTCCGACTCCGTGTCCGACTCCACCTCGCACGCCGCCGCCTTCTGCTTGGAGCAAC
GGCGCAGCGCCTCCAGCTCCTCGTTGAAGTTCTCCCACAGCATGTCCATCTTCTCCTGATCCCCTTCCACGCCTCCGCGGACGAAGCCC
CTTTTAATGTCATCCAGAGCTTCTCCTCCGGCTCCTCTTTCCCCCTCGCCCCCCTCGGCGTCAGCCCAAGCCGGCTCTTGTTCTTCTTCTT
CTTCCATGTCGTCTTCAGTTCGGCTGGTCATCAGCGGCGGTGGCGCGACGGCGACGGCGGCGTCGACGGGGAACCAGAGCGGGGA
ATTGGCGAAGTGTGGGAAGGGAAGGAGGCTGTGGTGGCGGTGGTGATGTAGGCACAGCGTGTCCGCGGGGGACGACGACGACTC
TGCCGGGGCAGTGGTTGCGGGGAAGGTGAAGTCGTCCATTATCTGCCGCGGCAGCTCTCTCTATACCATACTCTGTTTCTAGCTCCGA
TCAATAATACTCCTGGCTGTTGCTGACAAGGATACGTTGCCAGTCGGGTTAACTGGCTTCTGCCTGCCGCTCGCTCTCTCGCTATAGCT
GCTAACTGGCCCGGGTCATTATATACTTGGAGCGTGGTAGCAGGGCACGGGAAGGCCGGGGGTAGATCGGCGCGGGGATCTATCT
ATCTACCGACAGAAGGCAAGGAGCCAAGGACGGCGGTACGTGACGGCCGTTGGAAGCGTACAAACTAAGCTTAAGAAACGTACGT
ACAACGTACGTACGTGCATTTGGCGTGTATTGGATGAAGTCAGCATGTGACTGTTTTGTGGCTCGTCTCGCCTGAATTAAGGGCGAG
CCCTGTTTTTTGCCGGGGCCAGTGAGCGAGCGATCGACCGGATCCGACCAACGCTGTACGGAGTAGGTGGCCAGCGGGGAGCGCG
GTTTACATGGATATACAATGGTGATTTTGGTCGATCGATCGGCCTCCGGCGCGGTCGTTTTCGGCCGTCAAGTGGGGCGCGCCACCA
TATATGGCACCGCATCTCGCACTTGGAGATATGGAATGTTTAGTTTTTTTATTTTATAATCACCCGTATTTTTATTCAAACTCGACGTTC
ATTACACGTACATTTGGATAAAACAATCCGCAAAACTACAAACTAACTCTATAAGTAAGAATTACATTCAAGTCTTTCAAAGTAATCTT
TTTCATGATATCAATCTTCGCAACACAAAGTACCGCCAAGGCTTCAGTAAAAATGTGGCATGTTTAGTTCTGCGGGGCCATCGGGAG
GTAATCTGAACTAGAGAGGTAGGCCGGCCGGGCCGGCCCAAAACTGTAAATAGTACTCCGGTTAATACCTTCAAAATATTCACATCC
AACGAGGCGCTGCCCTAGACGTCATCGGTTGAAGATTTAGCGGCCCCCTCGCCTCCGGCCGCCGTCTTGGCGTTGTGAGGTAGGGG
GAACCTCGGTTTCTCCCAAGAGTATTCTGAAGTGTTTGCTTATTTCAATTGTTTGGTTCTGATCGGCAACGCAATGGCGGAGGTTGCA
ATACCGTCGCGAATAAATATACTATACTTTTCCTCTGCTGTGGTGGTGGCTCAACGGGCTGTTGCTGCGGAGTTAGAGTTTTTAGTCC
CCGTAAGTTTAGTTACCCGATCTTGCGGATCTCGAGTTTCTATACTCGCAGATCCGTTTAAATATATCTGCTGAGTTTTTGTCAGTGTGT
CATGATGCTTTTGTTGGTTTTGTCTTTTACTATAATTTTGGAATCCTTCTCTGGCGATTCGACGACCTGCACCTTTAGGGGATCATTCTC
GGCCCAAATACGTTCAACGCTTACGGCTTCTCACCTTCTTGGATGAGCGACTCAAGGGCAGTTTCAAAACCTTTAAAGGTAATCAAGT
TCATGTAGGGATACTATTGACGATGTAACTGCTCCAGTCTTATTGCAATCTCTATGTTGAGGCCTCAACAGTATTTCATGCTGCAAATG
ATGATATCATGGAGAAGATGTATTTAAGAGATTTCATTATAATTCTTAATTATAAGAGTTACCTTGTAGTTTTTAGAACCTATGATCGA
GAGCATTGTACATGCACTAGTTCTCGCGTTTGAATAAAATTACCGTTATTCTAAAAAAAAAGCTAATATATACATATCTAAAAAATACG
GAGCGTTCCCTTTACCATCGTCTGTCAAACTTTCCATAAATTTGTGGGCCCACTAGGGTTGCATGTATATTAGGCTTACTAAGCGGTTG
GCCAAATTGGACTGGAACTCCACCAGATCAGTCCAGATTCAATCAATCACGAATACAACACAACTTCAAACTTAACGTATCAATCACG
ATCGGTCCAATACAGCCTCCCGTAACGAAGCATGTATCAATATCGGTATGGAGTATGGATCGTATGAAAAAAAGTCATAAAGCAATC
TCAGGAAAATCAATCCAATCCGAAACAGAAAAACTGATACATCCCTCTCTCGCAGCGAAGCTCCACGCCATGGCCCATGAATCGTTAT
GATAGTCGGGATAGGATCGATCAATGTACTTGTTCGTAAATTATGCCTAATTGTTGGTTAAAGTTTTGAGATTGTCTTATCGACTAAG
AAAAACGCATCCGAGAAAAGAAAAAAAAGAGAAAGCGAGTAGATGACTTGATAAAATCACACAGAGGATAAATTTATAAAATTTTA
TGTGTTGTGACGAGATTTTGACATATTTTATACATACATATTTGGATATTATTTAGATGCTTATATTAGGGGCCCGGTTTTTTAAATTCG
CCCGAGGCCCCCGAAATCACAGGAGCGGCCCTGGCCGGATGCCACCCAGTTACCTACCATATCATTGGCAGCCGCCTTCGTTGCTCCC
ATCAACACAACGCCAGGGCCACCGCCGCATCCACCCTCCAATCCGTGACCACCAGGTCCACCACCACGCTGCTGCCTATCATTGTTTCT
TCCTCACAGGTGAGCCCCAGGATCGTTCCCTCCTCTTATCTGAGCAATCCGGCCATCGCCTCACCTCGAGCCGCCTTGCTCCCTTCTGT
TTCCGCGCGAGGGGTTCCGTCGAATCAGGCCGCCCTGGTGTGTCAGTACTTTGCCGGAAAGAGTGCACTTGCTAGCTACTAGCTCCA
TCCATTAATTCAGAGAGATACATCGTTTGTGGATTGAATTTAGCTAGCTACTAAATACCATGTTTTAATTTTCTCAGTTGGTCAGTGTAT

ATACATTGCTTATGCTGCTATAGGTTTTTTTTTATGGAATCTTGCAATGTGAGAGAAGGCTTATGGCCCGTATGGAAAGTCGGAAACG
CAGCAACAAAGATTTTTGTGATAGTGGTCCTCTGTCTTGCGGGCTTCTGGTTTCTGGAGGTCTTCAACCGAGGGGTCGTCTCACTCGC
ATACATAATCAGGAAGAGAAACAGCATGACCGGGCTGTCATTCACCTACTAGACGGCATCTACCTTCTGTGGACAAGCTCAGTAGGC
CGTGGCCGTGGCAGCGCACGGGCATTTCGGCTAGTACCTTAAAAAAAACTAAAAAACTGATACGCCAATTTCGCTGCATGACGTTCTT
CTTGAGAGGAGATGATCCCATGTCCATTGGGCATGCTGTTGCTCAAACCATGGCAACCCAGAAGGTTGAGCTAGGTGAATTCACAAT
TTACACGGAGAAGGAGGACACAGTTTGCTGCGCTGATGATCTGATCCACTACGGGAGACAATTCATGTTGTTCTTTGATGCCTGTCCA
ATTGCATTCGGTGGTCTCACACGTCTTGACTTAGAGAATTTGAGATTTGGTGAATCTGACATCCGTAACGTCCTCAACACTTGCAAAC
GGTTAAAGCATCTACGCCTGTTCAATTGTGACTCTGGCAGTAGTCAGACCGTGCTGCAAGTTGAACACTCACAACTTTGTGAGCTTGC
TATTGTTGATTGTTCTATTGAAAGAGTCGAGCTCACCTCACTTCCCAAACTCACACGGTTCATATTTGAGTGTTGGATCGCTTTCGAAG
ATCCCCTATCTTTTGGTTATGTCCCGTTGCTCGAGGCTGTAAGCCTCGCCAATATTTGTCTTAGTTGGCACCAGATGGTCAAGTTAAGT
AAGTTTCTTTGGGTACCTCTGTGCGAGATTTGAAGTTGGGATTTGATTTCGAAAGGGTAAGTAAAGAATATGGTTTTGCTAGCTCTGG
TGACAACTTCTTGTCTGCTCATCATCATGTGTTTACTTGTTGCAGATTTGGGTTCAACCAGAATGTCTGACGGTAAGGCTGGCATCTGT
GTTCAACTAAATAAGGTTTGTGAATCTAGCTGATATTCCTGAAGGTTATGATCTCACCTGGACATTATTCTTTCTTAAAGCTGCACCGA
ACTTAAAGGAGCTATATATGACGGTATGTCCTTACCTAAAGCTTGCTTTTTCTTTCCATAGCATGTGCCCGTGCTCAATGTTGAATTTTG
CCAGTTTTTCATATTGAACATAAATCGCTGCAAACGAGTAACTGTGTTCTATTTAACTCTGTCTTCCTATGGCATCTAGGTTTGGGATCA
TCTTTGTGTAATGAAAACGAATGCAGCAGATAGGAGGGCACTCTTGTATAGCGAGAAGAAGGGTATAGAGTGGGAATTATCTCCAT
CTGATTTCCAACATCAGAGTCTGGCCACAGTCGCCATCTTTGGGTTTCAAGCTCAAGACTACATGATGTATTCTTGTATA

```
Alignment of Sequence_1:  [Untitled Sequence #1] with  Sequence_2: [Sequence Window #2]

Similarity : 6072/10327 (58.80 %)

Seq_1  1     AGCGAGTAGTCCAGCGAGTATGATGTTCCTACCTCCAGGTGGGATGGGATCTGTGTTCAG  60

Seq_2  1     ------------------------------------------------------------  0


Seq_1  61    GGATGCTTTGGGGCTCGTGTTAAGTGAGGAATTAGATCAGGTCTCAGTAATTTCATTATG  120

Seq_2  1     ------------------------------------------------------------  0


Seq_1  121   GTTGGGTACAATTCTTCCAGGTGTATGGTTTATAGTAGTGTACTAATTAATCAGATTCTG  180

Seq_2  1     ------------------------------------------------------------  0


Seq_1  181   TTATGAACTTATGATGCTGGCCTGCCTAGCAGTTTCCGGCGAAAGTCAAATTCAGACTGT  240

Seq_2  1     ------------------------------------------------------------  0


Seq_1  241   TGTTCTGGGACAGTGGACTTTCCATTGGGTATTCTTGTTATGACTTGGTGTTAGGAACAG  300

Seq_2  1     ------------------------------------------------------------  0


Seq_1  301   CTTTATGTCTAGCTGAAGTTGCCACTGGGTTATGGGCTGAGCTCTCAGTGAGATGTCCAC  360

Seq_2  1     ------------------------------------------------------------  0


Seq_1  361   TGTTATTACGTGTATAACTAACCAGTTTGTGATACTCGTAAGATTTTGGAAGACGATACA  420

Seq_2  1     ------------------------------------------------------------  0


Seq_1  421   ACTGAGCCGAGGTTCAGTGTGGCGGCTTGGTGAAAATTGCAGTATAAATTCTTGCTTTCT  480

Seq_2  1     ------------------------------------------------------------  0


Seq_1  481   GGCTCCTTGGCATGTTTTGGATTTTTAGTTTTGCACTGTGCAAATCCTTTGATGTTTTTT  540
```

```
Seq_2  1     ------------------------------------------------------------  0

Seq_1  541   CAGCGTGGGTCGGGGCAAATCACTCTCTTAGCATTTTCTGCACTTGTATATCAATGAATG  600

Seq_2  1     ------------------------------------------------------------  0

Seq_1  601   GCTAGGCATATTCACAGTGCGTTACAGAAGCTAGGAGGCTGTTAGTACTAACCCCTCAGT  660

Seq_2  1     ------------------------------------------------------------  0

Seq_1  661   TTTAAGTGACAGAAATACGTTAGCGGCGCATTGCCCAAAGTGACAGAAGTTAGCGGCGCA  720

Seq_2  1     ------------------------------------------------------------  0

Seq_1  721   TTGCCCATTTGCCCGACATCGCAAGCCTGCTGGACATGATGACTGAGGCCACAGCTATGG  780

Seq_2  1     ------------------------------------------------------------  0

Seq_1  781   ATGGAAGAAGGGGGGTTGATCCGTGGGGTGTTTGCCCCTCCTTGGGCTTGCACGTGGGCT  840

Seq_2  1     ------------------------------------------------------------  0

Seq_1  841   GACACATGACACATGACTCTTCTTATAATCAAAAACATAGGTGAACATGTAGCAACGCCA  900

Seq_2  1     ------------------------------------------------------------  0

Seq_1  901   TCTCGATTGCAAGATAAAAATGACCTATTAGCTTCTTACCTTTCGATCATCTCCCTAATA  960

Seq_2  1     ------------------------------------------------------------  0

Seq_1  961   TAGAAATGTAACTGCGTGTATTGCGCAAAAAAAAGGGTTGAATTTAATGTAAGTGTGTAT  1020

Seq_2  1     ------------------------------------------------------------  0

Seq_1  1021  TAATGTGACTGTTTTTAGGAGTATATGGGTATCGACTTGTAGCTCCACATGTCAGCCTAT  1080

Seq_2  1     ------------------------------------------------------------  0

Seq_1  1081  TAAAGTCTCAACCATTATTCCCTCACACTGCAGTCTCAACTCTCAAGTCGTCTTCTCCGC  1140

Seq_2  1     ------------------------------------------------------------  0

Seq_1  1141  AAGGAAAGTCTTGTACTACTTTGGGCTTGCGTGTCACTGTCAGTGACCACCGAACCGACC  1200

Seq_2  1     ------------------------------------------------------------  0

Seq_1  1201  CAACCTAGAAGAGACCGGTGCTAGGATTCTTTTTTTGTCGGATTCAGGTGCGGCTGCGAA  1260

Seq_2  1     ------------------------------------------------------------  0

Seq_1  1261  ATCCTCACCGGCTGTAGAACCGCCGCCGGCCATGGACCCTGAAGCGGTGCGGCGGACCAT  1320

Seq_2  1     ------------------------------------------------------------  0

Seq_1  1321  CGAGCCCACCGCCTTCCCCGCGGACATCACGGGCTCCACCCGCTACGACGCCCTCGTCGT  1380
```

```
Seq_2  1     ------------------------------------------------------------  0

Seq_1  1381  GAGCGGCGTCCGCCTCGACGCCGCCGAGCACGGCCGCGTCCTCTTCTCCTTCGTCGTCAC  1440

Seq_2  1     ------------------------------------------------------------  0

Seq_1  1441  CCCCCGCCTCGCCGTAAGACACGCGCTCCCACCCCGCAGAAACCTTAAGCTCAAGCTTCC  1500

Seq_2  1     ------------------------------------------------------------  0

Seq_1  1501  TTACGCGATTTTGATTTCGAGACGGTGACGGACGGCGACGCTTGGTTCGAATTGGCGGTG  1560

Seq_2  1     ------------------------------------------------------------  0

Seq_1  1561  GTTTTGCAGAGCCCCCAAGGATATCTCCTCAGCGGCGTCACGGCAACGCTCGCGGACCAA  1620

Seq_2  1     ------------------------------------------------------------  0

Seq_1  1621  TTGGGGTCAGCCGCGTTCTACAGCAGTGGGGTCGGTTTGATCGGGGTCAGCAGCGGTGTC  1680

Seq_2  1     ------------------------------------------------------------  0

Seq_1  1681  GGGTTGAGCGGGGTCTCTCTCGAGATCAACGTGTCCTATGTTGACACAGCCACCGTCGGC  1740

Seq_2  1     ------------------------------------------------------------  0

Seq_1  1741  GTGAGACCACTATCTCTCTATATATTTGCACTTAGAAACGCTTTACTGTTTGGTCCTGCT  1800

Seq_2  1     ------------------------------------------------------------  0

Seq_1  1801  TGTCATTGACTACTTTGGTTTATGCGGATTGGGAAATTCTGTGATGGAAATGCAATTCTG  1860

Seq_2  1     ------------------------------------------------------------  0

Seq_1  1861  TCGAACTTGGCTGTGTATTATTTCGTGGTAGAATTGGGCAATTGACAAATTAGGCCTAGC  1920

Seq_2  1     ------------------------------------------------------------  0

Seq_1  1921  TATGTGGCTCCTTCCTCCAGGTGGGGTTTGTGTTCAAGAATGCTTTAGGGATCATGTTGA  1980

Seq_2  1     ------------------------------------------------------------  0

Seq_1  1981  GCGAGCGAGGAAGTAGATCGGGTCTCGTTAATTGCACTGTGGTTGGTTAAAATTAATGCA  2040

Seq_2  1     ------------------------------------------------------------  0

Seq_1  2041  GGTGTCTGGTTTCCCTGTCTTGCACTCTTGCAGTTTCAGGTGAACCTCTGGATCTTGCAG  2100

Seq_2  1     ------------------------------------------------------------  0

Seq_1  2101  TTAGGTATGCTTGTCATGACTTGGTGTTATGAACAGCTTTATGTGTAGCTGAAGTTGACA  2160

Seq_2  1     ------------------------------------------------------------  0

Seq_1  2161  CTGAGTTGTGGGCTGGGCTCTTAGTGCTCAGAGGTACCTCCACTGTTATCACGCGTACAA  2220
```

```
Seq_2  1     ------------------------------------------------------------  0

Seq_1  2221  CTAGCCAGTTTGTAATAGTAAGAAGTTGTTGGAAGACGACAGGAGTGAACTGGGGTTAGG  2280

Seq_2  1     ------------------------------------------------------------  0

Seq_1  2281  TGTTGCAATCACTTTAAACTTGAAAGTTGATTAGAAATTCTTGTTTCCTGGCTTGACATA  2340

Seq_2  1     ------------------------------------------------------------  0

Seq_1  2341  GCTAGGATTTGTACCCGGAGCAGAAACAACATTGGTTTTAACTGTTATGGGATAACAACC  2400

Seq_2  1     ------------------------------------------------------------  0

Seq_1  2401  GTAGAACGTTTTATAGTGCAAATGCTTTGACATTTTTAGGTGTGGGAAAGCGTTCTTTCA  2460

Seq_2  1     ------------------------------------------------------------  0

Seq_1  2461  GAACTTCCGATGCTTCTCCATGAATGATTAGGCATACTCAATTAATCAAAACGGGTGACA  2520

Seq_2  1     ------------------------------------------------------------  0

Seq_1  2521  GAAGCTAGGAAGCTCTAAATATTTATGCATCAGTTTTCTGTAAGGATGCCATGGCTATTT  2580

Seq_2  1     ------------------------------------------------------------  0

Seq_1  2581  AAAGTTTTGGCCTTTATATACTTTTAGAAGATCTCATCTTCTCATGCTATGTTGATGTAG  2640

Seq_2  1     ------------------------------------------------------------  0

Seq_1  2641  TTTCTGTTTTCTCGGTGGGTTCTTCTCATCTTGGATTTTGGTTATGTTTCTCATCATTCT  2700

Seq_2  1     ------------------------------------------------------------  0

Seq_1  2701  TCATCATGTGACAATTTTTGTTGATATTTTGATACCTTATAAACAGGGTACTCATAATTG  2760

Seq_2  1     ------------------------------------------------------------  0

Seq_1  2761  TATTCTTTTCTCCATTTATATTCACTTTACTTGTTTTGAGACGCGTTGAGTGATGTTCAT  2820

Seq_2  1     ------------------------------------------------------------  0

Seq_1  2821  ATTGTATGCCAAAGTACATAATTTGATGAAAATAATTTCAGGATTGGCTTGTTATTGAGC  2880

Seq_2  1     ------------------------------------------------------------  0

Seq_1  2881  TTTCCTTGCTCAAGTATAATACTTACGCTTACTGTCAGTTCAGTGTCATTAGGGGTTAAA  2940

Seq_2  1     ------------------------------------------------------------  0

Seq_1  2941  TACTTACAGAGGTGGAAGTGAGATTCATAAAAGGATACTGGGGAAATAATGTGACGTGGA  3000

Seq_2  1     ------------------------------------------------------------  0

Seq_1  3001  ATAGATAAATATTCATTAGTTAAGAATATCGTAAAGCTTGAATGCATGCATATTGATAGT  3060
```

```
Seq_2  1     ------------------------------------------------------------  0

Seq_1  3061  CTTCTACTCCCTCCGTTTAACAAAAGATGTCTCAAGTTTGTCAAAATTTGGATGTATCTA  3120

Seq_2  1     ------------------------------------------------------------  0

Seq_1  3121  GACATGACTTAGTGTATAGATGCATTCAAATTTAGTCAAAGTTGAGACATCCTTTGTTGG  3180

Seq_2  1     ------------------------------------------------------------  0

Seq_1  3181  ACGGAGGAAGTAAAAAGATTCAAGTTGGTATTCTCATAAGTTAACATTGGTGAACATGGA  3240

Seq_2  1     ------------------------------------------------------------  0

Seq_1  3241  AGAATGGCATCTGAATAGTTAAAATAAAAGGACCACTATTTCTTGGTTCTTAGCTTCTTA  3300

Seq_2  1     ------------------------------------------------------------  0

Seq_1  3301  CCTTTCTGTCATTTTTCTATTATAGAAATATAAGTGTGTATTAAGAAGAAAAGATGTTTG  3360

Seq_2  1     ------------------------------------------------------------  0

Seq_1  3361  AATTTAGTGTGTGTGCATTCATTTTGCTTCCTTTGATGCCCTAAGTTCAGCTTGAGCTTG  3420

Seq_2  1     ------------------------------------------------------------  0

Seq_1  3421  CTGGCTACAACATGGTTCCTAGACAGAGTATAGGATCAGAATTGCTGTCTTCGTGTCCAT  3480

Seq_2  1     ------------------------------------------------------------  0

Seq_1  3481  ACATTGCTGTGAAGAATCTAAATGTCTTTTCATGGTTGCTTTGATGGAATTTTAACCAGA  3540

Seq_2  1     ------------------------------------------------------------  0

Seq_1  3541  AAAAAAGCAGCTCTAGGCAATTTCTTGCTAGAGTTGTATGAGAAAGCTTCACTAACTTGA  3600

Seq_2  1     ------------------------------------------------------------  0

Seq_1  3601  GCCTATCTTTGTATTTTTCTGGTATTTTAATTTTAAAAGTTATTTGCCATGATTTAAACA  3660

Seq_2  1     ------------------------------------------------------------  0

Seq_1  3661  ATTTATTTAATTTCCAGGAAGAAATAGAGGTTGAGGCAAAGCTGTTGCGTGCTGGAAAAT  3720

Seq_2  1     ------------------------------------------------------------  0

Seq_1  3721  CAGTCGGTGTTGTCTCTGTTGATTTCAGGAAGAAAAGGACCGGGAAATTGATGGCTCAGG  3780

Seq_2  1     ------------------------------------------------------------  0

Seq_1  3781  CCCGTCACACAAAGTACCTTGCTCTATCTAGCAAATTGTGAGACTCATCTTCTTTTGCAA  3840
                                                       |||||||||||||||||||
Seq_2  1     -----------------------------------------GACTCATCTTCTTTTGCAA  19

Seq_1  3841  GATAACACATGGATCAGCCCAACTCTTCGTTAGTGTTCTACATTGGCAACTTCAGCCACA  3900
```

```
                 ||||||||||||||||||||||||||||||||||||||||||||||||||||||||||||
Seq_2  20        GATAACACATGGATCAGCCCAACTCTTCGTTAGTGTTCTACATTGGCAACTTCAGCCACA  79

Seq_1  3901      CAAATTACCTTTTGCCTTCATCAATAGGCAAGAAAGAATGACAAGATGTTAATGTTTTAC  3960
                 ||||||||||||||||||||||||||||||||||||||||||||||||||||||||||||
Seq_2  80        CAAATTACCTTTTGCCTTCATCAATAGGCAAGAAAGAATGACAAGATGTTAATGTTTTAC  139

Seq_1  3961      ATTGTTAATGAAAAAAGACCAGCTGTCCTATATCAAATTCATGTATCAATGGCTATTTTG  4020
                 ||||||||||||||||||||||||||||||||||||||||||||||||||||||||||||
Seq_2  140       ATTGTTAATGAAAAAAGACCAGCTGTCCTATATCAAATTCATGTATCAATGGCTATTTTG  199

Seq_1  4021      TGATTAGTAATTTAGTATAGTTCTGCTCATTTAATCATTTTATTTTCGGGTTTCAGCCGT  4080
                 ||||||||||||||||||||||||||||||||||||||||||||||||||||||||||||
Seq_2  200       TGATTAGTAATTTAGTATAGTTCTGCTCATTTAATCATTTTATTTTCGGGTTTCAGCCGT  259

Seq_1  4081      TGCCAAATAATGTTTTGACAATTGCAAGGCACAATTCCTGTTTGGAGATCACGAACTTCA  4140
                 |||||||||||||||||||||| |||||||||||||||||||||||||||||||||||||
Seq_2  260       TGCCAAATAATGTTTTGACAATCGCAAGGCACAATTCCTGTTTGGAGATCACGAACTTCA  319

Seq_1  4141      CAGGAAAACGTAGTGATTCCTCCTGAATTCCAATAAAGGCCCTTAGGATAAGTGGCCGCT  4200
                 ||||||||||||||||||||||||||||||||||||||||||||||||||||||||||||
Seq_2  320       CAGGAAAACGTAGTGATTCCTCCTGAATTCCAATAAAGGCCCTTAGGATAAGTGGCCGCT  379

Seq_1  4201      GGAATTGAACAGTACTTAGATGTTACTGTACTGGTGCTAGATCTTCAGTTGATTGGCAAC  4260
                 ||||||||||||||||||||||||||||||| ||||||||||||||||||||||||||||
Seq_2  380       GGAATTGAACAGTACTTAGATGTTACTGTACCGGTGCTAGATCTTCAGTTGATTGGCAAC  439

Seq_1  4261      TGTATTCTGCAGTCATCTTGGGGGAGATCTTCTCTGCTGAAACGAGTGGATCATTGTTGT  4320
                 ||||||||||||||||||||||||||||||||||||||||||||||||||||||||||||
Seq_2  440       TGTATTCTGCAGTCATCTTGGGGGAGATCTTCTCTGCTGAAACGAGTGGATCATTGTTGT  499

Seq_1  4321      ATTATTGGTTTAGTGTCTTTAGATAGTTGGTGAAAACAGGCCCAGCTGCAATCATGCTCG  4380
                 ||||||||||||||||||||||||||||||||||||||||||||||||||||||||||||
Seq_2  500       ATTATTGGTTTAGTGTCTTTAGATAGTTGGTGAAAACAGGCCCAGCTGCAATCATGCTCG  559

Seq_1  4381      CGTATGAGTGTACAGACGATGTGTTCCTCTGCGCTGTCCCTGAGTGTCTCTAGCTGTTTG  4440
                 ||||||||||||||||||||||||||||||||||||||||||||||||||||||||||||
Seq_2  560       CGTATGAGTGTACAGACGATGTGTTCCTCTGCGCTGTCCCTGAGTGTCTCTAGCTGTTTG  619

Seq_1  4441      GCTGACTTCAGTAGGGAAACAAAAACAAAACTGTCCAACTCTGAACTTTCTTCCTGTGCT  4500
                 ||||||||||||||||||||||||||||||||||||||||||||||||||||||||||||
Seq_2  620       GCTGACTTCAGTAGGGAAACAAAAACAAAACTGTCCAACTCTGAACTTTCTTCCTGTGCT  679

Seq_1  4501      CATTTGGCTGTGACGCAGGTAGGTGCTAAACAAGACAAAAATCACACATCAAATGTCCAT  4560
                 ||||||||||||||||||||||||||||||||||||||||||||||||||||||||||||
Seq_2  680       CATTTGGCTGTGACGCAGGTAGGTGCTAAACAAGACAAAAATCACACATCAAATGTCCAT  739

Seq_1  4561      TTTGGCATTCTGAATTTCTTTCTGCCAAAAGTGACAGCCCCAGACCAGAATCAAAAGTAA  4620
                 ||||||||||||||||||||||||||||||||||||||||||||||||||||||||||||
Seq_2  740       TTTGGCATTCTGAATTTCTTTCTGCCAAAAGTGACAGCCCCAGACCAGAATCAAAAGTAA  799

Seq_1  4621      ACTTTACTTACATTTACATCAATTGGTTCATAGAATCACAATGCCACTATGTTTACGGTG  4680
                 ||||||||| ||||||||||||||||||||||||||||||||||||||||||||||||||
Seq_2  800       ACTTTACTTGCATTTACATCAATTGGTTCATAGAATCACAATGCCACTATGTTTACGGTG  859

Seq_1  4681      CGGGGTAATCTACCCGAGTCACCGAATTGGAAGAATCACAATTCACAAAGATGTTCTTGT  4740
```

```
                ||||||||||||||||||||||||||||||||||||||||||||||||||||||||||||
Seq_2  860      CGGGGTAATCTACCCGAGTCACCGAATTGGAAGAATCACAATTCACAAAGATGTTCTTGT  919

Seq_1  4741     GTGAGTGTTGATGACACACAGCTGGCTTGTAGAGAGGAGGAGACGCCTATACTGTTTCTA  4800
                ||||||||||||||||||||||||||||||||||||||||||||||||||||||||||||
Seq_2  920      GTGAGTGTTGATGACACACAGCTGGCTTGTAGAGAGGAGGAGACGCCTATACTGTTTCTA  979

Seq_1  4801     CTAATGAGGGAGGCGGTTGGATGTCCCTGTCCTGGCTCTCCTTTCCCCTTTATTAGTGCG  4860
                ||||||||||||||||||||||||||||||||||||||||||||||||||||||||||||
Seq_2  980      CTAATGAGGGAGGCGGTTGGATGTCCCTGTCCTGGCTCTCCTTTCCCCTTTATTAGTGCG  1039

Seq_1  4861     AGCTAACCTTGTGTCGCCATGCGAGTGAGCCAGCCCGATAAAATCACAAAACACGCACGC  4920
                ||||||||||||||||||||||||||||||||||||||||||||||||||||||||||||
Seq_2  1040     AGCTAACCTTGTGTCGCCATGCGAGTGAGCCAGCCCGATAAAATCACAAAACACGCACGC  1099

Seq_1  4921     GTCCTCCGATATTTTTCTCCCACTAAAACACAGCACAGCACACGCTTTATTTTTACCCCC  4980
                ||||||||||||||||||||||||||||||||||||||||||||||||||||||||||||
Seq_2  1100     GTCCTCCGATATTTTTCTCCCACTAAAACACAGCACAGCACACGCTTTATTTTTACCCCC  1159

Seq_1  4981     ACAAACGCACACGCAAAAGGCGTCCCAGAAAGATGCCGTACACCTTTCTGTCTTTGAGAT  5040
                ||||||||||||||||||||||||||||||||||||||||||||||||||||||||||||
Seq_2  1160     ACAAACGCACACGCAAAAGGCGTCCCAGAAAGATGCCGTACACCTTTCTGTCTTTGAGAT  1219

Seq_1  5041     CTAGAGATGATATCATGCATGCATCATTACTTAGCCATGACACTTGACACAAACAACATC  5100
                ||||||||||||||||||||||||||||||||||||||||||||||||||||||||||||
Seq_2  1220     CTAGAGATGATATCATGCATGCATCATTACTTAGCCATGACACTTGACACAAACAACATC  1279

Seq_1  5101     TCGTGACCAATGGACACGATCGAATTTTGTACGGGTCTTGATTCTTTATTTACTTATTCA  5160
                ||||||||||||||||||||||||||||||||||||||||||||||||||||||||||||
Seq_2  1280     TCGTGACCAATGGACACGATCGAATTTTGTACGGGTCTTGATTCTTTATTTACTTATTCA  1339

Seq_1  5161     CACCTTTACTAAATCCCTTGCTAAATAATTATGAAAACAAGTGACAATGAAGAAGATCGA  5220
                ||||||||||||||||||||||||||||||||||||||||||||||||||||||||||||
Seq_2  1340     CACCTTTACTAAATCCCTTGCTAAATAATTATGAAAACAAGTGACAATGAAGAAGATCGA  1399

Seq_1  5221     TGGAGAGAAAGAAAAAATAAATATGGAATGATGGTATGCTGCATTTCTCTGTACTCTAAT  5280
                ||||||||||||||||||||||||||||||||||||||||||||||||||||||||||||
Seq_2  1400     TGGAGAGAAAGAAAAAATAAATATGGAATGATGGTATGCTGCATTTCTCTGTACTCTAAT  1459

Seq_1  5281     TACATACACCCATGAATAACACCACTTAAATTACTCGTATGCGACATCTAGTATAATTTA  5340
                ||||||||||||||||||||||||||||||||||||||||||||||||||||||||||||
Seq_2  1460     TACATACACCCATGAATAACACCACTTAAATTACTCGTATGCGACATCTAGTATAATTTA  1519

Seq_1  5341     CTTGGCATTCGTTGAAGCATGACACTTGGACTTGCTGGCGATCGATGATGACCCGGTCAG  5400
                ||||||||||||||||||||||||||||||||||||||||||||||||||||||||||||
Seq_2  1520     CTTGGCATTCGTTGAAGCATGACACTTGGACTTGCTGGCGATCGATGATGACCCGGTCAG  1579

Seq_1  5401     GAATAAATTCACCGTCCAGCCGCGTTTGGTTGGCGGTGGCGAGCGGAGGCGGAGGAGGAG  5460
                ||||||||||||||||||||||||||||||||||||||||||||||||||||||||||||
Seq_2  1580     GAATAAATTCACCGTCCAGCCGCGTTTGGTTGGCGGTGGCGAGCGGAGGCGGAGGAGGAG  1639

Seq_1  5461     ------------AAGGTCTTCTCGACGACGAAGAGCCTCCTGAAGATCCTCATGAGCAGC  5508
                            ||||||||||||||||||||||||||||||||||||||||||||||||
Seq_2  1640     GCAGAGGAGGAGAAGGTCTTCTCGACGACGAAGAGCCTCCTGAAGATCCTCATGAGCAGC  1699

Seq_1  5509     ACCCAGCTGCTGGCCCGGCGGTAGTACTGCCCCGTCCCGCCGGCCCTCGACGACGCCTGC  5568
```

```
                ||||||||||||||||||||||||||||||||||||||||||||||||||||||||||||
Seq_2  1700   ACCCAGCTGCTGGCCCGGCGGTAGTACTGCCCCGTCCCGCCGGCCCTCGACGACGCCTGC  1759

Seq_1  5569   AGCATCGGCATCGGCGCGCACCCCCTTCCCCGCTCCTCCGACTCCGTGTCCGACTCCACC  5628
              ||||||||||||||||||||||||||||||||||||||||||||||||||||||||||||
Seq_2  1760   AGCATCGGCATCGGCGCGCACCCCCTTCCCCGCTCCTCCGACTCCGTGTCCGACTCCACC  1819

Seq_1  5629   TCGCACGCCGCCGCCTTCTGCTTGGAGCAACGGCGCAGCGCCTCCAGCTCCTCGTTGAAG  5688
              ||||||||||||||||||||||||||||||||||||||||||||||||||||||||||||
Seq_2  1820   TCGCACGCCGCCGCCTTCTGCTTGGAGCAACGGCGCAGCGCCTCCAGCTCCTCGTTGAAG  1879

Seq_1  5689   TTCTCCCACAGCATGTCCATCTTCTCCTGATCCCCTTCCACGCCTCCGCGGACGAAGCCC  5748
              ||||||||||||||||||||||||||||||||||||||||||||||||||||||||||||
Seq_2  1880   TTCTCCCACAGCATGTCCATCTTCTCCTGATCCCCTTCCACGCCTCCGCGGACGAAGCCC  1939

Seq_1  5749   CTTTTAATGTCATCCAGATCTTCTCCTCCGGCTCCTCTTTCCCCCTCGCCCTCCTCGGCG  5808
              |||||||||||||||||| |||||||||||||||||||||||||||||||| ||||||||
Seq_2  1940   CTTTTAATGTCATCCAGAGCTTCTCCTCCGGCTCCTCTTTCCCCCTCGCCCCCCTCGGCG  1999

Seq_1  5809   TCAGCCCAAGCCGGCTCTTGTTCTTCTTCTTCTTCCATGTCGTCTTCAGTTCGGCTGGTC  5868
              ||||||||||||||||||||||||||||||||||||||||||||||||||||||||||||
Seq_2  2000   TCAGCCCAAGCCGGCTCTTGTTCTTCTTCTTCTTCCATGTCGTCTTCAGTTCGGCTGGTC  2059

Seq_1  5869   ATCAGCGGCGGTGGCGCGACGGCGACGGCGGCGTCGACGGGGAACCAGAGCGGGGAATTG  5928
              ||||||||||||||||||||||||||||||||||||||||||||||||||||||||||||
Seq_2  2060   ATCAGCGGCGGTGGCGCGACGGCGACGGCGGCGTCGACGGGGAACCAGAGCGGGGAATTG  2119

Seq_1  5929   GCGAAGTGTGGGAAGGGAAGGAGGCTGTGGTGGCGGTGGTGATGTAGGCACAGCGTGTCC  5988
              ||||||||||||||||||||||||||||||||||||||||||||||||||||||||||||
Seq_2  2120   GCGAAGTGTGGGAAGGGAAGGAGGCTGTGGTGGCGGTGGTGATGTAGGCACAGCGTGTCC  2179

Seq_1  5989   GCGGGGGACGACGACGACTCTGCCGGGGCAGCGGTTGCGGGGAAGGTGAAGTCGTCCATT  6048
              ||||||||||||||||||||||||||||||| ||||||||||||||||||||||||||||
Seq_2  2180   GCGGGGGACGACGACGACTCTGCCGGGGCAGTGGTTGCGGGGAAGGTGAAGTCGTCCATT  2239

Seq_1  6049   ATCTGCCGCGGCAGCTCTCTCTATACCATACTCTGTTTCTAGCTCCGATCAATAATACTC  6108
              ||||||||||||||||||||||||||||||||||||||||||||||||||||||||||||
Seq_2  2240   ATCTGCCGCGGCAGCTCTCTCTATACCATACTCTGTTTCTAGCTCCGATCAATAATACTC  2299

Seq_1  6109   CTGGCTGTTGCTGACAAGGATACGTTGCCAGTCGGGTTAATTGGCTTCTGCCTGCCGCTC  6168
              |||||||||||||||||||||||||||||||||||||||| |||||||||||||||||||
Seq_2  2300   CTGGCTGTTGCTGACAAGGATACGTTGCCAGTCGGGTTAACTGGCTTCTGCCTGCCGCTC  2359

Seq_1  6169   GCTCTCTCGCTATAGCTGCTAACTGGCCCGGGTCATTATATACTTGGAGCGTGGTAGCAG  6228
              ||||||||||||||||||||||||||||||||||||||||||||||||||||||||||||
Seq_2  2360   GCTCTCTCGCTATAGCTGCTAACTGGCCCGGGTCATTATATACTTGGAGCGTGGTAGCAG  2419

Seq_1  6229   GGCACGGGAAGGCCGGGGGTAGATCGGCGCGGGGATCTATCTATCTACCGACAGAAGGCA  6288
              ||||||||||||||||||||||||||||||||||||||||||||||||||||||||||||
Seq_2  2420   GGCACGGGAAGGCCGGGGGTAGATCGGCGCGGGGATCTATCTATCTACCGACAGAAGGCA  2479

Seq_1  6289   AGGAGCCAAGGACGGCGGTACGTGACGGCCGTTGGAAGCGTACAAACTAAGCTTAAGAAA  6348
              ||||||||||||||||||||||||||||||||||||||||||||||||||||||||||||
Seq_2  2480   AGGAGCCAAGGACGGCGGTACGTGACGGCCGTTGGAAGCGTACAAACTAAGCTTAAGAAA  2539

Seq_1  6349   CGTACGTACAACGTACGTACGTGCATTTGGCGTGTATTGGATGAAGTCAGCATGTGACTG  6408
```

```
             ||||||||||||||||||||||||||||||||||||||||||||||||||||||||||||
Seq_2  2540  CGTACGTACAACGTACGTACGTGCATTTGGCGTGTATTGGATGAAGTCAGCATGTGACTG  2599


Seq_1  6409  TTTGTGGCTCGTCTCGCCTGAATTAAGGGCGAGCCCTGTTTTTTGCCGGGGCCAGTGAG  6468
             ||||||||||||||||||||||||||||||||||||||||||||||||||||||||||||
Seq_2  2600  TTTGTGGCTCGTCTCGCCTGAATTAAGGGCGAGCCCTGTTTTTTGCCGGGGCCAGTGAG  2659


Seq_1  6469  CGAGCGATCGACCGGATCCGACCAACGCTGTACGGAGTAGGTGGCCAGCCG-GAAGCGCG  6527
             ||||||||||||||||||||||||||||||||||||||||||||||||| | | ||||||
Seq_2  2660  CGAGCGATCGACCGGATCCGACCAACGCTGTACGGAGTAGGTGGCCAGC-GGGGAGCGCG  2718


Seq_1  6528  GTTTACATGGATATACAATGGTGATTTTGGTCGATCGATCGGCCTCCGGCG-GGTCGTTT  6586
             ||||||||||||||||||||||||||||||||||||||||||||||||||| ||||||||
Seq_2  2719  GTTTACATGGATATACAATGGTGATTTTGGTCGATCGATCGGCCTCCGGCGCGGTCGTTT  2778


Seq_1  6587  TCGGCCGTCAAGTGGGGCGCGCCACCATATATGGCACCGCATCTCGCACTTGGAGATATG  6646
             ||||||||||||||||||||||||||||||||||||||||||||||||||||||||||||
Seq_2  2779  TCGGCCGTCAAGTGGGGCGCGCCACCATATATGGCACCGCATCTCGCACTTGGAGATATG  2838


Seq_1  6647  GCATGTTTAGTTTTTTTATTTTATAATCACCTGTAATTTTATTCAAACTCGACGTTCATC  6706
             | ||||||||||||||||||||||||||||| ||| ||||||||||||||||||||||| 
Seq_2  2839  GAATGTTTAGTTTTTTTATTTTATAATCACCCGTATTTTTATTCAAACTCGACGTTCATT  2898


Seq_1  6707  ACACCTACATTTGGATAAAACAATCCGCAAAACTACAAACTAACTCTATAAGTAAGAATT  6766
             |||| |||||||||||||||||||||||||||||||||||||||||||||||||||||||
Seq_2  2899  ACACGTACATTTGGATAAAACAATCCGCAAAACTACAAACTAACTCTATAAGTAAGAATT  2958


Seq_1  6767  ACATTCAAGTCTTTCAAAGTAATCTTTTTCATGATATCAATCTTCGCAACACAAAGTACC  6826
             ||||||||||||||||||||||||||||||||||||||||||||||||||||||||||||
Seq_2  2959  ACATTCAAGTCTTTCAAAGTAATCTTTTTCATGATATCAATCTTCGCAACACAAAGTACC  3018


Seq_1  6827  GCCAAGACTTCAGTAAAAATGTGGCATGTTTAGTTCTGCGGGGCCATCGGGAGGTAATCT  6886
             |||||| |||||||||||||||||||||||||||||||||||||||||||||||||||||
Seq_2  3019  GCCAAGGCTTCAGTAAAAATGTGGCATGTTTAGTTCTGCGGGGCCATCGGGAGGTAATCT  3078


Seq_1  6887  TAACTAGAGAGGTAGGCCGGCCGGGCCGGCCCAAAACTGTAAATAGTACTCCGATTAATA  6946
              |||||||||||||||||||||||||||||||||||||||||||||||||||| ||||||
Seq_2  3079  GAACTAGAGAGGTAGGCCGGCCGGGCCGGCCCAAAACTGTAAATAGTACTCCGGTTAATA  3138


Seq_1  6947  CCTTCAAAATATTCACATCCAACGAGAGCGCTGCCCTAGCCGTCATCGGTTGAAGATTTA  7006
             |||||||||||||||||||||||||| |||||||||||| ||||||||||||||||||||
Seq_2  3139  CCTTCAAAATATTCACATCCAACGAG-GCGCTGCCCTAGACGTCATCGGTTGAAGATTTA  3197


Seq_1  7007  GCGGCCCCCTCGCCTCTTGCCGCCGTCTTGGCGTTGCGAGGTAGGGGGAACCTCGGTTTC  7066
             ||||||||||||||||  |||||||||||||||||| |||||||||||||||||||||||
Seq_2  3198  GCGGCCCCCTCGCCTCCGGCCGCCGTCTTGGCGTTGTGAGGTAGGGGGAACCTCGGTTTC  3257


Seq_1  7067  TCCTAAGAGTATCCTGAAGTGTTTGCTTGTTTCAATTGTTTGGTTCTAATCGGCAACGCA  7126
             ||| |||||||| ||||||||||||||| |||||||||||||||||| ||||||||||||
Seq_2  3258  TCCCAAGAGTATTCTGAAGTGTTTGCTTATTTCAATTGTTTGGTTCTGATCGGCAACGCA  3317


Seq_1  7127  ATGGCGGAGGTTGCAATACCGTCGCGAATAAATATACTATGCTTTTCCTCTGCTGTGGTG  7186
             |||||||||||||||||||||||||||||||||||||||| |||||||||||||||||||
Seq_2  3318  ATGGCGGAGGTTGCAATACCGTCGCGAATAAATATACTATACTTTTCCTCTGCTGTGGTG  3377


Seq_1  7187  GTGCCTCAACGGGCTGTTGCTGCGGAGTTAGAGTTTTTAGTCCCCGTAAGTTTAGTTACC  7246
```

```
                ||| ||||||||||||||||||||||||||||||||||||||||||||||||||||||||
Seq_2  3378   GTGGCTCAACGGGCTGTTGCTGCGGAGTTAGAGTTTTTAGTCCCCGTAAGTTTAGTTACC  3437


Seq_1  7247   CGATCTTGCGGATCTCGAGTTTCTATACTCGCAGATCCGTTTAAATATATCTGCTGAGTT  7306
              ||||||||||||||||||||||||||||||||||||||||||||||||||||||||||||
Seq_2  3438   CGATCTTGCGGATCTCGAGTTTCTATACTCGCAGATCCGTTTAAATATATCTGCTGAGTT  3497


Seq_1  7307   TTTGTCAGTGTGTCATGATGCTTTTGTTGGTTTTGTCTTTTACTATAATTTTGGAATCCT  7366
              ||||||||||||||||||||||||||||||||||||||||||||||||||||||||||||
Seq_2  3498   TTTGTCAGTGTGTCATGATGCTTTTGTTGGTTTTGTCTTTTACTATAATTTTGGAATCCT  3557


Seq_1  7367   TCTCTGGCGATTCGACGACCTGCACCTTTAGGGGATCATTCTCGGTCCAAATACGTTCAA  7426
              ||||||||||||||||||||||||||||||||||||||||||||| ||||||||||||||
Seq_2  3558   TCTCTGGCGATTCGACGACCTGCACCTTTAGGGGATCATTCTCGGCCCAAATACGTTCAA  3617


Seq_1  7427   TGCTTACGGCTTCTCACCTTTTTGGATGAGCGACTCAAGGGCATTTTCAAAACCTTTAAA  7486
               ||||||||||||||||||| |||||||||||||||||||||| ||||||||||||||||
Seq_2  3618   CGCTTACGGCTTCTCACCTTCTTGGATGAGCGACTCAAGGGCAGTTTCAAAACCTTTAAA  3677


Seq_1  7487   GGTAATCAAGTTCGTGTAGGGATACTAGTGACGATGTAACTGCTCCAGTCTGATTGCAAT  7546
              ||||||||||||| ||||||||||||| ||||||||||||||||||||||| ||||||||
Seq_2  3678   GGTAATCAAGTTCATGTAGGGATACTATTGACGATGTAACTGCTCCAGTCTTATTGCAAT  3737


Seq_1  7547   CTCTATGTTGAGGCCTCAACAGTATTTCATGCTGCAAATGATGATATCATGGAGAAGATG  7606
              ||||||||||||||||||||||||||||||||||||||||||||||||||||||||||||
Seq_2  3738   CTCTATGTTGAGGCCTCAACAGTATTTCATGCTGCAAATGATGATATCATGGAGAAGATG  3797


Seq_1  7607   TATTTAAGAGATTTCATTATAATTCTTAATTATAAGAGTTACCTTGTAGTTTTTAGAACC  7666
              ||||||||||||||||||||||||||||||||||||||||||||||||||||||||||||
Seq_2  3798   TATTTAAGAGATTTCATTATAATTCTTAATTATAAGAGTTACCTTGTAGTTTTTAGAACC  3857


Seq_1  7667   TATGATCGAGAGCATTGTACATGCACTAGTTCTCGCGTTTGAATAAAATTACAGTTATTC  7726
              |||||||||||||||||||||||||||||||||||||||||||||||||||| |||||||
Seq_2  3858   TATGATCGAGAGCATTGTACATGCACTAGTTCTCGCGTTTGAATAAAATTACCGTTATTC  3917


Seq_1  7727   TAAAAAAAAAGCTAATATATACATATCTAAAAAATACGGACCGTTCCCTTCACCATCGTC  7786
              |||||||||||||||||||||||||||||||||||||||| ||||||||| |||||||||
Seq_2  3918   TAAAAAAAAAGCTAATATATACATATCTAAAAAATACGGAGCGTTCCCTTTACCATCGTC  3977


Seq_1  7787   TGTCAAACTTTCCATAAAATTTGTGGGCCCACTAGGGTTGCATGTATATTAGGCTTACTA  7846
              |||||||||||||||||| |||||||||||||||||||||||||||||||||||||||||
Seq_2  3978   TGTCAAACTTTCCATAAA-TTTGTGGGCCCACTAGGGTTGCATGTATATTAGGCTTACTA  4036


Seq_1  7847   AGCAGTTGGCCAAATTGGACTAGAACTCCACCAGATCAGTCCAGATTCAATCAATCACGA  7906
              ||| ||||||||||||||||| ||||||||||||||||||||||||||||||||||||||
Seq_2  4037   AGCGGTTGGCCAAATTGGACTGGAACTCCACCAGATCAGTCCAGATTCAATCAATCACGA  4096


Seq_1  7907   ATACAACACAACTTCAAACTTAACGTATCAATCACGATCGGTCCAATACAGCCTCCCATA  7966
              ||||||||||||||||||||||||||||||||||||||||||||||||||||||||| ||
Seq_2  4097   ATACAACACAACTTCAAACTTAACGTATCAATCACGATCGGTCCAATACAGCCTCCCGTA  4156


Seq_1  7967   ACGAAGCATGTATCAATATCGGTATGGAGTACGGATCGTATGAAAAAAAGTCATAAAGCA  8026
              ||||||||||||||||||||||||||||||| ||||||||||||||||||||||||||||
Seq_2  4157   ACGAAGCATGTATCAATATCGGTATGGAGTATGGATCGTATGAAAAAAAGTCATAAAGCA  4216


Seq_1  8027   ATCTCAGGAAAATCAATCCAATCCGAAATAGAAAAACTGATACATCCCTCTCTCGCAGCG  8086
```

```
             |||||||||||||||||||||||||||| |||||||||||||||||||||||||||||||
Seq_2  4217  ATCTCAGGAAAATCAATCCAATCCGAAACAGAAAAACTGATACATCCCTCTCTCGCAGCG  4276

Seq_1  8087  AAGCTCCACGCCATGGCCCATGAATCGTTACGATAGTCGGGATAGGATCGATCAATGTAC  8146
             |||||||||||||||||||||||||||||| |||||||||||||||||||||||||||||
Seq_2  4277  AAGCTCCACGCCATGGCCCATGAATCGTTATGATAGTCGGGATAGGATCGATCAATGTAC  4336

Seq_1  8147  TTGCTCGTAAATTATGCCTACGACCTGCAAGCGTCCCTAAAAAAATTATCACTCCCTCTG  8206
             ||| ||||||||||||||||
Seq_2  4337  TTGTTCGTAAATTATGCCTA----------------------------------------  4356

Seq_1  8207  ATCAATATTAATTGTGTCAAATTTGCCCAAATATGGATGTATTTATGCCTAAAAAGCGTC  8266

Seq_2  4357  ------------------------------------------------------------  4356

Seq_1  8267  TAGATACATGTAATATTTCGGAGGGAGTACGTCTACGGATGGACAGAAACTACAAGTCCG  8326

Seq_2  4357  ------------------------------------------------------------  4356

Seq_1  8327  AACGCATGGCACAAGCCGCTGCACAACGCAATCACCCATGAAGATGCAAATCTGCACAGG  8386

Seq_2  4357  ------------------------------------------------------------  4356

Seq_1  8387  CGCACAATGACAGTTCAACTGTCGGCAGATCGGCCTATACGAGGGATTCTACACTAGACT  8446

Seq_2  4357  ------------------------------------------------------------  4356

Seq_1  8447  CTAGACATCGCTGACGCCTTGAAGCCAAAATTTATTTAGGTGGATCTATTTGTAAGCTAG  8506

Seq_2  4357  ------------------------------------------------------------  4356

Seq_1  8507  CCTTTTGTGATAGGTTTAATTAGCCCTAATTGTTGGTTAAAGTTTTGAGATTGTCTTATC  8566
                                         ||||||||||||||||||||||||||||||||
Seq_2  4357  ----------------------------ATTGTTGGTTAAAGTTTTGAGATTGTCTTATC  4388

Seq_1  8567  GACTAAGAAAAACGCATCCGAGAAAAGAAAAAAAAGAGAAAGCGAGTAGATGACTTGATA  8626
             ||||||||||||||||||||||||||||||||||||||||||||||||||||||||||||
Seq_2  4389  GACTAAGAAAAACGCATCCGAGAAAAGAAAAAAAAGAGAAAGCGAGTAGATGACTTGATA  4448

Seq_1  8627  AAATCACAGAGAGGATAAATTTATAAAATTTTATGTGTTGTCACGGGTTTTTGACATATT  8686
             |||||||| |||||||||||||||||||||||||||||||| ||| | ||||||||||||
Seq_2  4449  AAATCACACAGAGGATAAATTTATAAAATTTTATGTGTTGTGACGAGATTTTGACATATT  4508

Seq_1  8687  TTATACATACATATTTGGATATTATTTAGATGTTTATATTAGGGGCCCGGTTTTTTAAGT  8746
             |||||||||||||||||||||||||||||||| ||||||||||||||||||||||||| |
Seq_2  4509  TTATACATACATATTTGGATATTATTTAGATGCTTATATTAGGGGCCCGGTTTTTTAAAT  4568

Seq_1  8747  TCGCCCGAGGCCCCCGAAATCACAGGAGCGGCCCTGGCCGGATGCCACCCAGTTACCTAC  8806
             ||||||||||||||||||||||||||||||||||||||||||||||||||||||||||||
Seq_2  4569  TCGCCCGAGGCCCCCGAAATCACAGGAGCGGCCCTGGCCGGATGCCACCCAGTTACCTAC  4628

Seq_1  8807  CATATCATTGGCAGCCGCCTTCATTGCTCCCATCAACACAACGCCAGGGCCACCGCCGCA  8866
             |||||||||||||||||||||| |||||||||||||||||||||||||||||||||||||
Seq_2  4629  CATATCATTGGCAGCCGCCTTCGTTGCTCCCATCAACACAACGCCAGGGCCACCGCCGCA  4688

Seq_1  8867  TCCACCCTCCAATCCGTGACCACCAGGTCCACCACCACGCTGCTGCCTATCATTGTTTCT  8926
```

```
             ||||||||||||||||||||||||||||||||||||||||||||||||||||||||||||
Seq_2  4689  TCCACCCTCCAATCCGTGACCACCAGGTCCACCACCACGCTGCTGCCTATCATTGTTTCT  4748

Seq_1  8927  -CCTCACAGGTGAGCCCCAGGATCGTTCCCTCCTCTTATCTGAGCAATCCGGCCATCGCC  8985
              |||||||||||||||||||||||||||||||||||||||||||||||||||||||||||
Seq_2  4749  TCCTCACAGGTGAGCCCCAGGATCGTTCCCTCCTCTTATCTGAGCAATCCGGCCATCGCC  4808

Seq_1  8986  TCACCTCGAGCCGCCTTGCTCCCTTCTGTTTCCGCGCGAGGGGTTCCGTCGAATCAGGCC  9045
             ||||||||||||||||||||||||||||||||||||||||||||||||||||||||||||
Seq_2  4809  TCACCTCGAGCCGCCTTGCTCCCTTCTGTTTCCGCGCGAGGGGTTCCGTCGAATCAGGCC  4868

Seq_1  9046  GCCCTGGTGTGTCAGTACTTTGCCGGAAAGTGTGCACTTGCTAGCTAAAAGCTCCATCCA  9105
             |||||||||||||||||||||||||||||| ||||||||||||||||  |||||||||||
Seq_2  4869  GCCCTGGTGTGTCAGTACTTTGCCGGAAAGAGTGCACTTGCTAGCTACTAGCTCCATCCA  4928

Seq_1  9106  TTAATTCAGAGAGATACATCGTTTGTGGATTGAATTTAGCTAGCTACTAAATACCATGTT  9165
             ||||||||||||||||||||||||||||||||||||||||||||||||||||||||||||
Seq_2  4929  TTAATTCAGAGAGATACATCGTTTGTGGATTGAATTTAGCTAGCTACTAAATACCATGTT  4988

Seq_1  9166  TTAATTTTCTCAGTTGATCGGTGTATATACATTGCTTATGCTGCTATAGGTTTTTTTTA  9225
             |||||||||||||||| || ||||||||||||||||||||||||||||||||||||||||
Seq_2  4989  TTAATTTTCTCAGTTGGTCAGTGTATATACATTGCTTATGCTGCTATAGGTTTTTTTTA  5048

Seq_1  9226  TGGAATCTTGCAATGTGAGAGAAGGCTTATGGCCCGTATGGAAAGTCGGAAACGCAGCAA  9285
             ||||||||||||||||||||||||||||||||||||||||||||||||||||||||||||
Seq_2  5049  TGGAATCTTGCAATGTGAGAGAAGGCTTATGGCCCGTATGGAAAGTCGGAAACGCAGCAA  5108

Seq_1  9286  CAAAGATTTTTGTGATAGTGGTCCTCTGTCTTGCGAGCTTCTGGTTTCCGGAGGTCTTCA  9345
             ||||||||||||||||||||||||||||||||||| |||||||||||| |||||||||||
Seq_2  5109  CAAAGATTTTTGTGATAGTGGTCCTCTGTCTTGCGGGCTTCTGGTTTCTGGAGGTCTTCA  5168

Seq_1  9346  ACCGAGGGGTCGTCTCACTCACATGCATAATCAGGAAGAGGAACAGCATGACCGGGCTGT  9405
             |||||||||||||||||||| ||| ||||||||||||||| |||||||||||||||||||
Seq_2  5169  ACCGAGGGGTCGTCTCACTCGCATACATAATCAGGAAGAGAAACAGCATGACCGGGCTGT  5228

Seq_1  9406  CATTCACCTACTAGACGGCATCTACCTTCTGTGGACAAGCTCAGTAGGCCGTGGCCGTGG  9465
             ||||||||||||||||||||||||||||||||||||||||||||||||||||||||||||
Seq_2  5229  CATTCACCTACTAGACGGCATCTACCTTCTGTGGACAAGCTCAGTAGGCCGTGGCCGTGG  5288

Seq_1  9466  CAGCGCACGGGCATTTCGGCTAGTACCTTAAAAAAAACTAAAAAACTGATACGCCAATTT  9525
             ||||||||||||||||||||||||||||||||||||||||||||||||||||||||||||
Seq_2  5289  CAGCGCACGGGCATTTCGGCTAGTACCTTAAAAAAAACTAAAAAACTGATACGCCAATTT  5348

Seq_1  9526  CGCTGCATGACGTTCTTCTTGAGAGGAGATGATCCCATGTCCATTGGGCATGCTGTTGCT  9585
             ||||||||||||||||||||||||||||||||||||||||||||||||||||||||||||
Seq_2  5349  CGCTGCATGACGTTCTTCTTGAGAGGAGATGATCCCATGTCCATTGGGCATGCTGTTGCT  5408

Seq_1  9586  CAAACCATGGCAACCCAGAAGGTTGAGCTAGGTGAATTCACGATTTACACGGAGAAGGAG  9645
             ||||||||||||||||||||||||||||||||||||||||| ||||||||||||||||||
Seq_2  5409  CAAACCATGGCAACCCAGAAGGTTGAGCTAGGTGAATTCACAATTTACACGGAGAAGGAG  5468

Seq_1  9646  GACACAGTTTGCTGCGCTGATGATCTGATCCACTACGGGAGACAATTCATGTTGTTCTTT  9705
             ||||||||||||||||||||||||||||||||||||||||||||||||||||||||||||
Seq_2  5469  GACACAGTTTGCTGCGCTGATGATCTGATCCACTACGGGAGACAATTCATGTTGTTCTTT  5528

Seq_1  9706  GATGCCTGTCCAATTGCATTCGGTGGTCTCACACGTCTTGACTTAGAGAATTTGAGATTT  9765
```

```
               ||||||||||||||||||||||||||||||||||||||||||||||||||||||||||||
Seq_2  5529   GATGCCTGTCCAATTGCATTCGGTGGTCTCACACGTCTTGACTTAGAGAATTTGAGATTT  5588


Seq_1  9766   GGTTAATCTGACATCCGTAACGTCCTCAACACTTGCAAACGGTTAAAGCATCTACGCCTG  9825
              ||| ||||||||||||||||||||||||||||||||||||||||||||||||||||||||
Seq_2  5589   GGTGAATCTGACATCCGTAACGTCCTCAACACTTGCAAACGGTTAAAGCATCTACGCCTG  5648


Seq_1  9826   TTCAATTGTGACTCTGGCAGTAGTCAGACCGTGCTGCAAGTTGAACACTCACAACTTTGT  9885
              ||||||||||||||||||||||||||||||||||||||||||||||||||||||||||||
Seq_2  5649   TTCAATTGTGACTCTGGCAGTAGTCAGACCGTGCTGCAAGTTGAACACTCACAACTTTGT  5708


Seq_1  9886   GAGCTTGCTATTGTTGATTGTTCTATTGAAAGAGTCGAGCTCACCTCACTTCCCAAACTC  9945
              ||||||||||||||||||||||||||||||||||||||||||||||||||||||||||||
Seq_2  5709   GAGCTTGCTATTGTTGATTGTTCTATTGAAAGAGTCGAGCTCACCTCACTTCCCAAACTC  5768


Seq_1  9946   ACACGGTTCATATTTGAGTGTTGGATCGCTTTCGAAGATCCCCTATCTTTTGGTTATGTC  10005
              ||||||||||||||||||||||||||||||||||||||||||||||||||||||||||||
Seq_2  5769   ACACGGTTCATATTTGAGTGTTGGATCGCTTTCGAAGATCCCCTATCTTTTGGTTATGTC  5828


Seq_1  10006  CCGTTGCTCGAGGCTGTAAGCCTCGCCAATATTTGTCTTAGTTGGCACCAGATGGTCAAG  10065
              ||||||||||||||||||||||||||||||||||||||||||||||||||||||||||||
Seq_2  5829   CCGTTGCTCGAGGCTGTAAGCCTCGCCAATATTTGTCTTAGTTGGCACCAGATGGTCAAG  5888


Seq_1  10066  TTAAGTAAGTTTCTTTGGGTACCTCTGTGCGAGATTTGAAGTTGGGATTTGATTTCGAAA  10125
              ||||||||||||||||||||||||||||||||||||||||||||||||||||||||||||
Seq_2  5889   TTAAGTAAGTTTCTTTGGGTACCTCTGTGCGAGATTTGAAGTTGGGATTTGATTTCGAAA  5948


Seq_1  10126  GGGTAAGTAAAGAATATGGTTTTGCTAGCTCTGGTGACAACTTCTTGTCTGCTCATCATC  10185
              ||||||||||||||||||||||||||||||||||||||||||||||||||||||||||||
Seq_2  5949   GGGTAAGTAAAGAATATGGTTTTGCTAGCTCTGGTGACAACTTCTTGTCTGCTCATCATC  6008


Seq_1  10186  ATGTGTTTACTTGTTGCAGATTTGGGTTCAACCAGAATGTCTGACGGTAAGGCTGGCATC  10245
              ||||||||||||||||||||||||||||||||||||||||||||||||||||||||||||
Seq_2  6009   ATGTGTTTACTTGTTGCAGATTTGGGTTCAACCAGAATGTCTGACGGTAAGGCTGGCATC  6068


Seq_1  10246  TGTGTTCAACCAAATAAGGTTTGTGAATCTAGCTGATATTCCTGAAGGTTATGATCTCAC  10305
              |||||||||| |||||||||||||||||||||||||||||||||||||||||||||||||
Seq_2  6069   TGTGTTCAACTAAATAAGGTTTGTGAATCTAGCTGATATTCCTGAAGGTTATGATCTCAC  6128


Seq_1  10306  CTGGACATTATTCTTTCTTAAA--------------------------------------  10327
              ||||||||||||||||||||||
Seq_2  6129   CTGGACATTATTCTTTCTTAAAGCTGCACCGAACTTAAAGGAGCTATATATGACGGTATG  6188


Seq_1  10328  ------------------------------------------------------------  10327

Seq_2  6189   TCCTTACCTAAAGCTTGCTTTTTCTTTCCATAGCATGTGCCCGTGCTCAATGTTGAATTT  6248


Seq_1  10328  ------------------------------------------------------------  10327

Seq_2  6249   TGCCAGTTTTTCATATTGAACATAAATCGCTGCAAACGAGTAACTGTGTTCTATTTAACT  6308


Seq_1  10328  ------------------------------------------------------------  10327

Seq_2  6309   CTGTCTTCCTATGGCATCTAGGTTTGGGATCATCTTTGTGTAATGAAAACGAATGCAGCA  6368


Seq_1  10328  ------------------------------------------------------------  10327
```

```
Seq_2  6369  GATAGGAGGGCACTCTTGTATAGCGAGAAGAAGGGTATAGAGTGGGAATTATCTCCATCT  6428

Seq_1  10328 ------------------------------------------------------------  10327

Seq_2  6429  GATTTCCAACATCAGAGTCTGGCCACAGTCGCCATCTTTGGGTTTCAAGCTCAAGACTAC  6488

Seq_1  10328 ------------------  10327

Seq_2  6489  ATGATGTATTCTTGTATA  6506
```

BdindelWSU_19, upstream

>Bradi5g11240

TATTTTTTTGGTCAGACACGCGTAGGTTATTATGTTGATGGAAACTGGCCTGGATAACATTGATTGGCTAGGCCATAGCGTCTTCGTG
ATAGACACAATTATGGAACGGGAGGTCCGTCCAAATCTGGCTACAGTACCCATTCAATGTCTGGTTTTGTGTCTATTATATACTTAAA
GCAATATGAAGGATACGTGGAGCCTCCACGTTGATCTACATCATTCTAATTATTTTCACCGTTGGATATGAGATTAAGAGTCCACGTTT
CAACACGTTATGTTGGGTTCCTAAAAAGAACATGTTATGTTGGAAAAAAAAACTGCTAAAACAGACAGAACCATTGTTACGTACCCA
GAGATCGAATCCCACGCCTCCCAGGCTCCCACGTACATTAGCTTTTTCTTTTTCTTTTTGAGCTGCATATACGTTAGCTTTTGGCCTTAG
TTCTTTATATGGGATTCAATACCAGATCGATCCCGCTTCCCACAAATCAGTACAAAATTCCAATTGATCGAAACAACTAAATAAAATAA
CAAGCAATCTTCCCCGTAAAAAAAACAATGTTTTTTTCTCTAAAATTCCAATTGATCGAAACAACTAAAATGACGACTATAATTTTGAA
TAAACCGAAAACTATGTCAAAAATGTTTGAAGCAAACTTCTCTAACTGCCTCATTCGCATCTTGATATCTATTATATATACTAAATAAAAG
CAATATGATTTTCTCCAAATATCATGTAACAGAACGTTAGAAGATCAAACATGCATAAGCTGGACTATCCTTTTAAGTTTTACTAAGCC
GCATGAGCCGGCCACCCTAGCGGAAGAAAACTTGAGATCGTTGACGCGCGGTGGCCAGACACACCCCTCACAAGAAAGGTTGCCAT
GCCATTGAATCGTCGCCGAACCAATGAGCAAGAGGACAAAAGGCACGAAATCACGAACCGTGAGATCCACAATCTTCATGTAATATA
TGATGATAGCCACACCAGAAAAAGGATGGAGTTGGAAGACCATCAATCCATGCACCGTTGTTTCCACCGTAGACCGCCGCCGCTAAG
AACACACAAAACCTTACAAAAAAAGGTACTACTAATACAATGGCCAGACGTTGATCCGTGATTTCCCACACCTCCAGGCGCCCAATAG
GCTATAAAAGACGTGGGGAACCGATAAAAATGGAAACCTGGTCAAGATTTAATGAGGATGACATATTTGATTTGATTGATGTGCTAG
AAATAAACATCACGGGATGGCATATTTGATTTCATTGATGAGTTAGAAATAAACATCGCGTAGGGCCATTACAAATTAATAAAAGGG
TGCAAGGTAGCTGCCGGACGTGATTTGCAACTAGAGCTATATGCTTACGTGAATCACGTCTGACATGATTTTCAAGATAATTTTTCTTT
CTAAAAAATAAATTTACACGATCAATAACAATTTGTGTTTTAAATATGCATGCATTTTTTTCACGACCATGATTTATGCTAGGGAAACA
TTATGTTTGATCGATGAAAGGAAACATTCTGATTGATAAATAAAAGTATATTTTCTTTACACTACCAGAAACAAAATTACGCAGTAGC
GAAAACAATGTTTTGGTGTAATAAATAAAGGTTCATCGATACAAATTCATTTGACGGGTGTTCTAGCAACCGTATGTATGTTTGCGAA
AGGAAATTGTCATATATTAAAGCAACTAGCTCTTACAGTACTATATTCAATTGACCAAAAATAAAATTACACAACTCCTTGAGGAAAA
ATGACTTCGGACTTCCAAACCACGTCGAAGCTATGACAATGTTTTTGACGTACGAGCCGGGATATAACCGATTTGAGCCAGAAGACA
CCCGTGATATTGATCTGAGAAGCAGACCATCATTCCAAAAGGCGACGACGATGGTTGGATGACCATTGTAAATTCAGCCAATCAGAT
CAGTCGAGACCTAACCTTACTAGCTCCGAGGCGAAACTGAAGTTTGACAAACCTCGCCAATCAGAAAAAATGCGCACATACAGAACA
CGAACACACGGAAACGAATCGACTATTTCATAAGAGTCGCCATCAACGAGATCGTCACTGATGAGGTGGATCATTAGTCGAGAAAAC
CTAATTCTTAAACCGCACGGCGCCACAATATCCACCATAGCGTCACAGCCGAGGAACCAAACCTAACTCTAAGAAGCCTAACTACCAG
CCCTCAAACTGGTCGGAGTCCCACCCCTCCTGCTGTAGCGGCAAACAGGGAGGAGGGGACGTCCGCAACGCTTGCGGGAAGCAGTG
GTGCGAAAATGCTGTTTTGGAACAGAAGCGGATAGGGTTTCTGGAGATAGTACCTTTTTATTTTGACGAGTTATGAAAACCTTACGCA
AGACGTCATGTCAACGACATATGATGAAGAGTTCGCCGAAATGCATGGAGGATATGGCTATTGTGACTTCACACATGAGTGAGATCA
TCTATCATTGATAACTACCACCCCTCCTACCTGGCAAATGGGGGAGAAGGGGACCTTCACAGCTCCTGCGGGAAGCAGTGGAGCGG
AAATGCTTTTTTTGAATAGAAGCGGTTTCTGGAGATAGATATCTTTTTATTTTGATGAGTTATGTAAATCTTATGCAAGACACATCATC
GACATATGGTGAAGAGTTCGCCGAAATGCACGGAGGATATGGCTATTGTGACTTCACGTATGAGTGAGAGACACCTATCATTGATAA
GTGTTTTAAAGTAAATATAGAAATACGTATATGCAGCAAATACAAAAAATATTGTAAAGACGTTTCCCGCGCAATTGCGCGGGACAC
CTTGCTAGAATGCTAGTTTTCGCAAATAAAAAGAAGTCTATTTCGTACTCTCTCCGTTCCGAATTAACTAACGTCTTATACAAATCCAC
GTCCGTTAATACGGGACGGAGGGAGTACTTAGCGCAGTGGAAATTCTGTTCCCACCCAAGATAGCAAAGTGATCGAATTCGATTGGA
AGGAACTCCGTATTTAGAAACGGAGGGAATACACCAAAATTTATCTATCCGTAAACAGCGAATTGGCAGCGCGCGTGCACTGACTAT

CCGTAGACTCCAGTGTGGTCGATCGGGACGGCAATCCATTCCGATGGTGTATGATTGTTAGCGCAGGTGCCGTCGACACGCCGGAA
GCGCGCTCGGGCATACGTGGTACCACCCAGAGGAAACAGCCAAAAAAAACTTGAGACCGTGACCGTGAGAGCCACGGCTATATGCA
TGTTTCTTTCTCCCTGCCTTCAGGAACCCAGATTTCGTTTGCTGTGATCTCATTCATGACGTCCGCGACGGTGCCAGCGGCCGGCTCCG
TAGCTCGCCGTGGCTGCCAGCCAGAGGTCGAGGCCGTTCTTGGCGCCTTGCCAGACCGTTATATGGAAACGTCGACGCACGGCCATA
CAGGATCTTTCTTGTCTTTGTGTTACCTGATGTATTAAACCGTTCAACAATGTTGCCGCGCGTAGTGCCCGTTTTCTAAAAAGCAATGT
TCCAGGCAGTACAACATCAAGCTTAATAAACCACCAGCTAAAACAAAGCTTATAAACCACTGGAAATGCTTAATTGGGACTATTATCA
CCATGCACAAGATCAGTATACCGAATAATAATGCATGGAAGAAGCGCATATAGCACGCGCCAATCAGTCGCGCGCATGCAGGCAGC
ATGCCCACCTTGTTGGAATGCAGCAAAGTCCGAAAGGCCCCATGACCGAGTGGCGCCGCCGCAACCCCAACAAATTTACACGCCGTA
CAAAACAAAATCGCACCGATCTCGAGGCGCACACTCTATAATTCCACGTTCCGTGCCCCGCAGACACCAATAATTTCCGAGCACGGGC
AAACGTGCACGGCTGTACCCAGCTACATATACACCGTACCACCTGACGAGCTCAAGTGGCTCAAGGCTCGAGAAGCAGAGTGCGTTC
AACGTGGTATATAGTGAGAGAGAGACGGTGATCTTTGCTGAGCCAGCAGCCATGGCGGGGACGTCGTTGCCGGCGTCGGAGAGGC
CGCCGCACGCCGTGATGATCCCGTACCCGGCGCAGGGCCACGTGACGCCGATGCTGAAGCTCGCCAAGCTGCTCCACGCCCGGGGC
TTCCACGTCACCTTCGTCAACAACGAGTTCAACCAACGCCGCCTGCAGCGCGCGCAGGGCGGCGGTCCCGGCGCGCTCGACGGCGC
GCCCGGATTCCGCTTCGCCACCATCGACGACGGCCTCCCTCGCTCGGACCGCGACGCCCAGCAGGACGTCCCTTCGCTGTGCCGCTCC
ACCATGACCACCTGCCTCCCCAGGTTCAAGGCGCTCATCGCCAGGCTCAACGAAGACGCCGATGGCGCCGCGCCGCCCGTGACATGC
GTCGTCGGCGACAGCACCATGACCTTCGCCCTCCGCGCTGCTAAGGAGCTCGGCCTCCGCTGCGCCACGCTCTGGACCGCCAGCGCC
TGCGGCTTCATGGGGTACGCCCACTACAAGGACCTTGTCCAACGTGGTCTCTTCCCTCTCAAAGGTATATACGGGCATATGGCAGAAA
AAAATGAGTAAACAAAGAAATTACCCACTTGAGATCGATGATGAAAGTTCCGTTTTGGGTGCAGACGAGGCGCAGTTGAGCAATGG
GTACCTGGACACGACAGTGGACTGGATACCGGGGTTGCCGAAGGACCTGCGGCTGCGGGACTTGCCAAGCTTCGTGCGCAGCACCG
ACCCGGACGACATCATGTTCAACTTCTTCGTCCACGAGACGGCCGGCATGGCGCAGGCGTCCGGGGTGGTCATCAACACCTTCGACG
AGCTGGACGCGCCGCTGCTGGGCGCCATGTCGAAGCTCCTGCCGCCGGTCTACACCGTGGGGCCGCTCCATCTCACGGTGCGGAAC
AACGTGCCAGCGGAGAGCCCGGTGGCCGGCATCGACTCCAGCCTCTGGATTCAGCAGCAGGACGCGCCGCTCCGGTGGCTCGACGG
CCGGGCGCCGGGCTCCGTGGTGTACGTCAACTTCGGGAGCATCACGGTGATGTCGAACGAGCACCTGCTGGAGTTCGCGTGGGGAC
TGGCCAACACCGGCTACGCCTTCCTCTGGAACGTGCGGCCGGACCTCGTCCGGGGCGACGAGGCCGCCCTGCCGCCGGAGTTCTCCG
CGGCCACGGCGGGGCGGAGCATGCTGACGACGTGGTGCCCGCAGGAGAAGGTGCTGGAGCACGAGGCCGTGGGGGTGTTCCTCA
CGCACTCCGGCTGGAACTCCACGCTCGAGAGCATCTGCGGCGGGGTCCCCATGGTTTGCTGGCCGTTCTTCGCCGAGCAGCAGACCA
ACTGCCGGTTCAAGCGCACGGAGTGGGGAATTGGGGTGGAGGTGCCCGACGAGGTGAGGAGAGACGAGGTGGAGGCCATGATAC
GGGAGGCCATGGAGGGGGAGAAAGGCCGCGACATGCGGCGGCGCGTGCTGGAGCTCCGGGACAGCGCGCTGGCCTCGGCCAAGC
CTGGCGGCCGCTCCATGTGCAATGTTGATAGGCTCATTCAGGAAGTGTTGCTTGCTTGAAGCAGCCATTGTTGATGTCTTCTGATATA
AAACCTCATCTTGGGTTCGTGGCGTAGGCATGTGCAATTAATTGGTGCTTCTTCTAATGAGGAAATGCAACTGTTCACTCGCTGGTTTT
CAAAAGTAAATAGAAAATAAAAATAAAAATAAATAAACAACAGAAGAATGCACGGTCTCGACGTGCAGGCTGCAGCCATCTCCAGC
AAAGTGGCTTTTACTAACTGAAGCAATTTTCATTTTTTTTTACCAAGAAGCAATTTTCACTAATTCGGGAGGACGATCCATTTTTTCTTA
ATTATGCACTTAACGCTCTTTCCTTGGTACATACTCCATCTGTCCATCAATTTGCTCTTTTATCATATCACTCGATTTTTATTACTTCCTCC
GTTTCATAATTCTTGTCTCAAATTGTCCAAAAATGAATGTATCTATTTCTAAAAAGTATCTAAATACATGTAAGATTTCGACAAGAATTA
TGAAACGGAGGTAGTACTTTCGGTTGATAAACATGGACACTATGCTGTCTTGTGAATATGCAAGGTAGGTCTCAATTAACCTGCAGTA
AATATGCTCTTTTATCGTATCACTCGGCTCTTTTATCATATCACTCGATTCAACCACTGGCCATAAGCTAGCTTCATAAAAGAATTGAAT
TCTCCGAATTTATTACACCAACCATTCGATTGCAAATAATGTTGTAGTAGTTGTTCATGGTAGTACCAACTACGGACACTGTAAATTCA
AGTATGTACGTACACCGTAAATTCATCCTATTTGGTGAACAAGTATGCCGAGAGTACCTATATAAAAACAGTCCCGTCATGTTACACA
ACCGGTTGCATTCTCATCGTTCCAATGGAACTATAGTGGAGTAGTGAAAGCAAGGATAAGAGAATACAAGTAGCGTTCGTTCATGTT
GGAAATGAGTTTGTATTGGAAATGGAGGCATCGGAGTTCGTGCCACAGATCTTCCTCTCATCCCACCAGGCCACCACGGCCGATCTAT
GTGCAATGTTGATAGGCTCATTCAGGAAGTGTTGCTTGCTTGAAGTAGCCATTGTTGATGTCTTTTGATATAAGACCCCATCTTCGGTT
TGTGGCCAGGGCTGTGCAATTAGTTTGTGCTTCTTCTTCTAATGATGAAGAGTAATGCGGCTGTTCACTCGCTGTTTTTTTATTTCGAA
AAAGTAATTTTATTCTGAAGCTGCTATGCTCGACAAAACAGCAGAAGAATTCACGGCCTCAAGTCAGGCGCAGGCGTGCAGCCATCT
CCAGCAACAGCAAGTGTAACAGCCAGTGGCCTGTAGCCCTGTTTAACTGATTACCTGAAGCACTCTTCACTTGTCAGGAGGACGATCA
AGACTTTTCTAAATTCAGCAGTGTAACATATTAAGTACAAAATTTCCGGTTCAGTACCACGAAGGTTGCAGCTGCCATGATGTTCAAT
AGTACGTCTCTATGAATTTGCCCTCTTACAGTATCACTCTATTTTTATTACTTTCGGATGATAAACATGGACACTCAGCTATGTTGTGAA
TATGCAAGGTAGGTCTCAATTAACCTGTCATGCATCAAGGCCATTATTCAACCACTGGCCATAAGCTATCTTCATAAAAGAATTGAATT
CTCCCAATTTATTGTACCAACCATTCGATTGCAAATAATGTTGTCACAGTTGTTCATTATTGTACTGACTATGGACACTGTAAATTCATA
TGTGTACTGACACTGTAAATTCATCCGATTTTGTGAACAACTATGTCAGGAATACCTATATAAAAGCAGTCGAGTTCATGTTAAACAAC

CCGGTTGTAATCTCATCGTTCCAATGGAACTATAGTAGTGAAAGTAGAGGATATGAGAATACAAGTAGTGTTCGTTTATGTTGGCAAT
AAGTTTGTATCGGCAAAGGAGGCATCGGAAACTCAAGTTCGTGCCACAGATCTTCCACTCATCCGAGCACGGACCATGTTGCGAGAA
CCAAGATCAAAGGAAGGAATGGTAGCTGGATATAGCCAGGATACATTAATCTATTCTTCAGAATACGATGGACTGATAGCCAGAGAT
TCCAATTTCCGAGTTATTGAGGATGGATAGGTCGTGATGATGCTGGTTGCATTTGGAAGGGCGACGCTCCCGACCGTAACTAGCACA
CTGGCACCGTAGTAGTTTCGCTCAGAAAGTTCGTGCATCCTAGAGTTCATCAAGGAACATGCTGCATCTAGTATCATGACTCGTTTCG
TTCGGCAGGGGAAGCCGCCGGCAGCATCAAAACGTTAGGCATGCTGCTGCTCGTTTGCGTAGAATCATTCAGGGTTGTGGCATGTCC
GTCCGCAGTTCACTGGCAGCAATTGTAATCCCCCGGCTGCTGCAGAGAGCATACCACAAGAATCATTGGCCAGAAAAAAAGTTGGGG
GCGTTTGGACTGGAGCCGTTGGGGGCGCTTGGGACTCTCCCCCGCGTATCCTCGGCGATGTCTCCAGCGAGGATGTCAACCAGTTGG
TGTTGAGGGCTCTGCTCCGGCGACAAAAAATTCCAGCCTTCCAAAGCTTGTTCGTAGTTTCACACGATTAAACACAACCAATAATCAG
ACACATACAAAAGGGGCCTACCGAGCACAACACAGACATTGGGGAAAAAATATTCACCGAGTTGGGGGAAGGCATTCATTCAACAC
ACGGCACCGATTACAATGTTCAAGGAGAGCAATCACACTCTGGAACTGGTACACGAGTGGATATTTTAAGAAGCGATTTGCGGTTCC
TGATCCCAGGTACGATGCATAATTAGAAGCGGATAAATCCGGCTCCAGCTGCCTGCATCTCCAGGATCTGAGCGGCGGCTGTGGCGG
TGCTCCACTTTTCCTCTCTGATCTAACCGGCATGGGCGGCGACCAGTGGCGCGTCGCTGTCCGCTGCCTTTTCCTCCCTGATCTGAGCG
GCGGCGGCTGGTGCGTCGCCGTTCGCCATCTCGTTTCTGTCCACTTGGTGGCTCTGCTTCACGTCCTCGACTACGACGACCCTGATGC
GCCGATGCGCTTCGCATCTCCTTCCTCCTCGACCACACAGACACGACTGATACTCTGATAGGCCCCAAGATATTCCAAGGACGCTTTTT
GGCGCGATGCAACGGCATATCTTCGGCCTCCCTCCCTCTCTGCCCCTTCCGGCAGCGGCAGCCTTATCCTCTCCACCGCCCCAAACTCT
CACATCTGCTCTGCTTGGAGAAGCAGC

>BdiBd21-3.5G0143100

AGCTTCTTCGTGGAGCACGACGTTGGATTCGATGTTCAGCCAGGAGATCCAGATTAGGGTTCTGAGACTCGTGGCGGGTTTCTTTTCA
GCTTCCAGCCTCTTCAGGCCATGCTGTATTCTTGTCCGTACTCGGGCATAATTTGACTTCCGCTGATGTAATGATATAATGCTGGGACA
TGTGTCCTTTGTTTCTAATAATTCTTATTCTGCTTCTCGTACGAGCTTTGTAATTACTCTCTTCTGTAGAGTTTTGGTAATGATATTCATG
TTGCAAAACCCTGCGATGAGCACATTAATGCATGTATGTATTGAAGTGTTTATCGTAGGTTAAAAACCTGAGGCTTGAATTATGCAAA
GGTTAGCAACTTGGGGTTAAATTGCATAATTCCAGTCCGGGATCTTACAAAATGCCCCCCGAAAAAAAATATTTGGGAAATGTCGAA
GGCCTTGCCATCCCATTCGATGGACGTAATTTTCATCGACAAACAACCATATTTTGATTTTTATTTTTTTGGTCAGACACGCGTAGGTTA
TTATGTTGATGGAAACTGGCCTGGATAACATTGATTGGCTAGGCCATAGCGTCTTCGTGATAAACACAATCATGGAACGGGAGGTAC
GTCCAAATCTGGCTACAGTACCCGTTCAATGTCTGGTTTTGTGTCTATTATATATTAAAAGTAATATGAAGGGTACATGGAGCCTCCAC
GTTGATCTACATCATTCTAATTATTTTCAGCCGTTGGATATGAGATTAAGAGTCCACGTTTCAACACGTTATGTTGGGTTCCTAAAAAG
AACATGTTATGTTGGAAAAAAAAACTGCTAAAACAGACGGAACCATTGTTTCGTACCCAGGGATCGAATCCCACGCCTCCCAGGCTCC
CACGTACATTAGCTTTTTCTTTTTCTTTTTGAGCTGCATATACGTTAGCTTTTGGCCTTAGTTCTTTATATGGGATTCAATACCAGATCGA
TCCCGCTTCCCACAAATCAGTACAAAATTCCAATTGATCGAAACAACTAAATAAAATAACAAGCAATCTTCCCCGTAAAAAAAAACAA
TGTTTTTTTCTCTAAAATTCCAATTGATCGAAACAACTAAAATGACGATTATAATTTCGAATAAACCGAAAACTATGTCAAAAATGTTT
GAAGCAAACTTCTCTAAGTGCCTCATTCGCATCTTGATGTCTATTATATACTAAATAAAAGCAATATGATTTTCTCCAAATATCATGTAA
CAAAACATTAGAAGATCAAACATGCATAAGCTGGACTATCCTTTTAAGTTTTACTAAGCCGCATGAGCCGCCCATCCTAGCGGAAGAA
AACTTGAGATCGTTGACGCGCGGTGGCCAGACACACCCCTCACAAGAAAGGTTGCCATGCCATTGAATCGTCGCCGAACCAATGAGC
AAGAGGACAAAAGGCACGAAATCACGAGCCGTGAGATCCACAATCTTCATGTAATATACGATGATAGCCACACCAGAAAAAGGATG
GAGTTGGAAGACCATCAATCCATGCACCGTTGCTTCCACCGTAGACCGCCGCCGCTAAGAACACACAAAACCTTACAAAAAAAGGTA
CTACTAATACAATGGCCAGACGTTGATCCGTGATTTCCCACACCTCCAGGCGCCCAATAGGCTATAAAAGACGTGGGGAACCGATAA
AAATGGAAACCTGGTCAAGATTTAATGAGGATGACATATTTGATTTGATTGATGTGCTAGAAATAAACATCACGGGATGGCATATTT
GATTTCATTGATGGGTTAGAAATAAACATCGCGTAGGGCCATTACAAATTAATAATAGGGTGCAAGGTAGCTACCGGACGTGATTTG
CAACTAGAGCTATATGCTTACGTGAATCACGTCTGACATGATTTTCAAGATAATTTTTCTTTCTAAAAAATAAATTTACACGATCAATAA
CAATTTGTGTTTTAAATATGCATGCATTTTTTTCACGACCATGATTTATGCTAGGGAAACATTATGTTTGATCGATGAAAGGAAACATT
CTGATTGATAAATAAAAGTATATTTTCTTTACACTACCAGAAACAAAATTACGCAGTAGCAAAAACAATGTTTTGGTGTAATAAATAA
AGGTTCATCGATACAAATTCATTTGACGGGTGTTCTAGCAACCGTATTTATGTTTGTGAAAGGAAATTGTCATATATTAAAGCAACTA
GCTCTTACAGTACTATATTCAATTGACCAAAAATAAAATTACACATGAAGAGTTCGCCGAAATGCATGGAGGATATGGCTATTGTGAC
TTCACACATGAGTGAGATCATCTATCATTGATAACTACCACCCCTCCTACCTGGCAAATGGGGGAGAAGGGGACCTTCACAGCTCCTG
CGGGAAGCAGTGGAGCGGAAATGCTTTTTTTAAATAGAAGCGGTTTCTGGAGATAGATATCTTTTTATTTTGATGAGTTATGTAAACC
TTATGCAAGACACATCATCGACATATGGTGAAGAGTTCGCCGAAATGCACGGAGGATATGGCTATTGTGACTTCACGTATGAGTGAG
AGACACCTATCATTGATAAGTGTTTTTAAAGTAAATATAGAAATACGTATATGCAGCAAATACAAAAAATATTGTAAAGACGTTTCCC

GCGCAATTGCGCGGGACACCTTGCTAGAATGCTAGTTTTCGCAAATAAAAAGAAGTCTATTTCGTACTCTCTCCGTTCCGAATTAACT
GACGTCTTATACAAATCCACGTCCGTTAATACGGGACGGAGGGAGTACTTAGCGCAGTGGAAATTCTGTTCCCACCCAAGATAGCAA
AGTGATCGAATTCGATTGGAAGGAACTCCGTATTTAGAAACGGAGGGAATACACCAAAATTTATCTGTCCGTAAACAGCGAATTGGC
AGCGCGCGTGCACGGACTATGCGTAGACTCCAGTGTGGTCGATCGGGACGGCAATCCATTCCGATGGTGTATGATTGTTAGCGCAG
GTGCCGTCGAGACGCCGGAAGCGCGCTCGTGCATACGTGGTACCACCCAGAGGAAACAGCCAAAAAAAACTTGAGACCGTGGCCGT
GAGAGCCACGGCAATATGCATGTTTCTTTCTCCCTGCCTTCAGGAACCCAGATTTCGTTTGCTGTGATCTCATTCATGACGTCCGCGAC
GGTGCCAGCGGCCGGCTCCGTAGCTCGCCGTGCCTGCCAGCCAGAGGTCGAGGCCGTTCTTGGCGCCTTGCCAGACCGTTATATGGA
AACGTCGACGCACGGCCATACAGGATCTTTCTTGTCTTTGTGTTACCTGATGTATTAAACCGTTCAACAATGTTGCCGCGCGTAGTGCC
CGTTTTCTAAAAAGCAATGTTCCAGGCAGTACAACATCAAGCTTAATAAACCACCAGCTAAAACAAAGCTTATAAACCACTGGAAATG
CTTAATTGGGACTATTATCACCATGCACAAGATCAGTATACCGAATAATAATGCATGGAAGAAGCGCATATAGCACGCGCCAATCAG
TCGCGCACATGCAGGCAGCATGCCCACCTTGTTGGAATGCAGCAAAGTCCGAAAGGCCCCATGACCGAGTGGCGCCGCCGCAACCC
CAACAAATTTACACGCCGTACAAAACAAAATCGCACCGATCTCGAGGCGCACACTCTATAATTCCACGTTCCGTGCCCCGCAGACACC
AATAATTTCCGAGCACGGGCTAACGTGCACGGCTGTACCCAGCTACATATACACCGTACCACCTGACGAGCTCAAGTGGCTCAAGGC
TCGAGAAGCAGAGTGCGTTCAACGTGGTATATAGTGAGAGAGAGACGGTGATCTTTGCTGAGCCAGCAGCCATGGCGGGGACGTC
GTTGCCGGCGTCGGAGAGGCCGCCGCACGCCGTGATGATCCCGTACCCGGCGCAGGGCCACGTGACGCCGATGCTGAAGCTCGCCA
AGCTGCTCCACGCCCGGGGCTTCCACGTCACCTTCGTCAACAACGAGTTCAACCACCGCCGCCTGCAGCGCGCGCAGGGCGGCGGTC
CCGGCGCGCTCGACGGCGCGCCCGGATTCCGCTTCGCCACCATCGACGACGGCCTCCCTCGCTCGGACCGCGACGCCCAGCAGGAC
GTCCCTTCGCTGTGCCGCTCCACCATGACCACCTGCCTCCCCAGGTTCAAGGCGCTCATCGCCAGGCTCAACGAAGACGCCGATGGCG
CCGCGCCGCCCGTGACATGCGTCGTCGGCGACAGCACCATGACCTTCGCCCTCCGCGCTGCTAAGGAGCTCGGCCTCCGCTGCGCCA
CGCTCTGGACCGCCAGCGCCTGCGGCTTCATGGGGTACGCCCACTACAAGGACCTTGTCCAACGTGGTCTCTTCCCTCTCAAAGGTAT
ATACGGGCATATGGCAGAAAAAAATGAGTAAACAAAGAAATTACCCACTTGAGATCGATGATGAAGTTCCGTTTTGGGTGCAGACG
AGGCGCAGTTGAGCAATGGGTACCTGGACACGACAGTGGACTGGATACCCGGGTTGCCGAAGGACCTGCGGCTGCGGGACTTGCC
AAGCTTCGTGCGCAGCACCGACCCGGACGACATCATGTTCAACTTCTTCGTCCACGAGACGGCCGGCATGGCGCAGGCGTCCGGGGT
GGTCATCAACACCTTCGACGAGCTGGACGCGCCGCTGCTGGGCGCCATGTCGAAGCTCCTGCCGCCGGTCTACACCGTGGGGCCGCT
CCATCTCACGGTGCGGAACAACGTGCCAGCGGAGAGCCCGGTGGCCGGCATCGACTCCAGCCTCTGGATTCAGCAGCAGGACGCGC
CGCTCCGGTGGCTCGACGGCCGGGCGCCGGGCTCCGTGGTGTACGTCAACTTCGGGAGCATCACGGTGATGTCGAACGAGCACCTG
CTGGAGTTCGCGTGGGGACTGGCCAACACCGGCTACGCCTTCCTCTGGAACGTGCGGCCGGACCTCGTCCGGGGCGACGAGGCCGC
CCTGCCGCCGGAGTTCTCCGCGGCCACGGCGGGGCGGAGCATGCTGACGACGTGGTGCCCGCAGGAGAAGGTGCTGGAGCACGAG
GCCGTGGGGGTGTTCCTCACGCACTCCGGCTGGAACTCCACGCTCGAGAGCATCTGCGGCGGGGTCCCCATGGTTTGCTGGCCGTTC
TTCGCCGAGCAGCAGACCAACTGCCGGTTCAAGCGCACGGAGTGGGGAATTGGGGTGGAGGTGCCCGACGAGGTGAGGAGAGAC
GAGGTGGAGGCCATGATACGGGAGGCCATGGAGGGGGAGAAAGGCCGCGACATGCGGCGGCGCGTGCTGGAGCTCCGGGACAG
CGCGCTGGCCTCGGCCAAGCCTGGCGGCCGCTCCATGTGCAATGTTGATAGGCTCATTCAGGAAGTGTTGCTTGCTTGAAGCAGCCA
TTGTTGATGTCTTCTGATATAAAACCTCATCTTGGGTTCGTGGCGTAGGCATGTGCAATTAATTGGTGCTTCTTCTAATGAGGAAATGC
AACTGTTCACTCGCTGGTTTTCAAAAGTAAATAGAAAATAAAAATAAAAAATAAACAACAGAAGAATGCACGGTCTCGACGTGCAGG
CTGCAGCCATCTCCAGCAATTTTCATTTTTTTTTACCAAGAAGCAATTTTCACTAATTCGGGAGGACGATCCATTTTTTCTTAATTATGC
ACTTAACGCTCTTTCCTTGGTACATACTCCATCTGTCCATCAATTTGCTCTTTTATCGTATCACTCGATTTTTATTACTCCCTCCGTTTCAT
AATTCTTGTCTCAAATTGTCTAAAAATAAATGTATCTATTTCTAAAAAGTATCTAGATACATGTAAGATTTCGACAAGAATTATAAAAC
GGAGGTAGTACTTTCGGTTGATAAACATGGACACTATGCTGTCTTGTGAATATGCAAGGTAGGTCTCAATTAACCTGCAGTAAATATG
CTCTTTTATCGTATCACTCGGCTCTTTTATCATATCACTCGATTCAACCACTGGCCATAAGCTAGCTTCATAAAAGAATTGAATTCTCCG
AATTTATTACACCAACCATTCGATTGCAAATAATGTTGTAGTAGTTGTTCATGGTAGTACCAACTACGGACACTGTAAATTCAAGTATG
TACGTACACCGTAAATTCATCCTATTTGGTGAACAATTATGCCGAGAGTACCTATATAAAAACAGTCCCGTCATGTTACACAACCGGTT
GCATTCTCATCGTTCCAATGGAACTATAGTGGAGTAGTGAAAGCAAGGATAAGAGAATACAAGTAGCGTTGGTTCATGTTGGCAATA
AGTTTGTATTGGAAATGGAGGCATCGGAAACTCAAGTTCGTGCCACAGATCTTCCTCTCATCCCACCAGGCCACCACGGCCGATCTAT
GTGCAATGTTGATAGGCTCATTCAGGAAGTGTTACTTGCTTGAAGCAGCCATTGTTGATGTCTTCTGATATAAGACCCCATCTTGGGTT
CGTGGCCTGGGCTGTGCAATTAGTTTTTGCTTCTTCTTCTAATGATGAAGAGTAATGCGGCTGTTCACTCGCTGTTTTTTTATTTCAAAA
AAGTAATTTTATTCTGAAACTGCTATGCTCGACAAAACAGCAGAAGAATTCACGGCCTCAAGTCAGGCGCAGGCGTGCAGCCATCTC
CAACAACAGCAAGTGTAACAGCCAGTGGCCTGTAGCCTTGTTTAACTGATTACCTGAAGCACTCTTCACTTGTCAGGAGGACGATCAA
GACTTTTCTAAATTCAGCAGTGTAACATATTAAGTACAAAATTTCCGGTTCAGTACCACGAAGGTTGCAGCTGCCATGATGTTCAATA
GTACGTCTCTATGAATTTGCCCTCTTACAGTATCACTCTATTTTTATTACTTTCGGATGATAAACATGGACACTCAGCTATGTTGTGAAT

ATGCAAGGTAGGTCTCAATTAACCTGTCATGCATCAAGGCCATTATTCAACCACTGGCCATAAGCTATCTTCATAAAAGAATTGAATTC
TCCCAATTTATTGTACCAACCATTCGATTGCAAATAATGTTGTCACAGTTGTTCATTATTGTACTGACTATGGACAATGTAAATTCATAT
ATGTACTGACACTGTAAATTCATCCGATTTTGTGAACAACTATGTCAGGAATACCTATATAAAAGCAGTCGAGTTCATGTTAAACAACC
CGGTTGTAATCTCATCGTTCCAATGAAACTATAGTAGTGAAAGTAGAGGATATGAGAATACAAGTAGTGTTCGTTTATGTTGGCAATA
AATTTGTATCGGCAAAGGAGGCATCGGAAACTCAAGTTCGTGCCACAGATCTTCCACTCATCCGAGCACGGACCATGTTGCTAGAAC
CAAGATCAAAGGAAGGAATGGTAGCTGGATATAGCCAGGATACATTAATCTATTCTTCAGAATACGATGGACTGATAGCCAGAGATT
CCAATTTCCGAGTTATTGAGGATGGATAGGTCGTGATGATGCTGGTTGCATTTGGAAGGGCGACGCTCCCGACCGTAACTAGCACAC
TGGCACCGTAGTAGTTTCGCTCAGAAAGTTCGTGCATCCTAGAGTTCATCAAGGAACATGCTGCATCTAGTATCATGACTCGTTTCGTT
CGGCAGGGGAAGCCGCCGGCAGCACCAAAACGTTAGGCATGCTGCTGCTCGTTTGCGTAGAATCATTCAGGGTTGTGGCATGTCCG
TCCGCAGTTCACTGGCAGCAATTGTAATCCCCCGGCTGCTGCAGAGAGCATACCACAAGAATCATTGGCCAGAAAAAGAGTTGGGG
GCGTTTGGACTGGAGCCGTTGGGGGCGCTTGGGACTCTCCCCCGCGTATCCTCGGCGATGTCTCCAGCGAGGATGTCAACCAGTTGG
TGTTGAGGGCTCTGCTCCGGCGACAAAAAATTCCAGCCTTCCAAAGCTTGTTCGTAGTTTCACACGATTAAACACAACCAATAATCAG
ACACATACAAAAGGGGCCTACCGAGCACAACACAGACATTGGGGAAAAAATATTCACCGAGTTGGGGGAATGCATTCATTCAACAC
ACGGCACCGATTACAATGTTCAAGGAGAGCAATCACACTCTGGAACTGGTACACGAGTAGATATTTTAAGAAGCGATTTGCGGTTCC
TGATCCCAGGTACCATGCATAATTAGAAGCGGATAAATCCGGCTCCAGCTGCCTGCATCTCCAGGATCTGAGCGGCGGCTGTGGCGG
TGCTCCACTTTTCCTCTCTGATCTAACCGGCATGGGCGGCGACCAGTGGCGCGTCGCTGTCCGCTGCCTTTTCCTCCCTGATCTGAGCG
GCGGCGGCTGGTGCGTCGCCGTTCGCCATCTCGTTTCTGTCCACTTGGTGGCTCTGCTTCACGTCCTCGACTACGACGACCCTGATGC
GCCGATGCGCTTCGCATCTCCTTCCTCCTCGACCACACAGACACGACTGATACTCTGATAGGCCCCAAGATATTCCAAGGACGCTTTTT
GGCGCGATGCAACGGCATATCTTCGGCCTCCCTCCCTCTCTGCCCCTTCCGGCAGCGGCAGCCTTATCCTCTCCACCGCCCCAAACTCT
CACATCTGCTCTGCTTGGAGAAGCAGC

Alignment of Sequence_1: [Untitled Sequence #1] with Sequence_2: [Sequence Window #2]

Similarity : 7918/8786 (90.12 %)

```
Seq_1  1      ------------------------------------------------------------  0

Seq_2  1      AGCTTCTTCGTGGAGCACGACGTTGGATTCGATGTTCAGCCAGGAGATCCAGATTAGGGT  60


Seq_1  1      ------------------------------------------------------------  0

Seq_2  61     TCTGAGACTCGTGGCGGGTTTCTTTTCAGCTTCCAGCCTCTTCAGGCCATGCTGTATTCT  120


Seq_1  1      ------------------------------------------------------------  0

Seq_2  121    TGTCCGTACTCGGGCATAATTTGACTTCCGCTGATGTAATGATATAATGCTGGGACATGT  180


Seq_1  1      ------------------------------------------------------------  0

Seq_2  181    GTCCTTTGTTTCTAATAATTCTTATTCTGCTTCTCGTACGAGCTTTGTAATTACTCTCTT  240


Seq_1  1      ------------------------------------------------------------  0

Seq_2  241    CTGTAGAGTTTTGGTAATGATATTCATGTTGCAAAACCCTGCGATGAGCACATTAATGCA  300


Seq_1  1      ------------------------------------------------------------  0

Seq_2  301    TGTATGTATTGAAGTGTTTATCGTAGGTTAAAAACCTGAGGCTTGAATTATGCAAAGGTT  360


Seq_1  1      ------------------------------------------------------------  0

Seq_2  361    AGCAACTTGGGGTTAAATTGCATAATTCCAGTCCGGGATCTTACAAAATGCCCCCCGAAA  420


Seq_1  1      ------------------------------------------------------------  0
```

```
Seq_2  421   AAAAATATTTGGGAAATGTCGAAGGCCTTGCCATCCCATTCGATGGACGTAATTTTCATC  480


Seq_1  1     ------------------------TATTTTTTTGGTCAGACACGCGTAGGTTATTATGTT  36
                                     ||||||||||||||||||||||||||||||||||||
Seq_2  481   GACAAACAACCATATTTTGATTTTTATTTTTTTGGTCAGACACGCGTAGGTTATTATGTT  540


Seq_1  37    GATGGAAACTGGCCTGGATAACATTGATTGGCTAGGCCATAGCGTCTTCGTGATAGACAC  96
             ||||||||||||||||||||||||||||||||||||||||||||||||||||||| ||||
Seq_2  541   GATGGAAACTGGCCTGGATAACATTGATTGGCTAGGCCATAGCGTCTTCGTGATAAACAC  600


Seq_1  97    AATTATGGAACGGGAGGTCCGTCCAAATCTGGCTACAGTACCCATTCAATGTCTGGTTTT  156
             ||| |||||||||||||| |||||||||||||||||||||||| ||||||||||||||||
Seq_2  601   AATCATGGAACGGGAGGTACGTCCAAATCTGGCTACAGTACCCGTTCAATGTCTGGTTTT  660


Seq_1  157   GTGTCTATTATATACTTAAAGCAATATGAAGGATACGTGGAGCCTCCACGTTGATCTACA  216
             |||||||||||||| | |||| |||||||||| ||| |||||||||||||||||||||||
Seq_2  661   GTGTCTATTATATATTAAAAGTAATATGAAGGGTACATGGAGCCTCCACGTTGATCTACA  720


Seq_1  217   TCATTCTAATTATTTTCA-CCGTTGGATATGAGATTAAGAGTCCACGTTTCAACACGTTA  275
             |||||||||||||||||| |||||||||||||||||||||||||||||||||||||||||
Seq_2  721   TCATTCTAATTATTTTCAGCCGTTGGATATGAGATTAAGAGTCCACGTTTCAACACGTTA  780


Seq_1  276   TGTTGGGTTCCTAAAAAGAACATGTTATGTTGGAAAAAAAAACTGCTAAAACAGACAGAA  335
             |||||||||||||||||||||||||||||||||||||||||||||||||||||||| |||
Seq_2  781   TGTTGGGTTCCTAAAAAGAACATGTTATGTTGGAAAAAAAAACTGCTAAAACAGACGGAA  840


Seq_1  336   CCATTGTTACGTACCCAGAGATCGAATCCCACGCCTCCCAGGCTCCCACGTACATTAGCT  395
             |||||||| ||||||||| |||||||||||||||||||||||||||||||||||||||||
Seq_2  841   CCATTGTTTCGTACCCAGGGATCGAATCCCACGCCTCCCAGGCTCCCACGTACATTAGCT  900


Seq_1  396   TTTTCTTTTTCTTTTTGAGCTGCATATACGTTAGCTTTTGGCCTTAGTTCTTTATATGGG  455
             ||||||||||||||||||||||||||||||||||||||||||||||||||||||||||||
Seq_2  901   TTTTCTTTTTCTTTTTGAGCTGCATATACGTTAGCTTTTGGCCTTAGTTCTTTATATGGG  960


Seq_1  456   ATTCAATACCAGATCGATCCCGCTTCCCACAAATCAGTACAAAATTCCAATTGATCGAAA  515
             ||||||||||||||||||||||||||||||||||||||||||||||||||||||||||||
Seq_2  961   ATTCAATACCAGATCGATCCCGCTTCCCACAAATCAGTACAAAATTCCAATTGATCGAAA  1020


Seq_1  516   CAACTAAATAAAATAACAAGCAATCTTCCCCGTAAAAAAAA-CAATGTTTTTTTCTCTAA  574
             ||||||||||||||||||||||||||||||||||||||||| ||||||||||||||||||
Seq_2  1021  CAACTAAATAAAATAACAAGCAATCTTCCCCGTAAAAAAAAACAATGTTTTTTTCTCTAA  1080


Seq_1  575   AATTCCAATTGATCGAAACAACTAAAATGACGACTATAATTTTGAATAAACCGAAAACTA  634
             ||||||||||||||||||||||||||||||||| |||||||| |||||||||||||||||
Seq_2  1081  AATTCCAATTGATCGAAACAACTAAAATGACGATTATAATTTCGAATAAACCGAAAACTA  1140


Seq_1  635   TGTCAAAAATGTTTGAAGCAAACTTCTCTAACTGCCTCATTCGCATCTTGATATCTATTA  694
             ||||||||||||||||||||||||||||||| |||||||||||||||||||| |||||||
Seq_2  1141  TGTCAAAAATGTTTGAAGCAAACTTCTCTAAGTGCCTCATTCGCATCTTGATGTCTATTA  1200


Seq_1  695   TATACTAAATAAAAGCAATATGATTTTCTCCAAATATCATGTAACAGAACGTTAGAAGAT  754
             |||||||||||||||||||||||||||||||||||||||||||||| ||| |||||||||
Seq_2  1201  TATACTAAATAAAAGCAATATGATTTTCTCCAAATATCATGTAACAAAACATTAGAAGAT  1260


Seq_1  755   CAAACATGCATAAGCTGGACTATCCTTTTAAGTTTTACTAAGCCGCATGAGCCGGCCACC  814
             |||||||||||||||||||||||||||||||||||||||||||||||||||||| ||| |
```

```
Seq_2  1261  CAAACATGCATAAGCTGGACTATCCTTTTAAGTTTTACTAAGCCGCATGAGCCGCCCATC  1320

Seq_1  815   CTAGCGGAAGAAAACTTGAGATCGTTGACGCGCGGTGGCCAGACACACCCCTCACAAGAA  874
             ||||||||||||||||||||||||||||||||||||||||||||||||||||||||||||
Seq_2  1321  CTAGCGGAAGAAAACTTGAGATCGTTGACGCGCGGTGGCCAGACACACCCCTCACAAGAA  1380

Seq_1  875   AGGTTGCCATGCCATTGAATCGTCGCCGAACCAATGAGCAAGAGGACAAAAGGCACGAAA  934
             ||||||||||||||||||||||||||||||||||||||||||||||||||||||||||||
Seq_2  1381  AGGTTGCCATGCCATTGAATCGTCGCCGAACCAATGAGCAAGAGGACAAAAGGCACGAAA  1440

Seq_1  935   TCACGAACCGTGAGATCCACAATCTTCATGTAATATATGATGATAGCCACACCAGAAAAA  994
             |||||| |||||||||||||||||||||||||||||| ||||||||||||||||||||||
Seq_2  1441  TCACGAGCCGTGAGATCCACAATCTTCATGTAATATACGATGATAGCCACACCAGAAAAA  1500

Seq_1  995   GGATGGAGTTGGAAGACCATCAATCCATGCACCGTTGTTTCCACCGTAGACCGCCGCCGC  1054
             ||||||||||||||||||||||||||||||||||||| ||||||||||||||||||||||
Seq_2  1501  GGATGGAGTTGGAAGACCATCAATCCATGCACCGTTGCTTCCACCGTAGACCGCCGCCGC  1560

Seq_1  1055  TAAGAACACACAAAACCTTACAAAAAAAGGTACTACTAATACAATGGCCAGACGTTGATC  1114
             ||||||||||||||||||||||||||||||||||||||||||||||||||||||||||||
Seq_2  1561  TAAGAACACACAAAACCTTACAAAAAAAGGTACTACTAATACAATGGCCAGACGTTGATC  1620

Seq_1  1115  CGTGATTTCCCACACCTCCAGGCGCCCAATAGGCTATAAAAGACGTGGGGAACCGATAAA  1174
             ||||||||||||||||||||||||||||||||||||||||||||||||||||||||||||
Seq_2  1621  CGTGATTTCCCACACCTCCAGGCGCCCAATAGGCTATAAAAGACGTGGGGAACCGATAAA  1680

Seq_1  1175  AATGGAAACCTGGTCAAGATTTAATGAGGATGACATATTTGATTTGATTGATGTGCTAGA  1234
             ||||||||||||||||||||||||||||||||||||||||||||||||||||||||||||
Seq_2  1681  AATGGAAACCTGGTCAAGATTTAATGAGGATGACATATTTGATTTGATTGATGTGCTAGA  1740

Seq_1  1235  AATAAACATCACGGGATGGCATATTTGATTTCATTGATGAGTTAGAAATAAACATCGCGT  1294
             ||||||||||||||||||||||||||||||||||||||| ||||||||||||||||||||
Seq_2  1741  AATAAACATCACGGGATGGCATATTTGATTTCATTGATGGGTTAGAAATAAACATCGCGT  1800

Seq_1  1295  AGGGCCATTACAAATTAATAAAAGGGTGCAAGGTAGCTGCCGGACGTGATTTGCAACTAG  1354
             ||||||||||||||||||||| |||||||||||||||| |||||||||||||||||||||
Seq_2  1801  AGGGCCATTACAAATTAATAATAGGGTGCAAGGTAGCTACCGGACGTGATTTGCAACTAG  1860

Seq_1  1355  AGCTATATGCTTACGTGAATCACGTCTGACATGATTTTCAAGATAATTTTTCTTTCTAAA  1414
             ||||||||||||||||||||||||||||||||||||||||||||||||||||||||||||
Seq_2  1861  AGCTATATGCTTACGTGAATCACGTCTGACATGATTTTCAAGATAATTTTTCTTTCTAAA  1920

Seq_1  1415  AAATAAATTTACACGATCAATAACAATTTGTGTTTTAAATATGCATGCATTTTTTTCACG  1474
             ||||||||||||||||||||||||||||||||||||||||||||||||||||||||||||
Seq_2  1921  AAATAAATTTACACGATCAATAACAATTTGTGTTTTAAATATGCATGCATTTTTTTCACG  1980

Seq_1  1475  ACCATGATTTATGCTAGGGAAACATTATGTTTGATCGATGAAAGGAAACATTCTGATTGA  1534
             ||||||||||||||||||||||||||||||||||||||||||||||||||||||||||||
Seq_2  1981  ACCATGATTTATGCTAGGGAAACATTATGTTTGATCGATGAAAGGAAACATTCTGATTGA  2040

Seq_1  1535  TAAATAAAAGTATATTTTCTTTACACTACCAGAAACAAAATTACGCAGTAGCGAAAACAA  1594
             |||||||||||||||||||||||||||||||||||||||||||||||||||| |||||||
Seq_2  2041  TAAATAAAAGTATATTTTCTTTACACTACCAGAAACAAAATTACGCAGTAGCAAAAACAA  2100

Seq_1  1595  TGTTTTGGTGTAATAAATAAAGGTTCATCGATACAAATTCATTTGACGGGTGTTCTAGCA  1654
             ||||||||||||||||||||||||||||||||||||||||||||||||||||||||||||
```

```
Seq_2  2101  TGTTTTGGTGTAATAAATAAAGGTTCATCGATACAAATTCATTTGACGGGTGTTCTAGCA  2160

Seq_1  1655  ACCGTATGTATGTTTGCGAAAGGAAATTGTCATATATTAAAGCAACTAGCTCTTACAGTA  1714
             ||||||| |||||||| |||||||||||||||||||||||||||||||||||||||||||
Seq_2  2161  ACCGTATTTATGTTTGTGAAAGGAAATTGTCATATATTAAAGCAACTAGCTCTTACAGTA  2220

Seq_1  1715  CTATATTCAATTGACCAAAAATAAAATTACACAACTCCTTGAGGAAAAATGACTTCGGAC  1774
             |||||||||||||||||||||||||||||||||
Seq_2  2221  CTATATTCAATTGACCAAAAATAAAATTACACA---------------------------  2253

Seq_1  1775  TTCCAAACCACGTCGAAGCTATGACAATGTTTTTGACGTACGAGCCGGGATATAACCGAT  1834

Seq_2  2254  ------------------------------------------------------------  2253

Seq_1  1835  TTGAGCCAGAAGACACCCGTGATATTGATCTGAGAAGCAGACCATCATTCCAAAAGGCGA  1894

Seq_2  2254  ------------------------------------------------------------  2253

Seq_1  1895  CGACGATGGTTGGATGACCATTGTAAATTCAGCCAATCAGATCAGTCGAGACCTAACCTT  1954

Seq_2  2254  ------------------------------------------------------------  2253

Seq_1  1955  ACTAGCTCCGAGGCGAAACTGAAGTTTGACAAACCTCGCCAATCAGAAAAAATGCGCACA  2014

Seq_2  2254  ------------------------------------------------------------  2253

Seq_1  2015  TACAGAACACGAACACACGGAAACGAATCGACTATTTCATAAGAGTCGCCATCAACGAGA  2074

Seq_2  2254  ------------------------------------------------------------  2253

Seq_1  2075  TCGTCACTGATGAGGTGGATCATTAGTCGAGAAAACCTAATTCTTAAACCGCACGGCGCC  2134

Seq_2  2254  ------------------------------------------------------------  2253

Seq_1  2135  ACAATATCCACCATAGCGTCACAGCCGAGGAACCAAACCTAACTCTAAGAAGCCTAACTA  2194
               || |     |  |   |    |  |   |     |       ||    |  |
Seq_2  2254  TGAAGAGTTCGCCGAAA-TGCATGGAGGATATGGCTATTGTGA-CTTC--ACACATGAGT  2309

Seq_1  2195  CCAGCCCTCAAACTGGTCGGAGTCCCACCCCTCCTGCTGTAGCGGCAAACAGGGAGGAGG  2254
               ||  |    |    |  ||   | ||| | ||| ||     |  ||   ||||| |||
Seq_2  2310  G-AGATCATCTATCA-TT-GATAACTACCACCCCTCCTACCTGGCAAATGGGGGAGAAGG  2366

Seq_1  2255  GGACGTCCGCAACGCTTGCGGGAAGCAGTGGTGCGAAAATGCTGTTTTGGAACAGAAGCG  2314
             |||| | | || | | ||||||||||||||| ||| ||||||| ||||  || ||||||
Seq_2  2367  GGACCTTCACAGCTCCTGCGGGAAGCAGTGGAGCGGAAATGCTTTTTTTAAATAGAAGC-  2425

Seq_1  2315  GATAGGGTTTCTGGAGATAGTACC-TTTTTATTTTGACGAGTTATGAAAACCTTACGCAA  2373
                  |||||||||||||||      | || || |   ||    | |
Seq_2  2426  -----GGTTTCTGGAGATAG---ATATCTTTTTATTTTGATGAGTTATGTAAACCTTATG  2477

Seq_1  2374  GACGTCATGTCAACGACATATGATGAAGAGTTCGCCGAAATGCATGGAGGATATGGCTAT  2433
              | | ||  ||| ||||||||| ||||||||||||||||||||| |||||||||||||||
Seq_2  2478  CAAGACACATCATCGACATATGGTGAAGAGTTCGCCGAAATGCACGGAGGATATGGCTAT  2537

Seq_1  2434  TGTGACTTCACACATGAGTGAGATCATCTATCATTGATAACTACCACCCCTCCTACCTGG  2493
             |||||||||||
```

```
Seq_2  2538  TGTGACTTCAC-------------------------------------------------  2548

Seq_1  2494  CAAATGGGGGAGAAGGGGACCTTCACAGCTCCTGCGGGAAGCAGTGGAGCGGAAATGCTT  2553

Seq_2  2549  ------------------------------------------------------------  2548

Seq_1  2554  TTTTTGAATAGAAGCGGTTTCTGGAGATAGATATCTTTTTATTTTGATGAGTTATGTAAA  2613

Seq_2  2549  ------------------------------------------------------------  2548

Seq_1  2614  TCTTATGCAAGACACATCATCGACATATGGTGAAGAGTTCGCCGAAATGCACGGAGGATA  2673

Seq_2  2549  ------------------------------------------------------------  2548

Seq_1  2674  TGGCTATTGTGACTTCACGTATGAGTGAGAGACACCTATCATTGATAAGTGTTTT-AAAG  2732
                               ||||||||||||||||||||||||||||||||||||| ||||
Seq_2  2549  ------------------GTATGAGTGAGAGACACCTATCATTGATAAGTGTTTTTAAAG  2590

Seq_1  2733  TAAATATAGAAATACGTATATGCAGCAAATACAAAAAATATTGTAAAGACGTTTCCCGCG  2792
             ||||||||||||||||||||||||||||||||||||||||||||||||||||||||||||
Seq_2  2591  TAAATATAGAAATACGTATATGCAGCAAATACAAAAAATATTGTAAAGACGTTTCCCGCG  2650

Seq_1  2793  CAATTGCGCGGGACACCTTGCTAGAATGCTAGTTTTCGCAAATAAAAAGAAGTCTATTTC  2852
             ||||||||||||||||||||||||||||||||||||||||||||||||||||||||||||
Seq_2  2651  CAATTGCGCGGGACACCTTGCTAGAATGCTAGTTTTCGCAAATAAAAAGAAGTCTATTTC  2710

Seq_1  2853  GTACTCTCTCCGTTCCGAATTAACTAACGTCTTATACAAATCCACGTCCGTTAATACGGG  2912
             ||||||||||||||||||||||||| ||||||||||||||||||||||||||||||||||
Seq_2  2711  GTACTCTCTCCGTTCCGAATTAACTGACGTCTTATACAAATCCACGTCCGTTAATACGGG  2770

Seq_1  2913  ACGGAGGGAGTACTTAGCGCAGTGGAAATTCTGTTCCCACCCAAGATAGCAAAGTGATCG  2972
             ||||||||||||||||||||||||||||||||||||||||||||||||||||||||||||
Seq_2  2771  ACGGAGGGAGTACTTAGCGCAGTGGAAATTCTGTTCCCACCCAAGATAGCAAAGTGATCG  2830

Seq_1  2973  AATTCGATTGGAAGGAACTCCGTATTTAGAAACGGAGGGAATACACCAAAATTTATCTAT  3032
             |||||||||||||||||||||||||||||||||||||||||||||||||||||||||| |
Seq_2  2831  AATTCGATTGGAAGGAACTCCGTATTTAGAAACGGAGGGAATACACCAAAATTTATCTGT  2890

Seq_1  3033  CCGTAAACAGCGAATTGGCAGCGCGTGCACTGACTATCCGTAGACTCCAGTGTGGTCG  3092
             |||||||||||||||||||||||||||||| |||||| ||||||||||||||||||||||
Seq_2  2891  CCGTAAACAGCGAATTGGCAGCGCGTGCACGGACTATGCGTAGACTCCAGTGTGGTCG  2950

Seq_1  3093  ATCGGGACGGCAATCCATTCCGATGGTGTATGATTGTTAGCGCAGGTGCCGTCGACACGC  3152
             ||||||||||||||||||||||||||||||||||||||||||||||||||||||| ||||
Seq_2  2951  ATCGGGACGGCAATCCATTCCGATGGTGTATGATTGTTAGCGCAGGTGCCGTCGAGACGC  3010

Seq_1  3153  CGGAAGCGCGCTCGGGCATACGTGGTACCACCCAGAGGAAACAGCCAAAAAAAACTTGAG  3212
             |||||||||||||| |||||||||||||||||||||||||||||||||||||||||||||
Seq_2  3011  CGGAAGCGCGCTCGTGCATACGTGGTACCACCCAGAGGAAACAGCCAAAAAAAACTTGAG  3070

Seq_1  3213  ACCGTGACCGTGAGAGCCACGGCTATATGCATGTTTCTTTCTCCCTGCCTTCAGGAACCC  3272
             |||||| |||||||||||||||| ||||||||||||||||||||||||||||||||||||
Seq_2  3071  ACCGTGGCCGTGAGAGCCACGGCAATATGCATGTTTCTTTCTCCCTGCCTTCAGGAACCC  3130

Seq_1  3273  AGATTTCGTTTGCTGTGATCTCATTCATGACGTCCGCGACGGTGCCAGCGGCCGGCTCCG  3332
             ||||||||||||||||||||||||||||||||||||||||||||||||||||||||||||
```

```
Seq_2  3131  AGATTTCGTTTGCTGTGATCTCATTCATGACGTCCGCGACGGTGCCAGCGGCCGGCTCCG  3190

Seq_1  3333  TAGCTCGCCGTGGCTGCCAGCCAGAGGTCGAGGCCGTTCTTGGCGCCTTGCCAGACCGTT  3392
             |||||||||||| |||||||||||||||||||||||||||||||||||||||||||||||
Seq_2  3191  TAGCTCGCCGTGCCTGCCAGCCAGAGGTCGAGGCCGTTCTTGGCGCCTTGCCAGACCGTT  3250

Seq_1  3393  ATATGGAAACGTCGACGCACGGCCATACAGGATCTTTCTTGTCTTTGTGTTACCTGATGT  3452
             ||||||||||||||||||||||||||||||||||||||||||||||||||||||||||||
Seq_2  3251  ATATGGAAACGTCGACGCACGGCCATACAGGATCTTTCTTGTCTTTGTGTTACCTGATGT  3310

Seq_1  3453  ATTAAACCGTTCAACAATGTTGCCGCGCGTAGTGCCCGTTTTCTAAAAAGCAATGTTCCA  3512
             ||||||||||||||||||||||||||||||||||||||||||||||||||||||||||||
Seq_2  3311  ATTAAACCGTTCAACAATGTTGCCGCGCGTAGTGCCCGTTTTCTAAAAAGCAATGTTCCA  3370

Seq_1  3513  GGCAGTACAACATCAAGCTTAATAAACCACCAGCTAAAACAAAGCTTATAAACCACTGGA  3572
             ||||||||||||||||||||||||||||||||||||||||||||||||||||||||||||
Seq_2  3371  GGCAGTACAACATCAAGCTTAATAAACCACCAGCTAAAACAAAGCTTATAAACCACTGGA  3430

Seq_1  3573  AATGCTTAATTGGGACTATTATCACCATGCACAAGATCAGTATACCGAATAATAATGCAT  3632
             ||||||||||||||||||||||||||||||||||||||||||||||||||||||||||||
Seq_2  3431  AATGCTTAATTGGGACTATTATCACCATGCACAAGATCAGTATACCGAATAATAATGCAT  3490

Seq_1  3633  GGAAGAAGCGCATATAGCACGCGCCAATCAGTCGCGCGCATGCAGGCAGCATGCCCACCT  3692
             ||||||||||||||||||||||||||||||||||||||| ||||||||||||||||||||
Seq_2  3491  GGAAGAAGCGCATATAGCACGCGCCAATCAGTCGCGCACATGCAGGCAGCATGCCCACCT  3550

Seq_1  3693  TGTTGGAATGCAGCAAAGTCCGAAAGGCCCCATGACCGAGTGGCGCCGCCGCAACCCCAA  3752
             ||||||||||||||||||||||||||||||||||||||||||||||||||||||||||||
Seq_2  3551  TGTTGGAATGCAGCAAAGTCCGAAAGGCCCCATGACCGAGTGGCGCCGCCGCAACCCCAA  3610

Seq_1  3753  CAAATTTACACGCCGTACAAAACAAAATCGCACCGATCTCGAGGCGCACACTCTATAATT  3812
             ||||||||||||||||||||||||||||||||||||||||||||||||||||||||||||
Seq_2  3611  CAAATTTACACGCCGTACAAAACAAAATCGCACCGATCTCGAGGCGCACACTCTATAATT  3670

Seq_1  3813  CCACGTTCCGTGCCCCGCAGACACCAATAATTTCCGAGCACGGGCAAACGTGCACGGCTG  3872
             |||||||||||||||||||||||||||||||||||||||||||||| |||||||||||||
Seq_2  3671  CCACGTTCCGTGCCCCGCAGACACCAATAATTTCCGAGCACGGGCTAACGTGCACGGCTG  3730

Seq_1  3873  TACCCAGCTACATATACACCGTACCACCTGACGAGCTCAAGTGGCTCAAGGCTCGAGAAG  3932
             ||||||||||||||||||||||||||||||||||||||||||||||||||||||||||||
Seq_2  3731  TACCCAGCTACATATACACCGTACCACCTGACGAGCTCAAGTGGCTCAAGGCTCGAGAAG  3790

Seq_1  3933  CAGAGTGCGTTCAACGTGGTATATAGTGAGAGAGAGACGGTGATCTTTGCTGAGCCAGCA  3992
             ||||||||||||||||||||||||||||||||||||||||||||||||||||||||||||
Seq_2  3791  CAGAGTGCGTTCAACGTGGTATATAGTGAGAGAGAGACGGTGATCTTTGCTGAGCCAGCA  3850

Seq_1  3993  GCCATGGCGGGGACGTCGTTGCCGGCGTCGGAGAGGCCGCCGCACGCCGTGATGATCCCG  4052
             ||||||||||||||||||||||||||||||||||||||||||||||||||||||||||||
Seq_2  3851  GCCATGGCGGGGACGTCGTTGCCGGCGTCGGAGAGGCCGCCGCACGCCGTGATGATCCCG  3910

Seq_1  4053  TACCCGGCGCAGGGCCACGTGACGCCGATGCTGAAGCTCGCCAAGCTGCTCCACGCCCGG  4112
             ||||||||||||||||||||||||||||||||||||||||||||||||||||||||||||
Seq_2  3911  TACCCGGCGCAGGGCCACGTGACGCCGATGCTGAAGCTCGCCAAGCTGCTCCACGCCCGG  3970

Seq_1  4113  GGCTTCCACGTCACCTTCGTCAACAACGAGTTCAACCAACGCCGCCTGCAGCGCGCGCAG  4172
             ||||||||||||||||||||||||||||||||||||||| ||||||||||||||||||||
```

```
Seq_2  3971  GGCTTCCACGTCACCTTCGTCAACAACGAGTTCAACCACCGCCGCCTGCAGCGCGCGCAG  4030

Seq_1  4173  GGCGGCGGTCCCGGCGCGCTCGACGGCGCGCCCGGATTCCGCTTCGCCACCATCGACGAC  4232
             ||||||||||||||||||||||||||||||||||||||||||||||||||||||||||||
Seq_2  4031  GGCGGCGGTCCCGGCGCGCTCGACGGCGCGCCCGGATTCCGCTTCGCCACCATCGACGAC  4090

Seq_1  4233  GGCCTCCCTCGCTCGGACCGCGACGCCCAGCAGGACGTCCCTTCGCTGTGCCGCTCCACC  4292
             ||||||||||||||||||||||||||||||||||||||||||||||||||||||||||||
Seq_2  4091  GGCCTCCCTCGCTCGGACCGCGACGCCCAGCAGGACGTCCCTTCGCTGTGCCGCTCCACC  4150

Seq_1  4293  ATGACCACCTGCCTCCCCAGGTTCAAGGCGCTCATCGCCAGGCTCAACGAAGACGCCGAT  4352
             ||||||||||||||||||||||||||||||||||||||||||||||||||||||||||||
Seq_2  4151  ATGACCACCTGCCTCCCCAGGTTCAAGGCGCTCATCGCCAGGCTCAACGAAGACGCCGAT  4210

Seq_1  4353  GGCGCCGCGCCGCCCGTGACATGCGTCGTCGGCGACAGCACCATGACCTTCGCCCTCCGC  4412
             ||||||||||||||||||||||||||||||||||||||||||||||||||||||||||||
Seq_2  4211  GGCGCCGCGCCGCCCGTGACATGCGTCGTCGGCGACAGCACCATGACCTTCGCCCTCCGC  4270

Seq_1  4413  GCTGCTAAGGAGCTCGGCCTCCGCTGCGCCACGCTCTGGACCGCCAGCGCCTGCGGCTTC  4472
             ||||||||||||||||||||||||||||||||||||||||||||||||||||||||||||
Seq_2  4271  GCTGCTAAGGAGCTCGGCCTCCGCTGCGCCACGCTCTGGACCGCCAGCGCCTGCGGCTTC  4330

Seq_1  4473  ATGGGGTACGCCCACTACAAGGACCTTGTCCAACGTGGTCTCTTCCCTCTCAAAGGTATA  4532
             ||||||||||||||||||||||||||||||||||||||||||||||||||||||||||||
Seq_2  4331  ATGGGGTACGCCCACTACAAGGACCTTGTCCAACGTGGTCTCTTCCCTCTCAAAGGTATA  4390

Seq_1  4533  TACGGGCATATGGCAGAAAAAAATGAGTAAACAAAGAAATTACCCACTTGAGATCGATGA  4592
             ||||||||||||||||||||||||||||||||||||||||||||||||||||||||||||
Seq_2  4391  TACGGGCATATGGCAGAAAAAAATGAGTAAACAAAGAAATTACCCACTTGAGATCGATGA  4450

Seq_1  4593  TGAAAGTTCCGTTTTGGGTGCAGACGAGGCGCAGTTGAGCAATGGGTACCTGGACACGAC  4652
             |||| |||||||||||||||||||||||||||||||||||||||||||||||||||||||
Seq_2  4451  TGAA-GTTCCGTTTTGGGTGCAGACGAGGCGCAGTTGAGCAATGGGTACCTGGACACGAC  4509

Seq_1  4653  AGTGGACTGGATACCGGGGTTGCCGAAGGACCTGCGGCTGCGGGACTTGCCAAGCTTCGT  4712
             ||||||||||||||| ||||||||||||||||||||||||||||||||||||||||||||
Seq_2  4510  AGTGGACTGGATACCCGGGTTGCCGAAGGACCTGCGGCTGCGGGACTTGCCAAGCTTCGT  4569

Seq_1  4713  GCGCAGCACCGACCCGGACGACATCATGTTCAACTTCTTCGTCCACGAGACGGCCGGCAT  4772
             ||||||||||||||||||||||||||||||||||||||||||||||||||||||||||||
Seq_2  4570  GCGCAGCACCGACCCGGACGACATCATGTTCAACTTCTTCGTCCACGAGACGGCCGGCAT  4629

Seq_1  4773  GGCGCAGGCGTCCGGGGTGGTCATCAACACCTTCGACGAGCTGGACGCGCCGCTGCTGGG  4832
             ||||||||||||||||||||||||||||||||||||||||||||||||||||||||||||
Seq_2  4630  GGCGCAGGCGTCCGGGGTGGTCATCAACACCTTCGACGAGCTGGACGCGCCGCTGCTGGG  4689

Seq_1  4833  CGCCATGTCGAAGCTCCTGCCGCCGGTCTACACCGTGGGGCCGCTCCATCTCACGGTGCG  4892
             ||||||||||||||||||||||||||||||||||||||||||||||||||||||||||||
Seq_2  4690  CGCCATGTCGAAGCTCCTGCCGCCGGTCTACACCGTGGGGCCGCTCCATCTCACGGTGCG  4749

Seq_1  4893  GAACAACGTGCCAGCGGAGAGCCCGGTGGCCGGCATCGACTCCAGCCTCTGGATTCAGCA  4952
             ||||||||||||||||||||||||||||||||||||||||||||||||||||||||||||
Seq_2  4750  GAACAACGTGCCAGCGGAGAGCCCGGTGGCCGGCATCGACTCCAGCCTCTGGATTCAGCA  4809

Seq_1  4953  GCAGGACGCGCCGCTCCGGTGGCTCGACGGCCGGGCGCCGGGCTCCGTGGTGTACGTCAA  5012
             ||||||||||||||||||||||||||||||||||||||||||||||||||||||||||||
```

```
Seq_2  4810  GCAGGACGCGCCGCTCCGGTGGCTCGACGGCCGGGCGCCGGGCTCCGTGGTGTACGTCAA  4869

Seq_1  5013  CTTCGGGAGCATCACGGTGATGTCGAACGAGCACCTGCTGGAGTTCGCGTGGGGACTGGC  5072
             ||||||||||||||||||||||||||||||||||||||||||||||||||||||||||||
Seq_2  4870  CTTCGGGAGCATCACGGTGATGTCGAACGAGCACCTGCTGGAGTTCGCGTGGGGACTGGC  4929

Seq_1  5073  CAACACCGGCTACGCCTTCCTCTGGAACGTGCGGCCGGACCTCGTCCGGGGCGACGAGGC  5132
             ||||||||||||||||||||||||||||||||||||||||||||||||||||||||||||
Seq_2  4930  CAACACCGGCTACGCCTTCCTCTGGAACGTGCGGCCGGACCTCGTCCGGGGCGACGAGGC  4989

Seq_1  5133  CGCCCTGCCGCCGGAGTTCTCCGCGGCCACGGCGGGGCGGAGCATGCTGACGACGTGGTG  5192
             ||||||||||||||||||||||||||||||||||||||||||||||||||||||||||||
Seq_2  4990  CGCCCTGCCGCCGGAGTTCTCCGCGGCCACGGCGGGGCGGAGCATGCTGACGACGTGGTG  5049

Seq_1  5193  CCCGCAGGAGAAGGTGCTGGAGCACGAGGCCGTGGGGGTGTTCCTCACGCACTCCGGCTG  5252
             ||||||||||||||||||||||||||||||||||||||||||||||||||||||||||||
Seq_2  5050  CCCGCAGGAGAAGGTGCTGGAGCACGAGGCCGTGGGGGTGTTCCTCACGCACTCCGGCTG  5109

Seq_1  5253  GAACTCCACGCTCGAGAGCATCTGCGGCGGGGTCCCCATGGTTTGCTGGCCGTTCTTCGC  5312
             ||||||||||||||||||||||||||||||||||||||||||||||||||||||||||||
Seq_2  5110  GAACTCCACGCTCGAGAGCATCTGCGGCGGGGTCCCCATGGTTTGCTGGCCGTTCTTCGC  5169

Seq_1  5313  CGAGCAGCAGACCAACTGCCGGTTCAAGCGCACGGAGTGGGGAATTGGGGTGGAGGTGCC  5372
             ||||||||||||||||||||||||||||||||||||||||||||||||||||||||||||
Seq_2  5170  CGAGCAGCAGACCAACTGCCGGTTCAAGCGCACGGAGTGGGGAATTGGGGTGGAGGTGCC  5229

Seq_1  5373  CGACGAGGTGAGGAGAGACGAGGTGGAGGCCATGATACGGGAGGCCATGGAGGGGGAGAA  5432
             ||||||||||||||||||||||||||||||||||||||||||||||||||||||||||||
Seq_2  5230  CGACGAGGTGAGGAGAGACGAGGTGGAGGCCATGATACGGGAGGCCATGGAGGGGGAGAA  5289

Seq_1  5433  AGGCCGCGACATGCGGCGGCGCGTGCTGGAGCTCCGGGACAGCGCGCTGGCCTCGGCCAA  5492
             ||||||||||||||||||||||||||||||||||||||||||||||||||||||||||||
Seq_2  5290  AGGCCGCGACATGCGGCGGCGCGTGCTGGAGCTCCGGGACAGCGCGCTGGCCTCGGCCAA  5349

Seq_1  5493  GCCTGGCGGCCGCTCCATGTGCAATGTTGATAGGCTCATTCAGGAAGTGTTGCTTGCTTG  5552
             ||||||||||||||||||||||||||||||||||||||||||||||||||||||||||||
Seq_2  5350  GCCTGGCGGCCGCTCCATGTGCAATGTTGATAGGCTCATTCAGGAAGTGTTGCTTGCTTG  5409

Seq_1  5553  AAGCAGCCATTGTTGATGTCTTCTGATATAAAACCTCATCTTGGGTTCGTGGCGTAGGCA  5612
             ||||||||||||||||||||||||||||||||||||||||||||||||||||||||||||
Seq_2  5410  AAGCAGCCATTGTTGATGTCTTCTGATATAAAACCTCATCTTGGGTTCGTGGCGTAGGCA  5469

Seq_1  5613  TGTGCAATTAATTGGTGCTTCTTCTAATGAGGAAATGCAACTGTTCACTCGCTGGTTTTC  5672
             ||||||||||||||||||||||||||||||||||||||||||||||||||||||||||||
Seq_2  5470  TGTGCAATTAATTGGTGCTTCTTCTAATGAGGAAATGCAACTGTTCACTCGCTGGTTTTC  5529

Seq_1  5673  AAAAGTAAATAGAAAATAAAAATAAAAATAAATAAACAACAGAAGAATGCACGGTCTCGA  5732
             ||||||||||||||||||||||||||||   |||||||||||||||||||||||||||||
Seq_2  5530  AAAAGTAAATAGAAAATAAAAATAAAAA---ATAAACAACAGAAGAATGCACGGTCTCGA  5586

Seq_1  5733  CGTGCAGGCTGCAGCCATCTCCAGCAAAGTGGCTTTTACTAACTGAAGCAATTTTCATTT  5792
             |||||||||||||||||||||||||||                        |||||||||
Seq_2  5587  CGTGCAGGCTGCAGCCATCTCCAGCAA------------------------TTTTCATTT  5622

Seq_1  5793  TTTTTTACCAAGAAGCAATTTTCACTAATTCGGGAGGACGATCCATTTTTTCTTAATTAT  5852
             ||||||||||||||||||||||||||||||||||||||||||||||||||||||||||||
```

```
Seq_2  5623  TTTTTACCAAGAAGCAATTTTCACTAATTCGGGAGGACGATCCATTTTTTCTTAATTAT  5682

Seq_1  5853  GCACTTAACGCTCTTTCCTTGGTACATACTCCATCTGTCCATCAATTTGCTCTTTTATCA  5912
             ||||||||||||||||||||||||||||||||||||||||||||||||||||||||||| 
Seq_2  5683  GCACTTAACGCTCTTTCCTTGGTACATACTCCATCTGTCCATCAATTTGCTCTTTTATCG  5742

Seq_1  5913  TATCACTCGATTTTTATTACTTCCTCCGTTTCATAATTCTTGTCTCAAATTGTCCAAAAA  5972
             ||||||||||||||||||||| |||||||||||||||||||||||||||||||| |||||
Seq_2  5743  TATCACTCGATTTTTATTACTCCCTCCGTTTCATAATTCTTGTCTCAAATTGTCTAAAAA  5802

Seq_1  5973  TGAATGTATCTATTTCTAAAAAGTATCTAAATACATGTAAGATTTCGACAAGAATTATGA  6032
             | ||||||||||||||||||||||||||| |||||||||||||||||||||||||||| |
Seq_2  5803  TAAATGTATCTATTTCTAAAAAGTATCTAGATACATGTAAGATTTCGACAAGAATTATAA  5862

Seq_1  6033  AACGGAGGTAGTACTTTCGGTTGATAAACATGGACACTATGCTGTCTTGTGAATATGCAA  6092
             ||||||||||||||||||||||||||||||||||||||||||||||||||||||||||||
Seq_2  5863  AACGGAGGTAGTACTTTCGGTTGATAAACATGGACACTATGCTGTCTTGTGAATATGCAA  5922

Seq_1  6093  GGTAGGTCTCAATTAACCTGCAGTAAATATGCTCTTTTATCGTATCACTCGGCTCTTTTA  6152
             ||||||||||||||||||||||||||||||||||||||||||||||||||||||||||||
Seq_2  5923  GGTAGGTCTCAATTAACCTGCAGTAAATATGCTCTTTTATCGTATCACTCGGCTCTTTTA  5982

Seq_1  6153  TCATATCACTCGATTCAACCACTGGCCATAAGCTAGCTTCATAAAAGAATTGAATTCTCC  6212
             ||||||||||||||||||||||||||||||||||||||||||||||||||||||||||||
Seq_2  5983  TCATATCACTCGATTCAACCACTGGCCATAAGCTAGCTTCATAAAAGAATTGAATTCTCC  6042

Seq_1  6213  GAATTTATTACACCAACCATTCGATTGCAAATAATGTTGTAGTAGTTGTTCATGGTAGTA  6272
             ||||||||||||||||||||||||||||||||||||||||||||||||||||||||||||
Seq_2  6043  GAATTTATTACACCAACCATTCGATTGCAAATAATGTTGTAGTAGTTGTTCATGGTAGTA  6102

Seq_1  6273  CCAACTACGGACACTGTAAATTCAAGTATGTACGTACACCGTAAATTCATCCTATTTGGT  6332
             ||||||||||||||||||||||||||||||||||||||||||||||||||||||||||||
Seq_2  6103  CCAACTACGGACACTGTAAATTCAAGTATGTACGTACACCGTAAATTCATCCTATTTGGT  6162

Seq_1  6333  GAACAAGTATGCCGAGAGTACCTATATAAAAACAGTCCCGTCATGTTACACAACCGGTTG  6392
             |||||| |||||||||||||||||||||||||||||||||||||||||||||||||||||
Seq_2  6163  GAACAATTATGCCGAGAGTACCTATATAAAAACAGTCCCGTCATGTTACACAACCGGTTG  6222

Seq_1  6393  CATTCTCATCGTTCCAATGGAACTATAGTGGAGTAGTGAAAGCAAGGATAAGAGAATACA  6452
             ||||||||||||||||||||||||||||||||||||||||||||||||||||||||||||
Seq_2  6223  CATTCTCATCGTTCCAATGGAACTATAGTGGAGTAGTGAAAGCAAGGATAAGAGAATACA  6282

Seq_1  6453  AGTAGCGTTCGTTCATGTTGGAAATGAGTTTGTATTGGAAATGGAGGCATCGGA------  6506
             ||||||||| ||||||||||| ||| ||||||||||||||||||||||||||||      
Seq_2  6283  AGTAGCGTTGGTTCATGTTGGCAATAAGTTTGTATTGGAAATGGAGGCATCGGAAACTCA  6342

Seq_1  6507  -GTTCGTGCCACAGATCTTCCTCTCATCCCACCAGGCCACCACGGCCGATCTATGTGCAA  6565
              |||||||||||||||||||||||||||||||||||||||||||||||||||||||||||
Seq_2  6343  AGTTCGTGCCACAGATCTTCCTCTCATCCCACCAGGCCACCACGGCCGATCTATGTGCAA  6402

Seq_1  6566  TGTTGATAGGCTCATTCAGGAAGTGTTGCTTGCTTGAAGTAGCCATTGTTGATGTCTTTT  6625
             ||||||||||||||||||||||||||| ||||||||||| |||||||||||||||||| |
Seq_2  6403  TGTTGATAGGCTCATTCAGGAAGTGTTACTTGCTTGAAGCAGCCATTGTTGATGTCTTCT  6462

Seq_1  6626  GATATAAGACCCCATCTTCGGTTTGTGGCCAGGGCTGTGCAATTAGTTTGTGCTTCTTCT  6685
             |||||||||||||||||| |||| |||||| |||||||||||||||||| ||||||||||
```

```
Seq_2  6463  GATATAAGACCCCATCTTGGGTTCGTGGCCTGGGCTGTGCAATTAGTTTTTGCTTCTTCT  6522

Seq_1  6686  TCTAATGATGAAGAGTAATGCGGCTGTTCACTCGCTGTTTTTTATTTCGAAAAAGTAAT  6745
             |||||||||||||||||||||||||||||||||||||||||||||||| |||||||||||
Seq_2  6523  TCTAATGATGAAGAGTAATGCGGCTGTTCACTCGCTGTTTTTTATTTCAAAAAAGTAAT  6582

Seq_1  6746  TTTATTCTGAAGCTGCTATGCTCGACAAAACAGCAGAAGAATTCACGGCCTCAAGTCAGG  6805
             ||||||||||| ||||||||||||||||||||||||||||||||||||||||||||||||
Seq_2  6583  TTTATTCTGAAACTGCTATGCTCGACAAAACAGCAGAAGAATTCACGGCCTCAAGTCAGG  6642

Seq_1  6806  CGCAGGCGTGCAGCCATCTCCAGCAACAGCAAGTGTAACAGCCAGTGGCCTGTAGCCCTG  6865
             |||||||||||||||||||||| |||||||||||||||||||||||||||||||||| ||
Seq_2  6643  CGCAGGCGTGCAGCCATCTCCAACAACAGCAAGTGTAACAGCCAGTGGCCTGTAGCCTTG  6702

Seq_1  6866  TTTAACTGATTACCTGAAGCACTCTTCACTTGTCAGGAGGACGATCAAGACTTTTCTAAA  6925
             ||||||||||||||||||||||||||||||||||||||||||||||||||||||||||||
Seq_2  6703  TTTAACTGATTACCTGAAGCACTCTTCACTTGTCAGGAGGACGATCAAGACTTTTCTAAA  6762

Seq_1  6926  TTCAGCAGTGTAACATATTAAGTACAAAATTTCCGGTTCAGTACCACGAAGGTTGCAGCT  6985
             ||||||||||||||||||||||||||||||||||||||||||||||||||||||||||||
Seq_2  6763  TTCAGCAGTGTAACATATTAAGTACAAAATTTCCGGTTCAGTACCACGAAGGTTGCAGCT  6822

Seq_1  6986  GCCATGATGTTCAATAGTACGTCTCTATGAATTTGCCCTCTTACAGTATCACTCTATTTT  7045
             ||||||||||||||||||||||||||||||||||||||||||||||||||||||||||||
Seq_2  6823  GCCATGATGTTCAATAGTACGTCTCTATGAATTTGCCCTCTTACAGTATCACTCTATTTT  6882

Seq_1  7046  TATTACTTTCGGATGATAAACATGGACACTCAGCTATGTTGTGAATATGCAAGGTAGGTC  7105
             ||||||||||||||||||||||||||||||||||||||||||||||||||||||||||||
Seq_2  6883  TATTACTTTCGGATGATAAACATGGACACTCAGCTATGTTGTGAATATGCAAGGTAGGTC  6942

Seq_1  7106  TCAATTAACCTGTCATGCATCAAGGCCATTATTCAACCACTGGCCATAAGCTATCTTCAT  7165
             ||||||||||||||||||||||||||||||||||||||||||||||||||||||||||||
Seq_2  6943  TCAATTAACCTGTCATGCATCAAGGCCATTATTCAACCACTGGCCATAAGCTATCTTCAT  7002

Seq_1  7166  AAAAGAATTGAATTCTCCCAATTTATTGTACCAACCATTCGATTGCAAATAATGTTGTCA  7225
             ||||||||||||||||||||||||||||||||||||||||||||||||||||||||||||
Seq_2  7003  AAAAGAATTGAATTCTCCCAATTTATTGTACCAACCATTCGATTGCAAATAATGTTGTCA  7062

Seq_1  7226  CAGTTGTTCATTATTGTACTGACTATGGACACTGTAAATTCATATGTGTACTGACACTGT  7285
             ||||||||||||||||||||||||||||||| ||||||||||||| ||||||||||||||
Seq_2  7063  CAGTTGTTCATTATTGTACTGACTATGGACAATGTAAATTCATATATGTACTGACACTGT  7122

Seq_1  7286  AAATTCATCCGATTTTGTGAACAACTATGTCAGGAATACCTATATAAAAGCAGTCGAGTT  7345
             ||||||||||||||||||||||||||||||||||||||||||||||||||||||||||||
Seq_2  7123  AAATTCATCCGATTTTGTGAACAACTATGTCAGGAATACCTATATAAAAGCAGTCGAGTT  7182

Seq_1  7346  CATGTTAAACAACCCGGTTGTAATCTCATCGTTCCAATGGAACTATAGTAGTGAAAGTAG  7405
             ||||||||||||||||||||||||||||||||||||||| ||||||||||||||||||||
Seq_2  7183  CATGTTAAACAACCCGGTTGTAATCTCATCGTTCCAATGAAACTATAGTAGTGAAAGTAG  7242

Seq_1  7406  AGGATATGAGAATACAAGTAGTGTTCGTTTATGTTGGCAATAAGTTTGTATCGGCAAAGG  7465
             ||||||||||||||||||||||||||||||||||||||||||| ||||||||||||||||
Seq_2  7243  AGGATATGAGAATACAAGTAGTGTTCGTTTATGTTGGCAATAAATTTGTATCGGCAAAGG  7302

Seq_1  7466  AGGCATCGGAAACTCAAGTTCGTGCCACAGATCTTCCACTCATCCGAGCACGGACCATGT  7525
             ||||||||||||||||||||||||||||||||||||||||||||||||||||||||||||
```

Seq_2 7303 AGGCATCGGAAACTCAAGTTCGTGCCACAGATCTTCCACTCATCCGAGCACGGACCATGT 7362

Seq_1 7526 TGCGAGAACCAAGATCAAAGGAAGGAATGGTAGCTGGATATAGCCAGGATACATTAATCT 7585
||| ||||||||||||||||||||||||||||||||||||||||||||||||||||||||
Seq_2 7363 TGCTAGAACCAAGATCAAAGGAAGGAATGGTAGCTGGATATAGCCAGGATACATTAATCT 7422

Seq_1 7586 ATTCTTCAGAATACGATGGACTGATAGCCAGAGATTCCAATTTCCGAGTTATTGAGGATG 7645
||||||||||||||||||||||||||||||||||||||||||||||||||||||||||||
Seq_2 7423 ATTCTTCAGAATACGATGGACTGATAGCCAGAGATTCCAATTTCCGAGTTATTGAGGATG 7482

Seq_1 7646 GATAGGTCGTGATGATGCTGGTTGCATTTGGAAGGGCGACGCTCCCGACCGTAACTAGCA 7705
||||||||||||||||||||||||||||||||||||||||||||||||||||||||||||
Seq_2 7483 GATAGGTCGTGATGATGCTGGTTGCATTTGGAAGGGCGACGCTCCCGACCGTAACTAGCA 7542

Seq_1 7706 CACTGGCACCGTAGTAGTTTCGCTCAGAAAGTTCGTGCATCCTAGAGTTCATCAAGGAAC 7765
||||||||||||||||||||||||||||||||||||||||||||||||||||||||||||
Seq_2 7543 CACTGGCACCGTAGTAGTTTCGCTCAGAAAGTTCGTGCATCCTAGAGTTCATCAAGGAAC 7602

Seq_1 7766 ATGCTGCATCTAGTATCATGACTCGTTTCGTTCGGCAGGGGAAGCCGCCGGCAGCATCAA 7825
|||||||||||||||||||||||||||||||||||||||||||||||||||||||| |||
Seq_2 7603 ATGCTGCATCTAGTATCATGACTCGTTTCGTTCGGCAGGGGAAGCCGCCGGCAGCACCAA 7662

Seq_1 7826 AACGTTAGGCATGCTGCTGCTCGTTTGCGTAGAATCATTCAGGGTTGTGGCATGTCCGTC 7885
||||||||||||||||||||||||||||||||||||||||||||||||||||||||||||
Seq_2 7663 AACGTTAGGCATGCTGCTGCTCGTTTGCGTAGAATCATTCAGGGTTGTGGCATGTCCGTC 7722

Seq_1 7886 CGCAGTTCACTGGCAGCAATTGTAATCCCCCGGCTGCTGCAGAGAGCATACCACAAGAAT 7945
||||||||||||||||||||||||||||||||||||||||||||||||||||||||||||
Seq_2 7723 CGCAGTTCACTGGCAGCAATTGTAATCCCCCGGCTGCTGCAGAGAGCATACCACAAGAAT 7782

Seq_1 7946 CATTGGCCAGAAAAAAAGTTGGGGGCGTTTGGACTGGAGCCGTTGGGGGCGCTTGGGACT 8005
||||||||||||||| ||||||||||||||||||||||||||||||||||||||||||||
Seq_2 7783 CATTGGCCAGAAAAAGAGTTGGGGGCGTTTGGACTGGAGCCGTTGGGGGCGCTTGGGACT 7842

Seq_1 8006 CTCCCCCGCGTATCCTCGGCGATGTCTCCAGCGAGGATGTCAACCAGTTGGTGTTGAGGG 8065
||||||||||||||||||||||||||||||||||||||||||||||||||||||||||||
Seq_2 7843 CTCCCCCGCGTATCCTCGGCGATGTCTCCAGCGAGGATGTCAACCAGTTGGTGTTGAGGG 7902

Seq_1 8066 CTCTGCTCCGGCGACAAAAAATTCCAGCCTTCCAAAGCTTGTTCGTAGTTTCACACGATT 8125
||||||||||||||||||||||||||||||||||||||||||||||||||||||||||||
Seq_2 7903 CTCTGCTCCGGCGACAAAAAATTCCAGCCTTCCAAAGCTTGTTCGTAGTTTCACACGATT 7962

Seq_1 8126 AAACACAACCAATAATCAGACACATACAAAAGGGGCCTACCGAGCACAACACAGACATTG 8185
||||||||||||||||||||||||||||||||||||||||||||||||||||||||||||
Seq_2 7963 AAACACAACCAATAATCAGACACATACAAAAGGGGCCTACCGAGCACAACACAGACATTG 8022

Seq_1 8186 GGGAAAAAATATTCACCGAGTTGGGGGAAGGCATTCATTCAACACACGGCACCGATTACA 8245
||||||||||||||||||||||||||||| ||||||||||||||||||||||||||||||
Seq_2 8023 GGGAAAAAATATTCACCGAGTTGGGGGAATGCATTCATTCAACACACGGCACCGATTACA 8082

Seq_1 8246 ATGTTCAAGGAGAGCAATCACACTCTGGAACTGGTACACGAGTGGATATTTTAAGAAGCG 8305
||||||||||||||||||||||||||||||||||||||||||| ||||||||||||||||
Seq_2 8083 ATGTTCAAGGAGAGCAATCACACTCTGGAACTGGTACACGAGTAGATATTTTAAGAAGCG 8142

Seq_1 8306 ATTTGCGGTTCCTGATCCCAGGTACGATGCATAATTAGAAGCGGATAAATCCGGCTCCAG 8365
||||||||||||||||||||||||| ||||||||||||||||||||||||||||||||||

```
Seq_2  8143  ATTTGCGGTTCCTGATCCCAGGTACCATGCATAATTAGAAGCGGATAAATCCGGCTCCAG  8202

Seq_1  8366  CTGCCTGCATCTCCAGGATCTGAGCGGCGGCTGTGGCGGTGCTCCACTTTTCCTCTCTGA  8425
              ||||||||||||||||||||||||||||||||||||||||||||||||||||||||||||
Seq_2  8203  CTGCCTGCATCTCCAGGATCTGAGCGGCGGCTGTGGCGGTGCTCCACTTTTCCTCTCTGA  8262

Seq_1  8426  TCTAACCGGCATGGGCGGCGACCAGTGGCGCGTCGCTGTCCGCTGCCTTTTCCTCCCTGA  8485
              ||||||||||||||||||||||||||||||||||||||||||||||||||||||||||||
Seq_2  8263  TCTAACCGGCATGGGCGGCGACCAGTGGCGCGTCGCTGTCCGCTGCCTTTTCCTCCCTGA  8322

Seq_1  8486  TCTGAGCGGCGGCGGCTGGTGCGTCGCCGTTCGCCATCTCGTTTCTGTCCACTTGGTGGC  8545
              ||||||||||||||||||||||||||||||||||||||||||||||||||||||||||||
Seq_2  8323  TCTGAGCGGCGGCGGCTGGTGCGTCGCCGTTCGCCATCTCGTTTCTGTCCACTTGGTGGC  8382

Seq_1  8546  TCTGCTTCACGTCCTCGACTACGACGACCCTGATGCGCCGATGCGCTTCGCATCTCCTTC  8605
              ||||||||||||||||||||||||||||||||||||||||||||||||||||||||||||
Seq_2  8383  TCTGCTTCACGTCCTCGACTACGACGACCCTGATGCGCCGATGCGCTTCGCATCTCCTTC  8442

Seq_1  8606  CTCCTCGACCACACAGACACGACTGATACTCTGATAGGCCCCAAGATATTCCAAGGACGC  8665
              ||||||||||||||||||||||||||||||||||||||||||||||||||||||||||||
Seq_2  8443  CTCCTCGACCACACAGACACGACTGATACTCTGATAGGCCCCAAGATATTCCAAGGACGC  8502

Seq_1  8666  TTTTTGGCGCGATGCAACGGCATATCTTCGGCCTCCCTCCCTCTCTGCCCCTTCCGGCAG  8725
              ||||||||||||||||||||||||||||||||||||||||||||||||||||||||||||
Seq_2  8503  TTTTTGGCGCGATGCAACGGCATATCTTCGGCCTCCCTCCCTCTCTGCCCCTTCCGGCAG  8562

Seq_1  8726  CGGCAGCCTTATCCTCTCCACCGCCCCAAACTCTCACATCTGCTCTGCTTGGAGAAGCAG  8785
              ||||||||||||||||||||||||||||||||||||||||||||||||||||||||||||
Seq_2  8563  CGGCAGCCTTATCCTCTCCACCGCCCCAAACTCTCACATCTGCTCTGCTTGGAGAAGCAG  8622

Seq_1  8786  C  8786
              |
Seq_2  8623  C  8623
```

BdindelWSU_20, UPSTREAM

>Bradi5g16380

TCGGCCACGGCATCCACGAGCGGCACGCCCTGCTGCTCGAGGTCGTCATGACGTTCGGGCTCATGTACACCGTGTACGCCACCGCTG
TGGACCGGAACGACGGCGTCGGCGCCATAGCGCCCGTCGCCATCGGCTTCGTCCTGGGCGCGAACATCCTCACCGGCGGACCCTTCG
ACGGCGCCGCCATGAACCCGGCGCGGGCGTTCGGCCCCGCGCTCGTCGGCTGGAACTGGAGCCACCACTGGGTTTACTGGGTCGGG
CCGATGATCGGCGCCGGGCTGGCCGGCGCGTTGTATGAGTTTGTCGTGGGCGAGCAGCCTGATCAGGCGCCACCAGCTGCTGCACG
GCTGCCCGAGCCCGTTGAGGATTACTGAGGTGTTCAGTTGTGGTTAATGCCTCCAGGCTGTGCAGCTTGGAGTCTACGAGCAGACAG
GGATCTCATGGAAGGAATTAATTATGGCTACTAGAGAAAGAAACACTTGCAGGGTTTTAGGTTGCTAATACAAGTTTTCTAGTACAGT
AATACGTTCGATCACTCCATGATGTCCGAAATCCAATCTGCCACATTCGTTTCCTTCATTATAGCTGAATACTTCAGCTACTCTAATTTG
TGCGAACAATAGATTTTAAACTTTTTTTTTCCACCTGGATTTCTCCCCTTTCCATTTATGCAAACGGAAATGAAAACGAGAGGAGAAAC
ATGGGCACAAACTAACCTACTCTGGGCAGGAAACCCACAGACCGACCTCTGCCAATATATTTAAAACAGTAAACTGCGCAAAACCTTC
TAGAGCATTTGATATGTTACTAAAACAGTAAATTGCGCAAAACCACAATGCCGTTGTACGGGCGTATTGTAGGCGTATTATAGACAG
AACTACGGCAGTTAATTTGGATTGTGGGATTTATTATTTTGCAAAACACTACTTTCTAAACGATTCGTACACTAACTACGACGAAACCA
TCACAGTATTCCATCCGTGGAAACAAAATTTTGCCAAGCAGGTAACACTCTTTCCACTTTCTTTCTTTTTTTTCGTCGTATCCCGAACTCA
AAGCACGCATAGGCTACGTTTTTTTGACTTCTAGAAATCAGGTTTTCCAAAAAACTGTCTGCACACCAAATTTGGTTAGGTTTCCAAAC
TAGTTTAGATTAAACAGGTTTGAAAGCCAAACCAAATTTGGGATACAGCTTCTCTTGGAAGATGTGGCTTGAAGCCCTCAAGAAAACT
GGCCATAATCATTTTCTCCACGCTCGACCTCTTCTAGGCCTCCGGTTTCCTTTGTCCGGCTGGCAAAAATCCTATACGACCTAGGGGGA
CACAATCAGCGGTGAACTCGTGTCCTAAACGTACCGGAAAAGTCGTCACGTAAATTTGATTAGGATAACAAATTAGTTTGCCCTAAAT

TATTTTGTAAGTCTAACTAAATTTGTGATGTGGCTTTTGCAAGAAGTTAGGTGAGGACAGCTTGTACGAAAAGCCGGTTCTATGAGCC
GAAAAGAACACAGTATATACCAGAGTAGGCCTTAAACTTCTCCAGATTTAAAAGGGCCGAATAAGGAAATATGCTCACACAGCCTAT
AACCAGGTTTAACAATCGAGAACAGAAAACCAGTCTCTTCATGATCTCGCGCAAATAATCCCGCTGAATTATATTAGTTCTTCCGTGCC
ATATAAGTACGGAGGGAATACATGCCTTCGTCATGTCCGTCCAGTTTGAAAGCACACTGAACTTTTCCCCTGCCCATCCTTTTTTTTTC
AAAAAATGCCAGCATTCATAAGCTCGCAATGATATGAAATACAGAGTCGAGCCGTTCCAGAAGGCCCTCGCAAGCGGCCTGTATACG
CGCGTGGGCTAGAAGAAAGGAGAAACGCCTGAAAAACAATTCCAGTCAGGGCCCAGGGCACCCAATCCACATGTGGAGTTTGGACA
CTTGCCTGAGAGGCTCCCAAAGTTTCTTTTTTTTTAAAAAGGCTCCCAAAAGATCCCCTCCGCCTCCGTCCTCGAGCGAGCCAACGCAG
CCTCGCTTTTCGCCGCTCTCCGACCGCTCGACGCACGTTATCC

>BdiBd21-3.5G0212700

CTGATCAGGCGCCACCAGCTGCTGCACGGCTGCCCGAGCCCGTTGAGGATTACTGAGGTGTTCAGTTGTGGTTAATGCCTCCAGGCT
GTGCAGCTTGGAGTCTACGAGCAGACAGGGATCTCTTGGAAGGAATTAATTATGGCTACTAGAGAAAGAAACACTTGCAGGGTTTTA
TGTTGCTAATACAAGTTTTCTAGTACAGTAATACGTTCGATCACTCCATGATGTCCGAAATCCAATCTGCCACATTAGTTTCCTTCATTA
TAGCTGAATACTTCAGCTACTCTAATTTGTGCGAACAATAGATTTTAAACTTTTTTTTCCACCTGGATTTCTCCCCTTTCCATTTATGCAA
ACGGAAATGAAAACGAGAGGAGAAACATGGGCACAAACTAACCTAGTCTGGGCAGGAAACCCACAGACCGACCTCTGCCAATATAT
TTAAAACAGTAAACTGCGCAAAACCTTCTAGAGCATTTGATATGTTACTAAAACAGTAAATTGCGCAAAACTACAATGCCGTTGTACG
GGCGTATTGTAGACAGAACTACGGCAGTTAATTTGGATTGTAGGATTTATTATTTTGCAAAACACTACTTTCTAAACGATTCGTACACT
AACTACGACGAAACCATCACAGTATTCCATCCGTGGAAACAAAATTTTGCCAAGCAGGTAACACTCTTTCCACTTTCTTTCTGTCGTAT
CCTGAACTCAAAGCACGCATAGGCTGCGTTTTTTCGGCTTCTAGAAATCAGGTTCTCCGAAAAACTGCAGCACCAGCTTTCTAGATAA
ACTGCACACCAAATTTGGTTAGGTTTCCAAACTAATTTAGGTTAAATAGGTTTGAAAGCCAAACCAAATCTGAGATACAGCTTCTCTTG
AAAGACGTGGCTTGTGAGGAGGACAACTTTTCAGAGAAGCCCTCAAAAAAACTTGCCATAATCATTTTCTCCACGCTCGACCTCTTCT
AGGCCTCCGGCTTCCTTTGTCCGGCTGGCACAAATCCTATACGACCTAGGGGGACACAATCAGCGGTGAACTCGTGTCCTAAACGTA
CCTCGATCTGAGCGTATGAAGATAGTCACCGGAGTTGAGATATGGTATTAGATTTAGAGATGCAACGAACGATGGCAGGGGTCTAG
GGACGGATCGAGCAAGTCTAGGTTGTATGTGATTTTCCATCTTGCTAGAGAAGCAGATGGCGTCCCACATGAGCTTGCCCCCATCCCC
CACCCCCCACGTACGCACACACACAATCGTATATGTTTCACTTATAGATGTAACTGTGATCTCAGATGAATAAAGAGGCTTTACGATGT
AGAAAAGGAACCAACATGAGAATATCGTGTGGTTCGGCTTACCTTGTGTTTCCTGAACCGGCTTTTCCGAGAAGCTGTCCTTTTCTAG
TTTTTTTGAGAAATCGTCACCTAAATTTGATTAGGATAACAAATTAGTTTGTCCTAAACTATTTTGTAAGTCTAACTAAATTTGTGATGT
GGCTTTTGTAAGAAGTTAGGTGAGGACAGCTTGTACGAAAAACCAGTCCTATGAGCCGAAAAAAACATAGTATATACCAGAGTAGG
CCTTAAACTTCTCCAGATTTAAAAGGGCCGAATAAGGAAATATGCTCACACAGCCTATAACCAGGTTTAACAATCGAGAACAGAAAA
CCAGTTTCTTCATGATCTCGCGCAAATAATCCCGCTGAATTATATTAGTTCTTCCGTGCCATATAAGTACGGAGGGAATACATGCCTTC
GTCATGTCCGTCCAGTTTGAAAGCACACTGAACTTTTCCCCTGCCCATCCTTTTTTTTTTTCAAAAAATGCCAGCATTCATAAGCTCGCA
ATGATATGAAATACAGAGTCGAGCCGTTCCAGAAGGCCCTCGCAAGCGGCCTGTATACGCGCGTGGGCTAGAAGAAAGGAGAAAC
GCCTGAAAAACGATTCCAGTCAGGGCCCAGGGCACCCAATCCACATTTGGAGTTTGGACACTTGCCTGAGAGGCTCCCAAAGTTTCTT
TTATAAAAAAAAAAGGCTCCCAAAAGATCCCCTCCGCCTCCGTCCTCGAGCGAGCCAACGCAGCCTCGCTTTTCGCCGCTCTCCGACC
GCTCGACGCACGTTATCC

```
Alignment of Sequence_1:  [Untitled Sequence #1] with  Sequence_2: [Sequence Window #2]

Similarity : 1681/2131 (78.88 %)

Seq_1  1      TCGGCCACGGCATCCACGAGCGGCACGCCCTGCTGCTCGAGGTCGTCATGACGTTCGGGC  60

Seq_2  1      ------------------------------------------------------------  0


Seq_1  61     TCATGTACACCGTGTACGCCACCGCTGTGGACCGGAACGACGGCGTCGGCGCCATAGCGC  120

Seq_2  1      ------------------------------------------------------------  0


Seq_1  121    CCGTCGCCATCGGCTTCGTCCTGGGCGCGAACATCCTCACCGGCGGACCCTTCGACGGCG  180

Seq_2  1      ------------------------------------------------------------  0
```

```
Seq_1  181   CCGCCATGAACCCGGCGCGGGCGTTCGGCCCCGCGCTCGTCGGCTGGAACTGGAGCCACC  240

Seq_2  1     ------------------------------------------------------------  0


Seq_1  241   ACTGGGTTTACTGGGTCGGGCCGATGATCGGCGCCGGGCTGGCCGGCGCGTTGTATGAGT  300

Seq_2  1     ------------------------------------------------------------  0


Seq_1  301   TTGTCGTGGGCGAGCAGCCTGATCAGGCGCCACCAGCTGCTGCACGGCTGCCCGAGCCCG  360
                               ||||||||||||||||||||||||||||||||||||||||||
Seq_2  1     ------------------CTGATCAGGCGCCACCAGCTGCTGCACGGCTGCCCGAGCCCG  42


Seq_1  361   TTGAGGATTACTGAGGTGTTCAGTTGTGGTTAATGCCTCCAGGCTGTGCAGCTTGGAGTC  420
             ||||||||||||||||||||||||||||||||||||||||||||||||||||||||||||
Seq_2  43    TTGAGGATTACTGAGGTGTTCAGTTGTGGTTAATGCCTCCAGGCTGTGCAGCTTGGAGTC  102


Seq_1  421   TACGAGCAGACAGGGATCTCATGGAAGGAATTAATTATGGCTACTAGAGAAAGAAACACT  480
             |||||||||||||||||||| |||||||||||||||||||||||||||||||||||||||
Seq_2  103   TACGAGCAGACAGGGATCTCTTGGAAGGAATTAATTATGGCTACTAGAGAAAGAAACACT  162


Seq_1  481   TGCAGGGTTTTAGGTTGCTAATACAAGTTTTCTAGTACAGTAATACGTTCGATCACTCCA  540
             |||||||||||| |||||||||||||||||||||||||||||||||||||||||||||||
Seq_2  163   TGCAGGGTTTTATGTTGCTAATACAAGTTTTCTAGTACAGTAATACGTTCGATCACTCCA  222


Seq_1  541   TGATGTCCGAAATCCAATCTGCCACATTCGTTTCCTTCATTATAGCTGAATACTTCAGCT  600
             |||||||||||||||||||||||||||| |||||||||||||||||||||||||||||||
Seq_2  223   TGATGTCCGAAATCCAATCTGCCACATTAGTTTCCTTCATTATAGCTGAATACTTCAGCT  282


Seq_1  601   ACTCTAATTTGTGCGAACAATAGATTTTAAACTTTTTTTTTCCACCTGGATTTCTCCCCT  660
             |||||||||||||||||||||||||||||||||||||||| |||||||||||||||||||
Seq_2  283   ACTCTAATTTGTGCGAACAATAGATTTTAAACTTTTTTTT-CCACCTGGATTTCTCCCCT  341


Seq_1  661   TTCCATTTATGCAAACGGAAATGAAAACGAGAGGAGAAACATGGGCACAAACTAACCTAC  720
             ||||||||||||||||||||||||||||||||||||||||||||||||||||||||||| 
Seq_2  342   TTCCATTTATGCAAACGGAAATGAAAACGAGAGGAGAAACATGGGCACAAACTAACCTAG  401


Seq_1  721   TCTGGGCAGGAAACCCACAGACCGACCTCTGCCAATATATTTAAAACAGTAAACTGCGCA  780
             ||||||||||||||||||||||||||||||||||||||||||||||||||||||||||||
Seq_2  402   TCTGGGCAGGAAACCCACAGACCGACCTCTGCCAATATATTTAAAACAGTAAACTGCGCA  461


Seq_1  781   AAACCTTCTAGAGCATTTGATATGTTACTAAAACAGTAAATTGCGCAAAACCACAATGCC  840
             ||||||||||||||||||||||||||||||||||||||||||||||||||| ||||||||
Seq_2  462   AAACCTTCTAGAGCATTTGATATGTTACTAAAACAGTAAATTGCGCAAAACTACAATGCC  521


Seq_1  841   GTTGTACGGGCGTATTGTAGGCGTATTATAGACAGAACTACGGCAGTTAATTTGGATTGT  900
             ||||||||||||||||||||           |||||||||||||||||||||||||||||
Seq_2  522   GTTGTACGGGCGTATTGTAG-----------ACAGAACTACGGCAGTTAATTTGGATTGT  570


Seq_1  901   GGGATTTATTATTTTGCAAAACACTACTTTCTAAACGATTCGTACACTAACTACGACGAA  960
              |||||||||||||||||||||||||||||||||||||||||||||||||||||||||||
Seq_2  571   AGGATTTATTATTTTGCAAAACACTACTTTCTAAACGATTCGTACACTAACTACGACGAA  630


Seq_1  961   ACCATCACAGTATTCCATCCGTGGAAACAAAATTTTGCCAAGCAGGTAACACTCTTTCCA  1020
             ||||||||||||||||||||||||||||||||||||||||||||||||||||||||||||
Seq_2  631   ACCATCACAGTATTCCATCCGTGGAAACAAAATTTTGCCAAGCAGGTAACACTCTTTCCA  690
```

```
Seq_1  1021  CTTTCTTTCTTTTTTTTCGTCGTATCCCGAACTCAAAGCACGCATAGGCTACGTTTTTTT  1080
             ||||||||||        ||||||||| ||||||||||||||||||||| ||||||||
Seq_2  691   CTTTCTTTCT--------GTCGTATCCTGAACTCAAAGCACGCATAGGCTGCGTTTTTTC  742

Seq_1  1081  GACTTCTAGAAATCAGGTTTTCCAAAAAACTGT---------------------CTGCAC  1119
             | ||||||||||||||||| ||| ||||||||                      ||||||
Seq_2  743   GGCTTCTAGAAATCAGGTTCTCCGAAAAACTGCAGCACCAGCTTTCTAGATAAACTGCAC  802

Seq_1  1120  ACCAAATTTGGTTAGGTTTCCAAACTAGTTTAGATTAAACAGGTTTGAAAGCCAAACCAA  1179
             ||||||||||||||||||||||||||| ||||| ||||| ||||||||||||||||||||
Seq_2  803   ACCAAATTTGGTTAGGTTTCCAAACTAATTTAGGTTAAATAGGTTTGAAAGCCAAACCAA  862

Seq_1  1180  ATTTGGGATACAGCTTCTCTTG---------------------GAAGATGTGGCTT-GAA  1217
             || || ||||||||||||||||                     ||   |   |   |   |||
Seq_2  863   ATCTGAGATACAGCTTCTCTTGAAAGACGTGGCTTGTGAGGAGGACAACTTTTCAGAGAA  922

Seq_1  1218  GCCCTCAAGAAAACTGGCCATAATCATTTTCTCCACGCTCGACCTCTTCTAGGCCTCCGG  1277
             |||||||| |||||| ||||||||||||||||||||||||||||||||||||||||||||
Seq_2  923   GCCCTCAAAAAAACTTGCCATAATCATTTTCTCCACGCTCGACCTCTTCTAGGCCTCCGG  982

Seq_1  1278  TTTCCTTTGTCCGGCTGGCAAAAATCCTATACGACCTAGGGGGACACAATCAGCGGTGAA  1337
              ||||||||||||||||||| ||||||||||||||||||||||||||||||||||||||||
Seq_2  983   CTTCCTTTGTCCGGCTGGCACAAATCCTATACGACCTAGGGGGACACAATCAGCGGTGAA  1042

Seq_1  1338  CTCGTGTCCTAAACGTACC-----------------------------------------  1356
             |||||||||||||||||||
Seq_2  1043  CTCGTGTCCTAAACGTACCTCGATCTGAGCGTATGAAGATAGTCACCGGAGTTGAGATAT  1102

Seq_1  1357  ------------------------------------------------------------  1356

Seq_2  1103  GGTATTAGATTTAGAGATGCAACGAACGATGGCAGGGGTCTAGGGACGGATCGAGCAAGT  1162

Seq_1  1357  ------------------------------------------------------------  1356

Seq_2  1163  CTAGGTTGTATGTGATTTTCCATCTTGCTAGAGAAGCAGATGGCGTCCCACATGAGCTTG  1222

Seq_1  1357  ------------------------------------------------------------  1356

Seq_2  1223  CCCCCATCCCCCACCCCCCACGTACGCACACACACAATCGTATATGTTTCACTTATAGAT  1282

Seq_1  1357  ------------------------------------------------------------  1356

Seq_2  1283  GTAACTGTGATCTCAGATGAATAAAGAGGCTTTACGATGTAGAAAAGGAACCAACATGAG  1342

Seq_1  1357  ------------------------------------------------------------  1356

Seq_2  1343  AATATCGTGTGGTTCGGCTTACCTTGTGTTTCCTGAACCGGCTTTTCCGAGAAGCTGTCC  1402

Seq_1  1357  ---------------GGAAAAGTCGTCACGTAAATTTGATTAGGATAACAAATTAGTTTGC  1402
                              || || ||||||| |||||||||||||||||||||||||||||||
Seq_2  1403  TTTTCTAGTTTTTTTGAGAAATCGTCACCTAAATTTGATTAGGATAACAAATTAGTTTGT  1462

Seq_1  1403  CCTAAATTATTTTGTAAGTCTAACTAAATTTGTGATGTGGCTTTTGCAAGAAGTTAGGTG  1462
             |||||| ||||||||||||||||||||||||||||||||||||||||| |||||||||||||
Seq_2  1463  CCTAAACTATTTTGTAAGTCTAACTAAATTTGTGATGTGGCTTTTGTAAGAAGTTAGGTG  1522
```

```
Seq_1  1463  AGGACAGCTTGTACGAAAAGCCGGTTCTATGAGCCGAAAAGAACACAGTATATACCAGAG  1522
             ||||||||||||||||||| || || |||||||||||||| |||| ||||||||||||||
Seq_2  1523  AGGACAGCTTGTACGAAAAACCAGTCCTATGAGCCGAAAAAAACATAGTATATACCAGAG  1582


Seq_1  1523  TAGGCCTTAAACTTCTCCAGATTTAAAAGGGCCGAATAAGGAAATATGCTCACACAGCCT  1582
             ||||||||||||||||||||||||||||||||||||||||||||||||||||||||||||
Seq_2  1583  TAGGCCTTAAACTTCTCCAGATTTAAAAGGGCCGAATAAGGAAATATGCTCACACAGCCT  1642


Seq_1  1583  ATAACCAGGTTTAACAATCGAGAACAGAAAACCAGTCTCTTCATGATCTCGCGCAAATAA  1642
             |||||||||||||||||||||||||||||||||||| |||||||||||||||||||||||
Seq_2  1643  ATAACCAGGTTTAACAATCGAGAACAGAAAACCAGTTTCTTCATGATCTCGCGCAAATAA  1702


Seq_1  1643  TCCCGCTGAATTATATTAGTTCTTCCGTGCCATATAAGTACGGAGGGAATACATGCCTTC  1702
             ||||||||||||||||||||||||||||||||||||||||||||||||||||||||||||
Seq_2  1703  TCCCGCTGAATTATATTAGTTCTTCCGTGCCATATAAGTACGGAGGGAATACATGCCTTC  1762


Seq_1  1703  GTCATGTCCGTCCAGTTTGAAAGCACACTGAACTTTTCCCCTGCCCATCCTTTTTTTTTT  1762
             ||||||||||||||||||||||||||||||||||||||||||||||||||||||||||||
Seq_2  1763  GTCATGTCCGTCCAGTTTGAAAGCACACTGAACTTTTCCCCTGCCCATCCTTTTTTTTTT  1822


Seq_1  1763  -CAAAAAATGCCAGCATTCATAAGCTCGCAATGATATGAAATACAGAGTCGAGCCGTTCC  1821
              |||||||||||||||||||||||||||||||||||||||||||||||||||||||||||
Seq_2  1823  TCAAAAAATGCCAGCATTCATAAGCTCGCAATGATATGAAATACAGAGTCGAGCCGTTCC  1882


Seq_1  1822  AGAAGGCCCTCGCAAGCGGCCTGTATACGCGCGTGGGCTAGAAGAAAGGAGAAACGCCTG  1881
             ||||||||||||||||||||||||||||||||||||||||||||||||||||||||||||
Seq_2  1883  AGAAGGCCCTCGCAAGCGGCCTGTATACGCGCGTGGGCTAGAAGAAAGGAGAAACGCCTG  1942


Seq_1  1882  AAAAACAATTCCAGTCAGGGCCCAGGGCACCCAATCCACATGTGGAGTTTGGACACTTGC  1941
             |||||| |||||||||||||||||||||||||||||||||| ||||||||||||||||||
Seq_2  1943  AAAAACGATTCCAGTCAGGGCCCAGGGCACCCAATCCACATTTGGAGTTTGGACACTTGC  2002


Seq_1  1942  CTGAGAGGCTCCCAAAGTTTCTTTTTTTTT----AAAAAGGCTCCCAAAAGATCCCCTCC  1997
             |||||||||||||||||||||||||   |     ||||||||||||||||||||||||||
Seq_2  2003  CTGAGAGGCTCCCAAAGTTTCTTTTA--TAAAAAAAAAAGGCTCCCAAAAGATCCCCTCC  2060


Seq_1  1998  GCCTCCGTCCTCGAGCGAGCCAACGCAGCCTCGCTTTTCGCCGCTCTCCGACCGCTCGAC  2057
             ||||||||||||||||||||||||||||||||||||||||||||||||||||||||||||
Seq_2  2061  GCCTCCGTCCTCGAGCGAGCCAACGCAGCCTCGCTTTTCGCCGCTCTCCGACCGCTCGAC  2120


Seq_1  2058  GCACGTTATCC  2068
             |||||||||||
Seq_2  2121  GCACGTTATCC  2131
```

BdindelWSU_21, UPSTREAM

>Bradi5g17540

CACAAAGAGAGAATTATAAGAGCGAATCAAATGCATGAAATTCCTAGACCGCCTCCCACCACAAAAAGAAGCCAGCTCCCGCAAAGG
CGAAGGCCACTCTCTATACTTAGGCAGACATATACGGCCACCCCGGCAACACAAATTATAAACCGCATTCCCTTCAATACTGCTCGAC
ACCACCCTCTCAGAAAACCAAAACAATGCACCACAAAATAGGCACACATAATCCATCCCACCATGGTACGACCTTCCATAATAATCAA
CTGAAAAAAGGAGAAGGAAAAAAAGCAAAAAAGAAAAAAGAAAAAAAGAAGAAGTTACAAACCACATTTATACAGAAAGTAAAAG
TAAAGAGGAAGAAATTTTAAGTCGAGAGTCACTCCAAACACCAAATCAAACCTTTCAAAAAAGCCACAAACTCTTTGGACAGAGTAT
ACCCTTCGCCAGCAGACACCCAGCACGCCAACGTTTCTACCCACCAAACAAAACACACATCATACAACGTATAAATAGCATACAAAAT
ACAGAAGAAAAAAAAACAAAAATAAACTCGTCTTTTACCAAACAATCCAGACCGCTGTTGCACACACCTTCTCTTTCTTTTGGGTCTAG

CCTCCGACGCACTAAGATGCGGCAATATAAGGTTGCCTACGCCCCAATCAAATAGCACGTGAGAAGGAGCGAACACACGAAACAATT
GAAAAAGAAACAAAAGGAGAACCAAAAGCAAGACAATACCACTGAAATAACCACGAACAACTGGCACCACGGGAGGCAAGTCAAC
GACGGGCCCCGCAGAAGAAGAAGCGGCTACCGAATCGACAACACGAGACACAGCAGCACCTAATATAAAACACCAGATCATAAGAC
AGAGACACACGCCCCAAAAAATACAAATAACTAAAGGAAGAACACACGCACTTAGAGAATGAACAGCTCGAGTAGCCAACAAACCA
CGCCCCCTCCCGCGACGACCACGCGTTCCGGACGACCCATCCACAGCAGCATCAACACCACGGCCCCCAACAACGGACGATCCTCGT
CGCCTTCCACTACGACCGCATCCCCGAGGCAACCCATCCGCAGACACACCCTCACAATGCTCCCCAACAATCGAGGACACACGACCAC
CTCCATGACGCCGCCGCCGCCCAGACGGCTCATCCGCGAAATCATCACCAGCAGATCTCCCTCTCACACGATGCCCACGACTACCGGA
TGGCACATCCGCATGCAGATCCAAGCCACCACGCAGCCCATCCGCGGACGCACCATCACAACGCCCACCAGCAACCGAGGACACCCG
GCCACCTCCAAGGCGACAGCGCCGTCCAGACGGCTCATCCAAGGAAGCACCACCAACAGATCTCCCCCTCACACGACGCCCACGACT
ACTGGAAGGGACATCTGCATGCAGGTCCAAACCACCACGTCTGGCCTCACGTCGGCCAGGACAGCCGGTAGCGAACACGTCCATCA
ACGCACGGCAACCCAAAAAAAGTAACACCTAGCAAAACACAATACAAAATAAACTAAAAAACAAGGGAGATAAGAGATGTATCTTG
GTACGCCGAAATAAAAAATACCACGAAGCAAAACCAAATAATTTTTTTAAAGAGATGCAAGAGATACTCAACAAACACGACCCTAG
GGGCCCCACCGTCAGCGAGAAAACTAACACAGCACCCGCCACCCTGGCAAAACCAGAAAAACATCCGCGTCAGTACAAAACGCTGA
CGTGGATCGCTGAGAAGCCGAATTATCATGCGGCTATGGTAGTTGTATCTTACTAGGCAGGCGAGCTGTGTGGCGTGTCCTCGGTTG
ATGGCGCTCCCCTGGTCAAAAAGTCAACCGTTCGGCACCTCACAGGATGGACTTTTCGTCGAGGAACGGCTATGGGCCTCGTGAACG
CCTCTGCAATGGGCTTCATCCACGATCCAGCCCATAACTTTTCTTATGTTTTCGACAACCACAAAACCGAACACTTCCTTCCTGCCTCCA
GCGTATCCTTCTCCCCGAGCTACTCGGCGCCGCCTCCCTACCGCCGCGCAGGGTTTTAGGGTTTAGCGGCGGCGACACCATCCCCACA
GCACCAGGCG

>BdiBd21-3.5G0229800

AGCCATTCGGAAGCAATCCGAAAACTCCGCACCAACACATTATGCTGATTCAACATGGCAATCAACGATGCCACTATTTCCGGATCGG
GAGCAGCACGAGTATCACCATCACATCCAAACACGCCTAACCGATTGGCGAGCTCATTCGCTACATCGTACACATAAAGCTGTGCAAA
TTGAGGGGCGTTGCCATCCACAGGCACCAAAGAACCAATCCGATGATGCACCTTGCCGTGTACACGAAACACGTATGGACCACTTCC
TGTGTTAATGCTCTTATCCACGTTAACCCCAAGTGAAGTAAACGCAAAGAGAGAATTATAAGAGCGAATCAAATGCATGAAATTCCTA
GACCGCCTCCCACCACAAAAAGAAGCCAGCTCCCGCAAAGGCGAAGGCCACTCTCTATACTTAGGCTGACATACACGGCCACCCCGG
CAACACAAATTATAAACCGCATTCCCTTCAACACTGCTCGACACCACCCTCTTAGAAAACCAAAACAATGCACCACAAAATAGACACA
CATAATCCATCCCACCATGGCACGACCTTCCATAGTAATCAACTGAAAAAAGGAGAAGGAAAAAAAGCAAAAAAGCAAAAAGAAAA
AAAGAAGTTACAAACCACATTTATACAGAAAGTAAAAGTAAAGATGAAGAAATTTTAAGTTGAGAGTCGCTCCAAACACCAAAACAA
ACCTTTCAAAAAAGCCACAAACTCTTTGGACAGAGTATACCCTTCGCCAACAGACACCCAGCACGCCAATGTTTCTACCCACCAAACA
AAATACACATCATACAACGTATAAATAGCATACAAAATACAAAAAAAAACCCGAAAATAAACTCGTCTTTTACCAAACAATCCAGACC
GCTGTTGCATACACCTTCTCTTTCTTTTGGGTCTAGCCTCCGACGCACTAAGATGCGGCAATATAAGGTTGCCTAGGCCCCAATCAAAT
AGCACGTGAGAAGGAGCGAACACACGAAACAATTGAAAATGAAACAAAAGGAGAACCAAAAGCAAGACAATACCAATCAAATAAC
CACGAACAACTGGCACCACGGGAGGCAAGTCAACGACGGGCCCCGCAGAAGAAGAAGCGGCTACCGAATCGACAACATGAGACAC
AGCAGCACCTAATATAAAACACCAGATCATAAGACAGAGACACACGCCCCAAAAAATACAAATAACTGAAGGAAGAACACACGCAC
TTCGAGAATGAACAGCTCGACGACGCCCACGTCCTCGAGTAGCCAACAAACCACGGGCCCTCCCGCGACGACCACGCGTTCCGGACG
ACCCATCCACAGCAGCATCAACACCACGGCCCCCAACAACGTACGATCCTCGTCGCCTTCCACTACGACCGCATTCCCGAGGCAACCC
ATCCGCAGACACACCCTCACAACGCCCCCCAACAACCGAGGACACACGACCACCTCCACGACGACGACGCCGCCCAGACGGCTCATC
CGCGAAATCATCACCAGCAGATCTCCCTCTCACACGACGCCTACGACTACCGGATGGCACATCCGCATGCAGATCCAAGCCACCACGC
AGCCCATACTCTAGAAAACTCCAAACGACAGCACAGAGGACACGCGCCGTCATGCATGAACCACAACACACAGCACTCACGCTGACA
ATGGGCCTCATGCAAACCGACCAACTCTATTGAGCAGACACGTACCCCTCCCGCACACCAGTTCTTGCTGATCAACAACCTCTCACTGA
CAACAACGACCCCAACAAACACGACCCCACCGTCAGCGAGAAAACCAACACAGCACCCGCCACCCTCGCAAAACCAGAAAAACATCC
GCGTCAGTACAAAACGCTGAGAAGCCGAATTATCATGCGGCTATGGTGGCAGGCGAGCTGTGTGGCGTGTCCTCGGTTGATGGCGC
TCCCCTGGTCAAAAAGTCAACCGTTCGGCACCTCACAGGATGGACTTTTCGTCGAGGAACGGCTATGGGCCTCGTGAACGCCTCTGC
AATGGGCTTCATCCACGATCCAGCCCATAACTTTTCTTATGTTTTCGACAACCACAAAACCGAACACTTCCTTCCTGCCTCCAGCGTATC
CATCTCCCCGAGCTACTCGGCGCCGCCTCCCTACCGCCGCGCAGGGTTTTAGGGTTTAGCGGCGGCGACACCATCCCCACAGCACCA
GGCG

```
Alignment of Sequence_1:  [Untitled Sequence #1] with  Sequence_2: [Sequence Window #2]

Similarity : 1696/2187 (77.55 %)
```

```
Seq_1  1     ---CA-------------------------------------------------------  2
                ||
Seq_2  1     AGCCATTCGGAAGCAATCCGAAAACTCCGCACCAACACATTATGCTGATTCAACATGGCA  60

Seq_1  3     ------------------------------------------------------------  2

Seq_2  61    ATCAACGATGCCACTATTTCCGGATCGGGAGCAGCACGAGTATCACCATCACATCCAAAC  120

Seq_1  3     ------------------------------------------------------------  2

Seq_2  121   ACGCCTAACCGATTGGCGAGCTCATTCGCTACATCGTACACATAAAGCTGTGCAAATTGA  180

Seq_1  3     ------------------------------------------------------------  2

Seq_2  181   GGGGCGTTGCCATCCACAGGCACCAAAGAACCAATCCGATGATGCACCTTGCCGTGTACA  240

Seq_1  3     ------------------------------------------------------------  2

Seq_2  241   CGAAACACGTATGGACCACTTCCTGTGTTAATGCTCTTATCCACGTTAACCCCAAGTGAA  300

Seq_1  3     -------CAAAGAGAGAATTATAAGAGCGAATCAAATGCATGAAATTCCTAGACCGCCTC  55
                    |||||||||||||||||||||||||||||||||||||||||||||||||||||
Seq_2  301   GTAAACGCAAAGAGAGAATTATAAGAGCGAATCAAATGCATGAAATTCCTAGACCGCCTC  360

Seq_1  56    CCACCACAAAAAGAAGCCAGCTCCCGCAAAGGCGAAGGCCACTCTCTATACTTAGGCAGA  115
             ||||||||||||||||||||||||||||||||||||||||||||||||||||||||| ||
Seq_2  361   CCACCACAAAAAGAAGCCAGCTCCCGCAAAGGCGAAGGCCACTCTCTATACTTAGGCTGA  420

Seq_1  116   CATATACGGCCACCCCGGCAACACAAATTATAAACCGCATTCCCTTCAATACTGCTCGAC  175
             |||| |||||||||||||||||||||||||||||||||||||||||||| ||||||||||
Seq_2  421   CATACACGGCCACCCCGGCAACACAAATTATAAACCGCATTCCCTTCAACACTGCTCGAC  480

Seq_1  176   ACCACCCTCTCAGAAAACCAAAACAATGCACCACAAAATAGGCACACATAATCCATCCCA  235
             |||||||||| |||||||||||||||||||||||||||||| ||||||||||||||||||
Seq_2  481   ACCACCCTCTTAGAAAACCAAAACAATGCACCACAAAATAGACACACATAATCCATCCCA  540

Seq_1  236   CCATGGTACGACCTTCCATAATAATCAACTGAAAAAAGGAGAAGGAAAAAAAGCAAAAAA  295
             |||||| ||||||||||||| |||||||||||||||||||||||||||||||||||||||
Seq_2  541   CCATGGCACGACCTTCCATAGTAATCAACTGAAAAAAGGAGAAGGAAAAAAAGCAAAAAA  600

Seq_1  296   GAAAAAAGAAAAAAAGAAGAAGTTACAAACCACATTTATACAGAAAGTAAAAGTAAAGAG  355
             | |||||||||||||||||   ||||||||||||||||||||||||||||||||||||| 
Seq_2  601   GCAAAAAGAAAAAAAGAAG---TTACAAACCACATTTATACAGAAAGTAAAAGTAAAGAT  657

Seq_1  356   GAAGAAATTTTAAGTCGAGAGTCACTCCAAACACCAAATCAAACCTTTCAAAAAAGCCAC  415
             ||||||||||||||| ||||||| |||||||||||||| |||||||||||||||||||||
Seq_2  658   GAAGAAATTTTAAGTTGAGAGTCGCTCCAAACACCAAAACAAACCTTTCAAAAAAGCCAC  717

Seq_1  416   AAACTCTTTGGACAGAGTATACCCTTCGCCAGCAGACACCCAGCACGCCAACGTTTCTAC  475
             ||||||||||||||||||||||||||||||| ||||||||||||||||||| ||||||||
Seq_2  718   AAACTCTTTGGACAGAGTATACCCTTCGCCAACAGACACCCAGCACGCCAATGTTTCTAC  777

Seq_1  476   CCACCAAACAAAACACACATCATACAACGTATAAATAGCATACAAAATACAGAAGAAAAA  535
             ||||||||||||| |||||||||||||||||||||||||||||||||||||     ||||
Seq_2  778   CCACCAAACAAAATACACATCATACAACGTATAAATAGCATACAAAATACA-----AAAA  832
```

```
Seq_1  536   AAAACA--AAAATAAACTCGTCTTTTACCAAACAATCCAGACCGCTGTTGCACACACCTT  593
             |||||   |||||||||||||||||||||||||||||||||||||||||||| |||||||
Seq_2  833   AAAACCCGAAAATAAACTCGTCTTTTACCAAACAATCCAGACCGCTGTTGCATACACCTT  892

Seq_1  594   CTCTTTCTTTTGGGTCTAGCCTCCGACGCACTAAGATGCGGCAATATAAGGTTGCCTACG  653
             |||||||||||||||||||||||||||||||||||||||||||||||||||||||||| |
Seq_2  893   CTCTTTCTTTTGGGTCTAGCCTCCGACGCACTAAGATGCGGCAATATAAGGTTGCCTAGG  952

Seq_1  654   CCCCAATCAAATAGCACGTGAGAAGGAGCGAACACACGAAACAATTGAAAAAGAAACAAA  713
             ||||||||||||||||||||||||||||||||||||||||||||||||||| ||||||||
Seq_2  953   CCCCAATCAAATAGCACGTGAGAAGGAGCGAACACACGAAACAATTGAAAATGAAACAAA  1012

Seq_1  714   AGGAGAACCAAAAGCAAGACAATACCA--CTGAAATAACCACGAACAACTGGCACCACGG  771
             |||||||||||||||||||||||||||  |  ||||||||||||||||||||||||||||
Seq_2  1013  AGGAGAACCAAAAGCAAGACAATACCAATC--AAATAACCACGAACAACTGGCACCACGG  1070

Seq_1  772   GAGGCAAGTCAACGACGGGCCCCGCAGAAGAAGAAGCGGCTACCGAATCGACAACACGAG  831
             |||||||||||||||||||||||||||||||||||||||||||||||||||||||| |||
Seq_2  1071  GAGGCAAGTCAACGACGGGCCCCGCAGAAGAAGAAGCGGCTACCGAATCGACAACATGAG  1130

Seq_1  832   ACACAGCAGCACCTAATATAAAACACCAGATCATAAGACAGAGACACACGCCCCAAAAAA  891
             ||||||||||||||||||||||||||||||||||||||||||||||||||||||||||||
Seq_2  1131  ACACAGCAGCACCTAATATAAAACACCAGATCATAAGACAGAGACACACGCCCCAAAAAA  1190

Seq_1  892   TACAAATAACTAAAGGAAGAACACACGCACTTAGAGAATGAACAGCTCGA----------  941
             ||||||||||| |||||||||||||||||||| |||||||||||||||||
Seq_2  1191  TACAAATAACTGAAGGAAGAACACACGCACTTCGAGAATGAACAGCTCGACGACGCCCAC  1250

Seq_1  942   --------GTAGCCAACAAACCACGCCCCCTCCCGCGACGACCACGCGTTCCGGACGACC  993
                     |||||||||||||||||  |||||||||||||||||||||||||||||||||
Seq_2  1251  GTCCTCGAGTAGCCAACAAACCACGGGCCCTCCCGCGACGACCACGCGTTCCGGACGACC  1310

Seq_1  994   CATCCACAGCAGCATCAACACCACGGCCCCCAACAACGGACGATCCTCGTCGCCTTCCAC  1053
             |||||||||||||||||||||||||||||||||||||| |||||||||||||||||||||
Seq_2  1311  CATCCACAGCAGCATCAACACCACGGCCCCCAACAACGTACGATCCTCGTCGCCTTCCAC  1370

Seq_1  1054  TACGACCGCATCCCCGAGGCAACCCATCCGCAGACACACCCTCACAATGCTCCCCAACAA  1113
             ||||||||||| ||||||||||||||||||||||||||||||||||| || |||||||||
Seq_2  1371  TACGACCGCATTCCCGAGGCAACCCATCCGCAGACACACCCTCACAACGCCCCCCAACAA  1430

Seq_1  1114  TCGAGGACACACGACCACCTCCATGACGCCGCCGCCGCCCAGACGGCTCATCCGCGAAAT  1173
              |||||||||||||||||||||| |||| || ||||||||||||||||||||||||||||
Seq_2  1431  CCGAGGACACACGACCACCTCCACGACGACGACGCCGCCCAGACGGCTCATCCGCGAAAT  1490

Seq_1  1174  CATCACCAGCAGATCTCCCTCTCACACGATGCCCACGACTACCGGATGGCACATCCGCAT  1233
             ||||||||||||||||||||||||||||| ||| ||||||||||||||||||||||||||
Seq_2  1491  CATCACCAGCAGATCTCCCTCTCACACGACGCCTACGACTACCGGATGGCACATCCGCAT  1550

Seq_1  1234  GCAGATCCAAGCCACCACGCAGCCCATCCGCGGACGCACCATCACAACGCCCACCAGCAA  1293
             |||||||||||||||||||||||||||                               |
Seq_2  1551  GCAGATCCAAGCCACCACGCAGCCCAT-------------------------------AC  1579

Seq_1  1294  CCGAGGACACCCGGCCACCTCCAAGGCGACAGCGCCGTCCAGACGGCTCATCCAAGGAAG  1353
              | | || |  |  |||   | |  ||      | |   |       |     ||  |
Seq_2  1580  TCTA-GAAAA-CT-CCAAA-CGACAGCACAGAGGACACGCGCCGTCATGCATGAACCACA  1635
```

```
Seq_1  1354  CACCACCAACAGATCTCCCCCTCACACGACGCCCACGACTACTGGAAGGGACATCTGCAT  1413
                |||        | |   |                ||      |    |
Seq_2  1636  ACACACAGCACTCACGCTGACAATGGGCCTCATGCAAACCGACCAACTCTATTGAGCAGA  1695

Seq_1  1414  GCAGGTCCAAACCACCACGTCTGGCCTCACGTCGGCCAGGACAGCCGGTAGCGAACACGT  1473
                |  ||   ||  |||  | |  ||  |           |   |  |  | |  |||
Seq_2  1696  CACGTACCCCTCCCGCACACCAGTTCTTGCTGATCAACAACCTCTCACTGACAACAACGA  1755

Seq_1  1474  CCATCAACGCACGGCAACCCAAAAAAAGTAACACCTAGCAAAACACAATACAAAATAAAC  1533
             ||
Seq_2  1756  CCC---------------------------------------------------------  1758

Seq_1  1534  TAAAAAACAAGGGAGATAAGAGATGTATCTTGGTACGCCGAAATAAAAAATACCACGAAG  1593

Seq_2  1759  ------------------------------------------------------------  1758

Seq_1  1594  CAAAACCAAATAATTTTTTTAAAGAGATGCAAGAGATACTCAACAAACACGACCCTAGG  1653
                                                      |||||||||||||||
Seq_2  1759  -----------------------------------------CAACAAACACGACCC----  1773

Seq_1  1654  GGCCCCACCGTCAGCGAGAAAACTAACACAGCACCCGCCACCCTGGCAAAACCAGAAAAA  1713
                  |||||||||||||||||| |||||||||||||||||||| |||||||||||||||
Seq_2  1774  -----CACCGTCAGCGAGAAAACCAACACAGCACCCGCCACCCTCGCAAAACCAGAAAAA  1828

Seq_1  1714  CATCCGCGTCAGTACAAAACGCTGACGTGGATCGCTGAGAAGCCGAATTATCATGCGGCT  1773
             |||||||||||||||||||||||||             ||||||||||||||||||||||
Seq_2  1829  CATCCGCGTCAGTACAAAACGCTGA-------------GAAGCCGAATTATCATGCGGCT  1875

Seq_1  1774  ATGGTAGTTGTATCTTACTAGGCAGGCGAGCTGTGTGGCGTGTCCTCGGTTGATGGCGCT  1833
             |||||               ||||||||||||||||||||||||||||||||||||||||
Seq_2  1876  ATGGT---------------GGCAGGCGAGCTGTGTGGCGTGTCCTCGGTTGATGGCGCT  1920

Seq_1  1834  CCCCTGGTCAAAAAGTCAACCGTTCGGCACCTCACAGGATGGACTTTTCGTCGAGGAACG  1893
             ||||||||||||||||||||||||||||||||||||||||||||||||||||||||||||
Seq_2  1921  CCCCTGGTCAAAAAGTCAACCGTTCGGCACCTCACAGGATGGACTTTTCGTCGAGGAACG  1980

Seq_1  1894  GCTATGGGCCTCGTGAACGCCTCTGCAATGGGCTTCATCCACGATCCAGCCCATAACTTT  1953
             ||||||||||||||||||||||||||||||||||||||||||||||||||||||||||||
Seq_2  1981  GCTATGGGCCTCGTGAACGCCTCTGCAATGGGCTTCATCCACGATCCAGCCCATAACTTT  2040

Seq_1  1954  TCTTATGTTTTCGACAACCACAAAACCGAACACTTCCTTCCTGCCTCCAGCGTATCCTTC  2013
             ||||||||||||||||||||||||||||||||||||||||||||||||||||||||| ||
Seq_2  2041  TCTTATGTTTTCGACAACCACAAAACCGAACACTTCCTTCCTGCCTCCAGCGTATCCATC  2100

Seq_1  2014  TCCCCGAGCTACTCGGCGCCGCCTCCCTACCGCCGCGCAGGGTTTTAGGGTTTAGCGGCG  2073
             ||||||||||||||||||||||||||||||||||||||||||||||||||||||||||||
Seq_2  2101  TCCCCGAGCTACTCGGCGCCGCCTCCCTACCGCCGCGCAGGGTTTTAGGGTTTAGCGGCG  2160

Seq_1  2074  GCGACACCATCCCCACAGCACCAGGCG  2100
             |||||||||||||||||||||||||||
Seq_2  2161  GCGACACCATCCCCACAGCACCAGGCG  2187
```

BdindelWSU_22, UPSTREAM

> Bradi5g22050

TTTTGGGCGGTGGAGTGAGTGTGAAGGTACTAGCTAGAATTAACGACCCCACTGCCATTGCCATTTTGCTCGGGCCGAAAAACGGAG
AGAAGAGACGAACGAGTCCCCCACCGCGCGCATGCAGTAGGTGGGCGTGGCTCGGCATGTGAGCCTTTTACCAACCTCTGTCGGCTT
GCTCTCACATCCACTGCTTAGCTAAGGATGCTACTACCTAGTCTTAGAGTACTGCTGCTAACTAATCCAATTATCCAGTCCACCCGTGC
CAAGATGCTGCTGTGCCTGTGCTGCTGGTATTTTACTGTTGCAGTGCTCCTGTAGACTGTGGACTGTAGTATGTACTCTTCCCCGGTTC
TCTTGGGCTTGGGGTCGGGTTTATTTATGTACCGATCCCCGCGCTTCACGGGCAAGCGAATGACGCCGGCCGGGGCGCACCGGTGTC
GCATGCGTCGTCTCGGCACGCAGATGCGTCCCATTCCCAACACGCTTCCGTGCCTCCGTCCCATGGCGTTTGCCCGTCAAATCACGGA
ACTCGCCCATCCATTCCCGACGGAGATGGGGCTTTACAATGCTGGCACGGGCCTTGGTTGTTGGACGAACAAAAACGGAGAGGCAA
TTCTCGATTCTATTAATTCGGCCGGTGTTTTTAGGTGAGAGTGATAGCACTAGTATTAACACGTATACTGGTGGATCGGGGGCGGGG
GATGGAATCTAATGGCGTACGTCCCCAGGTCTGGTCCGGTACTTGGATCTCCAGCGACGTTGTACAGATGGCACCCAGTCGGCCAGT
CCATTTGGCCTGGCACGACGTACGTACGCACGCCATGCATGGGCATGGTGGCCTGACGATTCGTTGGCAATGCTTTGTACGCTACTCC
TACCTAACCCGATAAAACCATAAAGGTCATCATCATAAACAAAAGGCCATCGATCATGCCGTGACACTATACACTGGAGTACTAGTAG
TACTATCTCGTTTCTGTACTGGCCGTCAGGAAAGAAAGATCTCATTCCCGTACTGAATGACTGTAATCAGCACGCTCGGAGTTTTCCG
AGCTTCTGCAGACACGGAAATTGGATTGCTAGAAATCAGCACGCTCGGAGTTTAACTCTCGAAATTAAGTAGGTTCGGAGGAAGAG
GTTACCTCGGTCCAAAGCACTTTTGGTGCGCTGAAAATTGTCGGCTGTTTGGATCGGACTTAAATAAAATGATATCCGGTGCGGTCTA
TTCTCTCTCACGACCAAACCATGAAGCTACCGTGCACCGCTAGTAGTACTAGTGTCACCCCCCTGACCTGACTCCCGACCAAGTGTGAT
GCAGCCTGCTTTCCCGTCCATTCAGCCGTGCATGTTGCTTTAGCCGATGAGACAACACCTGTCTGTACGGGGATCTGACCGTGACAGT
GAGGTAGCACCGGTTGATGATTGGGGTTGGTACGTCCTAGGACGCTTTTAAAGGACGACGATTTTTGGTCCCATCCATCCAGCCAGC
TCGATGCTCCGTCCGTGACAGTGTTACGACTTGACTGTAAACGGCAGGTCTTCCTCGTCGGTCGGTCGGTCAGTGCGATGTGGTGTTT
CGACACGCACGCGCGCTGTGCAGCAGGCTGCTATGCTACACTTGCAGCACGAGAGGCCTCGCGGCGCCTTTCGTTGCCGGATCTCGG
TGGATCGCCCCTCCCAAAACAAGGAGGACTCCCTCAGTGCATGCCACAACCACCCCGTAATTAGGCCGGAATACCAAGGAAGAGTTT
GGACGAAACGGAAGGCCCAAGCTTGATAGCGACTATGCGCATTGATCGGCCTTGTCAGCAGCTAGCAGCCTGGCCCAGAGAAGAAC
AAAGCAGATGGATTCCCACTCGCCCGGCCCGTATCGATCGGCCCATTCAAGAAGCCAGAAAAAGGAAGCAGCAAGCACGAGCCCCT
TGCGTTGGTAGGCACCGACGGTCCAGATCGGGAGCAAGGACACATCCGGACCGTTCGTTCCTGCGCCAGCACGGGCGGTGCAGACG
AAGCGTCCCGTCTCTTGGCTCCCAGCGGCCGCAATTTTCCACGAGGAGAAAAAAAGGGGTAGAAGGGGAAGTTTCCAAGCGAAATC
GAGATCGGAGGCACAGCTAGAAGGTGATTTCTTCCGCTCAATCTCTTGTTCCGTACTATTAGTTTGATTAGCTAATTTCACTAGGCATT
CTGGTGCGCGATGTTTAGCCATGCCTCCTCGATTCGATAGGAACTTGCGGTTTCTTCTGCCGTTCTGTCGCCTTGCAGTACTATTTGCT
CGTCAAAGGATGCATTTTTTTTATTTTGTTGTTGGTGTCTTCGATGATGTAGAGAGCTAAAACAGCGGTTTCCCGCGACTCCTCGGCGT
AGGTTATTGGATTTGGTTGCGACTTAGGGTTTCGCGTCTGTATTGGCCCAGAATCGTTGGATCTGTTCCTCATGTTTCGACTAGGTTTG
CTTGATTTCATGGCCTTGTCTGCTTAATTGGTCGAATTGATATAAAATCTATGCTGCTCGCTTTGTATAGTCTGCGGTACTCTATGCTTC
AATTGAAGCCAATTTTCAGCTTATCTTTCATGCTTGGTTCAGGAGTTGCCTCTTGTTGTGCAGGTTGATGCTTTTAATTTCGGTGCAAA
AGTAGAGGGTGATTTACTCTTTTGAACAAACCTGGTCTATCTTGATCTTATTACAAGTTTCATCTTCAACTGAAAATCTGGTTACAGGT
TTGGAGCTTAATCCAAATCTGCTCCTTTGCAAATTTAGGTTTCTGATTTTATATACATAAATAAGTGCATCGTAGTTGTTGTTCAGACTC
CTTCTACATGCGCTGAACAATTACTGTGTTGTATTCCTTGGAGTGACTAAGTGTCCGTTATTTTTTTTCCCAGTAATGCTTACACGAACA
TACAGTTTTTTTAGTGCAATTTGAGCTGAAGAGTTTGATTATTGCATTGCAGGTATTTGTATTTTTGTTTGCCTGGTTTGAGGGAATTG
CACTCGGAAGTTCACCCCAAGATCGGATTCAAATTTTT

> BdiBd21-3.5G0289000

GTATTTTACTGTTGCAGTGCTCCTGTAGACTGTGGACTGTAGTATGTACTCTTCCCCGGTTCTCTTGGGCTTGGGGTCGGGTTTATTTA
TGTACCGATCCCCGCGCTTCACGGGCAAGCGAATGACGCCGGCCGGGGCGCGCCGGTGTCGCATGCGTCGTCTCGTCTGCTTCGATC
GTCTCGGCACGCAGATGCATCCCATTCCCAACACGCTTCCGTGCCTCCGTCCCATGGCGTTTGCCCGTCAAATCACGGAACTCGCCCAT
CAATTCCCGACGGAGATGGGGCTTTACAATGCTGGCACGGACCTTGGTTGTTGGACGAACAAAAACGGAGAGGCAATTCTCGATTCT
ATTAATTCGGCCGGTGTTTTTAGGTGAGAGTGATAGCACTAGTATTAACACGTATACTGGTGGATCGGGGGCGGGGGATGGAATCT
AATGGCGTACGTCCCCAGGTCTGGTCCGGTACTTGGATCTCCAGCGACGTTGTACAGATGGCACCCAGTCGGCCAGTCCATTTGGCCT
GGCACGACGTACGTACGCACGCCATGCATGGGCATGGTGGCCTGACGATTCGTTGGCGATGCTTTGTACGCTACTCCTACCTAACCC
GATAAAACCATAAAGGTCATCATCATAAACAAAAGGCCATCGATCATGCCGTGACACTATACACTGGAGTACTAGTAGTACTATCTCG
TTTCTGTACTGGCCGTCAGGAAAGAAAGATCTCATTCCCGTACTGAATGACTGTAATCAGCACGCTCGGAGTTTTCCGAGCTTCTGCA
GACACGGAAATTGGATTGCTAGAAATCAGCACGCTCGGAGTTTAACTCTCGAAATTAAGTAGGTTCGGAGGAAGAGGTTACCTCGG
CCCAAAGCACTTTTGGTGCGCTGAAAATTGTCGGCTGTTTGGATCGGACTTAAATAAAATGATATCCGGTGCGGTCTATTCTCTCTCAC

GACCAAACCATGAAGCTACCGTGCACCGCTAGTAGTACAAGTATCACCCCCCTGACCTGACTCCCGACCAAGTGTGATGCAGCCTGCT
TTCCCGTCCATTCAGCCGTGCATGTTGCTTTAGCCGATGAGACAACACCTGTCTGTACGGGGATCTGACCGTGACAGTGAGGTAGCAC
CGGTTGATGATTGGGGTTGGTACGTCCTAGGACGCTTTTAAAGGACGACGATTTTTGGTCCCATCCATCCAGCCAGCTCGATGCTCCG
TCCGTGACAGTGTTACGACTTGACTGTAAACGGCAGGTCTTCCTCGTCGGTCGGTCGGTCAGTGCGATGTGGTGTTTCGACACGCAC
GCGCGCTGTGCAGCAGGCTGCTATGCTACACTTGCAGCACGAGAGGCCTCGCGGCGCCTTTCGTTGCCGGATCTCGGTGGATCGCCC
CTCCCAAAACAAGGACGACTCCCTCAGTGCATGCCACAACCACCCCGTTAATTAGGCCCAAGTGTCGATGACAATCCGTCCACGTCGA
ATCGAGGGCGATCAATCCAGATCCAGAGTGGGCTAGGACATCAAAGACTCCCCGATGTCATCCAAGAATACCGCATTAAGACGGGA
ATTCAACCGTGACACTTTTATATTAGATGGAAACCCTTTTTATCAGCGAGCGGCCTCTACACCATCCTAAACATAGGCAAAACTGTACT
ATACTGGTAACAGAGCTTTCGTGACTCGTCTTCTCTGCAGAGTGCAGACCCCGTAATTAGGCCGGAATACCAAGGAAGAGTTTGGAC
GAAACGGAAGGCCCAAGCTTGATAGCGACTAGGCGCATTGATCGGCCTTGTCAGCAGCTAGCAGCCTGGCCCAGAGAAGAACAAAG
CAGATGGATTCCCACTCGCCCGGCCCGTATCAATCGGCCCATTCAAGAAGCCAGAAAAAGGAAGCAGCAAGCACGAGTCCCTTGCGT
TGGTAGGCACCGACGGTCCAGATCGGGAGCAAGGACACATCCCGACCGTTCGTTCCTGCGCCAGCACGGGCGGTGCAGACGAAGC
GTCCCGTCTCTTGGCTCCAAGCGGCCGCAATTTTCCACGAGGAGAAAAAAAGGGGTAGAAGGGGAAGTTTCCAAGCGAAATCGAGA
TCGGAGGCACAGCTAGAAGGTGATTTCTTCCGCTCAATCTCTTGTTCCGTACTATTAGTTTGATTAGCTAATTTCACTAGGCATTCTGG
TGCGCGATGTTTAGCCATGCCTCCTCGATTCGATAGGAACTTGCGGTTTCTTCTGCCGTTCTGTCGCCTTGCAGTACTATTTGCTCGTC
AAAGGATGCATTTTTTTTATTTTGTTGTTGGTGTCTTCGATGATGTAGAGAGCTAAAACAGCGGTTTCCCGCGACTCCTCGGCGTAGG
TTATTGGATTTGGTTGCGACTTAGGGTTTCGCGTCTGTATTGGCCCAGAATCGTTGGATCTGTTCCTCATGTTTCGACTAGGTTTGCTT
GATTTCATGGCCTTGTCTGCTTAATTGGTCGAATTGATATAAAATCTATGCTGCTCGCTTTGTATAGTCTGCGGTACTCTATGCTTCAAT
TGAAGCCAATTTTCAGCTTATCTTGCATGCTTGGTTCAGGAGTTGCCTCTTGTTGTGCAGGTTGATGCTTTTAATTTCGGTGCAAAAGT
AGAGGGTGATTTACTCTTTTGAACAAACCTGGTCTATCTTGATCTTATTACAAGTTTCATCTTCAACTGAAAATCTGGTTACAGGTTTG
GAGCTTAATCCAAATCTGCTCCTTTGCAAATTTAGGTTTCTGATTTTATATACATAAATAAGTGCATCGTAGTTGTTGTTCAGACTCCTT
CTACATGCGCCGAACAATTACTGTGTTGTATTCCTTGGAGTGACTAAGTGTCCGTTATTTTTTTTCCCAGTAATGCTTACACGAACATAC
AGTTTTTTTAGTGCAATTTGAGCTGAAGAGTTTGATTATTGCATTGCAGGTATTTGTATTTTTGTTTGCCTGGTTTGAGGGAATTGCAC
TCGGAAGTTCACCCCAAGATCGGATTCAAATTTTT

```
Alignment of Sequence_1:  [Untitled Sequence #1] with  Sequence_2: [Sequence Window #2]

Similarity : 2720/3026 (89.89 %)

Seq_1  1      TTTTGGGCGGTGGAGTGAGTGTGAAGGTACTAGCTAGAATTAACGACCCCACTGCCATTG  60

Seq_2  1      ------------------------------------------------------------  0


Seq_1  61     CCATTTTGCTCGGGCCGAAAAACGGAGAGAAGAGACGAACGAGTCCCCCACCGCGCGCAT  120

Seq_2  1      ------------------------------------------------------------  0


Seq_1  121    GCAGTAGGTGGGCGTGGCTCGGCATGTGAGCCTTTTACCAACCTCTGTCGGCTTGCTCTC  180

Seq_2  1      ------------------------------------------------------------  0


Seq_1  181    ACATCCACTGCTTAGCTAAGGATGCTACTACCTAGTCTTAGAGTACTGCTGCTAACTAAT  240

Seq_2  1      ------------------------------------------------------------  0


Seq_1  241    CCAATTATCCAGTCCACCCGTGCCAAGATGCTGCTGTGCCTGTGCTGCTGGTATTTTACT  300
                                                                |||||||||| 
Seq_2  1      --------------------------------------------------GTATTTTACT  10


Seq_1  301    GTTGCAGTGCTCCTGTAGACTGTGGACTGTAGTATGTACTCTTCCCCGGTTCTCTTGGGC  360
              ||||||||||||||||||||||||||||||||||||||||||||||||||||||||||||
Seq_2  11     GTTGCAGTGCTCCTGTAGACTGTGGACTGTAGTATGTACTCTTCCCCGGTTCTCTTGGGC  70


Seq_1  361    TTGGGGTCGGGTTTATTTATGTACCGATCCCCGCGCTTCACGGGCAAGCGAATGACGCCG  420
```

```
             ||||||||||||||||||||||||||||||||||||||||||||||||||||||||||||
Seq_2  71    TTGGGGTCGGGTTTATTTATGTACCGATCCCCGCGCTTCACGGGCAAGCGAATGACGCCG  130

Seq_1  421   GCCGGGGCGCACCGGTGTCGCATGCGTCGTCTCG------------------GCACGCAG  462
             |||||||||| |||||||||||||||||||||||                  ||||||||
Seq_2  131   GCCGGGGCGCGCCGGTGTCGCATGCGTCGTCTCGTCTGCTTCGATCGTCTCGGCACGCAG  190

Seq_1  463   ATGCGTCCCATTCCCAACACGCTTCCGTGCCTCCGTCCCATGGCGTTTGCCCGTCAAATC  522
             |||| |||||||||||||||||||||||||||||||||||||||||||||||||||||||
Seq_2  191   ATGCATCCCATTCCCAACACGCTTCCGTGCCTCCGTCCCATGGCGTTTGCCCGTCAAATC  250

Seq_1  523   ACGGAACTCGCCCATCCATTCCCGACGGAGATGGGGCTTTACAATGCTGGCACGGGCCTT  582
             |||||||||||||||| |||||||||||||||||||||||||||||||||||||| ||||
Seq_2  251   ACGGAACTCGCCCATCAATTCCCGACGGAGATGGGGCTTTACAATGCTGGCACGGACCTT  310

Seq_1  583   GGTTGTTGGACGAACAAAAACGGAGAGGCAATTCTCGATTCTATTAATTCGGCCGGTGTT  642
             ||||||||||||||||||||||||||||||||||||||||||||||||||||||||||||
Seq_2  311   GGTTGTTGGACGAACAAAAACGGAGAGGCAATTCTCGATTCTATTAATTCGGCCGGTGTT  370

Seq_1  643   TTTAGGTGAGAGTGATAGCACTAGTATTAACACGTATACTGGTGGATCGGGGGCGGGGGA  702
             ||||||||||||||||||||||||||||||||||||||||||||||||||||||||||||
Seq_2  371   TTTAGGTGAGAGTGATAGCACTAGTATTAACACGTATACTGGTGGATCGGGGGCGGGGGA  430

Seq_1  703   TGGAATCTAATGGCGTACGTCCCCAGGTCTGGTCCGGTACTTGGATCTCCAGCGACGTTG  762
             ||||||||||||||||||||||||||||||||||||||||||||||||||||||||||||
Seq_2  431   TGGAATCTAATGGCGTACGTCCCCAGGTCTGGTCCGGTACTTGGATCTCCAGCGACGTTG  490

Seq_1  763   TACAGATGGCACCCAGTCGGCCAGTCCATTTGGCCTGGCACGACGTACGTACGCACGCCA  822
             ||||||||||||||||||||||||||||||||||||||||||||||||||||||||||||
Seq_2  491   TACAGATGGCACCCAGTCGGCCAGTCCATTTGGCCTGGCACGACGTACGTACGCACGCCA  550

Seq_1  823   TGCATGGGCATGGTGGCCTGACGATTCGTTGGCAATGCTTTGTACGCTACTCCTACCTAA  882
             ||||||||||||||||||||||||||||||||| ||||||||||||||||||||||||||
Seq_2  551   TGCATGGGCATGGTGGCCTGACGATTCGTTGGCGATGCTTTGTACGCTACTCCTACCTAA  610

Seq_1  883   CCCGATAAAACCATAAAGGTCATCATCATAAACAAAAGGCCATCGATCATGCCGTGACAC  942
             ||||||||||||||||||||||||||||||||||||||||||||||||||||||||||||
Seq_2  611   CCCGATAAAACCATAAAGGTCATCATCATAAACAAAAGGCCATCGATCATGCCGTGACAC  670

Seq_1  943   TATACACTGGAGTACTAGTAGTACTATCTCGTTTCTGTACTGGCCGTCAGGAAAGAAAGA  1002
             ||||||||||||||||||||||||||||||||||||||||||||||||||||||||||||
Seq_2  671   TATACACTGGAGTACTAGTAGTACTATCTCGTTTCTGTACTGGCCGTCAGGAAAGAAAGA  730

Seq_1  1003  TCTCATTCCCGTACTGAATGACTGTAATCAGCACGCTCGGAGTTTTCCGAGCTTCTGCAG  1062
             ||||||||||||||||||||||||||||||||||||||||||||||||||||||||||||
Seq_2  731   TCTCATTCCCGTACTGAATGACTGTAATCAGCACGCTCGGAGTTTTCCGAGCTTCTGCAG  790

Seq_1  1063  ACACGGAAATTGGATTGCTAGAAATCAGCACGCTCGGAGTTTAACTCTCGAAATTAAGTA  1122
             ||||||||||||||||||||||||||||||||||||||||||||||||||||||||||||
Seq_2  791   ACACGGAAATTGGATTGCTAGAAATCAGCACGCTCGGAGTTTAACTCTCGAAATTAAGTA  850

Seq_1  1123  GGTTCGGAGGAAGAGGTTACCTCGGTCCAAAGCACTTTTGGTGCGCTGAAAATTGTCGGC  1182
             ||||||||||||||||||||||||| ||||||||||||||||||||||||||||||||||
Seq_2  851   GGTTCGGAGGAAGAGGTTACCTCGGCCCAAAGCACTTTTGGTGCGCTGAAAATTGTCGGC  910

Seq_1  1183  TGTTTGGATCGGACTTAAATAAAATGATATCCGGTGCGGTCTATTCTCTCTCACGACCAA  1242
```

```
                ||||||||||||||||||||||||||||||||||||||||||||||||||||||||||||
Seq_2  911      TGTTTGGATCGGACTTAAATAAAATGATATCCGGTGCGGTCTATTCTCTCTCACGACCAA  970

Seq_1  1243     ACCATGAAGCTACCGTGCACCGCTAGTAGTACTAGTGTCACCCCCCTGACCTGACTCCCG  1302
                |||||||||||||||||||||||||||||||| ||| |||||||||||||||||||||||
Seq_2  971      ACCATGAAGCTACCGTGCACCGCTAGTAGTACAAGTATCACCCCCCTGACCTGACTCCCG  1030

Seq_1  1303     ACCAAGTGTGATGCAGCCTGCTTTCCCGTCCATTCAGCCGTGCATGTTGCTTTAGCCGAT  1362
                ||||||||||||||||||||||||||||||||||||||||||||||||||||||||||||
Seq_2  1031     ACCAAGTGTGATGCAGCCTGCTTTCCCGTCCATTCAGCCGTGCATGTTGCTTTAGCCGAT  1090

Seq_1  1363     GAGACAACACCTGTCTGTACGGGGATCTGACCGTGACAGTGAGGTAGCACCGGTTGATGA  1422
                ||||||||||||||||||||||||||||||||||||||||||||||||||||||||||||
Seq_2  1091     GAGACAACACCTGTCTGTACGGGGATCTGACCGTGACAGTGAGGTAGCACCGGTTGATGA  1150

Seq_1  1423     TTGGGGTTGGTACGTCCTAGGACGCTTTTAAAGGACGACGATTTTTGGTCCCATCCATCC  1482
                ||||||||||||||||||||||||||||||||||||||||||||||||||||||||||||
Seq_2  1151     TTGGGGTTGGTACGTCCTAGGACGCTTTTAAAGGACGACGATTTTTGGTCCCATCCATCC  1210

Seq_1  1483     AGCCAGCTCGATGCTCCGTCCGTGACAGTGTTACGACTTGACTGTAAACGGCAGGTCTTC  1542
                ||||||||||||||||||||||||||||||||||||||||||||||||||||||||||||
Seq_2  1211     AGCCAGCTCGATGCTCCGTCCGTGACAGTGTTACGACTTGACTGTAAACGGCAGGTCTTC  1270

Seq_1  1543     CTCGTCGGTCGGTCGGTCAGTGCGATGTGGTGTTTCGACACGCACGCGCGCTGTGCAGCA  1602
                ||||||||||||||||||||||||||||||||||||||||||||||||||||||||||||
Seq_2  1271     CTCGTCGGTCGGTCGGTCAGTGCGATGTGGTGTTTCGACACGCACGCGCGCTGTGCAGCA  1330

Seq_1  1603     GGCTGCTATGCTACACTTGCAGCACGAGAGGCCTCGCGGCGCCTTTCGTTGCCGGATCTC  1662
                ||||||||||||||||||||||||||||||||||||||||||||||||||||||||||||
Seq_2  1331     GGCTGCTATGCTACACTTGCAGCACGAGAGGCCTCGCGGCGCCTTTCGTTGCCGGATCTC  1390

Seq_1  1663     GGTGGATCGCCCCTCCCAAAACAAGGAGGACTCCCTCAGTGCATGCCACAACCACCCCGT  1722
                ||||||||||||||||||||||||||| ||||||||||||||||||||||||||||||||
Seq_2  1391     GGTGGATCGCCCCTCCCAAAACAAGGACGACTCCCTCAGTGCATGCCACAACCACCCCGT  1450

Seq_1  1723     ------------------------------------------------------------  1722

Seq_2  1451     TAATTAGGCCCAAGTGTCGATGACAATCCGTCCACGTCGAATCGAGGGCGATCAATCCAG  1510

Seq_1  1723     ------------------------------------------------------------  1722

Seq_2  1511     ATCCAGAGTGGGCTAGGACATCAAAGACTCCCCGATGTCATCCAAGAATACCGCATTAAG  1570

Seq_1  1723     ------------------------------------------------------------  1722

Seq_2  1571     ACGGGAATTCAACCGTGACACTTTTATATTAGATGGAAACCCTTTTTATCAGCGAGCGGC  1630

Seq_1  1723     ------------------------------------------------------------  1722

Seq_2  1631     CTCTACACCATCCTAAACATAGGCAAAACTGTACTATACTGGTAACAGAGCTTTCGTGAC  1690

Seq_1  1723     -----------------------------AATTAGGCCGGAATACCAAGGAAGAGTTTGG  1753
                                             |||||||||||||||||||||||||||||||
Seq_2  1691     TCGTCTTCTCTGCAGAGTGCAGACCCCGTAATTAGGCCGGAATACCAAGGAAGAGTTTGG  1750

Seq_1  1754     ACGAAACGGAAGGCCCAAGCTTGATAGCGACTATGCGCATTGATCGGCCTTGTCAGCAGC  1813
```

```
                 ||||||||||||||||||||||||||||||||| ||||||||||||||||||||||||||
Seq_2  1751  ACGAAACGGAAGGCCCAAGCTTGATAGCGACTAGGCGCATTGATCGGCCTTGTCAGCAGC  1810

Seq_1  1814  TAGCAGCCTGGCCCAGAGAAGAACAAAGCAGATGGATTCCCACTCGCCCGGCCCGTATCG  1873
             ||||||||||||||||||||||||||||||||||||||||||||||||||||||||||| 
Seq_2  1811  TAGCAGCCTGGCCCAGAGAAGAACAAAGCAGATGGATTCCCACTCGCCCGGCCCGTATCA  1870

Seq_1  1874  ATCGGCCCATTCAAGAAGCCAGAAAAAGGAAGCAGCAAGCACGAGCCCCTTGCGTTGGTA  1933
             ||||||||||||||||||||||||||||||||||||||||||||| ||||||||||||||
Seq_2  1871  ATCGGCCCATTCAAGAAGCCAGAAAAAGGAAGCAGCAAGCACGAGTCCCTTGCGTTGGTA  1930

Seq_1  1934  GGCACCGACGGTCCAGATCGGGAGCAAGGACACATCCGGACCGTTCGTTCCTGCGCCAGC  1993
             ||||||||||||||||||||||||||||||||||||| ||||||||||||||||||||||
Seq_2  1931  GGCACCGACGGTCCAGATCGGGAGCAAGGACACATCCCGACCGTTCGTTCCTGCGCCAGC  1990

Seq_1  1994  ACGGGCGGTGCAGACGAAGCGTCCCGTCTCTTGGCTCCCAGCGGCCGCAATTTTCCACGA  2053
             |||||||||||||||||||||||||||||||||||||| |||||||||||||||||||||
Seq_2  1991  ACGGGCGGTGCAGACGAAGCGTCCCGTCTCTTGGCTCCAAGCGGCCGCAATTTTCCACGA  2050

Seq_1  2054  GGAGAAAAAAAGGGGTAGAAGGGGAAGTTTCCAAGCGAAATCGAGATCGGAGGCACAGCT  2113
             ||||||||||||||||||||||||||||||||||||||||||||||||||||||||||||
Seq_2  2051  GGAGAAAAAAAGGGGTAGAAGGGGAAGTTTCCAAGCGAAATCGAGATCGGAGGCACAGCT  2110

Seq_1  2114  AGAAGGTGATTTCTTCCGCTCAATCTCTTGTTCCGTACTATTAGTTTGATTAGCTAATTT  2173
             ||||||||||||||||||||||||||||||||||||||||||||||||||||||||||||
Seq_2  2111  AGAAGGTGATTTCTTCCGCTCAATCTCTTGTTCCGTACTATTAGTTTGATTAGCTAATTT  2170

Seq_1  2174  CACTAGGCATTCTGGTGCGCGATGTTTAGCCATGCCTCCTCGATTCGATAGGAACTTGCG  2233
             ||||||||||||||||||||||||||||||||||||||||||||||||||||||||||||
Seq_2  2171  CACTAGGCATTCTGGTGCGCGATGTTTAGCCATGCCTCCTCGATTCGATAGGAACTTGCG  2230

Seq_1  2234  GTTTCTTCTGCCGTTCTGTCGCCTTGCAGTACTATTTGCTCGTCAAAGGATGCATTTTTT  2293
             ||||||||||||||||||||||||||||||||||||||||||||||||||||||||||||
Seq_2  2231  GTTTCTTCTGCCGTTCTGTCGCCTTGCAGTACTATTTGCTCGTCAAAGGATGCATTTTTT  2290

Seq_1  2294  TTATTTTGTTGTTGGTGTCTTCGATGATGTAGAGAGCTAAAACAGCGGTTTCCCGCGACT  2353
             ||||||||||||||||||||||||||||||||||||||||||||||||||||||||||||
Seq_2  2291  TTATTTTGTTGTTGGTGTCTTCGATGATGTAGAGAGCTAAAACAGCGGTTTCCCGCGACT  2350

Seq_1  2354  CCTCGGCGTAGGTTATTGGATTTGGTTGCGACTTAGGGTTTCGCGTCTGTATTGGCCCAG  2413
             ||||||||||||||||||||||||||||||||||||||||||||||||||||||||||||
Seq_2  2351  CCTCGGCGTAGGTTATTGGATTTGGTTGCGACTTAGGGTTTCGCGTCTGTATTGGCCCAG  2410

Seq_1  2414  AATCGTTGGATCTGTTCCTCATGTTTCGACTAGGTTTGCTTGATTTCATGGCCTTGTCTG  2473
             ||||||||||||||||||||||||||||||||||||||||||||||||||||||||||||
Seq_2  2411  AATCGTTGGATCTGTTCCTCATGTTTCGACTAGGTTTGCTTGATTTCATGGCCTTGTCTG  2470

Seq_1  2474  CTTAATTGGTCGAATTGATATAAAATCTATGCTGCTCGCTTTGTATAGTCTGCGGTACTC  2533
             ||||||||||||||||||||||||||||||||||||||||||||||||||||||||||||
Seq_2  2471  CTTAATTGGTCGAATTGATATAAAATCTATGCTGCTCGCTTTGTATAGTCTGCGGTACTC  2530

Seq_1  2534  TATGCTTCAATTGAAGCCAATTTTCAGCTTATCTTTCATGCTTGGTTCAGGAGTTGCCTC  2593
             ||||||||||||||||||||||||||||||||||| ||||||||||||||||||||||||
Seq_2  2531  TATGCTTCAATTGAAGCCAATTTTCAGCTTATCTTGCATGCTTGGTTCAGGAGTTGCCTC  2590

Seq_1  2594  TTGTTGTGCAGGTTGATGCTTTTAATTTCGGTGCAAAAGTAGAGGGTGATTTACTCTTTT  2653
```

```
               ||||||||||||||||||||||||||||||||||||||||||||||||||||||||||||
Seq_2  2591  TTGTTGTGCAGGTTGATGCTTTTAATTTCGGTGCAAAAGTAGAGGGTGATTTACTCTTTT  2650


Seq_1  2654  GAACAAACCTGGTCTATCTTGATCTTATTACAAGTTTCATCTTCAACTGAAAATCTGGTT  2713
             ||||||||||||||||||||||||||||||||||||||||||||||||||||||||||||
Seq_2  2651  GAACAAACCTGGTCTATCTTGATCTTATTACAAGTTTCATCTTCAACTGAAAATCTGGTT  2710


Seq_1  2714  ACAGGTTTGGAGCTTAATCCAAATCTGCTCCTTTGCAAATTTAGGTTTCTGATTTTATAT  2773
             ||||||||||||||||||||||||||||||||||||||||||||||||||||||||||||
Seq_2  2711  ACAGGTTTGGAGCTTAATCCAAATCTGCTCCTTTGCAAATTTAGGTTTCTGATTTTATAT  2770


Seq_1  2774  ACATAAATAAGTGCATCGTAGTTGTTGTTCAGACTCCTTCTACATGCGCTGAACAATTAC  2833
             ||||||||||||||||||||||||||||||||||||||||||||||||| ||||||||||
Seq_2  2771  ACATAAATAAGTGCATCGTAGTTGTTGTTCAGACTCCTTCTACATGCGCCGAACAATTAC  2830


Seq_1  2834  TGTGTTGTATTCCTTGGAGTGACTAAGTGTCCGTTATTTTTTTTCCCAGTAATGCTTACA  2893
             ||||||||||||||||||||||||||||||||||||||||||||||||||||||||||||
Seq_2  2831  TGTGTTGTATTCCTTGGAGTGACTAAGTGTCCGTTATTTTTTTTCCCAGTAATGCTTACA  2890


Seq_1  2894  CGAACATACAGTTTTTTTAGTGCAATTTGAGCTGAAGAGTTTGATTATTGCATTGCAGGT  2953
             ||||||||||||||||||||||||||||||||||||||||||||||||||||||||||||
Seq_2  2891  CGAACATACAGTTTTTTTAGTGCAATTTGAGCTGAAGAGTTTGATTATTGCATTGCAGGT  2950


Seq_1  2954  ATTTGTATTTTTGTTTGCCTGGTTTGAGGGAATTGCACTCGGAAGTTCACCCCAAGATCG  3013
             ||||||||||||||||||||||||||||||||||||||||||||||||||||||||||||
Seq_2  2951  ATTTGTATTTTTGTTTGCCTGGTTTGAGGGAATTGCACTCGGAAGTTCACCCCAAGATCG  3010


Seq_1  3014  GATTCAAATTTTT  3026
             |||||||||||||
Seq_2  3011  GATTCAAATTTTT  3023
```
