# Supplementary material for: Never the Two Shall Mix: Robust Indel Markers to Ensure the Fidelity of Two Pivotal and Closely-Related Accessions of Brachypodium distachyon
Source: Plants (Basel). 2019 Jun 6;8(6):153. doi: 10.3390/plants8060153 (PMC6630600; doi:10.3390/plants8060153)
Supplement: Supplementary file 1 [file plants-08-00153-s001.zip › Figure S2.pdf]

Figure S2. Observed amplicon sizes of indel markers on six *Brachypodium distachyon* accessions. The PCR results were visualized on 1.5% agarose gels stained with Ethidium bromide. M1 represents the GoldBio® 50 bp DNA ladder and M2 represents the GoldBio® 1 kb PLUS™ DNA ladder.

<table>
<tr><th>Marker Name</th><th>Results on 1.5% Agarose</th></tr>
<tr><td>BdindelWSU_1</td><td>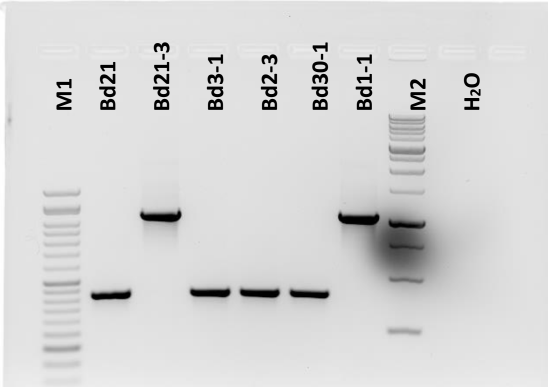
</td></tr>
<tr><td>BdindelWSU_2</td><td>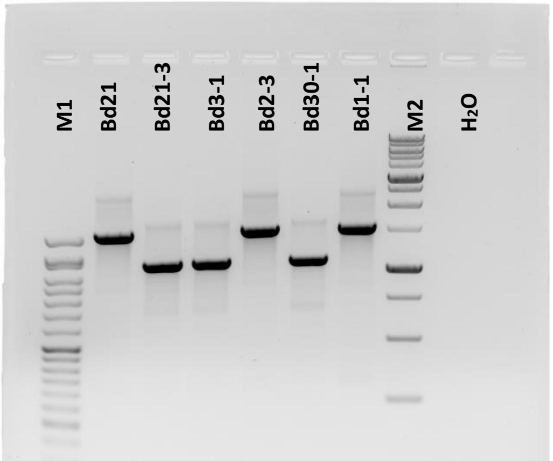
</td></tr>
<tr><td>BdindelWSU_3</td><td>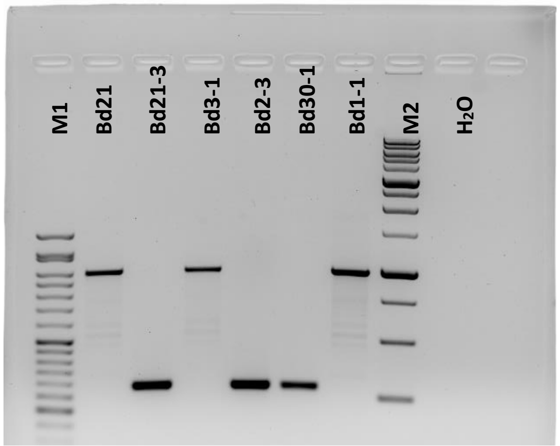
</td></tr>
</table>

| BdindelWSU_6 | BdindelWSU_5 | BdindelWSU_4 |
|---|---|---|
| 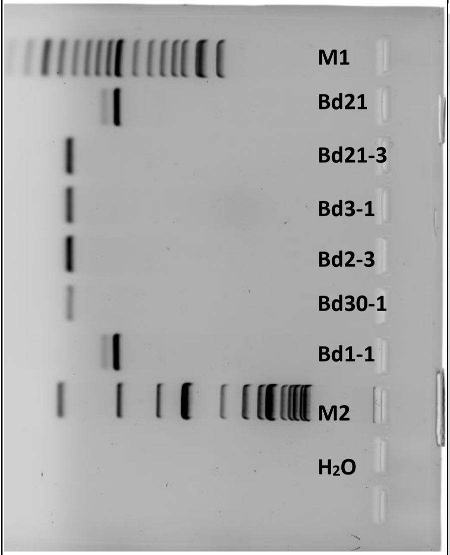<br> | 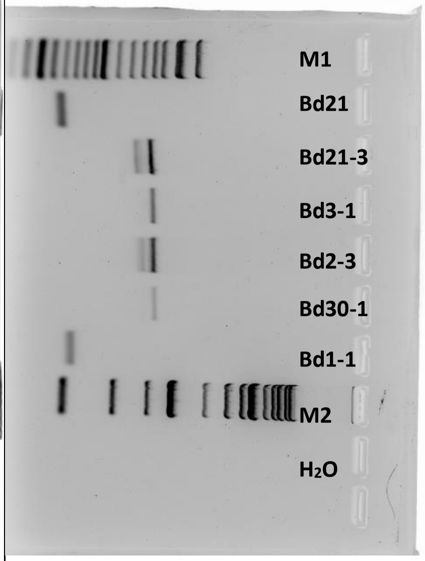<br> | 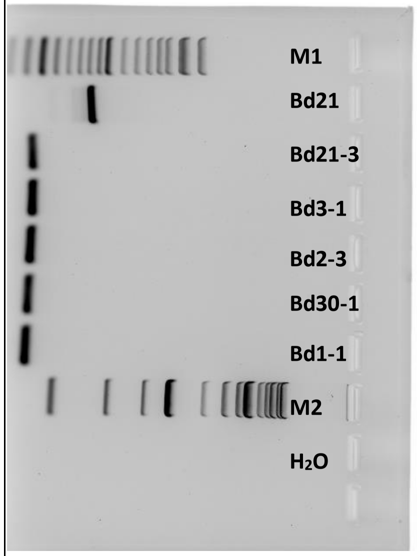<br> |

| BdindelWSU_9 | BdindelWSU_8 | BdindelWSU_7 |
| --- | --- | --- |
| 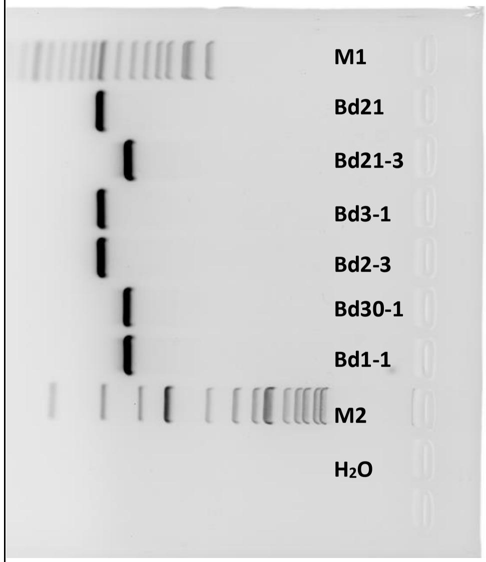 | 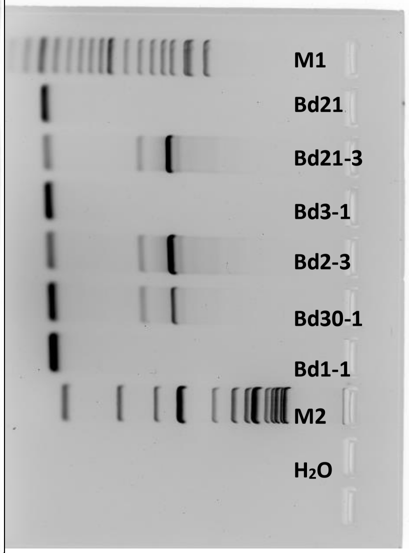 | 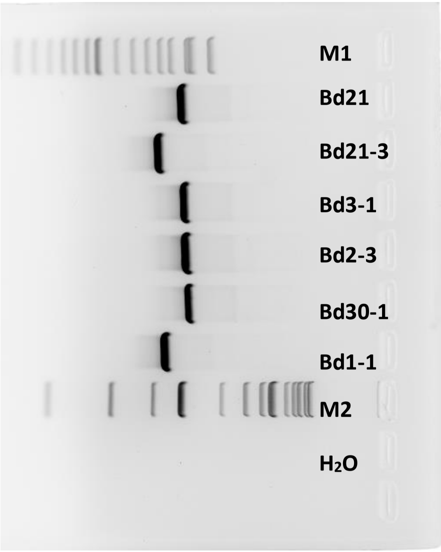 |

| BdindelWSU_12 | BdindelWSU_11 | BdindelWSU_10 |
| --- | --- | --- |
| 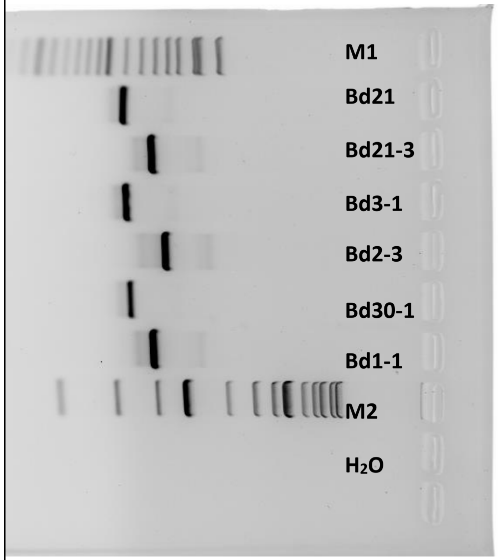  | 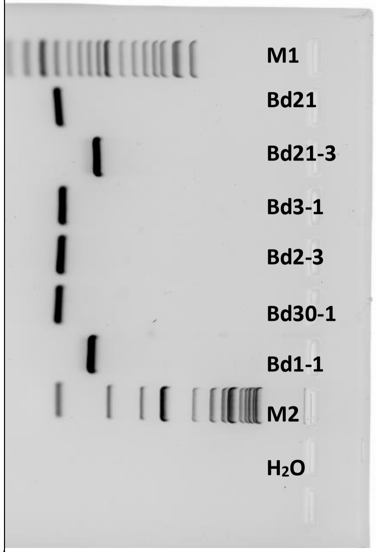  | 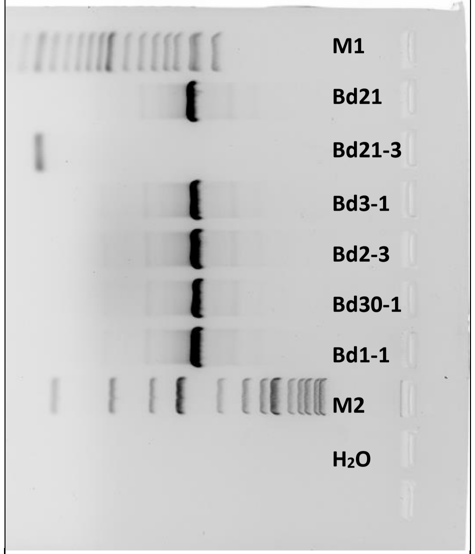  |

**BdindelWSU_16**

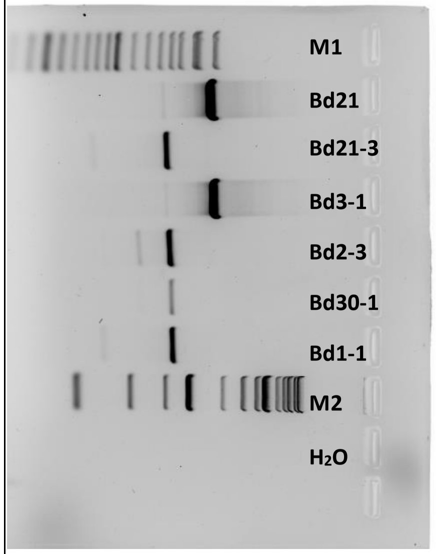


**BdindelWSU_15**

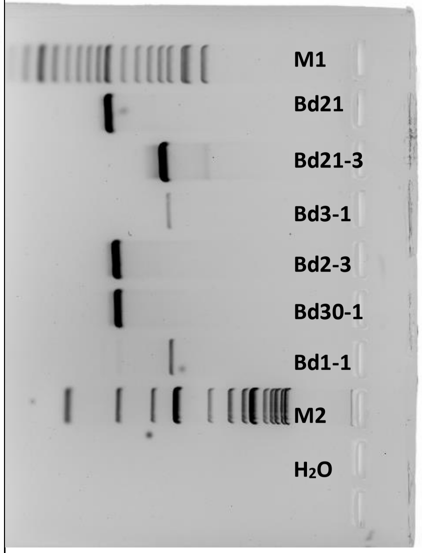


**BdindelWSU_14**

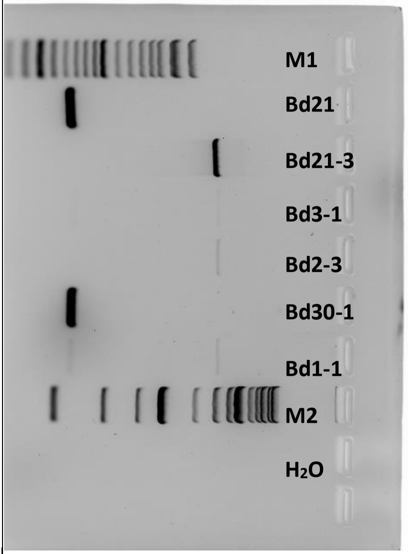


**BdindelWSU_13**

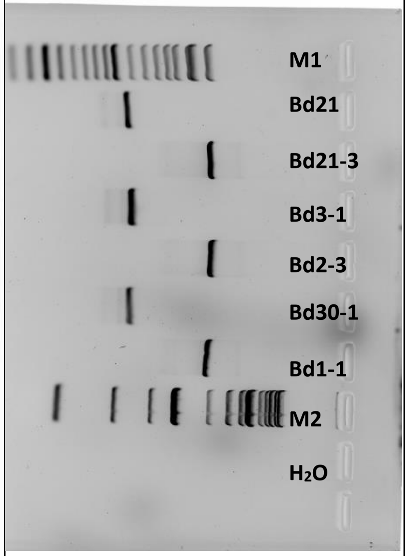

| BdindelWSU_20 | BdindelWSU_19 | BdindelWSU_18 | BdindelWSU_17 |
| --- | --- | --- | --- |
| 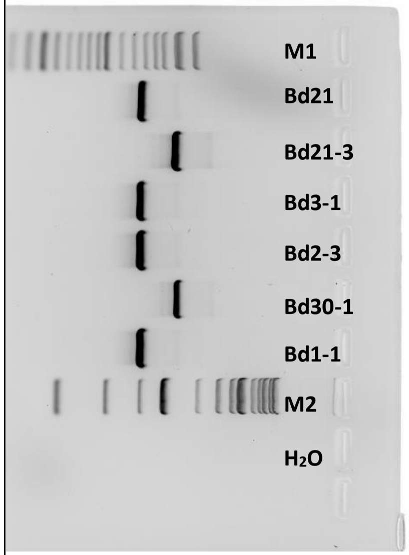 | 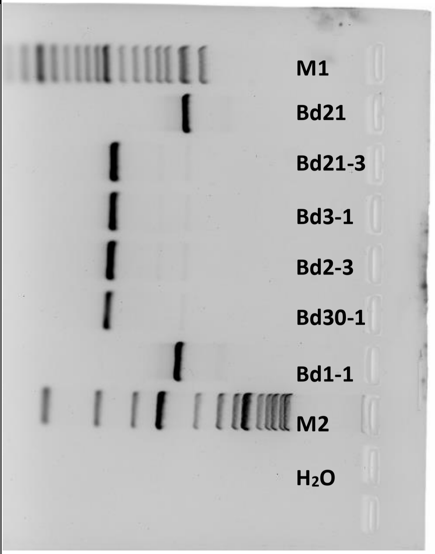 | 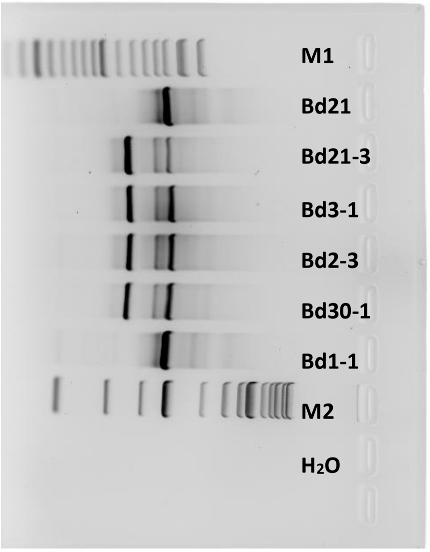 | 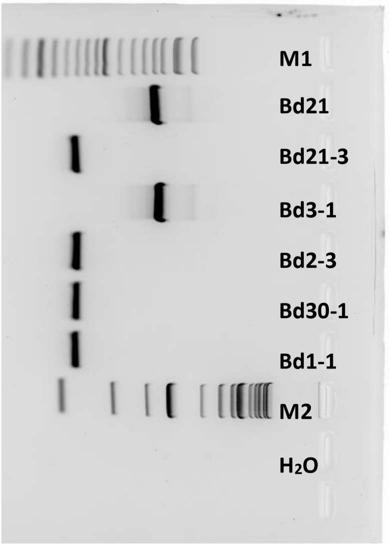 |

| BdindelWSU_22 | BdindelWSU_21 |
| --- | --- |
| 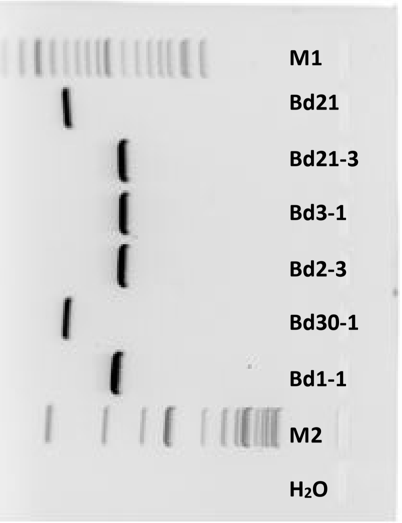  | 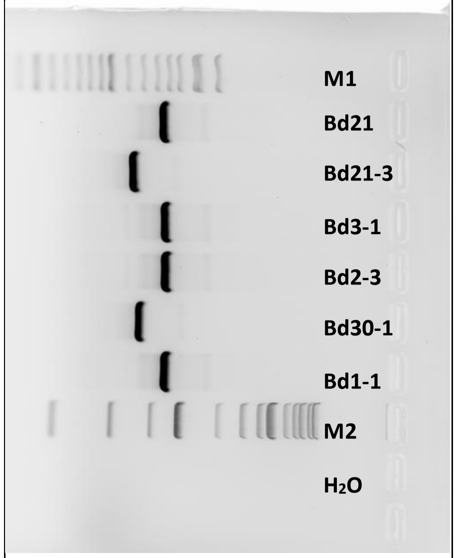  |
